# Supplementary material for: Analysis of Selective Pressure on Ancient Human Mitochondrial Genomes Reveals the Presence of Widespread Sequencing Artefacts
Source: Int J Mol Sci. 2025 Aug 11;26(16):7739. doi: 10.3390/ijms26167739 (PMC12386754; doi:10.3390/ijms26167739)
Supplement: Supplementary file 1 [file ijms-26-07739-s001.zip › ijms-3768908-supplementary.pdf]

Supplementary data

Group per Tree Comparison – R0

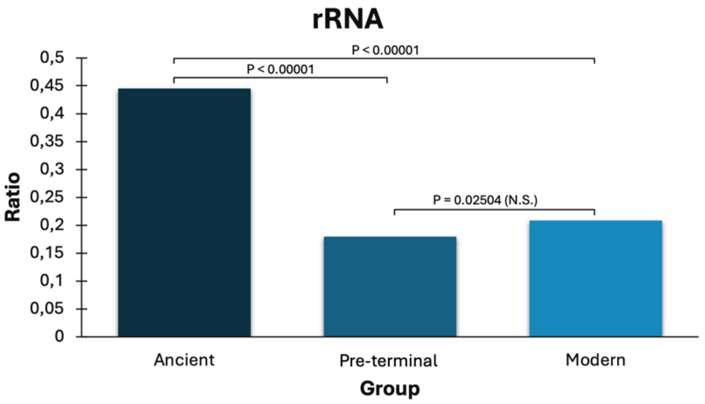

Figure S1. Comparison of the ratio of rRNA mitochondrial mutations measured against synonymous mutations per individual haplogroup, which in this case is **R0**. *P*-values between ratios in the three analysed groups are shown above the bars, where N.S. corresponds to non-significant.

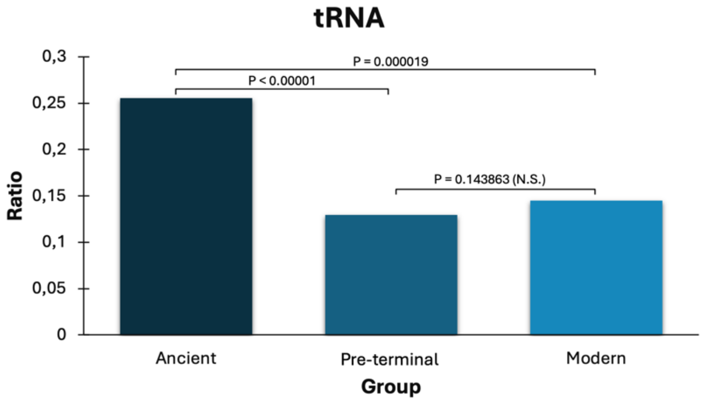

Figure S2. Comparison of the ratio of tRNA mitochondrial mutations measured against synonymous mutations per individual haplogroup, which in this case is **R0**. *P*-values between ratios in the three analysed groups are shown above the bars, where N.S. corresponds to non-significant.

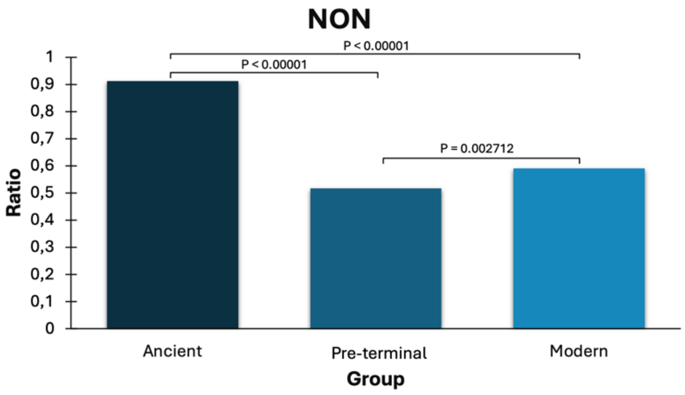

Figure S3. Comparison of the ratio of mitochondrial non-synonymous mutations measured against synonymous mutations per individual haplogroup, which in this case is **R0**. *P*-values between ratios in the three analysed groups are shown above the bars, where N.S. corresponds to non-significant.

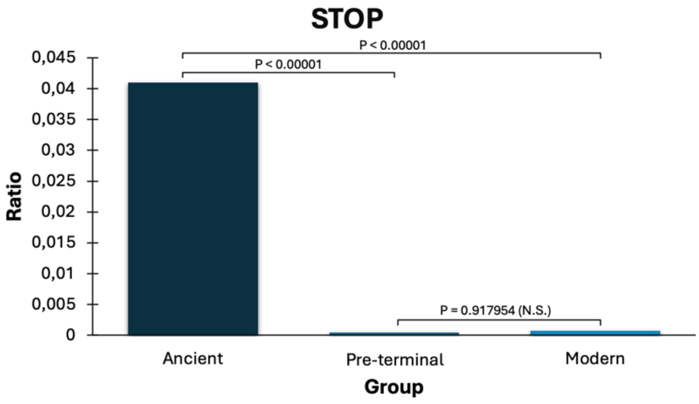

Figure S4. Comparison of the ratio of mitochondrial nonsense mutations measured against synonymous mutations per individual haplogroup, which in this case is **R0**. *P*-values between ratios in the three analysed groups are shown above the bars, where N.S. corresponds to non-significant.

Group per Tree Comparison – U

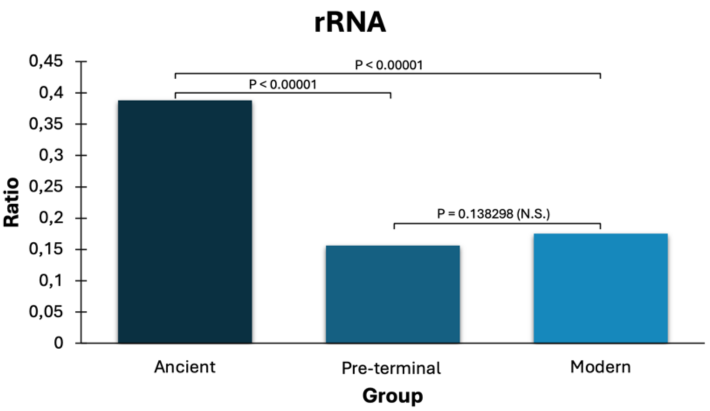

Figure S5. Comparison of the ratio of rRNA mitochondrial mutations measured against synonymous mutations per individual haplogroup, which in this case is **U**. *P*-values between ratios in the three analysed groups are shown above the bars, where N.S. corresponds to non-significant.

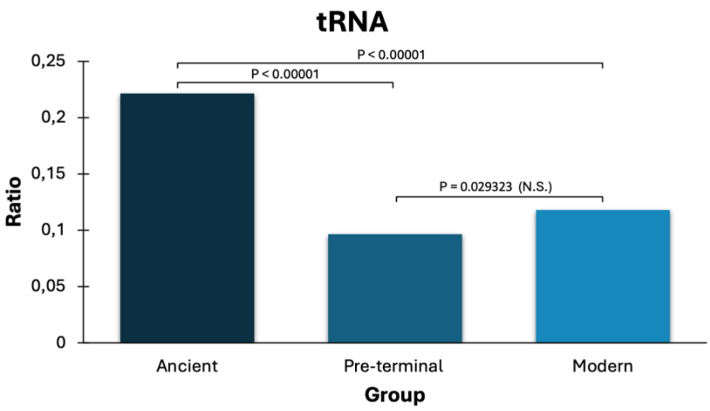

Figure S6. Comparison of the ratio of tRNA mitochondrial mutations measured against synonymous mutations per individual haplogroup, which in this case is **U**. *P*-values between ratios in the three analysed groups are shown above the bars, where N.S. corresponds to non-significant.

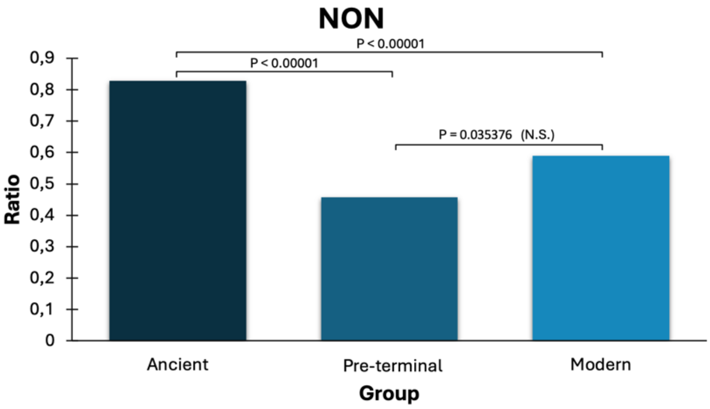

Figure S7. Comparison of the ratio of mitochondrial non-synonymous mutations measured against synonymous mutations per individual haplogroup, which in this case is **U**. *P*-values between ratios in the three analysed groups are shown above the bars, where N.S. corresponds to non-significant.

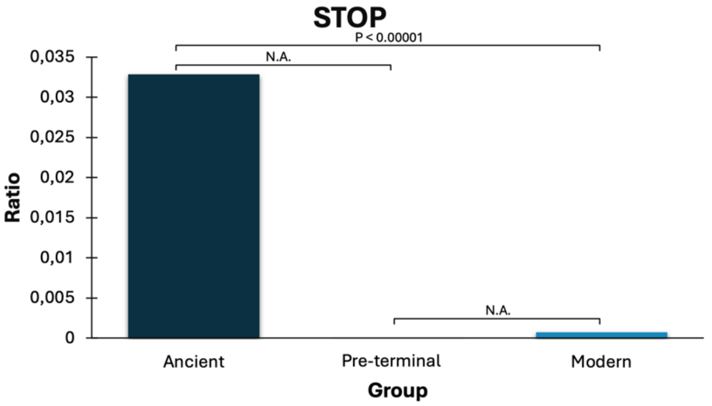

Figure S8. Comparison of the ratio of mitochondrial nonsense mutations measured against synonymous mutations per individual haplogroup, which in this case is **U**. *P*-values between ratios in the three analysed groups are shown above the bars, where N.S. corresponds to non-significant.

Group per Tree Comparison – JT

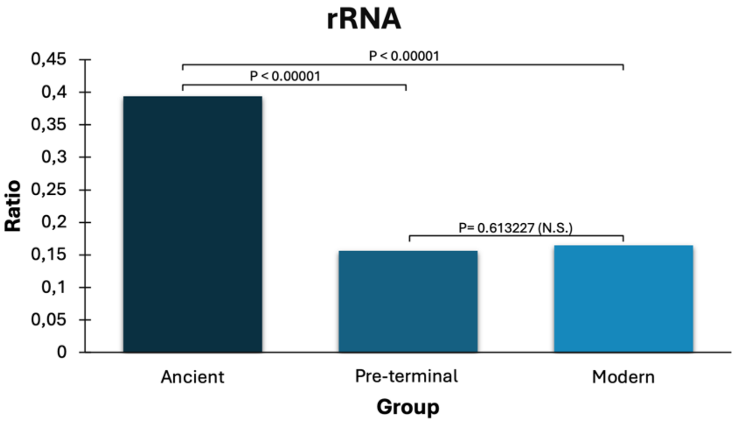

Figure S9. Comparison of the ratio of rRNA mitochondrial mutations measured against synonymous mutations per individual haplogroup, which in this case is **JT**. *P*-values between ratios in the three analysed groups are shown above the bars, where N.S. corresponds to non-significant.

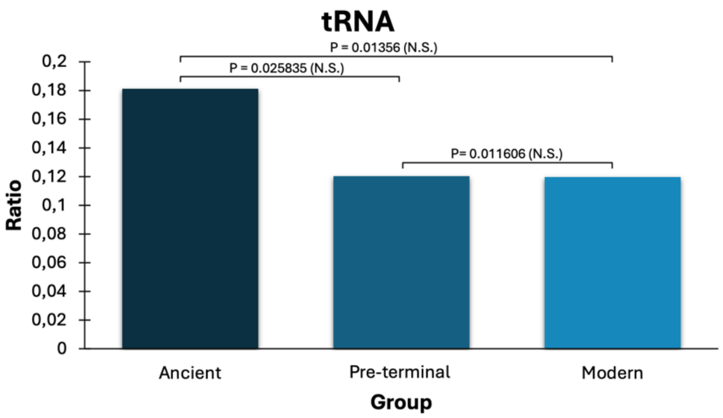

Figure S10. Comparison of the ratio of tRNA mitochondrial mutations measured against synonymous mutations per individual haplogroup, which in this case is **JT**. *P*-values between ratios in the three analysed groups are shown above the bars, where N.S. corresponds to non-significant.

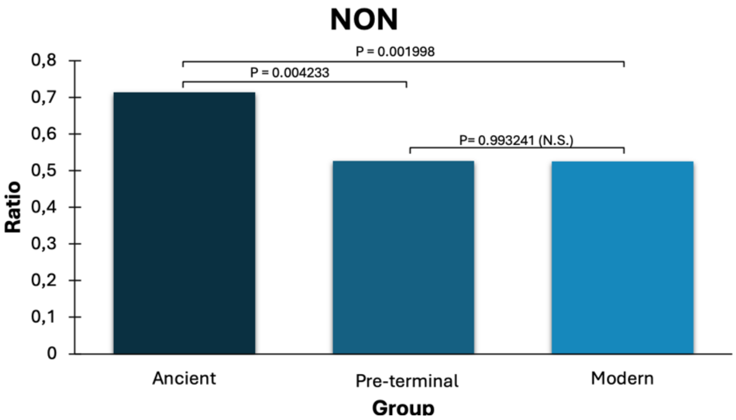

Figure S11. Comparison of the ratio of mitochondrial non-synonymous mutations measured against synonymous mutations per individual haplogroup, which in this case is **JT**. *P*-values between ratios in the three analysed groups are shown above the bars, where N.S. corresponds to non-significant.

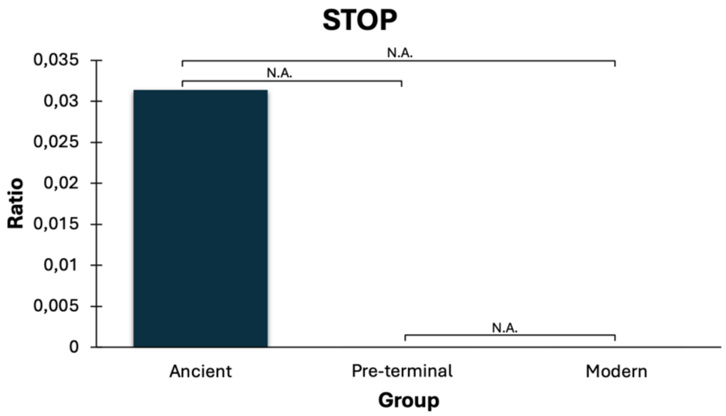

Figure S12. Comparison of the ratio of mitochondrial nonsense mutations measured against synonymous mutations per individual haplogroup, which in this case is **JT**. *P*-values between ratios in the three analysed groups are shown above the bars, where N.S. corresponds to non-significant.

Group per Tree Comparison – N1

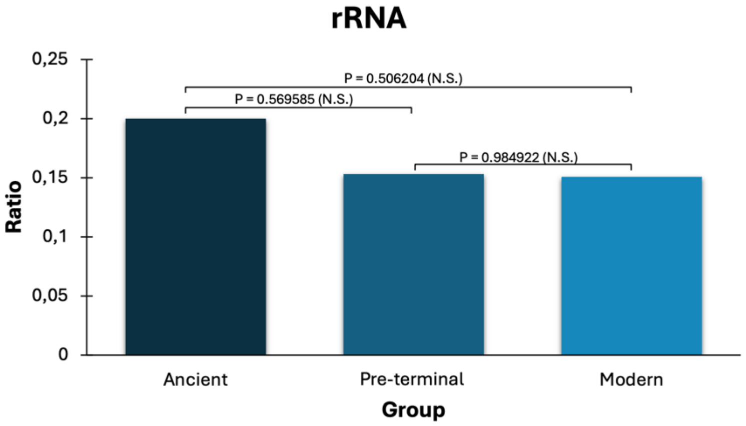

Figure S13. Comparison of the ratio of rRNA mitochondrial mutations measured against synonymous mutations per individual haplogroup, which in this case is **N1**. *P*-values between ratios in the three analysed groups are shown above the bars, where N.S. corresponds to non-significant.

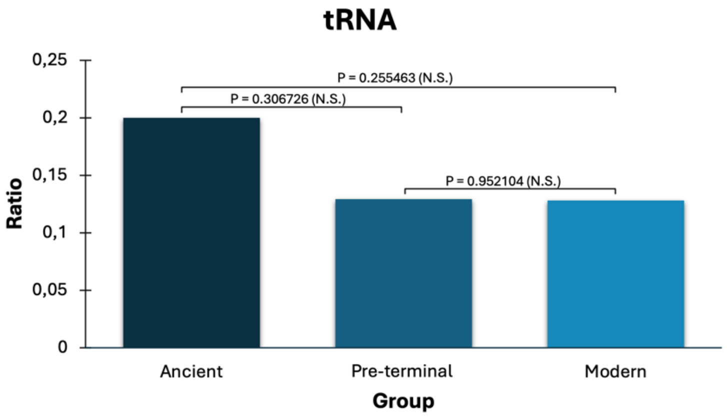

Figure S14. Comparison of the ratio of tRNA mitochondrial mutations measured against synonymous mutations per individual haplogroup, which in this case is **N1**. *P*-values between ratios in the three analysed groups are shown above the bars, where N.S. corresponds to non-significant.

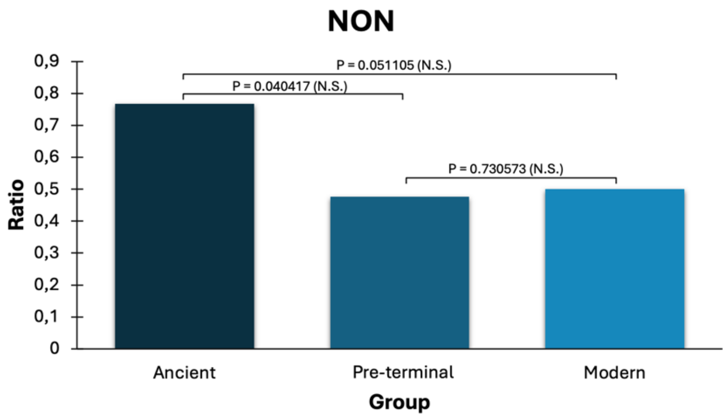

Figure S15. Comparison of the ratio of mitochondrial non-synonymous mutations measured against synonymous mutations per individual haplogroup, which in this case is **N1**. *P*-values between ratios in the three analysed groups are shown above the bars, where N.S. corresponds to non-significant.

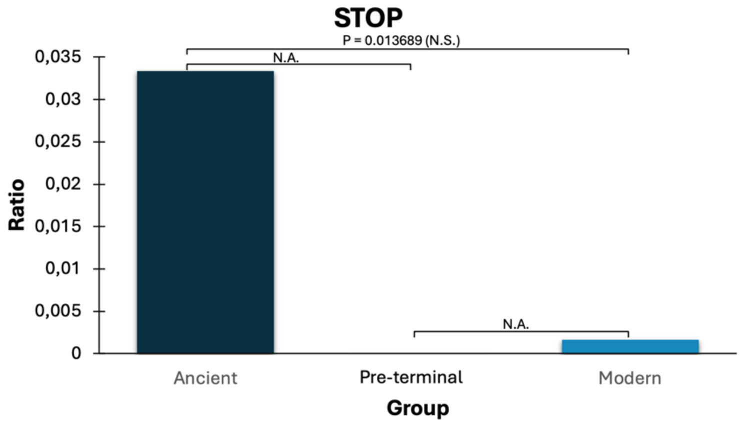

Figure S16. Comparison of the ratio of mitochondrial nonsense mutations measured against synonymous mutations per individual haplogroup, which in this case is **N1**. *P*-values between ratios in the three analysed groups are shown above the bars, where N.S. corresponds to non-significant.

Group per Tree Comparison – N2

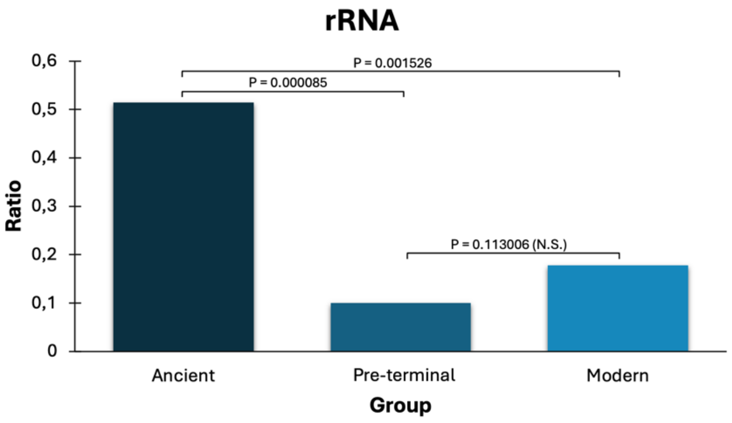

Figure S17. Comparison of the ratio of rRNA mitochondrial mutations measured against synonymous mutations per individual haplogroup, which in this case is **N2**. *P*-values between ratios in the three analysed groups are shown above the bars, where N.S. corresponds to non-significant.

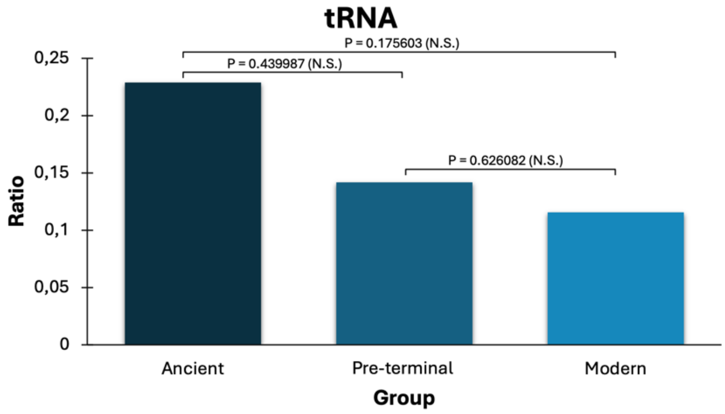

Figure S18. Comparison of the ratio of tRNA mitochondrial mutations measured against synonymous mutations per individual haplogroup, which in this case is **N2**. *P*-values between ratios in the three analysed groups are shown above the bars, where N.S. corresponds to non-significant.

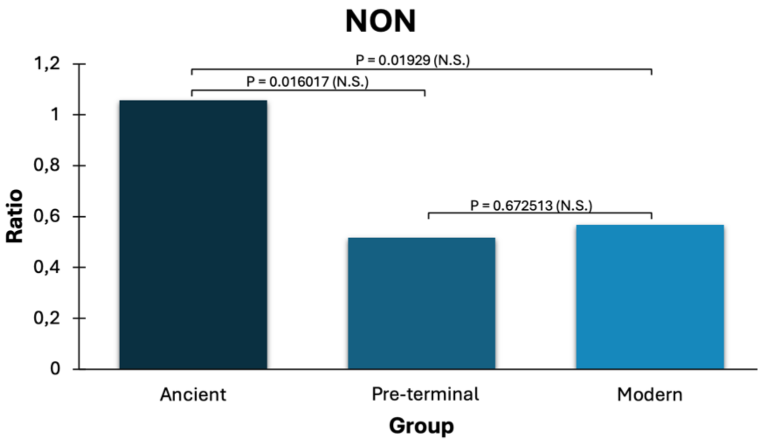

Figure S19. Comparison of the ratio of mitochondrial non-synonymous mutations measured against synonymous mutations per individual haplogroup, which in this case is **N2**. *P*-values between ratios in the three analysed groups are shown above the bars, where N.S. corresponds to non-significant.

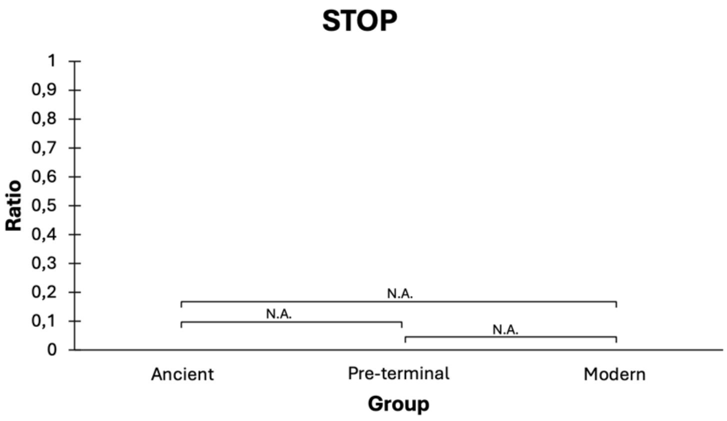

Figure S20. Comparison of the ratio of mitochondrial nonsense mutations measured against synonymous mutations per individual haplogroup, which in this case is **N2**. *P*-values between ratios in the three analysed groups are shown above the bars, where N.S. corresponds to non-significant.

Group per Tree Comparison – X

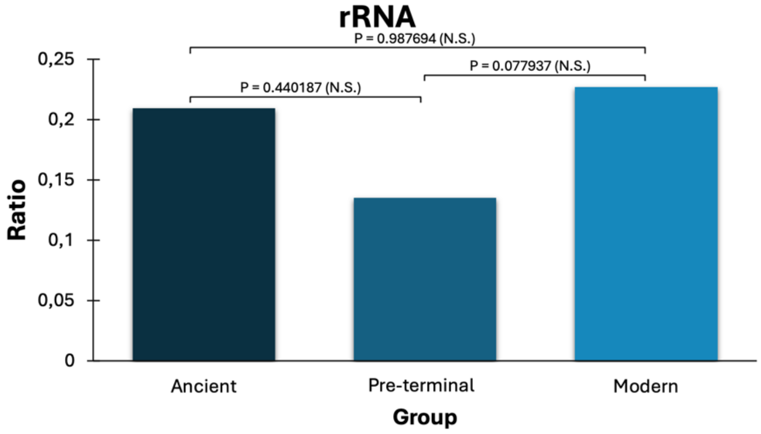

Figure S21. Comparison of the ratio of rRNA mitochondrial mutations measured against synonymous mutations per individual haplogroup, which in this case is **X**. *P*-values between ratios in the three analysed groups are shown above the bars, where N.S. corresponds to non-significant.

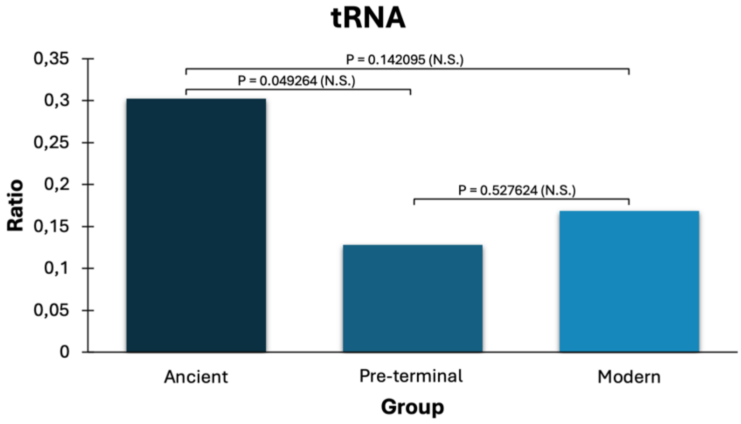

Figure S22. Comparison of the ratio of tRNA mitochondrial mutations measured against synonymous mutations per individual haplogroup, which in this case is **X**. *P*-values between ratios in the three analysed groups are shown above the bars, where N.S. corresponds to non-significant.

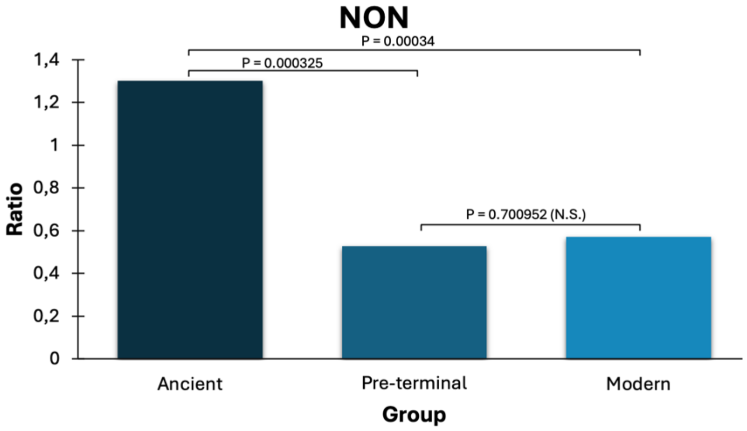

Figure S23. Comparison of the ratio of mitochondrial non-synonymous mutations measured against synonymous mutations per individual haplogroup, which in this case is **X**. *P*-values between ratios in the three analysed groups are shown above the bars, where N.S. corresponds to non-significant.

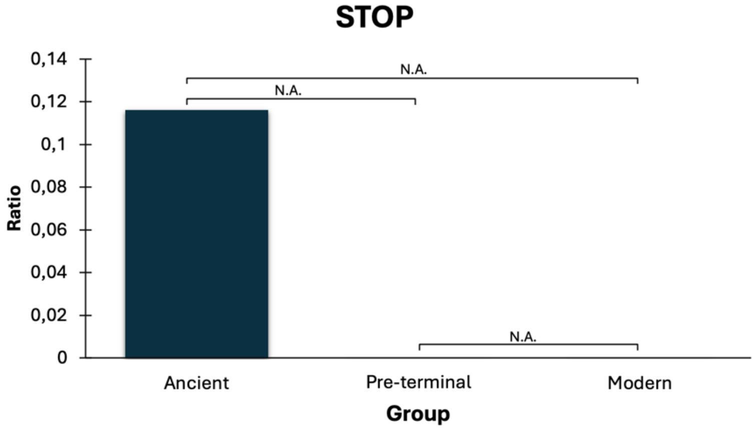

Figure S24. Comparison of the ratio of mitochondrial nonsense mutations measured against synonymous mutations per individual haplogroup, which in this case is **X**. *P*-values between ratios in the three analysed groups are shown above the bars, where N.S. corresponds to non-significant.

Tree Comparison – R0/U

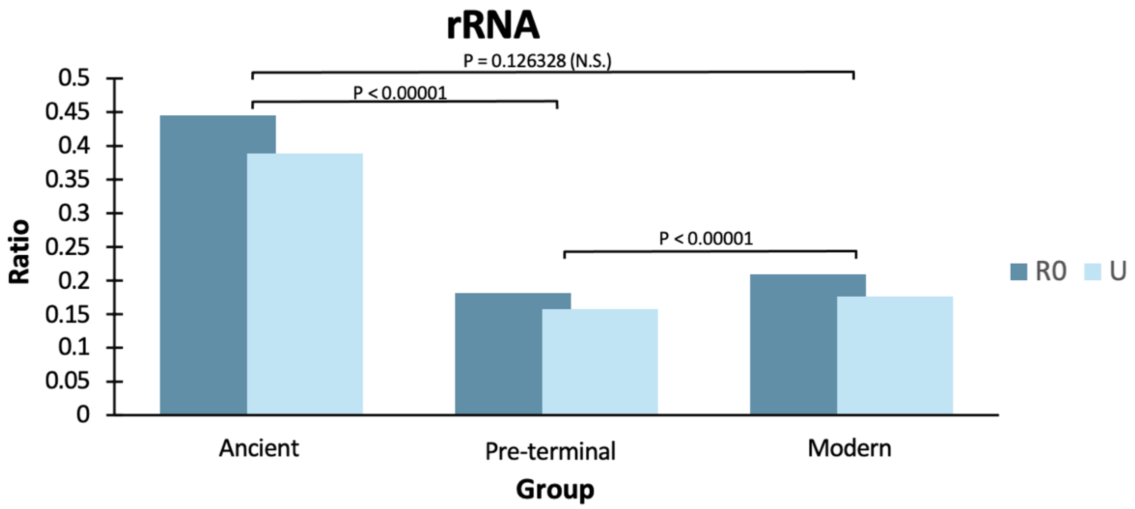

Figure S25. Comparison of the ratio of rRNA mitochondrial mutations measured against synonymous mutations between pairs of haplogroups, which in this case are **R0/U**. *P*-values between ratios in the three analysed groups are shown above the bars, where N.S. corresponds to non-significant.

Tree Comparison – R0/JT

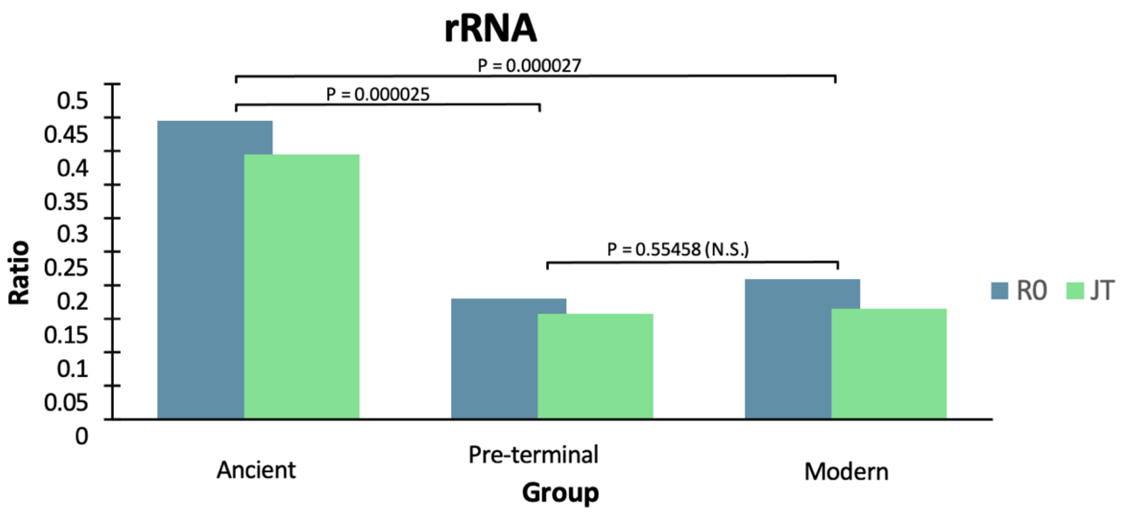

Figure S26. Comparison of the ratio of rRNA mitochondrial mutations measured against synonymous mutations between pairs of haplogroups, which in this case are **R0/JT**. *P*-values between ratios in the three analysed groups are shown above the bars, where N.S. corresponds to non-significant.

Tree Comparison – R0/N1

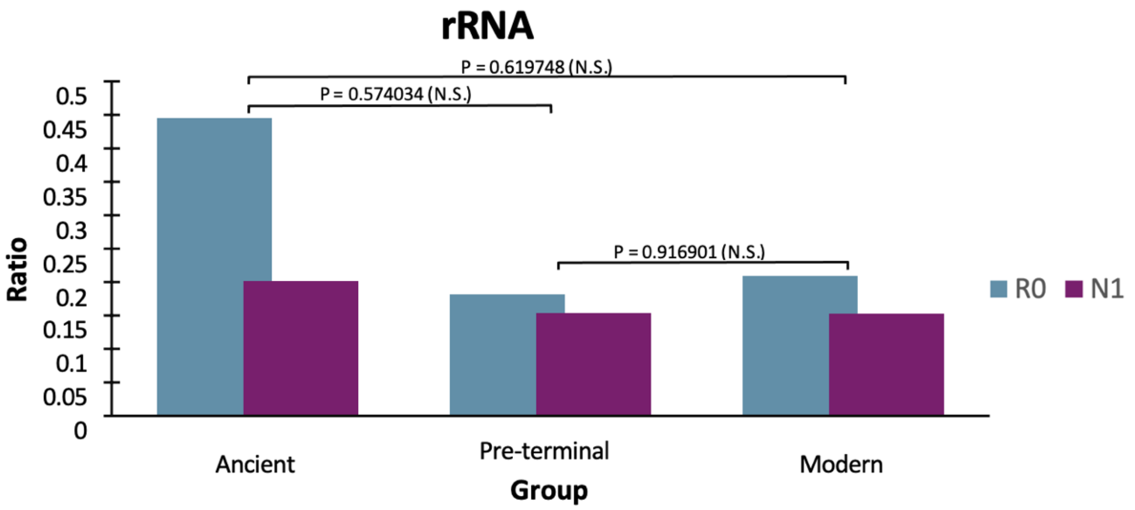

Figure S27. Comparison of the ratio of rRNA mitochondrial mutations measured against synonymous mutations between pairs of haplogroups, which in this case are **R0/N1**. *P*-values between ratios in the three analysed groups are shown above the bars, where N.S. corresponds to non-significant.

Tree Comparison – R0/N2

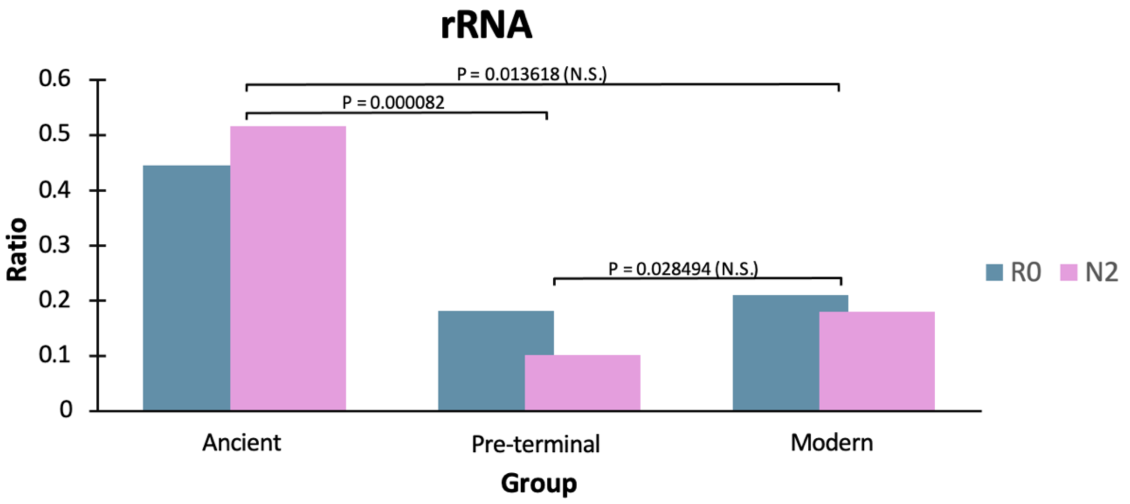

Figure S28. Comparison of the ratio of rRNA mitochondrial mutations measured against synonymous mutations between pairs of haplogroups, which in this case are **R0/N2**. *P*-values between ratios in the three analysed groups are shown above the bars, where N.S. corresponds to non-significant.

Tree Comparison – R0/X

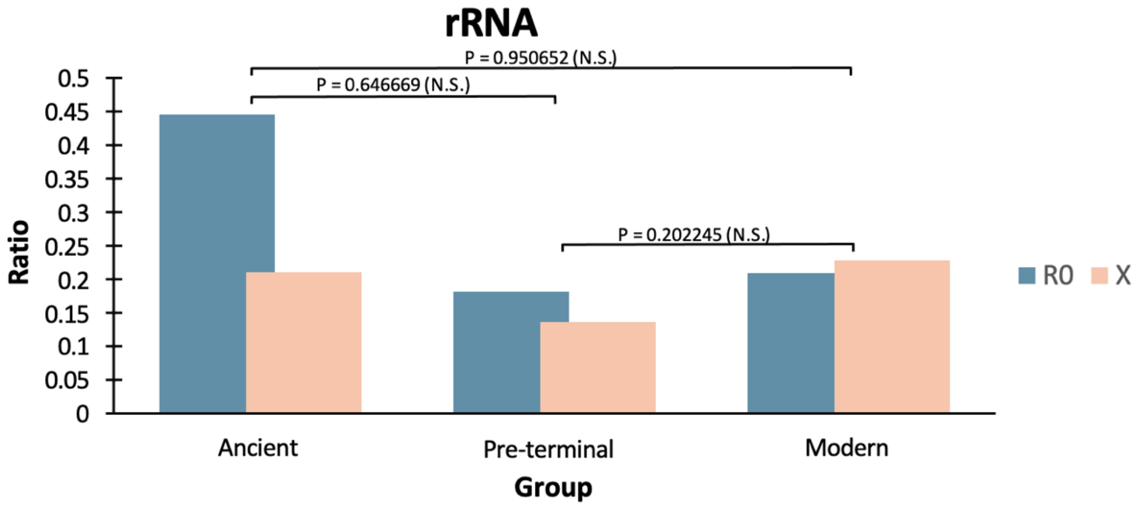

Figure S29. Comparison of the ratio of rRNA mitochondrial mutations measured against synonymous mutations between pairs of haplogroups, which in this case are **R0/X**. *P*-values between ratios in the three analysed groups are shown above the bars, where N.S. corresponds to non-significant.

Tree Comparison – U/JT

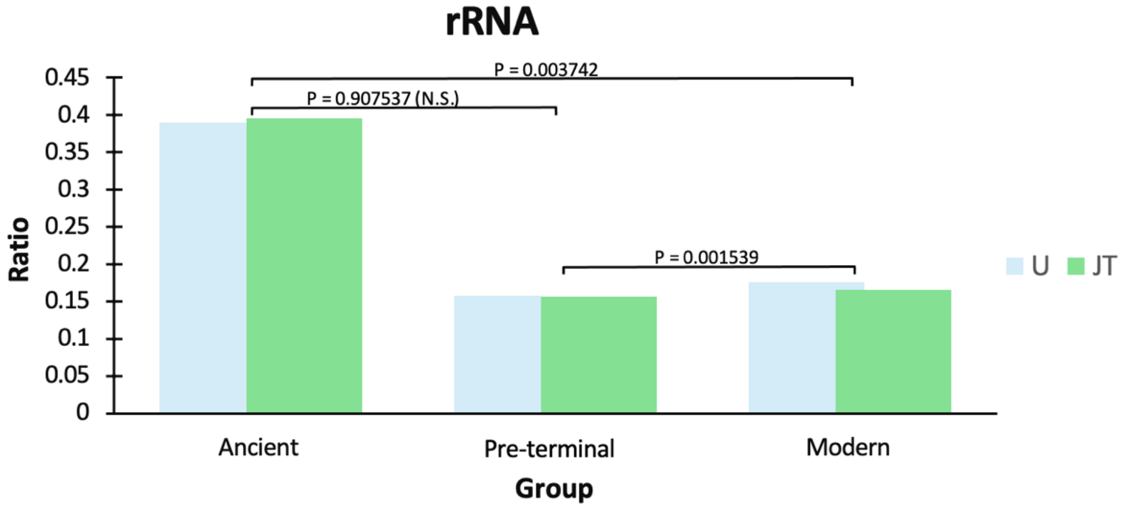

Figure S30. Comparison of the ratio of rRNA mitochondrial mutations measured against synonymous mutations between pairs of haplogroups, which in this case are **U/JT**. *P*-values between ratios in the three analysed groups are shown above the bars, where N.S. corresponds to non-significant.

Tree Comparison – U/N1

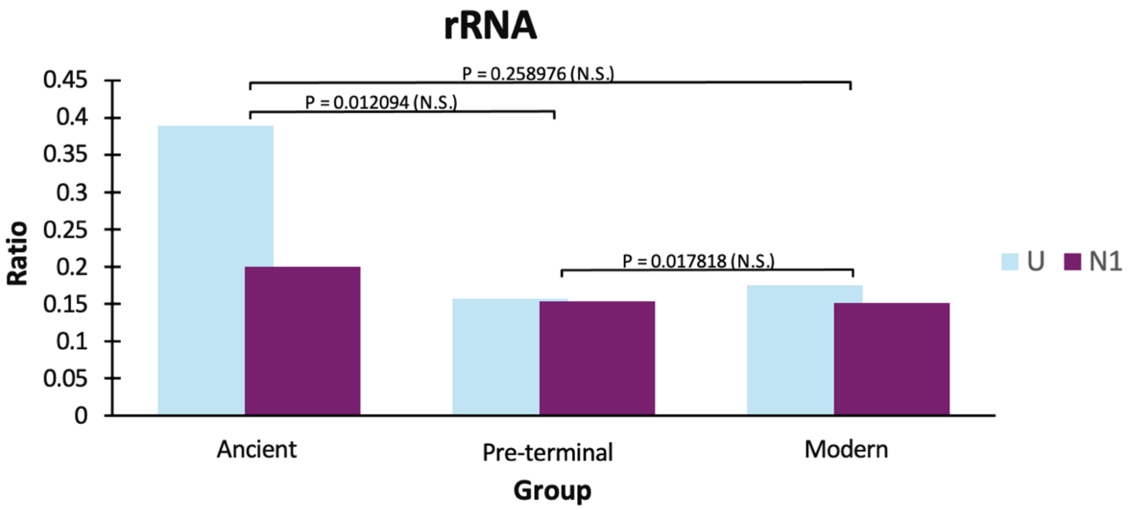

Figure S31. Comparison of the ratio of rRNA mitochondrial mutations measured against synonymous mutations between pairs of haplogroups, which in this case are **U/N1**. *P*-values between ratios in the three analysed groups are shown above the bars, where N.S. corresponds to non-significant.

Tree Comparison – U/N2

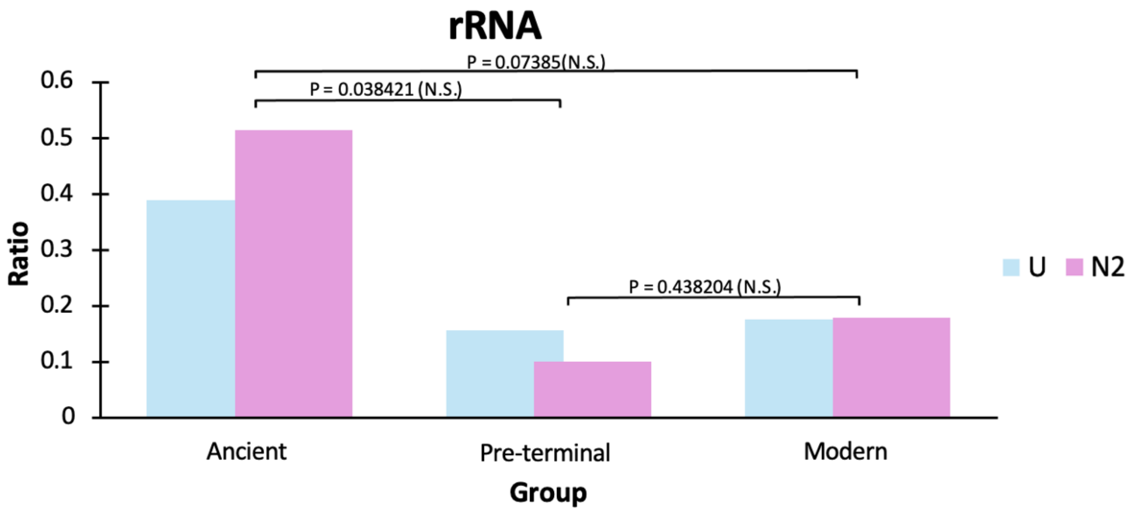

Figure S32. Comparison of the ratio of rRNA mitochondrial mutations measured against synonymous mutations between pairs of haplogroups, which in this case are **U/N2**. *P*-values between ratios in the three analysed groups are shown above the bars, where N.S. corresponds to non-significant.

Tree Comparison – U/X

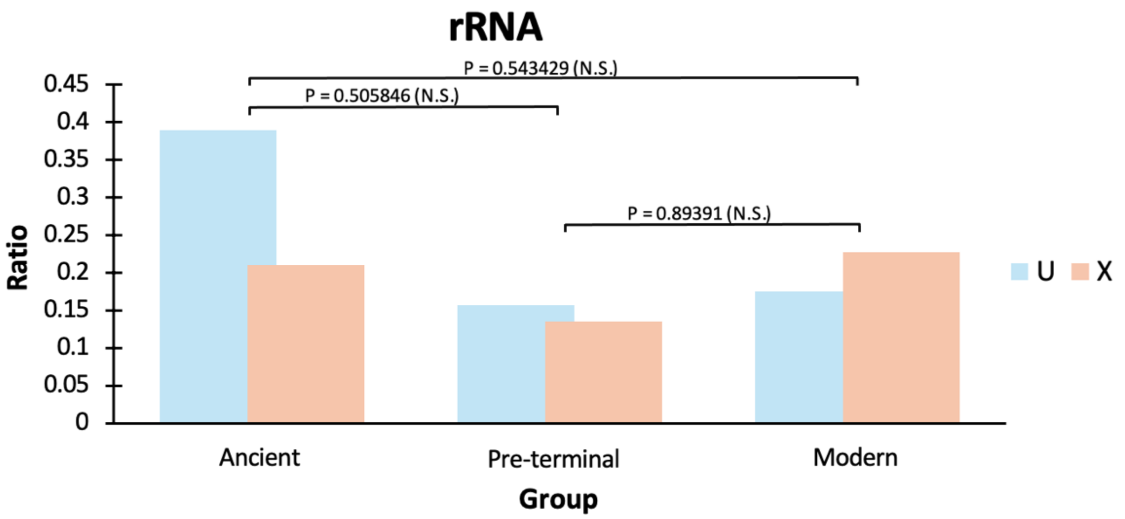

Figure S33. Comparison of the ratio of rRNA mitochondrial mutations measured against synonymous mutations between pairs of haplogroups, which in this case are **U/X**. *P*-values between ratios in the three analysed groups are shown above the bars, where N.S. corresponds to non-significant.

Tree Comparison – JT/N1

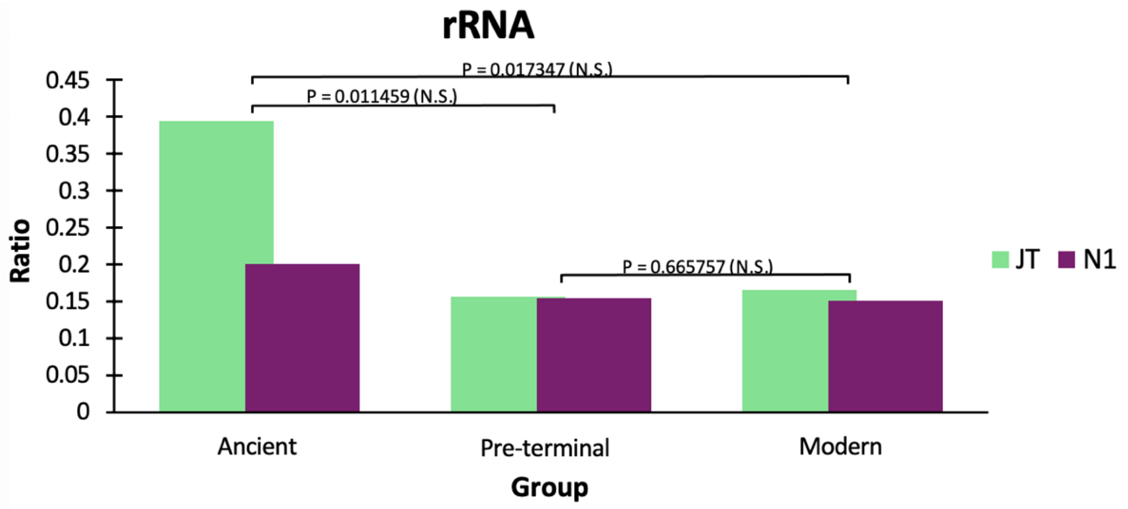

Figure S34. Comparison of the ratio of rRNA mitochondrial mutations measured against synonymous mutations between pairs of haplogroups, which in this case are **JT/N1**. *P*-values between ratios in the three analysed groups are shown above the bars, where N.S. corresponds to non-significant.

Tree Comparison – JT/N2

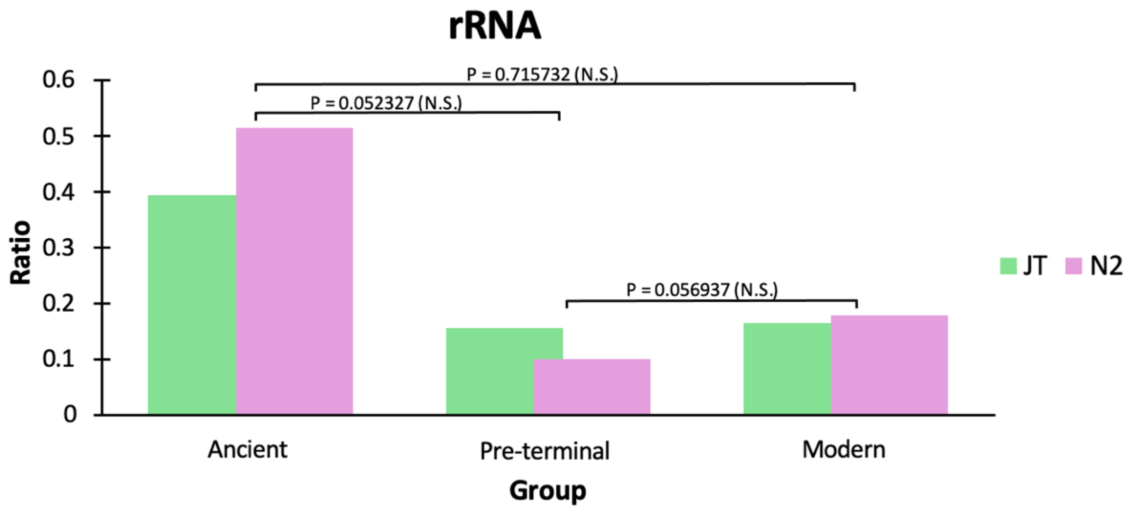

Figure S35. Comparison of the ratio of rRNA mitochondrial mutations measured against synonymous mutations between pairs of haplogroups, which in this case are **JT/N2**. *P*-values between ratios in the three analysed groups are shown above the bars, where N.S. corresponds to non-significant.

Tree Comparison – JT/X

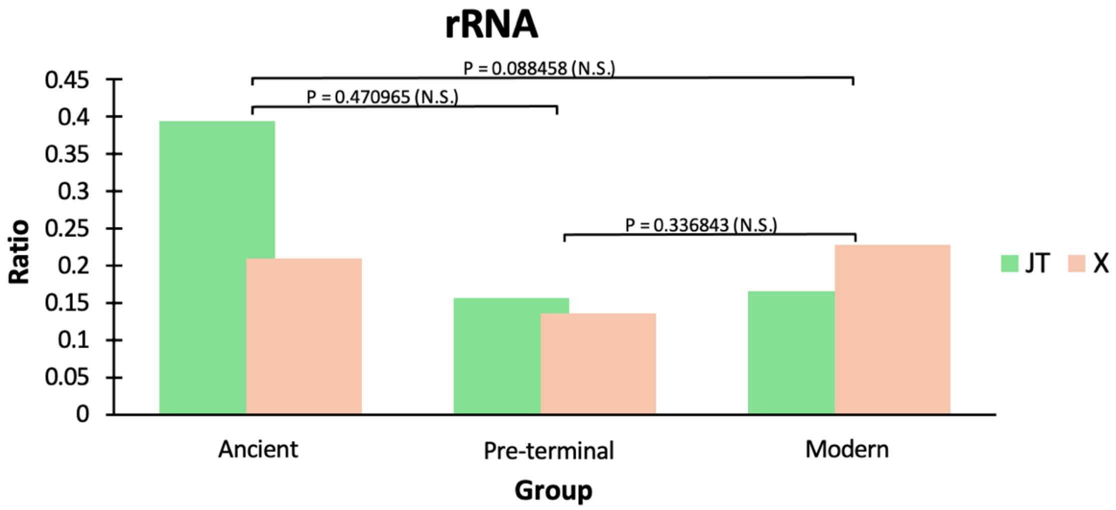

Figure S36. Comparison of the ratio of rRNA mitochondrial mutations measured against synonymous mutations between pairs of haplogroups, which in this case are **JT/X**. *P*-values between ratios in the three analysed groups are shown above the bars, where N.S. corresponds to non-significant.

Tree Comparison – N1/N2

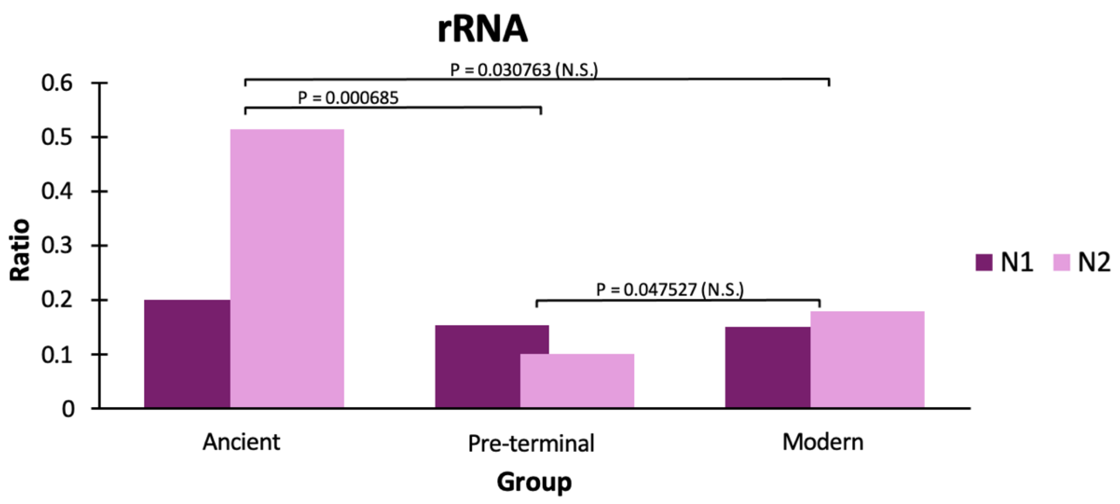

Figure S37. Comparison of the ratio of rRNA mitochondrial mutations measured against synonymous mutations between pairs of haplogroups, which in this case are **N1/N2**. *P*-values between ratios in the three analysed groups are shown above the bars, where N.S. corresponds to non-significant.

Tree Comparison – N1/X

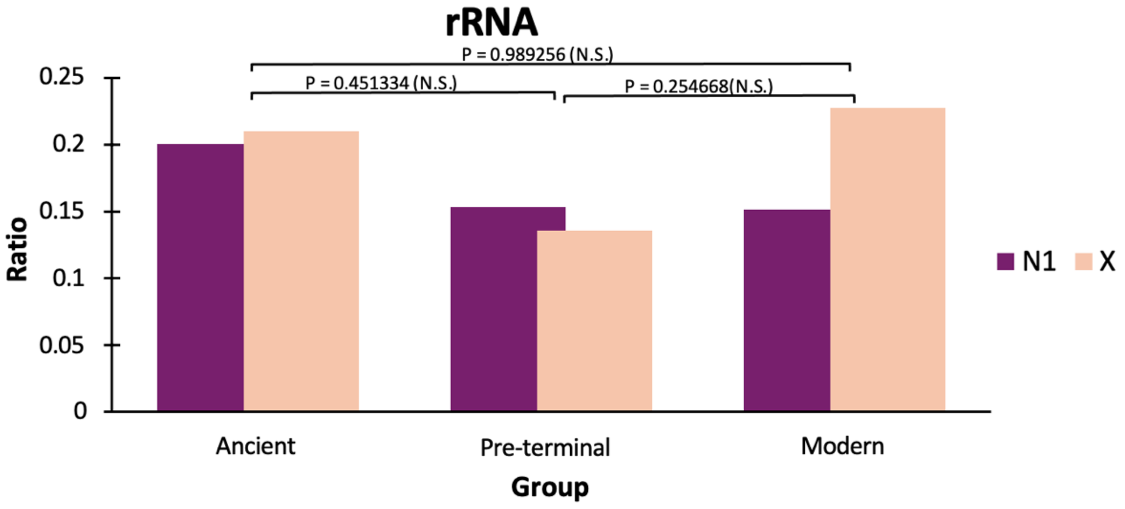

Figure S38. Comparison of the ratio of rRNA mitochondrial mutations measured against synonymous mutations between pairs of haplogroups, which in this case are **N1/X**. *P*-values between ratios in the three analysed groups are shown above the bars, where N.S. corresponds to non-significant.

Tree Comparison – N2/X

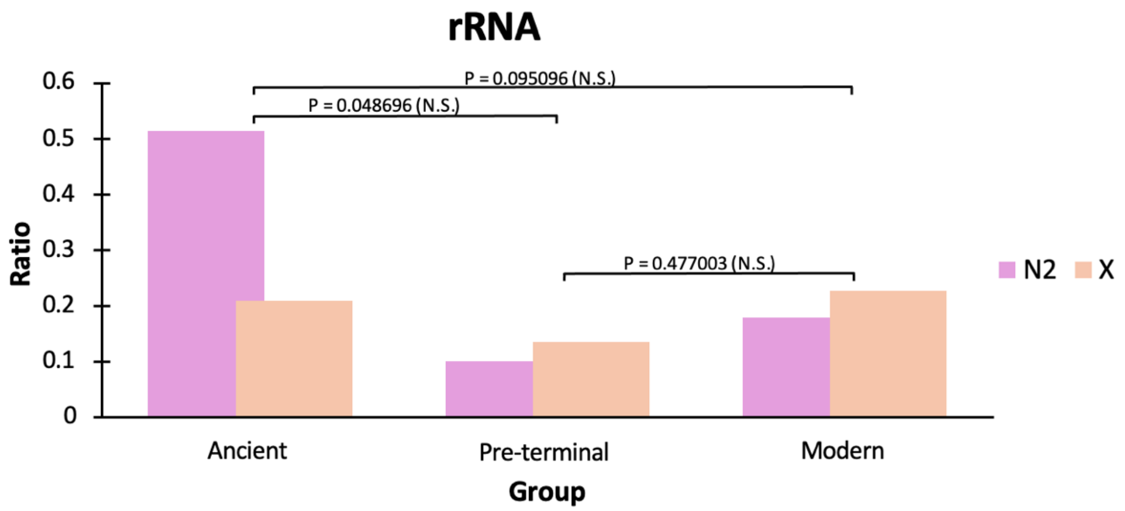

Figure S39. Comparison of the ratio of rRNA mitochondrial mutations measured against synonymous mutations between pairs of haplogroups, which in this case are **N2/X**. *P*-values between ratios in the three analysed groups are shown above the bars, where N.S. corresponds to non-significant.

Tree Comparison – R0/U

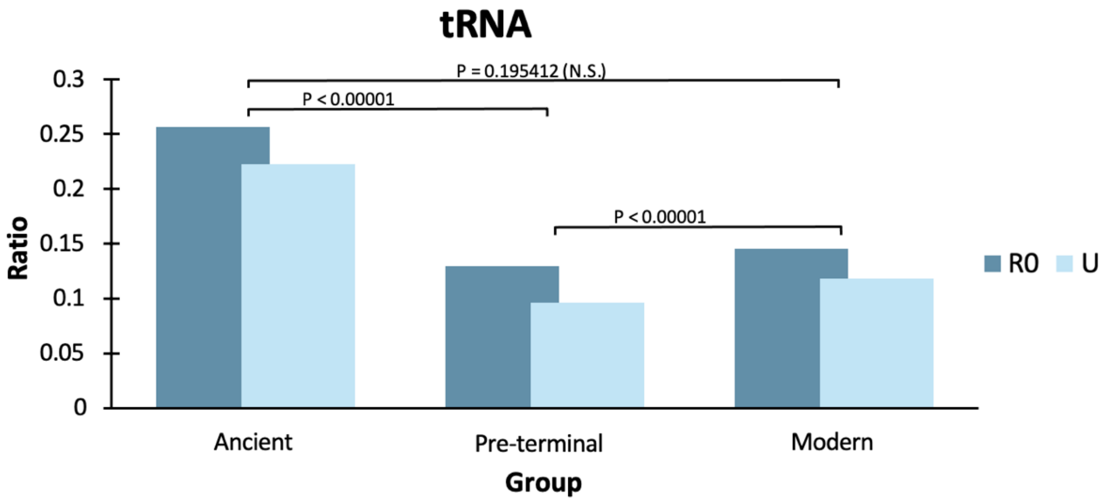

Figure S40. Comparison of the ratio of tRNA mitochondrial mutations measured against synonymous mutations between pairs of haplogroups, which in this case are **R0/U**. *P*-values between ratios in the three analysed groups are shown above the bars, where N.S. corresponds to non-significant.

Tree Comparison – R0/JT

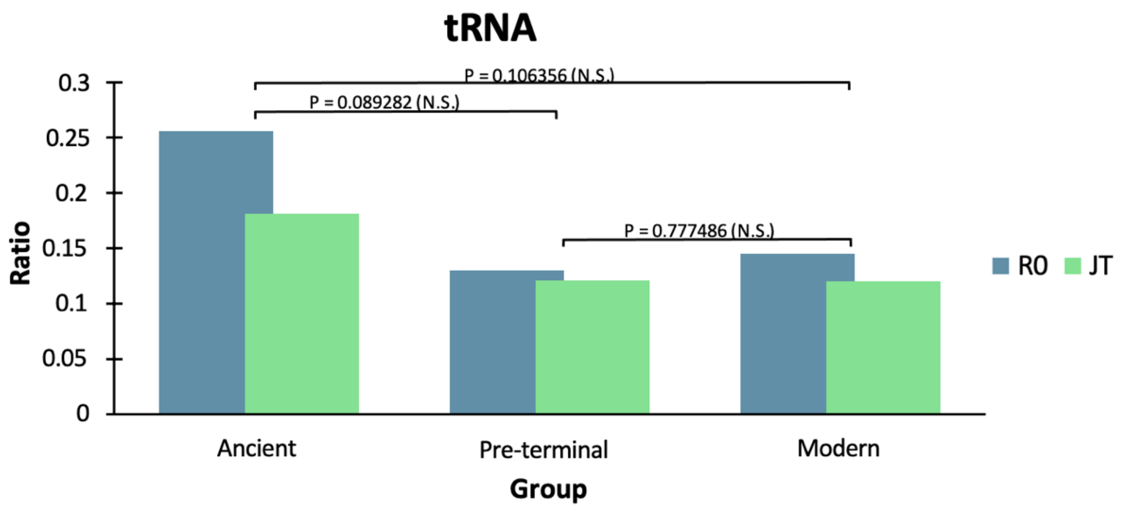

Figure S41. Comparison of the ratio of tRNA mitochondrial mutations measured against synonymous mutations between pairs of haplogroups, which in this case are **R0/JT**. *P*-values between ratios in the three analysed groups are shown above the bars, where N.S. corresponds to non-significant.

Tree Comparison – R0/N1

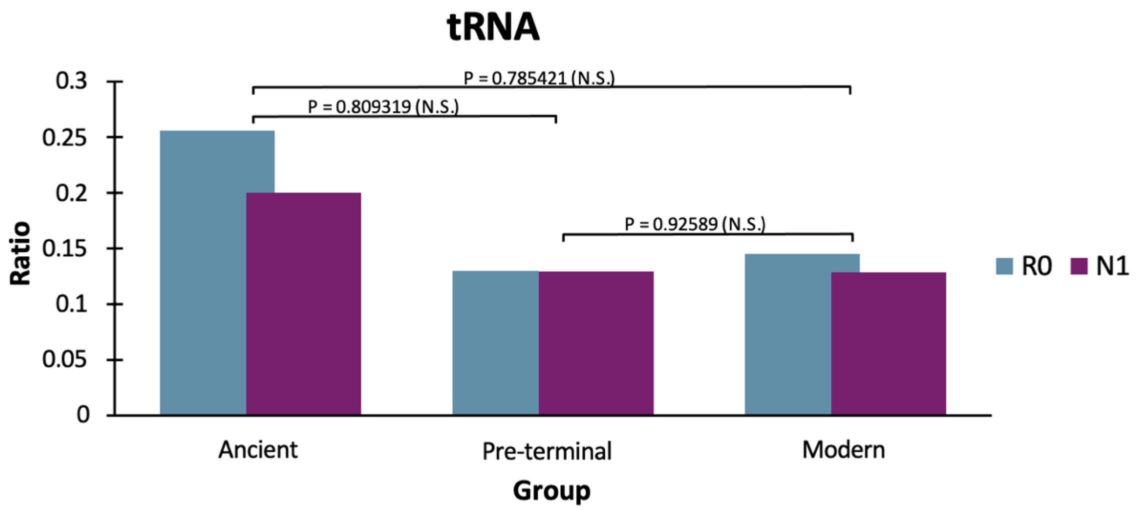

Figure S42. Comparison of the ratio of tRNA mitochondrial mutations measured against synonymous mutations between pairs of haplogroups, which in this case are **R0/N1**. *P*-values between ratios in the three analysed groups are shown above the bars, where N.S. corresponds to non-significant.

Tree Comparison – R0/N2

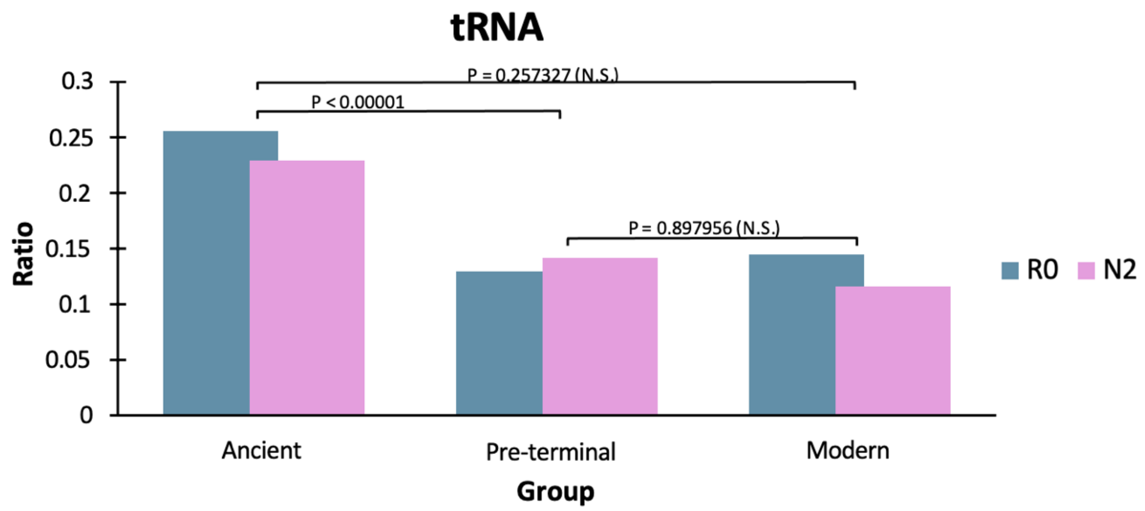

Figure S43. Comparison of the ratio of tRNA mitochondrial mutations measured against synonymous mutations between pairs of haplogroups, which in this case are **R0/N2**. *P*-values between ratios in the three analysed groups are shown above the bars, where N.S. corresponds to non-significant.

Tree Comparison – R0/X

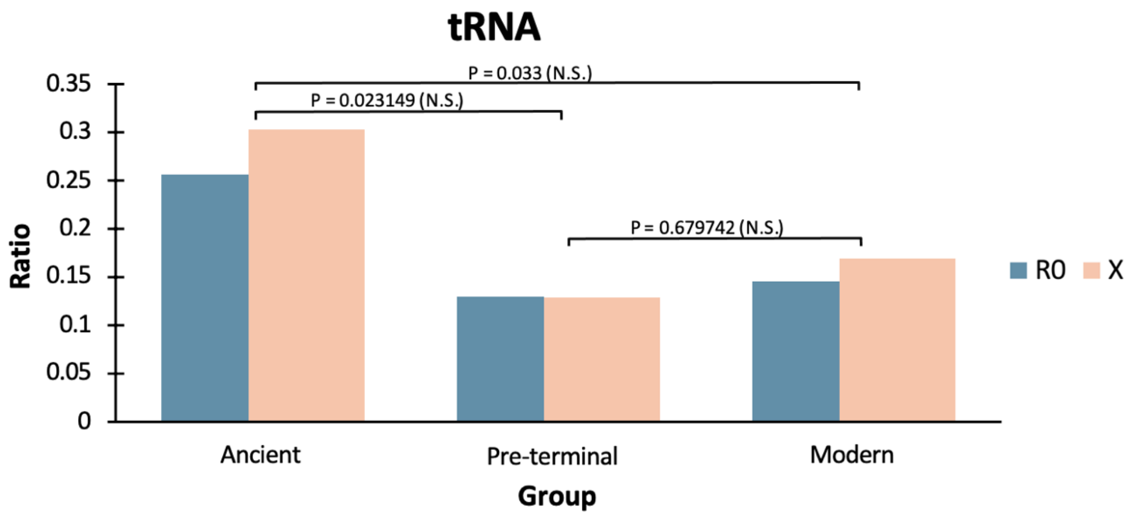

Figure S44. Comparison of the ratio of tRNA mitochondrial mutations measured against synonymous mutations between pairs of haplogroups, which in this case are **R0/X**. *P*-values between ratios in the three analysed groups are shown above the bars, where N.S. corresponds to non-significant.

Tree Comparison – U/JT

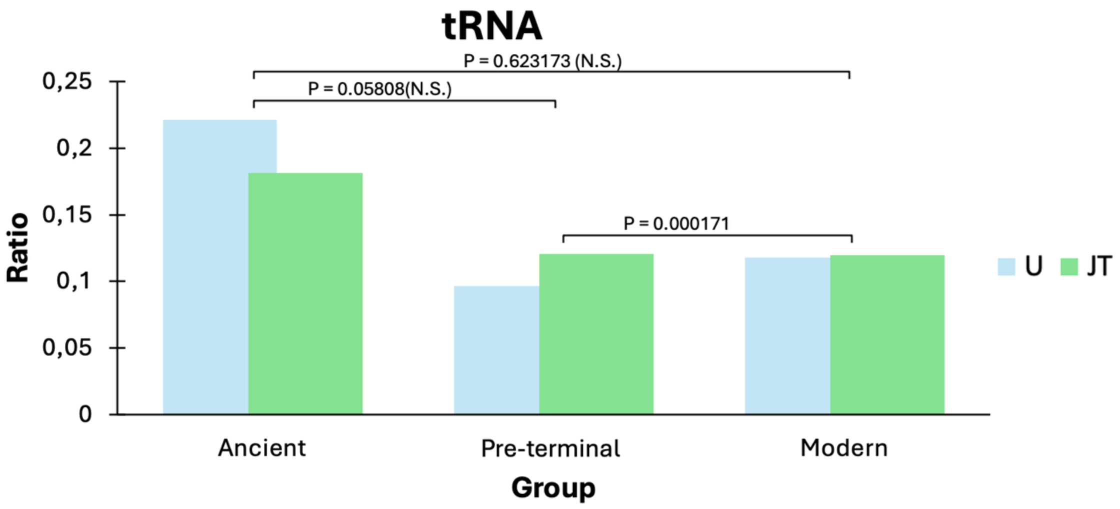

Figure S45. Comparison of the ratio of tRNA mitochondrial mutations measured against synonymous mutations between pairs of haplogroups, which in this case are **U/JT**. *P*-values between ratios in the three analysed groups are shown above the bars, where N.S. corresponds to non-significant.

Tree Comparison – U/N1

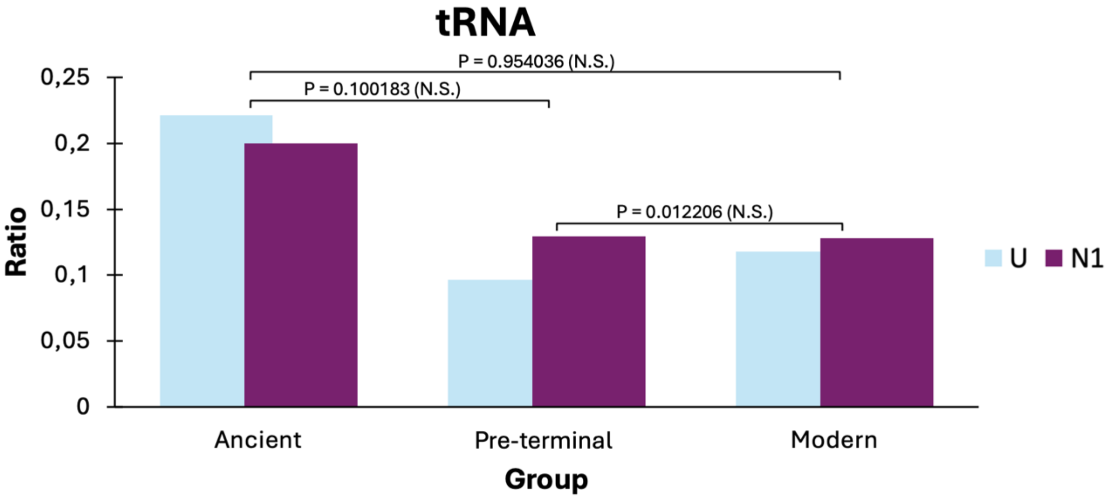

Figure S46. Comparison of the ratio of tRNA mitochondrial mutations measured against synonymous mutations between pairs of haplogroups, which in this case are **U/N1**. *P*-values between ratios in the three analysed groups are shown above the bars, where N.S. corresponds to non-significant.

Tree Comparison – U/N2

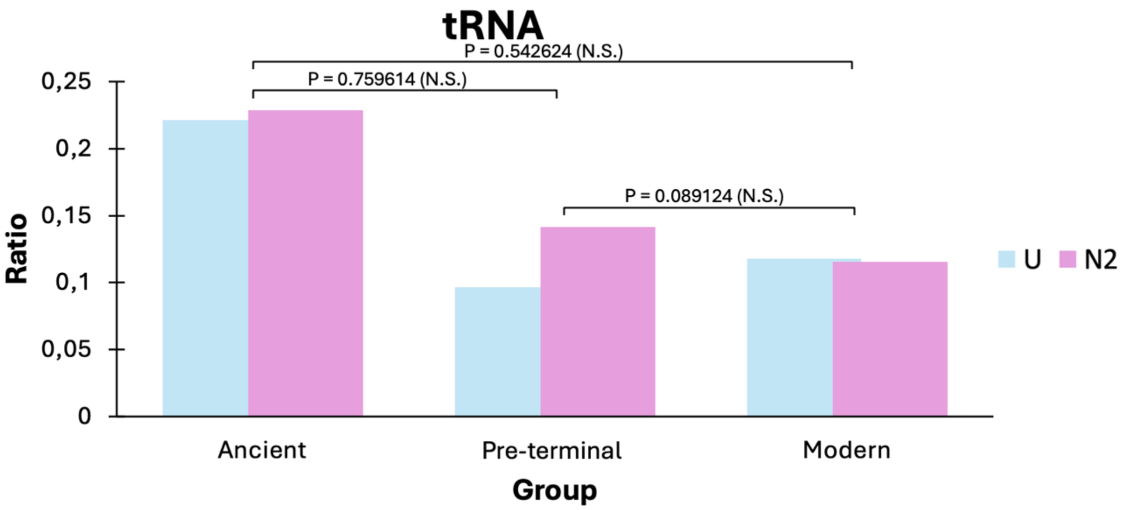

Figure S47. Comparison of the ratio of tRNA mitochondrial mutations measured against synonymous mutations between pairs of haplogroups, which in this case are **U/N2**. *P*-values between ratios in the three analysed groups are shown above the bars, where N.S. corresponds to non-significant.

Tree Comparison – U/X

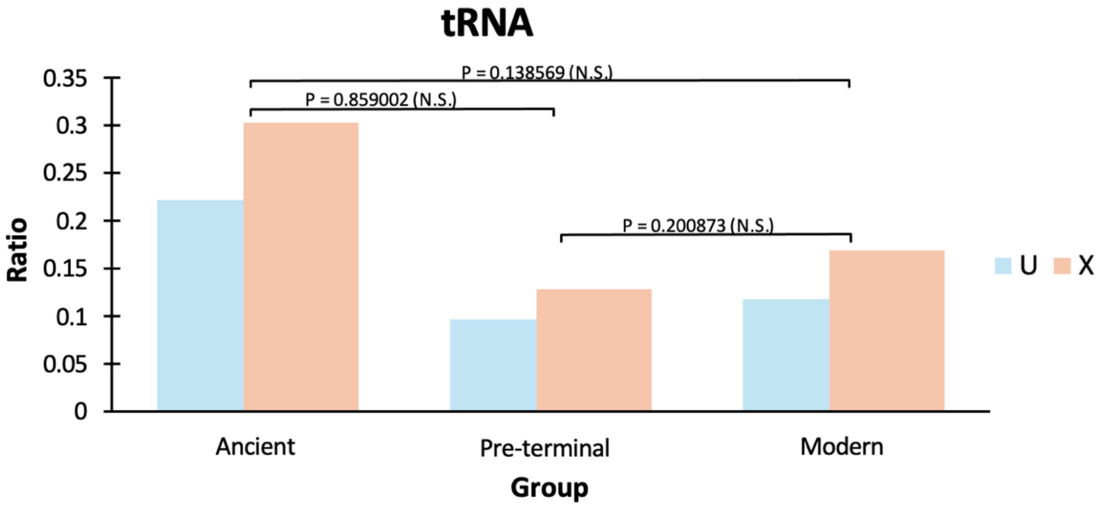

Figure S48. Comparison of the ratio of tRNA mitochondrial mutations measured against synonymous mutations between pairs of haplogroups, which in this case are **U/X**. *P*-values between ratios in the three analysed groups are shown above the bars, where N.S. corresponds to non-significant.

Tree Comparison – JT/N1

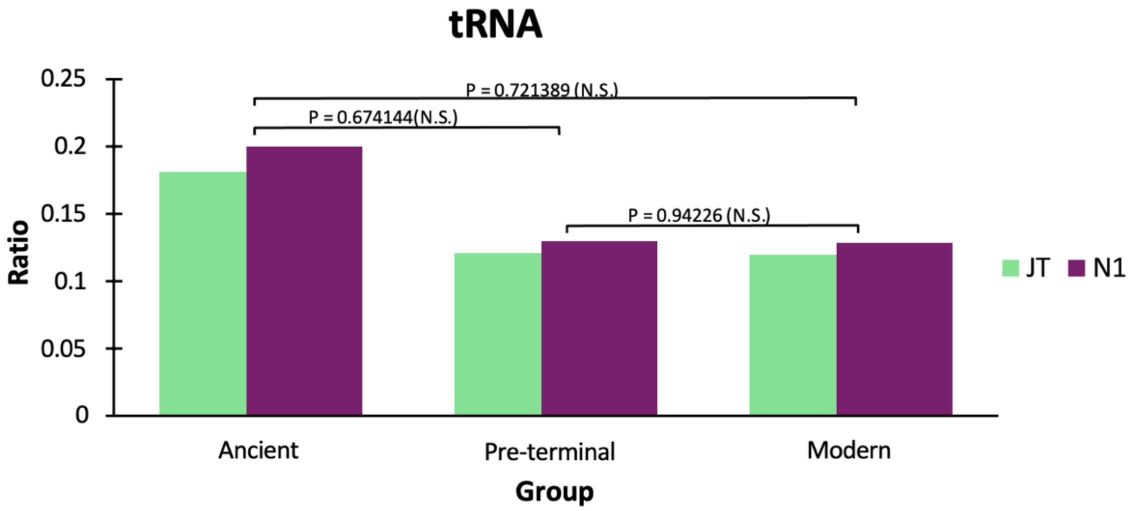

Figure S49. Comparison of the ratio of tRNA mitochondrial mutations measured against synonymous mutations between pairs of haplogroups, which in this case are **JT/N1**. *P*-values between ratios in the three analysed groups are shown above the bars, where N.S. corresponds to non-significant.

Tree Comparison – JT/N2

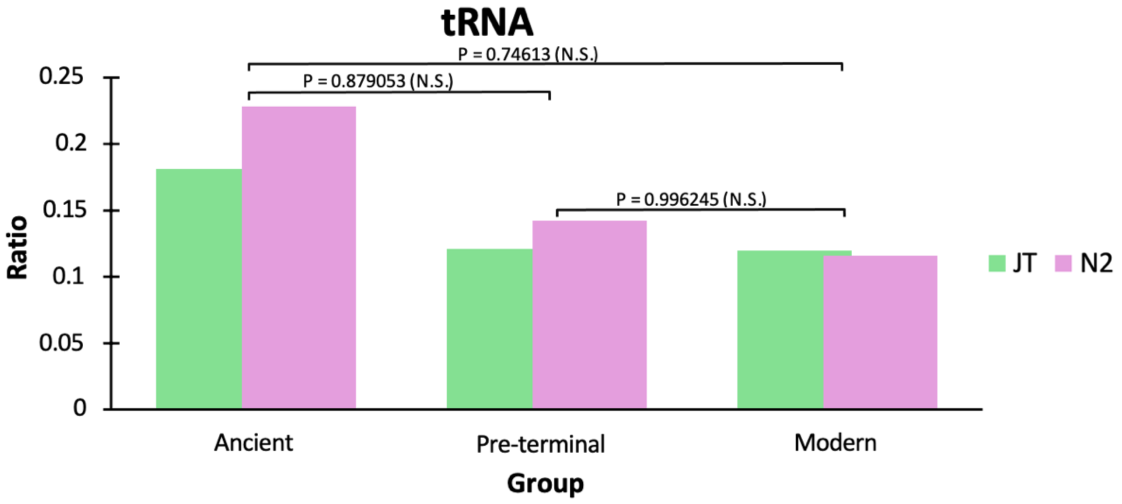

Figure S50. Comparison of the ratio of tRNA mitochondrial mutations measured against synonymous mutations between pairs of haplogroups, which in this case are **JT/N2**. *P*-values between ratios in the three analysed groups are shown above the bars, where N.S. corresponds to non-significant.

Tree Comparison – JT/X

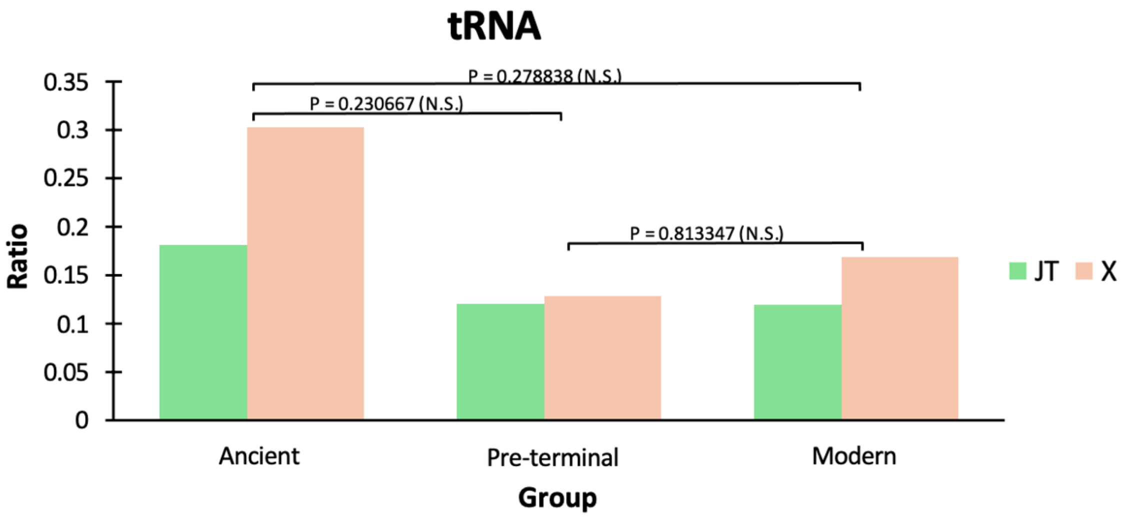

Figure S51. Comparison of the ratio of tRNA mitochondrial mutations measured against synonymous mutations between pairs of haplogroups, which in this case are **JT/X**. *P*-values between ratios in the three analysed groups are shown above the bars, where N.S. corresponds to non-significant.

Tree Comparison – N1/N2

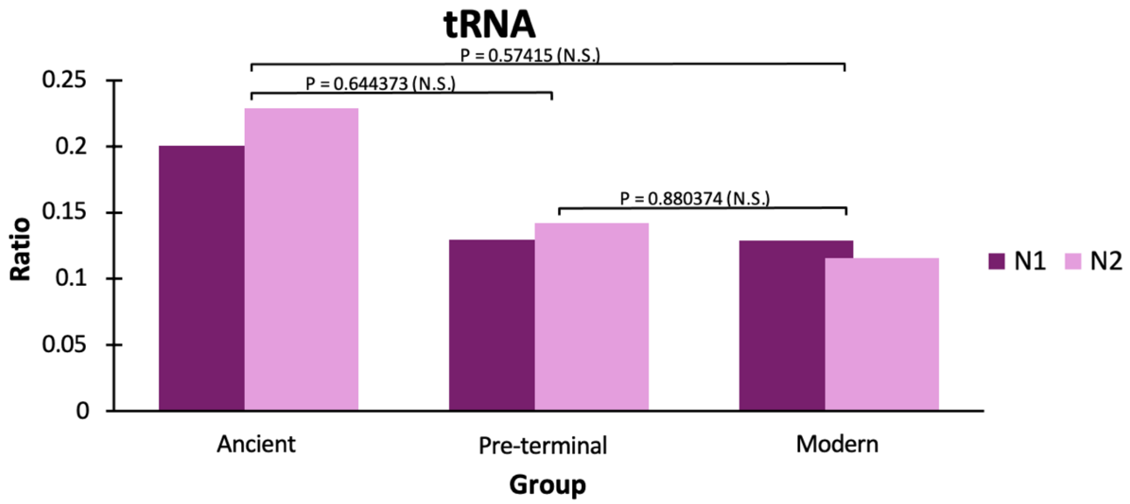

Figure S52. Comparison of the ratio of tRNA mitochondrial mutations measured against synonymous mutations between pairs of haplogroups, which in this case are **N1/N2**. *P*-values between ratios in the three analysed groups are shown above the bars, where N.S corresponds to non-significant.

Tree Comparison – N1/X

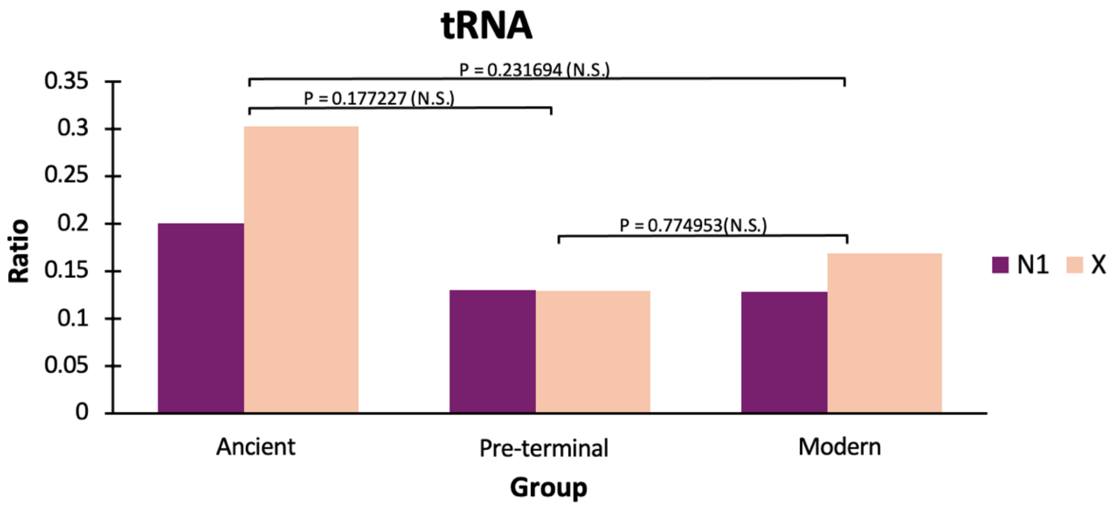

Figure S53. Comparison of the ratio of tRNA mitochondrial mutations measured against synonymous mutations between pairs of haplogroups, which in this case are **N1/X**. *P*-values between ratios in the three analysed groups are shown above the bars, where N.S. corresponds to non-significant.

Tree Comparison – N2/X

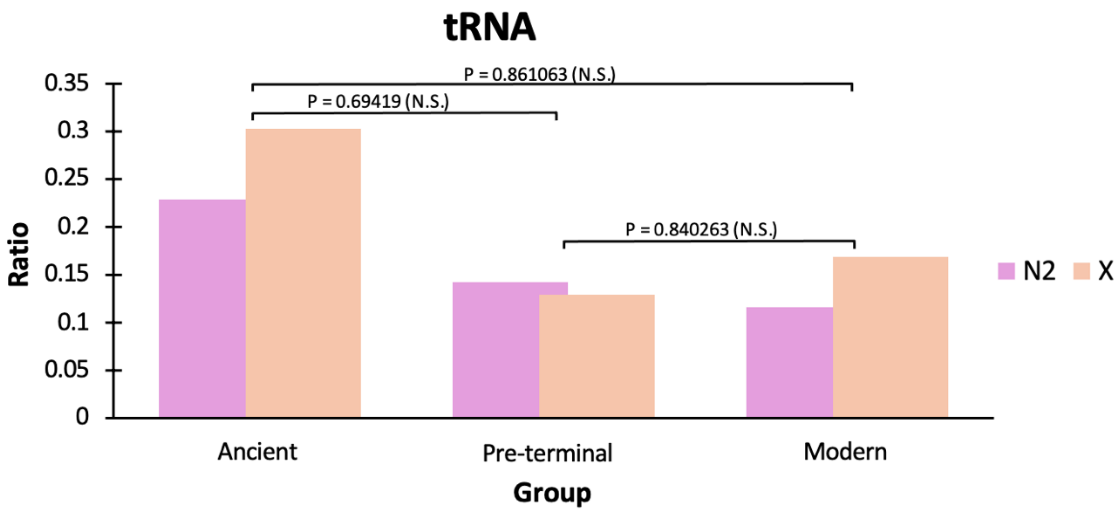

Figure S54. Comparison of the ratio of tRNA mitochondrial mutations measured against synonymous mutations between pairs of haplogroups, which in this case are **N2/X**. *P*-values between ratios in the three analysed groups are shown above the bars, where N.S. corresponds to non-significant.

Tree Comparison – R0/U

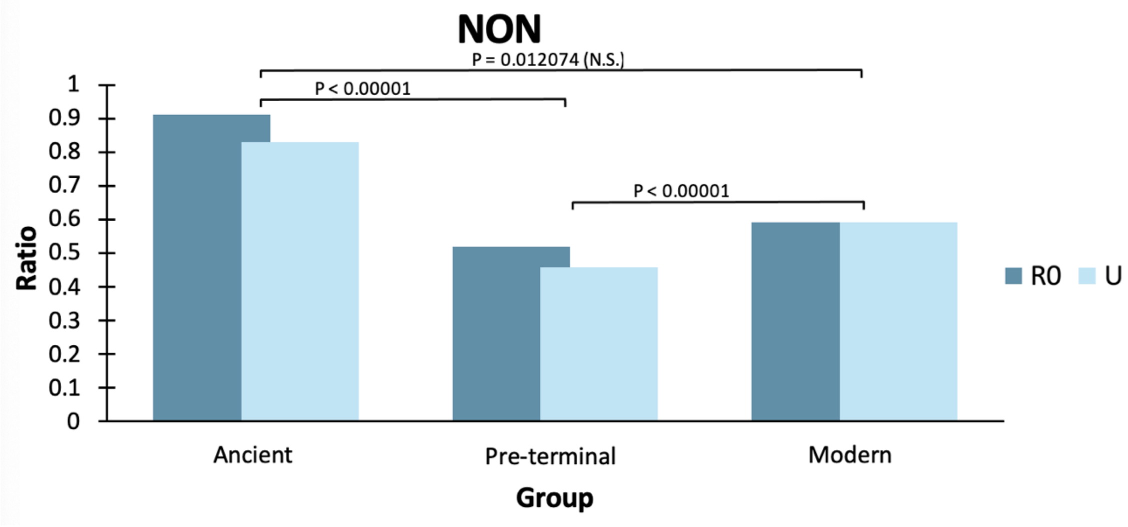

Figure S55. Comparison of the ratio of mitochondrial non-synonymous mutations measured against synonymous mutations between pairs of haplogroups, which in this case are **R0/U**. *P*-values between ratios in the three analysed groups are shown above the bars, where N.S. corresponds to non-significant.

Tree Comparison – R0/JT

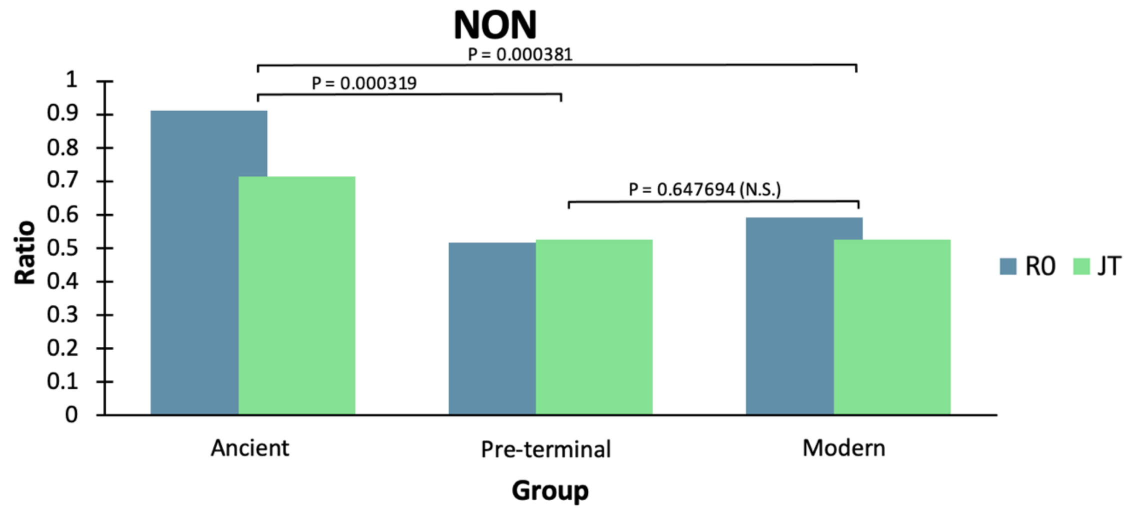

Figure S56. Comparison of the ratio of mitochondrial non-synonymous mutations measured against synonymous mutations between pairs of haplogroups, which in this case are **R0/JT**. *P*-values between ratios in the three analysed groups are shown above the bars, where N.S. corresponds to non-significant.

Tree Comparison – R0/N1

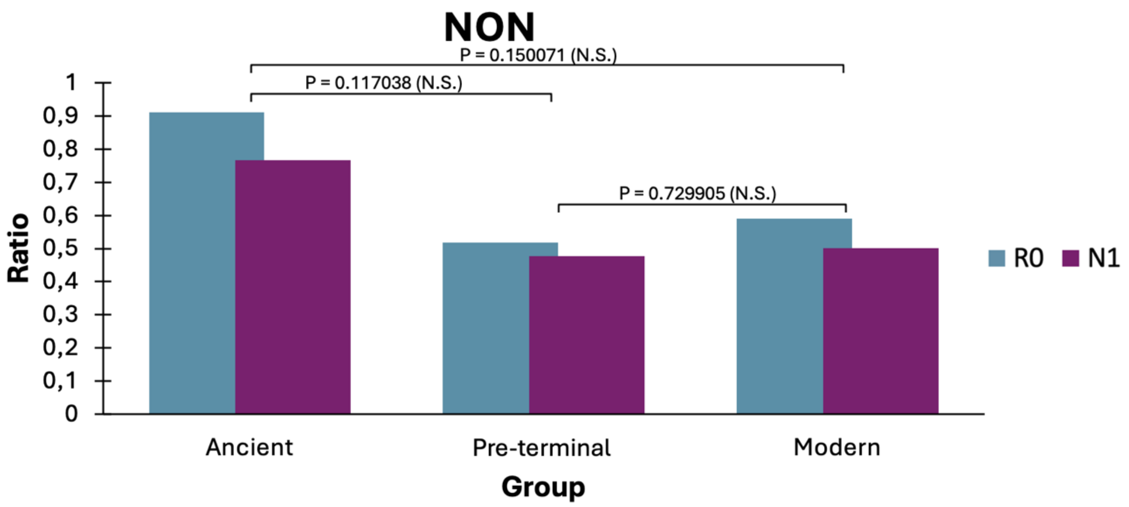

Figure S57. Comparison of the ratio of mitochondrial non-synonymous mutations measured against synonymous mutations between pairs of haplogroups, which in this case are **R0/N1**. *P*-values between ratios in the three analysed groups are shown above the bars, where N.S. corresponds to non-significant.

Tree Comparison – R0/N2

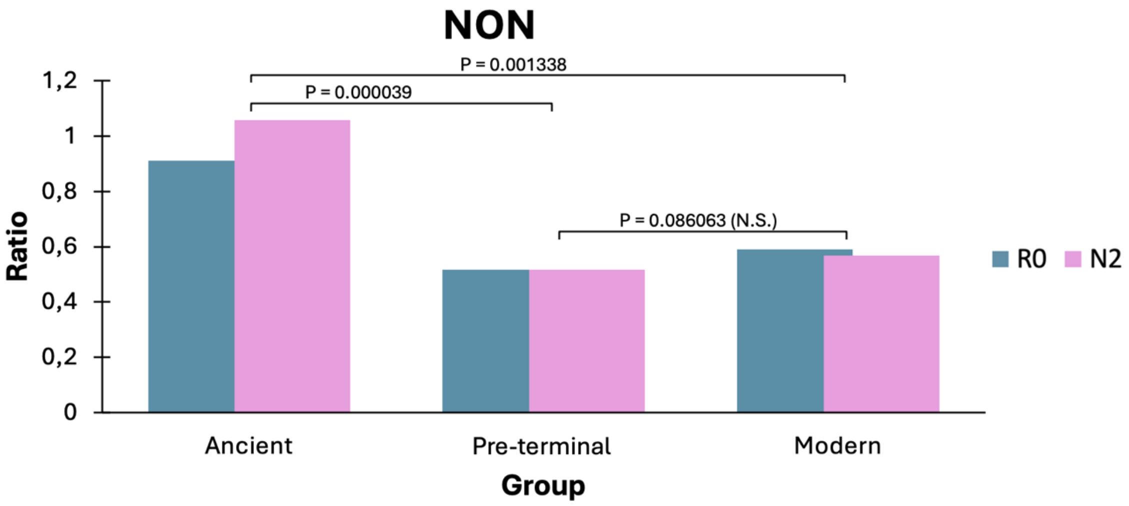

Figure S58. Comparison of the ratio of mitochondrial non-synonymous mutations measured against synonymous mutations between pairs of haplogroups, which in this case are **R0/N2**. *P*-values between ratios in the three analysed groups are shown above the bars, where N.S. corresponds to non-significant.

Tree Comparison – R0/X

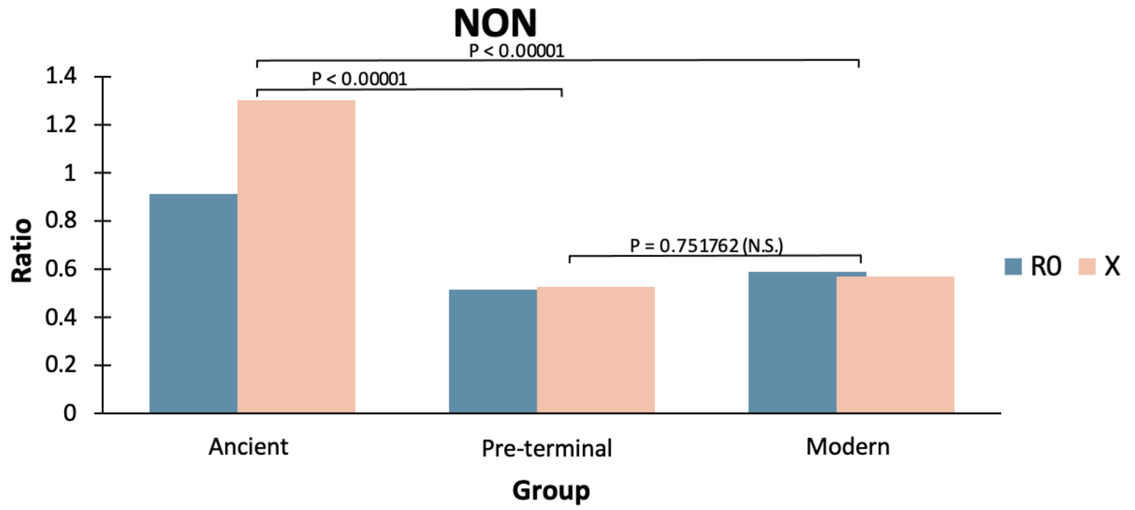

Figure S59. Comparison of the ratio of mitochondrial non-synonymous mutations measured against synonymous mutations between pairs of haplogroups, which in this case are **R0/X**. *P*-values between ratios in the three analysed groups are shown above the bars, where N.S. corresponds to non-significant.

Tree Comparison – U/JT

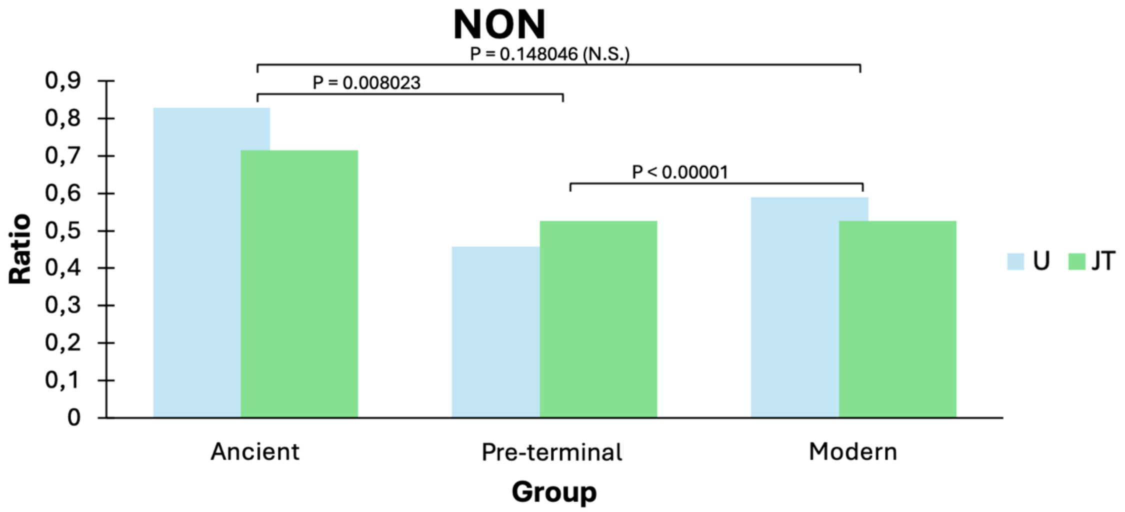

Figure S60. Comparison of the ratio of mitochondrial non-synonymous mutations measured against synonymous mutations between pairs of haplogroups, which in this case are **U/JT**. *P*-values between ratios in the three analysed groups are shown above the bars, where N.S. corresponds to non-significant.

Tree Comparison – U/N1

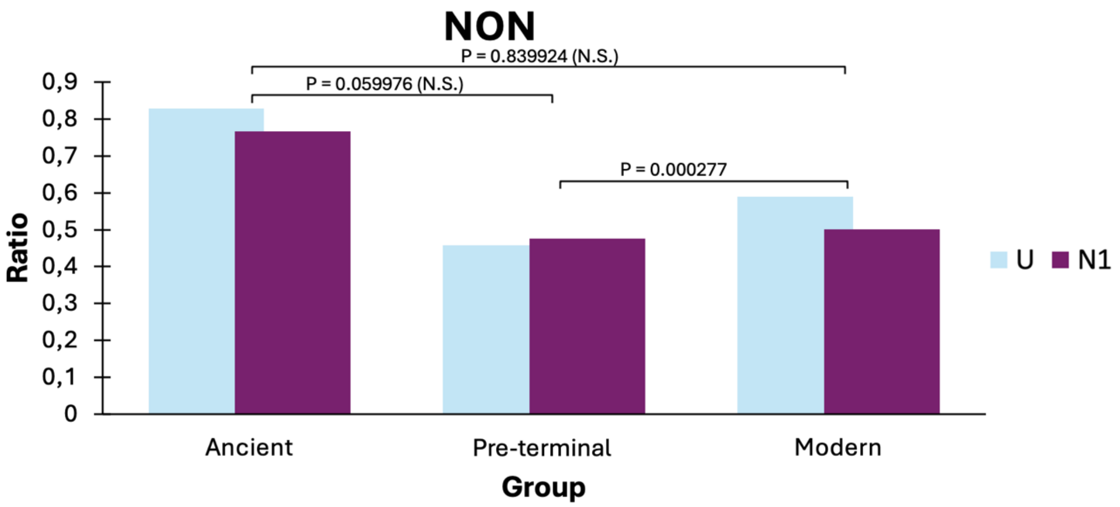

Figure S61. Comparison of the ratio of mitochondrial non-synonymous mutations measured against synonymous mutations between pairs of haplogroups, which in this case are **U/N1**. *P*-values between ratios in the three analysed groups are shown above the bars, where N.S. corresponds to non-significant.

Tree Comparison – U/N2

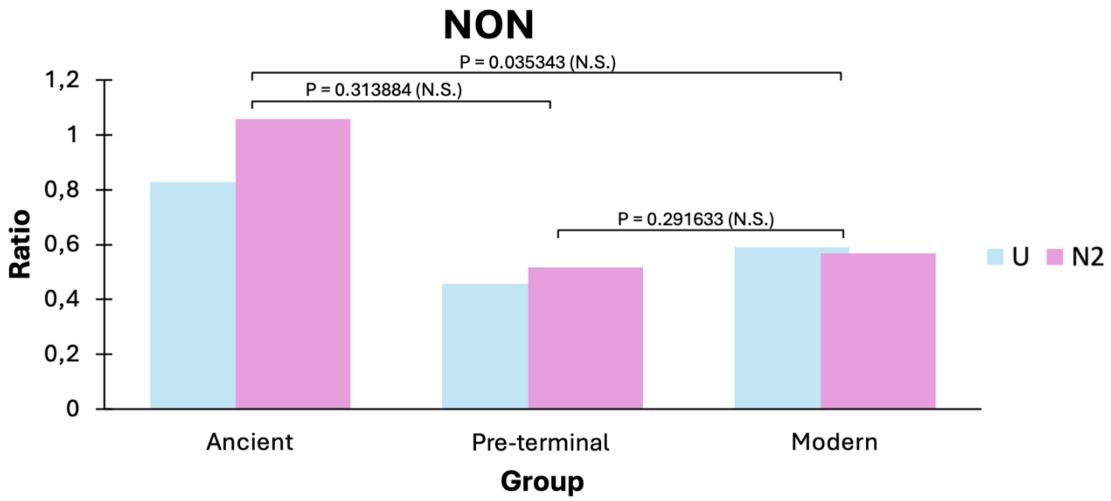

Figure S62. Comparison of the ratio of mitochondrial non-synonymous mutations measured against synonymous mutations between pairs of haplogroups, which in this case are **U/N2**. *P*-values between ratios in the three analysed groups are shown above the bars, where N.S. corresponds to non-significant.

Tree Comparison – U/X

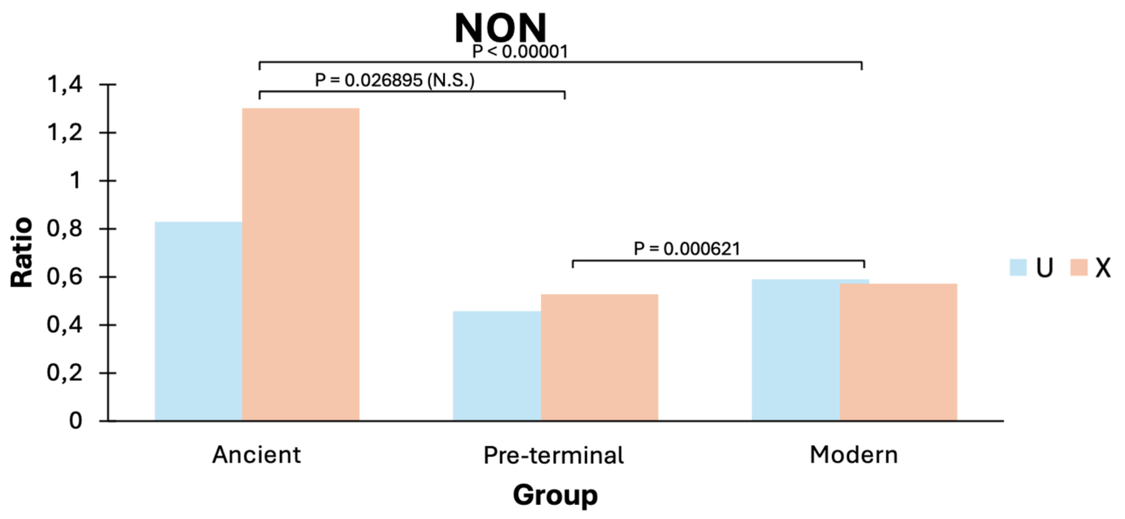

Figure S63. Comparison of the ratio of mitochondrial non-synonymous mutations measured against synonymous mutations between pairs of haplogroups, which in this case are **U/X**. *P*-values between ratios in the three analysed groups are shown above the bars, where N.S. corresponds to non-significant.

Tree Comparison – JT/N1

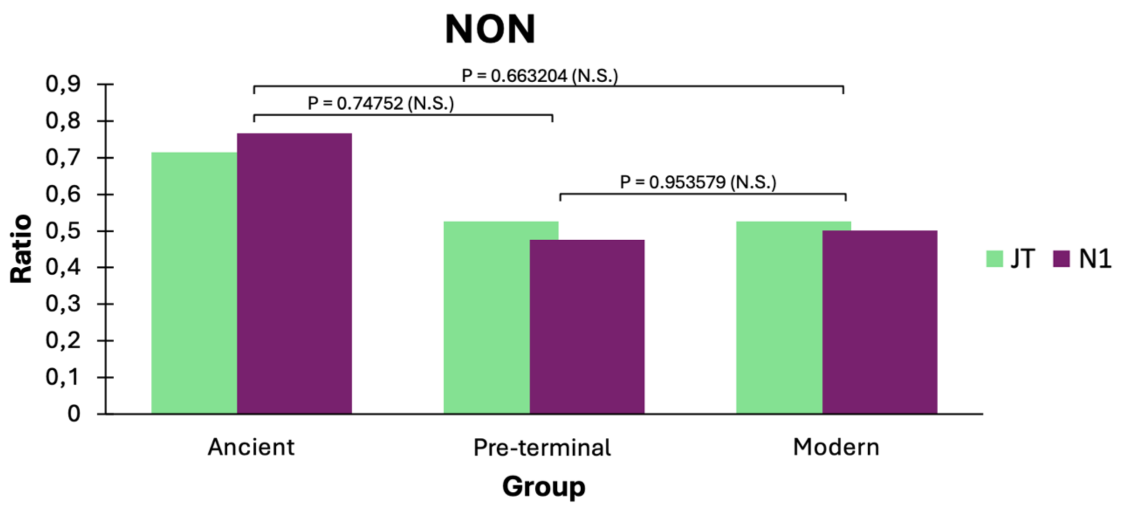

Figure S64. Comparison of the ratio of mitochondrial non-synonymous mutations measured against synonymous mutations between pairs of haplogroups, which in this case are **JT/N1**. *P*-values between ratios in the three analysed groups are shown above the bars, where N.S. corresponds to non-significant.

Tree Comparison – JT/N2

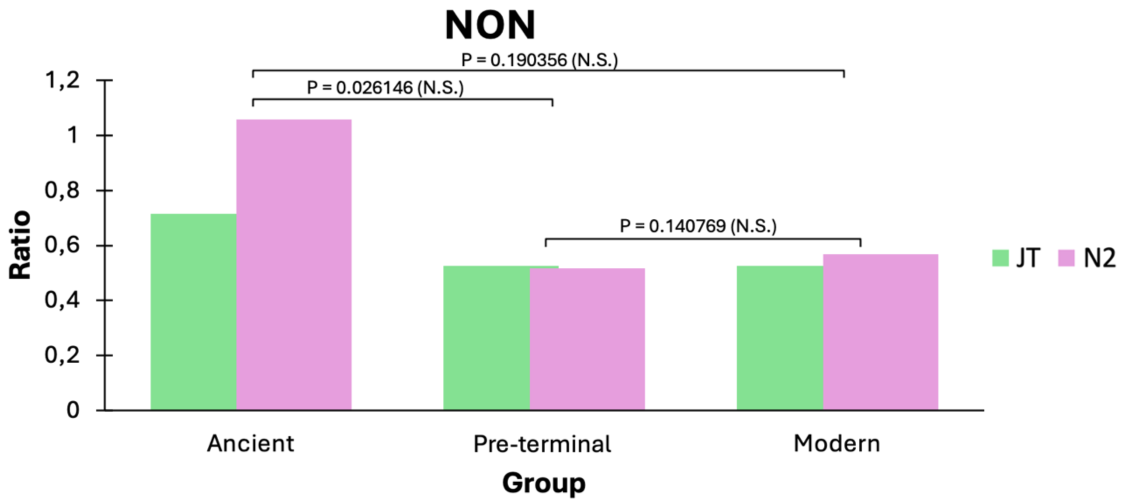

Figure S65. Comparison of the ratio of mitochondrial non-synonymous mutations measured against synonymous mutations between pairs of haplogroups, which in this case are **JT/N2**. *P*-values between ratios in the three analysed groups are shown above the bars, where N.S. corresponds to non-significant.

Tree Comparison – JT/X

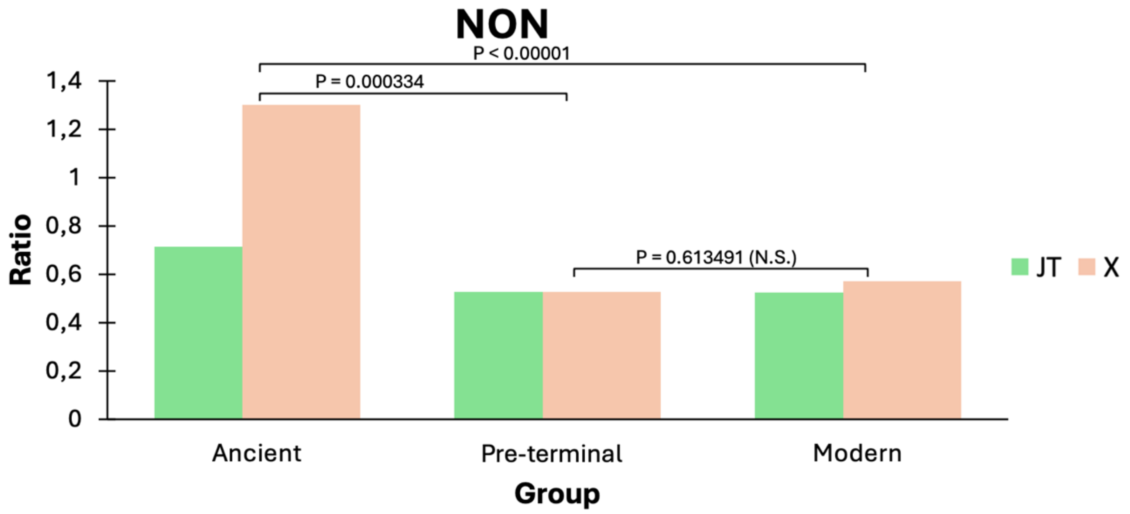

Figure S66. Comparison of the ratio of mitochondrial non-synonymous mutations measured against synonymous mutations between pairs of haplogroups, which in this case are **JT/X**. *P*-values between ratios in the three analysed groups are shown above the bars, where N.S. corresponds to non-significant.

Tree Comparison – N1/N2

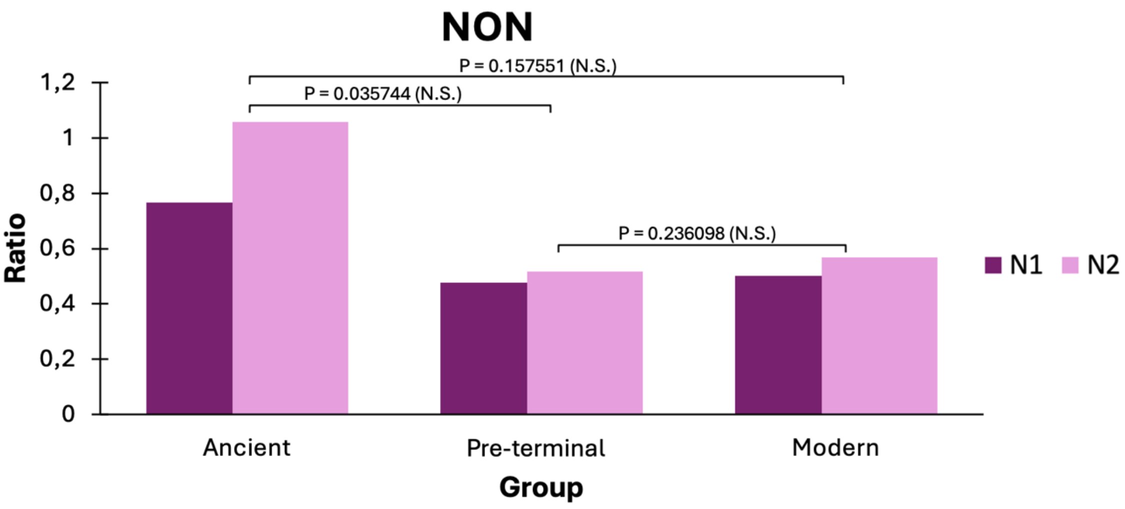

Figure S67. Comparison of the ratio of mitochondrial non-synonymous mutations measured against synonymous mutations between pairs of haplogroups, which in this case are **N1/N2**. *P*-values between ratios in the three analysed groups are shown above the bars, where N.S. corresponds to non-significant.

Tree Comparison – N1/X

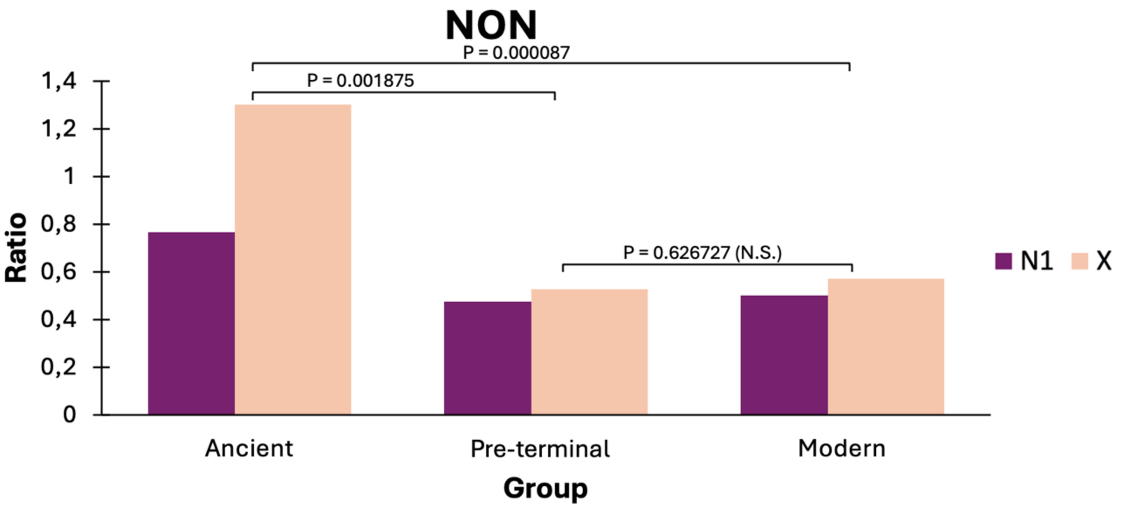

Figure S68. Comparison of the ratio of mitochondrial non-synonymous mutations measured against synonymous mutations between pairs of haplogroups, which in this case are **N1/X**. *P*-values between ratios in the three analysed groups are shown above the bars, where N.S. corresponds to non-significant.

Tree Comparison – N2/X

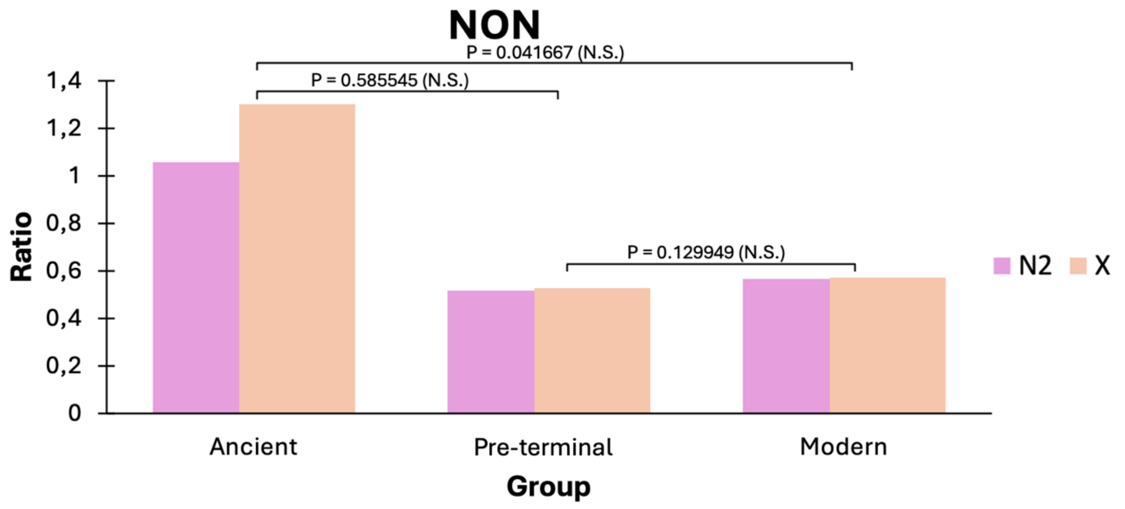

Figure S69. Comparison of the ratio of mitochondrial non-synonymous mutations measured against synonymous mutations between pairs of haplogroups, which in this case are **N2/X**. *P*-values between ratios in the three analysed groups are shown above the bars, where N.S. corresponds to non-significant.

Tree Comparison – R0/U

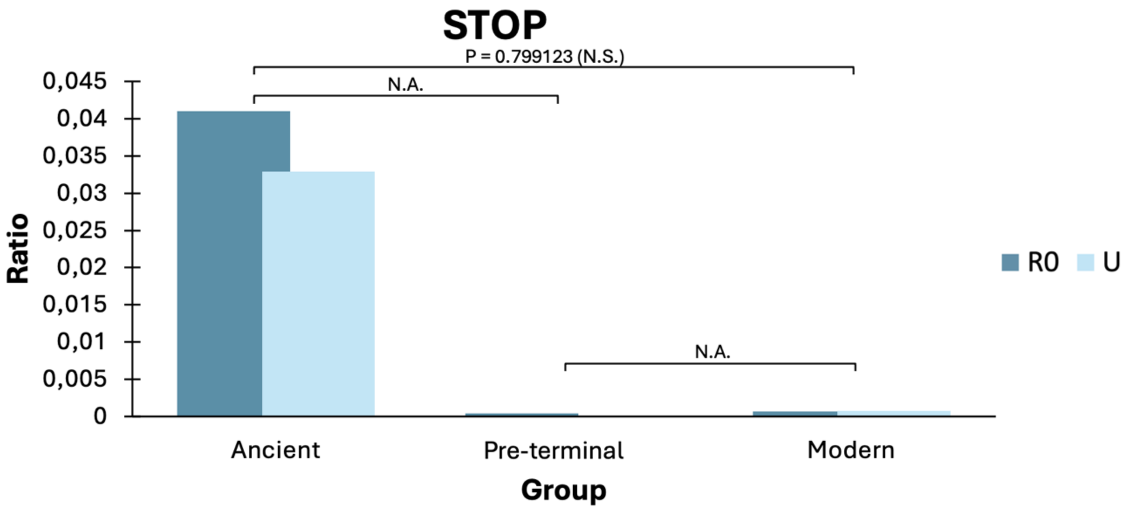

Figure S70. Comparison of the ratio of mitochondrial nonsense mutations measured against synonymous mutations between pairs of haplogroups, which in this case are **R0/U**. *P*-values between ratios in the three analysed groups are shown above the bars, where N.S. corresponds to non-significant.

Tree Comparison – R0/JT

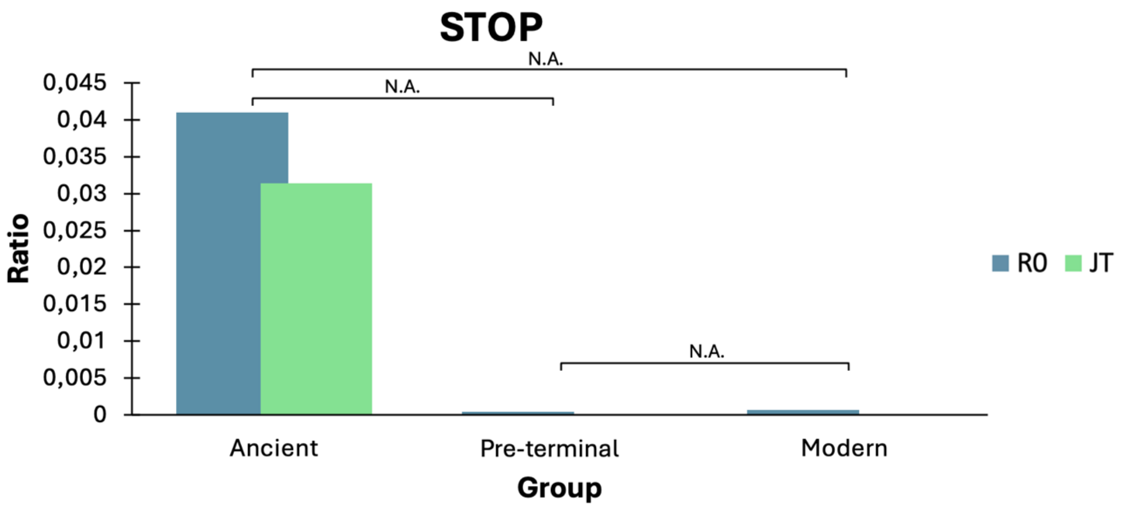

Figure S71. Comparison of the ratio of mitochondrial nonsense mutations measured against synonymous mutations between pairs of haplogroups, which in this case are **R0/JT**. *P*-values between ratios in the three analysed groups are shown above the bars, where N.S. corresponds to non-significant.

Tree Comparison – R0/N1

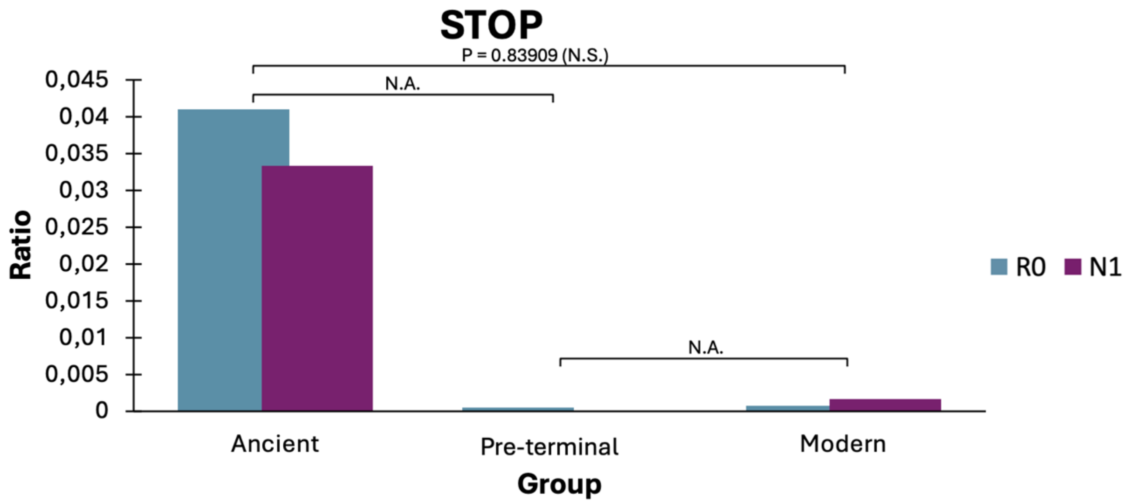

Figure S72. Comparison of the ratio of mitochondrial nonsense mutations measured against synonymous mutations between pairs of haplogroups, which in this case are **R0/N1**. *P*-values between ratios in the three analysed groups are shown above the bars, where N.S. corresponds to non-significant.

Tree Comparison – R0/N2

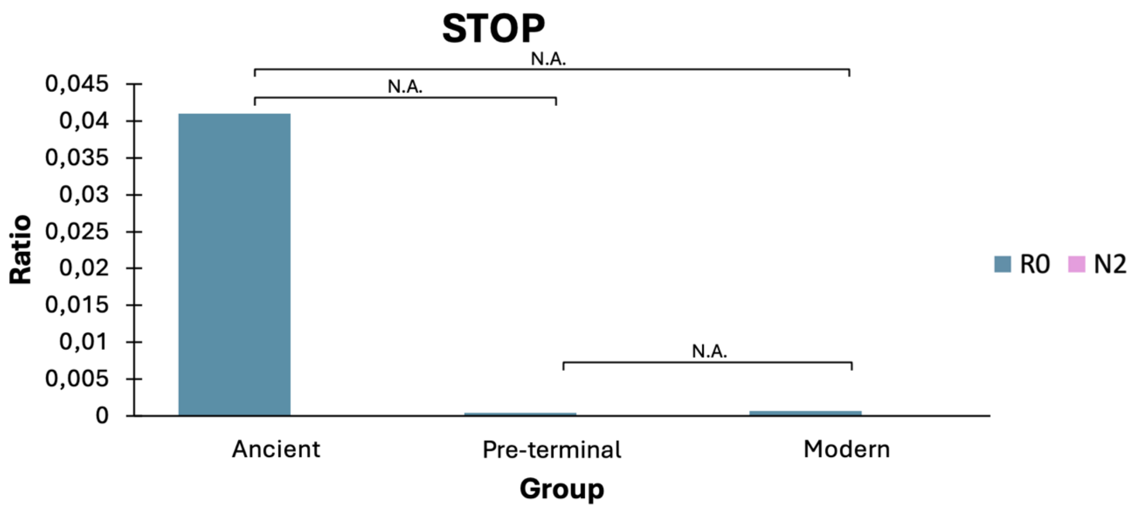

Figure S73. Comparison of the ratio of mitochondrial nonsense mutations measured against synonymous mutations between pairs of haplogroups, which in this case are **R0/N2**. *P*-values between ratios in the three analysed groups are shown above the bars, where N.S. corresponds to non-significant.

Tree Comparison – R0/X

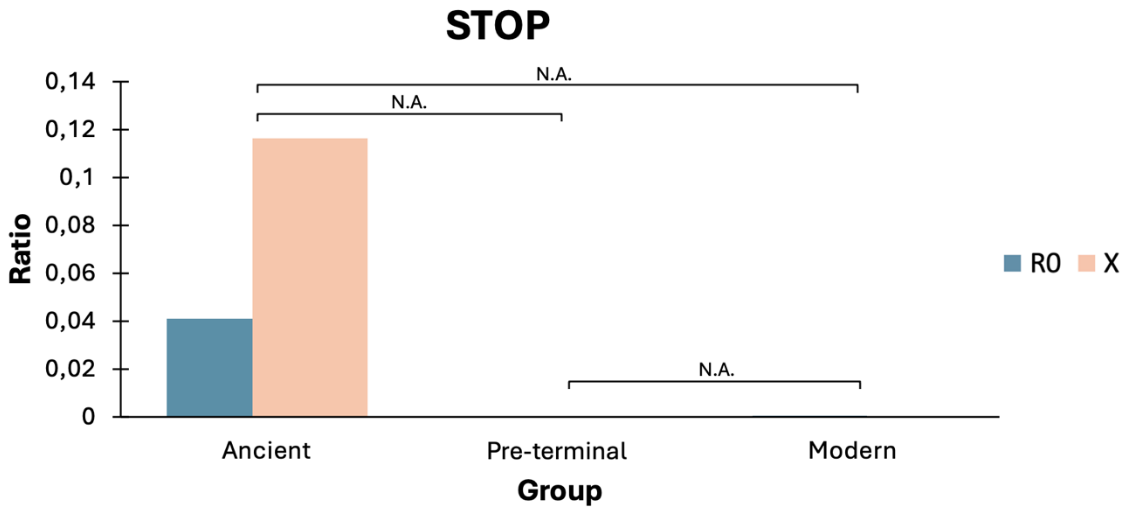

Figure S74. Comparison of the ratio of mitochondrial nonsense mutations measured against synonymous mutations between pairs of haplogroups, which in this case are **R0/X**. *P*-values between ratios in the three analysed groups are shown above the bars, where N.S. corresponds to non-significant.

Tree Comparison – U/JT

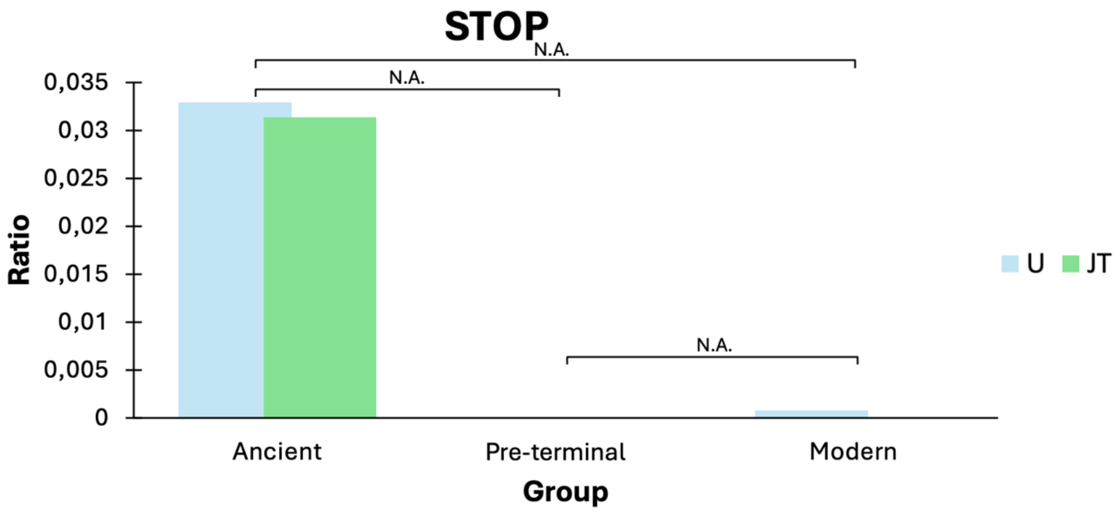

Figure S75. Comparison of the ratio of mitochondrial nonsense mutations measured against synonymous mutations between pairs of haplogroups, which in this case are **U/JT**. *P*-values between ratios in the three analysed groups are shown above the bars, where N.S. corresponds to non-significant.

Tree Comparison – U/N1

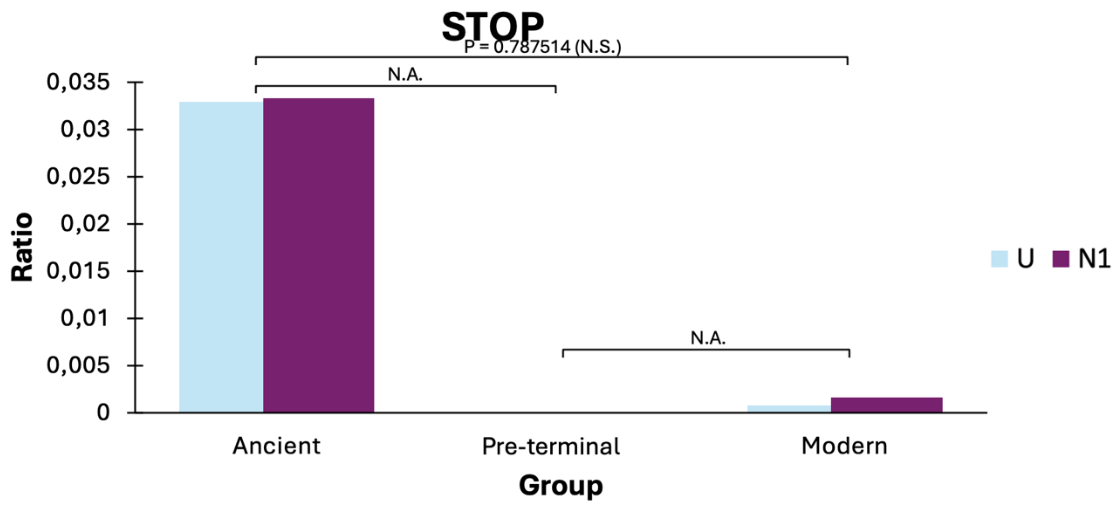

Figure S76. Comparison of the ratio of mitochondrial nonsense mutations measured against synonymous mutations between pairs of haplogroups, which in this case are **U/N1**. *P*-values between ratios in the three analysed groups are shown above the bars, where N.S. corresponds to non-significant.

Tree Comparison – U/N2

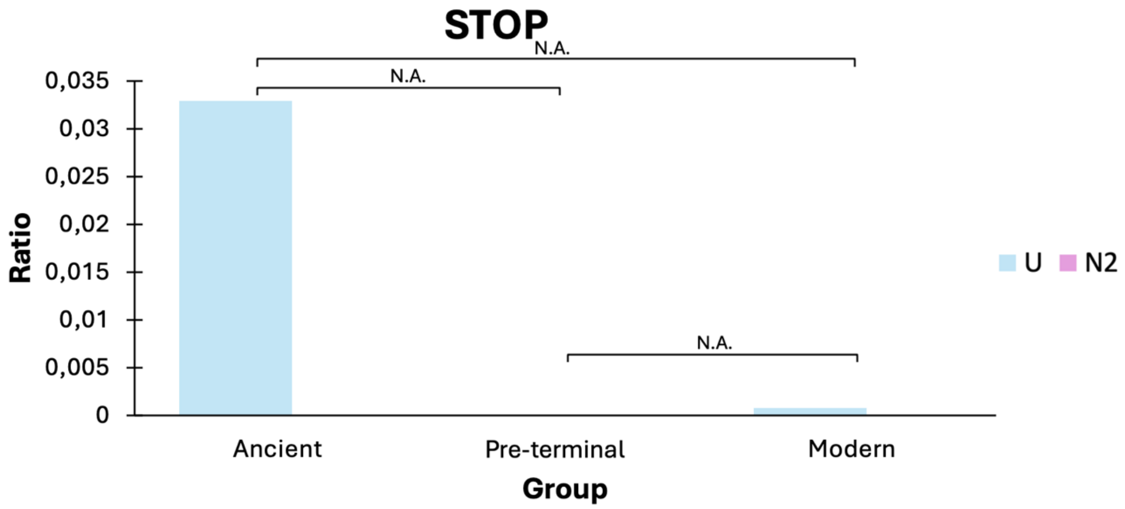

Figure S77. Comparison of the ratio of mitochondrial nonsense mutations measured against synonymous mutations between pairs of haplogroups, which in this case are **U/N2**. *P*-values between ratios in the three analysed groups are shown above the bars, where N.S. corresponds to non-significant.

Tree Comparison – U/X

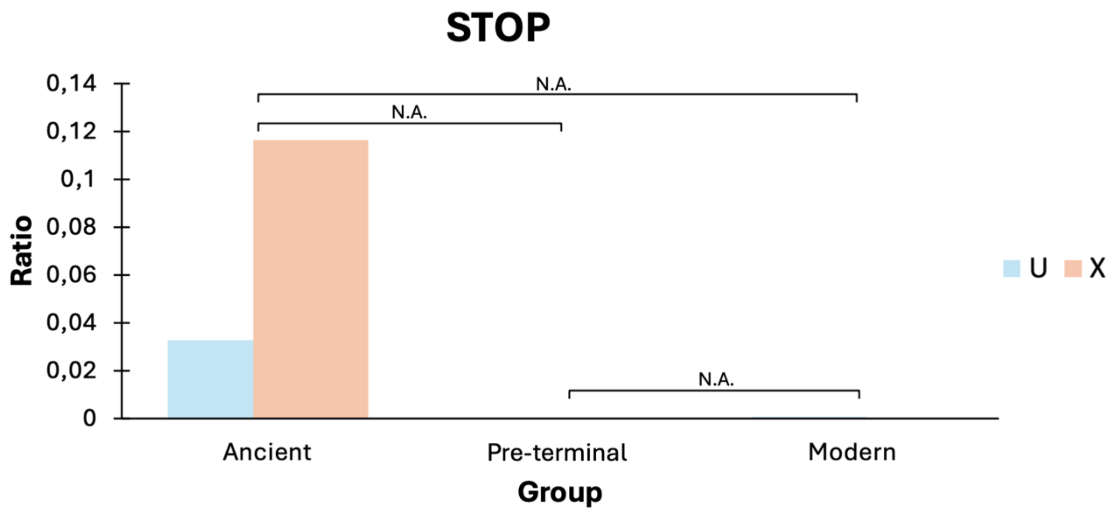

Figure S78. Comparison of the ratio of mitochondrial nonsense mutations measured against synonymous mutations between pairs of haplogroups, which in this case are **U/X**. *P*-values between ratios in the three analysed groups are shown above the bars, where N.S. corresponds to non-significant.

Tree Comparison – JT/N1

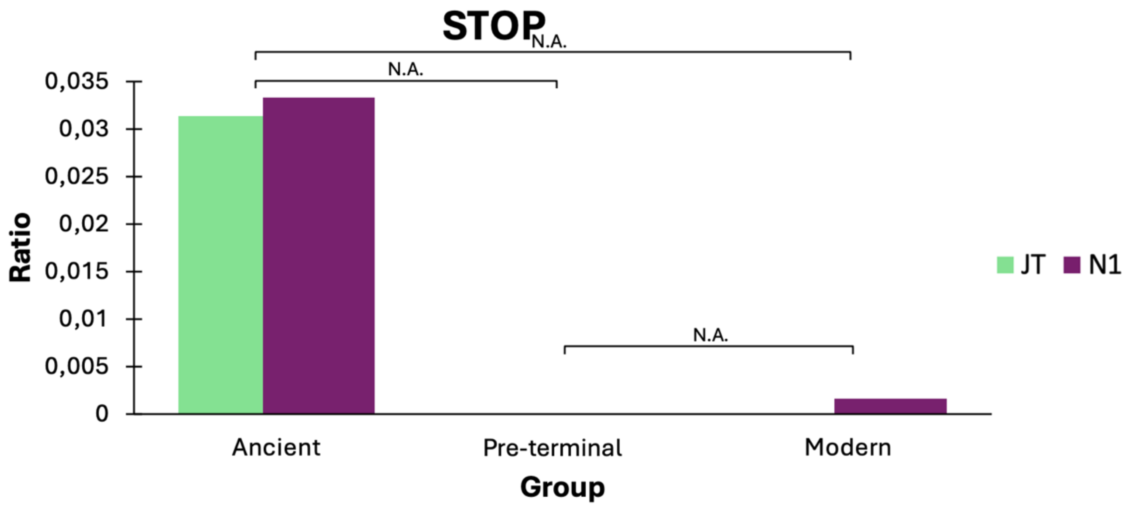

Figure S79. Comparison of the ratio of mitochondrial nonsense mutations measured against synonymous mutations between pairs of haplogroups, which in this case are **JT/N1**. *P*-values between ratios in the three analysed groups are shown above the bars, where N.S. corresponds to non-significant.

Tree Comparison – JT/N2

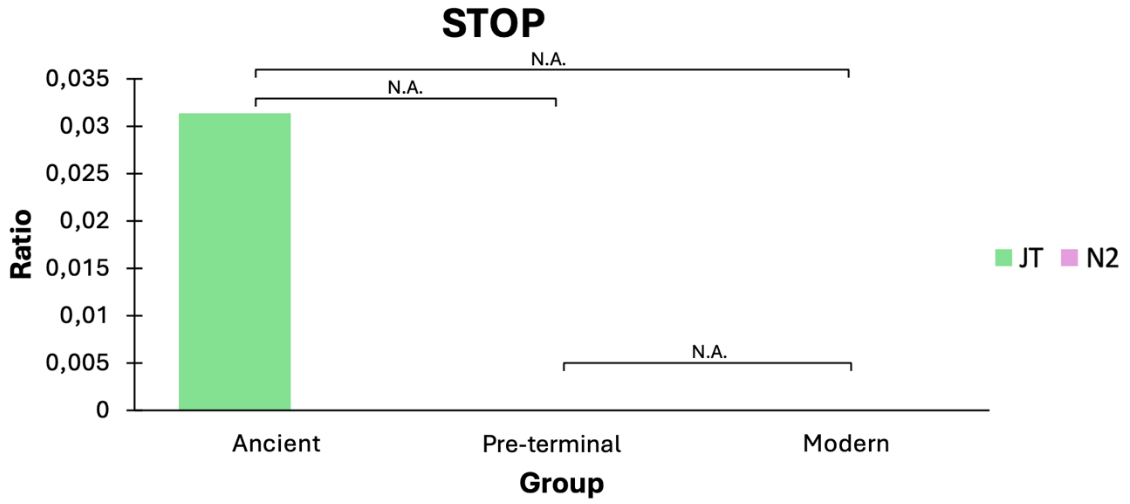

Figure S80. Comparison of the ratio of mitochondrial nonsense mutations measured against synonymous mutations between pairs of haplogroups, which in this case are **JT/N2**. *P*-values between ratios in the three analysed groups are shown above the bars, where N.S. corresponds to non-significant.

Tree Comparison – JT/X

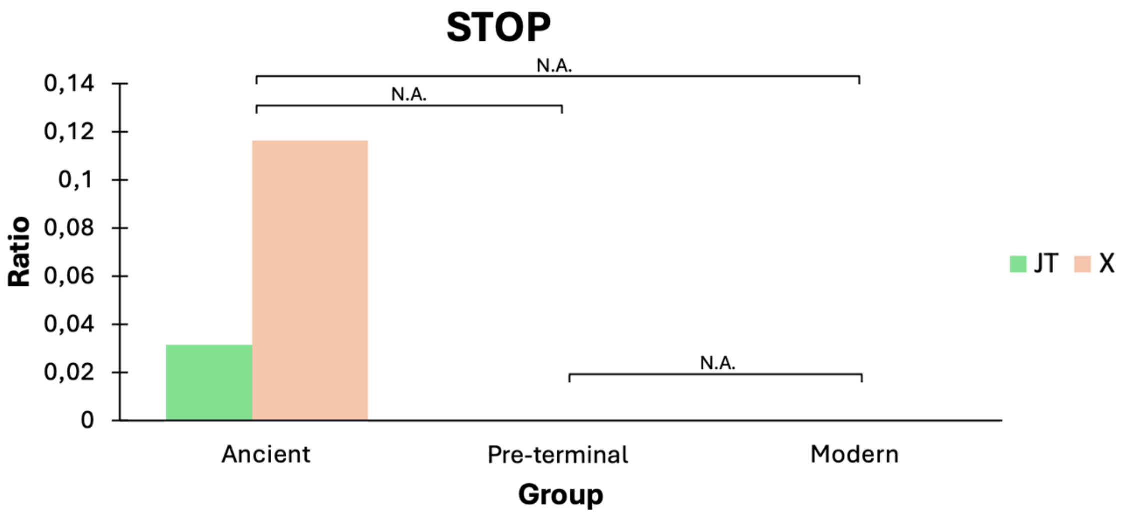

Figure S81. Comparison of the ratio of mitochondrial nonsense mutations measured against synonymous mutations between pairs of haplogroups, which in this case are **JT/X**. *P*-values between ratios in the three analysed groups are shown above the bars, where N.S. corresponds to non-significant.

Tree Comparison – N1/N2

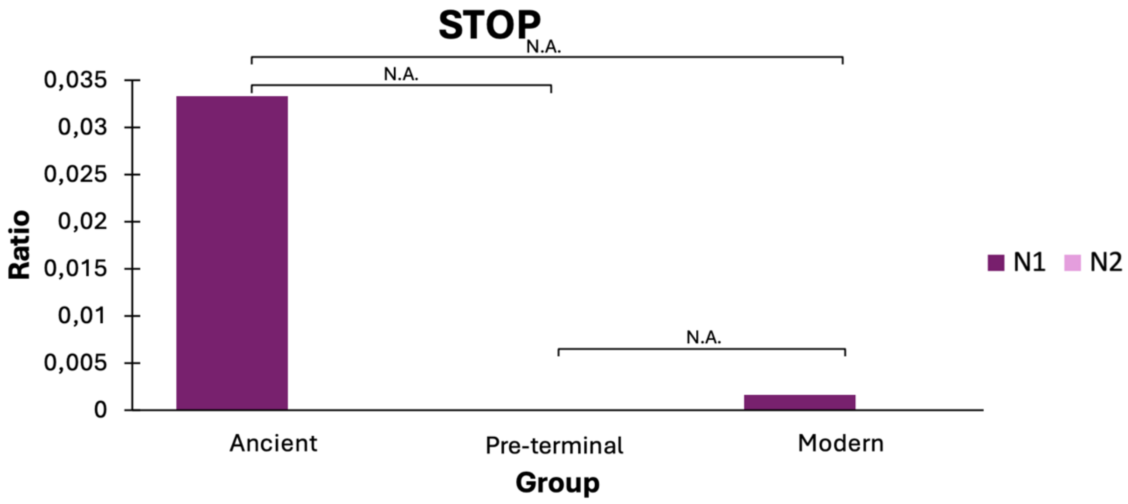

Figure S82. Comparison of the ratio of mitochondrial nonsense mutations measured against synonymous mutations between pairs of haplogroups, which in this case are **N1/N2**. *P*-values between ratios in the three analysed groups are shown above the bars, where N.S. corresponds to non-significant.

Tree Comparison – N1/X

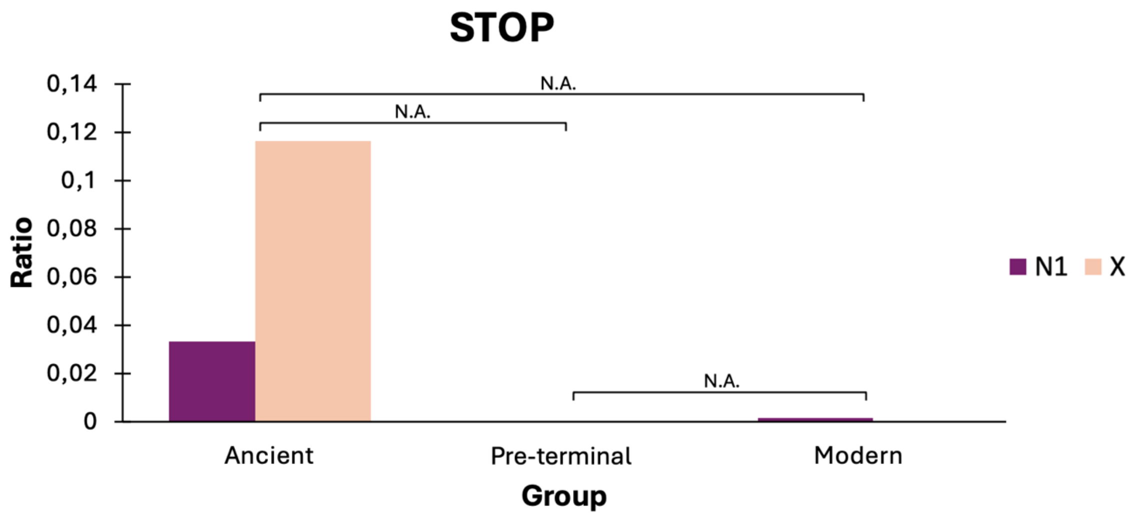

Figure S83. Comparison of the ratio of mitochondrial nonsense mutations measured against synonymous mutations between pairs of haplogroups, which in this case are **N1/X**. *P*-values between ratios in the three analysed groups are shown above the bars, where N.S. corresponds to non-significant.

Tree Comparison – N2/X

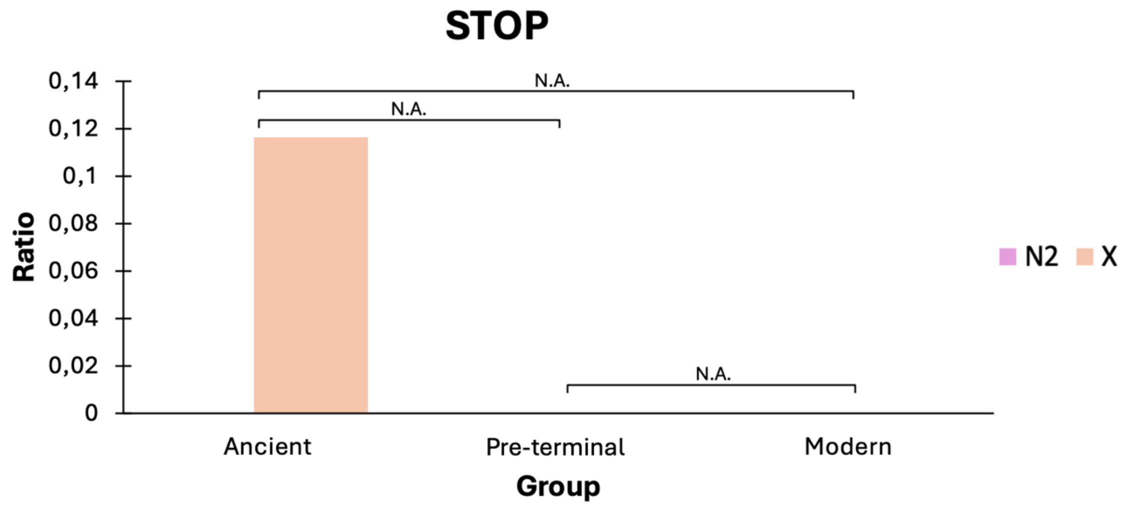

Figure S84. Comparison of the ratio of mitochondrial nonsense mutations measured against synonymous mutations between pairs of haplogroups, which in this case are **N2/X**. *P*-values between ratios in the three analysed groups are shown above the bars, where N.S. corresponds to non-significant.

Table S1. List of all detected nonsense and non-synonymous mutations, including their pathogenicity score using PolyPhen-2, SIFT and MutPred2.

Classification of mutations according phylogenetic position	Haplogroup	Ancestral base	Position	Derived base	Gene	Non-synonymous and nonsense variants	Aminoacid change	PolyPhen-2	SIFT	MutPred2
Ancient terminal	R0	T	3308	C	ND1	Non-synonymous	M1T	0.992	0	0.8
Modern terminal	R0	T	3308	C	ND1	Non-synonymous	M1T	0.992	0	0.8
Modern terminal	R0	T	3308	C	ND1	Non-synonymous	M1T	0.992	0	0.8
Modern terminal	R0	T	3308	C	ND1	Non-synonymous	M1T	0.992	0	0.8
Ancient terminal	U	T	3308	C	ND1	Non-synonymous	M1T	0.992	0	0.8
Modern terminal	U	T	3308	C	ND1	Non-synonymous	M1T	0.992	0	0.8
Modern terminal	U	T	3308	C	ND1	Non-synonymous	M1T	0.992	0	0.8
Modern terminal	U	T	3308	C	ND1	Non-synonymous	M1T	0.992	0	0.8
Modern terminal	U	T	3308	C	ND1	Non-synonymous	M1T	0.992	0	0.8
Modern terminal	U	T	3308	C	ND1	Non-synonymous	M1T	0.992	0	0.8
Modern terminal	U	T	3308	C	ND1	Non-synonymous	M1T	0.992	0	0.8
Pre-terminal	U	T	3308	C	ND1	Non-synonymous	M1T	0.992	0	0.8
Pre-terminal	U	T	3308	C	ND1	Non-synonymous	M1T	0.992	0	0.8
Modern terminal	JT	T	3308	C	ND1	Non-synonymous	M1T	0.992	0	0.8
Modern terminal	JT	T	3308	C	ND1	Non-synonymous	M1T	0.992	0	0.8
Modern terminal	JT	T	3308	C	ND1	Non-synonymous	M1T	0.992	0	0.8
Pre-terminal	JT	T	3308	G	ND1	Non-synonymous	M1R	0.992	0	0.897
Pre-terminal	JT	T	3308	C	ND1	Non-synonymous	M1T	0.992	0	0.8
Modern terminal	N1	T	3308	C	ND1	Non-synonymous	M1T	0.992	0	0.8
Modern terminal	N1	T	3308	C	ND1	Non-synonymous	M1T	0.992	0	0.8
Modern terminal	X	T	3308	C	ND1	Non-synonymous	M1T	0.992	0	0.8
Modern terminal	R0	C	3310	T	ND1	Non-synonymous	P2S	0.181	0.4	0.246
Pre-terminal	R0	C	3310	T	ND1	Non-synonymous	P2S	0.181	0.4	0.246
Pre-terminal	U	C	3310	T	ND1	Non-synonymous	P2S	0.181	0.4	0.246
Pre-terminal	U	C	3310	T	ND1	Non-synonymous	P2S	0.181	0.4	0.246
Pre-terminal	R0	C	3311	T	ND1	Non-synonymous	P2L	0.001	0.64	0.186
Modern terminal	R0	G	3316	A	ND1	Non-synonymous	A4T	0	0.39	0.091
Modern terminal	R0	G	3316	A	ND1	Non-synonymous	A4T	0	0.39	0.091
Modern terminal	R0	G	3316	A	ND1	Non-synonymous	A4T	0	0.39	0.091
Modern terminal	R0	G	3316	A	ND1	Non-synonymous	A4T	0	0.39	0.091
Modern terminal	R0	G	3316	A	ND1	Non-synonymous	A4T	0	0.39	0.091
Modern terminal	R0	G	3316	A	ND1	Non-synonymous	A4T	0	0.39	0.091
Modern terminal	R0	G	3316	A	ND1	Non-synonymous	A4T	0	0.39	0.091
Modern terminal	R0	G	3316	A	ND1	Non-synonymous	A4T	0	0.39	0.091
Modern terminal	R0	G	3316	A	ND1	Non-synonymous	A4T	0	0.39	0.091
Modern terminal	R0	G	3316	A	ND1	Non-synonymous	A4T	0	0.39	0.091
Modern terminal	R0	G	3316	A	ND1	Non-synonymous	A4T	0	0.39	0.091
Pre-terminal	R0	G	3316	A	ND1	Non-synonymous	A4T	0	0.39	0.091
Pre-terminal	R0	G	3316	A	ND1	Non-synonymous	A4T	0	0.39	0.091

Pre-terminal	R0	G	3316	A	ND1	Non-synonymous	A4T	0	0.39	0.091
Pre-terminal	R0	G	3316	A	ND1	Non-synonymous	A4T	0	0.39	0.091
Pre-terminal	R0	G	3316	A	ND1	Non-synonymous	A4T	0	0.39	0.091
Pre-terminal	R0	G	3316	A	ND1	Non-synonymous	A4T	0	0.39	0.091
Ancient terminal	U	G	3316	A	ND1	Non-synonymous	A4T	0	0.39	0.091
Ancient terminal	U	G	3316	A	ND1	Non-synonymous	A4T	0	0.39	0.091
Modern terminal	U	G	3316	A	ND1	Non-synonymous	A4T	0	0.39	0.091
Modern terminal	U	G	3316	A	ND1	Non-synonymous	A4T	0	0.39	0.091
Modern terminal	U	G	3316	A	ND1	Non-synonymous	A4T	0	0.39	0.091
Modern terminal	U	G	3316	A	ND1	Non-synonymous	A4T	0	0.39	0.091
Modern terminal	U	G	3316	A	ND1	Non-synonymous	A4T	0	0.39	0.091
Modern terminal	U	G	3316	A	ND1	Non-synonymous	A4T	0	0.39	0.091
Modern terminal	U	G	3316	A	ND1	Non-synonymous	A4T	0	0.39	0.091
Modern terminal	U	G	3316	A	ND1	Non-synonymous	A4T	0	0.39	0.091
Modern terminal	U	G	3316	A	ND1	Non-synonymous	A4T	0	0.39	0.091
Modern terminal	U	G	3316	A	ND1	Non-synonymous	A4T	0	0.39	0.091
Modern terminal	U	G	3316	A	ND1	Non-synonymous	A4T	0	0.39	0.091
Modern terminal	U	G	3316	A	ND1	Non-synonymous	A4T	0	0.39	0.091
Modern terminal	U	G	3316	A	ND1	Non-synonymous	A4T	0	0.39	0.091
Modern terminal	U	G	3316	A	ND1	Non-synonymous	A4T	0	0.39	0.091
Modern terminal	U	G	3316	A	ND1	Non-synonymous	A4T	0	0.39	0.091
Modern terminal	U	G	3316	A	ND1	Non-synonymous	A4T	0	0.39	0.091
Pre-terminal	U	G	3316	A	ND1	Non-synonymous	A4T	0	0.39	0.091
Pre-terminal	U	G	3316	A	ND1	Non-synonymous	A4T	0	0.39	0.091
Pre-terminal	U	G	3316	A	ND1	Non-synonymous	A4T	0	0.39	0.091
Pre-terminal	U	G	3316	A	ND1	Non-synonymous	A4T	0	0.39	0.091
Pre-terminal	U	G	3316	A	ND1	Non-synonymous	A4T	0	0.39	0.091
Pre-terminal	U	G	3316	A	ND1	Non-synonymous	A4T	0	0.39	0.091
Ancient terminal	JT	G	3316	A	ND1	Non-synonymous	A4T	0	0.39	0.091
Modern terminal	JT	G	3316	A	ND1	Non-synonymous	A4T	0	0.39	0.091
Modern terminal	JT	G	3316	A	ND1	Non-synonymous	A4T	0	0.39	0.091
Modern terminal	JT	G	3316	A	ND1	Non-synonymous	A4T	0	0.39	0.091
Modern terminal	JT	G	3316	A	ND1	Non-synonymous	A4T	0	0.39	0.091
Modern terminal	JT	G	3316	A	ND1	Non-synonymous	A4T	0	0.39	0.091
Modern terminal	JT	G	3316	A	ND1	Non-synonymous	A4T	0	0.39	0.091
Modern terminal	JT	G	3316	A	ND1	Non-synonymous	A4T	0	0.39	0.091
Pre-terminal	JT	G	3316	A	ND1	Non-synonymous	A4T	0	0.39	0.091
Ancient terminal	N1	G	3316	A	ND1	Non-synonymous	A4T	0	0.39	0.091
Modern terminal	N1	G	3316	A	ND1	Non-synonymous	A4T	0	0.39	0.091
Modern terminal	N1	G	3316	A	ND1	Non-synonymous	A4T	0	0.39	0.091
Pre-terminal	N1	G	3316	A	ND1	Non-synonymous	A4T	0	0.39	0.091
Modern terminal	X	G	3316	A	ND1	Non-synonymous	A4T	0	0.39	0.091
Pre-terminal	X	G	3316	A	ND1	Non-synonymous	A4T	0	0.39	0.091
Pre-terminal	N2	G	3316	A	ND1	Non-synonymous	A4T	0	0.39	0.091
Modern terminal	R0	C	3317	T	ND1	Non-synonymous	A4V	0	0.94	0.113
Modern terminal	JT	C	3317	T	ND1	Non-synonymous	A4V	0	0.94	0.113
Ancient terminal	JT	A	3320	C	ND1	Non-synonymous	N5T	0.994	0.02	0.166
Ancient terminal	R0	A	3334	T	ND1	Non-synonymous	I10F	0.541	0.05	0.271
Modern terminal	U	A	3334	G	ND1	Non-synonymous	I10V	0.001	0.18	0.075
Modern terminal	R0	T	3335	C	ND1	Non-synonymous	I10T	0.02	0.07	0.154

Modern terminal	R0	T	3335	C	ND1	Non-synonymous	I10T	0.02	0.07	0.154
Modern terminal	R0	T	3335	C	ND1	Non-synonymous	I10T	0.02	0.07	0.154
Modern terminal	R0	T	3335	C	ND1	Non-synonymous	I10T	0.02	0.07	0.154
Modern terminal	R0	T	3335	C	ND1	Non-synonymous	I10T	0.02	0.07	0.154
Modern terminal	R0	T	3335	C	ND1	Non-synonymous	I10T	0.02	0.07	0.154
Pre-terminal	R0	T	3335	C	ND1	Non-synonymous	I10T	0.02	0.07	0.154
Pre-terminal	R0	T	3335	C	ND1	Non-synonymous	I10T	0.02	0.07	0.154
Pre-terminal	R0	T	3335	C	ND1	Non-synonymous	I10T	0.02	0.07	0.154
Pre-terminal	R0	T	3335	C	ND1	Non-synonymous	I10T	0.02	0.07	0.154
Ancient terminal	U	T	3335	C	ND1	Non-synonymous	I10T	0.02	0.07	0.154
Modern terminal	U	T	3335	A	ND1	Non-synonymous	I10N	0.994	0	0.389
Modern terminal	U	T	3335	C	ND1	Non-synonymous	I10T	0.02	0.07	0.154
Modern terminal	U	T	3335	C	ND1	Non-synonymous	I10T	0.02	0.07	0.154
Modern terminal	U	T	3335	C	ND1	Non-synonymous	I10T	0.02	0.07	0.154
Modern terminal	U	T	3335	C	ND1	Non-synonymous	I10T	0.02	0.07	0.154
Pre-terminal	U	T	3335	C	ND1	Non-synonymous	I10T	0.02	0.07	0.154
Modern terminal	JT	T	3335	C	ND1	Non-synonymous	I10T	0.02	0.07	0.154
Modern terminal	JT	T	3335	C	ND1	Non-synonymous	I10T	0.02	0.07	0.154
Modern terminal	JT	T	3335	C	ND1	Non-synonymous	I10T	0.02	0.07	0.154
Pre-terminal	JT	T	3335	C	ND1	Non-synonymous	I10T	0.02	0.07	0.154
Pre-terminal	N1	T	3335	C	ND1	Non-synonymous	I10T	0.02	0.07	0.154
Modern terminal	X	T	3335	C	ND1	Non-synonymous	I10T	0.02	0.07	0.154
Modern terminal	X	T	3335	C	ND1	Non-synonymous	I10T	0.02	0.07	0.154
Modern terminal	R0	G	3337	A	ND1	Non-synonymous	V11M	0.004	0.09	0.158
Modern terminal	R0	G	3337	A	ND1	Non-synonymous	V11M	0.004	0.09	0.158
Modern terminal	R0	G	3337	A	ND1	Non-synonymous	V11M	0.004	0.09	0.158
Modern terminal	R0	G	3337	A	ND1	Non-synonymous	V11M	0.004	0.09	0.158
Pre-terminal	R0	G	3337	A	ND1	Non-synonymous	V11M	0.004	0.09	0.158
Pre-terminal	R0	G	3337	A	ND1	Non-synonymous	V11M	0.004	0.09	0.158
Modern terminal	U	G	3337	A	ND1	Non-synonymous	V11M	0.004	0.09	0.158
Modern terminal	U	G	3337	A	ND1	Non-synonymous	V11M	0.004	0.09	0.158
Modern terminal	U	G	3337	A	ND1	Non-synonymous	V11M	0.004	0.09	0.158
Pre-terminal	U	G	3337	A	ND1	Non-synonymous	V11M	0.004	0.09	0.158
Pre-terminal	U	G	3337	A	ND1	Non-synonymous	V11M	0.004	0.09	0.158
Pre-terminal	U	G	3337	A	ND1	Non-synonymous	V11M	0.004	0.09	0.158
Ancient terminal	JT	G	3337	A	ND1	Non-synonymous	V11M	0.004	0.09	0.158
Modern terminal	JT	G	3337	A	ND1	Non-synonymous	V11M	0.004	0.09	0.158
Modern terminal	JT	G	3337	A	ND1	Non-synonymous	V11M	0.004	0.09	0.158
Modern terminal	JT	G	3337	A	ND1	Non-synonymous	V11M	0.004	0.09	0.158
Modern terminal	JT	G	3337	A	ND1	Non-synonymous	V11M	0.004	0.09	0.158
Modern terminal	JT	G	3337	A	ND1	Non-synonymous	V11M	0.004	0.09	0.158
Modern terminal	JT	G	3337	A	ND1	Non-synonymous	V11M	0.004	0.09	0.158
Modern terminal	JT	G	3337	A	ND1	Non-synonymous	V11M	0.004	0.09	0.158
Modern terminal	JT	G	3337	A	ND1	Non-synonymous	V11M	0.004	0.09	0.158
Modern terminal	JT	G	3337	T	ND1	Non-synonymous	V11L	0	0.45	0.107
Modern terminal	N2	G	3337	A	ND1	Non-synonymous	V11M	0.004	0.09	0.158
Modern terminal	N2	G	3337	A	ND1	Non-synonymous	V11M	0.004	0.09	0.158
Pre-terminal	N2	G	3337	A	ND1	Non-synonymous	V11M	0.004	0.09	0.158
Modern terminal	R0	T	3338	C	ND1	Non-synonymous	V11A	0.016	0.14	0.185
Modern terminal	R0	T	3338	C	ND1	Non-synonymous	V11A	0.016	0.14	0.185

Modern terminal	R0	T	3338	C	ND1	Non-synonymous	V11A	0.016	0.14	0.185
Modern terminal	R0	T	3338	C	ND1	Non-synonymous	V11A	0.016	0.14	0.185
Modern terminal	R0	T	3338	C	ND1	Non-synonymous	V11A	0.016	0.14	0.185
Modern terminal	R0	T	3338	G	ND1	Non-synonymous	V11G	0.954	0	0.307
Modern terminal	R0	T	3338	C	ND1	Non-synonymous	V11A	0.016	0.14	0.185
Pre-terminal	R0	T	3338	C	ND1	Non-synonymous	V11A	0.016	0.14	0.185
Pre-terminal	R0	T	3338	C	ND1	Non-synonymous	V11A	0.016	0.14	0.185
Pre-terminal	R0	T	3338	C	ND1	Non-synonymous	V11A	0.016	0.14	0.185
Modern terminal	U	T	3338	C	ND1	Non-synonymous	V11A	0.016	0.14	0.185
Modern terminal	U	T	3338	C	ND1	Non-synonymous	V11A	0.016	0.14	0.185
Modern terminal	U	T	3338	C	ND1	Non-synonymous	V11A	0.016	0.14	0.185
Modern terminal	U	T	3338	C	ND1	Non-synonymous	V11A	0.016	0.14	0.185
Modern terminal	U	T	3338	C	ND1	Non-synonymous	V11A	0.016	0.14	0.185
Modern terminal	U	T	3338	C	ND1	Non-synonymous	V11A	0.016	0.14	0.185
Modern terminal	U	T	3338	C	ND1	Non-synonymous	V11A	0.016	0.14	0.185
Modern terminal	U	T	3338	C	ND1	Non-synonymous	V11A	0.016	0.14	0.185
Pre-terminal	U	T	3338	C	ND1	Non-synonymous	V11A	0.016	0.14	0.185
Pre-terminal	U	T	3338	C	ND1	Non-synonymous	V11A	0.016	0.14	0.185
Pre-terminal	U	T	3338	C	ND1	Non-synonymous	V11A	0.016	0.14	0.185
Modern terminal	JT	T	3338	C	ND1	Non-synonymous	V11A	0.016	0.14	0.185
Modern terminal	JT	T	3338	C	ND1	Non-synonymous	V11A	0.016	0.14	0.185
Modern terminal	JT	T	3338	C	ND1	Non-synonymous	V11A	0.016	0.14	0.185
Modern terminal	JT	T	3338	C	ND1	Non-synonymous	V11A	0.016	0.14	0.185
Modern terminal	JT	T	3338	C	ND1	Non-synonymous	V11A	0.016	0.14	0.185
Modern terminal	JT	T	3338	C	ND1	Non-synonymous	V11A	0.016	0.14	0.185
Pre-terminal	JT	T	3338	C	ND1	Non-synonymous	V11A	0.016	0.14	0.185
Pre-terminal	JT	T	3338	C	ND1	Non-synonymous	V11A	0.016	0.14	0.185
Modern terminal	N1	T	3338	C	ND1	Non-synonymous	V11A	0.016	0.14	0.185
Pre-terminal	N1	T	3338	C	ND1	Non-synonymous	V11A	0.016	0.14	0.185
Ancient terminal	N2	T	3338	C	ND1	Non-synonymous	V11A	0.016	0.14	0.185
Ancient terminal	X	C	3341	T	ND1	Non-synonymous	P12L	0.743	0	0.459
Modern terminal	R0	A	3349	G	ND1	Non-synonymous	I15V	0	0.17	0.06
Modern terminal	R0	A	3349	G	ND1	Non-synonymous	I15V	0	0.17	0.06
Ancient terminal	JT	A	3349	G	ND1	Non-synonymous	I15V	0	0.17	0.06
Modern terminal	JT	A	3349	G	ND1	Non-synonymous	I15V	0	0.17	0.06
Pre-terminal	JT	A	3349	G	ND1	Non-synonymous	I15V	0	0.17	0.06
Modern terminal	R0	T	3350	C	ND1	Non-synonymous	I15T	0.04	0	0.19
Pre-terminal	R0	T	3350	C	ND1	Non-synonymous	I15T	0.04	0	0.19
Ancient terminal	U	T	3350	C	ND1	Non-synonymous	I15T	0.04	0	0.19
Pre-terminal	JT	T	3350	C	ND1	Non-synonymous	I15T	0.04	0	0.19
Modern terminal	N2	T	3350	C	ND1	Non-synonymous	I15T	0.04	0	0.19
Pre-terminal	N2	T	3350	C	ND1	Non-synonymous	I15T	0.04	0	0.19
Modern terminal	R0	C	3351	A	ND1	Non-synonymous	I15M	0.978	0.03	0.122
Modern terminal	R0	A	3355	G	ND1	Non-synonymous	M17V	1	1	0.072
Modern terminal	R0	A	3355	G	ND1	Non-synonymous	M17V	1	1	0.072
Modern terminal	R0	A	3355	G	ND1	Non-synonymous	M17V	1	1	0.072
Pre-terminal	R0	A	3355	G	ND1	Non-synonymous	M17V	1	1	0.072
Modern terminal	U	A	3355	G	ND1	Non-synonymous	M17V	1	1	0.072
Modern terminal	X	A	3355	G	ND1	Non-synonymous	M17V	1	1	0.072
Pre-terminal	X	A	3355	G	ND1	Non-synonymous	M17V	1	1	0.072

Pre-terminal	X	A	3355	G	ND1	Non-synonymous	M17V	1	1	0.072
Modern terminal	R0	G	3357	C	ND1	Non-synonymous	M17I	0.923	0.05	0.205
Modern terminal	R0	G	3358	A	ND1	Non-synonymous	A18T	1	0	0.332
Modern terminal	U	G	3358	A	ND1	Non-synonymous	A18T	1	0	0.332
Modern terminal	U	G	3358	A	ND1	Non-synonymous	A18T	1	0	0.332
Ancient terminal	X	C	3359	A	ND1	Non-synonymous	A18E	1	0	0.652
Modern terminal	U	C	3364	T	ND1	Non-synonymous	L20S	1	0	0.586
Modern terminal	U	C	3364	T	ND1	Non-synonymous	L20S	1	0	0.586
Ancient terminal	R0	T	3368	C	ND1	Non-synonymous	M21T	0.001	1	0.054
Modern terminal	R0	T	3368	C	ND1	Non-synonymous	M21T	0.001	1	0.054
Modern terminal	R0	T	3368	C	ND1	Non-synonymous	M21T	0.001	1	0.054
Modern terminal	R0	T	3368	C	ND1	Non-synonymous	M21T	0.001	1	0.054
Modern terminal	U	T	3368	C	ND1	Non-synonymous	M21T	0.001	1	0.054
Modern terminal	N1	T	3368	C	ND1	Non-synonymous	M21T	0.001	1	0.054
Pre-terminal	N2	T	3368	C	ND1	Non-synonymous	M21T	0.001	1	0.054
Ancient terminal	R0	C	3379	A	ND1	Nonsense				
Ancient terminal	JT	A	3383	G	ND1	Nonsense				
Pre-terminal	N2	A	3385	T	ND1	Non-synonymous	I27F	0.451	0.01	0.512
Modern terminal	R0	T	3386	C	ND1	Non-synonymous	I27T	0.027	0.07	0.406
Modern terminal	R0	T	3387	G	ND1	Non-synonymous	I27M	0.967	0.04	0.241
Modern terminal	JT	T	3387	A	ND1	Non-synonymous	I27M	0.967	0.04	0.241
Pre-terminal	R0	C	3388	A	ND1	Non-synonymous	L28I	0.997	0.39	0.315
Modern terminal	U	C	3388	A	ND1	Non-synonymous	L28I	0.997	0.39	0.315
Modern terminal	U	C	3388	A	ND1	Non-synonymous	L28I	0.997	0.39	0.315
Pre-terminal	U	C	3388	A	ND1	Non-synonymous	L28I	0.997	0.39	0.315
Modern terminal	R0	G	3391	A	ND1	Non-synonymous	G29S	1	0.05	0.64
Modern terminal	R0	G	3391	A	ND1	Non-synonymous	G29S	1	0.05	0.64
Modern terminal	R0	G	3391	A	ND1	Non-synonymous	G29S	1	0.05	0.64
Ancient terminal	U	G	3391	A	ND1	Non-synonymous	G29S	1	0.05	0.64
Modern terminal	U	G	3391	A	ND1	Non-synonymous	G29S	1	0.05	0.64
Modern terminal	U	G	3391	A	ND1	Non-synonymous	G29S	1	0.05	0.64
Modern terminal	U	G	3391	A	ND1	Non-synonymous	G29S	1	0.05	0.64
Ancient terminal	U	G	3392	C	ND1	Non-synonymous	G29A	0.998	0.22	0.686
Ancient terminal	R0	T	3394	C	ND1	Non-synonymous	Y30H	0.164	0.05	0.651
Ancient terminal	R0	T	3394	C	ND1	Non-synonymous	Y30H	0.164	0.05	0.651
Ancient terminal	R0	T	3394	C	ND1	Non-synonymous	Y30H	0.164	0.05	0.651
Ancient terminal	R0	T	3394	C	ND1	Non-synonymous	Y30H	0.164	0.05	0.651
Ancient terminal	R0	T	3394	C	ND1	Non-synonymous	Y30H	0.164	0.05	0.651
Modern terminal	R0	T	3394	C	ND1	Non-synonymous	Y30H	0.164	0.05	0.651
Modern terminal	R0	T	3394	C	ND1	Non-synonymous	Y30H	0.164	0.05	0.651
Modern terminal	R0	T	3394	C	ND1	Non-synonymous	Y30H	0.164	0.05	0.651
Modern terminal	R0	T	3394	C	ND1	Non-synonymous	Y30H	0.164	0.05	0.651
Modern terminal	R0	T	3394	C	ND1	Non-synonymous	Y30H	0.164	0.05	0.651
Modern terminal	R0	T	3394	C	ND1	Non-synonymous	Y30H	0.164	0.05	0.651
Modern terminal	R0	T	3394	C	ND1	Non-synonymous	Y30H	0.164	0.05	0.651
Modern terminal	R0	T	3394	C	ND1	Non-synonymous	Y30H	0.164	0.05	0.651
Modern terminal	R0	T	3394	C	ND1	Non-synonymous	Y30H	0.164	0.05	0.651
Modern terminal	R0	T	3394	C	ND1	Non-synonymous	Y30H	0.164	0.05	0.651
Modern terminal	R0	T	3394	C	ND1	Non-synonymous	Y30H	0.164	0.05	0.651
Modern terminal	R0	T	3394	C	ND1	Non-synonymous	Y30H	0.164	0.05	0.651

Modern terminal	R0	T	3394	C	ND1	Non-synonymous	Y30H	0.164	0.05	0.651
Modern terminal	R0	T	3394	C	ND1	Non-synonymous	Y30H	0.164	0.05	0.651
Modern terminal	R0	T	3394	C	ND1	Non-synonymous	Y30H	0.164	0.05	0.651
Modern terminal	R0	T	3394	C	ND1	Non-synonymous	Y30H	0.164	0.05	0.651
Modern terminal	R0	T	3394	C	ND1	Non-synonymous	Y30H	0.164	0.05	0.651
Modern terminal	R0	T	3394	C	ND1	Non-synonymous	Y30H	0.164	0.05	0.651
Pre-terminal	R0	T	3394	C	ND1	Non-synonymous	Y30H	0.164	0.05	0.651
Pre-terminal	R0	T	3394	C	ND1	Non-synonymous	Y30H	0.164	0.05	0.651
Ancient terminal	U	T	3394	C	ND1	Non-synonymous	Y30H	0.164	0.05	0.651
Modern terminal	U	T	3394	C	ND1	Non-synonymous	Y30H	0.164	0.05	0.651
Modern terminal	U	T	3394	C	ND1	Non-synonymous	Y30H	0.164	0.05	0.651
Modern terminal	U	T	3394	C	ND1	Non-synonymous	Y30H	0.164	0.05	0.651
Modern terminal	U	T	3394	C	ND1	Non-synonymous	Y30H	0.164	0.05	0.651
Modern terminal	U	T	3394	C	ND1	Non-synonymous	Y30H	0.164	0.05	0.651
Pre-terminal	U	T	3394	C	ND1	Non-synonymous	Y30H	0.164	0.05	0.651
Pre-terminal	U	T	3394	C	ND1	Non-synonymous	Y30H	0.164	0.05	0.651
Pre-terminal	U	T	3394	C	ND1	Non-synonymous	Y30H	0.164	0.05	0.651
Pre-terminal	U	T	3394	C	ND1	Non-synonymous	Y30H	0.164	0.05	0.651
Pre-terminal	U	T	3394	C	ND1	Non-synonymous	Y30H	0.164	0.05	0.651
Pre-terminal	U	T	3394	C	ND1	Non-synonymous	Y30H	0.164	0.05	0.651
Modern terminal	JT	T	3394	C	ND1	Non-synonymous	Y30H	0.164	0.05	0.651
Modern terminal	JT	T	3394	C	ND1	Non-synonymous	Y30H	0.164	0.05	0.651
Modern terminal	JT	T	3394	C	ND1	Non-synonymous	Y30H	0.164	0.05	0.651
Pre-terminal	JT	T	3394	C	ND1	Non-synonymous	Y30H	0.164	0.05	0.651
Pre-terminal	JT	T	3394	C	ND1	Non-synonymous	Y30H	0.164	0.05	0.651
Pre-terminal	JT	T	3394	C	ND1	Non-synonymous	Y30H	0.164	0.05	0.651
Pre-terminal	JT	T	3394	C	ND1	Non-synonymous	Y30H	0.164	0.05	0.651
Pre-terminal	JT	T	3394	C	ND1	Non-synonymous	Y30H	0.164	0.05	0.651
Pre-terminal	JT	T	3394	C	ND1	Non-synonymous	Y30H	0.164	0.05	0.651
Modern terminal	X	T	3394	C	ND1	Non-synonymous	Y30H	0.164	0.05	0.651
Ancient terminal	R0	A	3395	G	ND1	Non-synonymous	Y30C	0.94	0.02	0.696
Modern terminal	R0	A	3395	G	ND1	Non-synonymous	Y30C	0.94	0.02	0.696
Modern terminal	R0	A	3395	G	ND1	Non-synonymous	Y30C	0.94	0.02	0.696
Modern terminal	R0	A	3395	C	ND1	Non-synonymous	Y30C	0.94	0.02	0.696
Pre-terminal	R0	A	3395	G	ND1	Non-synonymous	Y30C	0.94	0.02	0.696
Modern terminal	U	A	3395	G	ND1	Non-synonymous	Y30C	0.94	0.02	0.696
Modern terminal	U	A	3395	G	ND1	Non-synonymous	Y30C	0.94	0.02	0.696
Modern terminal	JT	A	3395	G	ND1	Non-synonymous	Y30C	0.94	0.02	0.696
Modern terminal	JT	A	3395	G	ND1	Non-synonymous	Y30C	0.94	0.02	0.696
Modern terminal	N1	A	3395	G	ND1	Non-synonymous	Y30C	0.94	0.02	0.696
Ancient terminal	R0	A	3397	G	ND1	Non-synonymous	M31V	0.802	0.09	0.466
Modern terminal	R0	A	3397	G	ND1	Non-synonymous	M31V	0.802	0.09	0.466
Modern terminal	R0	A	3397	G	ND1	Non-synonymous	M31V	0.802	0.09	0.466
Modern terminal	R0	A	3397	G	ND1	Non-synonymous	M31V	0.802	0.09	0.466
Modern terminal	R0	A	3397	G	ND1	Non-synonymous	M31V	0.802	0.09	0.466
Modern terminal	R0	A	3397	G	ND1	Non-synonymous	M31V	0.802	0.09	0.466
Modern terminal	R0	A	3397	G	ND1	Non-synonymous	M31V	0.802	0.09	0.466
Modern terminal	R0	A	3397	G	ND1	Non-synonymous	M31V	0.802	0.09	0.466
Modern terminal	R0	A	3397	G	ND1	Non-synonymous	M31V	0.802	0.09	0.466
Pre-terminal	R0	A	3397	G	ND1	Non-synonymous	M31V	0.802	0.09	0.466

Pre-terminal	R0	A	3397	G	ND1	Non-synonymous	M31V	0.802	0.09	0.466
Pre-terminal	R0	A	3397	G	ND1	Non-synonymous	M31V	0.802	0.09	0.466
Pre-terminal	R0	A	3397	G	ND1	Non-synonymous	M31V	0.802	0.09	0.466
Pre-terminal	R0	A	3397	G	ND1	Non-synonymous	M31V	0.802	0.09	0.466
Pre-terminal	R0	A	3397	G	ND1	Non-synonymous	M31V	0.802	0.09	0.466
Ancient terminal	U	A	3397	G	ND1	Non-synonymous	M31V	0.802	0.09	0.466
Ancient terminal	U	A	3397	G	ND1	Non-synonymous	M31V	0.802	0.09	0.466
Modern terminal	U	A	3397	G	ND1	Non-synonymous	M31V	0.802	0.09	0.466
Modern terminal	U	A	3397	G	ND1	Non-synonymous	M31V	0.802	0.09	0.466
Modern terminal	U	A	3397	G	ND1	Non-synonymous	M31V	0.802	0.09	0.466
Modern terminal	U	A	3397	G	ND1	Non-synonymous	M31V	0.802	0.09	0.466
Modern terminal	U	A	3397	G	ND1	Non-synonymous	M31V	0.802	0.09	0.466
Pre-terminal	U	A	3397	G	ND1	Non-synonymous	M31V	0.802	0.09	0.466
Pre-terminal	U	A	3397	G	ND1	Non-synonymous	M31V	0.802	0.09	0.466
Pre-terminal	U	A	3397	G	ND1	Non-synonymous	M31V	0.802	0.09	0.466
Pre-terminal	U	A	3397	G	ND1	Non-synonymous	M31V	0.802	0.09	0.466
Modern terminal	JT	A	3397	G	ND1	Non-synonymous	M31V	0.802	0.09	0.466
Modern terminal	JT	A	3397	G	ND1	Non-synonymous	M31V	0.802	0.09	0.466
Pre-terminal	JT	A	3397	G	ND1	Non-synonymous	M31V	0.802	0.09	0.466
Pre-terminal	JT	A	3397	G	ND1	Non-synonymous	M31V	0.802	0.09	0.466
Ancient terminal	R0	T	3398	C	ND1	Non-synonymous	M31T	0.004	0.03	0.541
Modern terminal	R0	T	3398	C	ND1	Non-synonymous	M31T	0.004	0.03	0.541
Modern terminal	R0	T	3398	C	ND1	Non-synonymous	M31T	0.004	0.03	0.541
Modern terminal	R0	T	3398	C	ND1	Non-synonymous	M31T	0.004	0.03	0.541
Modern terminal	R0	T	3398	C	ND1	Non-synonymous	M31T	0.004	0.03	0.541
Modern terminal	R0	T	3398	C	ND1	Non-synonymous	M31T	0.004	0.03	0.541
Modern terminal	R0	T	3398	C	ND1	Non-synonymous	M31T	0.004	0.03	0.541
Modern terminal	R0	T	3398	C	ND1	Non-synonymous	M31T	0.004	0.03	0.541
Modern terminal	R0	T	3398	C	ND1	Non-synonymous	M31T	0.004	0.03	0.541
Modern terminal	R0	T	3398	C	ND1	Non-synonymous	M31T	0.004	0.03	0.541
Modern terminal	R0	T	3398	C	ND1	Non-synonymous	M31T	0.004	0.03	0.541
Modern terminal	R0	T	3398	C	ND1	Non-synonymous	M31T	0.004	0.03	0.541
Modern terminal	R0	T	3398	C	ND1	Non-synonymous	M31T	0.004	0.03	0.541
Modern terminal	R0	T	3398	C	ND1	Non-synonymous	M31T	0.004	0.03	0.541
Modern terminal	R0	T	3398	C	ND1	Non-synonymous	M31T	0.004	0.03	0.541
Modern terminal	R0	T	3398	C	ND1	Non-synonymous	M31T	0.004	0.03	0.541
Modern terminal	R0	T	3398	C	ND1	Non-synonymous	M31T	0.004	0.03	0.541
Pre-terminal	R0	T	3398	C	ND1	Non-synonymous	M31T	0.004	0.03	0.541
Pre-terminal	R0	T	3398	C	ND1	Non-synonymous	M31T	0.004	0.03	0.541
Pre-terminal	R0	T	3398	C	ND1	Non-synonymous	M31T	0.004	0.03	0.541
Pre-terminal	R0	T	3398	C	ND1	Non-synonymous	M31T	0.004	0.03	0.541
Pre-terminal	R0	T	3398	C	ND1	Non-synonymous	M31T	0.004	0.03	0.541
Pre-terminal	R0	T	3398	C	ND1	Non-synonymous	M31T	0.004	0.03	0.541
Modern terminal	U	T	3398	C	ND1	Non-synonymous	M31T	0.004	0.03	0.541
Modern terminal	U	T	3398	C	ND1	Non-synonymous	M31T	0.004	0.03	0.541
Modern terminal	U	T	3398	C	ND1	Non-synonymous	M31T	0.004	0.03	0.541
Modern terminal	U	T	3398	C	ND1	Non-synonymous	M31T	0.004	0.03	0.541
Modern terminal	U	T	3398	C	ND1	Non-synonymous	M31T	0.004	0.03	0.541
Modern terminal	U	T	3398	C	ND1	Non-synonymous	M31T	0.004	0.03	0.541
Modern terminal	U	T	3398	C	ND1	Non-synonymous	M31T	0.004	0.03	0.541

Modern terminal	U	T	3398	C	ND1	Non-synonymous	M31T	0.004	0.03	0.541
Modern terminal	U	T	3398	C	ND1	Non-synonymous	M31T	0.004	0.03	0.541
Modern terminal	U	T	3398	C	ND1	Non-synonymous	M31T	0.004	0.03	0.541
Modern terminal	U	T	3398	C	ND1	Non-synonymous	M31T	0.004	0.03	0.541
Modern terminal	U	T	3398	C	ND1	Non-synonymous	M31T	0.004	0.03	0.541
Pre-terminal	U	T	3398	C	ND1	Non-synonymous	M31T	0.004	0.03	0.541
Pre-terminal	U	T	3398	C	ND1	Non-synonymous	M31T	0.004	0.03	0.541
Modern terminal	JT	T	3398	C	ND1	Non-synonymous	M31T	0.004	0.03	0.541
Modern terminal	JT	T	3398	C	ND1	Non-synonymous	M31T	0.004	0.03	0.541
Modern terminal	JT	T	3398	C	ND1	Non-synonymous	M31T	0.004	0.03	0.541
Modern terminal	JT	T	3398	C	ND1	Non-synonymous	M31T	0.004	0.03	0.541
Modern terminal	JT	T	3398	C	ND1	Non-synonymous	M31T	0.004	0.03	0.541
Modern terminal	JT	T	3398	C	ND1	Non-synonymous	M31T	0.004	0.03	0.541
Pre-terminal	JT	T	3398	C	ND1	Non-synonymous	M31T	0.004	0.03	0.541
Pre-terminal	JT	T	3398	C	ND1	Non-synonymous	M31T	0.004	0.03	0.541
Pre-terminal	JT	T	3398	C	ND1	Non-synonymous	M31T	0.004	0.03	0.541
Modern terminal	N1	T	3398	C	ND1	Non-synonymous	M31T	0.004	0.03	0.541
Pre-terminal	N1	T	3398	C	ND1	Non-synonymous	M31T	0.004	0.03	0.541
Pre-terminal	N1	T	3398	C	ND1	Non-synonymous	M31T	0.004	0.03	0.541
Pre-terminal	N1	T	3398	C	ND1	Non-synonymous	M31T	0.004	0.03	0.541
Pre-terminal	X	T	3398	C	ND1	Non-synonymous	M31T	0.004	0.03	0.541
Ancient terminal	N2	T	3398	C	ND1	Non-synonymous	M31T	0.004	0.03	0.541
Modern terminal	N2	T	3398	C	ND1	Non-synonymous	M31T	0.004	0.03	0.541
Modern terminal	R0	A	3399	C	ND1	Non-synonymous	M31I	0.95	0.13	0.517
Modern terminal	JT	A	3399	T	ND1	Non-synonymous	M31I	0.95	0.13	0.517
Modern terminal	R0	A	3418	G	ND1	Non-synonymous	N38D	0.999	0.05	0.421
Modern terminal	R0	A	3419	G	ND1	Non-synonymous	N38S	0.999	0.11	0.294
Pre-terminal	JT	C	3420	G	ND1	Non-synonymous	N38K	1	0	0.504
Modern terminal	R0	G	3421	A	ND1	Non-synonymous	V39N	0.384	0.02	0.487
Modern terminal	R0	G	3421	A	ND1	Non-synonymous	V39N	0.384	0.02	0.487
Modern terminal	R0	G	3421	A	ND1	Non-synonymous	V39N	0.384	0.02	0.487
Modern terminal	R0	G	3421	A	ND1	Non-synonymous	V39N	0.384	0.02	0.487
Modern terminal	R0	G	3421	A	ND1	Non-synonymous	V39N	0.384	0.02	0.487
Modern terminal	R0	G	3421	A	ND1	Non-synonymous	V39N	0.384	0.02	0.487
Modern terminal	R0	G	3421	A	ND1	Non-synonymous	V39N	0.384	0.02	0.487
Pre-terminal	R0	G	3421	A	ND1	Non-synonymous	V39N	0.384	0.02	0.487
Pre-terminal	R0	G	3421	A	ND1	Non-synonymous	V39N	0.384	0.02	0.487
Pre-terminal	R0	G	3421	A	ND1	Non-synonymous	V39N	0.384	0.02	0.487
Modern terminal	U	G	3421	A	ND1	Non-synonymous	V39N	0.384	0.02	0.487
Pre-terminal	U	G	3421	A	ND1	Non-synonymous	V39N	0.384	0.02	0.487
Pre-terminal	U	G	3421	A	ND1	Non-synonymous	V39N	0.384	0.02	0.487
Pre-terminal	N2	G	3421	A	ND1	Non-synonymous	V39N	0.384	0.02	0.487
Ancient terminal	U	G	3424	C	ND1	Non-synonymous	V40L	0.015	0	0.386
Ancient terminal	U	T	3425	C	ND1	Non-synonymous	V40A	0.989	0.03	0.421
Modern terminal	R0	C	3431	T	ND1	Non-synonymous	P42L	1	0.01	0.416
Ancient terminal	R0	T	3433	A	ND1	Non-synonymous	Y43N	0.056	0.02	0.718
Modern terminal	U	T	3433	C	ND1	Non-synonymous	Y43H	0.017	0.15	0.328
Pre-terminal	U	T	3433	C	ND1	Non-synonymous	Y43H	0.017	0.15	0.328
Modern terminal	R0	A	3434	G	ND1	Non-synonymous	Y43C	0.012	0.02	0.472
Modern terminal	R0	A	3434	G	ND1	Non-synonymous	Y43C	0.012	0.02	0.472

Modern terminal	R0	A	3434	G	ND1	Non-synonymous	Y43C	0.012	0.02	0.472
Pre-terminal	R0	A	3434	G	ND1	Non-synonymous	Y43C	0.012	0.02	0.472
Pre-terminal	R0	A	3434	G	ND1	Non-synonymous	Y43C	0.012	0.02	0.472
Pre-terminal	R0	A	3434	G	ND1	Non-synonymous	Y43C	0.012	0.02	0.472
Modern terminal	U	A	3434	G	ND1	Non-synonymous	Y43C	0.012	0.02	0.472
Modern terminal	U	A	3434	G	ND1	Non-synonymous	Y43C	0.012	0.02	0.472
Pre-terminal	U	A	3434	G	ND1	Non-synonymous	Y43C	0.012	0.02	0.472
Pre-terminal	U	A	3434	G	ND1	Non-synonymous	Y43C	0.012	0.02	0.472
Pre-terminal	U	A	3434	G	ND1	Non-synonymous	Y43C	0.012	0.02	0.472
Pre-terminal	U	A	3434	G	ND1	Non-synonymous	Y43C	0.012	0.02	0.472
Pre-terminal	U	A	3434	G	ND1	Non-synonymous	Y43C	0.012	0.02	0.472
Pre-terminal	U	A	3434	G	ND1	Non-synonymous	Y43C	0.012	0.02	0.472
Modern terminal	JT	A	3434	G	ND1	Non-synonymous	Y43C	0.012	0.02	0.472
Modern terminal	JT	A	3434	G	ND1	Non-synonymous	Y43C	0.012	0.02	0.472
Pre-terminal	JT	A	3434	G	ND1	Non-synonymous	Y43C	0.012	0.02	0.472
Pre-terminal	JT	A	3434	G	ND1	Non-synonymous	Y43C	0.012	0.02	0.472
Pre-terminal	JT	A	3434	G	ND1	Non-synonymous	Y43C	0.012	0.02	0.472
Ancient terminal	JT	T	3452	C	ND1	Non-synonymous	F49S	0.998	0	0.599
Modern terminal	R0	G	3460	A	ND1	Non-synonymous	A52T	1	0	0.418
Modern terminal	R0	G	3460	A	ND1	Non-synonymous	A52T	1	0	0.418
Pre-terminal	R0	G	3460	A	ND1	Non-synonymous	A52T	1	0	0.418
Pre-terminal	R0	G	3460	A	ND1	Non-synonymous	A52T	1	0	0.418
Pre-terminal	R0	G	3460	A	ND1	Non-synonymous	A52T	1	0	0.418
Modern terminal	U	G	3460	A	ND1	Non-synonymous	A52T	1	0	0.418
Modern terminal	JT	G	3460	A	ND1	Non-synonymous	A52T	1	0	0.418
Modern terminal	JT	G	3460	A	ND1	Non-synonymous	A52T	1	0	0.418
Modern terminal	JT	G	3460	A	ND1	Non-synonymous	A52T	1	0	0.418
Modern terminal	N2	A	3463	G	ND1	Non-synonymous	M53V	0.062	1	0.141
Ancient terminal	U	T	3472	A	ND1	Non-synonymous	F56I	0.986	0.2	0.442
Modern terminal	N2	A	3475	G	ND1	Non-synonymous	T57A	0.074	0.05	0.155
Ancient terminal	JT	C	3484	T	ND1	Non-synonymous	P60S	1	0.04	0.258
Modern terminal	N1	C	3487	A	ND1	Non-synonymous	L61M	0.577	0.04	0.142
Modern terminal	R0	T	3488	C	ND1	Non-synonymous	L61P	0.999	0	0.643
Modern terminal	R0	G	3496	A	ND1	Non-synonymous	A64T	0	0.55	0.107
Modern terminal	R0	G	3496	A	ND1	Non-synonymous	A64T	0	0.55	0.107
Modern terminal	R0	G	3496	A	ND1	Non-synonymous	A64T	0	0.55	0.107
Modern terminal	JT	G	3496	A	ND1	Non-synonymous	A64T	0	0.55	0.107
Modern terminal	U	C	3497	T	ND1	Non-synonymous	A64V	0.001	0.51	0.127
Modern terminal	R0	A	3505	G	ND1	Non-synonymous	T67A	0.001	0.02	0.133
Modern terminal	R0	A	3505	T	ND1	Non-synonymous	T67S	0.002	1	0.049
Modern terminal	R0	A	3505	G	ND1	Non-synonymous	T67A	0.001	0.02	0.133
Modern terminal	R0	A	3505	G	ND1	Non-synonymous	T67A	0.001	0.02	0.133
Modern terminal	R0	A	3505	G	ND1	Non-synonymous	T67A	0.001	0.02	0.133
Modern terminal	R0	A	3505	G	ND1	Non-synonymous	T67A	0.001	0.02	0.133
Pre-terminal	R0	A	3505	G	ND1	Non-synonymous	T67A	0.001	0.02	0.133
Pre-terminal	R0	A	3505	G	ND1	Non-synonymous	T67A	0.001	0.02	0.133
Pre-terminal	R0	A	3505	G	ND1	Non-synonymous	T67A	0.001	0.02	0.133
Modern terminal	U	A	3505	G	ND1	Non-synonymous	T67A	0.001	0.02	0.133
Modern terminal	U	A	3505	G	ND1	Non-synonymous	T67A	0.001	0.02	0.133
Modern terminal	R0	A	3508	G	ND1	Non-synonymous	I68V	0.003	0.47	0.059

Pre-terminal	JT	A	3508	G	ND1	Non-synonymous	I68V	0.003	0.47	0.059
Pre-terminal	R0	T	3509	C	ND1	Non-synonymous	I68T	0.001	0.71	0.085
Modern terminal	R0	A	3511	G	ND1	Non-synonymous	T69A	0	0.51	0.129
Modern terminal	R0	A	3511	G	ND1	Non-synonymous	T69A	0	0.51	0.129
Pre-terminal	R0	A	3511	G	ND1	Non-synonymous	T69A	0	0.51	0.129
Pre-terminal	R0	A	3511	G	ND1	Non-synonymous	T69A	0	0.51	0.129
Pre-terminal	U	A	3511	G	ND1	Non-synonymous	T69A	0	0.51	0.129
Pre-terminal	U	A	3511	G	ND1	Non-synonymous	T69A	0	0.51	0.129
Modern terminal	JT	A	3511	G	ND1	Non-synonymous	T69A	0	0.51	0.129
Modern terminal	JT	A	3511	G	ND1	Non-synonymous	T69A	0	0.51	0.129
Modern terminal	JT	A	3511	G	ND1	Non-synonymous	T69A	0	0.51	0.129
Pre-terminal	JT	A	3511	G	ND1	Non-synonymous	T69A	0	0.51	0.129
Modern terminal	R0	A	3520	G	ND1	Non-synonymous	I72V	0	0.16	0.094
Modern terminal	R0	A	3520	G	ND1	Non-synonymous	I72V	0	0.16	0.094
Pre-terminal	JT	A	3520	G	ND1	Non-synonymous	I72V	0	0.16	0.094
Ancient terminal	U	C	3522	A	ND1	Non-synonymous	I72M	0.029	0.04	0.138
Ancient terminal	R0	A	3523	G	ND1	Non-synonymous	T73A	0	0.29	0.11
Modern terminal	R0	A	3523	G	ND1	Non-synonymous	T73A	0	0.29	0.11
Modern terminal	R0	A	3523	G	ND1	Non-synonymous	T73A	0	0.29	0.11
Modern terminal	R0	A	3523	G	ND1	Non-synonymous	T73A	0	0.29	0.11
Pre-terminal	R0	A	3523	G	ND1	Non-synonymous	T73A	0	0.29	0.11
Modern terminal	U	A	3523	G	ND1	Non-synonymous	T73A	0	0.29	0.11
Pre-terminal	U	A	3523	G	ND1	Non-synonymous	T73A	0	0.29	0.11
Modern terminal	JT	A	3523	G	ND1	Non-synonymous	T73A	0	0.29	0.11
Modern terminal	JT	A	3523	G	ND1	Non-synonymous	T73A	0	0.29	0.11
Pre-terminal	JT	A	3523	G	ND1	Non-synonymous	T73A	0	0.29	0.11
Modern terminal	R0	G	3526	A	ND1	Non-synonymous	A74T	0.617	0.11	0.274
Modern terminal	R0	G	3526	A	ND1	Non-synonymous	A74T	0.617	0.11	0.274
Modern terminal	U	G	3526	A	ND1	Non-synonymous	A74T	0.617	0.11	0.274
Modern terminal	X	C	3527	G	ND1	Non-synonymous	A74G	0.774	0.03	0.28
Modern terminal	U	A	3532	G	ND1	Non-synonymous	T76A	0	0.15	0.065
Modern terminal	R0	C	3533	T	ND1	Non-synonymous	T76S	0.023	0.02	0.076
Ancient terminal	U	T	3535	A	ND1	Non-synonymous	L77M	1	0.12	0.253
Pre-terminal	U	T	3535	A	ND1	Non-synonymous	L77M	1	0.12	0.253
Ancient terminal	R0	C	3541	T	ND1	Non-synonymous	L79F	0.01	0.59	0.372
Pre-terminal	R0	C	3541	T	ND1	Non-synonymous	L79F	0.01	0.59	0.372
Modern terminal	R0	A	3547	C	ND1	Non-synonymous	I81L	0	1	0.086
Modern terminal	R0	A	3547	G	ND1	Non-synonymous	I81V	0.004	0.01	0.109
Modern terminal	R0	A	3547	G	ND1	Non-synonymous	I81V	0.004	0.01	0.109
Pre-terminal	R0	A	3547	G	ND1	Non-synonymous	I81V	0.004	0.01	0.109
Modern terminal	U	A	3547	G	ND1	Non-synonymous	I81V	0.004	0.01	0.109
Modern terminal	U	A	3547	G	ND1	Non-synonymous	I81V	0.004	0.01	0.109
Modern terminal	U	A	3547	G	ND1	Non-synonymous	I81V	0.004	0.01	0.109
Modern terminal	U	A	3547	G	ND1	Non-synonymous	I81V	0.004	0.01	0.109
Pre-terminal	U	A	3547	G	ND1	Non-synonymous	I81V	0.004	0.01	0.109
Modern terminal	JT	A	3547	G	ND1	Non-synonymous	I81V	0.004	0.01	0.109
Modern terminal	JT	A	3547	G	ND1	Non-synonymous	I81V	0.004	0.01	0.109
Pre-terminal	R0	T	3548	C	ND1	Non-synonymous	I81T	0.108	0.03	0.253
Pre-terminal	R0	T	3548	C	ND1	Non-synonymous	I81T	0.108	0.03	0.253
Pre-terminal	R0	T	3548	C	ND1	Non-synonymous	I81T	0.108	0.03	0.253

Pre-terminal	R0	T	3548	C	ND1	Non-synonymous	I81T	0.108	0.03	0.253
Pre-terminal	U	T	3548	C	ND1	Non-synonymous	I81T	0.108	0.03	0.253
Modern terminal	JT	T	3548	C	ND1	Non-synonymous	I81T	0.108	0.03	0.253
Pre-terminal	R0	C	3553	T	ND1	Non-synonymous	L83F	0.999	0.02	0.291
Modern terminal	U	C	3553	T	ND1	Non-synonymous	L83F	0.999	0.02	0.291
Modern terminal	U	C	3553	T	ND1	Non-synonymous	L83F	0.999	0.02	0.291
Modern terminal	JT	C	3553	T	ND1	Non-synonymous	L83F	0.999	0.02	0.291
Modern terminal	U	G	3563	A	ND1	Nonsense				
Modern terminal	N1	G	3563	A	ND1	Nonsense				
Modern terminal	R0	A	3565	G	ND1	Non-synonymous	T87A	0.001	1	0.075
Modern terminal	R0	A	3565	G	ND1	Non-synonymous	T87A	0.001	1	0.075
Modern terminal	R0	A	3565	G	ND1	Non-synonymous	T87A	0.001	1	0.075
Pre-terminal	R0	A	3565	G	ND1	Non-synonymous	T87A	0.001	1	0.075
Pre-terminal	U	A	3565	G	ND1	Non-synonymous	T87A	0.001	1	0.075
Modern terminal	JT	A	3565	G	ND1	Non-synonymous	T87A	0.001	1	0.075
Pre-terminal	N1	A	3565	G	ND1	Non-synonymous	T87A	0.001	1	0.075
Ancient terminal	JT	C	3569	T	ND1	Non-synonymous	P88L	1	0.57	0.242
Ancient terminal	JT	C	3569	T	ND1	Non-synonymous	P88L	1	0.57	0.242
Ancient terminal	JT	C	3571	A	ND1	Non-synonymous	L89I	0.054	0.82	0.153
Modern terminal	N2	C	3571	T	ND1	Non-synonymous	L89F	0.984	0.02	0.255
Ancient terminal	R0	C	3575	A	ND1	Non-synonymous	P90H	1	0	0.357
Modern terminal	R0	A	3584	G	ND1	Non-synonymous	N93S	0.009	0.43	0.115
Ancient terminal	JT	C	3586	A	ND1	Non-synonymous	P94T	0.403	0.27	0.315
Ancient terminal	U	C	3587	G	ND1	Non-synonymous	P94S	0.184	0.55	0.23
Ancient terminal	N2	C	3589	A	ND1	Non-synonymous	L95M	1	0.19	0.208
Ancient terminal	U	T	3590	C	ND1	Non-synonymous	L95P	1	0	0.716
Modern terminal	R0	G	3592	A	ND1	Non-synonymous	V96I	0	0.94	0.032
Pre-terminal	U	G	3592	A	ND1	Non-synonymous	V96I	0	0.94	0.032
Modern terminal	JT	G	3592	A	ND1	Non-synonymous	V96I	0	0.94	0.032
Modern terminal	JT	G	3592	A	ND1	Non-synonymous	V96I	0	0.94	0.032
Pre-terminal	JT	G	3592	A	ND1	Non-synonymous	V96I	0	0.94	0.032
Modern terminal	R0	T	3593	C	ND1	Non-synonymous	V96A	0.014	0.48	0.162
Modern terminal	R0	T	3593	C	ND1	Non-synonymous	V96A	0.014	0.48	0.162
Modern terminal	R0	T	3593	C	ND1	Non-synonymous	V96A	0.014	0.48	0.162
Modern terminal	R0	T	3593	C	ND1	Non-synonymous	V96A	0.014	0.48	0.162
Modern terminal	U	T	3593	C	ND1	Non-synonymous	V96A	0.014	0.48	0.162
Pre-terminal	U	T	3593	C	ND1	Non-synonymous	V96A	0.014	0.48	0.162
Pre-terminal	U	T	3593	C	ND1	Non-synonymous	V96A	0.014	0.48	0.162
Modern terminal	JT	T	3593	C	ND1	Non-synonymous	V96A	0.014	0.48	0.162
Modern terminal	JT	G	3608	A	ND1	Non-synonymous	G101D	0.999	0	0.443
Modern terminal	R0	C	3622	A	ND1	Non-synonymous	L106M	1	0.04	0.27
Modern terminal	R0	A	3628	G	ND1	Non-synonymous	T108A	0.001	0.03	0.469
Pre-terminal	R0	A	3628	G	ND1	Non-synonymous	T108A	0.001	0.03	0.469
Modern terminal	JT	G	3635	A	ND1	Non-synonymous	S110N	0.991	0	0.493
Modern terminal	U	C	3637	A	ND1	Non-synonymous	L111M	1	0.09	0.408
Pre-terminal	R0	G	3640	A	ND1	Non-synonymous	A112T	0	0.21	0.339
Ancient terminal	R0	T	3644	C	ND1	Non-synonymous	V113A	0.71	0	0.548
Modern terminal	R0	T	3644	C	ND1	Non-synonymous	V113A	0.71	0	0.548
Pre-terminal	R0	T	3644	C	ND1	Non-synonymous	V113A	0.71	0	0.548
Ancient terminal	U	T	3644	C	ND1	Non-synonymous	V113A	0.71	0	0.548

Modern terminal	U	T	3644	C	ND1	Non-synonymous	V113A	0.71	0	0.548
Modern terminal	U	T	3644	G	ND1	Non-synonymous	V113G	1	0	0.724
Pre-terminal	U	T	3644	C	ND1	Non-synonymous	V113A	0.71	0	0.548
Modern terminal	N2	T	3644	C	ND1	Non-synonymous	V113A	0.71	0	0.548
Pre-terminal	N2	T	3644	C	ND1	Non-synonymous	V113A	0.71	0	0.548
Modern terminal	R0	A	3652	G	ND1	Non-synonymous	I116V	0.287	0.07	0.189
Ancient terminal	U	A	3652	G	ND1	Non-synonymous	I116V	0.287	0.07	0.189
Modern terminal	U	A	3652	G	ND1	Non-synonymous	I116V	0.287	0.07	0.189
Modern terminal	R0	T	3661	G	ND1	Non-synonymous	S119A	0.981	0.58	0.347
Modern terminal	U	T	3661	G	ND1	Non-synonymous	S119A	0.981	0.58	0.347
Pre-terminal	U	G	3688	C	ND1	Non-synonymous	A128P	1	0.05	0.799
Modern terminal	R0	G	3700	A	ND1	Non-synonymous	A132T	0.023	0.01	0.502
Modern terminal	R0	G	3700	A	ND1	Non-synonymous	A132T	0.023	0.01	0.502
Modern terminal	U	C	3710	T	ND1	Non-synonymous	A135V	0.999	0	0.643
Pre-terminal	JT	A	3721	G	ND1	Non-synonymous	T139A	0.861	0.03	0.62
Pre-terminal	JT	A	3721	G	ND1	Non-synonymous	T139A	0.861	0.03	0.62
Modern terminal	N2	A	3721	G	ND1	Non-synonymous	T139A	0.861	0.03	0.62
Modern terminal	R0	G	3733	A	ND1	Non-synonymous	E143K	0.879	0	0.663
Modern terminal	X	G	3733	A	ND1	Non-synonymous	E143K	0.879	0	0.663
Modern terminal	U	A	3734	G	ND1	Non-synonymous	E143G	0.368	0.01	0.65
Modern terminal	R0	G	3736	A	ND1	Non-synonymous	V144I	0.001	0.08	0.201
Modern terminal	R0	G	3736	A	ND1	Non-synonymous	V144I	0.001	0.08	0.201
Pre-terminal	R0	G	3736	A	ND1	Non-synonymous	V144I	0.001	0.08	0.201
Modern terminal	U	G	3736	A	ND1	Non-synonymous	V144I	0.001	0.08	0.201
Modern terminal	U	G	3736	A	ND1	Non-synonymous	V144I	0.001	0.08	0.201
Modern terminal	U	G	3736	A	ND1	Non-synonymous	V144I	0.001	0.08	0.201
Modern terminal	U	G	3736	A	ND1	Non-synonymous	V144I	0.001	0.08	0.201
Pre-terminal	U	G	3736	A	ND1	Non-synonymous	V144I	0.001	0.08	0.201
Pre-terminal	U	G	3736	A	ND1	Non-synonymous	V144I	0.001	0.08	0.201
Ancient terminal	JT	G	3736	A	ND1	Non-synonymous	V144I	0.001	0.08	0.201
Modern terminal	JT	G	3736	A	ND1	Non-synonymous	V144I	0.001	0.08	0.201
Pre-terminal	JT	G	3736	A	ND1	Non-synonymous	V144I	0.001	0.08	0.201
Pre-terminal	N1	G	3736	A	ND1	Non-synonymous	V144I	0.001	0.08	0.201
Modern terminal	R0	G	3745	A	ND1	Non-synonymous	A147T	0.006	0.03	0.354
Modern terminal	R0	G	3745	A	ND1	Non-synonymous	A147T	0.006	0.03	0.354
Modern terminal	R0	G	3745	A	ND1	Non-synonymous	A147T	0.006	0.03	0.354
Modern terminal	R0	G	3745	A	ND1	Non-synonymous	A147T	0.006	0.03	0.354
Modern terminal	R0	G	3745	A	ND1	Non-synonymous	A147T	0.006	0.03	0.354
Pre-terminal	R0	G	3745	A	ND1	Non-synonymous	A147T	0.006	0.03	0.354
Pre-terminal	R0	G	3745	A	ND1	Non-synonymous	A147T	0.006	0.03	0.354
Pre-terminal	R0	G	3745	A	ND1	Non-synonymous	A147T	0.006	0.03	0.354
Pre-terminal	R0	G	3745	A	ND1	Non-synonymous	A147T	0.006	0.03	0.354
Modern terminal	U	G	3745	A	ND1	Non-synonymous	A147T	0.006	0.03	0.354
Modern terminal	U	G	3745	A	ND1	Non-synonymous	A147T	0.006	0.03	0.354
Modern terminal	U	G	3745	A	ND1	Non-synonymous	A147T	0.006	0.03	0.354
Modern terminal	U	G	3745	A	ND1	Non-synonymous	A147T	0.006	0.03	0.354
Modern terminal	U	G	3745	A	ND1	Non-synonymous	A147T	0.006	0.03	0.354
Modern terminal	U	G	3745	A	ND1	Non-synonymous	A147T	0.006	0.03	0.354
Pre-terminal	U	G	3745	A	ND1	Non-synonymous	A147T	0.006	0.03	0.354
Modern terminal	JT	G	3745	A	ND1	Non-synonymous	A147T	0.006	0.03	0.354

Modern terminal	JT	G	3745	A	ND1	Non-synonymous	A147T	0.006	0.03	0.354
Pre-terminal	X	G	3745	A	ND1	Non-synonymous	A147T	0.006	0.03	0.354
Modern terminal	R0	C	3746	T	ND1	Non-synonymous	A147V	0.029	0	0.395
Modern terminal	R0	C	3746	T	ND1	Non-synonymous	A147V	0.029	0	0.395
Pre-terminal	R0	C	3746	T	ND1	Non-synonymous	A147V	0.029	0	0.395
Modern terminal	U	C	3746	T	ND1	Non-synonymous	A147V	0.029	0	0.395
Modern terminal	U	C	3746	T	ND1	Non-synonymous	A147V	0.029	0	0.395
Modern terminal	JT	C	3746	T	ND1	Non-synonymous	A147V	0.029	0	0.395
Modern terminal	N1	C	3746	T	ND1	Non-synonymous	A147V	0.029	0	0.395
Pre-terminal	X	C	3746	T	ND1	Non-synonymous	A147V	0.029	0	0.395
Modern terminal	U	T	3760	G	ND1	Non-synonymous	S152A	0.002	0.15	0.275
Pre-terminal	U	T	3760	G	ND1	Non-synonymous	S152A	0.002	0.15	0.275
Modern terminal	R0	C	3764	T	ND1	Non-synonymous	T153M	0.539	0.12	0.125
Ancient terminal	JT	C	3764	T	ND1	Non-synonymous	T153M	0.539	0.12	0.125
Modern terminal	R0	G	3776	A	ND1	Non-synonymous	S157N	0	0.67	0.062
Modern terminal	R0	T	3784	C	ND1	Non-synonymous	F160L	0.992	0.56	0.323
Ancient terminal	X	A	3787	G	ND1	Non-synonymous	N161D	0.854	0.12	0.134
Modern terminal	R0	A	3788	G	ND1	Non-synonymous	N161S	0.074	0.26	0.082
Modern terminal	U	A	3788	G	ND1	Non-synonymous	N161S	0.074	0.26	0.082
Modern terminal	R0	A	3796	G	ND1	Non-synonymous	T164A	0.001	0.64	0.225
Pre-terminal	R0	A	3796	G	ND1	Non-synonymous	T164A	0.001	0.64	0.225
Modern terminal	U	A	3796	G	ND1	Non-synonymous	T164A	0.001	0.64	0.225
Modern terminal	U	A	3796	G	ND1	Non-synonymous	T164A	0.001	0.64	0.225
Modern terminal	U	A	3796	G	ND1	Non-synonymous	T164A	0.001	0.64	0.225
Pre-terminal	U	A	3796	G	ND1	Non-synonymous	T164A	0.001	0.64	0.225
Pre-terminal	U	A	3796	G	ND1	Non-synonymous	T164A	0.001	0.64	0.225
Pre-terminal	U	A	3796	G	ND1	Non-synonymous	T164A	0.001	0.64	0.225
Modern terminal	JT	A	3796	G	ND1	Non-synonymous	T164A	0.001	0.64	0.225
Pre-terminal	JT	A	3796	G	ND1	Non-synonymous	T164A	0.001	0.64	0.225
Pre-terminal	JT	A	3796	G	ND1	Non-synonymous	T164A	0.001	0.64	0.225
Ancient terminal	U	T	3800	A	ND1	Non-synonymous	L165H	1	0	0.658
Modern terminal	R0	T	3803	C	ND1	Non-synonymous	I166T	0.998	0.36	0.238
Modern terminal	R0	T	3803	C	ND1	Non-synonymous	I166T	0.998	0.36	0.238
Ancient terminal	U	T	3803	A	ND1	Non-synonymous	I166T	0.998	0.36	0.238
Pre-terminal	X	T	3803	G	ND1	Non-synonymous	I166T	0.998	0.36	0.238
Pre-terminal	R0	C	3806	T	ND1	Non-synonymous	T167M	0.282	0.17	0.173
Modern terminal	R0	A	3808	G	ND1	Non-synonymous	T168A	0.009	0.67	0.235
Modern terminal	R0	A	3808	G	ND1	Non-synonymous	T168A	0.009	0.67	0.235
Pre-terminal	R0	A	3808	G	ND1	Non-synonymous	T168A	0.009	0.67	0.235
Modern terminal	U	A	3808	G	ND1	Non-synonymous	T168A	0.009	0.67	0.235
Modern terminal	U	A	3808	G	ND1	Non-synonymous	T168A	0.009	0.67	0.235
Pre-terminal	JT	C	3820	T	ND1	Non-synonymous	L172F	0.001	0.06	0.222
Pre-terminal	R0	C	3832	A	ND1	Non-synonymous	L176M	0.989	0.06	0.213
Pre-terminal	JT	C	3832	A	ND1	Non-synonymous	L176M	0.989	0.06	0.213
Modern terminal	R0	T	3838	A	ND1	Non-synonymous	S178T	0	0.9	0.214
Modern terminal	R0	C	3839	A	ND1	Nonsense				
Modern terminal	JT	C	3839	T	ND1	Non-synonymous	S178L	0.371	0.51	0.378
Ancient terminal	U	G	3842	T	ND1	Non-synonymous	W179L	1	0.35	0.779
Modern terminal	JT	C	3845	T	ND1	Non-synonymous	P180L	1	0	0.623
Modern terminal	R0	A	3856	G	ND1	Non-synonymous	M184V	1	0.23	0.663

Modern terminal	R0	A	3865	G	ND1	Non-synonymous	I187V	0.002	0.13	0.172
Pre-terminal	R0	A	3865	G	ND1	Non-synonymous	I187V	0.002	0.13	0.172
Modern terminal	U	A	3865	G	ND1	Non-synonymous	I187V	0.002	0.13	0.172
Pre-terminal	U	A	3865	G	ND1	Non-synonymous	I187V	0.002	0.13	0.172
Modern terminal	R0	T	3866	C	ND1	Non-synonymous	I187T	0.076	0.08	0.345
Modern terminal	R0	T	3866	C	ND1	Non-synonymous	I187T	0.076	0.08	0.345
Modern terminal	R0	T	3866	C	ND1	Non-synonymous	I187T	0.076	0.08	0.345
Modern terminal	R0	T	3866	C	ND1	Non-synonymous	I187T	0.076	0.08	0.345
Modern terminal	R0	T	3866	C	ND1	Non-synonymous	I187T	0.076	0.08	0.345
Modern terminal	U	T	3866	C	ND1	Non-synonymous	I187T	0.076	0.08	0.345
Modern terminal	U	T	3866	C	ND1	Non-synonymous	I187T	0.076	0.08	0.345
Modern terminal	U	T	3866	C	ND1	Non-synonymous	I187T	0.076	0.08	0.345
Modern terminal	U	T	3866	C	ND1	Non-synonymous	I187T	0.076	0.08	0.345
Modern terminal	U	T	3866	C	ND1	Non-synonymous	I187T	0.076	0.08	0.345
Modern terminal	U	T	3866	G	ND1	Non-synonymous	I187T	0.076	0.08	0.345
Pre-terminal	U	T	3866	C	ND1	Non-synonymous	I187T	0.076	0.08	0.345
Modern terminal	JT	T	3866	C	ND1	Non-synonymous	I187T	0.076	0.08	0.345
Modern terminal	JT	T	3866	C	ND1	Non-synonymous	I187T	0.076	0.08	0.345
Pre-terminal	JT	T	3866	C	ND1	Non-synonymous	I187T	0.076	0.08	0.345
Pre-terminal	JT	T	3866	C	ND1	Non-synonymous	I187T	0.076	0.08	0.345
Pre-terminal	JT	T	3866	C	ND1	Non-synonymous	I187T	0.076	0.08	0.345
Modern terminal	N1	T	3866	C	ND1	Non-synonymous	I187T	0.076	0.08	0.345
Modern terminal	X	T	3866	C	ND1	Non-synonymous	I187T	0.076	0.08	0.345
Pre-terminal	N2	T	3866	C	ND1	Non-synonymous	I187T	0.076	0.08	0.345
Ancient terminal	R0	G	3880	T	ND1	Nonsense				
Modern terminal	R0	G	3890	A	ND1	Non-synonymous	R195Q	1	0	0.421
Modern terminal	R0	A	3892	G	ND1	Non-synonymous	T196A	0.001	1	0.035
Modern terminal	R0	A	3892	G	ND1	Non-synonymous	T196A	0.001	1	0.035
Modern terminal	R0	A	3892	G	ND1	Non-synonymous	T196A	0.001	1	0.035
Modern terminal	R0	A	3892	G	ND1	Non-synonymous	T196A	0.001	1	0.035
Pre-terminal	R0	A	3892	G	ND1	Non-synonymous	T196A	0.001	1	0.035
Pre-terminal	R0	A	3892	G	ND1	Non-synonymous	T196A	0.001	1	0.035
Ancient terminal	U	A	3892	G	ND1	Non-synonymous	T196A	0.001	1	0.035
Modern terminal	U	A	3892	G	ND1	Non-synonymous	T196A	0.001	1	0.035
Modern terminal	U	A	3892	G	ND1	Non-synonymous	T196A	0.001	1	0.035
Modern terminal	U	A	3892	T	ND1	Non-synonymous	T196S	0.222	0.17	0.126
Pre-terminal	U	A	3892	G	ND1	Non-synonymous	T196A	0.001	1	0.035
Modern terminal	JT	A	3892	G	ND1	Non-synonymous	T196A	0.001	1	0.035
Modern terminal	JT	A	3892	G	ND1	Non-synonymous	T196A	0.001	1	0.035
Modern terminal	JT	G	3907	A	ND1	Non-synonymous	A201T	0	1	0.029
Ancient terminal	U	C	3908	A	ND1	Non-synonymous	A201D	0	0.02	0.226
Ancient terminal	R0	G	3913	T	ND1	Non-synonymous	G203W	1	0	0.68
Modern terminal	U	G	3914	A	ND1	Non-synonymous	G203E	1	0	0.643
Ancient terminal	JT	G	3914	T	ND1	Non-synonymous	G203V	1	0	0.589
Ancient terminal	JT	G	3914	T	ND1	Non-synonymous	G203V	1	0	0.589
Ancient terminal	X	G	3928	A	ND1	Non-synonymous	V208I	0.001	0.01	0.144
Modern terminal	U	G	3935	A	ND1	Non-synonymous	G210D	1	0	0.783
Modern terminal	R0	A	3943	G	ND1	Non-synonymous	I213M	0.658	0	0.404
Pre-terminal	R0	A	3943	G	ND1	Non-synonymous	I213M	0.658	0	0.404
Modern terminal	U	A	3943	G	ND1	Non-synonymous	I213M	0.658	0	0.404

Pre-terminal	U	A	3943	G	ND1	Non-synonymous	I213M	0.658	0	0.404
Pre-terminal	U	A	3943	G	ND1	Non-synonymous	I213M	0.658	0	0.404
Modern terminal	R0	T	3944	C	ND1	Non-synonymous	I213T	0.002	0.04	0.48
Modern terminal	R0	T	3944	C	ND1	Non-synonymous	I213T	0.002	0.04	0.48
Modern terminal	R0	G	3952	T	ND1	Non-synonymous	A216S	0.007	0.76	0.424
Modern terminal	JT	G	3952	A	ND1	Non-synonymous	A216S	0.007	0.76	0.424
Modern terminal	R0	A	3991	G	ND1	Non-synonymous	T229A	0.014	1	0.118
Modern terminal	R0	C	3992	T	ND1	Non-synonymous	T229M	0	0.05	0.202
Pre-terminal	R0	C	3992	T	ND1	Non-synonymous	T229M	0	0.05	0.202
Modern terminal	U	C	3992	T	ND1	Non-synonymous	T229M	0	0.05	0.202
Pre-terminal	U	C	3992	T	ND1	Non-synonymous	T229M	0	0.05	0.202
Pre-terminal	U	C	3992	T	ND1	Non-synonymous	T229M	0	0.05	0.202
Modern terminal	JT	C	3992	T	ND1	Non-synonymous	T229M	0	0.05	0.202
Modern terminal	JT	C	3992	T	ND1	Non-synonymous	T229M	0	0.05	0.202
Pre-terminal	JT	C	3992	T	ND1	Non-synonymous	T229M	0	0.05	0.202
Pre-terminal	JT	C	3992	T	ND1	Non-synonymous	T229M	0	0.05	0.202
Modern terminal	R0	A	3995	G	ND1	Non-synonymous	N230S	0.487	0.02	0.476
Pre-terminal	U	A	3995	C	ND1	Non-synonymous	N230T	0.791	0	0.545
Pre-terminal	U	A	3995	C	ND1	Non-synonymous	N230T	0.791	0	0.545
Ancient terminal	JT	C	3996	A	ND1	Non-synonymous	N230K	0.984	0	0.669
Ancient terminal	JT	A	3997	G	ND1	Non-synonymous	I231V	0.993	0.01	0.3
Pre-terminal	U	A	4000	C	ND1	Non-synonymous	I232L	0.039	0.28	0.456
Modern terminal	R0	T	4002	C	ND1	Non-synonymous	I232L	0.039	0.28	0.456
Modern terminal	R0	T	4002	C	ND1	Non-synonymous	I232L	0.039	0.28	0.456
Modern terminal	R0	T	4002	C	ND1	Non-synonymous	I232L	0.039	0.28	0.456
Pre-terminal	R0	T	4002	C	ND1	Non-synonymous	I232L	0.039	0.28	0.456
Modern terminal	U	T	4002	C	ND1	Non-synonymous	I232L	0.039	0.28	0.456
Pre-terminal	U	T	4002	C	ND1	Non-synonymous	I232L	0.039	0.28	0.456
Modern terminal	JT	T	4002	C	ND1	Non-synonymous	I232L	0.039	0.28	0.456
Modern terminal	JT	T	4002	C	ND1	Non-synonymous	I232L	0.039	0.28	0.456
Modern terminal	R0	A	4012	G	ND1	Non-synonymous	T236A	0	1	0.074
Modern terminal	R0	A	4012	G	ND1	Non-synonymous	T236A	0	1	0.074
Pre-terminal	R0	A	4012	G	ND1	Non-synonymous	T236A	0	1	0.074
Pre-terminal	R0	A	4012	G	ND1	Non-synonymous	T236A	0	1	0.074
Ancient terminal	U	A	4012	G	ND1	Non-synonymous	T236A	0	1	0.074
Modern terminal	JT	A	4012	G	ND1	Non-synonymous	T236A	0	1	0.074
Modern terminal	JT	A	4012	G	ND1	Non-synonymous	T236A	0	1	0.074
Pre-terminal	JT	A	4012	G	ND1	Non-synonymous	T236A	0	1	0.074
Pre-terminal	JT	C	4013	T	ND1	Non-synonymous	T236I	0.064	0.16	0.161
Modern terminal	U	C	4015	T	ND1	Non-synonymous	L237F	1	0.12	0.26
Modern terminal	R0	A	4021	G	ND1	Non-synonymous	T239A	0	1	0.374
Pre-terminal	R0	A	4021	T	ND1	Non-synonymous	T239S	0.001	0.16	0.302
Pre-terminal	U	A	4021	G	ND1	Non-synonymous	T239A	0	1	0.374
Pre-terminal	U	A	4021	G	ND1	Non-synonymous	T239A	0	1	0.374
Modern terminal	JT	A	4021	G	ND1	Non-synonymous	T239A	0	1	0.374
Modern terminal	N1	A	4021	G	ND1	Non-synonymous	T239A	0	1	0.374
Pre-terminal	N1	A	4021	G	ND1	Non-synonymous	T239A	0	1	0.374
Ancient terminal	R0	A	4024	G	ND1	Non-synonymous	T240A	0.002	0.03	0.228
Modern terminal	U	A	4024	T	ND1	Non-synonymous	T240S	0.271	0.05	0.156
Modern terminal	JT	A	4024	T	ND1	Non-synonymous	T240S	0.271	0.05	0.156

Ancient terminal	R0	C	4025	T	ND1	Non-synonymous	T240M	0.023	0.08	0.234
Modern terminal	R0	C	4025	T	ND1	Non-synonymous	T240M	0.023	0.08	0.234
Modern terminal	R0	C	4025	T	ND1	Non-synonymous	T240M	0.023	0.08	0.234
Modern terminal	R0	C	4025	T	ND1	Non-synonymous	T240M	0.023	0.08	0.234
Modern terminal	R0	C	4025	T	ND1	Non-synonymous	T240M	0.023	0.08	0.234
Modern terminal	R0	C	4025	T	ND1	Non-synonymous	T240M	0.023	0.08	0.234
Modern terminal	R0	C	4025	T	ND1	Non-synonymous	T240M	0.023	0.08	0.234
Pre-terminal	R0	C	4025	T	ND1	Non-synonymous	T240M	0.023	0.08	0.234
Pre-terminal	R0	C	4025	T	ND1	Non-synonymous	T240M	0.023	0.08	0.234
Ancient terminal	U	C	4025	T	ND1	Non-synonymous	T240M	0.023	0.08	0.234
Modern terminal	U	C	4025	T	ND1	Non-synonymous	T240M	0.023	0.08	0.234
Pre-terminal	U	C	4025	T	ND1	Non-synonymous	T240M	0.023	0.08	0.234
Pre-terminal	U	C	4025	T	ND1	Non-synonymous	T240M	0.023	0.08	0.234
Modern terminal	JT	C	4025	T	ND1	Non-synonymous	T240M	0.023	0.08	0.234
Modern terminal	JT	C	4025	T	ND1	Non-synonymous	T240M	0.023	0.08	0.234
Modern terminal	JT	C	4025	T	ND1	Non-synonymous	T240M	0.023	0.08	0.234
Pre-terminal	JT	C	4025	T	ND1	Non-synonymous	T240M	0.023	0.08	0.234
Pre-terminal	JT	C	4025	T	ND1	Non-synonymous	T240M	0.023	0.08	0.234
Modern terminal	N1	C	4025	T	ND1	Non-synonymous	T240M	0.023	0.08	0.234
Modern terminal	N1	C	4025	T	ND1	Non-synonymous	T240M	0.023	0.08	0.234
Pre-terminal	N1	C	4025	T	ND1	Non-synonymous	T240M	0.023	0.08	0.234
Modern terminal	N2	C	4025	T	ND1	Non-synonymous	T240M	0.023	0.08	0.234
Modern terminal	U	C	4029	A	ND1	Non-synonymous	I241M	0.998	0.1	0.259
Modern terminal	U	C	4029	A	ND1	Non-synonymous	I241M	0.998	0.1	0.259
Ancient terminal	R0	T	4030	A	ND1	Non-synonymous	F242I	1	0	0.561
Modern terminal	N1	C	4033	A	ND1	Non-synonymous	L243M	0.745	0.08	0.203
Ancient terminal	JT	G	4037	T	ND1	Non-synonymous	G244V	0.971	0	0.648
Modern terminal	R0	G	4048	A	ND1	Non-synonymous	D248N	0	0.48	0.074
Modern terminal	R0	G	4048	A	ND1	Non-synonymous	D248N	0	0.48	0.074
Modern terminal	R0	G	4048	A	ND1	Non-synonymous	D248N	0	0.48	0.074
Modern terminal	R0	G	4048	A	ND1	Non-synonymous	D248N	0	0.48	0.074
Modern terminal	R0	G	4048	A	ND1	Non-synonymous	D248N	0	0.48	0.074
Modern terminal	R0	G	4048	A	ND1	Non-synonymous	D248N	0	0.48	0.074
Pre-terminal	R0	G	4048	A	ND1	Non-synonymous	D248N	0	0.48	0.074
Modern terminal	U	G	4048	A	ND1	Non-synonymous	D248N	0	0.48	0.074
Modern terminal	U	G	4048	A	ND1	Non-synonymous	D248N	0	0.48	0.074
Modern terminal	U	G	4048	A	ND1	Non-synonymous	D248N	0	0.48	0.074
Modern terminal	U	G	4048	A	ND1	Non-synonymous	D248N	0	0.48	0.074
Modern terminal	U	G	4048	A	ND1	Non-synonymous	D248N	0	0.48	0.074
Pre-terminal	U	G	4048	A	ND1	Non-synonymous	D248N	0	0.48	0.074
Modern terminal	JT	G	4048	A	ND1	Non-synonymous	D248N	0	0.48	0.074
Modern terminal	JT	G	4048	A	ND1	Non-synonymous	D248N	0	0.48	0.074
Modern terminal	N1	G	4048	A	ND1	Non-synonymous	D248N	0	0.48	0.074
Modern terminal	N1	G	4048	A	ND1	Non-synonymous	D248N	0	0.48	0.074
Modern terminal	JT	G	4051	A	ND1	Non-synonymous	A249T	0	0.22	0.067
Modern terminal	R0	C	4052	T	ND1	Non-synonymous	A249V	0	0.19	0.08
Modern terminal	U	C	4058	T	ND1	Non-synonymous	S251F	0	0.71	0.196
Modern terminal	U	C	4076	T	ND1	Non-synonymous	T257M	0.007	0.08	0.191
Pre-terminal	JT	T	4081	C	ND1	Non-synonymous	F259L	0.13	0.41	0.248
Modern terminal	R0	G	4084	A	ND1	Non-synonymous	V260I	0	0.87	0.058

Modern terminal	R0	G	4084	A	ND1	Non-synonymous	V260I	0	0.87	0.058
Pre-terminal	R0	G	4084	A	ND1	Non-synonymous	V260I	0	0.87	0.058
Pre-terminal	R0	G	4084	A	ND1	Non-synonymous	V260I	0	0.87	0.058
Pre-terminal	R0	G	4084	A	ND1	Non-synonymous	V260I	0	0.87	0.058
Pre-terminal	JT	G	4084	A	ND1	Non-synonymous	V260I	0	0.87	0.058
Ancient terminal	R0	C	4088	T	ND1	Non-synonymous	T261I	0	0.67	0.123
Ancient terminal	R0	A	4091	G	ND1	Nonsense				
Modern terminal	R0	A	4093	G	ND1	Non-synonymous	T263A	0	0.38	0.223
Pre-terminal	R0	A	4093	G	ND1	Non-synonymous	T263A	0	0.38	0.223
Pre-terminal	U	A	4093	G	ND1	Non-synonymous	T263A	0	0.38	0.223
Modern terminal	N1	A	4093	G	ND1	Non-synonymous	T263A	0	0.38	0.223
Pre-terminal	X	A	4093	G	ND1	Non-synonymous	T263A	0	0.38	0.223
Pre-terminal	N2	A	4093	G	ND1	Non-synonymous	T263A	0	0.38	0.223
Modern terminal	R0	T	4100	C	ND1	Non-synonymous	L265P	0.999	0.04	0.755
Ancient terminal	R0	C	4102	A	ND1	Non-synonymous	L266M	1	0.01	0.294
Modern terminal	R0	C	4102	A	ND1	Non-synonymous	L266M	1	0.01	0.294
Modern terminal	R0	A	4105	G	ND1	Non-synonymous	T267A	0.341	0.46	0.314
Pre-terminal	R0	C	4106	T	ND1	Non-synonymous	T267I	0.018	0.49	0.362
Modern terminal	R0	C	4109	T	ND1	Non-synonymous	S268F	0.831	1	0.287
Modern terminal	N2	T	4118	G	ND1	Non-synonymous	L271W	1	0	0.715
Modern terminal	R0	A	4123	G	ND1	Non-synonymous	I273V	0	0.84	0.111
Modern terminal	U	A	4123	G	ND1	Non-synonymous	I273V	0	0.84	0.111
Modern terminal	R0	A	4129	G	ND1	Non-synonymous	T275A	0	1	0.037
Modern terminal	R0	A	4129	G	ND1	Non-synonymous	T275A	0	1	0.037
Modern terminal	U	A	4129	G	ND1	Non-synonymous	T275A	0	1	0.037
Modern terminal	U	A	4129	G	ND1	Non-synonymous	T275A	0	1	0.037
Modern terminal	U	A	4129	G	ND1	Non-synonymous	T275A	0	1	0.037
Pre-terminal	U	A	4129	T	ND1	Non-synonymous	T275S	0.001	0.04	0.107
Pre-terminal	U	A	4129	G	ND1	Non-synonymous	T275A	0	1	0.037
Pre-terminal	U	A	4129	G	ND1	Non-synonymous	T275A	0	1	0.037
Modern terminal	R0	C	4130	T	ND1	Non-synonymous	T275M	0.934	0	0.084
Pre-terminal	U	G	4132	A	ND1	Non-synonymous	A276T	0	0.49	0.071
Modern terminal	R0	T	4135	C	ND1	Non-synonymous	Y277H	0.999	0	0.393
Modern terminal	R0	T	4135	C	ND1	Non-synonymous	Y277H	0.999	0	0.393
Modern terminal	R0	T	4135	C	ND1	Non-synonymous	Y277H	0.999	0	0.393
Modern terminal	R0	T	4135	C	ND1	Non-synonymous	Y277H	0.999	0	0.393
Modern terminal	R0	T	4135	C	ND1	Non-synonymous	Y277H	0.999	0	0.393
Pre-terminal	R0	T	4135	C	ND1	Non-synonymous	Y277H	0.999	0	0.393
Modern terminal	U	T	4135	C	ND1	Non-synonymous	Y277H	0.999	0	0.393
Modern terminal	U	T	4135	C	ND1	Non-synonymous	Y277H	0.999	0	0.393
Modern terminal	X	T	4135	C	ND1	Non-synonymous	Y277H	0.999	0	0.393
Modern terminal	R0	A	4136	G	ND1	Non-synonymous	Y277C	1	0.01	0.427
Modern terminal	R0	A	4136	G	ND1	Non-synonymous	Y277C	1	0.01	0.427
Modern terminal	R0	A	4136	G	ND1	Non-synonymous	Y277C	1	0.01	0.427
Modern terminal	R0	A	4136	G	ND1	Non-synonymous	Y277C	1	0.01	0.427
Modern terminal	R0	A	4136	G	ND1	Non-synonymous	Y277C	1	0.01	0.427
Modern terminal	R0	A	4136	G	ND1	Non-synonymous	Y277C	1	0.01	0.427
Modern terminal	R0	A	4136	G	ND1	Non-synonymous	Y277C	1	0.01	0.427
Pre-terminal	R0	A	4136	G	ND1	Non-synonymous	Y277C	1	0.01	0.427
Ancient terminal	U	A	4136	G	ND1	Non-synonymous	Y277C	1	0.01	0.427

Modern terminal	U	A	4136	G	ND1	Non-synonymous	Y277C	1	0.01	0.427
Modern terminal	JT	A	4136	G	ND1	Non-synonymous	Y277C	1	0.01	0.427
Modern terminal	JT	A	4136	G	ND1	Non-synonymous	Y277C	1	0.01	0.427
Modern terminal	JT	A	4136	G	ND1	Non-synonymous	Y277C	1	0.01	0.427
Modern terminal	JT	A	4136	G	ND1	Non-synonymous	Y277C	1	0.01	0.427
Modern terminal	N1	A	4136	G	ND1	Non-synonymous	Y277C	1	0.01	0.427
Modern terminal	X	C	4138	T	ND1	Non-synonymous	P278S	1	0	0.395
Ancient terminal	U	A	4151	T	ND1	Non-synonymous	Y282F	0.999	0	0.316
Modern terminal	N1	T	4160	C	ND1	Non-synonymous	L285P	1	0	0.833
Modern terminal	R0	A	4166	G	ND1	Non-synonymous	H287R	0.084	0.47	0.376
Modern terminal	R0	C	4171	A	ND1	Non-synonymous	L289M	1	0.21	0.285
Pre-terminal	U	T	4172	A	ND1	Non-synonymous	L289Q	1	0.03	0.683
Modern terminal	U	C	4192	A	ND1	Non-synonymous	L296I	0.962	0.32	0.19
Pre-terminal	U	A	4206	T	ND1	Non-synonymous	L300F	0	0.16	0.262
Modern terminal	R0	T	4208	G	ND1	Non-synonymous	L301R	0.967	0	0.813
Modern terminal	R0	T	4216	C	ND1	Non-synonymous	Y304H	0	1	0.094
Modern terminal	R0	T	4216	C	ND1	Non-synonymous	Y304H	0	1	0.094
Modern terminal	R0	T	4216	C	ND1	Non-synonymous	Y304H	0	1	0.094
Modern terminal	R0	T	4216	C	ND1	Non-synonymous	Y304H	0	1	0.094
Pre-terminal	R0	T	4216	C	ND1	Non-synonymous	Y304H	0	1	0.094
Pre-terminal	R0	T	4216	C	ND1	Non-synonymous	Y304H	0	1	0.094
Pre-terminal	R0	T	4216	C	ND1	Non-synonymous	Y304H	0	1	0.094
Pre-terminal	R0	T	4216	C	ND1	Non-synonymous	Y304H	0	1	0.094
Pre-terminal	R0	T	4216	C	ND1	Non-synonymous	Y304H	0	1	0.094
Pre-terminal	R0	T	4216	C	ND1	Non-synonymous	Y304H	0	1	0.094
Ancient terminal	U	T	4216	C	ND1	Non-synonymous	Y304H	0	1	0.094
Ancient terminal	U	T	4216	C	ND1	Non-synonymous	Y304H	0	1	0.094
Ancient terminal	U	T	4216	C	ND1	Non-synonymous	Y304H	0	1	0.094
Ancient terminal	U	T	4216	C	ND1	Non-synonymous	Y304H	0	1	0.094
Modern terminal	U	T	4216	C	ND1	Non-synonymous	Y304H	0	1	0.094
Modern terminal	U	T	4216	C	ND1	Non-synonymous	Y304H	0	1	0.094
Modern terminal	U	T	4216	C	ND1	Non-synonymous	Y304H	0	1	0.094
Modern terminal	U	T	4216	C	ND1	Non-synonymous	Y304H	0	1	0.094
Pre-terminal	U	T	4216	C	ND1	Non-synonymous	Y304H	0	1	0.094
Pre-terminal	U	T	4216	C	ND1	Non-synonymous	Y304H	0	1	0.094
Modern terminal	N1	T	4216	C	ND1	Non-synonymous	Y304H	0	1	0.094
Pre-terminal	X	T	4216	C	ND1	Non-synonymous	Y304H	0	1	0.094
Modern terminal	N2	T	4216	C	ND1	Non-synonymous	Y304H	0	1	0.094
Modern terminal	R0	G	4219	A	ND1	Non-synonymous	V305I	0	1	0.046
Modern terminal	R0	G	4219	A	ND1	Non-synonymous	V305I	0	1	0.046
Pre-terminal	R0	G	4219	A	ND1	Non-synonymous	V305I	0	1	0.046
Modern terminal	U	G	4219	A	ND1	Non-synonymous	V305I	0	1	0.046
Modern terminal	U	G	4219	A	ND1	Non-synonymous	V305I	0	1	0.046
Modern terminal	JT	G	4219	A	ND1	Non-synonymous	V305I	0	1	0.046
Pre-terminal	JT	G	4219	A	ND1	Non-synonymous	V305I	0	1	0.046
Pre-terminal	R0	A	4225	G	ND1	Non-synonymous	M307V	0.003	0.21	0.131
Modern terminal	U	A	4225	G	ND1	Non-synonymous	M307V	0.003	0.21	0.131
Pre-terminal	U	A	4225	G	ND1	Non-synonymous	M307V	0.003	0.21	0.131
Modern terminal	JT	A	4225	G	ND1	Non-synonymous	M307V	0.003	0.21	0.131
Modern terminal	JT	A	4225	G	ND1	Non-synonymous	M307V	0.003	0.21	0.131

Modern terminal	JT	A	4225	G	ND1	Non-synonymous	M307V	0.003	0.21	0.131
Pre-terminal	JT	A	4225	G	ND1	Non-synonymous	M307V	0.003	0.21	0.131
Pre-terminal	JT	A	4225	G	ND1	Non-synonymous	M307V	0.003	0.21	0.131
Pre-terminal	JT	A	4225	G	ND1	Non-synonymous	M307V	0.003	0.21	0.131
Modern terminal	N1	A	4225	G	ND1	Non-synonymous	M307V	0.003	0.21	0.131
Modern terminal	R0	T	4226	C	ND1	Non-synonymous	M307T	0	0.07	0.17
Modern terminal	R0	T	4226	C	ND1	Non-synonymous	M307T	0	0.07	0.17
Modern terminal	R0	A	4231	G	ND1	Non-synonymous	I309V	0	0.33	0.068
Pre-terminal	R0	A	4231	G	ND1	Non-synonymous	I309V	0	0.33	0.068
Modern terminal	U	A	4231	G	ND1	Non-synonymous	I309V	0	0.33	0.068
Modern terminal	U	A	4231	G	ND1	Non-synonymous	I309V	0	0.33	0.068
Modern terminal	JT	A	4231	G	ND1	Non-synonymous	I309V	0	0.33	0.068
Modern terminal	JT	A	4231	G	ND1	Non-synonymous	I309V	0	0.33	0.068
Ancient terminal	R0	T	4232	C	ND1	Non-synonymous	I309T	0.004	0.14	0.136
Ancient terminal	R0	T	4232	C	ND1	Non-synonymous	I309T	0.004	0.14	0.136
Modern terminal	R0	T	4232	C	ND1	Non-synonymous	I309T	0.004	0.14	0.136
Modern terminal	R0	T	4232	C	ND1	Non-synonymous	I309T	0.004	0.14	0.136
Modern terminal	R0	T	4232	C	ND1	Non-synonymous	I309T	0.004	0.14	0.136
Modern terminal	R0	T	4232	C	ND1	Non-synonymous	I309T	0.004	0.14	0.136
Modern terminal	U	T	4232	C	ND1	Non-synonymous	I309T	0.004	0.14	0.136
Modern terminal	U	T	4232	C	ND1	Non-synonymous	I309T	0.004	0.14	0.136
Modern terminal	U	T	4232	C	ND1	Non-synonymous	I309T	0.004	0.14	0.136
Pre-terminal	U	T	4232	C	ND1	Non-synonymous	I309T	0.004	0.14	0.136
Pre-terminal	U	T	4232	C	ND1	Non-synonymous	I309T	0.004	0.14	0.136
Modern terminal	JT	T	4232	C	ND1	Non-synonymous	I309T	0.004	0.14	0.136
Modern terminal	JT	T	4232	C	ND1	Non-synonymous	I309T	0.004	0.14	0.136
Modern terminal	JT	T	4232	C	ND1	Non-synonymous	I309T	0.004	0.14	0.136
Modern terminal	JT	T	4232	C	ND1	Non-synonymous	I309T	0.004	0.14	0.136
Modern terminal	JT	T	4232	C	ND1	Non-synonymous	I309T	0.004	0.14	0.136
Pre-terminal	JT	T	4232	C	ND1	Non-synonymous	I309T	0.004	0.14	0.136
Pre-terminal	JT	T	4232	C	ND1	Non-synonymous	I309T	0.004	0.14	0.136
Modern terminal	N2	T	4232	C	ND1	Non-synonymous	I309T	0.004	0.14	0.136
Modern terminal	N2	T	4232	C	ND1	Non-synonymous	I309T	0.004	0.14	0.136
Modern terminal	U	A	4234	G	ND1	Non-synonymous	T310A	0	0.94	0.088
Modern terminal	JT	A	4234	G	ND1	Non-synonymous	T310A	0	0.94	0.088
Modern terminal	R0	A	4243	G	ND1	Non-synonymous	S313G	0.003	1	0.065
Modern terminal	R0	A	4243	G	ND1	Non-synonymous	S313G	0.003	1	0.065
Pre-terminal	R0	A	4243	G	ND1	Non-synonymous	S313G	0.003	1	0.065
Pre-terminal	U	A	4243	G	ND1	Non-synonymous	S313G	0.003	1	0.065
Modern terminal	JT	A	4243	G	ND1	Non-synonymous	S313G	0.003	1	0.065
Modern terminal	JT	A	4243	G	ND1	Non-synonymous	S313G	0.003	1	0.065
Pre-terminal	JT	A	4243	G	ND1	Non-synonymous	S313G	0.003	1	0.065
Pre-terminal	R0	G	4244	A	ND1	Non-synonymous	S313N	0	0.07	0.113
Modern terminal	R0	C	4252	T	ND1	Non-synonymous	P315S	1	0	0.175
Ancient terminal	U	T	4475	A	ND2	Non-synonymous	N2K	1	0.12	0.484
Ancient terminal	U	T	4480	A	ND2	Non-synonymous	L4Q	1	0.16	0.433
Modern terminal	JT	A	4486	C	ND2	Non-synonymous	Q6P	1	0.03	0.384
Ancient terminal	R0	G	4491	A	ND2	Non-synonymous	V8I	0	1	0.071
Modern terminal	R0	G	4491	A	ND2	Non-synonymous	V8I	0	1	0.071
Modern terminal	R0	G	4491	A	ND2	Non-synonymous	V8I	0	1	0.071

Modern terminal	R0	G	4491	A	ND2	Non-synonymous	V8I	0	1	0.071
Modern terminal	R0	G	4491	A	ND2	Non-synonymous	V8I	0	1	0.071
Modern terminal	R0	G	4491	A	ND2	Non-synonymous	V8I	0	1	0.071
Modern terminal	R0	G	4491	A	ND2	Non-synonymous	V8I	0	1	0.071
Modern terminal	R0	G	4491	A	ND2	Non-synonymous	V8I	0	1	0.071
Pre-terminal	R0	G	4491	A	ND2	Non-synonymous	V8I	0	1	0.071
Pre-terminal	R0	G	4491	A	ND2	Non-synonymous	V8I	0	1	0.071
Modern terminal	U	G	4491	A	ND2	Non-synonymous	V8I	0	1	0.071
Modern terminal	U	G	4491	A	ND2	Non-synonymous	V8I	0	1	0.071
Modern terminal	U	G	4491	A	ND2	Non-synonymous	V8I	0	1	0.071
Modern terminal	U	G	4491	A	ND2	Non-synonymous	V8I	0	1	0.071
Modern terminal	U	G	4491	A	ND2	Non-synonymous	V8I	0	1	0.071
Pre-terminal	U	G	4491	A	ND2	Non-synonymous	V8I	0	1	0.071
Pre-terminal	U	G	4491	A	ND2	Non-synonymous	V8I	0	1	0.071
Pre-terminal	U	G	4491	A	ND2	Non-synonymous	V8I	0	1	0.071
Ancient terminal	JT	G	4491	A	ND2	Non-synonymous	V8I	0	1	0.071
Modern terminal	JT	G	4491	A	ND2	Non-synonymous	V8I	0	1	0.071
Modern terminal	JT	G	4491	A	ND2	Non-synonymous	V8I	0	1	0.071
Modern terminal	JT	G	4491	A	ND2	Non-synonymous	V8I	0	1	0.071
Pre-terminal	JT	G	4491	A	ND2	Non-synonymous	V8I	0	1	0.071
Pre-terminal	JT	G	4491	A	ND2	Non-synonymous	V8I	0	1	0.071
Modern terminal	N1	G	4491	A	ND2	Non-synonymous	V8I	0	1	0.071
Modern terminal	N2	G	4491	A	ND2	Non-synonymous	V8I	0	1	0.071
Modern terminal	R0	T	4500	A	ND2	Non-synonymous	S11T	0	0.59	0.192
Modern terminal	U	T	4500	C	ND2	Non-synonymous	S11P	0.002	0.2	0.535
Pre-terminal	U	C	4501	T	ND2	Non-synonymous	S11F	0.003	0.77	0.497
Modern terminal	R0	A	4506	G	ND2	Non-synonymous	I13V	0.004	0.68	0.156
Modern terminal	U	A	4506	G	ND2	Non-synonymous	I13V	0.004	0.68	0.156
Modern terminal	U	A	4506	G	ND2	Non-synonymous	I13V	0.004	0.68	0.156
Ancient terminal	JT	T	4507	A	ND2	Non-synonymous	I13N	1	0	0.706
Pre-terminal	JT	T	4509	C	ND2	Non-synonymous	F14L	0.011	1	0.308
Pre-terminal	R0	G	4512	A	ND2	Non-synonymous	A15T	0.001	0.24	0.117
Pre-terminal	R0	G	4512	A	ND2	Non-synonymous	A15T	0.001	0.24	0.117
Ancient terminal	R0	C	4519	A	ND2	Non-synonymous	T17K	1	0	0.646
Modern terminal	X	A	4527	G	ND2	Non-synonymous	T20A	0.086	0.07	0.392
Modern terminal	R0	G	4560	A	ND2	Non-synonymous	V31M	0.071	0.84	0.247
Modern terminal	R0	G	4560	A	ND2	Non-synonymous	V31M	0.071	0.84	0.247
Pre-terminal	R0	G	4560	A	ND2	Non-synonymous	V31M	0.071	0.84	0.247
Modern terminal	N1	G	4560	A	ND2	Non-synonymous	V31M	0.071	0.84	0.247
Ancient terminal	R0	T	4561	C	ND2	Non-synonymous	V31A	0.038	0.08	0.198
Ancient terminal	R0	T	4561	C	ND2	Non-synonymous	V31A	0.038	0.08	0.198
Modern terminal	R0	T	4561	C	ND2	Non-synonymous	V31A	0.038	0.08	0.198
Modern terminal	R0	T	4561	C	ND2	Non-synonymous	V31A	0.038	0.08	0.198
Modern terminal	R0	T	4561	C	ND2	Non-synonymous	V31A	0.038	0.08	0.198
Modern terminal	R0	T	4561	C	ND2	Non-synonymous	V31A	0.038	0.08	0.198
Modern terminal	R0	T	4561	C	ND2	Non-synonymous	V31A	0.038	0.08	0.198
Modern terminal	R0	T	4561	C	ND2	Non-synonymous	V31A	0.038	0.08	0.198
Modern terminal	U	T	4561	C	ND2	Non-synonymous	V31A	0.038	0.08	0.198
Modern terminal	U	T	4561	C	ND2	Non-synonymous	V31A	0.038	0.08	0.198
Modern terminal	U	T	4561	C	ND2	Non-synonymous	V31A	0.038	0.08	0.198

Modern terminal	U	T	4561	C	ND2	Non-synonymous	V31A	0.038	0.08	0.198
Pre-terminal	U	T	4561	C	ND2	Non-synonymous	V31A	0.038	0.08	0.198
Pre-terminal	U	T	4561	C	ND2	Non-synonymous	V31A	0.038	0.08	0.198
Modern terminal	JT	T	4561	C	ND2	Non-synonymous	V31A	0.038	0.08	0.198
Modern terminal	JT	T	4561	C	ND2	Non-synonymous	V31A	0.038	0.08	0.198
Modern terminal	JT	T	4561	C	ND2	Non-synonymous	V31A	0.038	0.08	0.198
Modern terminal	JT	T	4561	C	ND2	Non-synonymous	V31A	0.038	0.08	0.198
Modern terminal	JT	T	4561	C	ND2	Non-synonymous	V31A	0.038	0.08	0.198
Pre-terminal	JT	T	4561	C	ND2	Non-synonymous	V31A	0.038	0.08	0.198
Pre-terminal	JT	T	4561	C	ND2	Non-synonymous	V31A	0.038	0.08	0.198
Modern terminal	N1	T	4561	C	ND2	Non-synonymous	V31A	0.038	0.08	0.198
Modern terminal	N1	T	4561	C	ND2	Non-synonymous	V31A	0.038	0.08	0.198
Pre-terminal	N1	T	4561	C	ND2	Non-synonymous	V31A	0.038	0.08	0.198
Pre-terminal	N1	T	4561	C	ND2	Non-synonymous	V31A	0.038	0.08	0.198
Ancient terminal	X	T	4561	C	ND2	Non-synonymous	V31A	0.038	0.08	0.198
Pre-terminal	X	T	4561	C	ND2	Non-synonymous	V31A	0.038	0.08	0.198
Modern terminal	U	T	4579	C	ND2	Non-synonymous	M37T	0.425	0.58	0.801
Modern terminal	R0	T	4587	C	ND2	Non-synonymous	F40L	0.977	0.01	0.789
Modern terminal	N1	C	4593	T	ND2	Non-synonymous	P42S	0.807	0	0.73
Ancient terminal	X	C	4593	T	ND2	Non-synonymous	P42S	0.807	0	0.73
Modern terminal	N1	C	4594	T	ND2	Non-synonymous	P42L	0.999	0	0.783
Ancient terminal	X	C	4594	T	ND2	Non-synonymous	P42L	0.999	0	0.783
Modern terminal	R0	G	4596	A	ND2	Non-synonymous	V43I	0	0.98	0.08
Modern terminal	U	G	4596	A	ND2	Non-synonymous	V43I	0	0.98	0.08
Modern terminal	U	G	4596	A	ND2	Non-synonymous	V43I	0	0.98	0.08
Modern terminal	U	G	4596	A	ND2	Non-synonymous	V43I	0	0.98	0.08
Modern terminal	JT	G	4596	A	ND2	Non-synonymous	V43I	0	0.98	0.08
Modern terminal	JT	G	4596	A	ND2	Non-synonymous	V43I	0	0.98	0.08
Modern terminal	R0	T	4612	C	ND2	Non-synonymous	M48T	0.001	0.24	0.166
Modern terminal	U	T	4612	C	ND2	Non-synonymous	M48T	0.001	0.24	0.166
Ancient terminal	R0	A	4615	G	ND2	Non-synonymous	N49S	0.045	0.24	0.419
Pre-terminal	R0	A	4615	G	ND2	Non-synonymous	N49S	0.045	0.24	0.419
Pre-terminal	U	A	4615	G	ND2	Non-synonymous	N49S	0.045	0.24	0.419
Modern terminal	JT	A	4615	G	ND2	Non-synonymous	N49S	0.045	0.24	0.419
Ancient terminal	JT	G	4635	T	ND2	Non-synonymous	A56S	0.078	0.49	0.094
Modern terminal	R0	A	4638	G	ND2	Non-synonymous	I57V	0.016	0.04	0.088
Ancient terminal	U	A	4638	G	ND2	Non-synonymous	I57V	0.016	0.04	0.088
Modern terminal	U	A	4638	G	ND2	Non-synonymous	I57V	0.016	0.04	0.088
Modern terminal	R0	T	4639	C	ND2	Non-synonymous	I57T	0.006	1	0.047
Modern terminal	R0	T	4639	C	ND2	Non-synonymous	I57T	0.006	1	0.047
Modern terminal	R0	T	4639	C	ND2	Non-synonymous	I57T	0.006	1	0.047
Modern terminal	R0	T	4639	C	ND2	Non-synonymous	I57T	0.006	1	0.047
Modern terminal	R0	T	4639	C	ND2	Non-synonymous	I57T	0.006	1	0.047
Pre-terminal	R0	T	4639	C	ND2	Non-synonymous	I57T	0.006	1	0.047
Pre-terminal	R0	T	4639	C	ND2	Non-synonymous	I57T	0.006	1	0.047
Pre-terminal	R0	T	4639	C	ND2	Non-synonymous	I57T	0.006	1	0.047
Pre-terminal	R0	T	4639	C	ND2	Non-synonymous	I57T	0.006	1	0.047
Modern terminal	U	T	4639	C	ND2	Non-synonymous	I57T	0.006	1	0.047
Modern terminal	U	T	4639	C	ND2	Non-synonymous	I57T	0.006	1	0.047
Modern terminal	U	T	4639	C	ND2	Non-synonymous	I57T	0.006	1	0.047

Modern terminal	U	T	4639	C	ND2	Non-synonymous	I57T	0.006	1	0.047
Modern terminal	U	T	4639	C	ND2	Non-synonymous	I57T	0.006	1	0.047
Pre-terminal	U	T	4639	C	ND2	Non-synonymous	I57T	0.006	1	0.047
Pre-terminal	U	T	4639	C	ND2	Non-synonymous	I57T	0.006	1	0.047
Pre-terminal	U	T	4639	C	ND2	Non-synonymous	I57T	0.006	1	0.047
Pre-terminal	U	T	4639	C	ND2	Non-synonymous	I57T	0.006	1	0.047
Modern terminal	JT	T	4639	C	ND2	Non-synonymous	I57T	0.006	1	0.047
Modern terminal	JT	T	4639	C	ND2	Non-synonymous	I57T	0.006	1	0.047
Modern terminal	JT	T	4639	C	ND2	Non-synonymous	I57T	0.006	1	0.047
Modern terminal	JT	T	4639	C	ND2	Non-synonymous	I57T	0.006	1	0.047
Modern terminal	JT	T	4639	C	ND2	Non-synonymous	I57T	0.006	1	0.047
Pre-terminal	JT	T	4639	C	ND2	Non-synonymous	I57T	0.006	1	0.047
Modern terminal	R0	T	4648	C	ND2	Non-synonymous	F60S	1	0	0.617
Modern terminal	U	C	4650	T	ND2	Non-synonymous	L61F	1	0.03	0.464
Pre-terminal	R0	C	4654	T	ND2	Non-synonymous	T62M	0.027	0.01	0.355
Pre-terminal	U	C	4654	T	ND2	Non-synonymous	T62M	0.027	0.01	0.355
Modern terminal	R0	G	4659	A	ND2	Non-synonymous	A64T	0.019	0.01	0.256
Modern terminal	R0	G	4659	A	ND2	Non-synonymous	A64T	0.019	0.01	0.256
Modern terminal	R0	G	4659	A	ND2	Non-synonymous	A64T	0.019	0.01	0.256
Modern terminal	R0	G	4659	A	ND2	Non-synonymous	A64T	0.019	0.01	0.256
Modern terminal	R0	G	4659	A	ND2	Non-synonymous	A64T	0.019	0.01	0.256
Modern terminal	R0	G	4659	A	ND2	Non-synonymous	A64T	0.019	0.01	0.256
Modern terminal	R0	G	4659	A	ND2	Non-synonymous	A64T	0.019	0.01	0.256
Pre-terminal	R0	G	4659	A	ND2	Non-synonymous	A64T	0.019	0.01	0.256
Modern terminal	U	G	4659	A	ND2	Non-synonymous	A64T	0.019	0.01	0.256
Modern terminal	U	G	4659	A	ND2	Non-synonymous	A64T	0.019	0.01	0.256
Modern terminal	U	G	4659	A	ND2	Non-synonymous	A64T	0.019	0.01	0.256
Modern terminal	U	G	4659	A	ND2	Non-synonymous	A64T	0.019	0.01	0.256
Pre-terminal	U	G	4659	A	ND2	Non-synonymous	A64T	0.019	0.01	0.256
Pre-terminal	U	G	4659	A	ND2	Non-synonymous	A64T	0.019	0.01	0.256
Modern terminal	JT	G	4659	A	ND2	Non-synonymous	A64T	0.019	0.01	0.256
Pre-terminal	JT	G	4659	A	ND2	Non-synonymous	A64T	0.019	0.01	0.256
Pre-terminal	X	G	4659	A	ND2	Non-synonymous	A64T	0.019	0.01	0.256
Pre-terminal	N2	G	4659	A	ND2	Non-synonymous	A64T	0.019	0.01	0.256
Ancient terminal	U	C	4660	T	ND2	Non-synonymous	A64V	0.869	0	0.332
Pre-terminal	R0	A	4674	G	ND2	Non-synonymous	I69V	0.017	0.01	0.264
Pre-terminal	R0	A	4674	G	ND2	Non-synonymous	I69V	0.017	0.01	0.264
Modern terminal	U	A	4674	G	ND2	Non-synonymous	I69V	0.017	0.01	0.264
Modern terminal	U	A	4674	G	ND2	Non-synonymous	I69V	0.017	0.01	0.264
Pre-terminal	U	A	4674	G	ND2	Non-synonymous	I69V	0.017	0.01	0.264
Modern terminal	JT	A	4674	G	ND2	Non-synonymous	I69V	0.017	0.01	0.264
Pre-terminal	N1	A	4674	G	ND2	Non-synonymous	I69V	0.017	0.01	0.264
Modern terminal	U	C	4677	T	ND2	Non-synonymous	L70F	0.05	0.02	0.582
Ancient terminal	X	C	4677	T	ND2	Non-synonymous	L70F	0.05	0.02	0.582
Pre-terminal	R0	A	4689	G	ND2	Non-synonymous	I74V	0.998	0.58	0.134
Modern terminal	R0	T	4695	C	ND2	Non-synonymous	F76L	0	0.19	0.373
Modern terminal	R0	T	4695	C	ND2	Non-synonymous	F76L	0	0.19	0.373
Modern terminal	U	T	4695	C	ND2	Non-synonymous	F76L	0	0.19	0.373
Pre-terminal	JT	T	4695	C	ND2	Non-synonymous	F76L	0	0.19	0.373
Pre-terminal	JT	T	4695	C	ND2	Non-synonymous	F76L	0	0.19	0.373

Pre-terminal	JT	T	4695	C	ND2	Non-synonymous	F76L	0	0.19	0.373
Modern terminal	N1	T	4695	C	ND2	Non-synonymous	F76L	0	0.19	0.373
Pre-terminal	JT	T	4696	C	ND2	Non-synonymous	F76S	0.002	0.13	0.437
Modern terminal	R0	A	4702	G	ND2	Non-synonymous	N78S	0.506	0.15	0.286
Modern terminal	JT	A	4702	G	ND2	Non-synonymous	N78S	0.506	0.15	0.286
Modern terminal	JT	A	4702	G	ND2	Non-synonymous	N78S	0.506	0.15	0.286
Modern terminal	U	A	4704	G	ND2	Non-synonymous	M79V	0.666	0.35	0.349
Modern terminal	R0	T	4705	C	ND2	Non-synonymous	M79T	0.898	0.22	0.462
Modern terminal	R0	A	4706	C	ND2	Non-synonymous	M79I	0.076	0.3	0.295
Modern terminal	R0	C	4707	A	ND2	Non-synonymous	L80I	0.049	0.26	0.155
Ancient terminal	R0	C	4716	A	ND2	Non-synonymous	Q83K	0.739	0.01	0.504
Modern terminal	R0	A	4722	G	ND2	Non-synonymous	T85A	0.022	0.1	0.29
Modern terminal	R0	A	4722	G	ND2	Non-synonymous	T85A	0.022	0.1	0.29
Modern terminal	R0	A	4722	G	ND2	Non-synonymous	T85A	0.022	0.1	0.29
Pre-terminal	R0	A	4722	G	ND2	Non-synonymous	T85A	0.022	0.1	0.29
Modern terminal	U	A	4722	G	ND2	Non-synonymous	T85A	0.022	0.1	0.29
Modern terminal	JT	A	4722	G	ND2	Non-synonymous	T85A	0.022	0.1	0.29
Modern terminal	X	A	4722	G	ND2	Non-synonymous	T85A	0.022	0.1	0.29
Pre-terminal	X	A	4722	G	ND2	Non-synonymous	T85A	0.022	0.1	0.29
Ancient terminal	JT	T	4726	C	ND2	Non-synonymous	M86T	0	0.2	0.22
Pre-terminal	U	A	4728	G	ND2	Non-synonymous	T87A	0.002	0.3	0.287
Modern terminal	R0	A	4732	G	ND2	Non-synonymous	N88S	0.012	0.14	0.148
Ancient terminal	JT	A	4732	G	ND2	Non-synonymous	N88S	0.012	0.14	0.148
Modern terminal	JT	A	4732	G	ND2	Non-synonymous	N88S	0.012	0.14	0.148
Modern terminal	JT	A	4734	G	ND2	Non-synonymous	T89A	0	0.22	0.118
Pre-terminal	R0	C	4735	T	ND2	Non-synonymous	T89I	0	0.42	0.115
Modern terminal	R0	A	4740	G	ND2	Non-synonymous	N91D	0.058	0.09	0.254
Modern terminal	JT	C	4743	A	ND2	Non-synonymous	Q92K	0.036	0.01	0.219
Ancient terminal	U	A	4745	T	ND2	Non-synonymous	Q92H	0.984	0.03	0.212
Pre-terminal	U	A	4745	T	ND2	Non-synonymous	Q92H	0.984	0.03	0.212
Modern terminal	U	T	4746	C	ND2	Non-synonymous	Y93H	0.999	0.42	0.166
Modern terminal	U	T	4749	C	ND2	Non-synonymous	S94P	0.003	0.12	0.243
Modern terminal	U	T	4749	C	ND2	Non-synonymous	S94P	0.003	0.12	0.243
Modern terminal	U	T	4752	C	ND2	Non-synonymous	S95P	0.016	0.05	0.575
Modern terminal	R0	A	4761	G	ND2	Non-synonymous	I98V	0.015	0.14	0.132
Pre-terminal	R0	A	4761	G	ND2	Non-synonymous	I98V	0.015	0.14	0.132
Ancient terminal	R0	T	4762	C	ND2	Non-synonymous	I98T	0.145	0.13	0.308
Ancient terminal	U	T	4762	A	ND2	Non-synonymous	I98T	0.145	0.13	0.308
Pre-terminal	R0	C	4763	A	ND2	Non-synonymous	I98M	0.163	1	0.121
Ancient terminal	JT	T	4765	A	ND2	Non-synonymous	M99K	0.008	0.01	0.53
Modern terminal	R0	A	4767	G	ND2	Non-synonymous	M100V	0.003	0.37	0.284
Pre-terminal	R0	A	4767	G	ND2	Non-synonymous	M100V	0.003	0.37	0.284
Pre-terminal	R0	A	4767	G	ND2	Non-synonymous	M100V	0.003	0.37	0.284
Pre-terminal	R0	A	4767	G	ND2	Non-synonymous	M100V	0.003	0.37	0.284
Modern terminal	U	A	4767	G	ND2	Non-synonymous	M100V	0.003	0.37	0.284
Modern terminal	JT	A	4767	G	ND2	Non-synonymous	M100V	0.003	0.37	0.284
Modern terminal	JT	A	4767	G	ND2	Non-synonymous	M100V	0.003	0.37	0.284
Modern terminal	JT	A	4767	G	ND2	Non-synonymous	M100V	0.003	0.37	0.284
Pre-terminal	JT	A	4767	G	ND2	Non-synonymous	M100V	0.003	0.37	0.284
Modern terminal	R0	T	4768	C	ND2	Non-synonymous	M100T	0	0.32	0.242

Modern terminal	N1	T	4768	C	ND2	Non-synonymous	M100T	0	0.32	0.242
Modern terminal	JT	A	4769	C	ND2	Non-synonymous	M100I	0	1	0.233
Modern terminal	JT	A	4769	T	ND2	Non-synonymous	M100I	0	1	0.233
Ancient terminal	U	T	4792	A	ND2	Non-synonymous	M108K	0.99	0	0.68
Modern terminal	X	C	4795	T	ND2	Non-synonymous	A109V	0.974	0.14	0.344
Ancient terminal	U	C	4797	A	ND2	Non-synonymous	P110T	1	0	0.607
Pre-terminal	R0	G	4812	C	ND2	Non-synonymous	V115L	0	0.28	0.537
Modern terminal	JT	G	4812	A	ND2	Non-synonymous	V115I	0.003	0.06	0.24
Modern terminal	N1	T	4813	C	ND2	Non-synonymous	V115A	0.99	0	0.611
Modern terminal	U	A	4824	G	ND2	Non-synonymous	T119A	0.613	0.16	0.441
Modern terminal	U	A	4824	G	ND2	Non-synonymous	T119A	0.613	0.16	0.441
Pre-terminal	JT	A	4824	G	ND2	Non-synonymous	T119A	0.613	0.16	0.441
Pre-terminal	JT	A	4824	G	ND2	Non-synonymous	T119A	0.613	0.16	0.441
Ancient terminal	X	G	4830	A	ND2	Non-synonymous	G121S	1	0	0.575
Ancient terminal	R0	C	4836	G	ND2	Non-synonymous	P123A	0.03	0.06	0.335
Ancient terminal	R0	C	4837	A	ND2	Non-synonymous	P123H	0.999	0.08	0.522
Modern terminal	R0	C	4843	T	ND2	Non-synonymous	T125M	0.003	0.29	0.169
Modern terminal	U	C	4843	T	ND2	Non-synonymous	T125M	0.003	0.29	0.169
Pre-terminal	U	C	4843	T	ND2	Non-synonymous	T125M	0.003	0.29	0.169
Modern terminal	JT	C	4843	T	ND2	Non-synonymous	T125M	0.003	0.29	0.169
Pre-terminal	JT	C	4843	T	ND2	Non-synonymous	T125M	0.003	0.29	0.169
Modern terminal	JT	A	4884	G	ND2	Non-synonymous	I139V	0.015	0.01	0.117
Modern terminal	N1	A	4884	G	ND2	Non-synonymous	I139V	0.015	0.01	0.117
Pre-terminal	X	A	4884	G	ND2	Non-synonymous	I139V	0.015	0.01	0.117
Modern terminal	R0	A	4890	G	ND2	Non-synonymous	I141V	0.998	0.48	0.11
Modern terminal	JT	A	4890	G	ND2	Non-synonymous	I141V	0.998	0.48	0.11
Pre-terminal	JT	A	4890	G	ND2	Non-synonymous	I141V	0.998	0.48	0.11
Modern terminal	N1	A	4890	G	ND2	Non-synonymous	I141V	0.998	0.48	0.11
Modern terminal	X	A	4890	G	ND2	Non-synonymous	I141V	0.998	0.48	0.11
Modern terminal	U	A	4893	G	ND2	Non-synonymous	M142V	0.997	0	0.467
Ancient terminal	U	T	4896	A	ND2	Non-synonymous	Y143N	1	0.01	0.796
Pre-terminal	U	T	4896	C	ND2	Non-synonymous	Y143H	1	0.03	0.63
Pre-terminal	JT	A	4902	G	ND2	Non-synonymous	I145V	0.017	0.08	0.226
Modern terminal	N1	A	4902	G	ND2	Non-synonymous	I145V	0.017	0.08	0.226
Modern terminal	R0	C	4904	A	ND2	Non-synonymous	I145M	0.87	0.13	0.392
Modern terminal	U	C	4904	A	ND2	Non-synonymous	I145M	0.87	0.13	0.392
Pre-terminal	U	C	4908	T	ND2	Non-synonymous	P147S	0.004	0.11	0.354
Ancient terminal	U	A	4917	G	ND2	Non-synonymous	N150D	0.385	0.11	0.402
Modern terminal	U	A	4917	G	ND2	Non-synonymous	N150D	0.385	0.11	0.402
Modern terminal	U	A	4917	G	ND2	Non-synonymous	N150D	0.385	0.11	0.402
Modern terminal	U	A	4917	G	ND2	Non-synonymous	N150D	0.385	0.11	0.402
Modern terminal	JT	A	4917	G	ND2	Non-synonymous	N150D	0.385	0.11	0.402
Modern terminal	JT	A	4917	G	ND2	Non-synonymous	N150D	0.385	0.11	0.402
Modern terminal	JT	A	4917	G	ND2	Non-synonymous	N150D	0.385	0.11	0.402
Pre-terminal	N2	A	4917	G	ND2	Non-synonymous	N150D	0.385	0.11	0.402
Ancient terminal	R0	T	4921	A	ND2	Non-synonymous	V151E	0.973	0.08	0.291
Modern terminal	U	T	4921	C	ND2	Non-synonymous	V151A	0.89	0.47	0.135
Modern terminal	R0	G	4924	A	ND2	Non-synonymous	S152N	0.003	1	0.067
Modern terminal	R0	G	4924	A	ND2	Non-synonymous	S152N	0.003	1	0.067
Modern terminal	R0	G	4924	A	ND2	Non-synonymous	S152N	0.003	1	0.067

Modern terminal	R0	G	4924	A	ND2	Non-synonymous	S152N	0.003	1	0.067
Pre-terminal	R0	G	4924	A	ND2	Non-synonymous	S152N	0.003	1	0.067
Pre-terminal	R0	G	4924	A	ND2	Non-synonymous	S152N	0.003	1	0.067
Pre-terminal	R0	G	4924	A	ND2	Non-synonymous	S152N	0.003	1	0.067
Pre-terminal	R0	G	4924	A	ND2	Non-synonymous	S152N	0.003	1	0.067
Pre-terminal	R0	G	4924	A	ND2	Non-synonymous	S152N	0.003	1	0.067
Modern terminal	U	G	4924	A	ND2	Non-synonymous	S152N	0.003	1	0.067
Modern terminal	U	G	4924	A	ND2	Non-synonymous	S152N	0.003	1	0.067
Modern terminal	U	G	4924	A	ND2	Non-synonymous	S152N	0.003	1	0.067
Modern terminal	U	G	4924	C	ND2	Non-synonymous	S152T	0.026	0.42	0.103
Pre-terminal	U	G	4924	C	ND2	Non-synonymous	S152T	0.026	0.42	0.103
Pre-terminal	U	G	4924	A	ND2	Non-synonymous	S152N	0.003	1	0.067
Modern terminal	JT	G	4924	A	ND2	Non-synonymous	S152N	0.003	1	0.067
Modern terminal	JT	G	4924	C	ND2	Non-synonymous	S152T	0.026	0.42	0.103
Pre-terminal	JT	G	4924	A	ND2	Non-synonymous	S152N	0.003	1	0.067
Modern terminal	N1	G	4924	A	ND2	Non-synonymous	S152N	0.003	1	0.067
Ancient terminal	R0	A	4935	T	ND2	Non-synonymous	T156S	0.26	0.16	0.217
Modern terminal	R0	C	4936	T	ND2	Non-synonymous	T156I	0.002	0.62	0.467
Pre-terminal	U	C	4936	T	ND2	Non-synonymous	T156I	0.002	0.62	0.467
Pre-terminal	U	C	4936	G	ND2	Non-synonymous	T156S	0.26	0.16	0.217
Pre-terminal	JT	C	4936	T	ND2	Non-synonymous	T156I	0.002	0.62	0.467
Modern terminal	U	C	4938	T	ND2	Non-synonymous	L157F	0.064	0.1	0.547
Modern terminal	R0	T	4939	G	ND2	Non-synonymous	L157R	0.985	0	0.896
Modern terminal	U	A	4944	G	ND2	Non-synonymous	I159V	0.015	0.33	0.287
Modern terminal	U	A	4944	G	ND2	Non-synonymous	I159V	0.015	0.33	0.287
Pre-terminal	U	A	4944	G	ND2	Non-synonymous	I159V	0.015	0.33	0.287
Modern terminal	JT	A	4944	G	ND2	Non-synonymous	I159V	0.015	0.33	0.287
Modern terminal	U	C	4946	A	ND2	Non-synonymous	I159M	0.764	0.21	0.36
Modern terminal	R0	G	4959	A	ND2	Non-synonymous	A164T	0.011	0	0.327
Modern terminal	R0	G	4959	A	ND2	Non-synonymous	A164T	0.011	0	0.327
Pre-terminal	R0	G	4959	A	ND2	Non-synonymous	A164T	0.011	0	0.327
Pre-terminal	R0	G	4959	A	ND2	Non-synonymous	A164T	0.011	0	0.327
Modern terminal	U	G	4959	A	ND2	Non-synonymous	A164T	0.011	0	0.327
Modern terminal	U	G	4959	A	ND2	Non-synonymous	A164T	0.011	0	0.327
Modern terminal	U	G	4959	A	ND2	Non-synonymous	A164T	0.011	0	0.327
Pre-terminal	U	G	4959	A	ND2	Non-synonymous	A164T	0.011	0	0.327
Pre-terminal	U	G	4959	A	ND2	Non-synonymous	A164T	0.011	0	0.327
Pre-terminal	U	G	4959	A	ND2	Non-synonymous	A164T	0.011	0	0.327
Pre-terminal	JT	G	4959	A	ND2	Non-synonymous	A164T	0.011	0	0.327
Ancient terminal	X	G	4959	A	ND2	Non-synonymous	A164T	0.011	0	0.327
Modern terminal	R0	C	4960	T	ND2	Non-synonymous	A164V	0.001	0.85	0.303
Pre-terminal	U	C	4960	G	ND2	Non-synonymous	A164G	0.63	0	0.564
Pre-terminal	N2	C	4960	T	ND2	Non-synonymous	A164V	0.001	0.85	0.303
Pre-terminal	U	A	4965	G	ND2	Non-synonymous	S166G	0.267	1	0.15
Ancient terminal	U	T	4968	A	ND2	Nonsense				
Pre-terminal	U	T	4968	G	ND2	Non-synonymous	W167G	1	0	0.927
Pre-terminal	U	G	4975	A	ND2	Non-synonymous	G169E	1	0	0.894
Pre-terminal	U	T	4977	A	ND2	Non-synonymous	L170M	1	0	0.649
Modern terminal	JT	A	4984	G	ND2	Non-synonymous	Q172R	0.988	0	0.822
Ancient terminal	X	G	4996	A	ND2	Non-synonymous	R176H	0.311	0.01	0.666

Pre-terminal	U	A	4999	C	ND2	Non-synonymous	K177T	1	0	0.779
Pre-terminal	R0	A	5001	G	ND2	Non-synonymous	I178V	0.812	0.03	0.475
Pre-terminal	R0	A	5001	G	ND2	Non-synonymous	I178V	0.812	0.03	0.475
Pre-terminal	R0	A	5019	G	ND2	Non-synonymous	I184V	0.998	0	0.468
Pre-terminal	R0	A	5028	G	ND2	Non-synonymous	M187V	0.06	0.03	0.683
Modern terminal	N1	A	5028	G	ND2	Non-synonymous	M187V	0.06	0.03	0.683
Ancient terminal	X	A	5028	G	ND2	Non-synonymous	M187V	0.06	0.03	0.683
Modern terminal	R0	T	5029	C	ND2	Non-synonymous	M187T	0.038	0.02	0.751
Modern terminal	R0	T	5029	C	ND2	Non-synonymous	M187T	0.038	0.02	0.751
Modern terminal	U	T	5029	C	ND2	Non-synonymous	M187T	0.038	0.02	0.751
Modern terminal	JT	A	5040	G	ND2	Non-synonymous	M191V	0.317	0.96	0.562
Ancient terminal	JT	T	5041	C	ND2	Non-synonymous	M191T	0.027	1	0.224
Ancient terminal	R0	G	5043	T	ND2	Non-synonymous	A192S	0.394	0.18	0.479
Modern terminal	R0	G	5046	A	ND2	Non-synonymous	V193I	0.003	1	0.099
Modern terminal	R0	G	5046	A	ND2	Non-synonymous	V193I	0.003	1	0.099
Pre-terminal	R0	G	5046	A	ND2	Non-synonymous	V193I	0.003	1	0.099
Modern terminal	JT	G	5046	A	ND2	Non-synonymous	V193I	0.003	1	0.099
Ancient terminal	R0	T	5047	A	ND2	Non-synonymous	V193D	1	0	0.696
Modern terminal	R0	A	5067	G	ND2	Non-synonymous	M200V	0.041	0.02	0.422
Modern terminal	R0	A	5067	G	ND2	Non-synonymous	M200V	0.041	0.02	0.422
Pre-terminal	N2	A	5067	G	ND2	Non-synonymous	M200V	0.041	0.02	0.422
Modern terminal	R0	A	5070	G	ND2	Non-synonymous	T201A	0.169	0.26	0.45
Modern terminal	U	A	5070	G	ND2	Non-synonymous	T201A	0.169	0.26	0.45
Modern terminal	U	A	5070	G	ND2	Non-synonymous	T201A	0.169	0.26	0.45
Modern terminal	U	A	5073	G	ND2	Non-synonymous	I202V	0.061	0.04	0.137
Modern terminal	R0	T	5074	C	ND2	Non-synonymous	I202T	0.68	0.07	0.313
Modern terminal	R0	T	5074	C	ND2	Non-synonymous	I202T	0.68	0.07	0.313
Ancient terminal	U	T	5074	C	ND2	Non-synonymous	I202T	0.68	0.07	0.313
Modern terminal	U	T	5074	C	ND2	Non-synonymous	I202T	0.68	0.07	0.313
Pre-terminal	U	T	5074	C	ND2	Non-synonymous	I202T	0.68	0.07	0.313
Pre-terminal	U	T	5074	C	ND2	Non-synonymous	I202T	0.68	0.07	0.313
Ancient terminal	JT	T	5074	A	ND2	Non-synonymous	I202N	1	0	0.588
Modern terminal	U	C	5076	T	ND2	Non-synonymous	L203F	0.012	0.02	0.457
Ancient terminal	JT	A	5079	G	ND2	Non-synonymous	N204D	0.867	0.01	0.393
Pre-terminal	R0	A	5080	G	ND2	Non-synonymous	N204S	0.029	0.06	0.335
Pre-terminal	R0	C	5086	T	ND2	Non-synonymous	T206I	0.004	1	0.317
Pre-terminal	R0	C	5086	T	ND2	Non-synonymous	T206I	0.004	1	0.317
Pre-terminal	JT	C	5086	T	ND2	Non-synonymous	T206I	0.004	1	0.317
Ancient terminal	R0	A	5092	G	ND2	Non-synonymous	Y208C	1	0	0.652
Ancient terminal	N1	A	5092	G	ND2	Non-synonymous	Y208C	1	0	0.652
Modern terminal	R0	A	5094	C	ND2	Non-synonymous	I209L	0.046	0.11	0.421
Modern terminal	R0	T	5095	C	ND2	Non-synonymous	I209T	1	0.07	0.485
Pre-terminal	R0	T	5095	C	ND2	Non-synonymous	I209T	1	0.07	0.485
Modern terminal	U	T	5095	C	ND2	Non-synonymous	I209T	1	0.07	0.485
Pre-terminal	U	T	5095	C	ND2	Non-synonymous	I209T	1	0.07	0.485
Ancient terminal	JT	T	5095	C	ND2	Non-synonymous	I209T	1	0.07	0.485
Modern terminal	JT	T	5095	C	ND2	Non-synonymous	I209T	1	0.07	0.485
Ancient terminal	X	C	5104	T	ND2	Non-synonymous	T212I	0.984	0	0.554
Ancient terminal	R0	A	5106	G	ND2	Non-synonymous	T213A	0.703	0.43	0.333
Modern terminal	JT	C	5107	T	ND2	Non-synonymous	T213I	0.134	0.56	0.361

Ancient terminal	U	T	5115	C	ND2	Non-synonymous	F216L	0.722	0	0.59
Ancient terminal	JT	C	5121	G	ND2	Non-synonymous	L218V	0.001	0.39	0.353
Modern terminal	JT	A	5127	G	ND2	Non-synonymous	N220D	0.02	0.54	0.288
Modern terminal	R0	A	5128	G	ND2	Non-synonymous	N220S	0.869	0.72	0.273
Modern terminal	R0	A	5128	G	ND2	Non-synonymous	N220S	0.869	0.72	0.273
Pre-terminal	N2	A	5128	G	ND2	Non-synonymous	N220S	0.869	0.72	0.273
Pre-terminal	R0	A	5134	G	ND2	Non-synonymous	N222S	0.003	0.52	0.155
Modern terminal	N1	A	5134	G	ND2	Non-synonymous	N222S	0.003	0.52	0.155
Modern terminal	JT	C	5137	T	ND2	Non-synonymous	S223F	1	0.05	0.459
Modern terminal	JT	C	5146	T	ND2	Non-synonymous	T226M	1	0	0.252
Modern terminal	X	C	5149	T	ND2	Non-synonymous	T227I	0.034	0.6	0.295
Modern terminal	JT	C	5178	A	ND2	Non-synonymous	L237M	0.998	0.35	0.075
Modern terminal	R0	A	5181	G	ND2	Non-synonymous	T238A	0.002	0.06	0.113
Ancient terminal	U	A	5181	G	ND2	Non-synonymous	T238A	0.002	0.06	0.113
Modern terminal	JT	A	5181	G	ND2	Non-synonymous	T238A	0.002	0.06	0.113
Pre-terminal	U	A	5186	C	ND2	Non-synonymous	W239C	1	0.04	0.242
Modern terminal	R0	C	5187	G	ND2	Non-synonymous	L240V	0.034	0.16	0.181
Modern terminal	R0	C	5191	T	ND2	Non-synonymous	T241M	0.002	0.14	0.151
Ancient terminal	U	C	5191	G	ND2	Nonsense				
Modern terminal	U	C	5191	T	ND2	Non-synonymous	T241M	0.002	0.14	0.151
Ancient terminal	JT	C	5191	T	ND2	Non-synonymous	T241M	0.002	0.14	0.151
Modern terminal	JT	C	5191	T	ND2	Non-synonymous	T241M	0.002	0.14	0.151
Modern terminal	U	C	5194	T	ND2	Non-synonymous	P242L	0.325	0.35	0.176
Pre-terminal	U	C	5194	T	ND2	Non-synonymous	P242L	0.325	0.35	0.176
Pre-terminal	JT	C	5194	T	ND2	Non-synonymous	P242L	0.325	0.35	0.176
Modern terminal	N1	T	5196	G	ND2	Non-synonymous	L243V	0.034	0.29	0.192
Modern terminal	U	A	5199	G	ND2	Non-synonymous	I244V	0.006	0.24	0.107
Modern terminal	U	C	5202	T	ND2	Non-synonymous	P245S	0.024	0.23	0.245
Pre-terminal	R0	T	5205	C	ND2	Non-synonymous	S246P	0.004	0.05	0.483
Modern terminal	JT	T	5205	G	ND2	Non-synonymous	S246A	0.001	0.5	0.162
Ancient terminal	R0	C	5206	T	ND2	Non-synonymous	S246F	0.015	0.04	0.303
Pre-terminal	R0	C	5206	T	ND2	Non-synonymous	S246F	0.015	0.04	0.303
Pre-terminal	R0	C	5206	T	ND2	Non-synonymous	S246F	0.015	0.04	0.303
Pre-terminal	R0	C	5206	T	ND2	Non-synonymous	S246F	0.015	0.04	0.303
Modern terminal	U	C	5206	T	ND2	Non-synonymous	S246F	0.015	0.04	0.303
Modern terminal	JT	C	5206	T	ND2	Non-synonymous	S246F	0.015	0.04	0.303
Modern terminal	X	C	5206	G	ND2	Non-synonymous	S246C	0.965	0.05	0.356
Modern terminal	R0	A	5208	G	ND2	Non-synonymous	T247A	0.003	0.21	0.325
Modern terminal	R0	A	5208	G	ND2	Non-synonymous	T247A	0.003	0.21	0.325
Modern terminal	U	A	5208	G	ND2	Non-synonymous	T247A	0.003	0.21	0.325
Pre-terminal	N2	C	5211	T	ND2	Non-synonymous	L248F	0.979	0	0.411
Pre-terminal	N2	C	5211	T	ND2	Non-synonymous	L248F	0.979	0	0.411
Ancient terminal	JT	G	5223	A	ND2	Nonsense				
Ancient terminal	N2	G	5226	T	ND2	Non-synonymous	G253C	1	0	0.79
Modern terminal	R0	G	5262	A	ND2	Non-synonymous	A265T	0.001	0.02	0.431
Modern terminal	R0	G	5262	A	ND2	Non-synonymous	A265T	0.001	0.02	0.431
Modern terminal	R0	G	5262	A	ND2	Non-synonymous	A265T	0.001	0.02	0.431
Modern terminal	U	G	5262	A	ND2	Non-synonymous	A265T	0.001	0.02	0.431
Modern terminal	U	G	5262	A	ND2	Non-synonymous	A265T	0.001	0.02	0.431
Modern terminal	U	G	5262	A	ND2	Non-synonymous	A265T	0.001	0.02	0.431

Pre-terminal	U	G	5262	A	ND2	Non-synonymous	A265T	0.001	0.02	0.431
Modern terminal	R0	C	5263	T	ND2	Non-synonymous	A265V	0.001	0.05	0.483
Modern terminal	R0	C	5263	T	ND2	Non-synonymous	A265V	0.001	0.05	0.483
Modern terminal	R0	C	5263	T	ND2	Non-synonymous	A265V	0.001	0.05	0.483
Modern terminal	R0	C	5263	T	ND2	Non-synonymous	A265V	0.001	0.05	0.483
Modern terminal	R0	C	5263	T	ND2	Non-synonymous	A265V	0.001	0.05	0.483
Modern terminal	R0	C	5263	T	ND2	Non-synonymous	A265V	0.001	0.05	0.483
Pre-terminal	R0	C	5263	T	ND2	Non-synonymous	A265V	0.001	0.05	0.483
Pre-terminal	R0	C	5263	T	ND2	Non-synonymous	A265V	0.001	0.05	0.483
Pre-terminal	R0	C	5263	T	ND2	Non-synonymous	A265V	0.001	0.05	0.483
Ancient terminal	U	C	5263	T	ND2	Non-synonymous	A265V	0.001	0.05	0.483
Modern terminal	U	C	5263	T	ND2	Non-synonymous	A265V	0.001	0.05	0.483
Modern terminal	U	C	5263	T	ND2	Non-synonymous	A265V	0.001	0.05	0.483
Modern terminal	U	C	5263	T	ND2	Non-synonymous	A265V	0.001	0.05	0.483
Modern terminal	U	C	5263	T	ND2	Non-synonymous	A265V	0.001	0.05	0.483
Modern terminal	U	C	5263	T	ND2	Non-synonymous	A265V	0.001	0.05	0.483
Pre-terminal	U	C	5263	T	ND2	Non-synonymous	A265V	0.001	0.05	0.483
Pre-terminal	U	C	5263	T	ND2	Non-synonymous	A265V	0.001	0.05	0.483
Pre-terminal	U	C	5263	T	ND2	Non-synonymous	A265V	0.001	0.05	0.483
Pre-terminal	U	C	5263	T	ND2	Non-synonymous	A265V	0.001	0.05	0.483
Modern terminal	JT	C	5263	T	ND2	Non-synonymous	A265V	0.001	0.05	0.483
Modern terminal	JT	C	5263	T	ND2	Non-synonymous	A265V	0.001	0.05	0.483
Modern terminal	N1	C	5263	T	ND2	Non-synonymous	A265V	0.001	0.05	0.483
Modern terminal	N1	C	5263	T	ND2	Non-synonymous	A265V	0.001	0.05	0.483
Pre-terminal	U	A	5265	G	ND2	Non-synonymous	I266V	0.008	0.05	0.358
Modern terminal	R0	A	5268	G	ND2	Non-synonymous	I267V	0.747	0.02	0.514
Modern terminal	U	A	5268	G	ND2	Non-synonymous	I267V	0.747	0.02	0.514
Modern terminal	R0	T	5277	C	ND2	Non-synonymous	F270L	0.013	1	0.272
Modern terminal	R0	T	5277	C	ND2	Non-synonymous	F270L	0.013	1	0.272
Modern terminal	R0	T	5277	C	ND2	Non-synonymous	F270L	0.013	1	0.272
Modern terminal	R0	T	5277	C	ND2	Non-synonymous	F270L	0.013	1	0.272
Pre-terminal	R0	T	5277	C	ND2	Non-synonymous	F270L	0.013	1	0.272
Modern terminal	U	T	5277	C	ND2	Non-synonymous	F270L	0.013	1	0.272
Modern terminal	U	T	5277	C	ND2	Non-synonymous	F270L	0.013	1	0.272
Modern terminal	JT	T	5277	C	ND2	Non-synonymous	F270L	0.013	1	0.272
Modern terminal	JT	T	5277	C	ND2	Non-synonymous	F270L	0.013	1	0.272
Modern terminal	JT	T	5277	C	ND2	Non-synonymous	F270L	0.013	1	0.272
Pre-terminal	JT	T	5277	C	ND2	Non-synonymous	F270L	0.013	1	0.272
Pre-terminal	JT	T	5277	C	ND2	Non-synonymous	F270L	0.013	1	0.272
Pre-terminal	JT	T	5277	C	ND2	Non-synonymous	F270L	0.013	1	0.272
Modern terminal	N1	T	5277	C	ND2	Non-synonymous	F270L	0.013	1	0.272
Pre-terminal	N1	T	5277	C	ND2	Non-synonymous	F270L	0.013	1	0.272
Ancient terminal	U	A	5289	G	ND2	Non-synonymous	N274D	0.011	0.62	0.53
Modern terminal	JT	A	5290	G	ND2	Non-synonymous	N274S	0.107	0.12	0.57
Modern terminal	R0	G	5293	A	ND2	Non-synonymous	S275N	0	0.22	0.529
Modern terminal	R0	C	5295	T	ND2	Non-synonymous	L276F	0.005	0.05	0.329
Modern terminal	R0	C	5295	T	ND2	Non-synonymous	L276F	0.005	0.05	0.329
Modern terminal	U	C	5295	T	ND2	Non-synonymous	L276F	0.005	0.05	0.329
Modern terminal	U	C	5295	T	ND2	Non-synonymous	L276F	0.005	0.05	0.329
Pre-terminal	U	C	5295	T	ND2	Non-synonymous	L276F	0.005	0.05	0.329

Pre-terminal	JT	C	5295	T	ND2	Non-synonymous	L276F	0.005	0.05	0.329
Pre-terminal	U	A	5298	G	ND2	Non-synonymous	I277V	0.004	0.12	0.463
Modern terminal	JT	A	5298	G	ND2	Non-synonymous	I277V	0.004	0.12	0.463
Ancient terminal	R0	A	5301	G	ND2	Non-synonymous	I278V	0.007	0.29	0.29
Modern terminal	R0	A	5301	C	ND2	Non-synonymous	I278L	0	1	0.312
Pre-terminal	R0	A	5301	G	ND2	Non-synonymous	I278V	0.007	0.29	0.29
Modern terminal	U	A	5301	G	ND2	Non-synonymous	I278V	0.007	0.29	0.29
Modern terminal	U	A	5301	G	ND2	Non-synonymous	I278V	0.007	0.29	0.29
Pre-terminal	U	A	5301	G	ND2	Non-synonymous	I278V	0.007	0.29	0.29
Pre-terminal	U	A	5301	G	ND2	Non-synonymous	I278V	0.007	0.29	0.29
Modern terminal	X	A	5301	G	ND2	Non-synonymous	I278V	0.007	0.29	0.29
Modern terminal	R0	T	5302	C	ND2	Non-synonymous	I278T	0.008	0.31	0.608
Modern terminal	R0	T	5302	C	ND2	Non-synonymous	I278T	0.008	0.31	0.608
Modern terminal	R0	T	5302	C	ND2	Non-synonymous	I278T	0.008	0.31	0.608
Pre-terminal	R0	T	5302	C	ND2	Non-synonymous	I278T	0.008	0.31	0.608
Pre-terminal	R0	T	5302	C	ND2	Non-synonymous	I278T	0.008	0.31	0.608
Pre-terminal	X	T	5302	C	ND2	Non-synonymous	I278T	0.008	0.31	0.608
Ancient terminal	X	C	5304	G	ND2	Non-synonymous	P279A	1	0.8	0.803
Pre-terminal	R0	A	5307	G	ND2	Non-synonymous	T280A	0.523	0.03	0.676
Ancient terminal	U	A	5307	T	ND2	Non-synonymous	T280S	0.029	0.05	0.427
Pre-terminal	JT	A	5307	G	ND2	Non-synonymous	T280A	0.523	0.03	0.676
Pre-terminal	JT	C	5308	T	ND2	Non-synonymous	T280I	0.728	0.01	0.807
Modern terminal	R0	A	5310	G	ND2	Non-synonymous	I281V	0.007	0.35	0.333
Modern terminal	U	A	5310	G	ND2	Non-synonymous	I281V	0.007	0.35	0.333
Ancient terminal	X	A	5315	C	ND2	Non-synonymous	M282I	0.967	0.14	0.789
Ancient terminal	R0	A	5319	G	ND2	Non-synonymous	T284A	0	0.01	0.341
Modern terminal	R0	A	5319	G	ND2	Non-synonymous	T284A	0	0.01	0.341
Modern terminal	R0	A	5319	G	ND2	Non-synonymous	T284A	0	0.01	0.341
Modern terminal	R0	A	5319	G	ND2	Non-synonymous	T284A	0	0.01	0.341
Modern terminal	R0	A	5319	G	ND2	Non-synonymous	T284A	0	0.01	0.341
Pre-terminal	R0	A	5319	G	ND2	Non-synonymous	T284A	0	0.01	0.341
Modern terminal	U	A	5319	G	ND2	Non-synonymous	T284A	0	0.01	0.341
Pre-terminal	U	A	5319	G	ND2	Non-synonymous	T284A	0	0.01	0.341
Pre-terminal	U	A	5319	G	ND2	Non-synonymous	T284A	0	0.01	0.341
Pre-terminal	U	A	5319	T	ND2	Non-synonymous	T284S	0.016	0	0.22
Ancient terminal	JT	A	5319	G	ND2	Non-synonymous	T284A	0	0.01	0.341
Modern terminal	JT	A	5319	G	ND2	Non-synonymous	T284A	0	0.01	0.341
Modern terminal	JT	A	5319	G	ND2	Non-synonymous	T284A	0	0.01	0.341
Modern terminal	JT	A	5319	G	ND2	Non-synonymous	T284A	0	0.01	0.341
Modern terminal	JT	A	5319	G	ND2	Non-synonymous	T284A	0	0.01	0.341
Modern terminal	JT	A	5319	G	ND2	Non-synonymous	T284A	0	0.01	0.341
Pre-terminal	JT	A	5319	G	ND2	Non-synonymous	T284A	0	0.01	0.341
Modern terminal	N1	A	5319	G	ND2	Non-synonymous	T284A	0	0.01	0.341
Modern terminal	N1	A	5319	G	ND2	Non-synonymous	T284A	0	0.01	0.341
Modern terminal	N1	A	5319	G	ND2	Non-synonymous	T284A	0	0.01	0.341
Modern terminal	N1	A	5319	G	ND2	Non-synonymous	T284A	0	0.01	0.341
Modern terminal	JT	C	5320	T	ND2	Non-synonymous	T284I	0	1	0.219
Pre-terminal	JT	C	5320	T	ND2	Non-synonymous	T284I	0	1	0.219
Pre-terminal	R0	A	5322	C	ND2	Non-synonymous	I285L	0	1	0.328
Ancient terminal	JT	A	5322	C	ND2	Non-synonymous	I285L	0	1	0.328

Ancient terminal	JT	A	5322	G	ND2	Non-synonymous	I285V	0.002	0.64	0.224
Modern terminal	JT	A	5322	C	ND2	Non-synonymous	I285L	0	1	0.328
Modern terminal	JT	A	5322	C	ND2	Non-synonymous	I285L	0	1	0.328
Pre-terminal	JT	A	5322	C	ND2	Non-synonymous	I285L	0	1	0.328
Pre-terminal	JT	A	5322	C	ND2	Non-synonymous	I285L	0	1	0.328
Ancient terminal	R0	A	5325	T	ND2	Non-synonymous	T286S	0.213	0.17	0.257
Modern terminal	U	A	5325	G	ND2	Non-synonymous	T286A	0.06	1	0.108
Ancient terminal	X	C	5326	T	ND2	Non-synonymous	T286I	0.986	0	0.395
Modern terminal	U	C	5331	A	ND2	Non-synonymous	L288I	0.002	0.06	0.312
Ancient terminal	R0	T	5344	C	ND2	Non-synonymous	F292S	1	0	0.751
Ancient terminal	X	C	5352	T	ND2	Non-synonymous	R295C	1	0	0.542
Ancient terminal	JT	A	5367	C	ND2	Non-synonymous	T300P	0.998	0.01	0.563
Ancient terminal	U	T	5380	A	ND2	Non-synonymous	L304Q	1	0	0.732
Ancient terminal	JT	C	5386	A	ND2	Non-synonymous	P306H	1	0	0.67
Ancient terminal	U	T	5407	G	ND2	Nonsense				
Ancient terminal	U	A	5408	C	ND2	Non-synonymous	M313I	0.004	0.28	0.368
Ancient terminal	U	A	5410	C	ND2	Non-synonymous	K314T	0.087	0.31	0.556
Modern terminal	R0	T	5420	G	ND2	Non-synonymous	F317L	0	0.24	0.274
Pre-terminal	U	C	5424	T	ND2	Non-synonymous	H319Y	0	0.41	0.243
Pre-terminal	R0	A	5425	T	ND2	Non-synonymous	H319L	0.005	0.27	0.336
Modern terminal	R0	T	5426	G	ND2	Non-synonymous	H319Q	0.268	0.21	0.245
Modern terminal	U	T	5426	G	ND2	Non-synonymous	H319Q	0.268	0.21	0.245
Pre-terminal	X	A	5427	G	ND2	Non-synonymous	T320A	0.104	0.12	0.409
Modern terminal	R0	A	5436	G	ND2	Non-synonymous	T323A	0.333	0.22	0.304
Modern terminal	U	A	5436	G	ND2	Non-synonymous	T323A	0.333	0.22	0.304
Modern terminal	U	C	5437	T	ND2	Non-synonymous	T323I	0.378	0.28	0.329
Modern terminal	U	C	5437	T	ND2	Non-synonymous	T323I	0.378	0.28	0.329
Pre-terminal	N1	C	5440	T	ND2	Non-synonymous	P324L	0.997	0.8	0.129
Modern terminal	R0	T	5442	C	ND2	Non-synonymous	F325L	0.001	1	0.073
Pre-terminal	U	T	5442	C	ND2	Non-synonymous	F325L	0.001	1	0.073
Pre-terminal	U	T	5442	C	ND2	Non-synonymous	F325L	0.001	1	0.073
Pre-terminal	JT	T	5442	C	ND2	Non-synonymous	F325L	0.001	1	0.073
Modern terminal	N1	T	5442	C	ND2	Non-synonymous	F325L	0.001	1	0.073
Modern terminal	N2	T	5442	C	ND2	Non-synonymous	F325L	0.001	1	0.073
Modern terminal	R0	A	5451	C	ND2	Non-synonymous	T328P	0.002	0.56	0.147
Modern terminal	R0	A	5451	G	ND2	Non-synonymous	T328A	0.072	0.48	0.163
Pre-terminal	U	C	5452	T	ND2	Non-synonymous	T328M	0.603	0.15	0.171
Pre-terminal	U	A	5457	G	ND2	Non-synonymous	I330V	0.046	0.23	0.095
Modern terminal	N1	A	5457	G	ND2	Non-synonymous	I330V	0.046	0.23	0.095
Pre-terminal	X	A	5457	G	ND2	Non-synonymous	I330V	0.046	0.23	0.095
Modern terminal	R0	G	5460	A	ND2	Non-synonymous	A331T	0	0.41	0.112
Modern terminal	R0	G	5460	A	ND2	Non-synonymous	A331T	0	0.41	0.112
Modern terminal	R0	G	5460	A	ND2	Non-synonymous	A331T	0	0.41	0.112
Modern terminal	R0	G	5460	A	ND2	Non-synonymous	A331T	0	0.41	0.112
Modern terminal	R0	G	5460	A	ND2	Non-synonymous	A331T	0	0.41	0.112
Modern terminal	R0	G	5460	A	ND2	Non-synonymous	A331T	0	0.41	0.112
Modern terminal	R0	G	5460	A	ND2	Non-synonymous	A331T	0	0.41	0.112
Modern terminal	R0	G	5460	A	ND2	Non-synonymous	A331T	0	0.41	0.112
Modern terminal	R0	G	5460	A	ND2	Non-synonymous	A331T	0	0.41	0.112
Modern terminal	R0	G	5460	A	ND2	Non-synonymous	A331T	0	0.41	0.112

Modern terminal	R0	G	5460	A	ND2	Non-synonymous	A331T	0	0.41	0.112
Modern terminal	R0	G	5460	A	ND2	Non-synonymous	A331T	0	0.41	0.112
Modern terminal	R0	G	5460	A	ND2	Non-synonymous	A331T	0	0.41	0.112
Modern terminal	R0	G	5460	A	ND2	Non-synonymous	A331T	0	0.41	0.112
Modern terminal	R0	G	5460	A	ND2	Non-synonymous	A331T	0	0.41	0.112
Modern terminal	R0	G	5460	A	ND2	Non-synonymous	A331T	0	0.41	0.112
Modern terminal	R0	G	5460	A	ND2	Non-synonymous	A331T	0	0.41	0.112
Modern terminal	R0	G	5460	A	ND2	Non-synonymous	A331T	0	0.41	0.112
Modern terminal	R0	G	5460	A	ND2	Non-synonymous	A331T	0	0.41	0.112
Modern terminal	R0	G	5460	A	ND2	Non-synonymous	A331T	0	0.41	0.112
Modern terminal	R0	G	5460	A	ND2	Non-synonymous	A331T	0	0.41	0.112
Modern terminal	R0	G	5460	A	ND2	Non-synonymous	A331T	0	0.41	0.112
Modern terminal	R0	G	5460	A	ND2	Non-synonymous	A331T	0	0.41	0.112
Modern terminal	R0	G	5460	A	ND2	Non-synonymous	A331T	0	0.41	0.112
Modern terminal	R0	G	5460	A	ND2	Non-synonymous	A331T	0	0.41	0.112
Modern terminal	R0	G	5460	A	ND2	Non-synonymous	A331T	0	0.41	0.112
Pre-terminal	R0	G	5460	A	ND2	Non-synonymous	A331T	0	0.41	0.112
Pre-terminal	R0	G	5460	A	ND2	Non-synonymous	A331T	0	0.41	0.112
Pre-terminal	R0	G	5460	A	ND2	Non-synonymous	A331T	0	0.41	0.112
Pre-terminal	R0	G	5460	A	ND2	Non-synonymous	A331T	0	0.41	0.112
Pre-terminal	R0	G	5460	A	ND2	Non-synonymous	A331T	0	0.41	0.112
Pre-terminal	R0	G	5460	A	ND2	Non-synonymous	A331T	0	0.41	0.112
Pre-terminal	R0	G	5460	A	ND2	Non-synonymous	A331T	0	0.41	0.112
Pre-terminal	R0	G	5460	A	ND2	Non-synonymous	A331T	0	0.41	0.112
Pre-terminal	R0	G	5460	A	ND2	Non-synonymous	A331T	0	0.41	0.112
Pre-terminal	R0	G	5460	A	ND2	Non-synonymous	A331T	0	0.41	0.112
Pre-terminal	R0	G	5460	A	ND2	Non-synonymous	A331T	0	0.41	0.112
Pre-terminal	R0	G	5460	A	ND2	Non-synonymous	A331T	0	0.41	0.112
Pre-terminal	R0	G	5460	A	ND2	Non-synonymous	A331T	0	0.41	0.112
Pre-terminal	R0	G	5460	A	ND2	Non-synonymous	A331T	0	0.41	0.112
Pre-terminal	R0	G	5460	A	ND2	Non-synonymous	A331T	0	0.41	0.112
Pre-terminal	R0	G	5460	A	ND2	Non-synonymous	A331T	0	0.41	0.112
Pre-terminal	R0	G	5460	A	ND2	Non-synonymous	A331T	0	0.41	0.112
Pre-terminal	R0	G	5460	A	ND2	Non-synonymous	A331T	0	0.41	0.112
Ancient terminal	U	G	5460	A	ND2	Non-synonymous	A331T	0	0.41	0.112
Ancient terminal	U	G	5460	A	ND2	Non-synonymous	A331T	0	0.41	0.112
Ancient terminal	U	G	5460	A	ND2	Non-synonymous	A331T	0	0.41	0.112
Ancient terminal	U	G	5460	A	ND2	Non-synonymous	A331T	0	0.41	0.112
Modern terminal	U	G	5460	A	ND2	Non-synonymous	A331T	0	0.41	0.112
Modern terminal	U	G	5460	A	ND2	Non-synonymous	A331T	0	0.41	0.112
Modern terminal	U	G	5460	A	ND2	Non-synonymous	A331T	0	0.41	0.112
Modern terminal	U	G	5460	A	ND2	Non-synonymous	A331T	0	0.41	0.112
Modern terminal	U	G	5460	A	ND2	Non-synonymous	A331T	0	0.41	0.112
Modern terminal	U	G	5460	A	ND2	Non-synonymous	A331T	0	0.41	0.112
Modern terminal	U	G	5460	A	ND2	Non-synonymous	A331T	0	0.41	0.112
Modern terminal	U	G	5460	A	ND2	Non-synonymous	A331T	0	0.41	0.112
Modern terminal	U	G	5460	A	ND2	Non-synonymous	A331T	0	0.41	0.112
Modern terminal	U	G	5460	A	ND2	Non-synonymous	A331T	0	0.41	0.112
Modern terminal	U	G	5460	A	ND2	Non-synonymous	A331T	0	0.41	0.112
Modern terminal	U	G	5460	A	ND2	Non-synonymous	A331T	0	0.41	0.112

Modern terminal	U	G	5460	A	ND2	Non-synonymous	A331T	0	0.41	0.112
Modern terminal	U	G	5460	A	ND2	Non-synonymous	A331T	0	0.41	0.112
Modern terminal	U	G	5460	A	ND2	Non-synonymous	A331T	0	0.41	0.112
Modern terminal	U	G	5460	A	ND2	Non-synonymous	A331T	0	0.41	0.112
Modern terminal	U	G	5460	A	ND2	Non-synonymous	A331T	0	0.41	0.112
Modern terminal	U	G	5460	A	ND2	Non-synonymous	A331T	0	0.41	0.112
Modern terminal	U	G	5460	A	ND2	Non-synonymous	A331T	0	0.41	0.112
Modern terminal	U	G	5460	A	ND2	Non-synonymous	A331T	0	0.41	0.112
Modern terminal	U	G	5460	A	ND2	Non-synonymous	A331T	0	0.41	0.112
Modern terminal	U	G	5460	A	ND2	Non-synonymous	A331T	0	0.41	0.112
Modern terminal	U	G	5460	A	ND2	Non-synonymous	A331T	0	0.41	0.112
Modern terminal	U	G	5460	A	ND2	Non-synonymous	A331T	0	0.41	0.112
Modern terminal	U	G	5460	A	ND2	Non-synonymous	A331T	0	0.41	0.112
Modern terminal	U	G	5460	A	ND2	Non-synonymous	A331T	0	0.41	0.112
Modern terminal	U	G	5460	A	ND2	Non-synonymous	A331T	0	0.41	0.112
Modern terminal	U	G	5460	A	ND2	Non-synonymous	A331T	0	0.41	0.112
Modern terminal	U	G	5460	A	ND2	Non-synonymous	A331T	0	0.41	0.112
Modern terminal	U	G	5460	A	ND2	Non-synonymous	A331T	0	0.41	0.112
Pre-terminal	U	G	5460	A	ND2	Non-synonymous	A331T	0	0.41	0.112
Pre-terminal	U	G	5460	A	ND2	Non-synonymous	A331T	0	0.41	0.112
Pre-terminal	U	G	5460	A	ND2	Non-synonymous	A331T	0	0.41	0.112
Pre-terminal	U	G	5460	A	ND2	Non-synonymous	A331T	0	0.41	0.112
Pre-terminal	U	G	5460	A	ND2	Non-synonymous	A331T	0	0.41	0.112
Pre-terminal	U	G	5460	A	ND2	Non-synonymous	A331T	0	0.41	0.112
Pre-terminal	U	G	5460	A	ND2	Non-synonymous	A331T	0	0.41	0.112
Pre-terminal	U	G	5460	A	ND2	Non-synonymous	A331T	0	0.41	0.112
Pre-terminal	U	G	5460	A	ND2	Non-synonymous	A331T	0	0.41	0.112
Pre-terminal	U	G	5460	A	ND2	Non-synonymous	A331T	0	0.41	0.112
Pre-terminal	U	G	5460	A	ND2	Non-synonymous	A331T	0	0.41	0.112
Pre-terminal	U	G	5460	A	ND2	Non-synonymous	A331T	0	0.41	0.112
Pre-terminal	U	G	5460	A	ND2	Non-synonymous	A331T	0	0.41	0.112
Pre-terminal	U	G	5460	A	ND2	Non-synonymous	A331T	0	0.41	0.112
Pre-terminal	U	G	5460	A	ND2	Non-synonymous	A331T	0	0.41	0.112
Ancient terminal	JT	G	5460	A	ND2	Non-synonymous	A331T	0	0.41	0.112
Modern terminal	JT	G	5460	A	ND2	Non-synonymous	A331T	0	0.41	0.112
Modern terminal	JT	G	5460	A	ND2	Non-synonymous	A331T	0	0.41	0.112
Modern terminal	JT	G	5460	A	ND2	Non-synonymous	A331T	0	0.41	0.112
Modern terminal	JT	G	5460	A	ND2	Non-synonymous	A331T	0	0.41	0.112
Modern terminal	JT	G	5460	A	ND2	Non-synonymous	A331T	0	0.41	0.112
Modern terminal	JT	G	5460	A	ND2	Non-synonymous	A331T	0	0.41	0.112
Modern terminal	JT	G	5460	A	ND2	Non-synonymous	A331T	0	0.41	0.112
Modern terminal	JT	G	5460	A	ND2	Non-synonymous	A331T	0	0.41	0.112
Modern terminal	JT	G	5460	A	ND2	Non-synonymous	A331T	0	0.41	0.112
Modern terminal	JT	G	5460	A	ND2	Non-synonymous	A331T	0	0.41	0.112
Pre-terminal	JT	G	5460	A	ND2	Non-synonymous	A331T	0	0.41	0.112
Pre-terminal	JT	G	5460	A	ND2	Non-synonymous	A331T	0	0.41	0.112
Pre-terminal	JT	G	5460	A	ND2	Non-synonymous	A331T	0	0.41	0.112
Pre-terminal	JT	G	5460	A	ND2	Non-synonymous	A331T	0	0.41	0.112
Pre-terminal	JT	G	5460	A	ND2	Non-synonymous	A331T	0	0.41	0.112
Pre-terminal	JT	G	5460	A	ND2	Non-synonymous	A331T	0	0.41	0.112

Pre-terminal	JT	G	5460	A	ND2	Non-synonymous	A331T	0	0.41	0.112
Pre-terminal	JT	G	5460	A	ND2	Non-synonymous	A331T	0	0.41	0.112
Pre-terminal	JT	G	5460	A	ND2	Non-synonymous	A331T	0	0.41	0.112
Modern terminal	N1	G	5460	A	ND2	Non-synonymous	A331T	0	0.41	0.112
Modern terminal	N1	G	5460	A	ND2	Non-synonymous	A331T	0	0.41	0.112
Modern terminal	N1	G	5460	A	ND2	Non-synonymous	A331T	0	0.41	0.112
Pre-terminal	N1	G	5460	A	ND2	Non-synonymous	A331T	0	0.41	0.112
Pre-terminal	N1	G	5460	A	ND2	Non-synonymous	A331T	0	0.41	0.112
Pre-terminal	N1	G	5460	A	ND2	Non-synonymous	A331T	0	0.41	0.112
Pre-terminal	N1	G	5460	A	ND2	Non-synonymous	A331T	0	0.41	0.112
Pre-terminal	X	G	5460	A	ND2	Non-synonymous	A331T	0	0.41	0.112
Modern terminal	R0	C	5461	T	ND2	Non-synonymous	A331V	0	0.63	0.115
Modern terminal	R0	C	5461	T	ND2	Non-synonymous	A331V	0	0.63	0.115
Modern terminal	U	C	5461	T	ND2	Non-synonymous	A331V	0	0.63	0.115
Pre-terminal	JT	C	5463	T	ND2	Non-synonymous	L332F	0.386	0.02	0.361
Pre-terminal	U	A	5466	G	ND2	Non-synonymous	T333A	0.036	0.09	0.217
Modern terminal	U	C	5470	T	ND2	Non-synonymous	T334M	0.992	0.05	0.219
Modern terminal	N2	C	5470	T	ND2	Non-synonymous	T334M	0.992	0.05	0.219
Modern terminal	N2	C	5475	T	ND2	Non-synonymous	L336F	0.994	0.01	0.191
Ancient terminal	JT	A	5484	G	ND2	Non-synonymous	I339V	0.015	0.02	0.063
Modern terminal	JT	A	5484	G	ND2	Non-synonymous	I339V	0.015	0.02	0.063
Pre-terminal	JT	A	5484	G	ND2	Non-synonymous	I339V	0.015	0.02	0.063
Pre-terminal	N1	A	5484	G	ND2	Non-synonymous	I339V	0.015	0.02	0.063
Ancient terminal	N2	C	5486	G	ND2	Non-synonymous	I339M	0.996	0.18	0.078
Modern terminal	R0	T	5493	C	ND2	Non-synonymous	F342L	0.001	0.59	0.151
Modern terminal	R0	T	5493	C	ND2	Non-synonymous	F342L	0.001	0.59	0.151
Modern terminal	JT	T	5493	C	ND2	Non-synonymous	F342L	0.001	0.59	0.151
Pre-terminal	JT	T	5493	C	ND2	Non-synonymous	F342L	0.001	0.59	0.151
Modern terminal	R0	T	5494	C	ND2	Non-synonymous	F342S	0.983	0.01	0.278
Modern terminal	R0	A	5496	G	ND2	Non-synonymous	M343V	0.988	0.07	0.074
Modern terminal	R0	A	5496	G	ND2	Non-synonymous	M343V	0.988	0.07	0.074
Modern terminal	X	A	5496	G	ND2	Non-synonymous	M343V	0.988	0.07	0.074
Pre-terminal	X	A	5496	G	ND2	Non-synonymous	M343V	0.988	0.07	0.074
Ancient terminal	U	C	5499	A	ND2	Non-synonymous	L344M	0.999	0.21	0.09
Ancient terminal	U	C	5499	A	ND2	Non-synonymous	L344M	0.999	0.21	0.09
Ancient terminal	N1	T	5500	C	ND2	Non-synonymous	L344P	1	0.01	0.263
Ancient terminal	U	A	5502	G	ND2	Non-synonymous	M345V	0.966	0.3	0.059
Modern terminal	U	A	5502	G	ND2	Non-synonymous	M345V	0.966	0.3	0.059
Ancient terminal	U	T	5503	C	ND2	Non-synonymous	M345T	0.9	0.71	0.054
Modern terminal	N1	A	5504	T	ND2	Non-synonymous	M345I	0.145	0.16	0.049
Ancient terminal	R0	A	5505	G	ND2	Non-synonymous	I346V	0	0.01	0.053
Modern terminal	R0	A	5505	G	ND2	Non-synonymous	I346V	0	0.01	0.053
Ancient terminal	U	A	5505	T	ND2	Non-synonymous	I346F	0.002	0.01	0.118
Modern terminal	JT	A	5505	G	ND2	Non-synonymous	I346V	0	0.01	0.053
Pre-terminal	N1	C	5507	A	ND2	Non-synonymous	I346M	0.092	0.07	0.078
Ancient terminal	U	T	5508	A	ND2	Non-synonymous	L347M	0.974	0.21	0.067
Ancient terminal	R0	A	5510	C	ND2	Non-synonymous	L347F	0.678	0.02	0.055
Modern terminal	R0	G	5910	A	COX1	Non-synonymous	A3T	0.002	0.16	0.132
Modern terminal	U	G	5910	A	COX1	Non-synonymous	A3T	0.002	0.16	0.132
Modern terminal	R0	C	5911	T	COX1	Non-synonymous	A3V	0	0.81	0.123

Pre-terminal	R0	C	5911	T	COX1	Non-synonymous	A3V	0	0.81	0.123
Modern terminal	N1	C	5911	T	COX1	Non-synonymous	A3V	0	0.81	0.123
Ancient terminal	R0	G	5913	A	COX1	Non-synonymous	D4N	0	0.69	0.052
Modern terminal	R0	G	5913	A	COX1	Non-synonymous	D4N	0	0.69	0.052
Modern terminal	R0	G	5913	A	COX1	Non-synonymous	D4N	0	0.69	0.052
Modern terminal	R0	G	5913	A	COX1	Non-synonymous	D4N	0	0.69	0.052
Modern terminal	R0	G	5913	A	COX1	Non-synonymous	D4N	0	0.69	0.052
Modern terminal	R0	G	5913	A	COX1	Non-synonymous	D4N	0	0.69	0.052
Pre-terminal	R0	G	5913	A	COX1	Non-synonymous	D4N	0	0.69	0.052
Modern terminal	U	G	5913	A	COX1	Non-synonymous	D4N	0	0.69	0.052
Modern terminal	JT	G	5913	A	COX1	Non-synonymous	D4N	0	0.69	0.052
Modern terminal	JT	G	5913	A	COX1	Non-synonymous	D4N	0	0.69	0.052
Modern terminal	JT	G	5913	A	COX1	Non-synonymous	D4N	0	0.69	0.052
Pre-terminal	JT	G	5913	A	COX1	Non-synonymous	D4N	0	0.69	0.052
Modern terminal	N1	G	5913	A	COX1	Non-synonymous	D4N	0	0.69	0.052
Modern terminal	N1	G	5913	A	COX1	Non-synonymous	D4N	0	0.69	0.052
Modern terminal	N1	G	5913	A	COX1	Non-synonymous	D4N	0	0.69	0.052
Ancient terminal	X	T	5919	A	COX1	Nonsense				
Pre-terminal	N1	A	5959	G	COX1	Non-synonymous	Y19C	1	0	0.49
Modern terminal	U	G	5970	A	COX1	Non-synonymous	G23S	0.021	0.21	0.531
Modern terminal	R0	G	5973	A	COX1	Non-synonymous	A24T	0.004	0.27	0.41
Modern terminal	R0	G	5973	A	COX1	Non-synonymous	A24T	0.004	0.27	0.41
Ancient terminal	U	G	5973	A	COX1	Non-synonymous	A24T	0.004	0.27	0.41
Modern terminal	U	G	5973	A	COX1	Non-synonymous	A24T	0.004	0.27	0.41
Modern terminal	U	G	5973	A	COX1	Non-synonymous	A24T	0.004	0.27	0.41
Pre-terminal	JT	G	5973	A	COX1	Non-synonymous	A24T	0.004	0.27	0.41
Modern terminal	N1	G	5973	A	COX1	Non-synonymous	A24T	0.004	0.27	0.41
Modern terminal	U	T	5976	G	COX1	Non-synonymous	W25G	1	0.05	0.86
Ancient terminal	JT	A	5978	T	COX1	Non-synonymous	W25C	1	0.01	0.866
Ancient terminal	R0	G	5979	A	COX1	Non-synonymous	A26T	0.999	0.02	0.352
Ancient terminal	R0	G	5979	A	COX1	Non-synonymous	A26T	0.999	0.02	0.352
Modern terminal	R0	G	5979	A	COX1	Non-synonymous	A26T	0.999	0.02	0.352
Pre-terminal	R0	G	5979	A	COX1	Non-synonymous	A26T	0.999	0.02	0.352
Pre-terminal	R0	G	5979	A	COX1	Non-synonymous	A26T	0.999	0.02	0.352
Modern terminal	U	G	5979	A	COX1	Non-synonymous	A26T	0.999	0.02	0.352
Modern terminal	U	G	5979	A	COX1	Non-synonymous	A26T	0.999	0.02	0.352
Modern terminal	U	G	5979	A	COX1	Non-synonymous	A26T	0.999	0.02	0.352
Modern terminal	U	G	5979	A	COX1	Non-synonymous	A26T	0.999	0.02	0.352
Modern terminal	U	G	5979	A	COX1	Non-synonymous	A26T	0.999	0.02	0.352
Pre-terminal	U	G	5979	A	COX1	Non-synonymous	A26T	0.999	0.02	0.352
Pre-terminal	U	G	5979	A	COX1	Non-synonymous	A26T	0.999	0.02	0.352
Pre-terminal	JT	G	5979	A	COX1	Non-synonymous	A26T	0.999	0.02	0.352
Modern terminal	N1	G	5979	A	COX1	Non-synonymous	A26T	0.999	0.02	0.352
Modern terminal	R0	G	5985	A	COX1	Non-synonymous	V28I	0	0.21	0.089
Modern terminal	R0	G	5985	A	COX1	Non-synonymous	V28I	0	0.21	0.089
Modern terminal	R0	G	5985	A	COX1	Non-synonymous	V28I	0	0.21	0.089
Modern terminal	U	G	5985	A	COX1	Non-synonymous	V28I	0	0.21	0.089
Modern terminal	U	G	5985	A	COX1	Non-synonymous	V28I	0	0.21	0.089
Pre-terminal	U	G	5985	A	COX1	Non-synonymous	V28I	0	0.21	0.089
Modern terminal	JT	G	5985	A	COX1	Non-synonymous	V28I	0	0.21	0.089

Modern terminal	JT	G	5991	A	COX1	Non-synonymous	G30S	1	0.05	0.513
Modern terminal	U	A	5994	G	COX1	Non-synonymous	T31A	0.033	0.24	0.35
Modern terminal	R0	G	5997	A	COX1	Non-synonymous	A32T	0.999	0.28	0.411
Ancient terminal	X	C	5998	T	COX1	Non-synonymous	A32V	1	0.13	0.45
Ancient terminal	JT	C	6000	A	COX1	Non-synonymous	L33M	0.245	0.56	0.198
Modern terminal	U	C	6009	T	COX1	Non-synonymous	L36F	1	0.01	0.485
Modern terminal	R0	G	6018	A	COX1	Non-synonymous	A39T	0.004	0.38	0.139
Modern terminal	R0	G	6018	A	COX1	Non-synonymous	A39T	0.004	0.38	0.139
Pre-terminal	R0	G	6018	A	COX1	Non-synonymous	A39T	0.004	0.38	0.139
Modern terminal	U	G	6018	A	COX1	Non-synonymous	A39T	0.004	0.38	0.139
Modern terminal	U	G	6018	A	COX1	Non-synonymous	A39T	0.004	0.38	0.139
Modern terminal	JT	G	6018	A	COX1	Non-synonymous	A39T	0.004	0.38	0.139
Pre-terminal	N1	C	6034	T	COX1	Non-synonymous	P44L	1	0	0.194
Modern terminal	R0	A	6039	G	COX1	Non-synonymous	N46D	0.021	0.5	0.101
Modern terminal	R0	A	6040	G	COX1	Non-synonymous	N46S	0	1	0.045
Modern terminal	R0	A	6040	G	COX1	Non-synonymous	N46S	0	1	0.045
Modern terminal	R0	A	6040	G	COX1	Non-synonymous	N46S	0	1	0.045
Modern terminal	R0	A	6040	G	COX1	Non-synonymous	N46S	0	1	0.045
Modern terminal	R0	A	6040	G	COX1	Non-synonymous	N46S	0	1	0.045
Modern terminal	R0	A	6040	G	COX1	Non-synonymous	N46S	0	1	0.045
Modern terminal	R0	A	6040	G	COX1	Non-synonymous	N46S	0	1	0.045
Modern terminal	R0	A	6040	G	COX1	Non-synonymous	N46S	0	1	0.045
Modern terminal	R0	A	6040	G	COX1	Non-synonymous	N46S	0	1	0.045
Pre-terminal	R0	A	6040	G	COX1	Non-synonymous	N46S	0	1	0.045
Pre-terminal	R0	A	6040	G	COX1	Non-synonymous	N46S	0	1	0.045
Pre-terminal	R0	A	6040	G	COX1	Non-synonymous	N46S	0	1	0.045
Modern terminal	U	A	6040	G	COX1	Non-synonymous	N46S	0	1	0.045
Modern terminal	U	A	6040	G	COX1	Non-synonymous	N46S	0	1	0.045
Modern terminal	U	A	6040	G	COX1	Non-synonymous	N46S	0	1	0.045
Modern terminal	U	A	6040	G	COX1	Non-synonymous	N46S	0	1	0.045
Modern terminal	U	A	6040	G	COX1	Non-synonymous	N46S	0	1	0.045
Modern terminal	U	A	6040	G	COX1	Non-synonymous	N46S	0	1	0.045
Modern terminal	U	A	6040	G	COX1	Non-synonymous	N46S	0	1	0.045
Modern terminal	U	A	6040	G	COX1	Non-synonymous	N46S	0	1	0.045
Pre-terminal	U	A	6040	G	COX1	Non-synonymous	N46S	0	1	0.045
Pre-terminal	U	A	6040	G	COX1	Non-synonymous	N46S	0	1	0.045
Pre-terminal	U	A	6040	G	COX1	Non-synonymous	N46S	0	1	0.045
Ancient terminal	JT	A	6040	G	COX1	Non-synonymous	N46S	0	1	0.045
Modern terminal	JT	A	6040	G	COX1	Non-synonymous	N46S	0	1	0.045
Modern terminal	JT	A	6040	G	COX1	Non-synonymous	N46S	0	1	0.045
Modern terminal	JT	A	6040	G	COX1	Non-synonymous	N46S	0	1	0.045
Modern terminal	JT	A	6040	G	COX1	Non-synonymous	N46S	0	1	0.045
Pre-terminal	N1	A	6040	G	COX1	Non-synonymous	N46S	0	1	0.045
Ancient terminal	N2	A	6040	G	COX1	Non-synonymous	N46S	0	1	0.045
Modern terminal	N2	A	6040	G	COX1	Non-synonymous	N46S	0	1	0.045
Modern terminal	R0	C	6042	T	COX1	Non-synonymous	L47F	0.001	0.24	0.219
Ancient terminal	JT	T	6043	G	COX1	Non-synonymous	L47R	0.752	0.03	0.452
Pre-terminal	U	C	6045	A	COX1	Non-synonymous	L48M	0.062	0.23	0.116
Pre-terminal	JT	C	6045	A	COX1	Non-synonymous	L48M	0.062	0.23	0.116
Modern terminal	U	G	6048	A	COX1	Non-synonymous	G49S	0.16	0.14	0.247

Modern terminal	R0	A	6052	G	COX1	Non-synonymous	N50S	0	0.25	0.073
Modern terminal	R0	A	6052	G	COX1	Non-synonymous	N50S	0	0.25	0.073
Pre-terminal	JT	A	6052	G	COX1	Non-synonymous	N50S	0	0.25	0.073
Modern terminal	R0	G	6054	T	COX1	Non-synonymous	D51Y	1	0.21	0.34
Modern terminal	R0	G	6054	T	COX1	Non-synonymous	D51Y	1	0.21	0.34
Modern terminal	U	C	6059	G	COX1	Non-synonymous	H52Q	0.019	1	0.117
Modern terminal	U	C	6059	G	COX1	Non-synonymous	H52Q	0.019	1	0.117
Modern terminal	R0	A	6060	G	COX1	Non-synonymous	I53V	0	0.13	0.074
Modern terminal	R0	A	6060	C	COX1	Non-synonymous	I53L	0.004	1	0.179
Pre-terminal	R0	A	6060	G	COX1	Non-synonymous	I53V	0	0.13	0.074
Pre-terminal	R0	A	6060	C	COX1	Non-synonymous	I53L	0.004	1	0.179
Ancient terminal	U	A	6060	G	COX1	Non-synonymous	I53V	0	0.13	0.074
Modern terminal	U	A	6060	G	COX1	Non-synonymous	I53V	0	0.13	0.074
Pre-terminal	JT	A	6060	G	COX1	Non-synonymous	I53V	0	0.13	0.074
Pre-terminal	U	T	6061	C	COX1	Non-synonymous	I53T	0.861	0.05	0.173
Ancient terminal	R0	C	6062	A	COX1	Non-synonymous	I53M	0.945	0.1	0.148
Ancient terminal	U	T	6070	C	COX1	Non-synonymous	V56A	0.975	0.12	0.302
Modern terminal	R0	T	6076	G	COX1	Non-synonymous	V58G	1	0.02	0.456
Modern terminal	R0	G	6081	T	COX1	Non-synonymous	A60S	0	0.1	0.29
Modern terminal	R0	C	6082	G	COX1	Non-synonymous	A60G	0.228	0.28	0.344
Modern terminal	U	T	6109	C	COX1	Non-synonymous	M69T	0.002	0.01	0.518
Modern terminal	U	T	6112	C	COX1	Non-synonymous	V70A	1	0	0.523
Pre-terminal	U	T	6112	C	COX1	Non-synonymous	V70A	1	0	0.523
Modern terminal	N1	T	6112	C	COX1	Non-synonymous	V70A	1	0	0.523
Pre-terminal	U	A	6120	G	COX1	Non-synonymous	I73V	0.944	0.79	0.201
Modern terminal	R0	T	6121	C	COX1	Non-synonymous	I73T	1	0.08	0.382
Modern terminal	N1	A	6123	C	COX1	Non-synonymous	M74L	0.515	1	0.416
Modern terminal	U	A	6126	G	COX1	Non-synonymous	I75V	0.944	0.07	0.152
Modern terminal	JT	A	6126	G	COX1	Non-synonymous	I75V	0.944	0.07	0.152
Ancient terminal	JT	T	6144	C	COX1	Non-synonymous	W81R	1	0	0.756
Modern terminal	R0	G	6150	A	COX1	Non-synonymous	V83I	0	0.37	0.183
Modern terminal	R0	G	6150	A	COX1	Non-synonymous	V83I	0	0.37	0.183
Modern terminal	R0	G	6150	A	COX1	Non-synonymous	V83I	0	0.37	0.183
Modern terminal	R0	G	6150	A	COX1	Non-synonymous	V83I	0	0.37	0.183
Pre-terminal	R0	G	6150	A	COX1	Non-synonymous	V83I	0	0.37	0.183
Modern terminal	U	G	6150	A	COX1	Non-synonymous	V83I	0	0.37	0.183
Pre-terminal	U	G	6150	A	COX1	Non-synonymous	V83I	0	0.37	0.183
Pre-terminal	U	G	6150	A	COX1	Non-synonymous	V83I	0	0.37	0.183
Pre-terminal	U	G	6150	A	COX1	Non-synonymous	V83I	0	0.37	0.183
Ancient terminal	JT	G	6150	A	COX1	Non-synonymous	V83I	0	0.37	0.183
Pre-terminal	JT	G	6150	A	COX1	Non-synonymous	V83I	0	0.37	0.183
Pre-terminal	JT	G	6150	A	COX1	Non-synonymous	V83I	0	0.37	0.183
Modern terminal	R0	A	6162	G	COX1	Non-synonymous	I87V	0.913	0.24	0.12
Pre-terminal	JT	T	6176	A	COX1	Non-synonymous	D91E	0.066	0	0.365
Modern terminal	R0	A	6177	G	COX1	Non-synonymous	M92V	0.997	0.06	0.297
Ancient terminal	R0	T	6184	A	COX1	Non-synonymous	F94Y	0.957	0.07	0.323
Ancient terminal	R0	C	6189	A	COX1	Non-synonymous	R96S	0.981	0	0.515
Ancient terminal	U	C	6197	A	COX1	Non-synonymous	N98K	0.999	0	0.223
Ancient terminal	X	G	6211	A	COX1	Nonsense				
Ancient terminal	X	T	6214	C	COX1	Non-synonymous	L104P	1	0	0.448

Modern terminal	U	C	6228	T	COX1	Non-synonymous	L109F	0	0.36	0.034
Pre-terminal	R0	C	6237	A	COX1	Non-synonymous	L112M	1	0.06	0.144
Modern terminal	R0	G	6249	A	COX1	Non-synonymous	A116T	0	0.95	0.198
Modern terminal	R0	G	6249	A	COX1	Non-synonymous	A116T	0	0.95	0.198
Modern terminal	R0	G	6249	A	COX1	Non-synonymous	A116T	0	0.95	0.198
Pre-terminal	R0	G	6249	A	COX1	Non-synonymous	A116T	0	0.95	0.198
Modern terminal	U	G	6249	A	COX1	Non-synonymous	A116T	0	0.95	0.198
Modern terminal	U	G	6249	A	COX1	Non-synonymous	A116T	0	0.95	0.198
Modern terminal	U	G	6249	A	COX1	Non-synonymous	A116T	0	0.95	0.198
Modern terminal	U	G	6249	A	COX1	Non-synonymous	A116T	0	0.95	0.198
Modern terminal	JT	G	6249	A	COX1	Non-synonymous	A116T	0	0.95	0.198
Pre-terminal	JT	G	6249	A	COX1	Non-synonymous	A116T	0	0.95	0.198
Pre-terminal	JT	G	6249	A	COX1	Non-synonymous	A116T	0	0.95	0.198
Pre-terminal	N1	G	6249	A	COX1	Non-synonymous	A116T	0	0.95	0.198
Ancient terminal	R0	A	6252	G	COX1	Non-synonymous	M117V	0.002	0.19	0.319
Modern terminal	R0	A	6252	G	COX1	Non-synonymous	M117V	0.002	0.19	0.319
Modern terminal	R0	A	6252	G	COX1	Non-synonymous	M117V	0.002	0.19	0.319
Modern terminal	R0	T	6253	C	COX1	Non-synonymous	M117T	0	0.21	0.382
Modern terminal	R0	T	6253	C	COX1	Non-synonymous	M117T	0	0.21	0.382
Modern terminal	R0	T	6253	C	COX1	Non-synonymous	M117T	0	0.21	0.382
Pre-terminal	R0	T	6253	C	COX1	Non-synonymous	M117T	0	0.21	0.382
Modern terminal	U	T	6253	C	COX1	Non-synonymous	M117T	0	0.21	0.382
Modern terminal	U	T	6253	C	COX1	Non-synonymous	M117T	0	0.21	0.382
Pre-terminal	U	T	6253	C	COX1	Non-synonymous	M117T	0	0.21	0.382
Pre-terminal	U	T	6253	C	COX1	Non-synonymous	M117T	0	0.21	0.382
Modern terminal	JT	T	6253	C	COX1	Non-synonymous	M117T	0	0.21	0.382
Modern terminal	JT	T	6253	C	COX1	Non-synonymous	M117T	0	0.21	0.382
Pre-terminal	JT	T	6253	C	COX1	Non-synonymous	M117T	0	0.21	0.382
Modern terminal	R0	G	6255	A	COX1	Non-synonymous	V118M	0.088	0.05	0.343
Modern terminal	JT	A	6259	G	COX1	Non-synonymous	E119G	1	0.1	0.413
Modern terminal	JT	G	6260	T	COX1	Non-synonymous	E119D	0.986	0.21	0.348
Modern terminal	R0	G	6261	A	COX1	Non-synonymous	A120T	0.995	0.52	0.324
Modern terminal	R0	G	6261	A	COX1	Non-synonymous	A120T	0.995	0.52	0.324
Modern terminal	R0	G	6261	A	COX1	Non-synonymous	A120T	0.995	0.52	0.324
Modern terminal	R0	G	6261	A	COX1	Non-synonymous	A120T	0.995	0.52	0.324
Modern terminal	R0	G	6261	A	COX1	Non-synonymous	A120T	0.995	0.52	0.324
Modern terminal	R0	G	6261	A	COX1	Non-synonymous	A120T	0.995	0.52	0.324
Modern terminal	R0	G	6261	A	COX1	Non-synonymous	A120T	0.995	0.52	0.324
Modern terminal	R0	G	6261	A	COX1	Non-synonymous	A120T	0.995	0.52	0.324
Modern terminal	R0	G	6261	A	COX1	Non-synonymous	A120T	0.995	0.52	0.324
Pre-terminal	R0	G	6261	A	COX1	Non-synonymous	A120T	0.995	0.52	0.324
Pre-terminal	R0	G	6261	A	COX1	Non-synonymous	A120T	0.995	0.52	0.324
Pre-terminal	R0	G	6261	A	COX1	Non-synonymous	A120T	0.995	0.52	0.324
Pre-terminal	R0	G	6261	A	COX1	Non-synonymous	A120T	0.995	0.52	0.324
Pre-terminal	R0	G	6261	A	COX1	Non-synonymous	A120T	0.995	0.52	0.324
Pre-terminal	R0	G	6261	A	COX1	Non-synonymous	A120T	0.995	0.52	0.324
Pre-terminal	R0	G	6261	A	COX1	Non-synonymous	A120T	0.995	0.52	0.324
Pre-terminal	R0	G	6261	A	COX1	Non-synonymous	A120T	0.995	0.52	0.324
Pre-terminal	R0	G	6261	A	COX1	Non-synonymous	A120T	0.995	0.52	0.324
Pre-terminal	R0	G	6261	A	COX1	Non-synonymous	A120T	0.995	0.52	0.324

Ancient terminal	U	G	6261	A	COX1	Non-synonymous	A120T	0.995	0.52	0.324
Ancient terminal	U	G	6261	A	COX1	Non-synonymous	A120T	0.995	0.52	0.324
Modern terminal	U	G	6261	A	COX1	Non-synonymous	A120T	0.995	0.52	0.324
Modern terminal	U	G	6261	A	COX1	Non-synonymous	A120T	0.995	0.52	0.324
Modern terminal	U	G	6261	A	COX1	Non-synonymous	A120T	0.995	0.52	0.324
Modern terminal	U	G	6261	A	COX1	Non-synonymous	A120T	0.995	0.52	0.324
Modern terminal	U	G	6261	A	COX1	Non-synonymous	A120T	0.995	0.52	0.324
Modern terminal	U	G	6261	A	COX1	Non-synonymous	A120T	0.995	0.52	0.324
Modern terminal	U	G	6261	A	COX1	Non-synonymous	A120T	0.995	0.52	0.324
Modern terminal	U	G	6261	A	COX1	Non-synonymous	A120T	0.995	0.52	0.324
Modern terminal	U	G	6261	A	COX1	Non-synonymous	A120T	0.995	0.52	0.324
Pre-terminal	U	G	6261	A	COX1	Non-synonymous	A120T	0.995	0.52	0.324
Pre-terminal	U	G	6261	A	COX1	Non-synonymous	A120T	0.995	0.52	0.324
Modern terminal	JT	G	6261	A	COX1	Non-synonymous	A120T	0.995	0.52	0.324
Modern terminal	JT	G	6261	A	COX1	Non-synonymous	A120T	0.995	0.52	0.324
Modern terminal	JT	G	6261	A	COX1	Non-synonymous	A120T	0.995	0.52	0.324
Modern terminal	JT	G	6261	A	COX1	Non-synonymous	A120T	0.995	0.52	0.324
Modern terminal	JT	G	6261	A	COX1	Non-synonymous	A120T	0.995	0.52	0.324
Modern terminal	JT	G	6261	A	COX1	Non-synonymous	A120T	0.995	0.52	0.324
Modern terminal	JT	G	6261	A	COX1	Non-synonymous	A120T	0.995	0.52	0.324
Modern terminal	JT	G	6261	T	COX1	Non-synonymous	A120S	0.911	0.76	0.212
Pre-terminal	JT	G	6261	A	COX1	Non-synonymous	A120T	0.995	0.52	0.324
Pre-terminal	JT	G	6261	A	COX1	Non-synonymous	A120T	0.995	0.52	0.324
Pre-terminal	JT	G	6261	A	COX1	Non-synonymous	A120T	0.995	0.52	0.324
Pre-terminal	JT	G	6261	A	COX1	Non-synonymous	A120T	0.995	0.52	0.324
Pre-terminal	N1	G	6261	A	COX1	Non-synonymous	A120T	0.995	0.52	0.324
Modern terminal	X	G	6261	A	COX1	Non-synonymous	A120T	0.995	0.52	0.324
Modern terminal	N2	G	6261	A	COX1	Non-synonymous	A120T	0.995	0.52	0.324
Ancient terminal	R0	G	6267	A	COX1	Non-synonymous	A122T	0.001	0.08	0.303
Modern terminal	R0	G	6267	A	COX1	Non-synonymous	A122T	0.001	0.08	0.303
Modern terminal	R0	G	6267	A	COX1	Non-synonymous	A122T	0.001	0.08	0.303
Modern terminal	R0	G	6267	A	COX1	Non-synonymous	A122T	0.001	0.08	0.303
Modern terminal	R0	G	6267	A	COX1	Non-synonymous	A122T	0.001	0.08	0.303
Modern terminal	R0	G	6267	A	COX1	Non-synonymous	A122T	0.001	0.08	0.303
Modern terminal	R0	G	6267	A	COX1	Non-synonymous	A122T	0.001	0.08	0.303
Modern terminal	R0	G	6267	A	COX1	Non-synonymous	A122T	0.001	0.08	0.303
Modern terminal	R0	G	6267	A	COX1	Non-synonymous	A122T	0.001	0.08	0.303
Modern terminal	R0	G	6267	A	COX1	Non-synonymous	A122T	0.001	0.08	0.303
Pre-terminal	R0	G	6267	A	COX1	Non-synonymous	A122T	0.001	0.08	0.303
Pre-terminal	R0	G	6267	A	COX1	Non-synonymous	A122T	0.001	0.08	0.303
Pre-terminal	R0	G	6267	A	COX1	Non-synonymous	A122T	0.001	0.08	0.303
Pre-terminal	R0	G	6267	A	COX1	Non-synonymous	A122T	0.001	0.08	0.303
Pre-terminal	R0	G	6267	A	COX1	Non-synonymous	A122T	0.001	0.08	0.303
Modern terminal	U	G	6267	A	COX1	Non-synonymous	A122T	0.001	0.08	0.303
Modern terminal	U	G	6267	A	COX1	Non-synonymous	A122T	0.001	0.08	0.303
Modern terminal	U	G	6267	A	COX1	Non-synonymous	A122T	0.001	0.08	0.303
Modern terminal	U	G	6267	A	COX1	Non-synonymous	A122T	0.001	0.08	0.303
Modern terminal	U	G	6267	A	COX1	Non-synonymous	A122T	0.001	0.08	0.303
Modern terminal	U	G	6267	A	COX1	Non-synonymous	A122T	0.001	0.08	0.303
Modern terminal	U	G	6267	A	COX1	Non-synonymous	A122T	0.001	0.08	0.303

Modern terminal	U	G	6267	A	COX1	Non-synonymous	A122T	0.001	0.08	0.303
Modern terminal	U	G	6267	A	COX1	Non-synonymous	A122T	0.001	0.08	0.303
Pre-terminal	U	G	6267	A	COX1	Non-synonymous	A122T	0.001	0.08	0.303
Modern terminal	JT	G	6267	A	COX1	Non-synonymous	A122T	0.001	0.08	0.303
Modern terminal	JT	G	6267	A	COX1	Non-synonymous	A122T	0.001	0.08	0.303
Pre-terminal	JT	G	6267	A	COX1	Non-synonymous	A122T	0.001	0.08	0.303
Modern terminal	N1	G	6267	A	COX1	Non-synonymous	A122T	0.001	0.08	0.303
Pre-terminal	N1	G	6267	A	COX1	Non-synonymous	A122T	0.001	0.08	0.303
Pre-terminal	X	G	6267	A	COX1	Non-synonymous	A122T	0.001	0.08	0.303
Modern terminal	N2	G	6267	A	COX1	Non-synonymous	A122T	0.001	0.08	0.303
Pre-terminal	N2	G	6267	A	COX1	Non-synonymous	A122T	0.001	0.08	0.303
Pre-terminal	N2	G	6267	A	COX1	Non-synonymous	A122T	0.001	0.08	0.303
Pre-terminal	N2	G	6267	A	COX1	Non-synonymous	A122T	0.001	0.08	0.303
Modern terminal	R0	C	6268	T	COX1	Non-synonymous	A122V	0.003	0.15	0.32
Ancient terminal	N1	G	6271	C	COX1	Non-synonymous	G123A	1	0.06	0.414
Ancient terminal	N2	G	6271	T	COX1	Non-synonymous	G123V	1	0	0.566
Ancient terminal	R0	G	6285	A	COX1	Non-synonymous	V128I	0.022	0.16	0.121
Modern terminal	R0	G	6285	A	COX1	Non-synonymous	V128I	0.022	0.16	0.121
Modern terminal	R0	G	6285	A	COX1	Non-synonymous	V128I	0.022	0.16	0.121
Modern terminal	R0	G	6285	A	COX1	Non-synonymous	V128I	0.022	0.16	0.121
Modern terminal	R0	G	6285	A	COX1	Non-synonymous	V128I	0.022	0.16	0.121
Pre-terminal	R0	G	6285	A	COX1	Non-synonymous	V128I	0.022	0.16	0.121
Pre-terminal	U	G	6285	A	COX1	Non-synonymous	V128I	0.022	0.16	0.121
Modern terminal	JT	G	6285	A	COX1	Non-synonymous	V128I	0.022	0.16	0.121
Modern terminal	JT	G	6285	A	COX1	Non-synonymous	V128I	0.022	0.16	0.121
Modern terminal	JT	G	6285	A	COX1	Non-synonymous	V128I	0.022	0.16	0.121
Modern terminal	JT	G	6285	A	COX1	Non-synonymous	V128I	0.022	0.16	0.121
Modern terminal	JT	G	6285	A	COX1	Non-synonymous	V128I	0.022	0.16	0.121
Modern terminal	N2	G	6285	A	COX1	Non-synonymous	V128I	0.022	0.16	0.121
Modern terminal	N2	G	6285	A	COX1	Non-synonymous	V128I	0.022	0.16	0.121
Ancient terminal	JT	T	6286	A	COX1	Non-synonymous	V128D	1	0	0.588
Modern terminal	U	G	6300	A	COX1	Non-synonymous	A133T	0.431	0	0.159
Modern terminal	U	G	6300	T	COX1	Non-synonymous	A133S	0.001	1	0.146
Modern terminal	U	A	6307	G	COX1	Non-synonymous	N135S	0.995	0.42	0.138
Modern terminal	JT	A	6307	G	COX1	Non-synonymous	N135S	0.995	0.42	0.138
Ancient terminal	U	C	6317	A	COX1	Non-synonymous	H138Q	0.998	0.01	0.214
Modern terminal	JT	C	6318	T	COX1	Non-synonymous	P139S	0.553	1	0.165
Ancient terminal	X	G	6321	A	COX1	Nonsense				
Ancient terminal	X	G	6330	A	COX1	Non-synonymous	V143M	0.998	0.08	0.177
Ancient terminal	X	G	6333	A	COX1	Non-synonymous	D144N	0.996	0	0.254
Modern terminal	R0	A	6339	G	COX1	Non-synonymous	T146A	0.088	1	0.118
Modern terminal	R0	A	6339	G	COX1	Non-synonymous	T146A	0.088	1	0.118
Modern terminal	R0	A	6339	G	COX1	Non-synonymous	T146A	0.088	1	0.118
Modern terminal	R0	C	6340	T	COX1	Non-synonymous	T146I	0.001	0.01	0.134
Modern terminal	R0	C	6340	T	COX1	Non-synonymous	T146I	0.001	0.01	0.134
Modern terminal	R0	C	6340	T	COX1	Non-synonymous	T146I	0.001	0.01	0.134
Modern terminal	R0	C	6340	T	COX1	Non-synonymous	T146I	0.001	0.01	0.134
Modern terminal	R0	C	6340	T	COX1	Non-synonymous	T146I	0.001	0.01	0.134
Modern terminal	R0	C	6340	T	COX1	Non-synonymous	T146I	0.001	0.01	0.134
Modern terminal	R0	C	6340	T	COX1	Non-synonymous	T146I	0.001	0.01	0.134

Modern terminal	R0	C	6340	T	COX1	Non-synonymous	T146I	0.001	0.01	0.134
Modern terminal	R0	C	6340	T	COX1	Non-synonymous	T146I	0.001	0.01	0.134
Modern terminal	R0	C	6340	T	COX1	Non-synonymous	T146I	0.001	0.01	0.134
Pre-terminal	R0	C	6340	T	COX1	Non-synonymous	T146I	0.001	0.01	0.134
Ancient terminal	U	C	6340	T	COX1	Non-synonymous	T146I	0.001	0.01	0.134
Ancient terminal	U	C	6340	T	COX1	Non-synonymous	T146I	0.001	0.01	0.134
Modern terminal	U	C	6340	T	COX1	Non-synonymous	T146I	0.001	0.01	0.134
Modern terminal	U	C	6340	T	COX1	Non-synonymous	T146I	0.001	0.01	0.134
Modern terminal	U	C	6340	T	COX1	Non-synonymous	T146I	0.001	0.01	0.134
Modern terminal	U	C	6340	T	COX1	Non-synonymous	T146I	0.001	0.01	0.134
Pre-terminal	U	C	6340	T	COX1	Non-synonymous	T146I	0.001	0.01	0.134
Ancient terminal	JT	C	6340	T	COX1	Non-synonymous	T146I	0.001	0.01	0.134
Ancient terminal	JT	C	6340	T	COX1	Non-synonymous	T146I	0.001	0.01	0.134
Modern terminal	JT	C	6340	T	COX1	Non-synonymous	T146I	0.001	0.01	0.134
Modern terminal	JT	C	6340	T	COX1	Non-synonymous	T146I	0.001	0.01	0.134
Pre-terminal	JT	C	6340	T	COX1	Non-synonymous	T146I	0.001	0.01	0.134
Pre-terminal	JT	C	6340	T	COX1	Non-synonymous	T146I	0.001	0.01	0.134
Modern terminal	X	C	6340	T	COX1	Non-synonymous	T146I	0.001	0.01	0.134
Modern terminal	N2	C	6340	T	COX1	Non-synonymous	T146I	0.001	0.01	0.134
Modern terminal	N2	C	6340	T	COX1	Non-synonymous	T146I	0.001	0.01	0.134
Modern terminal	R0	T	6345	C	COX1	Non-synonymous	F148L	0.999	0.29	0.202
Modern terminal	JT	T	6345	C	COX1	Non-synonymous	F148L	0.999	0.29	0.202
Pre-terminal	JT	T	6345	C	COX1	Non-synonymous	F148L	0.999	0.29	0.202
Pre-terminal	JT	T	6345	C	COX1	Non-synonymous	F148L	0.999	0.29	0.202
Modern terminal	N1	T	6345	C	COX1	Non-synonymous	F148L	0.999	0.29	0.202
Modern terminal	N2	T	6345	C	COX1	Non-synonymous	F148L	0.999	0.29	0.202
Ancient terminal	N1	C	6357	A	COX1	Non-synonymous	L152M	1	0.03	0.09
Ancient terminal	U	G	6364	T	COX1	Non-synonymous	G154V	1	0	0.504
Modern terminal	R0	G	6366	A	COX1	Non-synonymous	V155I	0	1	0.061
Modern terminal	R0	G	6366	A	COX1	Non-synonymous	V155I	0	1	0.061
Modern terminal	R0	G	6366	A	COX1	Non-synonymous	V155I	0	1	0.061
Modern terminal	R0	G	6366	A	COX1	Non-synonymous	V155I	0	1	0.061
Modern terminal	R0	G	6366	A	COX1	Non-synonymous	V155I	0	1	0.061
Modern terminal	R0	G	6366	A	COX1	Non-synonymous	V155I	0	1	0.061
Pre-terminal	R0	G	6366	A	COX1	Non-synonymous	V155I	0	1	0.061
Pre-terminal	R0	G	6366	A	COX1	Non-synonymous	V155I	0	1	0.061
Pre-terminal	R0	G	6366	A	COX1	Non-synonymous	V155I	0	1	0.061
Pre-terminal	R0	G	6366	C	COX1	Non-synonymous	V155L	0.002	0.35	0.203
Pre-terminal	R0	G	6366	A	COX1	Non-synonymous	V155I	0	1	0.061
Pre-terminal	R0	G	6366	C	COX1	Non-synonymous	V155L	0.002	0.35	0.203
Pre-terminal	R0	G	6366	C	COX1	Non-synonymous	V155L	0.002	0.35	0.203
Modern terminal	U	G	6366	A	COX1	Non-synonymous	V155I	0	1	0.061
Modern terminal	U	G	6366	A	COX1	Non-synonymous	V155I	0	1	0.061
Modern terminal	U	G	6366	A	COX1	Non-synonymous	V155I	0	1	0.061
Modern terminal	U	G	6366	A	COX1	Non-synonymous	V155I	0	1	0.061
Modern terminal	U	G	6366	A	COX1	Non-synonymous	V155I	0	1	0.061
Modern terminal	U	G	6366	A	COX1	Non-synonymous	V155I	0	1	0.061
Modern terminal	U	G	6366	C	COX1	Non-synonymous	V155L	0.002	0.35	0.203
Pre-terminal	U	G	6366	A	COX1	Non-synonymous	V155I	0	1	0.061
Pre-terminal	U	G	6366	C	COX1	Non-synonymous	V155L	0.002	0.35	0.203

Pre-terminal	U	G	6366	C	COX1	Non-synonymous	V155L	0.002	0.35	0.203
Ancient terminal	JT	G	6366	A	COX1	Non-synonymous	V155I	0	1	0.061
Ancient terminal	JT	G	6366	A	COX1	Non-synonymous	V155I	0	1	0.061
Modern terminal	JT	G	6366	A	COX1	Non-synonymous	V155I	0	1	0.061
Modern terminal	JT	G	6366	A	COX1	Non-synonymous	V155I	0	1	0.061
Modern terminal	JT	G	6366	C	COX1	Non-synonymous	V155L	0.002	0.35	0.203
Pre-terminal	JT	G	6366	A	COX1	Non-synonymous	V155I	0	1	0.061
Pre-terminal	JT	G	6366	A	COX1	Non-synonymous	V155I	0	1	0.061
Pre-terminal	JT	G	6366	A	COX1	Non-synonymous	V155I	0	1	0.061
Modern terminal	X	G	6366	A	COX1	Non-synonymous	V155I	0	1	0.061
Modern terminal	X	G	6366	A	COX1	Non-synonymous	V155I	0	1	0.061
Modern terminal	N2	G	6366	A	COX1	Non-synonymous	V155I	0	1	0.061
Modern terminal	N2	G	6366	A	COX1	Non-synonymous	V155I	0	1	0.061
Modern terminal	R0	T	6367	C	COX1	Non-synonymous	V155A	0.916	0.46	0.244
Pre-terminal	R0	T	6367	C	COX1	Non-synonymous	V155A	0.916	0.46	0.244
Ancient terminal	U	T	6367	C	COX1	Non-synonymous	V155A	0.916	0.46	0.244
Modern terminal	U	T	6367	C	COX1	Non-synonymous	V155A	0.916	0.46	0.244
Pre-terminal	U	T	6367	C	COX1	Non-synonymous	V155A	0.916	0.46	0.244
Ancient terminal	N1	T	6367	C	COX1	Non-synonymous	V155A	0.916	0.46	0.244
Modern terminal	N1	T	6367	C	COX1	Non-synonymous	V155A	0.916	0.46	0.244
Ancient terminal	R0	A	6387	T	COX1	Non-synonymous	I162F	0.994	0	0.538
Modern terminal	U	C	6400	T	COX1	Non-synonymous	T166M	1	0	0.397
Ancient terminal	U	A	6402	T	COX1	Non-synonymous	T167S	0.997	0	0.318
Modern terminal	R0	T	6406	C	COX1	Non-synonymous	I168T	0.957	0.24	0.375
Modern terminal	R0	A	6408	G	COX1	Non-synonymous	I169V	0	0.13	0.164
Modern terminal	R0	A	6408	C	COX1	Non-synonymous	I169L	0.003	0.87	0.265
Modern terminal	N2	A	6408	G	COX1	Non-synonymous	I169V	0	0.13	0.164
Modern terminal	U	C	6410	A	COX1	Non-synonymous	I169M	0.914	0.11	0.337
Modern terminal	R0	C	6423	G	COX1	Non-synonymous	P174A	0.998	0.15	0.243
Modern terminal	R0	G	6426	A	COX1	Non-synonymous	A175T	0.995	0.03	0.288
Modern terminal	U	A	6436	C	COX1	Non-synonymous	Q178P	0.999	0.07	0.512
Modern terminal	U	C	6445	T	COX1	Non-synonymous	T181M	0.03	1	0.112
Modern terminal	JT	C	6445	T	COX1	Non-synonymous	T181M	0.03	1	0.112
Pre-terminal	JT	C	6445	T	COX1	Non-synonymous	T181M	0.03	1	0.112
Pre-terminal	JT	C	6445	T	COX1	Non-synonymous	T181M	0.03	1	0.112
Modern terminal	N1	C	6445	T	COX1	Non-synonymous	T181M	0.03	1	0.112
Modern terminal	R0	C	6447	T	COX1	Non-synonymous	P182S	1	0.1	0.133
Modern terminal	R0	C	6448	A	COX1	Non-synonymous	P182H	1	0	0.163
Modern terminal	R0	G	6456	A	COX1	Non-synonymous	V185I	0	0.01	0.094
Modern terminal	R0	G	6456	A	COX1	Non-synonymous	V185I	0	0.01	0.094
Pre-terminal	R0	G	6456	A	COX1	Non-synonymous	V185I	0	0.01	0.094
Pre-terminal	R0	G	6456	A	COX1	Non-synonymous	V185I	0	0.01	0.094
Modern terminal	R0	G	6465	A	COX1	Non-synonymous	V188I	0	0.59	0.05
Modern terminal	R0	G	6465	A	COX1	Non-synonymous	V188I	0	0.59	0.05
Modern terminal	R0	G	6465	A	COX1	Non-synonymous	V188I	0	0.59	0.05
Modern terminal	R0	G	6465	A	COX1	Non-synonymous	V188I	0	0.59	0.05
Pre-terminal	R0	G	6465	A	COX1	Non-synonymous	V188I	0	0.59	0.05
Pre-terminal	R0	G	6465	A	COX1	Non-synonymous	V188I	0	0.59	0.05
Modern terminal	U	G	6465	A	COX1	Non-synonymous	V188I	0	0.59	0.05
Modern terminal	U	G	6465	C	COX1	Non-synonymous	V188L	0.008	0.06	0.111

Pre-terminal	U	G	6465	A	COX1	Non-synonymous	V188I	0	0.59	0.05
Modern terminal	JT	G	6465	A	COX1	Non-synonymous	V188I	0	0.59	0.05
Modern terminal	N1	G	6465	A	COX1	Non-synonymous	V188I	0	0.59	0.05
Modern terminal	R0	A	6471	G	COX1	Non-synonymous	I190V	0.002	0.75	0.052
Modern terminal	R0	G	6480	A	COX1	Non-synonymous	V193I	0	0.79	0.045
Modern terminal	R0	G	6480	A	COX1	Non-synonymous	V193I	0	0.79	0.045
Modern terminal	R0	G	6480	A	COX1	Non-synonymous	V193I	0	0.79	0.045
Modern terminal	R0	G	6480	A	COX1	Non-synonymous	V193I	0	0.79	0.045
Modern terminal	R0	G	6480	A	COX1	Non-synonymous	V193I	0	0.79	0.045
Modern terminal	R0	G	6480	A	COX1	Non-synonymous	V193I	0	0.79	0.045
Modern terminal	R0	G	6480	A	COX1	Non-synonymous	V193I	0	0.79	0.045
Pre-terminal	R0	G	6480	A	COX1	Non-synonymous	V193I	0	0.79	0.045
Modern terminal	U	G	6480	A	COX1	Non-synonymous	V193I	0	0.79	0.045
Pre-terminal	U	G	6480	A	COX1	Non-synonymous	V193I	0	0.79	0.045
Modern terminal	JT	G	6480	A	COX1	Non-synonymous	V193I	0	0.79	0.045
Pre-terminal	JT	G	6480	A	COX1	Non-synonymous	V193I	0	0.79	0.045
Ancient terminal	N1	G	6480	A	COX1	Non-synonymous	V193I	0	0.79	0.045
Pre-terminal	N1	G	6480	A	COX1	Non-synonymous	V193I	0	0.79	0.045
Pre-terminal	R0	T	6481	C	COX1	Non-synonymous	V193A	0.975	0.04	0.132
Pre-terminal	R0	T	6481	C	COX1	Non-synonymous	V193A	0.975	0.04	0.132
Modern terminal	U	T	6481	C	COX1	Non-synonymous	V193A	0.975	0.04	0.132
Modern terminal	JT	T	6481	C	COX1	Non-synonymous	V193A	0.975	0.04	0.132
Pre-terminal	JT	T	6481	C	COX1	Non-synonymous	V193A	0.975	0.04	0.132
Pre-terminal	JT	C	6489	A	COX1	Non-synonymous	L196I	0.996	0.13	0.125
Ancient terminal	U	C	6498	A	COX1	Non-synonymous	L199I	0.996	0.04	0.141
Modern terminal	U	C	6498	A	COX1	Non-synonymous	L199I	0.996	0.04	0.141
Modern terminal	JT	T	6499	C	COX1	Non-synonymous	L199P	1	0	0.735
Modern terminal	R0	T	6505	C	COX1	Non-synonymous	V201A	1	0	0.228
Modern terminal	R0	G	6510	A	COX1	Non-synonymous	A203T	0.999	0.07	0.328
Pre-terminal	R0	G	6510	A	COX1	Non-synonymous	A203T	0.999	0.07	0.328
Ancient terminal	U	G	6510	A	COX1	Non-synonymous	A203T	0.999	0.07	0.328
Ancient terminal	U	G	6510	T	COX1	Non-synonymous	A203S	0.981	0.09	0.235
Modern terminal	U	G	6510	A	COX1	Non-synonymous	A203T	0.999	0.07	0.328
Modern terminal	U	G	6510	A	COX1	Non-synonymous	A203T	0.999	0.07	0.328
Modern terminal	JT	G	6510	A	COX1	Non-synonymous	A203T	0.999	0.07	0.328
Ancient terminal	N1	G	6510	A	COX1	Non-synonymous	A203T	0.999	0.07	0.328
Modern terminal	U	G	6516	A	COX1	Non-synonymous	G205S	0.013	0.01	0.411
Modern terminal	N1	A	6519	C	COX1	Non-synonymous	I206L	0.429	0.29	0.285
Pre-terminal	U	C	6546	T	COX1	Non-synonymous	L215F	0.001	1	0.444
Modern terminal	JT	C	6546	T	COX1	Non-synonymous	L215F	0.001	1	0.444
Ancient terminal	U	T	6558	C	COX1	Non-synonymous	F219L	0.999	0	0.451
Ancient terminal	X	T	6561	C	COX1	Non-synonymous	F220L	0.999	0	0.405
Ancient terminal	X	C	6567	G	COX1	Non-synonymous	P222A	0.998	0.06	0.248
Ancient terminal	U	C	6568	T	COX1	Non-synonymous	P222L	1	0.04	0.393
Modern terminal	R0	G	6570	T	COX1	Non-synonymous	A223S	0.003	0.36	0.182
Modern terminal	R0	G	6570	T	COX1	Non-synonymous	A223S	0.003	0.36	0.182
Pre-terminal	R0	G	6570	T	COX1	Non-synonymous	A223S	0.003	0.36	0.182
Pre-terminal	R0	G	6570	T	COX1	Non-synonymous	A223S	0.003	0.36	0.182
Modern terminal	U	G	6570	T	COX1	Non-synonymous	A223S	0.003	0.36	0.182
Modern terminal	U	G	6570	T	COX1	Non-synonymous	A223S	0.003	0.36	0.182

Modern terminal	U	G	6570	T	COX1	Non-synonymous	A223S	0.003	0.36	0.182
Pre-terminal	U	G	6570	T	COX1	Non-synonymous	A223S	0.003	0.36	0.182
Modern terminal	JT	G	6570	T	COX1	Non-synonymous	A223S	0.003	0.36	0.182
Modern terminal	JT	G	6570	T	COX1	Non-synonymous	A223S	0.003	0.36	0.182
Modern terminal	JT	G	6570	T	COX1	Non-synonymous	A223S	0.003	0.36	0.182
Pre-terminal	JT	G	6570	T	COX1	Non-synonymous	A223S	0.003	0.36	0.182
Ancient terminal	N1	G	6573	C	COX1	Non-synonymous	G224R	1	0	0.444
Modern terminal	U	G	6574	C	COX1	Non-synonymous	G224A	0.999	0.11	0.326
Ancient terminal	X	G	6579	A	COX1	Nonsense				
Ancient terminal	N1	T	6589	G	COX1	Non-synonymous	I229S	0.962	0	0.376
Ancient terminal	X	T	6594	C	COX1	Non-synonymous	Y231H	1	0	0.332
Modern terminal	R0	A	6598	G	COX1	Non-synonymous	Q232R	0.977	0	0.281
Modern terminal	R0	T	6609	C	COX1	Non-synonymous	W236R	1	0.03	0.632
Modern terminal	R0	A	6663	G	COX1	Non-synonymous	I254V	0.002	0.36	0.167
Ancient terminal	U	A	6663	G	COX1	Non-synonymous	I254V	0.002	0.36	0.167
Modern terminal	U	A	6663	G	COX1	Non-synonymous	I254V	0.002	0.36	0.167
Modern terminal	U	A	6663	G	COX1	Non-synonymous	I254V	0.002	0.36	0.167
Pre-terminal	JT	A	6663	G	COX1	Non-synonymous	I254V	0.002	0.36	0.167
Ancient terminal	U	C	6669	A	COX1	Non-synonymous	H256N	0.999	0.02	0.474
Modern terminal	U	A	6672	G	COX1	Non-synonymous	I257V	0.032	0.93	0.145
Pre-terminal	U	A	6678	G	COX1	Non-synonymous	T259A	0.001	0.4	0.307
Modern terminal	R0	T	6681	C	COX1	Non-synonymous	Y260H	0.058	0.53	0.422
Modern terminal	R0	T	6681	C	COX1	Non-synonymous	Y260H	0.058	0.53	0.422
Modern terminal	JT	T	6681	C	COX1	Non-synonymous	Y260H	0.058	0.53	0.422
Pre-terminal	JT	T	6681	C	COX1	Non-synonymous	Y260H	0.058	0.53	0.422
Modern terminal	JT	A	6685	G	COX1	Non-synonymous	Y261C	0.999	0.09	0.444
Ancient terminal	N2	G	6691	C	COX1	Non-synonymous	G263A	0.276	0.08	0.398
Ancient terminal	R0	A	6714	T	COX1	Non-synonymous	M271L	0.178	1	0.398
Modern terminal	R0	G	6717	A	COX1	Non-synonymous	G272S	1	0.14	0.607
Modern terminal	JT	G	6717	A	COX1	Non-synonymous	G272S	1	0.14	0.607
Modern terminal	N2	G	6717	A	COX1	Non-synonymous	G272S	1	0.14	0.607
Ancient terminal	JT	A	6720	G	COX1	Non-synonymous	M273V	0.995	0	0.41
Pre-terminal	R0	G	6723	A	COX1	Non-synonymous	V274I	0.01	0.5	0.388
Modern terminal	JT	G	6723	A	COX1	Non-synonymous	V274I	0.01	0.5	0.388
Ancient terminal	X	G	6723	A	COX1	Non-synonymous	V274I	0.01	0.5	0.388
Ancient terminal	R0	G	6727	A	COX1	Nonsense				
Modern terminal	JT	A	6732	T	COX1	Non-synonymous	M277L	0.178	0.06	0.566
Modern terminal	JT	T	6733	C	COX1	Non-synonymous	M277T	0.984	0	0.564
Modern terminal	R0	A	6735	G	COX1	Non-synonymous	M278V	0.012	0.42	0.435
Ancient terminal	R0	C	6739	A	COX1	Nonsense				
Modern terminal	R0	G	6745	A	COX1	Non-synonymous	G281D	1	0	0.752
Modern terminal	N2	T	6748	C	COX1	Non-synonymous	F282S	1	0.01	0.76
Modern terminal	JT	T	6756	C	COX1	Non-synonymous	F285L	0.999	0.15	0.505
Ancient terminal	U	T	6776	A	COX1	Non-synonymous	H291Q	1	0	0.38
Ancient terminal	U	A	6783	T	COX1	Non-synonymous	T294S	0.992	0	0.203
Ancient terminal	U	C	6820	T	COX1	Non-synonymous	T306I	0.998	0.02	0.491
Modern terminal	X	C	6820	T	COX1	Non-synonymous	T306I	0.998	0.02	0.491
Ancient terminal	U	G	6825	T	COX1	Non-synonymous	A308S	0.011	0.07	0.424
Modern terminal	X	C	6847	T	COX1	Non-synonymous	P315L	1	0	0.558
Ancient terminal	R0	G	6852	A	COX1	Non-synonymous	G317S	1	0.04	0.453

Modern terminal	R0	G	6852	A	COX1	Non-synonymous	G317S	1	0.04	0.453
Modern terminal	U	G	6852	A	COX1	Non-synonymous	G317S	1	0.04	0.453
Ancient terminal	JT	G	6852	A	COX1	Non-synonymous	G317S	1	0.04	0.453
Modern terminal	JT	G	6852	A	COX1	Non-synonymous	G317S	1	0.04	0.453
Modern terminal	JT	G	6852	A	COX1	Non-synonymous	G317S	1	0.04	0.453
Modern terminal	X	G	6852	A	COX1	Non-synonymous	G317S	1	0.04	0.453
Ancient terminal	N2	G	6855	A	COX1	Non-synonymous	V318I	0.591	1	0.168
Ancient terminal	R0	G	6876	A	COX1	Non-synonymous	A325T	0.995	0.02	0.18
Ancient terminal	R0	T	6883	A	COX1	Non-synonymous	L327H	1	0	0.494
Modern terminal	R0	A	6891	G	COX1	Non-synonymous	S330G	0	1	0.047
Pre-terminal	R0	A	6891	G	COX1	Non-synonymous	S330G	0	1	0.047
Ancient terminal	U	A	6891	G	COX1	Non-synonymous	S330G	0	1	0.047
Modern terminal	JT	A	6891	G	COX1	Non-synonymous	S330G	0	1	0.047
Ancient terminal	R0	A	6895	G	COX1	Non-synonymous	N331S	0.277	1	0.212
Modern terminal	U	A	6895	G	COX1	Non-synonymous	N331S	0.277	1	0.212
Modern terminal	U	A	6895	G	COX1	Non-synonymous	N331S	0.277	1	0.212
Modern terminal	JT	T	6898	C	COX1	Non-synonymous	M332T	0.001	0.01	0.142
Ancient terminal	R0	C	6907	A	COX1	Non-synonymous	S335Y	0.942	0.02	0.514
Modern terminal	U	C	6910	T	COX1	Non-synonymous	A336V	0.051	0.75	0.137
Pre-terminal	JT	G	6912	C	COX1	Non-synonymous	A337P	0.999	1	0.528
Ancient terminal	R0	G	6915	A	COX1	Non-synonymous	V338M	0	1	0.058
Modern terminal	R0	G	6915	A	COX1	Non-synonymous	V338M	0	1	0.058
Modern terminal	R0	G	6915	A	COX1	Non-synonymous	V338M	0	1	0.058
Pre-terminal	R0	G	6915	A	COX1	Non-synonymous	V338M	0	1	0.058
Modern terminal	U	G	6915	A	COX1	Non-synonymous	V338M	0	1	0.058
Modern terminal	U	G	6915	A	COX1	Non-synonymous	V338M	0	1	0.058
Pre-terminal	U	G	6915	A	COX1	Non-synonymous	V338M	0	1	0.058
Pre-terminal	U	G	6915	A	COX1	Non-synonymous	V338M	0	1	0.058
Pre-terminal	U	G	6915	A	COX1	Non-synonymous	V338M	0	1	0.058
Pre-terminal	U	G	6915	A	COX1	Non-synonymous	V338M	0	1	0.058
Pre-terminal	JT	G	6915	A	COX1	Non-synonymous	V338M	0	1	0.058
Modern terminal	X	G	6915	A	COX1	Non-synonymous	V338M	0	1	0.058
Modern terminal	JT	T	6916	C	COX1	Non-synonymous	V338A	0.018	0.08	0.234
Ancient terminal	N1	T	6934	C	COX1	Non-synonymous	F344S	1	0.01	0.671
Ancient terminal	R0	C	6949	T	COX1	Non-synonymous	T349I	0.999	0	0.385
Ancient terminal	R0	G	6951	A	COX1	Non-synonymous	V350M	0.029	0.2	0.202
Pre-terminal	R0	G	6951	A	COX1	Non-synonymous	V350M	0.029	0.2	0.202
Pre-terminal	R0	G	6951	A	COX1	Non-synonymous	V350M	0.029	0.2	0.202
Modern terminal	U	G	6951	A	COX1	Non-synonymous	V350M	0.029	0.2	0.202
Modern terminal	JT	G	6951	A	COX1	Non-synonymous	V350M	0.029	0.2	0.202
Modern terminal	X	G	6951	A	COX1	Non-synonymous	V350M	0.029	0.2	0.202
Modern terminal	X	G	6957	T	COX1	Non-synonymous	G352C	1	0	0.607
Pre-terminal	U	T	6973	C	COX1	Non-synonymous	V357A	0.975	0.11	0.288
Ancient terminal	JT	A	6994	G	COX1	Non-synonymous	D364G	0.743	0	0.456
Ancient terminal	R0	G	7008	A	COX1	Non-synonymous	D369N	0.999	0	0.242
Ancient terminal	JT	C	7016	G	COX1	Nonsense				
Ancient terminal	N1	C	7019	A	COX1	Nonsense				
Modern terminal	U	G	7041	A	COX1	Non-synonymous	V380I	0.876	0	0.139
Modern terminal	U	G	7041	A	COX1	Non-synonymous	V380I	0.876	0	0.139
Pre-terminal	JT	G	7041	A	COX1	Non-synonymous	V380I	0.876	0	0.139

Modern terminal	JT	T	7042	C	COX1	Non-synonymous	V380A	1	0	0.289
Ancient terminal	R0	T	7051	C	COX1	Non-synonymous	M383T	0.997	0	0.35
Modern terminal	R0	T	7051	C	COX1	Non-synonymous	M383T	0.997	0	0.35
Modern terminal	R0	T	7051	C	COX1	Non-synonymous	M383T	0.997	0	0.35
Modern terminal	U	T	7051	C	COX1	Non-synonymous	M383T	0.997	0	0.35
Modern terminal	U	T	7051	C	COX1	Non-synonymous	M383T	0.997	0	0.35
Modern terminal	JT	T	7051	C	COX1	Non-synonymous	M383T	0.997	0	0.35
Modern terminal	JT	T	7051	C	COX1	Non-synonymous	M383T	0.997	0	0.35
Modern terminal	N1	T	7051	C	COX1	Non-synonymous	M383T	0.997	0	0.35
Modern terminal	N2	T	7051	C	COX1	Non-synonymous	M383T	0.997	0	0.35
Modern terminal	U	T	7060	C	COX1	Non-synonymous	V386A	1	0.06	0.403
Modern terminal	R0	C	7070	G	COX1	Non-synonymous	I389M	1	0.08	0.345
Modern terminal	N1	G	7075	C	COX1	Non-synonymous	G391A	1	1	0.498
Pre-terminal	U	G	7077	A	COX1	Non-synonymous	G392S	1	0.02	0.482
Ancient terminal	N2	G	7077	T	COX1	Non-synonymous	G392C	1	0	0.673
Modern terminal	R0	T	7080	C	COX1	Non-synonymous	F393L	1	0.16	0.601
Modern terminal	R0	T	7080	C	COX1	Non-synonymous	F393L	1	0.16	0.601
Modern terminal	R0	T	7080	C	COX1	Non-synonymous	F393L	1	0.16	0.601
Modern terminal	R0	T	7080	C	COX1	Non-synonymous	F393L	1	0.16	0.601
Modern terminal	R0	T	7080	C	COX1	Non-synonymous	F393L	1	0.16	0.601
Modern terminal	R0	T	7080	C	COX1	Non-synonymous	F393L	1	0.16	0.601
Modern terminal	R0	T	7080	C	COX1	Non-synonymous	F393L	1	0.16	0.601
Modern terminal	R0	T	7080	C	COX1	Non-synonymous	F393L	1	0.16	0.601
Pre-terminal	R0	T	7080	C	COX1	Non-synonymous	F393L	1	0.16	0.601
Pre-terminal	R0	T	7080	C	COX1	Non-synonymous	F393L	1	0.16	0.601
Pre-terminal	R0	T	7080	C	COX1	Non-synonymous	F393L	1	0.16	0.601
Ancient terminal	U	T	7080	C	COX1	Non-synonymous	F393L	1	0.16	0.601
Modern terminal	U	T	7080	C	COX1	Non-synonymous	F393L	1	0.16	0.601
Modern terminal	U	T	7080	C	COX1	Non-synonymous	F393L	1	0.16	0.601
Modern terminal	U	T	7080	C	COX1	Non-synonymous	F393L	1	0.16	0.601
Modern terminal	U	T	7080	C	COX1	Non-synonymous	F393L	1	0.16	0.601
Pre-terminal	U	T	7080	C	COX1	Non-synonymous	F393L	1	0.16	0.601
Pre-terminal	U	T	7080	C	COX1	Non-synonymous	F393L	1	0.16	0.601
Pre-terminal	U	T	7080	C	COX1	Non-synonymous	F393L	1	0.16	0.601
Pre-terminal	U	T	7080	C	COX1	Non-synonymous	F393L	1	0.16	0.601
Modern terminal	JT	T	7080	C	COX1	Non-synonymous	F393L	1	0.16	0.601
Modern terminal	JT	T	7080	C	COX1	Non-synonymous	F393L	1	0.16	0.601
Pre-terminal	JT	T	7080	C	COX1	Non-synonymous	F393L	1	0.16	0.601
Pre-terminal	JT	T	7080	C	COX1	Non-synonymous	F393L	1	0.16	0.601
Pre-terminal	N2	T	7080	C	COX1	Non-synonymous	F393L	1	0.16	0.601
Pre-terminal	N2	T	7080	C	COX1	Non-synonymous	F393L	1	0.16	0.601
Modern terminal	R0	A	7083	G	COX1	Non-synonymous	I394V	0.001	0.29	0.089
Modern terminal	R0	A	7083	G	COX1	Non-synonymous	I394V	0.001	0.29	0.089
Modern terminal	R0	A	7083	G	COX1	Non-synonymous	I394V	0.001	0.29	0.089
Pre-terminal	R0	A	7083	G	COX1	Non-synonymous	I394V	0.001	0.29	0.089
Pre-terminal	R0	A	7083	G	COX1	Non-synonymous	I394V	0.001	0.29	0.089
Ancient terminal	R0	T	7084	C	COX1	Non-synonymous	I394T	0.804	0.24	0.355
Modern terminal	N1	T	7092	C	COX1	Non-synonymous	F397L	1	0.16	0.517
Pre-terminal	R0	C	7114	T	COX1	Non-synonymous	T404I	0.998	0.41	0.138
Modern terminal	R0	G	7119	A	COX1	Non-synonymous	D406N	0	1	0.068

Pre-terminal	R0	G	7119	A	COX1	Non-synonymous	D406N	0	1	0.068
Modern terminal	U	G	7119	A	COX1	Non-synonymous	D406N	0	1	0.068
Modern terminal	U	G	7119	A	COX1	Non-synonymous	D406N	0	1	0.068
Modern terminal	U	G	7119	A	COX1	Non-synonymous	D406N	0	1	0.068
Modern terminal	JT	G	7119	A	COX1	Non-synonymous	D406N	0	1	0.068
Modern terminal	JT	G	7119	A	COX1	Non-synonymous	D406N	0	1	0.068
Modern terminal	JT	G	7119	A	COX1	Non-synonymous	D406N	0	1	0.068
Modern terminal	X	G	7119	A	COX1	Non-synonymous	D406N	0	1	0.068
Modern terminal	R0	A	7120	G	COX1	Non-synonymous	D406G	0.11	0.07	0.307
Modern terminal	U	A	7120	G	COX1	Non-synonymous	D406G	0.11	0.07	0.307
Modern terminal	R0	A	7125	G	COX1	Non-synonymous	T408A	0	0.51	0.196
Modern terminal	U	A	7129	G	COX1	Non-synonymous	Y409C	0.009	0.02	0.171
Modern terminal	R0	C	7132	T	COX1	Non-synonymous	A410V	0.23	0.08	0.24
Modern terminal	R0	A	7146	G	COX1	Non-synonymous	T415A	0.005	0.22	0.238
Modern terminal	U	A	7146	G	COX1	Non-synonymous	T415A	0.005	0.22	0.238
Modern terminal	U	A	7146	G	COX1	Non-synonymous	T415A	0.005	0.22	0.238
Modern terminal	R0	A	7149	G	COX1	Non-synonymous	I416V	0	0.47	0.119
Pre-terminal	R0	A	7149	G	COX1	Non-synonymous	I416V	0	0.47	0.119
Modern terminal	U	A	7149	G	COX1	Non-synonymous	I416V	0	0.47	0.119
Modern terminal	JT	A	7149	G	COX1	Non-synonymous	I416V	0	0.47	0.119
Pre-terminal	JT	A	7149	G	COX1	Non-synonymous	I416V	0	0.47	0.119
Modern terminal	R0	T	7150	C	COX1	Non-synonymous	I416T	0.679	0.3	0.33
Pre-terminal	JT	T	7150	C	COX1	Non-synonymous	I416T	0.679	0.3	0.33
Pre-terminal	R0	T	7153	C	COX1	Non-synonymous	M417T	0.001	0.54	0.313
Pre-terminal	R0	T	7153	C	COX1	Non-synonymous	M417T	0.001	0.54	0.313
Ancient terminal	U	T	7153	C	COX1	Non-synonymous	M417T	0.001	0.54	0.313
Modern terminal	U	T	7153	C	COX1	Non-synonymous	M417T	0.001	0.54	0.313
Pre-terminal	U	T	7153	C	COX1	Non-synonymous	M417T	0.001	0.54	0.313
Pre-terminal	U	T	7153	C	COX1	Non-synonymous	M417T	0.001	0.54	0.313
Modern terminal	JT	T	7153	C	COX1	Non-synonymous	M417T	0.001	0.54	0.313
Modern terminal	R0	A	7158	G	COX1	Non-synonymous	I419V	0	0.41	0.038
Pre-terminal	R0	A	7158	G	COX1	Non-synonymous	I419V	0	0.41	0.038
Pre-terminal	R0	A	7158	G	COX1	Non-synonymous	I419V	0	0.41	0.038
Ancient terminal	U	A	7158	G	COX1	Non-synonymous	I419V	0	0.41	0.038
Modern terminal	U	A	7158	G	COX1	Non-synonymous	I419V	0	0.41	0.038
Modern terminal	U	A	7158	G	COX1	Non-synonymous	I419V	0	0.41	0.038
Modern terminal	U	A	7158	G	COX1	Non-synonymous	I419V	0	0.41	0.038
Modern terminal	U	A	7158	G	COX1	Non-synonymous	I419V	0	0.41	0.038
Modern terminal	JT	A	7158	G	COX1	Non-synonymous	I419V	0	0.41	0.038
Modern terminal	N1	A	7158	T	COX1	Non-synonymous	I419F	0.107	0.16	0.304
Modern terminal	R0	T	7159	C	COX1	Non-synonymous	I419T	0.007	0.06	0.219
Modern terminal	R0	T	7159	C	COX1	Non-synonymous	I419T	0.007	0.06	0.219
Modern terminal	U	T	7165	C	COX1	Non-synonymous	V421A	1	0.08	0.257
Modern terminal	N2	A	7167	G	COX1	Non-synonymous	N422D	0.999	0	0.236
Ancient terminal	JT	C	7182	A	COX1	Non-synonymous	P427T	1	0	0.353
Modern terminal	R0	T	7191	C	COX1	Non-synonymous	F430L	1	0.03	0.371
Modern terminal	R0	T	7191	C	COX1	Non-synonymous	F430L	1	0.03	0.371
Modern terminal	R0	T	7191	C	COX1	Non-synonymous	F430L	1	0.03	0.371
Modern terminal	R0	T	7191	C	COX1	Non-synonymous	F430L	1	0.03	0.371
Modern terminal	R0	C	7194	T	COX1	Non-synonymous	L431F	1	0	0.181

Modern terminal	JT	T	7230	C	COX1	Non-synonymous	Y443H	1	0	0.42
Ancient terminal	U	T	7242	A	COX1	Non-synonymous	Y447N	1	0	0.615
Ancient terminal	R0	A	7245	G	COX1	Non-synonymous	T448A	0.995	1	0.22
Modern terminal	R0	A	7245	G	COX1	Non-synonymous	T448A	0.995	1	0.22
Modern terminal	R0	A	7245	G	COX1	Non-synonymous	T448A	0.995	1	0.22
Modern terminal	R0	A	7245	G	COX1	Non-synonymous	T448A	0.995	1	0.22
Modern terminal	R0	A	7245	G	COX1	Non-synonymous	T448A	0.995	1	0.22
Pre-terminal	R0	A	7245	G	COX1	Non-synonymous	T448A	0.995	1	0.22
Modern terminal	U	A	7245	G	COX1	Non-synonymous	T448A	0.995	1	0.22
Modern terminal	U	A	7245	G	COX1	Non-synonymous	T448A	0.995	1	0.22
Modern terminal	U	A	7245	G	COX1	Non-synonymous	T448A	0.995	1	0.22
Modern terminal	U	A	7245	G	COX1	Non-synonymous	T448A	0.995	1	0.22
Modern terminal	U	A	7245	G	COX1	Non-synonymous	T448A	0.995	1	0.22
Pre-terminal	U	A	7245	G	COX1	Non-synonymous	T448A	0.995	1	0.22
Modern terminal	JT	A	7245	G	COX1	Non-synonymous	T448A	0.995	1	0.22
Pre-terminal	JT	A	7245	G	COX1	Non-synonymous	T448A	0.995	1	0.22
Pre-terminal	JT	A	7245	G	COX1	Non-synonymous	T448A	0.995	1	0.22
Pre-terminal	N1	A	7245	G	COX1	Non-synonymous	T448A	0.995	1	0.22
Pre-terminal	X	A	7245	G	COX1	Non-synonymous	T448A	0.995	1	0.22
Modern terminal	R0	A	7248	G	COX1	Non-synonymous	T449A	0.001	0.51	0.25
Pre-terminal	R0	C	7249	T	COX1	Non-synonymous	T449M	0.014	0.22	0.173
Modern terminal	U	A	7257	G	COX1	Non-synonymous	I452V	0.001	0.71	0.064
Modern terminal	JT	A	7257	G	COX1	Non-synonymous	I452V	0.001	0.71	0.064
Pre-terminal	N1	A	7257	G	COX1	Non-synonymous	I452V	0.001	0.71	0.064
Modern terminal	R0	T	7258	C	COX1	Non-synonymous	I452T	0.026	0.69	0.071
Modern terminal	R0	T	7258	C	COX1	Non-synonymous	I452T	0.026	0.69	0.071
Modern terminal	R0	T	7258	C	COX1	Non-synonymous	I452T	0.026	0.69	0.071
Modern terminal	R0	T	7258	C	COX1	Non-synonymous	I452T	0.026	0.69	0.071
Modern terminal	R0	T	7258	C	COX1	Non-synonymous	I452T	0.026	0.69	0.071
Modern terminal	R0	T	7258	C	COX1	Non-synonymous	I452T	0.026	0.69	0.071
Modern terminal	R0	T	7258	C	COX1	Non-synonymous	I452T	0.026	0.69	0.071
Pre-terminal	R0	T	7258	C	COX1	Non-synonymous	I452T	0.026	0.69	0.071
Modern terminal	U	T	7258	C	COX1	Non-synonymous	I452T	0.026	0.69	0.071
Modern terminal	U	T	7258	C	COX1	Non-synonymous	I452T	0.026	0.69	0.071
Modern terminal	U	T	7258	C	COX1	Non-synonymous	I452T	0.026	0.69	0.071
Modern terminal	U	T	7258	C	COX1	Non-synonymous	I452T	0.026	0.69	0.071
Modern terminal	JT	T	7258	C	COX1	Non-synonymous	I452T	0.026	0.69	0.071
Pre-terminal	JT	T	7258	C	COX1	Non-synonymous	I452T	0.026	0.69	0.071
Modern terminal	N1	T	7258	C	COX1	Non-synonymous	I452T	0.026	0.69	0.071
Modern terminal	R0	G	7269	A	COX1	Non-synonymous	V456M	0.002	0.22	0.072
Modern terminal	R0	G	7269	A	COX1	Non-synonymous	V456M	0.002	0.22	0.072
Modern terminal	R0	G	7269	A	COX1	Non-synonymous	V456M	0.002	0.22	0.072
Modern terminal	R0	G	7269	A	COX1	Non-synonymous	V456M	0.002	0.22	0.072
Modern terminal	R0	G	7269	A	COX1	Non-synonymous	V456M	0.002	0.22	0.072
Modern terminal	R0	G	7269	A	COX1	Non-synonymous	V456M	0.002	0.22	0.072
Pre-terminal	R0	G	7269	A	COX1	Non-synonymous	V456M	0.002	0.22	0.072
Pre-terminal	R0	G	7269	A	COX1	Non-synonymous	V456M	0.002	0.22	0.072
Pre-terminal	R0	G	7269	A	COX1	Non-synonymous	V456M	0.002	0.22	0.072
Pre-terminal	R0	G	7269	A	COX1	Non-synonymous	V456M	0.002	0.22	0.072
Ancient terminal	U	G	7269	A	COX1	Non-synonymous	V456M	0.002	0.22	0.072

Ancient terminal	U	G	7269	A	COX1	Non-synonymous	V456M	0.002	0.22	0.072
Ancient terminal	U	G	7269	A	COX1	Non-synonymous	V456M	0.002	0.22	0.072
Modern terminal	U	G	7269	A	COX1	Non-synonymous	V456M	0.002	0.22	0.072
Modern terminal	U	G	7269	A	COX1	Non-synonymous	V456M	0.002	0.22	0.072
Pre-terminal	U	G	7269	A	COX1	Non-synonymous	V456M	0.002	0.22	0.072
Modern terminal	JT	G	7269	A	COX1	Non-synonymous	V456M	0.002	0.22	0.072
Modern terminal	JT	G	7269	A	COX1	Non-synonymous	V456M	0.002	0.22	0.072
Modern terminal	JT	G	7269	A	COX1	Non-synonymous	V456M	0.002	0.22	0.072
Modern terminal	JT	G	7269	A	COX1	Non-synonymous	V456M	0.002	0.22	0.072
Modern terminal	JT	G	7269	A	COX1	Non-synonymous	V456M	0.002	0.22	0.072
Modern terminal	JT	G	7269	A	COX1	Non-synonymous	V456M	0.002	0.22	0.072
Pre-terminal	JT	G	7269	A	COX1	Non-synonymous	V456M	0.002	0.22	0.072
Pre-terminal	JT	G	7269	A	COX1	Non-synonymous	V456M	0.002	0.22	0.072
Pre-terminal	JT	G	7269	A	COX1	Non-synonymous	V456M	0.002	0.22	0.072
Pre-terminal	JT	G	7269	A	COX1	Non-synonymous	V456M	0.002	0.22	0.072
Pre-terminal	JT	G	7269	A	COX1	Non-synonymous	V456M	0.002	0.22	0.072
Pre-terminal	JT	G	7269	A	COX1	Non-synonymous	V456M	0.002	0.22	0.072
Pre-terminal	JT	G	7269	A	COX1	Non-synonymous	V456M	0.002	0.22	0.072
Modern terminal	N1	G	7269	A	COX1	Non-synonymous	V456M	0.002	0.22	0.072
Pre-terminal	N2	G	7269	A	COX1	Non-synonymous	V456M	0.002	0.22	0.072
Pre-terminal	N2	G	7269	A	COX1	Non-synonymous	V456M	0.002	0.22	0.072
Pre-terminal	N2	G	7269	A	COX1	Non-synonymous	V456M	0.002	0.22	0.072
Modern terminal	R0	T	7270	C	COX1	Non-synonymous	V456A	0.613	0.01	0.19
Modern terminal	R0	T	7270	C	COX1	Non-synonymous	V456A	0.613	0.01	0.19
Modern terminal	R0	T	7270	C	COX1	Non-synonymous	V456A	0.613	0.01	0.19
Modern terminal	R0	T	7270	C	COX1	Non-synonymous	V456A	0.613	0.01	0.19
Modern terminal	R0	T	7270	C	COX1	Non-synonymous	V456A	0.613	0.01	0.19
Modern terminal	R0	T	7270	C	COX1	Non-synonymous	V456A	0.613	0.01	0.19
Modern terminal	R0	T	7270	C	COX1	Non-synonymous	V456A	0.613	0.01	0.19
Modern terminal	R0	T	7270	C	COX1	Non-synonymous	V456A	0.613	0.01	0.19
Pre-terminal	R0	T	7270	C	COX1	Non-synonymous	V456A	0.613	0.01	0.19
Pre-terminal	R0	T	7270	C	COX1	Non-synonymous	V456A	0.613	0.01	0.19
Pre-terminal	R0	T	7270	C	COX1	Non-synonymous	V456A	0.613	0.01	0.19
Ancient terminal	U	T	7270	C	COX1	Non-synonymous	V456A	0.613	0.01	0.19
Modern terminal	U	T	7270	C	COX1	Non-synonymous	V456A	0.613	0.01	0.19
Modern terminal	U	T	7270	C	COX1	Non-synonymous	V456A	0.613	0.01	0.19
Modern terminal	U	T	7270	C	COX1	Non-synonymous	V456A	0.613	0.01	0.19
Pre-terminal	U	T	7270	C	COX1	Non-synonymous	V456A	0.613	0.01	0.19
Pre-terminal	U	T	7270	C	COX1	Non-synonymous	V456A	0.613	0.01	0.19
Modern terminal	JT	T	7270	C	COX1	Non-synonymous	V456A	0.613	0.01	0.19
Modern terminal	JT	T	7270	C	COX1	Non-synonymous	V456A	0.613	0.01	0.19
Modern terminal	JT	T	7270	C	COX1	Non-synonymous	V456A	0.613	0.01	0.19
Modern terminal	JT	T	7270	C	COX1	Non-synonymous	V456A	0.613	0.01	0.19
Modern terminal	JT	T	7270	C	COX1	Non-synonymous	V456A	0.613	0.01	0.19
Modern terminal	JT	T	7270	C	COX1	Non-synonymous	V456A	0.613	0.01	0.19
Pre-terminal	JT	T	7270	C	COX1	Non-synonymous	V456A	0.613	0.01	0.19
Modern terminal	R0	T	7278	C	COX1	Non-synonymous	F459L	0.007	0.64	0.466
Modern terminal	R0	T	7278	C	COX1	Non-synonymous	F459L	0.007	0.64	0.466
Modern terminal	R0	T	7278	C	COX1	Non-synonymous	F459L	0.007	0.64	0.466
Pre-terminal	R0	T	7278	C	COX1	Non-synonymous	F459L	0.007	0.64	0.466

Pre-terminal	R0	T	7278	C	COX1	Non-synonymous	F459L	0.007	0.64	0.466
Pre-terminal	R0	T	7278	C	COX1	Non-synonymous	F459L	0.007	0.64	0.466
Ancient terminal	U	T	7278	C	COX1	Non-synonymous	F459L	0.007	0.64	0.466
Modern terminal	U	T	7278	C	COX1	Non-synonymous	F459L	0.007	0.64	0.466
Pre-terminal	U	T	7278	C	COX1	Non-synonymous	F459L	0.007	0.64	0.466
Pre-terminal	U	T	7278	C	COX1	Non-synonymous	F459L	0.007	0.64	0.466
Modern terminal	JT	T	7278	C	COX1	Non-synonymous	F459L	0.007	0.64	0.466
Ancient terminal	N1	T	7278	C	COX1	Non-synonymous	F459L	0.007	0.64	0.466
Ancient terminal	N1	T	7278	C	COX1	Non-synonymous	F459L	0.007	0.64	0.466
Ancient terminal	N1	T	7278	C	COX1	Non-synonymous	F459L	0.007	0.64	0.466
Modern terminal	X	T	7278	C	COX1	Non-synonymous	F459L	0.007	0.64	0.466
Modern terminal	N2	T	7278	C	COX1	Non-synonymous	F459L	0.007	0.64	0.466
Modern terminal	N2	T	7278	C	COX1	Non-synonymous	F459L	0.007	0.64	0.466
Modern terminal	R0	C	7280	A	COX1	Non-synonymous	F459L	0.007	0.64	0.466
Pre-terminal	R0	C	7280	A	COX1	Non-synonymous	F459L	0.007	0.64	0.466
Ancient terminal	R0	A	7299	G	COX1	Non-synonymous	M466V	0.001	0.26	0.391
Ancient terminal	R0	A	7299	G	COX1	Non-synonymous	M466V	0.001	0.26	0.391
Modern terminal	R0	A	7299	G	COX1	Non-synonymous	M466V	0.001	0.26	0.391
Modern terminal	R0	A	7299	G	COX1	Non-synonymous	M466V	0.001	0.26	0.391
Modern terminal	R0	A	7299	G	COX1	Non-synonymous	M466V	0.001	0.26	0.391
Pre-terminal	R0	A	7299	G	COX1	Non-synonymous	M466V	0.001	0.26	0.391
Pre-terminal	R0	A	7299	G	COX1	Non-synonymous	M466V	0.001	0.26	0.391
Pre-terminal	R0	A	7299	G	COX1	Non-synonymous	M466V	0.001	0.26	0.391
Pre-terminal	R0	A	7299	G	COX1	Non-synonymous	M466V	0.001	0.26	0.391
Pre-terminal	R0	A	7299	G	COX1	Non-synonymous	M466V	0.001	0.26	0.391
Ancient terminal	U	A	7299	G	COX1	Non-synonymous	M466V	0.001	0.26	0.391
Modern terminal	U	A	7299	G	COX1	Non-synonymous	M466V	0.001	0.26	0.391
Modern terminal	U	A	7299	G	COX1	Non-synonymous	M466V	0.001	0.26	0.391
Modern terminal	U	A	7299	G	COX1	Non-synonymous	M466V	0.001	0.26	0.391
Modern terminal	U	A	7299	G	COX1	Non-synonymous	M466V	0.001	0.26	0.391
Modern terminal	U	A	7299	G	COX1	Non-synonymous	M466V	0.001	0.26	0.391
Modern terminal	U	A	7299	G	COX1	Non-synonymous	M466V	0.001	0.26	0.391
Pre-terminal	U	A	7299	G	COX1	Non-synonymous	M466V	0.001	0.26	0.391
Pre-terminal	U	A	7299	G	COX1	Non-synonymous	M466V	0.001	0.26	0.391
Modern terminal	JT	A	7299	G	COX1	Non-synonymous	M466V	0.001	0.26	0.391
Modern terminal	JT	A	7299	G	COX1	Non-synonymous	M466V	0.001	0.26	0.391
Pre-terminal	JT	A	7299	G	COX1	Non-synonymous	M466V	0.001	0.26	0.391
Modern terminal	N1	A	7299	G	COX1	Non-synonymous	M466V	0.001	0.26	0.391
Modern terminal	N1	A	7299	G	COX1	Non-synonymous	M466V	0.001	0.26	0.391
Pre-terminal	X	A	7299	G	COX1	Non-synonymous	M466V	0.001	0.26	0.391
Modern terminal	N2	A	7299	G	COX1	Non-synonymous	M466V	0.001	0.26	0.391
Ancient terminal	JT	T	7300	C	COX1	Non-synonymous	M466T	0.013	0.16	0.418
Ancient terminal	R0	A	7301	T	COX1	Non-synonymous	M466I	0	0.97	0.226
Ancient terminal	U	T	7303	C	COX1	Non-synonymous	L467S	1	0.04	0.694
Ancient terminal	U	A	7305	T	COX1	Non-synonymous	M468L	0	0.22	0.365
Modern terminal	R0	A	7308	G	COX1	Non-synonymous	I469V	0	0.49	0.113
Modern terminal	JT	A	7308	G	COX1	Non-synonymous	I469V	0	0.49	0.113
Modern terminal	R0	T	7309	C	COX1	Non-synonymous	I469T	0.097	0.08	0.33
Pre-terminal	R0	T	7309	C	COX1	Non-synonymous	I469T	0.097	0.08	0.33
Pre-terminal	R0	T	7309	C	COX1	Non-synonymous	I469T	0.097	0.08	0.33

Ancient terminal	U	A	7317	G	COX1	Non-synonymous	I472V	0.005	0.77	0.157
Modern terminal	U	A	7317	G	COX1	Non-synonymous	I472V	0.005	0.77	0.157
Pre-terminal	U	A	7317	G	COX1	Non-synonymous	I472V	0.005	0.77	0.157
Ancient terminal	JT	G	7326	A	COX1	Non-synonymous	A475T	0.99	0.51	0.35
Ancient terminal	N1	G	7326	A	COX1	Non-synonymous	A475T	0.99	0.51	0.35
Modern terminal	U	T	7329	C	COX1	Non-synonymous	F476L	0.002	0.93	0.424
Ancient terminal	R0	G	7332	A	COX1	Non-synonymous	A477T	0.031	0.72	0.252
Ancient terminal	JT	G	7332	T	COX1	Non-synonymous	A477S	0	0.83	0.216
Ancient terminal	R0	T	7348	C	COX1	Non-synonymous	V482A	0.002	0.31	0.326
Modern terminal	R0	A	7353	G	COX1	Non-synonymous	M484V	0	0.5	0.195
Pre-terminal	R0	A	7353	G	COX1	Non-synonymous	M484V	0	0.5	0.195
Ancient terminal	U	T	7354	C	COX1	Non-synonymous	M484T	0	0.4	0.143
Modern terminal	JT	T	7354	C	COX1	Non-synonymous	M484T	0	0.4	0.143
Modern terminal	R0	G	7356	A	COX1	Non-synonymous	V485M	0	0.27	0.175
Pre-terminal	R0	G	7356	A	COX1	Non-synonymous	V485M	0	0.27	0.175
Pre-terminal	R0	G	7356	A	COX1	Non-synonymous	V485M	0	0.27	0.175
Modern terminal	U	G	7356	A	COX1	Non-synonymous	V485M	0	0.27	0.175
Modern terminal	U	G	7356	A	COX1	Non-synonymous	V485M	0	0.27	0.175
Modern terminal	U	G	7356	A	COX1	Non-synonymous	V485M	0	0.27	0.175
Pre-terminal	U	G	7356	A	COX1	Non-synonymous	V485M	0	0.27	0.175
Modern terminal	JT	G	7356	A	COX1	Non-synonymous	V485M	0	0.27	0.175
Modern terminal	R0	G	7362	A	COX1	Non-synonymous	E487K	0	0.92	0.23
Ancient terminal	U	G	7362	T	COX1	Nonsense				
Pre-terminal	N1	G	7362	A	COX1	Non-synonymous	E487K	0	0.92	0.23
Modern terminal	R0	C	7365	T	COX1	Non-synonymous	P488S	0	0.83	0.121
Modern terminal	R0	C	7365	T	COX1	Non-synonymous	P488S	0	0.83	0.121
Pre-terminal	JT	C	7365	T	COX1	Non-synonymous	P488S	0	0.83	0.121
Modern terminal	U	C	7366	T	COX1	Non-synonymous	P488L	0	0.45	0.15
Modern terminal	U	T	7368	A	COX1	Non-synonymous	S489T	0	0.39	0.097
Modern terminal	R0	A	7371	G	COX1	Non-synonymous	M490V	0.003	0.35	0.083
Modern terminal	R0	T	7372	C	COX1	Non-synonymous	M490T	0	0.99	0.041
Pre-terminal	R0	T	7372	C	COX1	Non-synonymous	M490T	0	0.99	0.041
Pre-terminal	R0	T	7372	C	COX1	Non-synonymous	M490T	0	0.99	0.041
Modern terminal	U	T	7372	C	COX1	Non-synonymous	M490T	0	0.99	0.041
Modern terminal	JT	T	7372	C	COX1	Non-synonymous	M490T	0	0.99	0.041
Modern terminal	JT	T	7372	C	COX1	Non-synonymous	M490T	0	0.99	0.041
Pre-terminal	JT	T	7372	C	COX1	Non-synonymous	M490T	0	0.99	0.041
Modern terminal	U	A	7374	G	COX1	Non-synonymous	N491D	0.259	0.13	0.174
Modern terminal	JT	A	7374	G	COX1	Non-synonymous	N491D	0.259	0.13	0.174
Modern terminal	X	A	7374	G	COX1	Non-synonymous	N491D	0.259	0.13	0.174
Modern terminal	R0	A	7375	G	COX1	Non-synonymous	N491S	0.001	0.93	0.109
Modern terminal	R0	T	7389	C	COX1	Non-synonymous	Y496H	0	0.64	0.071
Modern terminal	R0	T	7389	C	COX1	Non-synonymous	Y496H	0	0.64	0.071
Modern terminal	R0	T	7389	C	COX1	Non-synonymous	Y496H	0	0.64	0.071
Modern terminal	R0	T	7389	C	COX1	Non-synonymous	Y496H	0	0.64	0.071
Modern terminal	R0	T	7389	C	COX1	Non-synonymous	Y496H	0	0.64	0.071
Ancient terminal	U	T	7389	C	COX1	Non-synonymous	Y496H	0	0.64	0.071
Modern terminal	U	T	7389	C	COX1	Non-synonymous	Y496H	0	0.64	0.071
Modern terminal	U	T	7389	C	COX1	Non-synonymous	Y496H	0	0.64	0.071
Modern terminal	U	T	7389	C	COX1	Non-synonymous	Y496H	0	0.64	0.071

Pre-terminal	U	T	7389	C	COX1	Non-synonymous	Y496H	0	0.64	0.071
Ancient terminal	JT	T	7389	C	COX1	Non-synonymous	Y496H	0	0.64	0.071
Modern terminal	JT	T	7389	C	COX1	Non-synonymous	Y496H	0	0.64	0.071
Pre-terminal	N1	T	7389	C	COX1	Non-synonymous	Y496H	0	0.64	0.071
Pre-terminal	X	T	7389	C	COX1	Non-synonymous	Y496H	0	0.64	0.071
Ancient terminal	JT	A	7390	T	COX1	Non-synonymous	Y496F	0.02	0.67	0.102
Ancient terminal	U	C	7398	G	COX1	Non-synonymous	P499A	0.998	0.01	0.142
Modern terminal	R0	C	7399	T	COX1	Non-synonymous	P499L	1	0	0.211
Ancient terminal	JT	C	7399	G	COX1	Non-synonymous	P499R	1	0	0.27
Ancient terminal	R0	C	7401	G	COX1	Non-synonymous	P500A	0.998	0.06	0.137
Modern terminal	R0	C	7401	T	COX1	Non-synonymous	P500S	0.999	0.03	0.174
Ancient terminal	U	C	7402	A	COX1	Non-synonymous	P500Q	0.999	0	0.209
Ancient terminal	X	C	7402	A	COX1	Non-synonymous	P500Q	0.999	0	0.209
Ancient terminal	R0	C	7404	T	COX1	Non-synonymous	P501S	0.365	0.45	0.158
Ancient terminal	U	T	7407	G	COX1	Non-synonymous	Y502D	0.98	0.2	0.467
Modern terminal	U	T	7407	C	COX1	Non-synonymous	Y502H	0.013	0.53	0.141
Modern terminal	JT	T	7407	C	COX1	Non-synonymous	Y502H	0.013	0.53	0.141
Ancient terminal	N1	T	7407	C	COX1	Non-synonymous	Y502H	0.013	0.53	0.141
Modern terminal	U	A	7408	C	COX1	Non-synonymous	Y502S	0.934	0.37	0.282
Modern terminal	R0	G	7419	A	COX1	Non-synonymous	E506K	0.217	0.26	0.116
Modern terminal	R0	G	7419	A	COX1	Non-synonymous	E506K	0.217	0.26	0.116
Modern terminal	R0	G	7419	A	COX1	Non-synonymous	E506K	0.217	0.26	0.116
Modern terminal	R0	G	7419	A	COX1	Non-synonymous	E506K	0.217	0.26	0.116
Modern terminal	R0	G	7419	A	COX1	Non-synonymous	E506K	0.217	0.26	0.116
Pre-terminal	R0	G	7419	A	COX1	Non-synonymous	E506K	0.217	0.26	0.116
Pre-terminal	U	G	7419	A	COX1	Non-synonymous	E506K	0.217	0.26	0.116
Pre-terminal	N2	G	7419	A	COX1	Non-synonymous	E506K	0.217	0.26	0.116
Modern terminal	U	T	7429	C	COX1	Non-synonymous	V509A	0	0.61	0.083
Ancient terminal	JT	T	7429	A	COX1	Non-synonymous	V509E	0.125	0.06	0.238
Modern terminal	JT	A	7432	T	COX1	Non-synonymous	Y510F	0	1	0.069
Ancient terminal	R0	T	7435	A	COX1	Non-synonymous	M511K	0.001	0.01	0.203
Ancient terminal	R0	T	7435	A	COX1	Non-synonymous	M511K	0.001	0.01	0.203
Ancient terminal	R0	T	7440	A	COX1	Non-synonymous	S513T	0	0.76	0.078
Pre-terminal	JT	T	7440	G	COX1	Non-synonymous	S513A	0	0.5	0.091
Modern terminal	N2	T	7440	C	COX1	Non-synonymous	S513P	0.003	0.1	0.199
Modern terminal	N1	C	7441	T	COX1	Non-synonymous	S513F	0.07	0.02	0.143
Ancient terminal	R0	G	7589	T	COX2	Non-synonymous	A2S	0.215	0.11	0.169
Modern terminal	R0	C	7592	T	COX2	Non-synonymous	H3Y	0.994	1	0.065
Modern terminal	R0	G	7595	A	COX2	Non-synonymous	A4T	0	0.7	0.093
Ancient terminal	N1	G	7595	A	COX2	Non-synonymous	A4T	0	0.7	0.093
Ancient terminal	R0	G	7598	A	COX2	Non-synonymous	A5T	0	0.4	0.09
Modern terminal	R0	G	7598	A	COX2	Non-synonymous	A5T	0	0.4	0.09
Modern terminal	R0	G	7598	A	COX2	Non-synonymous	A5T	0	0.4	0.09
Modern terminal	R0	G	7598	A	COX2	Non-synonymous	A5T	0	0.4	0.09
Modern terminal	R0	G	7598	A	COX2	Non-synonymous	A5T	0	0.4	0.09
Modern terminal	R0	G	7598	A	COX2	Non-synonymous	A5T	0	0.4	0.09
Modern terminal	R0	G	7598	A	COX2	Non-synonymous	A5T	0	0.4	0.09
Modern terminal	R0	G	7598	A	COX2	Non-synonymous	A5T	0	0.4	0.09
Modern terminal	R0	G	7598	A	COX2	Non-synonymous	A5T	0	0.4	0.09
Modern terminal	R0	G	7598	A	COX2	Non-synonymous	A5T	0	0.4	0.09

Modern terminal	R0	G	7598	A	COX2	Non-synonymous	A5T	0	0.4	0.09
Modern terminal	R0	G	7598	A	COX2	Non-synonymous	A5T	0	0.4	0.09
Modern terminal	R0	G	7598	A	COX2	Non-synonymous	A5T	0	0.4	0.09
Modern terminal	R0	G	7598	A	COX2	Non-synonymous	A5T	0	0.4	0.09
Pre-terminal	R0	G	7598	A	COX2	Non-synonymous	A5T	0	0.4	0.09
Pre-terminal	R0	G	7598	A	COX2	Non-synonymous	A5T	0	0.4	0.09
Pre-terminal	R0	G	7598	A	COX2	Non-synonymous	A5T	0	0.4	0.09
Pre-terminal	R0	G	7598	A	COX2	Non-synonymous	A5T	0	0.4	0.09
Pre-terminal	R0	G	7598	A	COX2	Non-synonymous	A5T	0	0.4	0.09
Modern terminal	U	G	7598	A	COX2	Non-synonymous	A5T	0	0.4	0.09
Modern terminal	U	G	7598	A	COX2	Non-synonymous	A5T	0	0.4	0.09
Modern terminal	U	G	7598	A	COX2	Non-synonymous	A5T	0	0.4	0.09
Modern terminal	U	G	7598	A	COX2	Non-synonymous	A5T	0	0.4	0.09
Modern terminal	U	G	7598	A	COX2	Non-synonymous	A5T	0	0.4	0.09
Modern terminal	U	G	7598	A	COX2	Non-synonymous	A5T	0	0.4	0.09
Modern terminal	U	G	7598	A	COX2	Non-synonymous	A5T	0	0.4	0.09
Modern terminal	U	G	7598	A	COX2	Non-synonymous	A5T	0	0.4	0.09
Modern terminal	U	G	7598	A	COX2	Non-synonymous	A5T	0	0.4	0.09
Modern terminal	U	G	7598	A	COX2	Non-synonymous	A5T	0	0.4	0.09
Pre-terminal	U	G	7598	A	COX2	Non-synonymous	A5T	0	0.4	0.09
Modern terminal	JT	G	7598	A	COX2	Non-synonymous	A5T	0	0.4	0.09
Modern terminal	JT	G	7598	A	COX2	Non-synonymous	A5T	0	0.4	0.09
Modern terminal	JT	G	7598	A	COX2	Non-synonymous	A5T	0	0.4	0.09
Modern terminal	JT	G	7598	A	COX2	Non-synonymous	A5T	0	0.4	0.09
Pre-terminal	JT	G	7598	A	COX2	Non-synonymous	A5T	0	0.4	0.09
Modern terminal	N1	G	7598	A	COX2	Non-synonymous	A5T	0	0.4	0.09
Pre-terminal	X	G	7598	A	COX2	Non-synonymous	A5T	0	0.4	0.09
Modern terminal	N2	G	7598	A	COX2	Non-synonymous	A5T	0	0.4	0.09
Ancient terminal	R0	A	7602	T	COX2	Non-synonymous	Q6L	0.991	0	0.369
Pre-terminal	R0	G	7604	A	COX2	Non-synonymous	V7M	0.003	0.04	0.079
Modern terminal	JT	G	7604	A	COX2	Non-synonymous	V7M	0.003	0.04	0.079
Modern terminal	JT	G	7604	A	COX2	Non-synonymous	V7M	0.003	0.04	0.079
Modern terminal	JT	G	7604	A	COX2	Non-synonymous	V7M	0.003	0.04	0.079
Modern terminal	JT	G	7604	A	COX2	Non-synonymous	V7M	0.003	0.04	0.079
Modern terminal	N1	G	7604	A	COX2	Non-synonymous	V7M	0.003	0.04	0.079
Modern terminal	R0	G	7607	A	COX2	Non-synonymous	G8S	0.036	0.05	0.412
Modern terminal	R0	G	7607	A	COX2	Non-synonymous	G8S	0.036	0.05	0.412
Pre-terminal	R0	G	7607	A	COX2	Non-synonymous	G8S	0.036	0.05	0.412
Pre-terminal	U	G	7607	A	COX2	Non-synonymous	G8S	0.036	0.05	0.412
Ancient terminal	N1	T	7611	C	COX2	Non-synonymous	L9P	1	0	0.48
Modern terminal	R0	T	7632	C	COX2	Non-synonymous	I16T	0.98	0.05	0.327
Pre-terminal	R0	T	7632	C	COX2	Non-synonymous	I16T	0.98	0.05	0.327
Modern terminal	U	T	7632	C	COX2	Non-synonymous	I16T	0.98	0.05	0.327
Modern terminal	U	T	7632	C	COX2	Non-synonymous	I16T	0.98	0.05	0.327
Pre-terminal	JT	T	7632	C	COX2	Non-synonymous	I16T	0.98	0.05	0.327
Modern terminal	U	G	7637	A	COX2	Non-synonymous	E18K	0.995	0	0.407
Ancient terminal	U	G	7642	T	COX2	Non-synonymous	E19D	0.995	0.11	0.274
Modern terminal	R0	A	7646	G	COX2	Non-synonymous	I21V	0	0.3	0.076
Ancient terminal	U	T	7647	C	COX2	Non-synonymous	I21T	0.718	0.3	0.133
Modern terminal	JT	T	7647	C	COX2	Non-synonymous	I21T	0.718	0.3	0.133

Modern terminal	JT	T	7647	C	COX2	Non-synonymous	I21T	0.718	0.3	0.133
Ancient terminal	R0	T	7653	C	COX2	Non-synonymous	F23S	1	0	0.664
Pre-terminal	U	C	7661	T	COX2	Non-synonymous	H26Y	0	0.04	0.384
Modern terminal	JT	C	7661	T	COX2	Non-synonymous	H26Y	0	0.04	0.384
Ancient terminal	R0	G	7664	A	COX2	Non-synonymous	A27T	0.001	1	0.049
Modern terminal	R0	G	7664	A	COX2	Non-synonymous	A27T	0.001	1	0.049
Modern terminal	R0	G	7664	A	COX2	Non-synonymous	A27T	0.001	1	0.049
Modern terminal	R0	G	7664	A	COX2	Non-synonymous	A27T	0.001	1	0.049
Pre-terminal	R0	G	7664	A	COX2	Non-synonymous	A27T	0.001	1	0.049
Pre-terminal	R0	G	7664	A	COX2	Non-synonymous	A27T	0.001	1	0.049
Pre-terminal	R0	G	7664	A	COX2	Non-synonymous	A27T	0.001	1	0.049
Pre-terminal	R0	G	7664	A	COX2	Non-synonymous	A27T	0.001	1	0.049
Modern terminal	U	G	7664	A	COX2	Non-synonymous	A27T	0.001	1	0.049
Modern terminal	U	G	7664	A	COX2	Non-synonymous	A27T	0.001	1	0.049
Modern terminal	JT	G	7664	A	COX2	Non-synonymous	A27T	0.001	1	0.049
Modern terminal	JT	C	7665	T	COX2	Non-synonymous	A27V	0.003	0.21	0.142
Modern terminal	U	A	7673	G	COX2	Non-synonymous	I30V	0.014	0.21	0.103
Modern terminal	U	A	7673	G	COX2	Non-synonymous	I30V	0.014	0.21	0.103
Pre-terminal	U	A	7673	G	COX2	Non-synonymous	I30V	0.014	0.21	0.103
Modern terminal	JT	A	7673	G	COX2	Non-synonymous	I30V	0.014	0.21	0.103
Modern terminal	JT	A	7673	G	COX2	Non-synonymous	I30V	0.014	0.21	0.103
Modern terminal	X	A	7673	G	COX2	Non-synonymous	I30V	0.014	0.21	0.103
Modern terminal	N2	A	7673	G	COX2	Non-synonymous	I30V	0.014	0.21	0.103
Modern terminal	R0	T	7674	C	COX2	Non-synonymous	I30T	0.016	0.02	0.194
Modern terminal	R0	T	7674	C	COX2	Non-synonymous	I30T	0.016	0.02	0.194
Pre-terminal	R0	T	7674	C	COX2	Non-synonymous	I30T	0.016	0.02	0.194
Ancient terminal	U	T	7674	C	COX2	Non-synonymous	I30T	0.016	0.02	0.194
Modern terminal	U	T	7674	C	COX2	Non-synonymous	I30T	0.016	0.02	0.194
Modern terminal	U	T	7674	C	COX2	Non-synonymous	I30T	0.016	0.02	0.194
Modern terminal	JT	T	7674	C	COX2	Non-synonymous	I30T	0.016	0.02	0.194
Modern terminal	JT	T	7674	C	COX2	Non-synonymous	I30T	0.016	0.02	0.194
Pre-terminal	N2	T	7674	C	COX2	Non-synonymous	I30T	0.016	0.02	0.194
Ancient terminal	R0	A	7676	T	COX2	Non-synonymous	I31F	0.378	0	0.199
Ancient terminal	R0	T	7679	C	COX2	Non-synonymous	F32L	0.084	0.31	0.291
Modern terminal	R0	T	7679	C	COX2	Non-synonymous	F32L	0.084	0.31	0.291
Modern terminal	R0	T	7679	C	COX2	Non-synonymous	F32L	0.084	0.31	0.291
Ancient terminal	U	T	7679	C	COX2	Non-synonymous	F32L	0.084	0.31	0.291
Pre-terminal	JT	T	7679	C	COX2	Non-synonymous	F32L	0.084	0.31	0.291
Modern terminal	R0	T	7680	C	COX2	Non-synonymous	F32S	0.01	0.08	0.533
Modern terminal	U	T	7680	C	COX2	Non-synonymous	F32S	0.01	0.08	0.533
Modern terminal	JT	T	7680	C	COX2	Non-synonymous	F32S	0.01	0.08	0.533
Modern terminal	R0	C	7681	A	COX2	Non-synonymous	F32L	0.084	0.31	0.291
Pre-terminal	U	C	7681	A	COX2	Non-synonymous	F32L	0.084	0.31	0.291
Modern terminal	R0	A	7685	G	COX2	Non-synonymous	I34V	0.008	0.03	0.134
Modern terminal	R0	A	7685	G	COX2	Non-synonymous	I34V	0.008	0.03	0.134
Modern terminal	JT	A	7685	G	COX2	Non-synonymous	I34V	0.008	0.03	0.134
Modern terminal	N1	A	7685	G	COX2	Non-synonymous	I34V	0.008	0.03	0.134
Modern terminal	R0	G	7689	C	COX2	Non-synonymous	C35S	0.002	1	0.056
Modern terminal	R0	T	7691	C	COX2	Non-synonymous	F36L	0.012	0.63	0.124
Pre-terminal	R0	T	7691	C	COX2	Non-synonymous	F36L	0.012	0.63	0.124

Pre-terminal	R0	T	7691	C	COX2	Non-synonymous	F36L	0.012	0.63	0.124
Modern terminal	JT	T	7691	C	COX2	Non-synonymous	F36L	0.012	0.63	0.124
Pre-terminal	JT	T	7691	C	COX2	Non-synonymous	F36L	0.012	0.63	0.124
Modern terminal	R0	T	7692	C	COX2	Non-synonymous	F36S	0.995	0.31	0.083
Modern terminal	U	T	7692	C	COX2	Non-synonymous	F36S	0.995	0.31	0.083
Pre-terminal	R0	C	7693	A	COX2	Non-synonymous	F36L	0.012	0.63	0.124
Modern terminal	R0	G	7697	A	COX2	Non-synonymous	V38I	0	0.07	0.184
Pre-terminal	R0	G	7697	A	COX2	Non-synonymous	V38I	0	0.07	0.184
Modern terminal	U	G	7697	A	COX2	Non-synonymous	V38I	0	0.07	0.184
Pre-terminal	JT	G	7697	A	COX2	Non-synonymous	V38I	0	0.07	0.184
Modern terminal	X	G	7697	A	COX2	Non-synonymous	V38I	0	0.07	0.184
Modern terminal	R0	T	7698	C	COX2	Non-synonymous	V38A	0.975	0	0.391
Modern terminal	R0	T	7701	C	COX2	Non-synonymous	L39P	1	0.24	0.841
Modern terminal	R0	G	7706	A	COX2	Non-synonymous	A41T	0.391	0.03	0.084
Modern terminal	R0	G	7706	A	COX2	Non-synonymous	A41T	0.391	0.03	0.084
Modern terminal	U	G	7706	A	COX2	Non-synonymous	A41T	0.391	0.03	0.084
Modern terminal	N2	G	7706	A	COX2	Non-synonymous	A41T	0.391	0.03	0.084
Modern terminal	R0	C	7707	T	COX2	Non-synonymous	A41V	0.002	0.19	0.112
Modern terminal	R0	T	7712	C	COX2	Non-synonymous	F43L	0.005	0.68	0.105
Modern terminal	U	A	7718	G	COX2	Non-synonymous	T45A	0.22	0.24	0.22
Modern terminal	JT	A	7718	G	COX2	Non-synonymous	T45A	0.22	0.24	0.22
Modern terminal	N1	A	7718	G	COX2	Non-synonymous	T45A	0.22	0.24	0.22
Ancient terminal	U	C	7719	A	COX2	Non-synonymous	T45K	0.578	0	0.287
Modern terminal	U	C	7721	T	COX2	Non-synonymous	L46F	0.999	0.19	0.296
Modern terminal	JT	A	7730	G	COX2	Non-synonymous	K49E	0.255	0.12	0.29
Modern terminal	U	A	7740	G	COX2	Non-synonymous	N52S	0.004	0.05	0.138
Modern terminal	R0	A	7746	G	COX2	Non-synonymous	N54S	0.001	0.69	0.101
Pre-terminal	R0	A	7746	G	COX2	Non-synonymous	N54S	0.001	0.69	0.101
Modern terminal	U	A	7746	G	COX2	Non-synonymous	N54S	0.001	0.69	0.101
Modern terminal	U	A	7746	G	COX2	Non-synonymous	N54S	0.001	0.69	0.101
Modern terminal	U	A	7746	G	COX2	Non-synonymous	N54S	0.001	0.69	0.101
Pre-terminal	U	A	7746	G	COX2	Non-synonymous	N54S	0.001	0.69	0.101
Modern terminal	JT	A	7746	G	COX2	Non-synonymous	N54S	0.001	0.69	0.101
Modern terminal	R0	T	7749	C	COX2	Non-synonymous	I55T	0.999	0.36	0.072
Ancient terminal	U	T	7749	A	COX2	Non-synonymous	I55N	1	0.05	0.383
Modern terminal	JT	T	7749	G	COX2	Non-synonymous	I55S	1	0.08	0.366
Modern terminal	R0	T	7751	G	COX2	Non-synonymous	S56A	0	0.12	0.221
Modern terminal	U	T	7751	G	COX2	Non-synonymous	S56A	0	0.12	0.221
Ancient terminal	R0	G	7754	A	COX2	Non-synonymous	D57N	0.002	0.17	0.35
Modern terminal	R0	G	7754	A	COX2	Non-synonymous	D57N	0.002	0.17	0.35
Modern terminal	R0	G	7754	A	COX2	Non-synonymous	D57N	0.002	0.17	0.35
Modern terminal	R0	G	7754	A	COX2	Non-synonymous	D57N	0.002	0.17	0.35
Modern terminal	R0	G	7754	C	COX2	Non-synonymous	D57H	0.842	0.67	0.454
Modern terminal	R0	G	7754	A	COX2	Non-synonymous	D57N	0.002	0.17	0.35
Modern terminal	U	G	7754	A	COX2	Non-synonymous	D57N	0.002	0.17	0.35
Modern terminal	U	G	7754	A	COX2	Non-synonymous	D57N	0.002	0.17	0.35
Modern terminal	U	G	7754	A	COX2	Non-synonymous	D57N	0.002	0.17	0.35
Modern terminal	U	G	7754	A	COX2	Non-synonymous	D57N	0.002	0.17	0.35
Ancient terminal	JT	G	7754	A	COX2	Non-synonymous	D57N	0.002	0.17	0.35
Modern terminal	JT	G	7754	A	COX2	Non-synonymous	D57N	0.002	0.17	0.35

Modern terminal	JT	G	7754	A	COX2	Non-synonymous	D57N	0.002	0.17	0.35
Pre-terminal	JT	G	7754	A	COX2	Non-synonymous	D57N	0.002	0.17	0.35
Modern terminal	R0	G	7757	A	COX2	Non-synonymous	A58T	0.995	0	0.435
Modern terminal	R0	G	7757	A	COX2	Non-synonymous	A58T	0.995	0	0.435
Modern terminal	R0	A	7761	G	COX2	Non-synonymous	Q59R	0.896	0.01	0.456
Modern terminal	R0	A	7761	G	COX2	Non-synonymous	Q59R	0.896	0.01	0.456
Modern terminal	R0	A	7761	G	COX2	Non-synonymous	Q59R	0.896	0.01	0.456
Modern terminal	R0	A	7765	T	COX2	Non-synonymous	E60D	0.986	0.25	0.512
Modern terminal	U	A	7766	T	COX2	Non-synonymous	M61L	0.178	0.06	0.318
Modern terminal	R0	G	7769	A	COX2	Non-synonymous	E62K	0.986	0	0.68
Ancient terminal	R0	A	7772	G	COX2	Non-synonymous	T63A	0.197	0.07	0.49
Modern terminal	R0	A	7772	G	COX2	Non-synonymous	T63A	0.197	0.07	0.49
Modern terminal	R0	A	7772	G	COX2	Non-synonymous	T63A	0.197	0.07	0.49
Pre-terminal	R0	A	7772	G	COX2	Non-synonymous	T63A	0.197	0.07	0.49
Modern terminal	JT	A	7772	G	COX2	Non-synonymous	T63A	0.197	0.07	0.49
Pre-terminal	JT	A	7772	G	COX2	Non-synonymous	T63A	0.197	0.07	0.49
Modern terminal	N1	A	7772	G	COX2	Non-synonymous	T63A	0.197	0.07	0.49
Modern terminal	R0	G	7775	A	COX2	Non-synonymous	V64I	0	1	0.062
Modern terminal	R0	G	7775	A	COX2	Non-synonymous	V64I	0	1	0.062
Modern terminal	R0	G	7775	A	COX2	Non-synonymous	V64I	0	1	0.062
Modern terminal	R0	G	7775	A	COX2	Non-synonymous	V64I	0	1	0.062
Modern terminal	R0	G	7775	A	COX2	Non-synonymous	V64I	0	1	0.062
Pre-terminal	R0	G	7775	A	COX2	Non-synonymous	V64I	0	1	0.062
Pre-terminal	R0	G	7775	A	COX2	Non-synonymous	V64I	0	1	0.062
Pre-terminal	R0	G	7775	A	COX2	Non-synonymous	V64I	0	1	0.062
Modern terminal	U	G	7775	A	COX2	Non-synonymous	V64I	0	1	0.062
Modern terminal	U	G	7775	A	COX2	Non-synonymous	V64I	0	1	0.062
Modern terminal	U	G	7775	A	COX2	Non-synonymous	V64I	0	1	0.062
Modern terminal	U	G	7775	A	COX2	Non-synonymous	V64I	0	1	0.062
Modern terminal	U	G	7775	A	COX2	Non-synonymous	V64I	0	1	0.062
Pre-terminal	U	G	7775	A	COX2	Non-synonymous	V64I	0	1	0.062
Pre-terminal	U	G	7775	A	COX2	Non-synonymous	V64I	0	1	0.062
Pre-terminal	U	G	7775	A	COX2	Non-synonymous	V64I	0	1	0.062
Ancient terminal	JT	G	7775	A	COX2	Non-synonymous	V64I	0	1	0.062
Modern terminal	JT	G	7775	A	COX2	Non-synonymous	V64I	0	1	0.062
Modern terminal	JT	G	7775	A	COX2	Non-synonymous	V64I	0	1	0.062
Pre-terminal	JT	G	7775	A	COX2	Non-synonymous	V64I	0	1	0.062
Pre-terminal	JT	G	7775	A	COX2	Non-synonymous	V64I	0	1	0.062
Modern terminal	X	G	7775	A	COX2	Non-synonymous	V64I	0	1	0.062
Modern terminal	JT	A	7784	G	COX2	Non-synonymous	I67V	0	0.19	0.28
Modern terminal	R0	T	7785	C	COX2	Non-synonymous	I67T	0.003	0.01	0.687
Pre-terminal	R0	T	7785	C	COX2	Non-synonymous	I67T	0.003	0.01	0.687
Modern terminal	U	T	7785	C	COX2	Non-synonymous	I67T	0.003	0.01	0.687
Modern terminal	U	T	7785	C	COX2	Non-synonymous	I67T	0.003	0.01	0.687
Modern terminal	JT	T	7785	C	COX2	Non-synonymous	I67T	0.003	0.01	0.687
Ancient terminal	U	T	7788	A	COX2	Non-synonymous	L68Q	1	0	0.836
Modern terminal	R0	G	7793	A	COX2	Non-synonymous	A70T	0.997	0.01	0.671
Pre-terminal	R0	A	7796	G	COX2	Non-synonymous	I71V	0.001	0.51	0.162
Pre-terminal	N1	A	7796	G	COX2	Non-synonymous	I71V	0.001	0.51	0.162
Ancient terminal	R0	G	7805	A	COX2	Non-synonymous	V74I	0	1	0.051

Ancient terminal	R0	G	7805	A	COX2	Non-synonymous	V74I	0	1	0.051
Modern terminal	R0	G	7805	A	COX2	Non-synonymous	V74I	0	1	0.051
Modern terminal	R0	G	7805	A	COX2	Non-synonymous	V74I	0	1	0.051
Modern terminal	R0	G	7805	A	COX2	Non-synonymous	V74I	0	1	0.051
Modern terminal	R0	G	7805	A	COX2	Non-synonymous	V74I	0	1	0.051
Pre-terminal	R0	G	7805	A	COX2	Non-synonymous	V74I	0	1	0.051
Pre-terminal	R0	G	7805	A	COX2	Non-synonymous	V74I	0	1	0.051
Pre-terminal	R0	G	7805	A	COX2	Non-synonymous	V74I	0	1	0.051
Pre-terminal	R0	G	7805	A	COX2	Non-synonymous	V74I	0	1	0.051
Pre-terminal	R0	G	7805	A	COX2	Non-synonymous	V74I	0	1	0.051
Pre-terminal	R0	G	7805	A	COX2	Non-synonymous	V74I	0	1	0.051
Pre-terminal	R0	G	7805	A	COX2	Non-synonymous	V74I	0	1	0.051
Ancient terminal	U	G	7805	A	COX2	Non-synonymous	V74I	0	1	0.051
Modern terminal	U	G	7805	A	COX2	Non-synonymous	V74I	0	1	0.051
Modern terminal	U	G	7805	A	COX2	Non-synonymous	V74I	0	1	0.051
Modern terminal	U	G	7805	A	COX2	Non-synonymous	V74I	0	1	0.051
Modern terminal	U	G	7805	A	COX2	Non-synonymous	V74I	0	1	0.051
Modern terminal	U	G	7805	A	COX2	Non-synonymous	V74I	0	1	0.051
Pre-terminal	U	G	7805	A	COX2	Non-synonymous	V74I	0	1	0.051
Pre-terminal	U	G	7805	A	COX2	Non-synonymous	V74I	0	1	0.051
Pre-terminal	U	G	7805	A	COX2	Non-synonymous	V74I	0	1	0.051
Ancient terminal	JT	G	7805	A	COX2	Non-synonymous	V74I	0	1	0.051
Modern terminal	JT	G	7805	A	COX2	Non-synonymous	V74I	0	1	0.051
Modern terminal	JT	G	7805	A	COX2	Non-synonymous	V74I	0	1	0.051
Pre-terminal	JT	G	7805	A	COX2	Non-synonymous	V74I	0	1	0.051
Pre-terminal	JT	G	7805	A	COX2	Non-synonymous	V74I	0	1	0.051
Modern terminal	N1	G	7805	A	COX2	Non-synonymous	V74I	0	1	0.051
Pre-terminal	N1	G	7805	A	COX2	Non-synonymous	V74I	0	1	0.051
Ancient terminal	JT	T	7806	G	COX2	Non-synonymous	V74G	0.934	0	0.484
Modern terminal	R0	T	7812	C	COX2	Non-synonymous	I76T	1	0	0.601
Ancient terminal	R0	T	7827	A	COX2	Non-synonymous	L81Q	1	0	0.848
Modern terminal	R0	G	7830	A	COX2	Non-synonymous	R82H	1	0.04	0.594
Modern terminal	R0	G	7830	A	COX2	Non-synonymous	R82H	1	0.04	0.594
Modern terminal	R0	G	7830	A	COX2	Non-synonymous	R82H	1	0.04	0.594
Modern terminal	R0	G	7830	A	COX2	Non-synonymous	R82H	1	0.04	0.594
Pre-terminal	R0	G	7830	A	COX2	Non-synonymous	R82H	1	0.04	0.594
Pre-terminal	R0	G	7830	A	COX2	Non-synonymous	R82H	1	0.04	0.594
Pre-terminal	R0	G	7830	A	COX2	Non-synonymous	R82H	1	0.04	0.594
Pre-terminal	R0	G	7830	A	COX2	Non-synonymous	R82H	1	0.04	0.594
Ancient terminal	U	G	7830	A	COX2	Non-synonymous	R82H	1	0.04	0.594
Modern terminal	U	G	7830	A	COX2	Non-synonymous	R82H	1	0.04	0.594
Modern terminal	U	G	7830	A	COX2	Non-synonymous	R82H	1	0.04	0.594
Modern terminal	U	G	7830	A	COX2	Non-synonymous	R82H	1	0.04	0.594
Modern terminal	U	G	7830	A	COX2	Non-synonymous	R82H	1	0.04	0.594
Modern terminal	U	G	7830	A	COX2	Non-synonymous	R82H	1	0.04	0.594
Pre-terminal	U	G	7830	A	COX2	Non-synonymous	R82H	1	0.04	0.594
Modern terminal	JT	G	7830	A	COX2	Non-synonymous	R82H	1	0.04	0.594
Modern terminal	JT	G	7830	A	COX2	Non-synonymous	R82H	1	0.04	0.594
Modern terminal	JT	G	7830	A	COX2	Non-synonymous	R82H	1	0.04	0.594
Pre-terminal	N1	G	7830	A	COX2	Non-synonymous	R82H	1	0.04	0.594

Modern terminal	R0	A	7844	G	COX2	Non-synonymous	T87A	0	0	0.525
Modern terminal	R0	A	7844	G	COX2	Non-synonymous	T87A	0	0	0.525
Modern terminal	R0	A	7844	G	COX2	Non-synonymous	T87A	0	0	0.525
Modern terminal	R0	A	7844	G	COX2	Non-synonymous	T87A	0	0	0.525
Pre-terminal	R0	A	7844	G	COX2	Non-synonymous	T87A	0	0	0.525
Modern terminal	U	A	7844	G	COX2	Non-synonymous	T87A	0	0	0.525
Modern terminal	U	A	7844	G	COX2	Non-synonymous	T87A	0	0	0.525
Pre-terminal	U	A	7844	G	COX2	Non-synonymous	T87A	0	0	0.525
Modern terminal	JT	A	7844	G	COX2	Non-synonymous	T87A	0	0	0.525
Modern terminal	JT	A	7844	G	COX2	Non-synonymous	T87A	0	0	0.525
Pre-terminal	N2	A	7844	G	COX2	Non-synonymous	T87A	0	0	0.525
Modern terminal	R0	C	7845	T	COX2	Non-synonymous	T87M	0.663	1	0.162
Ancient terminal	R0	G	7853	A	COX2	Non-synonymous	V90I	0	1	0.039
Modern terminal	R0	G	7853	A	COX2	Non-synonymous	V90I	0	1	0.039
Modern terminal	R0	G	7853	A	COX2	Non-synonymous	V90I	0	1	0.039
Modern terminal	R0	G	7853	A	COX2	Non-synonymous	V90I	0	1	0.039
Modern terminal	R0	G	7853	A	COX2	Non-synonymous	V90I	0	1	0.039
Modern terminal	R0	G	7853	A	COX2	Non-synonymous	V90I	0	1	0.039
Modern terminal	R0	G	7853	A	COX2	Non-synonymous	V90I	0	1	0.039
Modern terminal	R0	G	7853	A	COX2	Non-synonymous	V90I	0	1	0.039
Pre-terminal	R0	G	7853	A	COX2	Non-synonymous	V90I	0	1	0.039
Pre-terminal	R0	G	7853	A	COX2	Non-synonymous	V90I	0	1	0.039
Ancient terminal	U	G	7853	A	COX2	Non-synonymous	V90I	0	1	0.039
Modern terminal	U	G	7853	A	COX2	Non-synonymous	V90I	0	1	0.039
Modern terminal	U	G	7853	A	COX2	Non-synonymous	V90I	0	1	0.039
Modern terminal	U	G	7853	A	COX2	Non-synonymous	V90I	0	1	0.039
Modern terminal	U	G	7853	A	COX2	Non-synonymous	V90I	0	1	0.039
Modern terminal	U	G	7853	A	COX2	Non-synonymous	V90I	0	1	0.039
Pre-terminal	U	G	7853	A	COX2	Non-synonymous	V90I	0	1	0.039
Pre-terminal	U	G	7853	A	COX2	Non-synonymous	V90I	0	1	0.039
Pre-terminal	U	G	7853	A	COX2	Non-synonymous	V90I	0	1	0.039
Pre-terminal	U	G	7853	A	COX2	Non-synonymous	V90I	0	1	0.039
Pre-terminal	U	G	7853	A	COX2	Non-synonymous	V90I	0	1	0.039
Pre-terminal	U	G	7853	A	COX2	Non-synonymous	V90I	0	1	0.039
Pre-terminal	U	G	7853	A	COX2	Non-synonymous	V90I	0	1	0.039
Pre-terminal	U	G	7853	A	COX2	Non-synonymous	V90I	0	1	0.039
Pre-terminal	U	G	7853	A	COX2	Non-synonymous	V90I	0	1	0.039
Modern terminal	JT	G	7853	A	COX2	Non-synonymous	V90I	0	1	0.039
Modern terminal	JT	G	7853	A	COX2	Non-synonymous	V90I	0	1	0.039
Modern terminal	JT	G	7853	A	COX2	Non-synonymous	V90I	0	1	0.039
Modern terminal	JT	G	7853	A	COX2	Non-synonymous	V90I	0	1	0.039
Modern terminal	JT	G	7853	A	COX2	Non-synonymous	V90I	0	1	0.039
Pre-terminal	JT	G	7853	A	COX2	Non-synonymous	V90I	0	1	0.039
Pre-terminal	JT	G	7853	A	COX2	Non-synonymous	V90I	0	1	0.039
Pre-terminal	JT	G	7853	A	COX2	Non-synonymous	V90I	0	1	0.039
Pre-terminal	JT	G	7853	A	COX2	Non-synonymous	V90I	0	1	0.039
Modern terminal	N1	G	7853	A	COX2	Non-synonymous	V90I	0	1	0.039
Pre-terminal	N1	G	7853	A	COX2	Non-synonymous	V90I	0	1	0.039
Pre-terminal	X	G	7853	A	COX2	Non-synonymous	V90I	0	1	0.039
Pre-terminal	X	G	7853	A	COX2	Non-synonymous	V90I	0	1	0.039

Modern terminal	N2	G	7853	A	COX2	Non-synonymous	V90I	0	1	0.039
Modern terminal	N2	G	7853	A	COX2	Non-synonymous	V90I	0	1	0.039
Modern terminal	N2	G	7853	A	COX2	Non-synonymous	V90I	0	1	0.039
Pre-terminal	N2	G	7853	A	COX2	Non-synonymous	V90I	0	1	0.039
Modern terminal	R0	T	7854	C	COX2	Non-synonymous	V90A	0.507	0	0.297
Modern terminal	JT	T	7854	C	COX2	Non-synonymous	V90A	0.507	0	0.297
Ancient terminal	U	A	7856	T	COX2	Non-synonymous	N91Y	0.999	0.25	0.643
Ancient terminal	R0	C	7858	A	COX2	Non-synonymous	N91K	0.995	0.1	0.682
Modern terminal	R0	G	7859	A	COX2	Non-synonymous	D92N	0	1	0.109
Modern terminal	R0	G	7859	A	COX2	Non-synonymous	D92N	0	1	0.109
Modern terminal	R0	G	7859	A	COX2	Non-synonymous	D92N	0	1	0.109
Modern terminal	R0	G	7859	A	COX2	Non-synonymous	D92N	0	1	0.109
Modern terminal	R0	G	7859	A	COX2	Non-synonymous	D92N	0	1	0.109
Modern terminal	R0	G	7859	A	COX2	Non-synonymous	D92N	0	1	0.109
Modern terminal	R0	G	7859	A	COX2	Non-synonymous	D92N	0	1	0.109
Pre-terminal	R0	G	7859	A	COX2	Non-synonymous	D92N	0	1	0.109
Pre-terminal	R0	G	7859	A	COX2	Non-synonymous	D92N	0	1	0.109
Pre-terminal	R0	G	7859	A	COX2	Non-synonymous	D92N	0	1	0.109
Ancient terminal	U	G	7859	A	COX2	Non-synonymous	D92N	0	1	0.109
Modern terminal	U	G	7859	A	COX2	Non-synonymous	D92N	0	1	0.109
Modern terminal	U	G	7859	A	COX2	Non-synonymous	D92N	0	1	0.109
Modern terminal	U	G	7859	A	COX2	Non-synonymous	D92N	0	1	0.109
Modern terminal	U	G	7859	A	COX2	Non-synonymous	D92N	0	1	0.109
Modern terminal	U	G	7859	A	COX2	Non-synonymous	D92N	0	1	0.109
Modern terminal	U	G	7859	A	COX2	Non-synonymous	D92N	0	1	0.109
Pre-terminal	U	G	7859	A	COX2	Non-synonymous	D92N	0	1	0.109
Pre-terminal	U	G	7859	A	COX2	Non-synonymous	D92N	0	1	0.109
Pre-terminal	U	G	7859	A	COX2	Non-synonymous	D92N	0	1	0.109
Modern terminal	JT	G	7859	A	COX2	Non-synonymous	D92N	0	1	0.109
Modern terminal	JT	G	7859	A	COX2	Non-synonymous	D92N	0	1	0.109
Pre-terminal	JT	G	7859	A	COX2	Non-synonymous	D92N	0	1	0.109
Modern terminal	X	G	7859	A	COX2	Non-synonymous	D92N	0	1	0.109
Pre-terminal	U	C	7868	T	COX2	Non-synonymous	L95F	0	0.08	0.562
Modern terminal	N1	A	7874	G	COX2	Non-synonymous	I97V	0.913	0.62	0.075
Modern terminal	N2	A	7874	G	COX2	Non-synonymous	I97V	0.913	0.62	0.075
Ancient terminal	R0	C	7876	A	COX2	Non-synonymous	I97M	1	0.03	0.387
Modern terminal	U	A	7883	G	COX2	Non-synonymous	I100V	0	0.14	0.099
Modern terminal	JT	G	7887	C	COX2	Non-synonymous	G101A	1	0	0.611
Ancient terminal	R0	C	7892	A	COX2	Non-synonymous	Q103K	0.966	0	0.583
Modern terminal	U	C	7892	T	COX2	Nonsense				
Ancient terminal	U	A	7893	T	COX2	Non-synonymous	Q103L	0.985	0	0.703
Ancient terminal	U	T	7895	C	COX2	Non-synonymous	W104R	1	0	0.864
Ancient terminal	U	A	7904	G	COX2	Non-synonymous	T107A	0.001	0	0.279
Ancient terminal	U	G	7910	T	COX2	Nonsense				
Ancient terminal	U	A	7916	G	COX2	Non-synonymous	T111A	0.992	0.03	0.461
Modern terminal	R0	T	7922	C	COX2	Non-synonymous	Y113H	0.217	0	0.639
Modern terminal	U	T	7922	G	COX2	Non-synonymous	Y113D	0.999	0	0.79
Modern terminal	R0	G	7925	A	COX2	Non-synonymous	G114S	0.862	0.35	0.145
Modern terminal	R0	G	7925	A	COX2	Non-synonymous	G114S	0.862	0.35	0.145
Modern terminal	JT	G	7925	A	COX2	Non-synonymous	G114S	0.862	0.35	0.145

Modern terminal	X	G	7925	A	COX2	Non-synonymous	G114S	0.862	0.35	0.145
Modern terminal	R0	A	7934	G	COX2	Non-synonymous	I117V	0	0.27	0.081
Modern terminal	N1	A	7934	G	COX2	Non-synonymous	I117V	0	0.27	0.081
Modern terminal	U	A	7940	G	COX2	Non-synonymous	N119D	0.999	1	0.08
Modern terminal	R0	A	7941	G	COX2	Non-synonymous	N119S	0.999	0	0.213
Modern terminal	R0	A	7941	G	COX2	Non-synonymous	N119S	0.999	0	0.213
Pre-terminal	U	A	7941	G	COX2	Non-synonymous	N119S	0.999	0	0.213
Ancient terminal	JT	A	7941	G	COX2	Non-synonymous	N119S	0.999	0	0.213
Ancient terminal	JT	A	7941	G	COX2	Non-synonymous	N119S	0.999	0	0.213
Ancient terminal	JT	A	7941	G	COX2	Non-synonymous	N119S	0.999	0	0.213
Pre-terminal	N1	A	7941	G	COX2	Non-synonymous	N119S	0.999	0	0.213
Ancient terminal	N2	C	7942	A	COX2	Non-synonymous	N119K	1	0	0.521
Modern terminal	JT	C	7955	T	COX2	Non-synonymous	P124S	0.999	0.02	0.576
Modern terminal	R0	C	7958	T	COX2	Non-synonymous	P125S	0.019	0.2	0.165
Ancient terminal	JT	C	7958	T	COX2	Non-synonymous	P125S	0.019	0.2	0.165
Modern terminal	JT	C	7958	T	COX2	Non-synonymous	P125S	0.019	0.2	0.165
Pre-terminal	N2	T	7961	G	COX2	Non-synonymous	L126V	0.965	0.08	0.111
Modern terminal	R0	T	7962	C	COX2	Non-synonymous	L126S	1	0.96	0.104
Pre-terminal	R0	T	7962	C	COX2	Non-synonymous	L126S	1	0.96	0.104
Ancient terminal	U	T	7962	C	COX2	Non-synonymous	L126S	1	0.96	0.104
Modern terminal	R0	T	7964	C	COX2	Non-synonymous	F127L	0.998	0.12	0.168
Modern terminal	U	T	7964	C	COX2	Non-synonymous	F127L	0.998	0.12	0.168
Pre-terminal	U	T	7964	C	COX2	Non-synonymous	F127L	0.998	0.12	0.168
Pre-terminal	U	T	7964	C	COX2	Non-synonymous	F127L	0.998	0.12	0.168
Modern terminal	JT	T	7964	C	COX2	Non-synonymous	F127L	0.998	0.12	0.168
Modern terminal	N1	T	7964	C	COX2	Non-synonymous	F127L	0.998	0.12	0.168
Modern terminal	N2	T	7964	C	COX2	Non-synonymous	F127L	0.998	0.12	0.168
Pre-terminal	R0	C	7967	A	COX2	Non-synonymous	L128M	1	0.04	0.24
Modern terminal	U	A	7971	G	COX2	Non-synonymous	E129G	0.398	0.07	0.266
Modern terminal	U	A	7971	G	COX2	Non-synonymous	E129G	0.398	0.07	0.266
Modern terminal	R0	G	7976	A	COX2	Non-synonymous	G131S	1	0.03	0.501
Pre-terminal	R0	G	7976	A	COX2	Non-synonymous	G131S	1	0.03	0.501
Modern terminal	JT	G	7977	C	COX2	Non-synonymous	G131A	0.999	0	0.521
Modern terminal	R0	G	7979	A	COX2	Non-synonymous	D132N	0.994	0.32	0.139
Pre-terminal	R0	G	7979	A	COX2	Non-synonymous	D132N	0.994	0.32	0.139
Modern terminal	U	G	7979	A	COX2	Non-synonymous	D132N	0.994	0.32	0.139
Ancient terminal	JT	G	7979	A	COX2	Non-synonymous	D132N	0.994	0.32	0.139
Modern terminal	JT	G	7979	A	COX2	Non-synonymous	D132N	0.994	0.32	0.139
Modern terminal	N2	G	7979	A	COX2	Non-synonymous	D132N	0.994	0.32	0.139
Modern terminal	R0	A	7980	G	COX2	Non-synonymous	D132G	1	0.39	0.238
Modern terminal	R0	A	7980	G	COX2	Non-synonymous	D132G	1	0.39	0.238
Modern terminal	R0	A	7980	G	COX2	Non-synonymous	D132G	1	0.39	0.238
Modern terminal	R0	A	7980	G	COX2	Non-synonymous	D132G	1	0.39	0.238
Modern terminal	R0	A	7980	G	COX2	Non-synonymous	D132G	1	0.39	0.238
Modern terminal	R0	A	7980	G	COX2	Non-synonymous	D132G	1	0.39	0.238
Pre-terminal	R0	A	7980	G	COX2	Non-synonymous	D132G	1	0.39	0.238
Pre-terminal	N1	A	7980	G	COX2	Non-synonymous	D132G	1	0.39	0.238
Modern terminal	U	G	8009	A	COX2	Non-synonymous	V142M	0.999	0.25	0.382
Modern terminal	R0	T	8022	C	COX2	Non-synonymous	I146T	0.679	0.17	0.209
Modern terminal	R0	T	8022	C	COX2	Non-synonymous	I146T	0.679	0.17	0.209

Pre-terminal	R0	T	8022	C	COX2	Non-synonymous	I146T	0.679	0.17	0.209
Pre-terminal	R0	T	8022	C	COX2	Non-synonymous	I146T	0.679	0.17	0.209
Modern terminal	U	T	8022	C	COX2	Non-synonymous	I146T	0.679	0.17	0.209
Modern terminal	JT	T	8022	C	COX2	Non-synonymous	I146T	0.679	0.17	0.209
Modern terminal	R0	G	8024	A	COX2	Non-synonymous	E147K	0	0.23	0.56
Pre-terminal	R0	A	8026	T	COX2	Non-synonymous	E147D	0	0.31	0.398
Modern terminal	R0	G	8027	A	COX2	Non-synonymous	A148T	0.997	1	0.159
Modern terminal	R0	G	8027	A	COX2	Non-synonymous	A148T	0.997	1	0.159
Modern terminal	R0	G	8027	A	COX2	Non-synonymous	A148T	0.997	1	0.159
Modern terminal	R0	G	8027	A	COX2	Non-synonymous	A148T	0.997	1	0.159
Modern terminal	R0	G	8027	A	COX2	Non-synonymous	A148T	0.997	1	0.159
Modern terminal	R0	G	8027	A	COX2	Non-synonymous	A148T	0.997	1	0.159
Pre-terminal	R0	G	8027	A	COX2	Non-synonymous	A148T	0.997	1	0.159
Pre-terminal	R0	G	8027	A	COX2	Non-synonymous	A148T	0.997	1	0.159
Pre-terminal	R0	G	8027	A	COX2	Non-synonymous	A148T	0.997	1	0.159
Pre-terminal	R0	G	8027	A	COX2	Non-synonymous	A148T	0.997	1	0.159
Ancient terminal	U	G	8027	A	COX2	Non-synonymous	A148T	0.997	1	0.159
Modern terminal	U	G	8027	A	COX2	Non-synonymous	A148T	0.997	1	0.159
Modern terminal	U	G	8027	A	COX2	Non-synonymous	A148T	0.997	1	0.159
Modern terminal	U	G	8027	A	COX2	Non-synonymous	A148T	0.997	1	0.159
Modern terminal	U	G	8027	A	COX2	Non-synonymous	A148T	0.997	1	0.159
Modern terminal	U	G	8027	A	COX2	Non-synonymous	A148T	0.997	1	0.159
Modern terminal	U	G	8027	A	COX2	Non-synonymous	A148T	0.997	1	0.159
Modern terminal	U	G	8027	A	COX2	Non-synonymous	A148T	0.997	1	0.159
Pre-terminal	U	G	8027	A	COX2	Non-synonymous	A148T	0.997	1	0.159
Pre-terminal	U	G	8027	A	COX2	Non-synonymous	A148T	0.997	1	0.159
Pre-terminal	U	G	8027	A	COX2	Non-synonymous	A148T	0.997	1	0.159
Pre-terminal	U	G	8027	A	COX2	Non-synonymous	A148T	0.997	1	0.159
Modern terminal	JT	G	8027	A	COX2	Non-synonymous	A148T	0.997	1	0.159
Modern terminal	JT	G	8027	A	COX2	Non-synonymous	A148T	0.997	1	0.159
Modern terminal	JT	G	8027	A	COX2	Non-synonymous	A148T	0.997	1	0.159
Modern terminal	JT	G	8027	A	COX2	Non-synonymous	A148T	0.997	1	0.159
Modern terminal	JT	G	8027	A	COX2	Non-synonymous	A148T	0.997	1	0.159
Modern terminal	JT	G	8027	A	COX2	Non-synonymous	A148T	0.997	1	0.159
Pre-terminal	JT	G	8027	A	COX2	Non-synonymous	A148T	0.997	1	0.159
Pre-terminal	JT	G	8027	A	COX2	Non-synonymous	A148T	0.997	1	0.159
Pre-terminal	N1	G	8027	A	COX2	Non-synonymous	A148T	0.997	1	0.159
Modern terminal	X	G	8027	A	COX2	Non-synonymous	A148T	0.997	1	0.159
Modern terminal	N2	G	8027	A	COX2	Non-synonymous	A148T	0.997	1	0.159
Modern terminal	N1	C	8028	T	COX2	Non-synonymous	A148V	0.999	0.09	0.215
Modern terminal	R0	A	8033	G	COX2	Non-synonymous	I150V	0	0.3	0.119
Pre-terminal	R0	A	8033	G	COX2	Non-synonymous	I150V	0	0.3	0.119
Modern terminal	R0	T	8051	C	COX2	Non-synonymous	S156P	0.997	0.07	0.743
Modern terminal	R0	A	8058	G	COX2	Non-synonymous	D158G	1	0	0.611
Modern terminal	R0	G	8060	A	COX2	Non-synonymous	V159I	0.876	0	0.128
Ancient terminal	JT	C	8066	T	COX2	Non-synonymous	H161Y	0.989	0	0.439
Modern terminal	R0	G	8075	A	COX2	Non-synonymous	A164T	0	0.44	0.197
Modern terminal	R0	G	8075	A	COX2	Non-synonymous	A164T	0	0.44	0.197
Modern terminal	U	G	8075	A	COX2	Non-synonymous	A164T	0	0.44	0.197
Pre-terminal	U	G	8075	A	COX2	Non-synonymous	A164T	0	0.44	0.197

Ancient terminal	U	G	8078	A	COX2	Non-synonymous	V165I	0	0.35	0.063
Modern terminal	U	G	8078	A	COX2	Non-synonymous	V165I	0	0.35	0.063
Modern terminal	U	G	8078	A	COX2	Non-synonymous	V165I	0	0.35	0.063
Modern terminal	U	G	8078	A	COX2	Non-synonymous	V165I	0	0.35	0.063
Modern terminal	JT	G	8078	A	COX2	Non-synonymous	V165I	0	0.35	0.063
Modern terminal	JT	G	8078	A	COX2	Non-synonymous	V165I	0	0.35	0.063
Pre-terminal	JT	G	8078	A	COX2	Non-synonymous	V165I	0	0.35	0.063
Modern terminal	N1	G	8078	A	COX2	Non-synonymous	V165I	0	0.35	0.063
Pre-terminal	N2	G	8078	A	COX2	Non-synonymous	V165I	0	0.35	0.063
Modern terminal	R0	A	8084	G	COX2	Non-synonymous	T167A	0	0.18	0.102
Modern terminal	R0	A	8084	G	COX2	Non-synonymous	T167A	0	0.18	0.102
Modern terminal	R0	A	8084	G	COX2	Non-synonymous	T167A	0	0.18	0.102
Modern terminal	R0	A	8084	G	COX2	Non-synonymous	T167A	0	0.18	0.102
Modern terminal	R0	A	8084	T	COX2	Non-synonymous	T167S	0	1	0.044
Modern terminal	R0	A	8084	T	COX2	Non-synonymous	T167S	0	1	0.044
Modern terminal	R0	A	8084	G	COX2	Non-synonymous	T167A	0	0.18	0.102
Pre-terminal	R0	A	8084	T	COX2	Non-synonymous	T167S	0	1	0.044
Ancient terminal	U	A	8084	G	COX2	Non-synonymous	T167A	0	0.18	0.102
Modern terminal	U	A	8084	G	COX2	Non-synonymous	T167A	0	0.18	0.102
Modern terminal	U	A	8084	G	COX2	Non-synonymous	T167A	0	0.18	0.102
Modern terminal	U	A	8084	G	COX2	Non-synonymous	T167A	0	0.18	0.102
Modern terminal	JT	A	8084	G	COX2	Non-synonymous	T167A	0	0.18	0.102
Modern terminal	JT	A	8084	G	COX2	Non-synonymous	T167A	0	0.18	0.102
Pre-terminal	JT	A	8084	G	COX2	Non-synonymous	T167A	0	0.18	0.102
Pre-terminal	N1	A	8084	G	COX2	Non-synonymous	T167A	0	0.18	0.102
Modern terminal	R0	A	8108	G	COX2	Non-synonymous	I175V	0.001	0.77	0.166
Pre-terminal	R0	A	8108	G	COX2	Non-synonymous	I175V	0.001	0.77	0.166
Pre-terminal	R0	A	8108	G	COX2	Non-synonymous	I175V	0.001	0.77	0.166
Pre-terminal	R0	A	8108	G	COX2	Non-synonymous	I175V	0.001	0.77	0.166
Modern terminal	U	A	8108	G	COX2	Non-synonymous	I175V	0.001	0.77	0.166
Modern terminal	U	A	8108	G	COX2	Non-synonymous	I175V	0.001	0.77	0.166
Modern terminal	JT	A	8108	G	COX2	Non-synonymous	I175V	0.001	0.77	0.166
Modern terminal	N1	A	8108	G	COX2	Non-synonymous	I175V	0.001	0.77	0.166
Ancient terminal	U	T	8121	A	COX2	Non-synonymous	L179Q	1	0	0.81
Modern terminal	R0	A	8129	G	COX2	Non-synonymous	T182A	0.001	0.47	0.12
Modern terminal	R0	A	8129	G	COX2	Non-synonymous	T182A	0.001	0.47	0.12
Modern terminal	R0	C	8130	T	COX2	Non-synonymous	T182I	0.334	0.27	0.283
Modern terminal	R0	C	8139	T	COX2	Non-synonymous	T185I	0.999	0.27	0.236
Pre-terminal	R0	G	8141	A	COX2	Non-synonymous	A186T	0.997	0.29	0.162
Ancient terminal	N2	C	8142	A	COX2	Non-synonymous	A186D	0.999	0.01	0.381
Pre-terminal	U	C	8145	T	COX2	Non-synonymous	T187M	0.03	0.29	0.195
Modern terminal	JT	G	8156	C	COX2	Non-synonymous	V191L	0.074	1	0.061
Ancient terminal	JT	C	8161	A	COX2	Nonsense				
Ancient terminal	JT	C	8168	A	COX2	Non-synonymous	Q195K	0.966	0	0.542
Ancient terminal	U	A	8196	T	COX2	Non-synonymous	H204L	0.994	0	0.788
Ancient terminal	JT	C	8197	A	COX2	Non-synonymous	H204Q	1	0	0.697
Ancient terminal	U	A	8198	T	COX2	Non-synonymous	S205C	1	0.01	0.56
Modern terminal	R0	C	8207	A	COX2	Non-synonymous	P208T	1	0	0.725
Pre-terminal	R0	A	8210	G	COX2	Non-synonymous	I209V	0.944	0.02	0.296
Ancient terminal	R0	A	8225	T	COX2	Non-synonymous	I214F	0.982	0	0.403

Modern terminal	R0	A	8225	G	COX2	Non-synonymous	I214V	0.853	1	0.033
Modern terminal	U	A	8225	G	COX2	Non-synonymous	I214V	0.853	1	0.033
Modern terminal	R0	A	8234	G	COX2	Non-synonymous	K217E	0.99	0.27	0.246
Modern terminal	R0	T	8238	C	COX2	Non-synonymous	I218T	0.369	0.36	0.065
Modern terminal	R0	T	8238	C	COX2	Non-synonymous	I218T	0.369	0.36	0.065
Modern terminal	U	T	8238	C	COX2	Non-synonymous	I218T	0.369	0.36	0.065
Modern terminal	U	T	8238	C	COX2	Non-synonymous	I218T	0.369	0.36	0.065
Pre-terminal	JT	T	8238	C	COX2	Non-synonymous	I218T	0.369	0.36	0.065
Modern terminal	R0	T	8247	C	COX2	Non-synonymous	M221T	0.967	0.35	0.058
Modern terminal	R0	T	8247	C	COX2	Non-synonymous	M221T	0.967	0.35	0.058
Ancient terminal	U	G	8249	T	COX2	Non-synonymous	G222W	1	0.26	0.224
Ancient terminal	R0	G	8255	A	COX2	Non-synonymous	V224M	0.991	0.01	0.043
Pre-terminal	R0	G	8255	A	COX2	Non-synonymous	V224M	0.991	0.01	0.043
Ancient terminal	U	G	8255	A	COX2	Non-synonymous	V224M	0.991	0.01	0.043
Ancient terminal	U	G	8255	A	COX2	Non-synonymous	V224M	0.991	0.01	0.043
Ancient terminal	U	G	8255	A	COX2	Non-synonymous	V224M	0.991	0.01	0.043
Pre-terminal	JT	G	8255	A	COX2	Non-synonymous	V224M	0.991	0.01	0.043
Modern terminal	N1	G	8255	C	COX2	Non-synonymous	V224L	0.401	0.18	0.047
Pre-terminal	JT	T	8256	C	COX2	Non-synonymous	V224A	0.998	0.04	0.046
Modern terminal	R0	T	8258	C	COX2	Non-synonymous	F225L	0.005	0.39	0.064
Pre-terminal	R0	T	8258	C	COX2	Non-synonymous	F225L	0.005	0.39	0.064
Pre-terminal	R0	T	8258	C	COX2	Non-synonymous	F225L	0.005	0.39	0.064
Modern terminal	U	T	8258	C	COX2	Non-synonymous	F225L	0.005	0.39	0.064
Pre-terminal	U	T	8258	A	COX2	Non-synonymous	F225I	0.22	0.04	0.108
Modern terminal	N1	T	8258	C	COX2	Non-synonymous	F225L	0.005	0.39	0.064
Modern terminal	N1	T	8258	C	COX2	Non-synonymous	F225L	0.005	0.39	0.064
Modern terminal	R0	T	8260	A	COX2	Non-synonymous	F225L	0.005	0.39	0.064
Pre-terminal	R0	T	8260	A	COX2	Non-synonymous	F225L	0.005	0.39	0.064
Pre-terminal	N1	A	8261	G	COX2	Non-synonymous	T226A	0	0	0.109
Ancient terminal	R0	C	8264	A	COX2	Non-synonymous	L227M	1	0	0.135
Modern terminal	R0	C	8264	A	COX2	Non-synonymous	L227M	1	0	0.135
Modern terminal	R0	T	8265	C	COX2	Non-synonymous	L227P	1	0	0.522
Pre-terminal	R0	T	8265	C	COX2	Non-synonymous	L227P	1	0	0.522
Modern terminal	U	T	8265	C	COX2	Non-synonymous	L227P	1	0	0.522
Modern terminal	U	T	8265	C	COX2	Non-synonymous	L227P	1	0	0.522
Modern terminal	R0	C	8369	A	ATP8	Non-synonymous	P2T	0.998	0	0.591
Modern terminal	R0	T	8376	C	ATP8	Non-synonymous	L4P	0.994	0	0.851
Modern terminal	R0	T	8376	C	ATP8	Non-synonymous	L4P	0.994	0	0.851
Modern terminal	U	T	8376	C	ATP8	Non-synonymous	L4P	0.994	0	0.851
Modern terminal	JT	T	8376	C	ATP8	Non-synonymous	L4P	0.994	0	0.851
Pre-terminal	JT	T	8376	C	ATP8	Non-synonymous	L4P	0.994	0	0.851
Ancient terminal	R0	A	8381	G	ATP8	Non-synonymous	T6A	0.955	0.01	0.606
Modern terminal	R0	A	8381	G	ATP8	Non-synonymous	T6A	0.955	0.01	0.606
Modern terminal	R0	A	8381	G	ATP8	Non-synonymous	T6A	0.955	0.01	0.606
Pre-terminal	R0	A	8381	G	ATP8	Non-synonymous	T6A	0.955	0.01	0.606
Pre-terminal	R0	A	8381	G	ATP8	Non-synonymous	T6A	0.955	0.01	0.606
Pre-terminal	U	C	8382	T	ATP8	Non-synonymous	T6I	0.994	0	0.625
Ancient terminal	R0	A	8384	G	ATP8	Non-synonymous	T7A	0	0.01	0.364
Ancient terminal	JT	A	8384	G	ATP8	Non-synonymous	T7A	0	0.01	0.364
Modern terminal	JT	A	8384	G	ATP8	Non-synonymous	T7A	0	0.01	0.364

Modern terminal	N2	C	8385	T	ATP8	Non-synonymous	T7I	0.023	0	0.517
Pre-terminal	R0	G	8387	A	ATP8	Non-synonymous	V8M	0.99	0.03	0.312
Pre-terminal	R0	G	8387	A	ATP8	Non-synonymous	V8M	0.99	0.03	0.312
Modern terminal	U	G	8387	A	ATP8	Non-synonymous	V8M	0.99	0.03	0.312
Pre-terminal	U	G	8387	A	ATP8	Non-synonymous	V8M	0.99	0.03	0.312
Modern terminal	JT	G	8387	A	ATP8	Non-synonymous	V8M	0.99	0.03	0.312
Pre-terminal	JT	G	8387	A	ATP8	Non-synonymous	V8M	0.99	0.03	0.312
Ancient terminal	R0	T	8388	C	ATP8	Non-synonymous	V8A	0.922	0.04	0.406
Modern terminal	R0	T	8388	C	ATP8	Non-synonymous	V8A	0.922	0.04	0.406
Modern terminal	R0	T	8388	C	ATP8	Non-synonymous	V8A	0.922	0.04	0.406
Modern terminal	R0	T	8388	C	ATP8	Non-synonymous	V8A	0.922	0.04	0.406
Modern terminal	U	T	8388	C	ATP8	Non-synonymous	V8A	0.922	0.04	0.406
Modern terminal	JT	T	8388	C	ATP8	Non-synonymous	V8A	0.922	0.04	0.406
Modern terminal	R0	C	8393	T	ATP8	Non-synonymous	P10S	0.993	0.38	0.398
Modern terminal	R0	C	8393	T	ATP8	Non-synonymous	P10S	0.993	0.38	0.398
Modern terminal	R0	C	8393	T	ATP8	Non-synonymous	P10S	0.993	0.38	0.398
Pre-terminal	U	C	8393	T	ATP8	Non-synonymous	P10S	0.993	0.38	0.398
Ancient terminal	JT	C	8393	A	ATP8	Non-synonymous	P10T	0.998	0.36	0.426
Modern terminal	JT	C	8393	T	ATP8	Non-synonymous	P10S	0.993	0.38	0.398
Pre-terminal	JT	C	8393	T	ATP8	Non-synonymous	P10S	0.993	0.38	0.398
Modern terminal	X	C	8393	T	ATP8	Non-synonymous	P10S	0.993	0.38	0.398
Modern terminal	R0	C	8394	T	ATP8	Non-synonymous	P10L	0.998	0.75	0.296
Modern terminal	U	C	8394	T	ATP8	Non-synonymous	P10L	0.998	0.75	0.296
Modern terminal	R0	A	8396	G	ATP8	Non-synonymous	T11A	0.955	0.05	0.306
Modern terminal	R0	C	8397	G	ATP8	Non-synonymous	T11S	0.955	0.01	0.23
Modern terminal	R0	A	8399	G	ATP8	Non-synonymous	M12V	0	0.33	0.18
Modern terminal	R0	T	8400	C	ATP8	Non-synonymous	M12T	0	1	0.118
Modern terminal	R0	T	8400	C	ATP8	Non-synonymous	M12T	0	1	0.118
Pre-terminal	R0	T	8400	C	ATP8	Non-synonymous	M12T	0	1	0.118
Pre-terminal	U	T	8400	C	ATP8	Non-synonymous	M12T	0	1	0.118
Modern terminal	JT	T	8400	C	ATP8	Non-synonymous	M12T	0	1	0.118
Pre-terminal	JT	T	8400	C	ATP8	Non-synonymous	M12T	0	1	0.118
Pre-terminal	X	T	8400	C	ATP8	Non-synonymous	M12T	0	1	0.118
Pre-terminal	R0	T	8403	C	ATP8	Non-synonymous	I13T	0.931	0	0.595
Ancient terminal	N1	A	8405	G	ATP8	Non-synonymous	T14A	0	0.19	0.271
Pre-terminal	R0	C	8406	T	ATP8	Non-synonymous	T14I	0.077	0.35	0.298
Pre-terminal	R0	C	8406	T	ATP8	Non-synonymous	T14I	0.077	0.35	0.298
Pre-terminal	R0	C	8406	T	ATP8	Non-synonymous	T14I	0.077	0.35	0.298
Ancient terminal	U	C	8406	T	ATP8	Non-synonymous	T14I	0.077	0.35	0.298
Modern terminal	JT	C	8406	T	ATP8	Non-synonymous	T14I	0.077	0.35	0.298
Modern terminal	JT	C	8406	T	ATP8	Non-synonymous	T14I	0.077	0.35	0.298
Modern terminal	JT	C	8406	T	ATP8	Non-synonymous	T14I	0.077	0.35	0.298
Modern terminal	N1	C	8406	T	ATP8	Non-synonymous	T14I	0.077	0.35	0.298
Pre-terminal	X	C	8406	T	ATP8	Non-synonymous	T14I	0.077	0.35	0.298
Modern terminal	R0	C	8409	T	ATP8	Non-synonymous	P15L	0.998	0.17	0.279
Modern terminal	U	C	8409	T	ATP8	Non-synonymous	P15L	0.998	0.17	0.279
Modern terminal	R0	A	8411	G	ATP8	Non-synonymous	M16V	0.317	0.05	0.554
Modern terminal	R0	A	8411	C	ATP8	Non-synonymous	M16L	0.163	0.01	0.593
Modern terminal	U	A	8411	C	ATP8	Non-synonymous	M16L	0.163	0.01	0.593
Modern terminal	N2	A	8411	G	ATP8	Non-synonymous	M16V	0.317	0.05	0.554

Pre-terminal	R0	T	8412	C	ATP8	Non-synonymous	M16T	0.711	0.19	0.542
Modern terminal	U	T	8412	C	ATP8	Non-synonymous	M16T	0.711	0.19	0.542
Modern terminal	U	T	8412	C	ATP8	Non-synonymous	M16T	0.711	0.19	0.542
Pre-terminal	U	T	8412	C	ATP8	Non-synonymous	M16T	0.711	0.19	0.542
Pre-terminal	JT	T	8412	C	ATP8	Non-synonymous	M16T	0.711	0.19	0.542
Pre-terminal	JT	T	8412	C	ATP8	Non-synonymous	M16T	0.711	0.19	0.542
Modern terminal	N2	T	8412	C	ATP8	Non-synonymous	M16T	0.711	0.19	0.542
Modern terminal	JT	C	8414	T	ATP8	Non-synonymous	L17F	0.994	0.29	0.308
Modern terminal	X	C	8414	T	ATP8	Non-synonymous	L17F	0.994	0.29	0.308
Modern terminal	U	T	8415	C	ATP8	Non-synonymous	L17P	0.994	0.01	0.758
Pre-terminal	R0	C	8417	A	ATP8	Non-synonymous	L18I	0.955	0.37	0.16
Modern terminal	R0	A	8420	G	ATP8	Non-synonymous	T19A	0.955	0.26	0.361
Modern terminal	JT	A	8420	G	ATP8	Non-synonymous	T19A	0.955	0.26	0.361
Ancient terminal	JT	C	8421	A	ATP8	Non-synonymous	T19K	0.981	0	0.648
Ancient terminal	R0	T	8426	C	ATP8	Non-synonymous	F21L	0.922	0.03	0.519
Ancient terminal	R0	T	8426	C	ATP8	Non-synonymous	F21L	0.922	0.03	0.519
Modern terminal	R0	T	8426	C	ATP8	Non-synonymous	F21L	0.922	0.03	0.519
Pre-terminal	R0	T	8426	C	ATP8	Non-synonymous	F21L	0.922	0.03	0.519
Ancient terminal	U	T	8426	C	ATP8	Non-synonymous	F21L	0.922	0.03	0.519
Modern terminal	U	T	8426	C	ATP8	Non-synonymous	F21L	0.922	0.03	0.519
Modern terminal	U	T	8426	C	ATP8	Non-synonymous	F21L	0.922	0.03	0.519
Pre-terminal	U	T	8426	C	ATP8	Non-synonymous	F21L	0.922	0.03	0.519
Modern terminal	JT	T	8426	C	ATP8	Non-synonymous	F21L	0.922	0.03	0.519
Modern terminal	JT	T	8426	C	ATP8	Non-synonymous	F21L	0.922	0.03	0.519
Modern terminal	JT	T	8427	C	ATP8	Non-synonymous	F21S	0.966	0	0.776
Pre-terminal	U	C	8428	A	ATP8	Non-synonymous	F21L	0.922	0.03	0.519
Pre-terminal	R0	C	8429	T	ATP8	Non-synonymous	L22F	0.994	0.01	0.357
Ancient terminal	U	C	8429	T	ATP8	Non-synonymous	L22F	0.994	0.01	0.357
Pre-terminal	U	C	8429	T	ATP8	Non-synonymous	L22F	0.994	0.01	0.357
Modern terminal	R0	T	8433	C	ATP8	Non-synonymous	I23T	0.005	0.1	0.384
Modern terminal	R0	T	8433	C	ATP8	Non-synonymous	I23T	0.005	0.1	0.384
Pre-terminal	R0	T	8433	C	ATP8	Non-synonymous	I23T	0.005	0.1	0.384
Pre-terminal	R0	T	8433	C	ATP8	Non-synonymous	I23T	0.005	0.1	0.384
Ancient terminal	U	T	8433	G	ATP8	Non-synonymous	I23S	0.023	0	0.636
Modern terminal	U	T	8433	C	ATP8	Non-synonymous	I23T	0.005	0.1	0.384
Modern terminal	U	T	8433	G	ATP8	Non-synonymous	I23S	0.023	0	0.636
Pre-terminal	U	T	8433	C	ATP8	Non-synonymous	I23T	0.005	0.1	0.384
Modern terminal	JT	T	8433	C	ATP8	Non-synonymous	I23T	0.005	0.1	0.384
Modern terminal	JT	T	8433	C	ATP8	Non-synonymous	I23T	0.005	0.1	0.384
Modern terminal	JT	T	8433	C	ATP8	Non-synonymous	I23T	0.005	0.1	0.384
Modern terminal	R0	C	8434	G	ATP8	Non-synonymous	I23M	0.075	0.14	0.25
Modern terminal	R0	A	8435	G	ATP8	Non-synonymous	T24A	0.069	0.02	0.317
Modern terminal	R0	A	8435	G	ATP8	Non-synonymous	T24A	0.069	0.02	0.317
Pre-terminal	U	A	8435	G	ATP8	Non-synonymous	T24A	0.069	0.02	0.317
Pre-terminal	JT	A	8435	G	ATP8	Non-synonymous	T24A	0.069	0.02	0.317
Pre-terminal	JT	A	8435	G	ATP8	Non-synonymous	T24A	0.069	0.02	0.317
Modern terminal	N2	A	8435	G	ATP8	Non-synonymous	T24A	0.069	0.02	0.317
Modern terminal	JT	A	8439	C	ATP8	Non-synonymous	Q25P	0.931	0	0.755
Modern terminal	U	T	8442	C	ATP8	Non-synonymous	L26P	0.994	0.01	0.822
Modern terminal	JT	T	8442	C	ATP8	Non-synonymous	L26P	0.994	0.01	0.822

Ancient terminal	R0	T	8448	G	ATP8	Nonsense				
Pre-terminal	R0	T	8448	C	ATP8	Non-synonymous	M28T	0.004	0.1	0.234
Modern terminal	N1	T	8451	C	ATP8	Non-synonymous	L29S	0.994	0.09	0.172
Modern terminal	JT	A	8454	G	ATP8	Non-synonymous	N30S	0.955	0.42	0.151
Pre-terminal	JT	A	8454	G	ATP8	Non-synonymous	N30S	0.955	0.42	0.151
Modern terminal	R0	A	8460	G	ATP8	Non-synonymous	N32S	0.955	0.44	0.159
Modern terminal	R0	A	8460	G	ATP8	Non-synonymous	N32S	0.955	0.44	0.159
Modern terminal	R0	A	8460	G	ATP8	Non-synonymous	N32S	0.955	0.44	0.159
Pre-terminal	JT	A	8460	G	ATP8	Non-synonymous	N32S	0.955	0.44	0.159
Pre-terminal	JT	A	8460	G	ATP8	Non-synonymous	N32S	0.955	0.44	0.159
Modern terminal	N1	A	8460	G	ATP8	Non-synonymous	N32S	0.955	0.44	0.159
Modern terminal	R0	T	8462	C	ATP8	Non-synonymous	Y33H	0.994	0.01	0.491
Modern terminal	R0	T	8462	C	ATP8	Non-synonymous	Y33H	0.994	0.01	0.491
Pre-terminal	R0	T	8462	C	ATP8	Non-synonymous	Y33H	0.994	0.01	0.491
Modern terminal	U	T	8462	C	ATP8	Non-synonymous	Y33H	0.994	0.01	0.491
Modern terminal	U	T	8462	C	ATP8	Non-synonymous	Y33H	0.994	0.01	0.491
Modern terminal	JT	T	8462	C	ATP8	Non-synonymous	Y33H	0.994	0.01	0.491
Pre-terminal	JT	T	8462	C	ATP8	Non-synonymous	Y33H	0.994	0.01	0.491
Ancient terminal	R0	A	8463	T	ATP8	Non-synonymous	Y33F	0.955	0.54	0.183
Modern terminal	JT	A	8463	G	ATP8	Non-synonymous	Y33C	0.999	0	0.615
Pre-terminal	X	A	8463	G	ATP8	Non-synonymous	Y33C	0.999	0	0.615
Modern terminal	JT	C	8465	T	ATP8	Non-synonymous	H34Y	0.847	1	0.195
Modern terminal	U	A	8466	G	ATP8	Non-synonymous	H34R	0.931	0.34	0.242
Modern terminal	JT	A	8466	C	ATP8	Non-synonymous	H34P	0.979	0.27	0.313
Modern terminal	U	T	8469	G	ATP8	Non-synonymous	L35R	0.994	0.5	0.346
Ancient terminal	JT	T	8469	C	ATP8	Non-synonymous	L35P	0.994	0.29	0.303
Pre-terminal	N1	T	8469	C	ATP8	Non-synonymous	L35P	0.994	0.29	0.303
Ancient terminal	R0	C	8472	T	ATP8	Non-synonymous	P36L	0.004	0.26	0.31
Ancient terminal	U	C	8474	A	ATP8	Non-synonymous	P37T	0.998	0.05	0.544
Modern terminal	R0	T	8477	C	ATP8	Non-synonymous	S38P	0.02	0.25	0.379
Modern terminal	R0	T	8477	C	ATP8	Non-synonymous	S38P	0.02	0.25	0.379
Modern terminal	R0	T	8477	C	ATP8	Non-synonymous	S38P	0.02	0.25	0.379
Pre-terminal	R0	T	8477	C	ATP8	Non-synonymous	S38P	0.02	0.25	0.379
Modern terminal	U	T	8477	C	ATP8	Non-synonymous	S38P	0.02	0.25	0.379
Pre-terminal	U	T	8477	C	ATP8	Non-synonymous	S38P	0.02	0.25	0.379
Modern terminal	JT	T	8477	A	ATP8	Non-synonymous	S38T	0	0.52	0.145
Modern terminal	JT	T	8477	C	ATP8	Non-synonymous	S38P	0.02	0.25	0.379
Modern terminal	JT	T	8477	C	ATP8	Non-synonymous	S38P	0.02	0.25	0.379
Ancient terminal	N1	T	8477	C	ATP8	Non-synonymous	S38P	0.02	0.25	0.379
Modern terminal	N1	T	8477	C	ATP8	Non-synonymous	S38P	0.02	0.25	0.379
Pre-terminal	N1	T	8477	C	ATP8	Non-synonymous	S38P	0.02	0.25	0.379
Modern terminal	X	T	8477	C	ATP8	Non-synonymous	S38P	0.02	0.25	0.379
Modern terminal	N2	T	8477	G	ATP8	Non-synonymous	S38A	0	0.67	0.149
Pre-terminal	R0	C	8478	T	ATP8	Non-synonymous	S38L	0	0.3	0.234
Modern terminal	U	C	8478	T	ATP8	Non-synonymous	S38L	0	0.3	0.234
Modern terminal	U	C	8478	T	ATP8	Non-synonymous	S38L	0	0.3	0.234
Ancient terminal	U	C	8480	G	ATP8	Non-synonymous	P39A	0.993	0.98	0.21
Modern terminal	R0	C	8481	T	ATP8	Non-synonymous	P39L	0.998	0.82	0.191
Modern terminal	R0	C	8481	T	ATP8	Non-synonymous	P39L	0.998	0.82	0.191
Pre-terminal	R0	C	8481	T	ATP8	Non-synonymous	P39L	0.998	0.82	0.191

Ancient terminal	U	C	8481	T	ATP8	Non-synonymous	P39L	0.998	0.82	0.191
Ancient terminal	U	C	8481	T	ATP8	Non-synonymous	P39L	0.998	0.82	0.191
Modern terminal	R0	A	8483	G	ATP8	Non-synonymous	K40E	0.266	0.19	0.324
Modern terminal	R0	A	8483	G	ATP8	Non-synonymous	K40E	0.266	0.19	0.324
Ancient terminal	U	A	8483	G	ATP8	Non-synonymous	K40E	0.266	0.19	0.324
Modern terminal	JT	A	8483	G	ATP8	Non-synonymous	K40E	0.266	0.19	0.324
Modern terminal	U	A	8484	C	ATP8	Non-synonymous	K40T	0.152	0.19	0.368
Modern terminal	U	A	8484	C	ATP8	Non-synonymous	K40T	0.152	0.19	0.368
Modern terminal	R0	G	8485	T	ATP8	Non-synonymous	K40N	0.266	0.11	0.347
Ancient terminal	JT	G	8485	C	ATP8	Non-synonymous	K40N	0.266	0.11	0.347
Modern terminal	X	G	8485	C	ATP8	Non-synonymous	K40N	0.266	0.11	0.347
Ancient terminal	U	C	8487	T	ATP8	Non-synonymous	P41L	0.017	0.67	0.13
Modern terminal	R0	A	8489	C	ATP8	Non-synonymous	M42L	0.163	0.63	0.084
Ancient terminal	U	A	8489	G	ATP8	Non-synonymous	M42V	0.317	0.8	0.129
Pre-terminal	R0	T	8490	C	ATP8	Non-synonymous	M42T	0.711	1	0.107
Pre-terminal	U	T	8490	C	ATP8	Non-synonymous	M42T	0.711	1	0.107
Pre-terminal	U	T	8490	C	ATP8	Non-synonymous	M42T	0.711	1	0.107
Modern terminal	JT	T	8490	C	ATP8	Non-synonymous	M42T	0.711	1	0.107
Pre-terminal	R0	A	8492	G	ATP8	Non-synonymous	K43E	0.955	0.47	0.271
Ancient terminal	U	A	8492	T	ATP8	Nonsense				
Pre-terminal	JT	A	8492	G	ATP8	Non-synonymous	K43E	0.955	0.47	0.271
Ancient terminal	R0	A	8495	G	ATP8	Non-synonymous	M44V	0.317	0.51	0.098
Ancient terminal	U	A	8495	G	ATP8	Non-synonymous	M44V	0.317	0.51	0.098
Ancient terminal	U	A	8495	T	ATP8	Non-synonymous	M44L	0.163	0.71	0.067
Ancient terminal	R0	T	8496	G	ATP8	Nonsense				
Ancient terminal	R0	T	8496	A	ATP8	Non-synonymous	M44K	0.711	0.32	0.237
Ancient terminal	R0	T	8496	C	ATP8	Non-synonymous	M44T	0.711	0.5	0.074
Modern terminal	R0	T	8496	C	ATP8	Non-synonymous	M44T	0.711	0.5	0.074
Pre-terminal	R0	T	8496	C	ATP8	Non-synonymous	M44T	0.711	0.5	0.074
Ancient terminal	U	T	8496	A	ATP8	Non-synonymous	M44K	0.711	0.32	0.237
Pre-terminal	U	T	8496	C	ATP8	Non-synonymous	M44T	0.711	0.5	0.074
Ancient terminal	JT	T	8496	C	ATP8	Non-synonymous	M44T	0.711	0.5	0.074
Ancient terminal	JT	T	8496	G	ATP8	Nonsense				
Modern terminal	JT	T	8496	C	ATP8	Non-synonymous	M44T	0.711	0.5	0.074
Modern terminal	JT	T	8496	C	ATP8	Non-synonymous	M44T	0.711	0.5	0.074
Pre-terminal	JT	T	8496	C	ATP8	Non-synonymous	M44T	0.711	0.5	0.074
Ancient terminal	R0	A	8497	C	ATP8	Non-synonymous	M44I	0.518	0.42	0.106
Ancient terminal	R0	A	8497	C	ATP8	Non-synonymous	M44I	0.518	0.42	0.106
Ancient terminal	U	A	8497	T	ATP8	Non-synonymous	M44I	0.518	0.42	0.106
Ancient terminal	R0	A	8498	T	ATP8	Nonsense				
Ancient terminal	R0	A	8501	T	ATP8	Non-synonymous	N46Y	0.994	0.01	0.299
Modern terminal	U	A	8502	G	ATP8	Non-synonymous	N46S	0.955	0.21	0.126
Modern terminal	U	A	8502	T	ATP8	Non-synonymous	N46I	0.994	0.05	0.262
Ancient terminal	R0	T	8504	A	ATP8	Non-synonymous	Y47N	0.645	0.2	0.336
Modern terminal	R0	T	8504	C	ATP8	Non-synonymous	Y47H	0.645	1	0.1
Pre-terminal	R0	T	8504	C	ATP8	Non-synonymous	Y47H	0.645	1	0.1
Modern terminal	JT	T	8504	C	ATP8	Non-synonymous	Y47H	0.645	1	0.1
Pre-terminal	JT	T	8504	C	ATP8	Non-synonymous	Y47H	0.645	1	0.1
Modern terminal	X	A	8507	G	ATP8	Non-synonymous	N48D	0.087	0.65	0.162
Modern terminal	R0	A	8508	G	ATP8	Non-synonymous	N48S	0.007	0.84	0.096

Modern terminal	R0	A	8508	G	ATP8	Non-synonymous	N48S	0.007	0.84	0.096
Pre-terminal	R0	A	8508	G	ATP8	Non-synonymous	N48S	0.007	0.84	0.096
Pre-terminal	R0	A	8508	G	ATP8	Non-synonymous	N48S	0.007	0.84	0.096
Ancient terminal	JT	A	8508	G	ATP8	Non-synonymous	N48S	0.007	0.84	0.096
Modern terminal	JT	A	8508	T	ATP8	Non-synonymous	N48I	0.036	0.19	0.225
Pre-terminal	JT	A	8508	G	ATP8	Non-synonymous	N48S	0.007	0.84	0.096
Pre-terminal	JT	A	8508	G	ATP8	Non-synonymous	N48S	0.007	0.84	0.096
Pre-terminal	R0	C	8513	T	ATP8	Non-synonymous	P50S	0.993	0.02	0.284
Pre-terminal	U	C	8513	T	ATP8	Non-synonymous	P50S	0.993	0.02	0.284
Pre-terminal	JT	C	8513	T	ATP8	Non-synonymous	P50S	0.993	0.02	0.284
Pre-terminal	N2	C	8514	T	ATP8	Non-synonymous	P50L	0.998	0	0.386
Ancient terminal	R0	T	8516	A	ATP8	Nonsense				
Modern terminal	R0	T	8516	C	ATP8	Non-synonymous	W51R	0.99	0	0.737
Modern terminal	R0	T	8516	C	ATP8	Non-synonymous	W51R	0.99	0	0.737
Modern terminal	R0	T	8516	C	ATP8	Non-synonymous	W51R	0.99	0	0.737
Modern terminal	R0	T	8516	C	ATP8	Non-synonymous	W51R	0.99	0	0.737
Pre-terminal	R0	T	8516	C	ATP8	Non-synonymous	W51R	0.99	0	0.737
Ancient terminal	U	T	8516	A	ATP8	Nonsense				
Modern terminal	U	T	8516	C	ATP8	Non-synonymous	W51R	0.99	0	0.737
Modern terminal	N2	T	8516	C	ATP8	Non-synonymous	W51R	0.99	0	0.737
Modern terminal	U	G	8517	C	ATP8	Non-synonymous	W51S	0.99	0	0.721
Modern terminal	R0	G	8519	C	ATP8	Non-synonymous	E52Q	0.981	0.01	0.124
Modern terminal	R0	G	8519	C	ATP8	Non-synonymous	E52Q	0.981	0.01	0.124
Pre-terminal	R0	G	8519	A	ATP8	Non-synonymous	E52K	0.955	0.06	0.212
Modern terminal	U	G	8519	A	ATP8	Non-synonymous	E52K	0.955	0.06	0.212
Modern terminal	U	G	8519	A	ATP8	Non-synonymous	E52K	0.955	0.06	0.212
Pre-terminal	N1	G	8519	A	ATP8	Non-synonymous	E52K	0.955	0.06	0.212
Modern terminal	R0	A	8520	G	ATP8	Non-synonymous	E52G	0.981	0.01	0.264
Modern terminal	R0	A	8520	G	ATP8	Non-synonymous	E52G	0.981	0.01	0.264
Modern terminal	R0	A	8520	G	ATP8	Non-synonymous	E52G	0.981	0.01	0.264
Modern terminal	R0	A	8520	G	ATP8	Non-synonymous	E52G	0.981	0.01	0.264
Modern terminal	R0	A	8520	G	ATP8	Non-synonymous	E52G	0.981	0.01	0.264
Pre-terminal	R0	A	8520	G	ATP8	Non-synonymous	E52G	0.981	0.01	0.264
Pre-terminal	U	A	8520	G	ATP8	Non-synonymous	E52G	0.981	0.01	0.264
Modern terminal	JT	A	8520	G	ATP8	Non-synonymous	E52G	0.981	0.01	0.264
Modern terminal	JT	A	8520	G	ATP8	Non-synonymous	E52G	0.981	0.01	0.264
Modern terminal	R0	A	8521	C	ATP8	Non-synonymous	E52D	0.955	0.01	0.151
Modern terminal	R0	C	8522	T	ATP8	Non-synonymous	P53S	0	0.81	0.101
Pre-terminal	R0	C	8522	T	ATP8	Non-synonymous	P53S	0	0.81	0.101
Pre-terminal	R0	C	8522	T	ATP8	Non-synonymous	P53S	0	0.81	0.101
Pre-terminal	U	C	8522	T	ATP8	Non-synonymous	P53S	0	0.81	0.101
Modern terminal	R0	C	8523	T	ATP8	Non-synonymous	P53L	0.077	0.35	0.089
Modern terminal	R0	A	8527	G	ATP6	Non-synonymous	M1V	0.619	0	0.761
Pre-terminal	R0	A	8527	G	ATP6	Non-synonymous	M1V	0.619	0	0.761
Pre-terminal	R0	A	8527	G	ATP6	Non-synonymous	M1V	0.619	0	0.761
Modern terminal	R0	A	8530	G	ATP6	Non-synonymous	N2D	0.985	0.05	0.214
Pre-terminal	R0	A	8530	G	ATP6	Non-synonymous	N2D	0.985	0.05	0.214
Ancient terminal	U	A	8530	G	ATP6	Non-synonymous	N2D	0.985	0.05	0.214
Pre-terminal	JT	A	8530	G	ATP6	Non-synonymous	N2D	0.985	0.05	0.214
Modern terminal	R0	A	8531	G	ATP8	Non-synonymous	T56A	0.907	0.01	0.354

Modern terminal	U	A	8531	G	ATP8	Non-synonymous	T56A	0.907	0.01	0.354
Pre-terminal	U	A	8531	G	ATP8	Non-synonymous	T56A	0.907	0.01	0.354
Modern terminal	N1	A	8531	G	ATP8	Non-synonymous	T56A	0.907	0.01	0.354
Modern terminal	N2	A	8531	G	ATP8	Non-synonymous	T56A	0.907	0.01	0.354
Pre-terminal	N2	A	8531	G	ATP8	Non-synonymous	T56A	0.907	0.01	0.354
Pre-terminal	U	G	8533	A	ATP6	Non-synonymous	E3K	0.954	0.06	0.302
Ancient terminal	U	A	8534	T	ATP8	Nonsense				
Pre-terminal	R0	A	8536	G	ATP6	Non-synonymous	N4D	0.001	0.12	0.243
Modern terminal	R0	A	8537	G	ATP8	Non-synonymous	I58V	0.326	0.03	0.088
Pre-terminal	R0	A	8537	G	ATP8	Non-synonymous	I58V	0.326	0.03	0.088
Pre-terminal	R0	A	8537	G	ATP8	Non-synonymous	I58V	0.326	0.03	0.088
Modern terminal	R0	T	8538	C	ATP8	Non-synonymous	I58T	0.861	0.14	0.134
Modern terminal	R0	T	8538	C	ATP8	Non-synonymous	I58T	0.861	0.14	0.134
Pre-terminal	R0	T	8538	C	ATP8	Non-synonymous	I58T	0.861	0.14	0.134
Pre-terminal	R0	T	8538	C	ATP8	Non-synonymous	I58T	0.861	0.14	0.134
Modern terminal	U	T	8538	C	ATP8	Non-synonymous	I58T	0.861	0.14	0.134
Modern terminal	JT	T	8538	C	ATP8	Non-synonymous	I58T	0.861	0.14	0.134
Modern terminal	JT	T	8538	C	ATP8	Non-synonymous	I58T	0.861	0.14	0.134
Modern terminal	JT	T	8538	C	ATP8	Non-synonymous	I58T	0.861	0.14	0.134
Pre-terminal	JT	T	8538	C	ATP8	Non-synonymous	I58T	0.861	0.14	0.134
Modern terminal	R0	T	8540	C	ATP8	Non-synonymous	C59R	0.005	0	0.254
Modern terminal	R0	G	8541	A	ATP8	Non-synonymous	C59Y	0	1	0.05
Modern terminal	R0	G	8541	A	ATP8	Non-synonymous	C59Y	0	1	0.05
Modern terminal	R0	G	8541	A	ATP8	Non-synonymous	C59Y	0	1	0.05
Pre-terminal	R0	G	8541	A	ATP8	Non-synonymous	C59Y	0	1	0.05
Modern terminal	U	G	8541	A	ATP8	Non-synonymous	C59Y	0	1	0.05
Modern terminal	R0	T	8542	C	ATP6	Non-synonymous	F6L	0.992	0.03	0.313
Modern terminal	R0	T	8542	C	ATP6	Non-synonymous	F6L	0.992	0.03	0.313
Modern terminal	R0	T	8542	C	ATP6	Non-synonymous	F6L	0.992	0.03	0.313
Modern terminal	U	T	8542	C	ATP6	Non-synonymous	F6L	0.992	0.03	0.313
Modern terminal	U	T	8542	C	ATP6	Non-synonymous	F6L	0.992	0.03	0.313
Pre-terminal	U	T	8542	C	ATP6	Non-synonymous	F6L	0.992	0.03	0.313
Ancient terminal	U	C	8544	T	ATP8	Non-synonymous	S60L	0.93	1	0.042
Modern terminal	R0	G	8545	A	ATP6	Non-synonymous	A7T	0	0.98	0.198
Modern terminal	R0	G	8545	A	ATP6	Non-synonymous	A7T	0	0.98	0.198
Modern terminal	R0	G	8545	A	ATP6	Non-synonymous	A7T	0	0.98	0.198
Modern terminal	R0	G	8545	A	ATP6	Non-synonymous	A7T	0	0.98	0.198
Modern terminal	R0	G	8545	A	ATP6	Non-synonymous	A7T	0	0.98	0.198
Modern terminal	R0	G	8545	A	ATP6	Non-synonymous	A7T	0	0.98	0.198
Pre-terminal	R0	G	8545	A	ATP6	Non-synonymous	A7T	0	0.98	0.198
Ancient terminal	U	G	8545	A	ATP6	Non-synonymous	A7T	0	0.98	0.198
Modern terminal	U	G	8545	A	ATP6	Non-synonymous	A7T	0	0.98	0.198
Modern terminal	U	G	8545	A	ATP6	Non-synonymous	A7T	0	0.98	0.198
Modern terminal	U	G	8545	A	ATP6	Non-synonymous	A7T	0	0.98	0.198
Modern terminal	U	G	8545	A	ATP6	Non-synonymous	A7T	0	0.98	0.198
Modern terminal	JT	G	8545	A	ATP6	Non-synonymous	A7T	0	0.98	0.198
Pre-terminal	JT	G	8545	A	ATP6	Non-synonymous	A7T	0	0.98	0.198
Modern terminal	X	G	8545	A	ATP6	Non-synonymous	A7T	0	0.98	0.198
Modern terminal	JT	T	8547	C	ATP8	Non-synonymous	L61P	0.988	1	0.064
Modern terminal	N1	T	8547	C	ATP8	Non-synonymous	L61P	0.988	1	0.064

Pre-terminal	N1	T	8547	C	ATP8	Non-synonymous	L61P	0.988	1	0.064
Modern terminal	R0	T	8551	C	ATP6	Non-synonymous	F9L	0.997	0	0.418
Modern terminal	R0	T	8551	C	ATP6	Non-synonymous	F9L	0.997	0	0.418
Pre-terminal	R0	T	8551	C	ATP6	Non-synonymous	F9L	0.997	0	0.418
Modern terminal	U	T	8551	C	ATP6	Non-synonymous	F9L	0.997	0	0.418
Modern terminal	U	T	8551	C	ATP6	Non-synonymous	F9L	0.997	0	0.418
Pre-terminal	U	T	8551	C	ATP6	Non-synonymous	F9L	0.997	0	0.418
Modern terminal	JT	T	8551	C	ATP6	Non-synonymous	F9L	0.997	0	0.418
Modern terminal	N2	T	8551	C	ATP6	Non-synonymous	F9L	0.997	0	0.418
Modern terminal	R0	T	8552	C	ATP8	Non-synonymous	S63P	0.93	0.01	0.491
Pre-terminal	U	T	8552	C	ATP8	Non-synonymous	S63P	0.93	0.01	0.491
Ancient terminal	JT	T	8552	C	ATP8	Non-synonymous	S63P	0.93	0.01	0.491
Modern terminal	R0	C	8553	T	ATP8	Non-synonymous	S63L	0.93	0.05	0.224
Modern terminal	R0	C	8553	T	ATP8	Non-synonymous	S63L	0.93	0.05	0.224
Modern terminal	U	C	8553	T	ATP8	Non-synonymous	S63L	0.93	0.05	0.224
Modern terminal	JT	C	8553	T	ATP8	Non-synonymous	S63L	0.93	0.05	0.224
Modern terminal	R0	T	8555	C	ATP6	Non-synonymous	I10T	0	0.78	0.153
Ancient terminal	U	T	8555	C	ATP6	Non-synonymous	I10T	0	0.78	0.153
Modern terminal	JT	T	8555	C	ATP6	Non-synonymous	I10T	0	0.78	0.153
Modern terminal	JT	T	8555	C	ATP6	Non-synonymous	I10T	0	0.78	0.153
Modern terminal	R0	T	8556	C	ATP8	Non-synonymous	L64S	0.988	0.11	0.137
Modern terminal	R0	T	8556	C	ATP8	Non-synonymous	L64S	0.988	0.11	0.137
Ancient terminal	R0	G	8557	A	ATP6	Non-synonymous	A11T	0	0.65	0.047
Modern terminal	R0	G	8557	A	ATP6	Non-synonymous	A11T	0	0.65	0.047
Modern terminal	R0	G	8557	A	ATP6	Non-synonymous	A11T	0	0.65	0.047
Modern terminal	R0	G	8557	A	ATP6	Non-synonymous	A11T	0	0.65	0.047
Modern terminal	R0	G	8557	A	ATP6	Non-synonymous	A11T	0	0.65	0.047
Modern terminal	R0	G	8557	A	ATP6	Non-synonymous	A11T	0	0.65	0.047
Modern terminal	R0	G	8557	A	ATP6	Non-synonymous	A11T	0	0.65	0.047
Pre-terminal	R0	G	8557	A	ATP6	Non-synonymous	A11T	0	0.65	0.047
Pre-terminal	R0	G	8557	A	ATP6	Non-synonymous	A11T	0	0.65	0.047
Pre-terminal	R0	G	8557	A	ATP6	Non-synonymous	A11T	0	0.65	0.047
Modern terminal	U	G	8557	A	ATP6	Non-synonymous	A11T	0	0.65	0.047
Modern terminal	U	G	8557	A	ATP6	Non-synonymous	A11T	0	0.65	0.047
Modern terminal	U	G	8557	A	ATP6	Non-synonymous	A11T	0	0.65	0.047
Modern terminal	U	G	8557	A	ATP6	Non-synonymous	A11T	0	0.65	0.047
Modern terminal	U	G	8557	A	ATP6	Non-synonymous	A11T	0	0.65	0.047
Modern terminal	U	G	8557	A	ATP6	Non-synonymous	A11T	0	0.65	0.047
Modern terminal	U	G	8557	A	ATP6	Non-synonymous	A11T	0	0.65	0.047
Pre-terminal	U	G	8557	A	ATP6	Non-synonymous	A11T	0	0.65	0.047
Pre-terminal	U	G	8557	A	ATP6	Non-synonymous	A11T	0	0.65	0.047
Pre-terminal	U	G	8557	A	ATP6	Non-synonymous	A11T	0	0.65	0.047
Modern terminal	JT	G	8557	A	ATP6	Non-synonymous	A11T	0	0.65	0.047
Modern terminal	JT	G	8557	A	ATP6	Non-synonymous	A11T	0	0.65	0.047
Modern terminal	JT	G	8557	A	ATP6	Non-synonymous	A11T	0	0.65	0.047
Modern terminal	JT	G	8557	A	ATP6	Non-synonymous	A11T	0	0.65	0.047
Modern terminal	JT	G	8557	A	ATP6	Non-synonymous	A11T	0	0.65	0.047
Modern terminal	JT	G	8557	C	ATP8	Non-synonymous	L64F	0.988	0.02	0.117
Pre-terminal	N1	G	8557	A	ATP6	Non-synonymous	A11T	0	0.65	0.047
Pre-terminal	X	G	8557	A	ATP6	Non-synonymous	A11T	0	0.65	0.047

Pre-terminal	X	G	8557	A	ATP6	Non-synonymous	A11T	0	0.65	0.047
Modern terminal	N2	G	8557	A	ATP6	Non-synonymous	A11T	0	0.65	0.047
Pre-terminal	R0	C	8558	T	ATP8	Non-synonymous	P65S	0.985	0.01	0.145
Modern terminal	U	C	8558	T	ATP8	Non-synonymous	P65S	0.985	0.01	0.145
Pre-terminal	U	C	8558	T	ATP8	Non-synonymous	P65S	0.985	0.01	0.145
Modern terminal	JT	C	8558	T	ATP8	Non-synonymous	P65S	0.985	0.01	0.145
Modern terminal	U	C	8559	T	ATP8	Non-synonymous	P65L	0.996	0.01	0.154
Modern terminal	R0	C	8562	T	ATP8	Non-synonymous	P66L	0	1	0.051
Pre-terminal	R0	C	8562	T	ATP8	Non-synonymous	P66L	0	1	0.051
Modern terminal	JT	C	8562	T	ATP8	Non-synonymous	P66L	0	1	0.051
Pre-terminal	N1	C	8562	T	ATP8	Non-synonymous	P66L	0	1	0.051
Modern terminal	R0	A	8563	G	ATP6	Non-synonymous	T13A	0.978	0.22	0.267
Modern terminal	R0	A	8563	G	ATP6	Non-synonymous	T13A	0.978	0.22	0.267
Modern terminal	R0	A	8563	G	ATP6	Non-synonymous	T13A	0.978	0.22	0.267
Pre-terminal	R0	A	8563	G	ATP6	Non-synonymous	T13A	0.978	0.22	0.267
Pre-terminal	R0	A	8563	G	ATP6	Non-synonymous	T13A	0.978	0.22	0.267
Ancient terminal	U	A	8563	G	ATP6	Non-synonymous	T13A	0.978	0.22	0.267
Modern terminal	JT	A	8563	G	ATP6	Non-synonymous	T13A	0.978	0.22	0.267
Modern terminal	JT	A	8563	G	ATP6	Non-synonymous	T13A	0.978	0.22	0.267
Modern terminal	JT	A	8563	G	ATP6	Non-synonymous	T13A	0.978	0.22	0.267
Pre-terminal	JT	A	8563	T	ATP6	Non-synonymous	T13S	0.978	0.42	0.169
Modern terminal	N1	A	8563	G	ATP6	Non-synonymous	T13A	0.978	0.22	0.267
Modern terminal	N1	A	8563	G	ATP6	Non-synonymous	T13A	0.978	0.22	0.267
Pre-terminal	N2	A	8563	G	ATP6	Non-synonymous	T13A	0.978	0.22	0.267
Modern terminal	R0	A	8565	G	ATP8	Non-synonymous	Q67R	0	0.09	0.188
Modern terminal	R0	A	8566	G	ATP6	Non-synonymous	I14V	0.003	0.26	0.078
Modern terminal	R0	A	8566	G	ATP6	Non-synonymous	I14V	0.003	0.26	0.078
Modern terminal	R0	A	8566	G	ATP6	Non-synonymous	I14V	0.003	0.26	0.078
Pre-terminal	R0	A	8566	G	ATP6	Non-synonymous	I14V	0.003	0.26	0.078
Pre-terminal	R0	A	8566	G	ATP6	Non-synonymous	I14V	0.003	0.26	0.078
Modern terminal	U	A	8566	G	ATP6	Non-synonymous	I14V	0.003	0.26	0.078
Modern terminal	U	A	8566	G	ATP6	Non-synonymous	I14V	0.003	0.26	0.078
Modern terminal	U	A	8566	G	ATP6	Non-synonymous	I14V	0.003	0.26	0.078
Modern terminal	JT	A	8566	G	ATP6	Non-synonymous	I14V	0.003	0.26	0.078
Modern terminal	JT	A	8566	G	ATP6	Non-synonymous	I14V	0.003	0.26	0.078
Modern terminal	R0	T	8567	C	ATP8	Non-synonymous	S68P	0.93	0.01	0.357
Modern terminal	R0	T	8567	C	ATP8	Non-synonymous	S68P	0.93	0.01	0.357
Modern terminal	R0	T	8567	C	ATP8	Non-synonymous	S68P	0.93	0.01	0.357
Pre-terminal	R0	T	8567	C	ATP8	Non-synonymous	S68P	0.93	0.01	0.357
Pre-terminal	R0	T	8567	C	ATP8	Non-synonymous	S68P	0.93	0.01	0.357
Modern terminal	U	T	8567	C	ATP8	Non-synonymous	S68P	0.93	0.01	0.357
Modern terminal	U	T	8567	C	ATP8	Non-synonymous	S68P	0.93	0.01	0.357
Modern terminal	U	T	8567	C	ATP8	Non-synonymous	S68P	0.93	0.01	0.357
Pre-terminal	U	T	8567	C	ATP8	Non-synonymous	S68P	0.93	0.01	0.357
Pre-terminal	U	T	8567	C	ATP8	Non-synonymous	S68P	0.93	0.01	0.357
Modern terminal	JT	T	8567	C	ATP8	Non-synonymous	S68P	0.93	0.01	0.357
Modern terminal	JT	T	8567	C	ATP8	Non-synonymous	S68P	0.93	0.01	0.357
Modern terminal	JT	T	8567	C	ATP8	Non-synonymous	S68P	0.93	0.01	0.357
Modern terminal	N1	T	8567	C	ATP8	Non-synonymous	S68P	0.93	0.01	0.357
Pre-terminal	N1	T	8567	C	ATP8	Non-synonymous	S68P	0.93	0.01	0.357

Modern terminal	R0	C	8568	A	ATP8	Non-synonymous	S68Y	0.979	0	0.288
Pre-terminal	R0	C	8568	A	ATP8	Non-synonymous	S68Y	0.979	0	0.288
Modern terminal	U	C	8568	T	ATP8	Non-synonymous	S68F	0.979	0	0.299
Pre-terminal	U	C	8568	G	ATP8	Non-synonymous	S68C	0.994	0.01	0.209
Modern terminal	R0	G	8572	A	ATP6	Non-synonymous	G16S	0.846	0.02	0.439
Modern terminal	R0	G	8572	A	ATP6	Non-synonymous	G16S	0.846	0.02	0.439
Modern terminal	R0	G	8572	A	ATP6	Non-synonymous	G16S	0.846	0.02	0.439
Pre-terminal	R0	G	8572	A	ATP6	Non-synonymous	G16S	0.846	0.02	0.439
Pre-terminal	R0	G	8572	A	ATP6	Non-synonymous	G16S	0.846	0.02	0.439
Modern terminal	U	G	8572	A	ATP6	Non-synonymous	G16S	0.846	0.02	0.439
Modern terminal	U	G	8572	A	ATP6	Non-synonymous	G16S	0.846	0.02	0.439
Modern terminal	U	G	8572	A	ATP6	Non-synonymous	G16S	0.846	0.02	0.439
Pre-terminal	U	G	8572	A	ATP6	Non-synonymous	G16S	0.846	0.02	0.439
Pre-terminal	U	G	8572	A	ATP6	Non-synonymous	G16S	0.846	0.02	0.439
Pre-terminal	U	G	8572	A	ATP6	Non-synonymous	G16S	0.846	0.02	0.439
Pre-terminal	JT	G	8572	A	ATP6	Non-synonymous	G16S	0.846	0.02	0.439
Ancient terminal	X	G	8572	A	ATP6	Non-synonymous	G16S	0.846	0.02	0.439
Modern terminal	N2	G	8572	A	ATP6	Non-synonymous	G16S	0.846	0.02	0.439
Modern terminal	R0	G	8573	A	ATP6	Non-synonymous	G16D	0.068	0.01	0.665
Modern terminal	R0	G	8573	A	ATP6	Non-synonymous	G16D	0.068	0.01	0.665
Modern terminal	R0	G	8573	A	ATP6	Non-synonymous	G16D	0.068	0.01	0.665
Pre-terminal	R0	G	8573	A	ATP6	Non-synonymous	G16D	0.068	0.01	0.665
Pre-terminal	R0	G	8573	A	ATP6	Non-synonymous	G16D	0.068	0.01	0.665
Pre-terminal	R0	G	8573	A	ATP6	Non-synonymous	G16D	0.068	0.01	0.665
Ancient terminal	U	G	8573	A	ATP6	Non-synonymous	G16D	0.068	0.01	0.665
Ancient terminal	U	G	8573	A	ATP6	Non-synonymous	G16D	0.068	0.01	0.665
Modern terminal	U	G	8573	A	ATP6	Non-synonymous	G16D	0.068	0.01	0.665
Modern terminal	U	G	8573	A	ATP6	Non-synonymous	G16D	0.068	0.01	0.665
Modern terminal	JT	G	8573	A	ATP6	Non-synonymous	G16D	0.068	0.01	0.665
Modern terminal	JT	G	8573	A	ATP6	Non-synonymous	G16D	0.068	0.01	0.665
Modern terminal	N1	G	8573	A	ATP6	Non-synonymous	G16D	0.068	0.01	0.665
Pre-terminal	N1	G	8573	A	ATP6	Non-synonymous	G16D	0.068	0.01	0.665
Pre-terminal	N1	G	8573	A	ATP6	Non-synonymous	G16D	0.068	0.01	0.665
Modern terminal	JT	C	8575	A	ATP6	Non-synonymous	L17M	1	0.12	0.28
Modern terminal	R0	C	8578	T	ATP6	Non-synonymous	P18S	1	0.17	0.414
Pre-terminal	R0	C	8578	T	ATP6	Non-synonymous	P18S	1	0.17	0.414
Modern terminal	R0	G	8581	A	ATP6	Non-synonymous	A19T	0.999	0.24	0.156
Modern terminal	U	G	8581	A	ATP6	Non-synonymous	A19T	0.999	0.24	0.156
Modern terminal	U	G	8581	A	ATP6	Non-synonymous	A19T	0.999	0.24	0.156
Pre-terminal	JT	G	8581	A	ATP6	Non-synonymous	A19T	0.999	0.24	0.156
Pre-terminal	N1	G	8581	A	ATP6	Non-synonymous	A19T	0.999	0.24	0.156
Modern terminal	U	C	8582	T	ATP6	Non-synonymous	A19V	0.999	0.17	0.19
Modern terminal	R0	G	8584	A	ATP6	Non-synonymous	A20T	0.004	0.21	0.243
Modern terminal	R0	G	8584	A	ATP6	Non-synonymous	A20T	0.004	0.21	0.243
Modern terminal	R0	G	8584	A	ATP6	Non-synonymous	A20T	0.004	0.21	0.243
Modern terminal	R0	G	8584	A	ATP6	Non-synonymous	A20T	0.004	0.21	0.243
Modern terminal	R0	G	8584	A	ATP6	Non-synonymous	A20T	0.004	0.21	0.243
Pre-terminal	R0	G	8584	A	ATP6	Non-synonymous	A20T	0.004	0.21	0.243
Pre-terminal	R0	G	8584	A	ATP6	Non-synonymous	A20T	0.004	0.21	0.243
Pre-terminal	R0	G	8584	A	ATP6	Non-synonymous	A20T	0.004	0.21	0.243

Modern terminal	U	G	8584	A	ATP6	Non-synonymous	A20T	0.004	0.21	0.243
Modern terminal	U	G	8584	A	ATP6	Non-synonymous	A20T	0.004	0.21	0.243
Modern terminal	U	G	8584	A	ATP6	Non-synonymous	A20T	0.004	0.21	0.243
Modern terminal	U	G	8584	A	ATP6	Non-synonymous	A20T	0.004	0.21	0.243
Modern terminal	U	G	8584	A	ATP6	Non-synonymous	A20T	0.004	0.21	0.243
Pre-terminal	U	G	8584	A	ATP6	Non-synonymous	A20T	0.004	0.21	0.243
Pre-terminal	U	G	8584	A	ATP6	Non-synonymous	A20T	0.004	0.21	0.243
Ancient terminal	JT	G	8584	A	ATP6	Non-synonymous	A20T	0.004	0.21	0.243
Ancient terminal	JT	G	8584	A	ATP6	Non-synonymous	A20T	0.004	0.21	0.243
Modern terminal	JT	G	8584	A	ATP6	Non-synonymous	A20T	0.004	0.21	0.243
Modern terminal	JT	G	8584	A	ATP6	Non-synonymous	A20T	0.004	0.21	0.243
Modern terminal	JT	G	8584	A	ATP6	Non-synonymous	A20T	0.004	0.21	0.243
Modern terminal	JT	G	8584	A	ATP6	Non-synonymous	A20T	0.004	0.21	0.243
Modern terminal	R0	G	8587	A	ATP6	Non-synonymous	V21M	0.923	0.18	0.152
Ancient terminal	U	G	8587	A	ATP6	Non-synonymous	V21M	0.923	0.18	0.152
Modern terminal	U	G	8587	A	ATP6	Non-synonymous	V21M	0.923	0.18	0.152
Pre-terminal	U	G	8587	A	ATP6	Non-synonymous	V21M	0.923	0.18	0.152
Modern terminal	JT	G	8587	A	ATP6	Non-synonymous	V21M	0.923	0.18	0.152
Modern terminal	R0	T	8588	A	ATP6	Non-synonymous	V21E	0.987	0.07	0.524
Modern terminal	R0	T	8588	A	ATP6	Non-synonymous	V21E	0.987	0.07	0.524
Modern terminal	U	T	8588	C	ATP6	Non-synonymous	V21A	0.587	0.55	0.294
Modern terminal	U	T	8588	C	ATP6	Non-synonymous	V21A	0.587	0.55	0.294
Modern terminal	N1	T	8588	C	ATP6	Non-synonymous	V21A	0.587	0.55	0.294
Ancient terminal	JT	T	8591	C	ATP6	Non-synonymous	L22P	0.211	0.1	0.803
Modern terminal	JT	T	8591	C	ATP6	Non-synonymous	L22P	0.211	0.1	0.803
Pre-terminal	R0	A	8593	G	ATP6	Non-synonymous	I23V	0.003	1	0.167
Pre-terminal	R0	A	8593	G	ATP6	Non-synonymous	I23V	0.003	1	0.167
Modern terminal	R0	T	8594	C	ATP6	Non-synonymous	I23T	0.986	0.34	0.331
Modern terminal	R0	T	8594	C	ATP6	Non-synonymous	I23T	0.986	0.34	0.331
Pre-terminal	R0	T	8594	C	ATP6	Non-synonymous	I23T	0.986	0.34	0.331
Modern terminal	JT	T	8594	C	ATP6	Non-synonymous	I23T	0.986	0.34	0.331
Modern terminal	R0	A	8596	C	ATP6	Non-synonymous	I24L	0.762	0.52	0.274
Modern terminal	R0	A	8596	G	ATP6	Non-synonymous	I24V	0.916	0.31	0.091
Modern terminal	U	A	8596	G	ATP6	Non-synonymous	I24V	0.916	0.31	0.091
Pre-terminal	JT	A	8596	G	ATP6	Non-synonymous	I24V	0.916	0.31	0.091
Modern terminal	R0	C	8599	A	ATP6	Non-synonymous	L25M	1	0.48	0.081
Modern terminal	R0	T	8600	C	ATP6	Non-synonymous	L25P	1	0.02	0.608
Modern terminal	R0	T	8602	G	ATP6	Non-synonymous	F26V	0.13	0.25	0.43
Pre-terminal	R0	T	8602	C	ATP6	Non-synonymous	F26L	0.003	0.82	0.417
Pre-terminal	R0	T	8602	C	ATP6	Non-synonymous	F26L	0.003	0.82	0.417
Pre-terminal	R0	T	8602	C	ATP6	Non-synonymous	F26L	0.003	0.82	0.417
Pre-terminal	U	T	8602	C	ATP6	Non-synonymous	F26L	0.003	0.82	0.417
Ancient terminal	JT	T	8602	C	ATP6	Non-synonymous	F26L	0.003	0.82	0.417
Ancient terminal	JT	T	8602	C	ATP6	Non-synonymous	F26L	0.003	0.82	0.417
Pre-terminal	JT	T	8602	C	ATP6	Non-synonymous	F26L	0.003	0.82	0.417
Modern terminal	X	T	8602	C	ATP6	Non-synonymous	F26L	0.003	0.82	0.417
Modern terminal	X	T	8602	C	ATP6	Non-synonymous	F26L	0.003	0.82	0.417
Modern terminal	R0	T	8603	C	ATP6	Non-synonymous	F26S	0.982	0.1	0.706
Modern terminal	R0	T	8603	C	ATP6	Non-synonymous	F26S	0.982	0.1	0.706
Modern terminal	R0	T	8603	C	ATP6	Non-synonymous	F26S	0.982	0.1	0.706

Pre-terminal	R0	T	8603	C	ATP6	Non-synonymous	F26S	0.982	0.1	0.706
Pre-terminal	R0	T	8603	C	ATP6	Non-synonymous	F26S	0.982	0.1	0.706
Modern terminal	U	T	8603	C	ATP6	Non-synonymous	F26S	0.982	0.1	0.706
Modern terminal	N1	T	8603	C	ATP6	Non-synonymous	F26S	0.982	0.1	0.706
Modern terminal	R0	T	8604	A	ATP6	Non-synonymous	F26L	0.003	0.82	0.417
Modern terminal	R0	C	8605	T	ATP6	Non-synonymous	P27S	1	0.06	0.428
Modern terminal	U	C	8605	T	ATP6	Non-synonymous	P27S	1	0.06	0.428
Modern terminal	X	C	8605	A	ATP6	Non-synonymous	P27T	0.999	0.04	0.402
Ancient terminal	U	C	8608	T	ATP6	Non-synonymous	P28S	0.004	0.41	0.058
Pre-terminal	JT	C	8608	T	ATP6	Non-synonymous	P28S	0.004	0.41	0.058
Modern terminal	U	T	8615	C	ATP6	Non-synonymous	L30S	1	0	0.506
Modern terminal	N1	T	8615	C	ATP6	Non-synonymous	L30S	1	0	0.506
Pre-terminal	N1	G	8616	T	ATP6	Non-synonymous	L30F	0.999	0.08	0.343
Ancient terminal	U	A	8617	G	ATP6	Non-synonymous	I31V	0	0.19	0.058
Pre-terminal	U	A	8617	G	ATP6	Non-synonymous	I31V	0	0.19	0.058
Modern terminal	R0	T	8618	C	ATP6	Non-synonymous	I31T	0.081	0.1	0.198
Modern terminal	R0	T	8618	C	ATP6	Non-synonymous	I31T	0.081	0.1	0.198
Modern terminal	R0	T	8618	C	ATP6	Non-synonymous	I31T	0.081	0.1	0.198
Modern terminal	R0	T	8618	C	ATP6	Non-synonymous	I31T	0.081	0.1	0.198
Modern terminal	R0	T	8618	C	ATP6	Non-synonymous	I31T	0.081	0.1	0.198
Pre-terminal	R0	T	8618	C	ATP6	Non-synonymous	I31T	0.081	0.1	0.198
Pre-terminal	R0	T	8618	C	ATP6	Non-synonymous	I31T	0.081	0.1	0.198
Pre-terminal	R0	T	8618	C	ATP6	Non-synonymous	I31T	0.081	0.1	0.198
Pre-terminal	R0	T	8618	C	ATP6	Non-synonymous	I31T	0.081	0.1	0.198
Ancient terminal	U	T	8618	C	ATP6	Non-synonymous	I31T	0.081	0.1	0.198
Modern terminal	U	T	8618	C	ATP6	Non-synonymous	I31T	0.081	0.1	0.198
Pre-terminal	U	T	8618	C	ATP6	Non-synonymous	I31T	0.081	0.1	0.198
Modern terminal	JT	T	8618	C	ATP6	Non-synonymous	I31T	0.081	0.1	0.198
Modern terminal	JT	T	8618	C	ATP6	Non-synonymous	I31T	0.081	0.1	0.198
Modern terminal	JT	T	8618	C	ATP6	Non-synonymous	I31T	0.081	0.1	0.198
Modern terminal	N1	T	8618	C	ATP6	Non-synonymous	I31T	0.081	0.1	0.198
Pre-terminal	N1	T	8618	C	ATP6	Non-synonymous	I31T	0.081	0.1	0.198
Modern terminal	N2	T	8618	C	ATP6	Non-synonymous	I31T	0.081	0.1	0.198
Modern terminal	N2	T	8618	C	ATP6	Non-synonymous	I31T	0.081	0.1	0.198
Pre-terminal	N2	T	8618	C	ATP6	Non-synonymous	I31T	0.081	0.1	0.198
Pre-terminal	U	C	8619	A	ATP6	Non-synonymous	I31M	0.38	0.07	0.156
Modern terminal	R0	A	8623	G	ATP6	Non-synonymous	T33A	0	0.34	0.168
Modern terminal	R0	A	8623	G	ATP6	Non-synonymous	T33A	0	0.34	0.168
Modern terminal	R0	A	8623	G	ATP6	Non-synonymous	T33A	0	0.34	0.168
Pre-terminal	U	A	8623	G	ATP6	Non-synonymous	T33A	0	0.34	0.168
Modern terminal	R0	T	8626	A	ATP6	Non-synonymous	S34T	0.78	0.62	0.114
Modern terminal	R0	T	8626	C	ATP6	Non-synonymous	S34P	0.987	0.54	0.135
Ancient terminal	R0	A	8629	G	ATP6	Non-synonymous	K35E	0.669	0.92	0.254
Modern terminal	R0	T	8632	C	ATP6	Non-synonymous	Y36H	0	0.25	0.1
Modern terminal	R0	T	8632	C	ATP6	Non-synonymous	Y36H	0	0.25	0.1
Modern terminal	R0	T	8632	C	ATP6	Non-synonymous	Y36H	0	0.25	0.1
Pre-terminal	R0	T	8632	C	ATP6	Non-synonymous	Y36H	0	0.25	0.1
Modern terminal	U	T	8632	C	ATP6	Non-synonymous	Y36H	0	0.25	0.1
Modern terminal	U	T	8632	C	ATP6	Non-synonymous	Y36H	0	0.25	0.1
Modern terminal	U	T	8632	C	ATP6	Non-synonymous	Y36H	0	0.25	0.1

Modern terminal	U	T	8632	C	ATP6	Non-synonymous	Y36H	0	0.25	0.1
Pre-terminal	U	T	8632	C	ATP6	Non-synonymous	Y36H	0	0.25	0.1
Modern terminal	JT	T	8632	C	ATP6	Non-synonymous	Y36H	0	0.25	0.1
Pre-terminal	JT	T	8632	C	ATP6	Non-synonymous	Y36H	0	0.25	0.1
Modern terminal	JT	A	8633	G	ATP6	Non-synonymous	Y36C	0.935	0.05	0.134
Modern terminal	JT	A	8633	G	ATP6	Non-synonymous	Y36C	0.935	0.05	0.134
Pre-terminal	JT	A	8633	G	ATP6	Non-synonymous	Y36C	0.935	0.05	0.134
Modern terminal	R0	C	8635	A	ATP6	Non-synonymous	L37I	0.132	0.1	0.141
Modern terminal	N1	C	8635	T	ATP6	Non-synonymous	L37F	0.002	0.1	0.252
Modern terminal	R0	T	8636	C	ATP6	Non-synonymous	L37P	1	0.01	0.611
Modern terminal	R0	T	8636	C	ATP6	Non-synonymous	L37P	1	0.01	0.611
Pre-terminal	R0	T	8636	C	ATP6	Non-synonymous	L37P	1	0.01	0.611
Modern terminal	JT	T	8636	C	ATP6	Non-synonymous	L37P	1	0.01	0.611
Modern terminal	R0	A	8638	G	ATP6	Non-synonymous	I38V	0.689	0.28	0.069
Modern terminal	U	A	8638	C	ATP6	Non-synonymous	I38L	0.396	0.46	0.188
Pre-terminal	U	A	8638	G	ATP6	Non-synonymous	I38V	0.689	0.28	0.069
Modern terminal	N1	A	8638	G	ATP6	Non-synonymous	I38V	0.689	0.28	0.069
Pre-terminal	N1	A	8638	G	ATP6	Non-synonymous	I38V	0.689	0.28	0.069
Modern terminal	R0	T	8639	C	ATP6	Non-synonymous	I38T	0.999	0.01	0.203
Pre-terminal	R0	T	8639	C	ATP6	Non-synonymous	I38T	0.999	0.01	0.203
Pre-terminal	R0	T	8639	G	ATP6	Non-synonymous	I38S	1	0.05	0.336
Modern terminal	U	T	8639	C	ATP6	Non-synonymous	I38T	0.999	0.01	0.203
Modern terminal	U	T	8639	C	ATP6	Non-synonymous	I38T	0.999	0.01	0.203
Pre-terminal	U	T	8639	C	ATP6	Non-synonymous	I38T	0.999	0.01	0.203
Ancient terminal	JT	T	8639	C	ATP6	Non-synonymous	I38T	0.999	0.01	0.203
Modern terminal	X	T	8639	C	ATP6	Non-synonymous	I38T	0.999	0.01	0.203
Modern terminal	U	A	8642	G	ATP6	Non-synonymous	N39S	0.996	0.51	0.177
Pre-terminal	U	A	8642	G	ATP6	Non-synonymous	N39S	0.996	0.51	0.177
Modern terminal	JT	A	8642	G	ATP6	Non-synonymous	N39S	0.996	0.51	0.177
Pre-terminal	N1	A	8642	C	ATP6	Non-synonymous	N39T	0.984	0.44	0.246
Modern terminal	R0	A	8645	G	ATP6	Non-synonymous	N40S	0.996	0.11	0.222
Pre-terminal	R0	A	8645	G	ATP6	Non-synonymous	N40S	0.996	0.11	0.222
Pre-terminal	X	A	8645	G	ATP6	Non-synonymous	N40S	0.996	0.11	0.222
Modern terminal	R0	G	8648	A	ATP6	Non-synonymous	R41Q	0.999	0.07	0.268
Modern terminal	R0	G	8648	A	ATP6	Non-synonymous	R41Q	0.999	0.07	0.268
Modern terminal	R0	G	8648	A	ATP6	Non-synonymous	R41Q	0.999	0.07	0.268
Modern terminal	R0	G	8648	A	ATP6	Non-synonymous	R41Q	0.999	0.07	0.268
Pre-terminal	R0	G	8648	C	ATP6	Non-synonymous	R41P	1	0.03	0.675
Pre-terminal	R0	G	8648	A	ATP6	Non-synonymous	R41Q	0.999	0.07	0.268
Modern terminal	U	G	8648	A	ATP6	Non-synonymous	R41Q	0.999	0.07	0.268
Modern terminal	JT	G	8648	A	ATP6	Non-synonymous	R41Q	0.999	0.07	0.268
Pre-terminal	JT	G	8648	A	ATP6	Non-synonymous	R41Q	0.999	0.07	0.268
Pre-terminal	JT	G	8648	A	ATP6	Non-synonymous	R41Q	0.999	0.07	0.268
Modern terminal	X	G	8648	A	ATP6	Non-synonymous	R41Q	0.999	0.07	0.268
Modern terminal	R0	T	8651	C	ATP6	Non-synonymous	L42P	1	0.02	0.767
Pre-terminal	R0	A	8653	G	ATP6	Non-synonymous	I43V	0.003	1	0.068
Pre-terminal	JT	A	8653	G	ATP6	Non-synonymous	I43V	0.003	1	0.068
Modern terminal	R0	T	8654	C	ATP6	Non-synonymous	I43T	0.986	0.51	0.275
Modern terminal	R0	T	8654	C	ATP6	Non-synonymous	I43T	0.986	0.51	0.275
Modern terminal	R0	T	8654	C	ATP6	Non-synonymous	I43T	0.986	0.51	0.275

Ancient terminal	U	T	8654	C	ATP6	Non-synonymous	I43T	0.986	0.51	0.275
Modern terminal	U	T	8654	C	ATP6	Non-synonymous	I43T	0.986	0.51	0.275
Modern terminal	JT	T	8654	C	ATP6	Non-synonymous	I43T	0.986	0.51	0.275
Modern terminal	JT	T	8654	C	ATP6	Non-synonymous	I43T	0.986	0.51	0.275
Ancient terminal	R0	C	8655	A	ATP6	Non-synonymous	I43M	0.99	0.15	0.208
Pre-terminal	R0	A	8656	G	ATP6	Non-synonymous	T44A	0	0.12	0.11
Ancient terminal	U	A	8656	C	ATP6	Non-synonymous	T44P	0.872	0.02	0.314
Pre-terminal	JT	A	8656	G	ATP6	Non-synonymous	T44A	0	0.12	0.11
Modern terminal	R0	C	8657	T	ATP6	Non-synonymous	T44I	0	0.18	0.209
Ancient terminal	U	C	8657	A	ATP6	Non-synonymous	T44N	0.766	0.02	0.202
Modern terminal	R0	A	8659	G	ATP6	Non-synonymous	T45A	0.001	0.17	0.1
Modern terminal	R0	A	8659	G	ATP6	Non-synonymous	T45A	0.001	0.17	0.1
Pre-terminal	R0	A	8659	G	ATP6	Non-synonymous	T45A	0.001	0.17	0.1
Ancient terminal	U	A	8659	G	ATP6	Non-synonymous	T45A	0.001	0.17	0.1
Modern terminal	U	A	8659	G	ATP6	Non-synonymous	T45A	0.001	0.17	0.1
Modern terminal	JT	A	8659	G	ATP6	Non-synonymous	T45A	0.001	0.17	0.1
Pre-terminal	JT	A	8659	G	ATP6	Non-synonymous	T45A	0.001	0.17	0.1
Ancient terminal	X	A	8659	G	ATP6	Non-synonymous	T45A	0.001	0.17	0.1
Modern terminal	JT	A	8666	G	ATP6	Non-synonymous	Q47R	0.001	0.59	0.337
Pre-terminal	JT	A	8666	G	ATP6	Non-synonymous	Q47R	0.001	0.59	0.337
Modern terminal	R0	T	8668	C	ATP6	Non-synonymous	W48R	1	0.14	0.762
Modern terminal	U	T	8668	C	ATP6	Non-synonymous	W48R	1	0.14	0.762
Pre-terminal	U	T	8668	C	ATP6	Non-synonymous	W48R	1	0.14	0.762
Modern terminal	JT	T	8668	C	ATP6	Non-synonymous	W48R	1	0.14	0.762
Modern terminal	R0	A	8674	G	ATP6	Non-synonymous	I50V	0.079	0.28	0.052
Modern terminal	R0	A	8674	G	ATP6	Non-synonymous	I50V	0.079	0.28	0.052
Modern terminal	U	A	8674	G	ATP6	Non-synonymous	I50V	0.079	0.28	0.052
Modern terminal	U	A	8674	G	ATP6	Non-synonymous	I50V	0.079	0.28	0.052
Modern terminal	R0	T	8675	C	ATP6	Non-synonymous	I50T	0.087	0.14	0.185
Ancient terminal	R0	A	8677	C	ATP6	Non-synonymous	K51Q	0	0.89	0.048
Modern terminal	U	A	8677	C	ATP6	Non-synonymous	K51Q	0	0.89	0.048
Modern terminal	U	A	8679	C	ATP6	Non-synonymous	K51N	0.031	0.71	0.116
Pre-terminal	U	A	8679	C	ATP6	Non-synonymous	K51N	0.031	0.71	0.116
Modern terminal	R0	T	8681	G	ATP6	Non-synonymous	L52R	0.011	0.34	0.538
Modern terminal	R0	T	8681	C	ATP6	Non-synonymous	L52P	1	0.06	0.679
Modern terminal	R0	A	8683	G	ATP6	Non-synonymous	T53A	0.003	0.14	0.116
Modern terminal	U	A	8683	G	ATP6	Non-synonymous	T53A	0.003	0.14	0.116
Modern terminal	U	A	8683	G	ATP6	Non-synonymous	T53A	0.003	0.14	0.116
Ancient terminal	U	C	8684	A	ATP6	Non-synonymous	T53N	0.98	0.06	0.173
Modern terminal	JT	C	8684	T	ATP6	Non-synonymous	T53I	0.007	1	0.116
Modern terminal	JT	A	8689	G	ATP6	Non-synonymous	K55E	0.998	0.01	0.206
Ancient terminal	JT	A	8693	G	ATP6	Non-synonymous	Q56R	0.964	0	0.236
Modern terminal	R0	T	8696	C	ATP6	Non-synonymous	M57T	0.995	0	0.312
Modern terminal	R0	T	8696	C	ATP6	Non-synonymous	M57T	0.995	0	0.312
Modern terminal	U	T	8696	C	ATP6	Non-synonymous	M57T	0.995	0	0.312
Ancient terminal	JT	T	8696	C	ATP6	Non-synonymous	M57T	0.995	0	0.312
Modern terminal	JT	T	8696	C	ATP6	Non-synonymous	M57T	0.995	0	0.312
Modern terminal	R0	T	8699	C	ATP6	Non-synonymous	M58T	0.995	0.07	0.25
Pre-terminal	R0	T	8699	C	ATP6	Non-synonymous	M58T	0.995	0.07	0.25
Modern terminal	U	T	8699	C	ATP6	Non-synonymous	M58T	0.995	0.07	0.25

Ancient terminal	R0	A	8701	G	ATP6	Non-synonymous	T59A	0.002	0.66	0.085
Modern terminal	R0	A	8701	G	ATP6	Non-synonymous	T59A	0.002	0.66	0.085
Modern terminal	R0	A	8701	G	ATP6	Non-synonymous	T59A	0.002	0.66	0.085
Modern terminal	R0	A	8701	G	ATP6	Non-synonymous	T59A	0.002	0.66	0.085
Modern terminal	R0	A	8701	G	ATP6	Non-synonymous	T59A	0.002	0.66	0.085
Modern terminal	R0	A	8701	G	ATP6	Non-synonymous	T59A	0.002	0.66	0.085
Pre-terminal	R0	A	8701	G	ATP6	Non-synonymous	T59A	0.002	0.66	0.085
Pre-terminal	R0	A	8701	G	ATP6	Non-synonymous	T59A	0.002	0.66	0.085
Pre-terminal	R0	A	8701	G	ATP6	Non-synonymous	T59A	0.002	0.66	0.085
Modern terminal	U	A	8701	G	ATP6	Non-synonymous	T59A	0.002	0.66	0.085
Modern terminal	U	A	8701	G	ATP6	Non-synonymous	T59A	0.002	0.66	0.085
Modern terminal	U	A	8701	T	ATP6	Non-synonymous	T59S	0.25	0.68	0.058
Pre-terminal	U	A	8701	G	ATP6	Non-synonymous	T59A	0.002	0.66	0.085
Pre-terminal	U	A	8701	G	ATP6	Non-synonymous	T59A	0.002	0.66	0.085
Modern terminal	JT	A	8701	G	ATP6	Non-synonymous	T59A	0.002	0.66	0.085
Modern terminal	JT	A	8701	G	ATP6	Non-synonymous	T59A	0.002	0.66	0.085
Modern terminal	JT	A	8701	G	ATP6	Non-synonymous	T59A	0.002	0.66	0.085
Pre-terminal	JT	A	8701	G	ATP6	Non-synonymous	T59A	0.002	0.66	0.085
Pre-terminal	JT	A	8701	G	ATP6	Non-synonymous	T59A	0.002	0.66	0.085
Modern terminal	N1	A	8701	G	ATP6	Non-synonymous	T59A	0.002	0.66	0.085
Modern terminal	N1	A	8701	G	ATP6	Non-synonymous	T59A	0.002	0.66	0.085
Modern terminal	R0	C	8702	T	ATP6	Non-synonymous	T59I	0.005	0.44	0.176
Modern terminal	R0	C	8702	T	ATP6	Non-synonymous	T59I	0.005	0.44	0.176
Modern terminal	R0	C	8702	A	ATP6	Non-synonymous	T59N	0.92	0.41	0.141
Modern terminal	R0	C	8702	T	ATP6	Non-synonymous	T59I	0.005	0.44	0.176
Modern terminal	U	C	8702	T	ATP6	Non-synonymous	T59I	0.005	0.44	0.176
Pre-terminal	U	C	8702	T	ATP6	Non-synonymous	T59I	0.005	0.44	0.176
Pre-terminal	R0	A	8704	G	ATP6	Non-synonymous	M60V	0	1	0.116
Pre-terminal	R0	A	8704	G	ATP6	Non-synonymous	M60V	0	1	0.116
Modern terminal	U	A	8704	G	ATP6	Non-synonymous	M60V	0	1	0.116
Pre-terminal	JT	A	8704	C	ATP6	Non-synonymous	M60L	0	0.97	0.112
Modern terminal	N2	A	8704	G	ATP6	Non-synonymous	M60V	0	1	0.116
Modern terminal	R0	T	8705	C	ATP6	Non-synonymous	M60T	0	0.3	0.121
Modern terminal	R0	T	8705	C	ATP6	Non-synonymous	M60T	0	0.3	0.121
Modern terminal	R0	T	8705	C	ATP6	Non-synonymous	M60T	0	0.3	0.121
Modern terminal	R0	T	8705	C	ATP6	Non-synonymous	M60T	0	0.3	0.121
Modern terminal	R0	T	8705	C	ATP6	Non-synonymous	M60T	0	0.3	0.121
Pre-terminal	R0	T	8705	C	ATP6	Non-synonymous	M60T	0	0.3	0.121
Pre-terminal	R0	T	8705	C	ATP6	Non-synonymous	M60T	0	0.3	0.121
Pre-terminal	R0	T	8705	C	ATP6	Non-synonymous	M60T	0	0.3	0.121
Pre-terminal	R0	T	8705	C	ATP6	Non-synonymous	M60T	0	0.3	0.121
Ancient terminal	U	T	8705	C	ATP6	Non-synonymous	M60T	0	0.3	0.121
Modern terminal	U	T	8705	C	ATP6	Non-synonymous	M60T	0	0.3	0.121
Modern terminal	U	T	8705	C	ATP6	Non-synonymous	M60T	0	0.3	0.121
Modern terminal	U	T	8705	C	ATP6	Non-synonymous	M60T	0	0.3	0.121
Pre-terminal	U	T	8705	C	ATP6	Non-synonymous	M60T	0	0.3	0.121
Pre-terminal	U	T	8705	C	ATP6	Non-synonymous	M60T	0	0.3	0.121
Pre-terminal	U	T	8705	C	ATP6	Non-synonymous	M60T	0	0.3	0.121
Pre-terminal	U	T	8705	C	ATP6	Non-synonymous	M60T	0	0.3	0.121
Pre-terminal	U	T	8705	C	ATP6	Non-synonymous	M60T	0	0.3	0.121

Pre-terminal	U	T	8705	C	ATP6	Non-synonymous	M60T	0	0.3	0.121
Pre-terminal	U	T	8705	C	ATP6	Non-synonymous	M60T	0	0.3	0.121
Pre-terminal	U	T	8705	C	ATP6	Non-synonymous	M60T	0	0.3	0.121
Modern terminal	JT	T	8705	C	ATP6	Non-synonymous	M60T	0	0.3	0.121
Modern terminal	JT	T	8705	C	ATP6	Non-synonymous	M60T	0	0.3	0.121
Modern terminal	JT	T	8705	C	ATP6	Non-synonymous	M60T	0	0.3	0.121
Modern terminal	N1	T	8705	C	ATP6	Non-synonymous	M60T	0	0.3	0.121
Modern terminal	X	T	8705	C	ATP6	Non-synonymous	M60T	0	0.3	0.121
Pre-terminal	X	T	8705	C	ATP6	Non-synonymous	M60T	0	0.3	0.121
Modern terminal	N1	A	8706	T	ATP6	Non-synonymous	M60I	0.004	0.67	0.045
Ancient terminal	R0	C	8707	T	ATP6	Non-synonymous	H61Y	0.983	0.02	0.316
Ancient terminal	JT	A	8711	G	ATP6	Non-synonymous	N62S	0.008	0.11	0.168
Modern terminal	JT	A	8711	G	ATP6	Non-synonymous	N62S	0.008	0.11	0.168
Pre-terminal	JT	A	8711	G	ATP6	Non-synonymous	N62S	0.008	0.11	0.168
Modern terminal	N1	A	8711	G	ATP6	Non-synonymous	N62S	0.008	0.11	0.168
Modern terminal	N1	A	8711	G	ATP6	Non-synonymous	N62S	0.008	0.11	0.168
Modern terminal	R0	A	8713	G	ATP6	Non-synonymous	T63A	0	0.64	0.075
Modern terminal	U	A	8713	G	ATP6	Non-synonymous	T63A	0	0.64	0.075
Pre-terminal	U	A	8713	G	ATP6	Non-synonymous	T63A	0	0.64	0.075
Modern terminal	N1	C	8714	T	ATP6	Non-synonymous	T63I	0.13	0.2	0.122
Modern terminal	N1	A	8717	T	ATP6	Non-synonymous	K64M	1	0.07	0.279
Modern terminal	R0	G	8720	C	ATP6	Non-synonymous	G65A	1	0.03	0.359
Modern terminal	R0	G	8720	A	ATP6	Non-synonymous	G65E	1	0	0.488
Modern terminal	R0	G	8723	A	ATP6	Non-synonymous	R66Q	0.021	0.51	0.052
Modern terminal	R0	G	8723	A	ATP6	Non-synonymous	R66Q	0.021	0.51	0.052
Modern terminal	R0	G	8723	A	ATP6	Non-synonymous	R66Q	0.021	0.51	0.052
Modern terminal	R0	G	8723	A	ATP6	Non-synonymous	R66Q	0.021	0.51	0.052
Pre-terminal	R0	G	8723	A	ATP6	Non-synonymous	R66Q	0.021	0.51	0.052
Pre-terminal	R0	G	8723	A	ATP6	Non-synonymous	R66Q	0.021	0.51	0.052
Pre-terminal	R0	G	8723	A	ATP6	Non-synonymous	R66Q	0.021	0.51	0.052
Ancient terminal	U	G	8723	A	ATP6	Non-synonymous	R66Q	0.021	0.51	0.052
Modern terminal	U	G	8723	A	ATP6	Non-synonymous	R66Q	0.021	0.51	0.052
Modern terminal	U	G	8723	A	ATP6	Non-synonymous	R66Q	0.021	0.51	0.052
Modern terminal	JT	G	8723	A	ATP6	Non-synonymous	R66Q	0.021	0.51	0.052
Modern terminal	JT	G	8723	A	ATP6	Non-synonymous	R66Q	0.021	0.51	0.052
Modern terminal	R0	A	8725	G	ATP6	Non-synonymous	T67A	0.006	0.11	0.209
Modern terminal	U	A	8725	G	ATP6	Non-synonymous	T67A	0.006	0.11	0.209
Modern terminal	U	A	8725	G	ATP6	Non-synonymous	T67A	0.006	0.11	0.209
Pre-terminal	U	A	8725	G	ATP6	Non-synonymous	T67A	0.006	0.11	0.209
Pre-terminal	U	A	8725	G	ATP6	Non-synonymous	T67A	0.006	0.11	0.209
Pre-terminal	U	A	8725	G	ATP6	Non-synonymous	T67A	0.006	0.11	0.209
Pre-terminal	JT	A	8725	G	ATP6	Non-synonymous	T67A	0.006	0.11	0.209
Modern terminal	R0	T	8728	C	ATP6	Non-synonymous	W68R	1	0.01	0.749
Modern terminal	U	A	8730	C	ATP6	Non-synonymous	W68C	1	0	0.724
Modern terminal	R0	C	8732	T	ATP6	Non-synonymous	S69F	0.999	0	0.201
Pre-terminal	JT	C	8734	A	ATP6	Non-synonymous	L70I	0.994	0.05	0.202
Pre-terminal	R0	T	8738	C	ATP6	Non-synonymous	M71T	0.995	0	0.371
Modern terminal	R0	G	8743	A	ATP6	Non-synonymous	V73M	0.001	0.68	0.061
Modern terminal	U	G	8743	A	ATP6	Non-synonymous	V73M	0.001	0.68	0.061
Modern terminal	U	G	8743	A	ATP6	Non-synonymous	V73M	0.001	0.68	0.061

Modern terminal	U	G	8743	A	ATP6	Non-synonymous	V73M	0.001	0.68	0.061
Modern terminal	U	G	8743	A	ATP6	Non-synonymous	V73M	0.001	0.68	0.061
Pre-terminal	R0	T	8744	G	ATP6	Non-synonymous	V73G	0.072	0	0.366
Ancient terminal	JT	T	8750	C	ATP6	Non-synonymous	L75S	1	0.03	0.651
Modern terminal	JT	T	8750	C	ATP6	Non-synonymous	L75S	1	0.03	0.651
Modern terminal	R0	A	8752	C	ATP6	Non-synonymous	I76L	0.762	0.66	0.376
Pre-terminal	R0	A	8752	G	ATP6	Non-synonymous	I76V	0.916	0.08	0.171
Modern terminal	JT	A	8752	G	ATP6	Non-synonymous	I76V	0.916	0.08	0.171
Pre-terminal	JT	A	8752	G	ATP6	Non-synonymous	I76V	0.916	0.08	0.171
Pre-terminal	R0	T	8756	C	ATP6	Non-synonymous	I77T	0	0.21	0.213
Pre-terminal	U	T	8756	C	ATP6	Non-synonymous	I77T	0	0.21	0.213
Modern terminal	N1	T	8756	C	ATP6	Non-synonymous	I77T	0	0.21	0.213
Pre-terminal	N1	T	8756	C	ATP6	Non-synonymous	I77T	0	0.21	0.213
Pre-terminal	U	A	8761	G	ATP6	Non-synonymous	I79V	0.916	0.02	0.153
Modern terminal	R0	T	8762	C	ATP6	Non-synonymous	I79T	1	0	0.32
Modern terminal	R0	T	8762	C	ATP6	Non-synonymous	I79T	1	0	0.32
Modern terminal	R0	T	8762	C	ATP6	Non-synonymous	I79T	1	0	0.32
Modern terminal	R0	T	8762	C	ATP6	Non-synonymous	I79T	1	0	0.32
Modern terminal	R0	G	8764	A	ATP6	Non-synonymous	A80T	0.001	0.26	0.241
Modern terminal	R0	G	8764	A	ATP6	Non-synonymous	A80T	0.001	0.26	0.241
Modern terminal	R0	G	8764	A	ATP6	Non-synonymous	A80T	0.001	0.26	0.241
Modern terminal	R0	G	8764	A	ATP6	Non-synonymous	A80T	0.001	0.26	0.241
Modern terminal	R0	G	8764	A	ATP6	Non-synonymous	A80T	0.001	0.26	0.241
Modern terminal	R0	G	8764	A	ATP6	Non-synonymous	A80T	0.001	0.26	0.241
Modern terminal	R0	G	8764	A	ATP6	Non-synonymous	A80T	0.001	0.26	0.241
Modern terminal	R0	G	8764	A	ATP6	Non-synonymous	A80T	0.001	0.26	0.241
Pre-terminal	R0	G	8764	A	ATP6	Non-synonymous	A80T	0.001	0.26	0.241
Pre-terminal	R0	G	8764	A	ATP6	Non-synonymous	A80T	0.001	0.26	0.241
Pre-terminal	R0	G	8764	A	ATP6	Non-synonymous	A80T	0.001	0.26	0.241
Pre-terminal	R0	G	8764	A	ATP6	Non-synonymous	A80T	0.001	0.26	0.241
Pre-terminal	R0	G	8764	A	ATP6	Non-synonymous	A80T	0.001	0.26	0.241
Pre-terminal	R0	G	8764	A	ATP6	Non-synonymous	A80T	0.001	0.26	0.241
Modern terminal	U	G	8764	A	ATP6	Non-synonymous	A80T	0.001	0.26	0.241
Modern terminal	U	G	8764	A	ATP6	Non-synonymous	A80T	0.001	0.26	0.241
Modern terminal	U	G	8764	A	ATP6	Non-synonymous	A80T	0.001	0.26	0.241
Modern terminal	U	G	8764	A	ATP6	Non-synonymous	A80T	0.001	0.26	0.241
Modern terminal	U	G	8764	A	ATP6	Non-synonymous	A80T	0.001	0.26	0.241
Pre-terminal	U	G	8764	A	ATP6	Non-synonymous	A80T	0.001	0.26	0.241
Pre-terminal	U	G	8764	A	ATP6	Non-synonymous	A80T	0.001	0.26	0.241
Pre-terminal	U	G	8764	A	ATP6	Non-synonymous	A80T	0.001	0.26	0.241
Modern terminal	JT	G	8764	A	ATP6	Non-synonymous	A80T	0.001	0.26	0.241
Modern terminal	JT	G	8764	A	ATP6	Non-synonymous	A80T	0.001	0.26	0.241
Modern terminal	JT	G	8764	A	ATP6	Non-synonymous	A80T	0.001	0.26	0.241
Pre-terminal	JT	G	8764	A	ATP6	Non-synonymous	A80T	0.001	0.26	0.241
Pre-terminal	JT	G	8764	A	ATP6	Non-synonymous	A80T	0.001	0.26	0.241
Modern terminal	N1	G	8764	A	ATP6	Non-synonymous	A80T	0.001	0.26	0.241
Pre-terminal	X	G	8764	A	ATP6	Non-synonymous	A80T	0.001	0.26	0.241
Modern terminal	R0	C	8765	T	ATP6	Non-synonymous	A80V	0.157	0.42	0.266
Modern terminal	R0	C	8765	T	ATP6	Non-synonymous	A80V	0.157	0.42	0.266
Pre-terminal	R0	C	8765	T	ATP6	Non-synonymous	A80V	0.157	0.42	0.266

Modern terminal	U	C	8765	T	ATP6	Non-synonymous	A80V	0.157	0.42	0.266
Modern terminal	JT	C	8765	T	ATP6	Non-synonymous	A80V	0.157	0.42	0.266
Pre-terminal	JT	C	8765	T	ATP6	Non-synonymous	A80V	0.157	0.42	0.266
Pre-terminal	N1	C	8768	T	ATP6	Non-synonymous	T81M	0.987	0.23	0.127
Modern terminal	U	A	8770	G	ATP6	Non-synonymous	T82A	0.995	0.14	0.226
Pre-terminal	R0	A	8774	G	ATP6	Non-synonymous	N83S	0.999	0	0.169
Modern terminal	JT	A	8774	G	ATP6	Non-synonymous	N83S	0.999	0	0.169
Pre-terminal	U	C	8776	A	ATP6	Non-synonymous	L84I	0.994	0.09	0.129
Modern terminal	JT	C	8776	A	ATP6	Non-synonymous	L84I	0.994	0.09	0.129
Modern terminal	JT	C	8776	A	ATP6	Non-synonymous	L84I	0.994	0.09	0.129
Modern terminal	JT	C	8779	T	ATP6	Non-synonymous	L85F	0.999	0	0.267
Modern terminal	R0	G	8783	A	ATP6	Non-synonymous	G86E	1	0	0.518
Modern terminal	U	T	8786	C	ATP6	Non-synonymous	L87P	1	0	0.718
Modern terminal	JT	C	8794	T	ATP6	Non-synonymous	H90Y	0.003	1	0.155
Ancient terminal	JT	T	8802	A	ATP6	Non-synonymous	F92L	0.999	0	0.218
Pre-terminal	R0	A	8803	G	ATP6	Non-synonymous	T93A	0.995	0.06	0.178
Pre-terminal	R0	A	8803	T	ATP6	Non-synonymous	T93S	0.995	0.01	0.105
Modern terminal	R0	C	8806	G	ATP6	Non-synonymous	P94A	0.999	0.02	0.199
Modern terminal	R0	C	8807	T	ATP6	Non-synonymous	P94L	1	0	0.276
Ancient terminal	U	A	8809	G	ATP6	Non-synonymous	T95A	0.995	0	0.234
Ancient terminal	R0	A	8812	G	ATP6	Non-synonymous	T96A	0.995	0.03	0.254
Modern terminal	R0	A	8812	G	ATP6	Non-synonymous	T96A	0.995	0.03	0.254
Modern terminal	R0	A	8812	G	ATP6	Non-synonymous	T96A	0.995	0.03	0.254
Modern terminal	R0	A	8812	G	ATP6	Non-synonymous	T96A	0.995	0.03	0.254
Modern terminal	R0	A	8812	G	ATP6	Non-synonymous	T96A	0.995	0.03	0.254
Modern terminal	R0	A	8812	G	ATP6	Non-synonymous	T96A	0.995	0.03	0.254
Pre-terminal	R0	A	8812	G	ATP6	Non-synonymous	T96A	0.995	0.03	0.254
Pre-terminal	R0	A	8812	G	ATP6	Non-synonymous	T96A	0.995	0.03	0.254
Ancient terminal	U	A	8812	G	ATP6	Non-synonymous	T96A	0.995	0.03	0.254
Pre-terminal	U	A	8812	G	ATP6	Non-synonymous	T96A	0.995	0.03	0.254
Pre-terminal	U	A	8812	T	ATP6	Non-synonymous	T96S	0.995	0.22	0.141
Pre-terminal	U	A	8812	G	ATP6	Non-synonymous	T96A	0.995	0.03	0.254
Modern terminal	JT	A	8812	G	ATP6	Non-synonymous	T96A	0.995	0.03	0.254
Modern terminal	JT	A	8812	G	ATP6	Non-synonymous	T96A	0.995	0.03	0.254
Pre-terminal	JT	A	8812	G	ATP6	Non-synonymous	T96A	0.995	0.03	0.254
Modern terminal	R0	C	8813	G	ATP6	Non-synonymous	T96S	0.995	0.22	0.141
Ancient terminal	U	A	8817	T	ATP6	Non-synonymous	Q97H	0.997	0.1	0.222
Modern terminal	R0	T	8821	C	ATP6	Non-synonymous	S99P	0.995	0	0.61
Modern terminal	R0	T	8821	C	ATP6	Non-synonymous	S99P	0.995	0	0.61
Pre-terminal	R0	T	8821	G	ATP6	Non-synonymous	S99A	0.941	0	0.243
Pre-terminal	R0	A	8824	G	ATP6	Non-synonymous	M100V	0.998	0.12	0.381
Modern terminal	U	T	8825	C	ATP6	Non-synonymous	M100T	0.995	0	0.444
Modern terminal	U	T	8825	C	ATP6	Non-synonymous	M100T	0.995	0	0.444
Modern terminal	N1	T	8825	C	ATP6	Non-synonymous	M100T	0.995	0	0.444
Modern terminal	JT	A	8827	G	ATP6	Non-synonymous	N101D	0.999	0.03	0.486
Pre-terminal	R0	A	8828	G	ATP6	Non-synonymous	N101S	0.999	0.01	0.349
Modern terminal	R0	G	8833	A	ATP6	Non-synonymous	A103T	0.999	0.07	0.384
Modern terminal	U	G	8833	A	ATP6	Non-synonymous	A103T	0.999	0.07	0.384
Pre-terminal	U	G	8833	A	ATP6	Non-synonymous	A103T	0.999	0.07	0.384
Modern terminal	JT	G	8833	A	ATP6	Non-synonymous	A103T	0.999	0.07	0.384

Modern terminal	N2	G	8833	A	ATP6	Non-synonymous	A103T	0.999	0.07	0.384
Pre-terminal	U	A	8836	G	ATP6	Non-synonymous	M104V	0.998	0.01	0.568
Modern terminal	R0	T	8837	C	ATP6	Non-synonymous	M104T	0.995	0	0.639
Modern terminal	R0	G	8839	A	ATP6	Non-synonymous	A105T	0.999	0	0.571
Modern terminal	R0	G	8839	A	ATP6	Non-synonymous	A105T	0.999	0	0.571
Modern terminal	R0	G	8839	A	ATP6	Non-synonymous	A105T	0.999	0	0.571
Modern terminal	R0	G	8839	A	ATP6	Non-synonymous	A105T	0.999	0	0.571
Modern terminal	R0	G	8839	A	ATP6	Non-synonymous	A105T	0.999	0	0.571
Pre-terminal	R0	G	8839	A	ATP6	Non-synonymous	A105T	0.999	0	0.571
Pre-terminal	R0	G	8839	A	ATP6	Non-synonymous	A105T	0.999	0	0.571
Pre-terminal	R0	G	8839	A	ATP6	Non-synonymous	A105T	0.999	0	0.571
Pre-terminal	R0	G	8839	A	ATP6	Non-synonymous	A105T	0.999	0	0.571
Pre-terminal	R0	G	8839	A	ATP6	Non-synonymous	A105T	0.999	0	0.571
Pre-terminal	R0	G	8839	A	ATP6	Non-synonymous	A105T	0.999	0	0.571
Modern terminal	U	G	8839	A	ATP6	Non-synonymous	A105T	0.999	0	0.571
Modern terminal	U	G	8839	A	ATP6	Non-synonymous	A105T	0.999	0	0.571
Modern terminal	U	G	8839	A	ATP6	Non-synonymous	A105T	0.999	0	0.571
Modern terminal	U	G	8839	A	ATP6	Non-synonymous	A105T	0.999	0	0.571
Modern terminal	U	G	8839	A	ATP6	Non-synonymous	A105T	0.999	0	0.571
Modern terminal	U	G	8839	A	ATP6	Non-synonymous	A105T	0.999	0	0.571
Modern terminal	JT	G	8839	A	ATP6	Non-synonymous	A105T	0.999	0	0.571
Modern terminal	JT	G	8839	A	ATP6	Non-synonymous	A105T	0.999	0	0.571
Modern terminal	JT	G	8839	A	ATP6	Non-synonymous	A105T	0.999	0	0.571
Modern terminal	JT	G	8839	A	ATP6	Non-synonymous	A105T	0.999	0	0.571
Modern terminal	JT	G	8839	A	ATP6	Non-synonymous	A105T	0.999	0	0.571
Modern terminal	JT	G	8839	A	ATP6	Non-synonymous	A105T	0.999	0	0.571
Pre-terminal	JT	G	8839	A	ATP6	Non-synonymous	A105T	0.999	0	0.571
Modern terminal	N1	G	8839	A	ATP6	Non-synonymous	A105T	0.999	0	0.571
Modern terminal	X	G	8839	A	ATP6	Non-synonymous	A105T	0.999	0	0.571
Ancient terminal	N2	G	8839	A	ATP6	Non-synonymous	A105T	0.999	0	0.571
Modern terminal	U	C	8840	T	ATP6	Non-synonymous	A105V	0.999	0	0.608
Modern terminal	U	C	8840	T	ATP6	Non-synonymous	A105V	0.999	0	0.608
Modern terminal	JT	C	8840	T	ATP6	Non-synonymous	A105V	0.999	0	0.608
Modern terminal	R0	A	8842	G	ATP6	Non-synonymous	I106V	0.003	0.45	0.236
Modern terminal	R0	A	8842	T	ATP6	Non-synonymous	I106F	0.848	0.25	0.664
Modern terminal	R0	A	8842	G	ATP6	Non-synonymous	I106V	0.003	0.45	0.236
Modern terminal	R0	A	8842	G	ATP6	Non-synonymous	I106V	0.003	0.45	0.236
Modern terminal	R0	A	8842	T	ATP6	Non-synonymous	I106F	0.848	0.25	0.664
Pre-terminal	R0	A	8842	G	ATP6	Non-synonymous	I106V	0.003	0.45	0.236
Modern terminal	U	A	8842	C	ATP6	Non-synonymous	I106L	0.025	0.18	0.475
Modern terminal	U	A	8842	G	ATP6	Non-synonymous	I106V	0.003	0.45	0.236
Pre-terminal	U	A	8842	G	ATP6	Non-synonymous	I106V	0.003	0.45	0.236
Pre-terminal	U	A	8842	G	ATP6	Non-synonymous	I106V	0.003	0.45	0.236
Pre-terminal	U	A	8842	C	ATP6	Non-synonymous	I106L	0.025	0.18	0.475
Pre-terminal	X	A	8842	G	ATP6	Non-synonymous	I106V	0.003	0.45	0.236
Modern terminal	R0	T	8843	C	ATP6	Non-synonymous	I106T	0.986	0	0.592
Modern terminal	R0	T	8843	C	ATP6	Non-synonymous	I106T	0.986	0	0.592
Modern terminal	R0	T	8843	C	ATP6	Non-synonymous	I106T	0.986	0	0.592
Pre-terminal	R0	T	8843	C	ATP6	Non-synonymous	I106T	0.986	0	0.592
Pre-terminal	R0	T	8843	C	ATP6	Non-synonymous	I106T	0.986	0	0.592

Pre-terminal	R0	T	8843	C	ATP6	Non-synonymous	I106T	0.986	0	0.592
Pre-terminal	R0	T	8843	C	ATP6	Non-synonymous	I106T	0.986	0	0.592
Pre-terminal	R0	T	8843	C	ATP6	Non-synonymous	I106T	0.986	0	0.592
Pre-terminal	R0	T	8843	C	ATP6	Non-synonymous	I106T	0.986	0	0.592
Pre-terminal	R0	T	8843	C	ATP6	Non-synonymous	I106T	0.986	0	0.592
Pre-terminal	R0	T	8843	C	ATP6	Non-synonymous	I106T	0.986	0	0.592
Ancient terminal	U	T	8843	C	ATP6	Non-synonymous	I106T	0.986	0	0.592
Ancient terminal	U	T	8843	C	ATP6	Non-synonymous	I106T	0.986	0	0.592
Modern terminal	U	T	8843	C	ATP6	Non-synonymous	I106T	0.986	0	0.592
Modern terminal	U	T	8843	C	ATP6	Non-synonymous	I106T	0.986	0	0.592
Modern terminal	U	T	8843	C	ATP6	Non-synonymous	I106T	0.986	0	0.592
Modern terminal	U	T	8843	C	ATP6	Non-synonymous	I106T	0.986	0	0.592
Pre-terminal	U	T	8843	C	ATP6	Non-synonymous	I106T	0.986	0	0.592
Pre-terminal	U	T	8843	C	ATP6	Non-synonymous	I106T	0.986	0	0.592
Pre-terminal	U	T	8843	C	ATP6	Non-synonymous	I106T	0.986	0	0.592
Modern terminal	JT	T	8843	C	ATP6	Non-synonymous	I106T	0.986	0	0.592
Modern terminal	JT	T	8843	C	ATP6	Non-synonymous	I106T	0.986	0	0.592
Modern terminal	JT	T	8843	C	ATP6	Non-synonymous	I106T	0.986	0	0.592
Modern terminal	JT	T	8843	C	ATP6	Non-synonymous	I106T	0.986	0	0.592
Modern terminal	JT	T	8843	C	ATP6	Non-synonymous	I106T	0.986	0	0.592
Modern terminal	JT	T	8843	C	ATP6	Non-synonymous	I106T	0.986	0	0.592
Modern terminal	JT	T	8843	C	ATP6	Non-synonymous	I106T	0.986	0	0.592
Pre-terminal	JT	T	8843	C	ATP6	Non-synonymous	I106T	0.986	0	0.592
Modern terminal	X	T	8843	C	ATP6	Non-synonymous	I106T	0.986	0	0.592
Modern terminal	N2	T	8843	C	ATP6	Non-synonymous	I106T	0.986	0	0.592
Modern terminal	JT	C	8844	A	ATP6	Non-synonymous	I106M	0.99	0.01	0.451
Ancient terminal	U	C	8845	T	ATP6	Non-synonymous	P107S	1	0.02	0.7
Modern terminal	R0	T	8851	C	ATP6	Non-synonymous	W109R	1	0	0.941
Modern terminal	R0	T	8851	C	ATP6	Non-synonymous	W109R	1	0	0.941
Modern terminal	JT	T	8851	C	ATP6	Non-synonymous	W109R	1	0	0.941
Modern terminal	R0	G	8854	A	ATP6	Non-synonymous	A110T	0.003	0.15	0.697
Modern terminal	R0	G	8854	A	ATP6	Non-synonymous	A110T	0.003	0.15	0.697
Modern terminal	U	G	8854	A	ATP6	Non-synonymous	A110T	0.003	0.15	0.697
Modern terminal	JT	G	8854	A	ATP6	Non-synonymous	A110T	0.003	0.15	0.697
Modern terminal	JT	G	8854	A	ATP6	Non-synonymous	A110T	0.003	0.15	0.697
Pre-terminal	JT	G	8854	A	ATP6	Non-synonymous	A110T	0.003	0.15	0.697
Pre-terminal	JT	G	8854	A	ATP6	Non-synonymous	A110T	0.003	0.15	0.697
Ancient terminal	X	G	8854	A	ATP6	Non-synonymous	A110T	0.003	0.15	0.697
Modern terminal	R0	C	8855	T	ATP6	Non-synonymous	A110V	0.483	0.09	0.721
Modern terminal	JT	C	8855	T	ATP6	Non-synonymous	A110V	0.483	0.09	0.721
Pre-terminal	N2	C	8855	T	ATP6	Non-synonymous	A110V	0.483	0.09	0.721
Modern terminal	R0	G	8857	A	ATP6	Non-synonymous	G111S	0.128	0.3	0.758
Modern terminal	R0	G	8857	A	ATP6	Non-synonymous	G111S	0.128	0.3	0.758
Pre-terminal	R0	G	8857	A	ATP6	Non-synonymous	G111S	0.128	0.3	0.758
Pre-terminal	R0	G	8857	A	ATP6	Non-synonymous	G111S	0.128	0.3	0.758
Pre-terminal	R0	G	8857	A	ATP6	Non-synonymous	G111S	0.128	0.3	0.758
Modern terminal	U	G	8857	A	ATP6	Non-synonymous	G111S	0.128	0.3	0.758
Modern terminal	U	G	8857	A	ATP6	Non-synonymous	G111S	0.128	0.3	0.758
Modern terminal	U	G	8857	A	ATP6	Non-synonymous	G111S	0.128	0.3	0.758
Modern terminal	U	G	8857	A	ATP6	Non-synonymous	G111S	0.128	0.3	0.758

Pre-terminal	U	G	8857	A	ATP6	Non-synonymous	G111S	0.128	0.3	0.758
Pre-terminal	U	G	8857	A	ATP6	Non-synonymous	G111S	0.128	0.3	0.758
Modern terminal	JT	G	8857	A	ATP6	Non-synonymous	G111S	0.128	0.3	0.758
Modern terminal	JT	G	8857	A	ATP6	Non-synonymous	G111S	0.128	0.3	0.758
Modern terminal	JT	G	8857	A	ATP6	Non-synonymous	G111S	0.128	0.3	0.758
Pre-terminal	JT	G	8857	A	ATP6	Non-synonymous	G111S	0.128	0.3	0.758
Modern terminal	N2	G	8857	A	ATP6	Non-synonymous	G111S	0.128	0.3	0.758
Modern terminal	N2	G	8857	A	ATP6	Non-synonymous	G111S	0.128	0.3	0.758
Ancient terminal	U	G	8858	T	ATP6	Non-synonymous	G111V	0.995	0.07	0.888
Modern terminal	R0	C	8861	T	ATP6	Non-synonymous	T112M	0.557	0	0.433
Pre-terminal	U	C	8861	T	ATP6	Non-synonymous	T112M	0.557	0	0.433
Pre-terminal	U	C	8861	T	ATP6	Non-synonymous	T112M	0.557	0	0.433
Modern terminal	R0	G	8863	A	ATP6	Non-synonymous	V113M	0.998	0.13	0.619
Modern terminal	U	G	8863	A	ATP6	Non-synonymous	V113M	0.998	0.13	0.619
Modern terminal	JT	G	8863	A	ATP6	Non-synonymous	V113M	0.998	0.13	0.619
Modern terminal	JT	G	8863	A	ATP6	Non-synonymous	V113M	0.998	0.13	0.619
Modern terminal	JT	G	8863	A	ATP6	Non-synonymous	V113M	0.998	0.13	0.619
Pre-terminal	JT	G	8863	A	ATP6	Non-synonymous	V113M	0.998	0.13	0.619
Modern terminal	R0	T	8864	C	ATP6	Non-synonymous	V113A	1	0	0.649
Modern terminal	R0	T	8864	C	ATP6	Non-synonymous	V113A	1	0	0.649
Modern terminal	R0	T	8864	C	ATP6	Non-synonymous	V113A	1	0	0.649
Pre-terminal	R0	T	8864	C	ATP6	Non-synonymous	V113A	1	0	0.649
Pre-terminal	R0	T	8864	C	ATP6	Non-synonymous	V113A	1	0	0.649
Modern terminal	U	T	8864	C	ATP6	Non-synonymous	V113A	1	0	0.649
Modern terminal	U	T	8864	C	ATP6	Non-synonymous	V113A	1	0	0.649
Pre-terminal	JT	T	8864	C	ATP6	Non-synonymous	V113A	1	0	0.649
Modern terminal	U	A	8866	G	ATP6	Non-synonymous	I114V	0	0.31	0.173
Pre-terminal	R0	T	8867	C	ATP6	Non-synonymous	I114T	0.001	0.04	0.521
Ancient terminal	U	T	8867	C	ATP6	Non-synonymous	I114T	0.001	0.04	0.521
Pre-terminal	U	T	8867	C	ATP6	Non-synonymous	I114T	0.001	0.04	0.521
Modern terminal	R0	A	8869	C	ATP6	Non-synonymous	M115L	0.001	1	0.16
Modern terminal	R0	A	8869	G	ATP6	Non-synonymous	M115V	0.002	0.51	0.287
Ancient terminal	U	A	8869	G	ATP6	Non-synonymous	M115V	0.002	0.51	0.287
Modern terminal	R0	T	8870	C	ATP6	Non-synonymous	M115T	0	0.31	0.118
Modern terminal	R0	T	8870	C	ATP6	Non-synonymous	M115T	0	0.31	0.118
Modern terminal	R0	T	8870	C	ATP6	Non-synonymous	M115T	0	0.31	0.118
Modern terminal	R0	T	8870	C	ATP6	Non-synonymous	M115T	0	0.31	0.118
Modern terminal	R0	T	8870	C	ATP6	Non-synonymous	M115T	0	0.31	0.118
Ancient terminal	U	T	8870	C	ATP6	Non-synonymous	M115T	0	0.31	0.118
Modern terminal	U	T	8870	C	ATP6	Non-synonymous	M115T	0	0.31	0.118
Modern terminal	U	T	8870	C	ATP6	Non-synonymous	M115T	0	0.31	0.118
Modern terminal	U	T	8870	C	ATP6	Non-synonymous	M115T	0	0.31	0.118
Pre-terminal	U	T	8870	C	ATP6	Non-synonymous	M115T	0	0.31	0.118
Modern terminal	X	T	8870	C	ATP6	Non-synonymous	M115T	0	0.31	0.118
Modern terminal	N1	G	8872	A	ATP6	Non-synonymous	G116S	0.012	0.01	0.661
Modern terminal	N1	G	8873	A	ATP6	Non-synonymous	G116D	0.966	0	0.809
Modern terminal	R0	T	8875	C	ATP6	Non-synonymous	F117L	0.001	0.83	0.429
Modern terminal	U	T	8875	C	ATP6	Non-synonymous	F117L	0.001	0.83	0.429
Modern terminal	U	T	8875	C	ATP6	Non-synonymous	F117L	0.001	0.83	0.429
Pre-terminal	U	T	8875	C	ATP6	Non-synonymous	F117L	0.001	0.83	0.429

Modern terminal	JT	T	8875	C	ATP6	Non-synonymous	F117L	0.001	0.83	0.429
Modern terminal	N1	T	8875	C	ATP6	Non-synonymous	F117L	0.001	0.83	0.429
Ancient terminal	R0	T	8877	C	ATP6	Non-synonymous	F117L	0.001	0.83	0.429
Modern terminal	R0	T	8877	C	ATP6	Non-synonymous	F117L	0.001	0.83	0.429
Pre-terminal	R0	T	8877	C	ATP6	Non-synonymous	F117L	0.001	0.83	0.429
Pre-terminal	R0	T	8877	C	ATP6	Non-synonymous	F117L	0.001	0.83	0.429
Pre-terminal	U	T	8877	C	ATP6	Non-synonymous	F117L	0.001	0.83	0.429
Ancient terminal	JT	T	8877	C	ATP6	Non-synonymous	F117L	0.001	0.83	0.429
Modern terminal	JT	T	8877	C	ATP6	Non-synonymous	F117L	0.001	0.83	0.429
Modern terminal	JT	C	8878	T	ATP6	Non-synonymous	R118C	1	0	0.524
Ancient terminal	N2	C	8878	A	ATP6	Non-synonymous	R118S	1	0.02	0.619
Modern terminal	R0	G	8879	A	ATP6	Non-synonymous	R118H	0.999	0.02	0.401
Modern terminal	U	G	8879	A	ATP6	Non-synonymous	R118H	0.999	0.02	0.401
Modern terminal	N1	G	8879	A	ATP6	Non-synonymous	R118H	0.999	0.02	0.401
Modern terminal	U	T	8881	C	ATP6	Non-synonymous	S119P	0.325	0.07	0.3
Modern terminal	R0	C	8882	T	ATP6	Non-synonymous	S119F	0	0.31	0.206
Modern terminal	U	C	8882	G	ATP6	Non-synonymous	S119C	0.628	0.11	0.217
Modern terminal	N1	C	8882	T	ATP6	Non-synonymous	S119F	0	0.31	0.206
Pre-terminal	N1	C	8882	T	ATP6	Non-synonymous	S119F	0	0.31	0.206
Modern terminal	U	A	8887	G	ATP6	Non-synonymous	I121V	0	0.18	0.064
Modern terminal	U	A	8887	G	ATP6	Non-synonymous	I121V	0	0.18	0.064
Modern terminal	JT	A	8887	G	ATP6	Non-synonymous	I121V	0	0.18	0.064
Ancient terminal	R0	T	8888	A	ATP6	Non-synonymous	I121N	0.001	0.01	0.247
Modern terminal	R0	T	8888	C	ATP6	Non-synonymous	I121T	0	0.76	0.031
Pre-terminal	R0	T	8888	C	ATP6	Non-synonymous	I121T	0	0.76	0.031
Pre-terminal	R0	T	8888	C	ATP6	Non-synonymous	I121T	0	0.76	0.031
Modern terminal	U	T	8888	C	ATP6	Non-synonymous	I121T	0	0.76	0.031
Modern terminal	U	T	8888	C	ATP6	Non-synonymous	I121T	0	0.76	0.031
Modern terminal	N1	T	8888	C	ATP6	Non-synonymous	I121T	0	0.76	0.031
Modern terminal	U	A	8894	G	ATP6	Non-synonymous	N123S	0.002	0.83	0.068
Pre-terminal	R0	T	8895	A	ATP6	Non-synonymous	N123K	0.447	0.94	0.176
Modern terminal	R0	G	8896	A	ATP6	Non-synonymous	A124T	0	0.24	0.073
Modern terminal	U	G	8896	A	ATP6	Non-synonymous	A124T	0	0.24	0.073
Pre-terminal	JT	G	8896	A	ATP6	Non-synonymous	A124T	0	0.24	0.073
Ancient terminal	R0	G	8902	A	ATP6	Non-synonymous	A126T	0.003	0.02	0.359
Modern terminal	R0	G	8902	A	ATP6	Non-synonymous	A126T	0.003	0.02	0.359
Modern terminal	U	G	8902	A	ATP6	Non-synonymous	A126T	0.003	0.02	0.359
Pre-terminal	U	G	8902	A	ATP6	Non-synonymous	A126T	0.003	0.02	0.359
Ancient terminal	JT	G	8902	A	ATP6	Non-synonymous	A126T	0.003	0.02	0.359
Ancient terminal	N2	G	8902	T	ATP6	Non-synonymous	A126S	0.019	0.01	0.195
Modern terminal	U	C	8905	T	ATP6	Non-synonymous	H127Y	0.922	0	0.286
Modern terminal	JT	C	8905	T	ATP6	Non-synonymous	H127Y	0.922	0	0.286
Pre-terminal	X	C	8905	T	ATP6	Non-synonymous	H127Y	0.922	0	0.286
Pre-terminal	U	T	8908	C	ATP6	Non-synonymous	F128L	0	1	0.321
Modern terminal	X	T	8908	C	ATP6	Non-synonymous	F128L	0	1	0.321
Ancient terminal	R0	G	8921	A	ATP6	Non-synonymous	G132D	1	0	0.522
Modern terminal	U	G	8921	A	ATP6	Non-synonymous	G132D	1	0	0.522
Modern terminal	U	G	8921	A	ATP6	Non-synonymous	G132D	1	0	0.522
Pre-terminal	JT	G	8921	A	ATP6	Non-synonymous	G132D	1	0	0.522
Modern terminal	X	G	8921	A	ATP6	Non-synonymous	G132D	1	0	0.522

Modern terminal	X	C	8926	T	ATP6	Non-synonymous	P134S	0.999	0	0.337
Modern terminal	R0	C	8927	T	ATP6	Non-synonymous	P134L	1	0	0.398
Modern terminal	R0	A	8929	G	ATP6	Non-synonymous	T135A	0.978	0.38	0.145
Ancient terminal	JT	A	8929	C	ATP6	Non-synonymous	T135P	1	0.21	0.38
Pre-terminal	R0	C	8930	T	ATP6	Non-synonymous	T135M	0.999	0.2	0.129
Ancient terminal	U	C	8930	T	ATP6	Non-synonymous	T135M	0.999	0.2	0.129
Modern terminal	U	C	8930	T	ATP6	Non-synonymous	T135M	0.999	0.2	0.129
Ancient terminal	JT	C	8930	A	ATP6	Non-synonymous	T135K	0.991	0.05	0.267
Modern terminal	JT	C	8932	T	ATP6	Non-synonymous	P136S	0.999	0.22	0.307
Pre-terminal	U	C	8933	T	ATP6	Non-synonymous	P136L	1	0.44	0.285
Modern terminal	R0	C	8935	T	ATP6	Non-synonymous	L137F	0.996	0	0.49
Ancient terminal	JT	C	8935	T	ATP6	Non-synonymous	L137F	0.996	0	0.49
Modern terminal	JT	C	8935	T	ATP6	Non-synonymous	L137F	0.996	0	0.49
Pre-terminal	JT	C	8935	T	ATP6	Non-synonymous	L137F	0.996	0	0.49
Modern terminal	N2	C	8935	T	ATP6	Non-synonymous	L137F	0.996	0	0.49
Pre-terminal	R0	A	8938	G	ATP6	Non-synonymous	I138V	0.689	0.12	0.175
Ancient terminal	U	A	8938	G	ATP6	Non-synonymous	I138V	0.689	0.12	0.175
Pre-terminal	U	A	8938	G	ATP6	Non-synonymous	I138V	0.689	0.12	0.175
Pre-terminal	U	A	8938	G	ATP6	Non-synonymous	I138V	0.689	0.12	0.175
Modern terminal	JT	A	8938	G	ATP6	Non-synonymous	I138V	0.689	0.12	0.175
Modern terminal	R0	T	8939	C	ATP6	Non-synonymous	I138T	0.999	0	0.482
Modern terminal	R0	T	8939	C	ATP6	Non-synonymous	I138T	0.999	0	0.482
Modern terminal	R0	T	8939	C	ATP6	Non-synonymous	I138T	0.999	0	0.482
Modern terminal	R0	T	8939	C	ATP6	Non-synonymous	I138T	0.999	0	0.482
Modern terminal	R0	T	8939	C	ATP6	Non-synonymous	I138T	0.999	0	0.482
Modern terminal	R0	T	8939	C	ATP6	Non-synonymous	I138T	0.999	0	0.482
Pre-terminal	R0	T	8939	C	ATP6	Non-synonymous	I138T	0.999	0	0.482
Modern terminal	U	T	8939	C	ATP6	Non-synonymous	I138T	0.999	0	0.482
Pre-terminal	U	T	8939	C	ATP6	Non-synonymous	I138T	0.999	0	0.482
Ancient terminal	JT	T	8939	C	ATP6	Non-synonymous	I138T	0.999	0	0.482
Modern terminal	JT	T	8939	C	ATP6	Non-synonymous	I138T	0.999	0	0.482
Modern terminal	N2	T	8939	C	ATP6	Non-synonymous	I138T	0.999	0	0.482
Modern terminal	U	C	8941	T	ATP6	Non-synonymous	P139S	0.999	0	0.579
Modern terminal	U	C	8941	T	ATP6	Non-synonymous	P139S	0.999	0	0.579
Ancient terminal	R0	A	8944	C	ATP6	Non-synonymous	M140L	0.122	0.17	0.582
Ancient terminal	R0	T	8945	C	ATP6	Non-synonymous	M140T	0.976	0.12	0.601
Ancient terminal	R0	T	8945	C	ATP6	Non-synonymous	M140T	0.976	0.12	0.601
Modern terminal	R0	T	8945	C	ATP6	Non-synonymous	M140T	0.976	0.12	0.601
Modern terminal	R0	T	8945	C	ATP6	Non-synonymous	M140T	0.976	0.12	0.601
Pre-terminal	R0	T	8945	C	ATP6	Non-synonymous	M140T	0.976	0.12	0.601
Modern terminal	U	T	8945	C	ATP6	Non-synonymous	M140T	0.976	0.12	0.601
Modern terminal	U	T	8945	C	ATP6	Non-synonymous	M140T	0.976	0.12	0.601
Pre-terminal	R0	G	8950	A	ATP6	Non-synonymous	V142I	0	1	0.159
Pre-terminal	R0	G	8950	A	ATP6	Non-synonymous	V142I	0	1	0.159
Modern terminal	U	G	8950	A	ATP6	Non-synonymous	V142I	0	1	0.159
Modern terminal	U	G	8950	A	ATP6	Non-synonymous	V142I	0	1	0.159
Modern terminal	U	G	8950	A	ATP6	Non-synonymous	V142I	0	1	0.159
Pre-terminal	U	G	8950	A	ATP6	Non-synonymous	V142I	0	1	0.159
Modern terminal	JT	G	8950	A	ATP6	Non-synonymous	V142I	0	1	0.159
Modern terminal	N1	G	8950	A	ATP6	Non-synonymous	V142I	0	1	0.159

Pre-terminal	X	G	8950	A	ATP6	Non-synonymous	V142I	0	1	0.159
Modern terminal	N2	G	8950	A	ATP6	Non-synonymous	V142I	0	1	0.159
Modern terminal	R0	T	8951	C	ATP6	Non-synonymous	V142A	0.4	0	0.571
Modern terminal	R0	T	8951	C	ATP6	Non-synonymous	V142A	0.4	0	0.571
Pre-terminal	JT	T	8951	C	ATP6	Non-synonymous	V142A	0.4	0	0.571
Pre-terminal	JT	A	8953	G	ATP6	Non-synonymous	I143V	0.689	0.27	0.324
Modern terminal	R0	T	8954	C	ATP6	Non-synonymous	I143T	0.999	0	0.615
Pre-terminal	R0	T	8954	C	ATP6	Non-synonymous	I143T	0.999	0	0.615
Modern terminal	JT	T	8954	C	ATP6	Non-synonymous	I143T	0.999	0	0.615
Pre-terminal	N1	T	8954	C	ATP6	Non-synonymous	I143T	0.999	0	0.615
Modern terminal	R0	T	8957	C	ATP6	Non-synonymous	I144T	0.999	0	0.579
Modern terminal	R0	A	8961	T	ATP6	Non-synonymous	E145D	0.978	0.01	0.583
Pre-terminal	R0	A	8962	G	ATP6	Non-synonymous	T146A	0.978	0	0.617
Modern terminal	U	A	8962	G	ATP6	Non-synonymous	T146A	0.978	0	0.617
Pre-terminal	U	A	8962	T	ATP6	Non-synonymous	T146S	0.978	0.09	0.408
Modern terminal	JT	A	8962	G	ATP6	Non-synonymous	T146A	0.978	0	0.617
Pre-terminal	JT	A	8962	G	ATP6	Non-synonymous	T146A	0.978	0	0.617
Modern terminal	N1	A	8962	G	ATP6	Non-synonymous	T146A	0.978	0	0.617
Modern terminal	N1	A	8962	G	ATP6	Non-synonymous	T146A	0.978	0	0.617
Pre-terminal	R0	C	8963	T	ATP6	Non-synonymous	T146I	0.997	0	0.687
Modern terminal	U	A	8965	G	ATP6	Non-synonymous	I147V	0.001	0.07	0.348
Pre-terminal	R0	T	8966	C	ATP6	Non-synonymous	I147T	0.964	0.06	0.672
Modern terminal	U	T	8966	C	ATP6	Non-synonymous	I147T	0.964	0.06	0.672
Modern terminal	X	T	8966	C	ATP6	Non-synonymous	I147T	0.964	0.06	0.672
Modern terminal	X	T	8966	C	ATP6	Non-synonymous	I147T	0.964	0.06	0.672
Pre-terminal	X	T	8966	C	ATP6	Non-synonymous	I147T	0.964	0.06	0.672
Modern terminal	R0	A	8968	G	ATP6	Non-synonymous	S148G	0.998	0	0.561
Modern terminal	U	A	8968	G	ATP6	Non-synonymous	S148G	0.998	0	0.561
Modern terminal	R0	T	8972	G	ATP6	Non-synonymous	L149R	0.998	0	0.909
Modern terminal	U	C	8974	T	ATP6	Non-synonymous	L150F	0	1	0.071
Pre-terminal	U	C	8974	T	ATP6	Non-synonymous	L150F	0	1	0.071
Modern terminal	JT	C	8974	T	ATP6	Non-synonymous	L150F	0	1	0.071
Pre-terminal	JT	C	8974	T	ATP6	Non-synonymous	L150F	0	1	0.071
Pre-terminal	JT	C	8974	T	ATP6	Non-synonymous	L150F	0	1	0.071
Modern terminal	R0	T	8975	C	ATP6	Non-synonymous	L150P	0.791	0	0.716
Pre-terminal	R0	T	8975	C	ATP6	Non-synonymous	L150P	0.791	0	0.716
Modern terminal	U	T	8975	C	ATP6	Non-synonymous	L150P	0.791	0	0.716
Modern terminal	U	T	8975	C	ATP6	Non-synonymous	L150P	0.791	0	0.716
Modern terminal	JT	T	8975	C	ATP6	Non-synonymous	L150P	0.791	0	0.716
Pre-terminal	JT	T	8975	C	ATP6	Non-synonymous	L150P	0.791	0	0.716
Modern terminal	X	T	8975	C	ATP6	Non-synonymous	L150P	0.791	0	0.716
Modern terminal	R0	T	8978	C	ATP6	Non-synonymous	I151T	0.999	0	0.485
Pre-terminal	JT	T	8978	C	ATP6	Non-synonymous	I151T	0.999	0	0.485
Modern terminal	R0	A	8981	G	ATP6	Non-synonymous	Q152R	0.944	1	0.681
Modern terminal	R0	A	8981	G	ATP6	Non-synonymous	Q152R	0.944	1	0.681
Modern terminal	U	A	8981	G	ATP6	Non-synonymous	Q152R	0.944	1	0.681
Modern terminal	R0	A	8986	G	ATP6	Non-synonymous	M154V	0.013	0.39	0.234
Modern terminal	R0	A	8986	G	ATP6	Non-synonymous	M154V	0.013	0.39	0.234
Pre-terminal	R0	A	8986	G	ATP6	Non-synonymous	M154V	0.013	0.39	0.234
Modern terminal	U	A	8986	G	ATP6	Non-synonymous	M154V	0.013	0.39	0.234

Pre-terminal	U	A	8986	G	ATP6	Non-synonymous	M154V	0.013	0.39	0.234
Modern terminal	R0	T	8987	C	ATP6	Non-synonymous	M154T	0.435	0.02	0.566
Modern terminal	N1	T	8987	C	ATP6	Non-synonymous	M154T	0.435	0.02	0.566
Modern terminal	R0	G	8989	A	ATP6	Non-synonymous	A155T	0.998	0.18	0.534
Modern terminal	R0	G	8989	A	ATP6	Non-synonymous	A155T	0.998	0.18	0.534
Modern terminal	R0	G	8989	A	ATP6	Non-synonymous	A155T	0.998	0.18	0.534
Modern terminal	R0	G	8989	A	ATP6	Non-synonymous	A155T	0.998	0.18	0.534
Modern terminal	R0	G	8989	A	ATP6	Non-synonymous	A155T	0.998	0.18	0.534
Ancient terminal	JT	G	8989	A	ATP6	Non-synonymous	A155T	0.998	0.18	0.534
Modern terminal	JT	G	8989	A	ATP6	Non-synonymous	A155T	0.998	0.18	0.534
Modern terminal	JT	G	8989	A	ATP6	Non-synonymous	A155T	0.998	0.18	0.534
Pre-terminal	JT	G	8989	A	ATP6	Non-synonymous	A155T	0.998	0.18	0.534
Modern terminal	N2	G	8989	A	ATP6	Non-synonymous	A155T	0.998	0.18	0.534
Modern terminal	R0	T	8993	G	ATP6	Non-synonymous	L156R	0.999	0	0.9
Modern terminal	R0	T	8993	G	ATP6	Non-synonymous	L156R	0.999	0	0.9
Modern terminal	U	T	8993	G	ATP6	Non-synonymous	L156R	0.999	0	0.9
Modern terminal	R0	G	8995	C	ATP6	Non-synonymous	A157P	1	0.01	0.887
Modern terminal	R0	G	8998	A	ATP6	Non-synonymous	V158M	0.724	0	0.537
Modern terminal	R0	G	8998	A	ATP6	Non-synonymous	V158M	0.724	0	0.537
Pre-terminal	R0	G	8998	A	ATP6	Non-synonymous	V158M	0.724	0	0.537
Modern terminal	U	G	8998	A	ATP6	Non-synonymous	V158M	0.724	0	0.537
Modern terminal	U	G	8998	A	ATP6	Non-synonymous	V158M	0.724	0	0.537
Modern terminal	JT	G	8998	A	ATP6	Non-synonymous	V158M	0.724	0	0.537
Modern terminal	JT	T	8999	C	ATP6	Non-synonymous	V158A	0.022	0	0.692
Modern terminal	JT	T	8999	C	ATP6	Non-synonymous	V158A	0.022	0	0.692
Modern terminal	X	C	9001	T	ATP6	Non-synonymous	R159C	1	0	0.726
Modern terminal	JT	T	9005	C	ATP6	Non-synonymous	L160P	0.997	0	0.928
Pre-terminal	JT	T	9005	C	ATP6	Non-synonymous	L160P	0.997	0	0.928
Modern terminal	R0	A	9007	G	ATP6	Non-synonymous	T161A	0.995	0	0.644
Modern terminal	R0	A	9007	G	ATP6	Non-synonymous	T161A	0.995	0	0.644
Modern terminal	R0	A	9007	G	ATP6	Non-synonymous	T161A	0.995	0	0.644
Modern terminal	R0	A	9007	G	ATP6	Non-synonymous	T161A	0.995	0	0.644
Modern terminal	R0	A	9007	G	ATP6	Non-synonymous	T161A	0.995	0	0.644
Modern terminal	R0	A	9007	G	ATP6	Non-synonymous	T161A	0.995	0	0.644
Modern terminal	R0	A	9007	G	ATP6	Non-synonymous	T161A	0.995	0	0.644
Modern terminal	R0	A	9007	G	ATP6	Non-synonymous	T161A	0.995	0	0.644
Modern terminal	R0	A	9007	G	ATP6	Non-synonymous	T161A	0.995	0	0.644
Modern terminal	R0	A	9007	G	ATP6	Non-synonymous	T161A	0.995	0	0.644
Modern terminal	R0	A	9007	G	ATP6	Non-synonymous	T161A	0.995	0	0.644
Pre-terminal	R0	A	9007	G	ATP6	Non-synonymous	T161A	0.995	0	0.644
Pre-terminal	R0	A	9007	G	ATP6	Non-synonymous	T161A	0.995	0	0.644
Pre-terminal	R0	A	9007	G	ATP6	Non-synonymous	T161A	0.995	0	0.644
Pre-terminal	R0	A	9007	G	ATP6	Non-synonymous	T161A	0.995	0	0.644
Pre-terminal	R0	A	9007	G	ATP6	Non-synonymous	T161A	0.995	0	0.644
Pre-terminal	R0	A	9007	G	ATP6	Non-synonymous	T161A	0.995	0	0.644
Pre-terminal	R0	A	9007	G	ATP6	Non-synonymous	T161A	0.995	0	0.644
Ancient terminal	U	A	9007	G	ATP6	Non-synonymous	T161A	0.995	0	0.644
Ancient terminal	U	A	9007	G	ATP6	Non-synonymous	T161A	0.995	0	0.644
Modern terminal	U	A	9007	G	ATP6	Non-synonymous	T161A	0.995	0	0.644
Modern terminal	U	A	9007	G	ATP6	Non-synonymous	T161A	0.995	0	0.644

Modern terminal	U	A	9007	G	ATP6	Non-synonymous	T161A	0.995	0	0.644
Modern terminal	U	A	9007	G	ATP6	Non-synonymous	T161A	0.995	0	0.644
Modern terminal	U	A	9007	G	ATP6	Non-synonymous	T161A	0.995	0	0.644
Modern terminal	U	A	9007	G	ATP6	Non-synonymous	T161A	0.995	0	0.644
Modern terminal	U	A	9007	G	ATP6	Non-synonymous	T161A	0.995	0	0.644
Pre-terminal	U	A	9007	G	ATP6	Non-synonymous	T161A	0.995	0	0.644
Pre-terminal	U	A	9007	G	ATP6	Non-synonymous	T161A	0.995	0	0.644
Ancient terminal	JT	A	9007	G	ATP6	Non-synonymous	T161A	0.995	0	0.644
Ancient terminal	JT	A	9007	G	ATP6	Non-synonymous	T161A	0.995	0	0.644
Modern terminal	JT	A	9007	G	ATP6	Non-synonymous	T161A	0.995	0	0.644
Modern terminal	JT	A	9007	G	ATP6	Non-synonymous	T161A	0.995	0	0.644
Modern terminal	JT	A	9007	G	ATP6	Non-synonymous	T161A	0.995	0	0.644
Modern terminal	JT	A	9007	G	ATP6	Non-synonymous	T161A	0.995	0	0.644
Modern terminal	JT	A	9007	G	ATP6	Non-synonymous	T161A	0.995	0	0.644
Modern terminal	JT	A	9007	G	ATP6	Non-synonymous	T161A	0.995	0	0.644
Modern terminal	JT	A	9007	G	ATP6	Non-synonymous	T161A	0.995	0	0.644
Modern terminal	JT	A	9007	G	ATP6	Non-synonymous	T161A	0.995	0	0.644
Modern terminal	JT	A	9007	G	ATP6	Non-synonymous	T161A	0.995	0	0.644
Modern terminal	JT	A	9007	G	ATP6	Non-synonymous	T161A	0.995	0	0.644
Pre-terminal	N1	A	9007	G	ATP6	Non-synonymous	T161A	0.995	0	0.644
Modern terminal	X	A	9007	G	ATP6	Non-synonymous	T161A	0.995	0	0.644
Pre-terminal	R0	C	9008	G	ATP6	Non-synonymous	T161S	0.995	0.05	0.399
Modern terminal	R0	G	9010	A	ATP6	Non-synonymous	A162T	0.998	0	0.568
Modern terminal	R0	G	9010	A	ATP6	Non-synonymous	A162T	0.998	0	0.568
Modern terminal	R0	G	9010	A	ATP6	Non-synonymous	A162T	0.998	0	0.568
Modern terminal	N1	G	9010	A	ATP6	Non-synonymous	A162T	0.998	0	0.568
Modern terminal	N1	G	9010	A	ATP6	Non-synonymous	A162T	0.998	0	0.568
Modern terminal	N2	G	9010	A	ATP6	Non-synonymous	A162T	0.998	0	0.568
Modern terminal	U	C	9011	T	ATP6	Non-synonymous	A162V	0.999	0	0.612
Modern terminal	R0	A	9016	G	ATP6	Non-synonymous	I164V	0.916	0.02	0.358
Pre-terminal	JT	A	9016	G	ATP6	Non-synonymous	I164V	0.916	0.02	0.358
Modern terminal	R0	T	9017	C	ATP6	Non-synonymous	I164T	1	0	0.643
Modern terminal	R0	T	9017	C	ATP6	Non-synonymous	I164T	1	0	0.643
Pre-terminal	R0	T	9017	C	ATP6	Non-synonymous	I164T	1	0	0.643
Modern terminal	JT	T	9017	C	ATP6	Non-synonymous	I164T	1	0	0.643
Pre-terminal	JT	T	9017	C	ATP6	Non-synonymous	I164T	1	0	0.643
Modern terminal	X	T	9017	C	ATP6	Non-synonymous	I164T	1	0	0.643
Modern terminal	R0	C	9020	T	ATP6	Non-synonymous	T165I	0.999	0.11	0.629
Modern terminal	R0	G	9022	A	ATP6	Non-synonymous	A166T	0.999	0	0.553
Modern terminal	R0	G	9022	A	ATP6	Non-synonymous	A166T	0.999	0	0.553
Modern terminal	R0	G	9022	A	ATP6	Non-synonymous	A166T	0.999	0	0.553
Pre-terminal	R0	G	9022	A	ATP6	Non-synonymous	A166T	0.999	0	0.553
Modern terminal	U	G	9022	A	ATP6	Non-synonymous	A166T	0.999	0	0.553
Pre-terminal	JT	G	9022	A	ATP6	Non-synonymous	A166T	0.999	0	0.553
Ancient terminal	R0	C	9023	A	ATP6	Non-synonymous	A166E	0.999	0	0.758
Modern terminal	R0	G	9025	A	ATP6	Non-synonymous	G167S	1	0	0.71
Modern terminal	R0	G	9025	A	ATP6	Non-synonymous	G167S	1	0	0.71
Modern terminal	R0	G	9025	A	ATP6	Non-synonymous	G167S	1	0	0.71
Modern terminal	R0	G	9025	A	ATP6	Non-synonymous	G167S	1	0	0.71
Modern terminal	R0	G	9025	A	ATP6	Non-synonymous	G167S	1	0	0.71

Pre-terminal	R0	G	9025	A	ATP6	Non-synonymous	G167S	1	0	0.71
Pre-terminal	R0	G	9025	A	ATP6	Non-synonymous	G167S	1	0	0.71
Pre-terminal	R0	G	9025	A	ATP6	Non-synonymous	G167S	1	0	0.71
Pre-terminal	R0	G	9025	A	ATP6	Non-synonymous	G167S	1	0	0.71
Pre-terminal	R0	G	9025	A	ATP6	Non-synonymous	G167S	1	0	0.71
Pre-terminal	R0	G	9025	A	ATP6	Non-synonymous	G167S	1	0	0.71
Pre-terminal	R0	G	9025	A	ATP6	Non-synonymous	G167S	1	0	0.71
Modern terminal	U	G	9025	A	ATP6	Non-synonymous	G167S	1	0	0.71
Modern terminal	U	G	9025	A	ATP6	Non-synonymous	G167S	1	0	0.71
Modern terminal	JT	G	9025	A	ATP6	Non-synonymous	G167S	1	0	0.71
Modern terminal	JT	G	9025	A	ATP6	Non-synonymous	G167S	1	0	0.71
Modern terminal	JT	G	9025	A	ATP6	Non-synonymous	G167S	1	0	0.71
Modern terminal	JT	G	9025	A	ATP6	Non-synonymous	G167S	1	0	0.71
Modern terminal	JT	G	9025	A	ATP6	Non-synonymous	G167S	1	0	0.71
Modern terminal	JT	G	9025	A	ATP6	Non-synonymous	G167S	1	0	0.71
Modern terminal	JT	G	9025	A	ATP6	Non-synonymous	G167S	1	0	0.71
Modern terminal	X	G	9025	A	ATP6	Non-synonymous	G167S	1	0	0.71
Modern terminal	R0	G	9026	A	ATP6	Non-synonymous	G167D	1	0	0.874
Modern terminal	R0	G	9026	C	ATP6	Non-synonymous	G167A	1	0	0.786
Modern terminal	R0	G	9026	C	ATP6	Non-synonymous	G167A	1	0	0.786
Modern terminal	U	G	9026	A	ATP6	Non-synonymous	G167D	1	0	0.874
Modern terminal	U	G	9026	A	ATP6	Non-synonymous	G167D	1	0	0.874
Modern terminal	R0	A	9029	G	ATP6	Non-synonymous	H168R	0.984	0	0.662
Modern terminal	N2	C	9031	A	ATP6	Non-synonymous	L169M	1	0	0.483
Modern terminal	R0	C	9034	G	ATP6	Non-synonymous	L170V	0.967	0	0.656
Modern terminal	R0	A	9037	G	ATP6	Non-synonymous	M171V	0.778	0.03	0.455
Modern terminal	R0	A	9037	G	ATP6	Non-synonymous	M171V	0.778	0.03	0.455
Pre-terminal	U	A	9037	G	ATP6	Non-synonymous	M171V	0.778	0.03	0.455
Modern terminal	R0	T	9038	C	ATP6	Non-synonymous	M171T	0.949	0.01	0.498
Modern terminal	R0	T	9038	C	ATP6	Non-synonymous	M171T	0.949	0.01	0.498
Modern terminal	U	T	9038	C	ATP6	Non-synonymous	M171T	0.949	0.01	0.498
Modern terminal	U	T	9038	C	ATP6	Non-synonymous	M171T	0.949	0.01	0.498
Modern terminal	U	T	9038	C	ATP6	Non-synonymous	M171T	0.949	0.01	0.498
Modern terminal	U	T	9038	C	ATP6	Non-synonymous	M171T	0.949	0.01	0.498
Modern terminal	U	T	9038	C	ATP6	Non-synonymous	M171T	0.949	0.01	0.498
Ancient terminal	R0	A	9041	G	ATP6	Non-synonymous	H172R	0.664	0.1	0.662
Modern terminal	R0	A	9041	G	ATP6	Non-synonymous	H172R	0.664	0.1	0.662
Modern terminal	R0	A	9041	G	ATP6	Non-synonymous	H172R	0.664	0.1	0.662
Ancient terminal	JT	A	9041	G	ATP6	Non-synonymous	H172R	0.664	0.1	0.662
Pre-terminal	JT	A	9041	G	ATP6	Non-synonymous	H172R	0.664	0.1	0.662
Pre-terminal	R0	A	9046	G	ATP6	Non-synonymous	I174V	0.788	0.18	0.252
Modern terminal	X	A	9046	G	ATP6	Non-synonymous	I174V	0.788	0.18	0.252
Modern terminal	R0	T	9047	C	ATP6	Non-synonymous	I174T	0.999	0.08	0.464
Modern terminal	R0	T	9047	C	ATP6	Non-synonymous	I174T	0.999	0.08	0.464
Modern terminal	JT	T	9047	C	ATP6	Non-synonymous	I174T	0.999	0.08	0.464
Ancient terminal	R0	T	9048	A	ATP6	Non-synonymous	I174M	1	0	0.444
Modern terminal	R0	A	9052	G	ATP6	Non-synonymous	S176G	0.116	0.53	0.04
Pre-terminal	R0	A	9052	G	ATP6	Non-synonymous	S176G	0.116	0.53	0.04
Pre-terminal	R0	A	9052	G	ATP6	Non-synonymous	S176G	0.116	0.53	0.04
Modern terminal	U	A	9052	G	ATP6	Non-synonymous	S176G	0.116	0.53	0.04

Pre-terminal	JT	A	9052	G	ATP6	Non-synonymous	S176G	0.116	0.53	0.04
Modern terminal	R0	G	9053	A	ATP6	Non-synonymous	S176N	0.388	0.56	0.166
Modern terminal	R0	G	9053	A	ATP6	Non-synonymous	S176N	0.388	0.56	0.166
Modern terminal	R0	G	9053	A	ATP6	Non-synonymous	S176N	0.388	0.56	0.166
Modern terminal	R0	G	9053	A	ATP6	Non-synonymous	S176N	0.388	0.56	0.166
Modern terminal	R0	G	9053	A	ATP6	Non-synonymous	S176N	0.388	0.56	0.166
Modern terminal	R0	G	9053	A	ATP6	Non-synonymous	S176N	0.388	0.56	0.166
Modern terminal	R0	G	9053	A	ATP6	Non-synonymous	S176N	0.388	0.56	0.166
Pre-terminal	R0	G	9053	A	ATP6	Non-synonymous	S176N	0.388	0.56	0.166
Pre-terminal	R0	G	9053	A	ATP6	Non-synonymous	S176N	0.388	0.56	0.166
Pre-terminal	R0	G	9053	A	ATP6	Non-synonymous	S176N	0.388	0.56	0.166
Pre-terminal	R0	G	9053	A	ATP6	Non-synonymous	S176N	0.388	0.56	0.166
Pre-terminal	R0	G	9053	A	ATP6	Non-synonymous	S176N	0.388	0.56	0.166
Ancient terminal	U	G	9053	A	ATP6	Non-synonymous	S176N	0.388	0.56	0.166
Ancient terminal	U	G	9053	A	ATP6	Non-synonymous	S176N	0.388	0.56	0.166
Modern terminal	U	G	9053	A	ATP6	Non-synonymous	S176N	0.388	0.56	0.166
Modern terminal	U	G	9053	A	ATP6	Non-synonymous	S176N	0.388	0.56	0.166
Modern terminal	U	G	9053	A	ATP6	Non-synonymous	S176N	0.388	0.56	0.166
Modern terminal	U	G	9053	A	ATP6	Non-synonymous	S176N	0.388	0.56	0.166
Modern terminal	U	G	9053	A	ATP6	Non-synonymous	S176N	0.388	0.56	0.166
Pre-terminal	U	G	9053	A	ATP6	Non-synonymous	S176N	0.388	0.56	0.166
Modern terminal	JT	G	9053	A	ATP6	Non-synonymous	S176N	0.388	0.56	0.166
Modern terminal	JT	G	9053	A	ATP6	Non-synonymous	S176N	0.388	0.56	0.166
Modern terminal	JT	G	9053	A	ATP6	Non-synonymous	S176N	0.388	0.56	0.166
Modern terminal	JT	G	9053	A	ATP6	Non-synonymous	S176N	0.388	0.56	0.166
Modern terminal	JT	G	9053	A	ATP6	Non-synonymous	S176N	0.388	0.56	0.166
Pre-terminal	JT	G	9053	A	ATP6	Non-synonymous	S176N	0.388	0.56	0.166
Pre-terminal	JT	G	9053	A	ATP6	Non-synonymous	S176N	0.388	0.56	0.166
Modern terminal	N1	G	9053	A	ATP6	Non-synonymous	S176N	0.388	0.56	0.166
Pre-terminal	N1	G	9053	A	ATP6	Non-synonymous	S176N	0.388	0.56	0.166
Pre-terminal	N1	G	9053	A	ATP6	Non-synonymous	S176N	0.388	0.56	0.166
Modern terminal	X	G	9053	A	ATP6	Non-synonymous	S176N	0.388	0.56	0.166
Ancient terminal	R0	G	9055	A	ATP6	Non-synonymous	A177T	0.42	0.16	0.297
Ancient terminal	R0	G	9055	A	ATP6	Non-synonymous	A177T	0.42	0.16	0.297
Modern terminal	R0	G	9055	A	ATP6	Non-synonymous	A177T	0.42	0.16	0.297
Modern terminal	R0	G	9055	A	ATP6	Non-synonymous	A177T	0.42	0.16	0.297
Modern terminal	R0	G	9055	A	ATP6	Non-synonymous	A177T	0.42	0.16	0.297
Modern terminal	R0	G	9055	A	ATP6	Non-synonymous	A177T	0.42	0.16	0.297
Modern terminal	R0	G	9055	A	ATP6	Non-synonymous	A177T	0.42	0.16	0.297
Modern terminal	R0	G	9055	A	ATP6	Non-synonymous	A177T	0.42	0.16	0.297
Modern terminal	R0	G	9055	A	ATP6	Non-synonymous	A177T	0.42	0.16	0.297
Modern terminal	R0	G	9055	A	ATP6	Non-synonymous	A177T	0.42	0.16	0.297
Modern terminal	R0	G	9055	A	ATP6	Non-synonymous	A177T	0.42	0.16	0.297
Modern terminal	R0	G	9055	A	ATP6	Non-synonymous	A177T	0.42	0.16	0.297
Modern terminal	R0	G	9055	A	ATP6	Non-synonymous	A177T	0.42	0.16	0.297
Modern terminal	R0	G	9055	A	ATP6	Non-synonymous	A177T	0.42	0.16	0.297
Pre-terminal	R0	G	9055	A	ATP6	Non-synonymous	A177T	0.42	0.16	0.297
Pre-terminal	R0	G	9055	A	ATP6	Non-synonymous	A177T	0.42	0.16	0.297
Pre-terminal	R0	G	9055	A	ATP6	Non-synonymous	A177T	0.42	0.16	0.297
Pre-terminal	R0	G	9055	A	ATP6	Non-synonymous	A177T	0.42	0.16	0.297

Pre-terminal	R0	G	9055	A	ATP6	Non-synonymous	A177T	0.42	0.16	0.297
Pre-terminal	R0	G	9055	A	ATP6	Non-synonymous	A177T	0.42	0.16	0.297
Pre-terminal	R0	G	9055	A	ATP6	Non-synonymous	A177T	0.42	0.16	0.297
Pre-terminal	R0	G	9055	A	ATP6	Non-synonymous	A177T	0.42	0.16	0.297
Pre-terminal	R0	G	9055	A	ATP6	Non-synonymous	A177T	0.42	0.16	0.297
Modern terminal	U	G	9055	A	ATP6	Non-synonymous	A177T	0.42	0.16	0.297
Modern terminal	U	G	9055	A	ATP6	Non-synonymous	A177T	0.42	0.16	0.297
Modern terminal	U	G	9055	A	ATP6	Non-synonymous	A177T	0.42	0.16	0.297
Modern terminal	U	G	9055	A	ATP6	Non-synonymous	A177T	0.42	0.16	0.297
Modern terminal	U	G	9055	A	ATP6	Non-synonymous	A177T	0.42	0.16	0.297
Modern terminal	U	G	9055	A	ATP6	Non-synonymous	A177T	0.42	0.16	0.297
Modern terminal	U	G	9055	A	ATP6	Non-synonymous	A177T	0.42	0.16	0.297
Modern terminal	U	G	9055	T	ATP6	Non-synonymous	A177S	0.002	0.08	0.253
Pre-terminal	U	G	9055	A	ATP6	Non-synonymous	A177T	0.42	0.16	0.297
Pre-terminal	U	G	9055	A	ATP6	Non-synonymous	A177T	0.42	0.16	0.297
Modern terminal	JT	G	9055	A	ATP6	Non-synonymous	A177T	0.42	0.16	0.297
Modern terminal	JT	G	9055	A	ATP6	Non-synonymous	A177T	0.42	0.16	0.297
Modern terminal	JT	G	9055	A	ATP6	Non-synonymous	A177T	0.42	0.16	0.297
Modern terminal	JT	G	9055	A	ATP6	Non-synonymous	A177T	0.42	0.16	0.297
Modern terminal	JT	G	9055	A	ATP6	Non-synonymous	A177T	0.42	0.16	0.297
Modern terminal	JT	G	9055	A	ATP6	Non-synonymous	A177T	0.42	0.16	0.297
Modern terminal	JT	G	9055	A	ATP6	Non-synonymous	A177T	0.42	0.16	0.297
Modern terminal	JT	G	9055	A	ATP6	Non-synonymous	A177T	0.42	0.16	0.297
Pre-terminal	JT	G	9055	A	ATP6	Non-synonymous	A177T	0.42	0.16	0.297
Pre-terminal	JT	G	9055	A	ATP6	Non-synonymous	A177T	0.42	0.16	0.297
Pre-terminal	JT	G	9055	A	ATP6	Non-synonymous	A177T	0.42	0.16	0.297
Pre-terminal	JT	G	9055	A	ATP6	Non-synonymous	A177T	0.42	0.16	0.297
Modern terminal	N1	G	9055	A	ATP6	Non-synonymous	A177T	0.42	0.16	0.297
Modern terminal	N1	G	9055	A	ATP6	Non-synonymous	A177T	0.42	0.16	0.297
Modern terminal	N2	G	9055	A	ATP6	Non-synonymous	A177T	0.42	0.16	0.297
Modern terminal	R0	C	9056	T	ATP6	Non-synonymous	A177V	0.755	0	0.37
Modern terminal	R0	C	9056	T	ATP6	Non-synonymous	A177V	0.755	0	0.37
Pre-terminal	R0	C	9056	T	ATP6	Non-synonymous	A177V	0.755	0	0.37
Modern terminal	U	C	9056	T	ATP6	Non-synonymous	A177V	0.755	0	0.37
Pre-terminal	R0	A	9058	G	ATP6	Non-synonymous	T178A	0.002	0.41	0.269
Pre-terminal	R0	A	9058	G	ATP6	Non-synonymous	T178A	0.002	0.41	0.269
Ancient terminal	U	A	9058	G	ATP6	Non-synonymous	T178A	0.002	0.41	0.269
Modern terminal	U	A	9058	C	ATP6	Non-synonymous	T178P	0.994	0.02	0.416
Modern terminal	U	A	9058	G	ATP6	Non-synonymous	T178A	0.002	0.41	0.269
Modern terminal	U	A	9058	G	ATP6	Non-synonymous	T178A	0.002	0.41	0.269
Modern terminal	U	A	9058	G	ATP6	Non-synonymous	T178A	0.002	0.41	0.269
Modern terminal	U	A	9058	G	ATP6	Non-synonymous	T178A	0.002	0.41	0.269
Modern terminal	U	A	9058	G	ATP6	Non-synonymous	T178A	0.002	0.41	0.269
Modern terminal	U	A	9058	G	ATP6	Non-synonymous	T178A	0.002	0.41	0.269
Pre-terminal	U	A	9058	G	ATP6	Non-synonymous	T178A	0.002	0.41	0.269
Modern terminal	N1	A	9058	G	ATP6	Non-synonymous	T178A	0.002	0.41	0.269
Modern terminal	R0	G	9064	A	ATP6	Non-synonymous	A180T	0.001	0.5	0.281
Modern terminal	R0	G	9064	A	ATP6	Non-synonymous	A180T	0.001	0.5	0.281
Modern terminal	R0	G	9064	A	ATP6	Non-synonymous	A180T	0.001	0.5	0.281
Modern terminal	R0	G	9064	A	ATP6	Non-synonymous	A180T	0.001	0.5	0.281

Modern terminal	R0	G	9064	A	ATP6	Non-synonymous	A180T	0.001	0.5	0.281
Modern terminal	R0	G	9064	A	ATP6	Non-synonymous	A180T	0.001	0.5	0.281
Modern terminal	R0	G	9064	A	ATP6	Non-synonymous	A180T	0.001	0.5	0.281
Pre-terminal	R0	G	9064	A	ATP6	Non-synonymous	A180T	0.001	0.5	0.281
Modern terminal	U	G	9064	A	ATP6	Non-synonymous	A180T	0.001	0.5	0.281
Modern terminal	U	G	9064	A	ATP6	Non-synonymous	A180T	0.001	0.5	0.281
Modern terminal	U	G	9064	A	ATP6	Non-synonymous	A180T	0.001	0.5	0.281
Pre-terminal	U	G	9064	A	ATP6	Non-synonymous	A180T	0.001	0.5	0.281
Pre-terminal	JT	G	9064	A	ATP6	Non-synonymous	A180T	0.001	0.5	0.281
Pre-terminal	N1	G	9064	A	ATP6	Non-synonymous	A180T	0.001	0.5	0.281
Modern terminal	R0	C	9065	T	ATP6	Non-synonymous	A180V	0.327	0.56	0.225
Pre-terminal	R0	C	9065	T	ATP6	Non-synonymous	A180V	0.327	0.56	0.225
Ancient terminal	U	A	9067	G	ATP6	Non-synonymous	M181V	0.003	0.01	0.185
Pre-terminal	JT	A	9067	G	ATP6	Non-synonymous	M181V	0.003	0.01	0.185
Modern terminal	R0	T	9068	C	ATP6	Non-synonymous	M181T	0.031	0	0.213
Modern terminal	R0	T	9070	C	ATP6	Non-synonymous	S182P	0.972	0.29	0.45
Pre-terminal	U	T	9070	G	ATP6	Non-synonymous	S182A	0.225	0.57	0.16
Modern terminal	JT	T	9070	C	ATP6	Non-synonymous	S182P	0.972	0.29	0.45
Modern terminal	R0	C	9071	T	ATP6	Non-synonymous	S182L	0.002	1	0.254
Modern terminal	R0	C	9071	T	ATP6	Non-synonymous	S182L	0.002	1	0.254
Pre-terminal	R0	C	9071	T	ATP6	Non-synonymous	S182L	0.002	1	0.254
Modern terminal	R0	A	9073	C	ATP6	Non-synonymous	T183P	0.978	0.47	0.372
Pre-terminal	R0	A	9073	G	ATP6	Non-synonymous	T183A	0.008	0.23	0.129
Modern terminal	U	A	9073	G	ATP6	Non-synonymous	T183A	0.008	0.23	0.129
Pre-terminal	U	A	9073	G	ATP6	Non-synonymous	T183A	0.008	0.23	0.129
Modern terminal	X	A	9073	G	ATP6	Non-synonymous	T183A	0.008	0.23	0.129
Modern terminal	R0	C	9074	T	ATP6	Non-synonymous	T183I	0.86	0.05	0.189
Modern terminal	R0	T	9077	A	ATP6	Non-synonymous	I184N	0.95	0.11	0.564
Pre-terminal	R0	T	9077	C	ATP6	Non-synonymous	I184T	0.002	0.12	0.358
Modern terminal	U	T	9077	C	ATP6	Non-synonymous	I184T	0.002	0.12	0.358
Modern terminal	JT	T	9077	C	ATP6	Non-synonymous	I184T	0.002	0.12	0.358
Modern terminal	JT	T	9077	C	ATP6	Non-synonymous	I184T	0.002	0.12	0.358
Pre-terminal	JT	T	9077	C	ATP6	Non-synonymous	I184T	0.002	0.12	0.358
Modern terminal	R0	A	9080	G	ATP6	Non-synonymous	N185S	0.002	0.53	0.041
Pre-terminal	R0	A	9080	G	ATP6	Non-synonymous	N185S	0.002	0.53	0.041
Modern terminal	R0	T	9083	C	ATP6	Non-synonymous	L186P	0.005	0.13	0.177
Modern terminal	R0	T	9083	C	ATP6	Non-synonymous	L186P	0.005	0.13	0.177
Pre-terminal	R0	T	9083	C	ATP6	Non-synonymous	L186P	0.005	0.13	0.177
Pre-terminal	R0	T	9083	C	ATP6	Non-synonymous	L186P	0.005	0.13	0.177
Pre-terminal	R0	T	9083	C	ATP6	Non-synonymous	L186P	0.005	0.13	0.177
Modern terminal	U	T	9083	C	ATP6	Non-synonymous	L186P	0.005	0.13	0.177
Modern terminal	U	T	9083	C	ATP6	Non-synonymous	L186P	0.005	0.13	0.177
Modern terminal	U	T	9083	C	ATP6	Non-synonymous	L186P	0.005	0.13	0.177
Modern terminal	U	T	9083	C	ATP6	Non-synonymous	L186P	0.005	0.13	0.177
Modern terminal	U	T	9083	C	ATP6	Non-synonymous	L186P	0.005	0.13	0.177
Pre-terminal	JT	T	9083	C	ATP6	Non-synonymous	L186P	0.005	0.13	0.177
Modern terminal	X	T	9083	C	ATP6	Non-synonymous	L186P	0.005	0.13	0.177
Modern terminal	R0	C	9085	T	ATP6	Non-synonymous	P187S	0.011	0.16	0.182
Modern terminal	X	C	9085	T	ATP6	Non-synonymous	P187S	0.011	0.16	0.182
Pre-terminal	R0	C	9086	T	ATP6	Non-synonymous	P187L	0.775	0.49	0.159

Modern terminal	U	C	9086	T	ATP6	Non-synonymous	P187L	0.775	0.49	0.159
Ancient terminal	R0	T	9088	C	ATP6	Non-synonymous	S188P	0.251	0.09	0.35
Modern terminal	R0	T	9088	C	ATP6	Non-synonymous	S188P	0.251	0.09	0.35
Pre-terminal	R0	T	9088	C	ATP6	Non-synonymous	S188P	0.251	0.09	0.35
Pre-terminal	U	T	9088	C	ATP6	Non-synonymous	S188P	0.251	0.09	0.35
Modern terminal	JT	T	9088	C	ATP6	Non-synonymous	S188P	0.251	0.09	0.35
Modern terminal	JT	T	9088	C	ATP6	Non-synonymous	S188P	0.251	0.09	0.35
Modern terminal	N2	T	9088	C	ATP6	Non-synonymous	S188P	0.251	0.09	0.35
Modern terminal	N2	T	9088	C	ATP6	Non-synonymous	S188P	0.251	0.09	0.35
Modern terminal	N2	T	9088	C	ATP6	Non-synonymous	S188P	0.251	0.09	0.35
Pre-terminal	N2	T	9088	C	ATP6	Non-synonymous	S188P	0.251	0.09	0.35
Modern terminal	R0	A	9091	G	ATP6	Non-synonymous	T189A	0	1	0.043
Pre-terminal	R0	A	9091	G	ATP6	Non-synonymous	T189A	0	1	0.043
Modern terminal	R0	T	9095	C	ATP6	Non-synonymous	L190P	0.052	0.1	0.601
Modern terminal	U	T	9095	C	ATP6	Non-synonymous	L190P	0.052	0.1	0.601
Modern terminal	U	T	9095	C	ATP6	Non-synonymous	L190P	0.052	0.1	0.601
Modern terminal	JT	T	9095	C	ATP6	Non-synonymous	L190P	0.052	0.1	0.601
Ancient terminal	R0	T	9098	C	ATP6	Non-synonymous	I191T	0.999	0	0.286
Modern terminal	R0	T	9098	C	ATP6	Non-synonymous	I191T	0.999	0	0.286
Modern terminal	R0	T	9098	C	ATP6	Non-synonymous	I191T	0.999	0	0.286
Modern terminal	R0	T	9098	C	ATP6	Non-synonymous	I191T	0.999	0	0.286
Pre-terminal	R0	T	9098	G	ATP6	Non-synonymous	I191S	1	0.04	0.494
Modern terminal	U	T	9098	C	ATP6	Non-synonymous	I191T	0.999	0	0.286
Modern terminal	U	T	9098	C	ATP6	Non-synonymous	I191T	0.999	0	0.286
Modern terminal	U	T	9098	G	ATP6	Non-synonymous	I191S	1	0.04	0.494
Pre-terminal	U	T	9098	C	ATP6	Non-synonymous	I191T	0.999	0	0.286
Pre-terminal	U	T	9098	C	ATP6	Non-synonymous	I191T	0.999	0	0.286
Pre-terminal	U	T	9098	C	ATP6	Non-synonymous	I191T	0.999	0	0.286
Modern terminal	N1	T	9098	C	ATP6	Non-synonymous	I191T	0.999	0	0.286
Pre-terminal	R0	A	9100	G	ATP6	Non-synonymous	I192V	0.059	0.1	0.101
Pre-terminal	R0	A	9100	G	ATP6	Non-synonymous	I192V	0.059	0.1	0.101
Pre-terminal	U	A	9100	G	ATP6	Non-synonymous	I192V	0.059	0.1	0.101
Pre-terminal	JT	A	9100	G	ATP6	Non-synonymous	I192V	0.059	0.1	0.101
Modern terminal	N2	A	9100	G	ATP6	Non-synonymous	I192V	0.059	0.1	0.101
Pre-terminal	N2	A	9100	G	ATP6	Non-synonymous	I192V	0.059	0.1	0.101
Modern terminal	R0	T	9101	C	ATP6	Non-synonymous	I192T	0.002	1	0.065
Modern terminal	R0	T	9101	C	ATP6	Non-synonymous	I192T	0.002	1	0.065
Modern terminal	R0	T	9101	G	ATP6	Non-synonymous	I192S	0.25	0.02	0.302
Pre-terminal	R0	T	9101	C	ATP6	Non-synonymous	I192T	0.002	1	0.065
Modern terminal	U	T	9101	C	ATP6	Non-synonymous	I192T	0.002	1	0.065
Pre-terminal	U	T	9101	C	ATP6	Non-synonymous	I192T	0.002	1	0.065
Pre-terminal	U	T	9101	C	ATP6	Non-synonymous	I192T	0.002	1	0.065
Pre-terminal	U	T	9101	C	ATP6	Non-synonymous	I192T	0.002	1	0.065
Pre-terminal	JT	T	9101	C	ATP6	Non-synonymous	I192T	0.002	1	0.065
Modern terminal	X	T	9101	C	ATP6	Non-synonymous	I192T	0.002	1	0.065
Modern terminal	X	T	9101	C	ATP6	Non-synonymous	I192T	0.002	1	0.065
Pre-terminal	R0	C	9102	A	ATP6	Non-synonymous	I192M	0.92	0.06	0.128
Modern terminal	U	C	9102	A	ATP6	Non-synonymous	I192M	0.92	0.06	0.128
Pre-terminal	JT	C	9102	A	ATP6	Non-synonymous	I192M	0.92	0.06	0.128
Modern terminal	R0	T	9103	C	ATP6	Non-synonymous	F193L	0.006	0.32	0.437

Modern terminal	R0	T	9103	C	ATP6	Non-synonymous	F193L	0.006	0.32	0.437
Modern terminal	R0	T	9103	C	ATP6	Non-synonymous	F193L	0.006	0.32	0.437
Modern terminal	R0	T	9103	C	ATP6	Non-synonymous	F193L	0.006	0.32	0.437
Modern terminal	R0	T	9103	C	ATP6	Non-synonymous	F193L	0.006	0.32	0.437
Pre-terminal	R0	T	9103	C	ATP6	Non-synonymous	F193L	0.006	0.32	0.437
Modern terminal	U	T	9103	C	ATP6	Non-synonymous	F193L	0.006	0.32	0.437
Modern terminal	JT	T	9103	C	ATP6	Non-synonymous	F193L	0.006	0.32	0.437
Modern terminal	JT	T	9103	C	ATP6	Non-synonymous	F193L	0.006	0.32	0.437
Pre-terminal	JT	T	9103	C	ATP6	Non-synonymous	F193L	0.006	0.32	0.437
Modern terminal	R0	T	9104	C	ATP6	Non-synonymous	F193S	0.99	0.25	0.652
Pre-terminal	R0	T	9104	C	ATP6	Non-synonymous	F193S	0.99	0.25	0.652
Modern terminal	U	T	9104	C	ATP6	Non-synonymous	F193S	0.99	0.25	0.652
Pre-terminal	R0	A	9106	G	ATP6	Non-synonymous	T194A	0.978	0.31	0.177
Modern terminal	U	C	9107	T	ATP6	Non-synonymous	T194M	0.999	0.06	0.153
Modern terminal	U	C	9107	T	ATP6	Non-synonymous	T194M	0.999	0.06	0.153
Modern terminal	R0	A	9109	G	ATP6	Non-synonymous	I195V	0	0.66	0.126
Modern terminal	R0	A	9109	G	ATP6	Non-synonymous	I195V	0	0.66	0.126
Pre-terminal	R0	A	9109	G	ATP6	Non-synonymous	I195V	0	0.66	0.126
Modern terminal	U	A	9109	G	ATP6	Non-synonymous	I195V	0	0.66	0.126
Pre-terminal	U	A	9109	G	ATP6	Non-synonymous	I195V	0	0.66	0.126
Modern terminal	R0	T	9110	C	ATP6	Non-synonymous	I195T	0.447	0.16	0.353
Modern terminal	R0	T	9110	C	ATP6	Non-synonymous	I195T	0.447	0.16	0.353
Modern terminal	R0	T	9110	C	ATP6	Non-synonymous	I195T	0.447	0.16	0.353
Modern terminal	R0	T	9110	C	ATP6	Non-synonymous	I195T	0.447	0.16	0.353
Pre-terminal	R0	T	9110	C	ATP6	Non-synonymous	I195T	0.447	0.16	0.353
Pre-terminal	R0	T	9110	C	ATP6	Non-synonymous	I195T	0.447	0.16	0.353
Pre-terminal	R0	T	9110	C	ATP6	Non-synonymous	I195T	0.447	0.16	0.353
Pre-terminal	R0	T	9110	C	ATP6	Non-synonymous	I195T	0.447	0.16	0.353
Modern terminal	U	T	9110	C	ATP6	Non-synonymous	I195T	0.447	0.16	0.353
Modern terminal	U	T	9110	C	ATP6	Non-synonymous	I195T	0.447	0.16	0.353
Pre-terminal	U	T	9110	C	ATP6	Non-synonymous	I195T	0.447	0.16	0.353
Modern terminal	JT	T	9110	C	ATP6	Non-synonymous	I195T	0.447	0.16	0.353
Modern terminal	JT	T	9110	C	ATP6	Non-synonymous	I195T	0.447	0.16	0.353
Modern terminal	N1	T	9110	C	ATP6	Non-synonymous	I195T	0.447	0.16	0.353
Modern terminal	N2	T	9110	C	ATP6	Non-synonymous	I195T	0.447	0.16	0.353
Modern terminal	R0	A	9115	G	ATP6	Non-synonymous	I197V	0.005	0.75	0.064
Pre-terminal	R0	A	9115	G	ATP6	Non-synonymous	I197V	0.005	0.75	0.064
Modern terminal	U	A	9115	G	ATP6	Non-synonymous	I197V	0.005	0.75	0.064
Pre-terminal	U	A	9115	G	ATP6	Non-synonymous	I197V	0.005	0.75	0.064
Modern terminal	JT	A	9115	G	ATP6	Non-synonymous	I197V	0.005	0.75	0.064
Modern terminal	N1	A	9115	G	ATP6	Non-synonymous	I197V	0.005	0.75	0.064
Modern terminal	R0	T	9116	C	ATP6	Non-synonymous	I197T	0.002	0.2	0.385
Modern terminal	R0	T	9116	C	ATP6	Non-synonymous	I197T	0.002	0.2	0.385
Modern terminal	R0	T	9116	C	ATP6	Non-synonymous	I197T	0.002	0.2	0.385
Pre-terminal	R0	T	9116	C	ATP6	Non-synonymous	I197T	0.002	0.2	0.385
Pre-terminal	R0	T	9116	C	ATP6	Non-synonymous	I197T	0.002	0.2	0.385
Ancient terminal	U	T	9116	C	ATP6	Non-synonymous	I197T	0.002	0.2	0.385
Ancient terminal	U	T	9116	C	ATP6	Non-synonymous	I197T	0.002	0.2	0.385
Modern terminal	U	T	9116	C	ATP6	Non-synonymous	I197T	0.002	0.2	0.385
Modern terminal	U	T	9116	C	ATP6	Non-synonymous	I197T	0.002	0.2	0.385

Modern terminal	JT	T	9116	C	ATP6	Non-synonymous	I197T	0.002	0.2	0.385
Modern terminal	N1	T	9116	C	ATP6	Non-synonymous	I197T	0.002	0.2	0.385
Modern terminal	U	A	9124	G	ATP6	Non-synonymous	T200A	0.97	0.02	0.51
Modern terminal	N2	C	9125	T	ATP6	Non-synonymous	T200I	0.996	0.01	0.527
Modern terminal	R0	A	9127	G	ATP6	Non-synonymous	I201V	0	0.25	0.122
Modern terminal	JT	A	9127	G	ATP6	Non-synonymous	I201V	0	0.25	0.122
Pre-terminal	N2	A	9127	G	ATP6	Non-synonymous	I201V	0	0.25	0.122
Modern terminal	R0	T	9128	C	ATP6	Non-synonymous	I201T	0.003	0.02	0.408
Modern terminal	R0	T	9128	C	ATP6	Non-synonymous	I201T	0.003	0.02	0.408
Pre-terminal	R0	T	9128	C	ATP6	Non-synonymous	I201T	0.003	0.02	0.408
Pre-terminal	R0	T	9128	C	ATP6	Non-synonymous	I201T	0.003	0.02	0.408
Pre-terminal	R0	T	9128	C	ATP6	Non-synonymous	I201T	0.003	0.02	0.408
Modern terminal	U	T	9128	C	ATP6	Non-synonymous	I201T	0.003	0.02	0.408
Modern terminal	U	T	9128	C	ATP6	Non-synonymous	I201T	0.003	0.02	0.408
Modern terminal	U	T	9128	C	ATP6	Non-synonymous	I201T	0.003	0.02	0.408
Pre-terminal	U	T	9128	C	ATP6	Non-synonymous	I201T	0.003	0.02	0.408
Pre-terminal	U	T	9128	C	ATP6	Non-synonymous	I201T	0.003	0.02	0.408
Modern terminal	JT	T	9128	C	ATP6	Non-synonymous	I201T	0.003	0.02	0.408
Modern terminal	JT	T	9128	C	ATP6	Non-synonymous	I201T	0.003	0.02	0.408
Modern terminal	N2	T	9128	C	ATP6	Non-synonymous	I201T	0.003	0.02	0.408
Modern terminal	U	G	9133	A	ATP6	Non-synonymous	E203K	0.993	0	0.665
Modern terminal	U	G	9133	A	ATP6	Non-synonymous	E203K	0.993	0	0.665
Modern terminal	U	G	9133	A	ATP6	Non-synonymous	E203K	0.993	0	0.665
Modern terminal	N1	G	9133	A	ATP6	Non-synonymous	E203K	0.993	0	0.665
Modern terminal	R0	T	9137	C	ATP6	Non-synonymous	I204T	0.002	0.08	0.453
Modern terminal	R0	C	9138	A	ATP6	Non-synonymous	I204M	0.85	0.14	0.305
Modern terminal	U	C	9138	A	ATP6	Non-synonymous	I204M	0.85	0.14	0.305
Modern terminal	JT	C	9138	A	ATP6	Non-synonymous	I204M	0.85	0.14	0.305
Modern terminal	R0	G	9139	A	ATP6	Non-synonymous	A205T	0.999	0	0.543
Modern terminal	R0	G	9139	A	ATP6	Non-synonymous	A205T	0.999	0	0.543
Modern terminal	R0	G	9139	A	ATP6	Non-synonymous	A205T	0.999	0	0.543
Pre-terminal	R0	G	9139	A	ATP6	Non-synonymous	A205T	0.999	0	0.543
Ancient terminal	U	G	9139	A	ATP6	Non-synonymous	A205T	0.999	0	0.543
Modern terminal	U	G	9139	A	ATP6	Non-synonymous	A205T	0.999	0	0.543
Modern terminal	U	G	9139	A	ATP6	Non-synonymous	A205T	0.999	0	0.543
Modern terminal	U	G	9139	A	ATP6	Non-synonymous	A205T	0.999	0	0.543
Pre-terminal	U	G	9139	A	ATP6	Non-synonymous	A205T	0.999	0	0.543
Pre-terminal	U	G	9139	A	ATP6	Non-synonymous	A205T	0.999	0	0.543
Pre-terminal	U	G	9139	A	ATP6	Non-synonymous	A205T	0.999	0	0.543
Modern terminal	JT	G	9139	A	ATP6	Non-synonymous	A205T	0.999	0	0.543
Modern terminal	JT	G	9139	A	ATP6	Non-synonymous	A205T	0.999	0	0.543
Pre-terminal	JT	G	9139	A	ATP6	Non-synonymous	A205T	0.999	0	0.543
Modern terminal	N1	G	9139	A	ATP6	Non-synonymous	A205T	0.999	0	0.543
Modern terminal	N2	G	9139	A	ATP6	Non-synonymous	A205T	0.999	0	0.543
Modern terminal	R0	G	9142	C	ATP6	Non-synonymous	V206L	0.022	0.03	0.532
Modern terminal	R0	G	9142	A	ATP6	Non-synonymous	V206I	0.001	0.03	0.269
Modern terminal	R0	G	9142	A	ATP6	Non-synonymous	V206I	0.001	0.03	0.269
Pre-terminal	R0	G	9142	A	ATP6	Non-synonymous	V206I	0.001	0.03	0.269
Ancient terminal	U	G	9142	A	ATP6	Non-synonymous	V206I	0.001	0.03	0.269
Modern terminal	U	G	9142	A	ATP6	Non-synonymous	V206I	0.001	0.03	0.269

Modern terminal	U	G	9142	C	ATP6	Non-synonymous	V206L	0.022	0.03	0.532
Pre-terminal	U	G	9142	A	ATP6	Non-synonymous	V206I	0.001	0.03	0.269
Ancient terminal	JT	T	9143	C	ATP6	Non-synonymous	V206A	0.991	0	0.574
Ancient terminal	R0	G	9145	T	ATP6	Non-synonymous	A207S	0.97	0.05	0.429
Ancient terminal	R0	G	9145	A	ATP6	Non-synonymous	A207T	0.999	0	0.563
Modern terminal	R0	G	9145	A	ATP6	Non-synonymous	A207T	0.999	0	0.563
Modern terminal	R0	G	9145	A	ATP6	Non-synonymous	A207T	0.999	0	0.563
Modern terminal	R0	G	9145	A	ATP6	Non-synonymous	A207T	0.999	0	0.563
Modern terminal	R0	G	9145	A	ATP6	Non-synonymous	A207T	0.999	0	0.563
Modern terminal	R0	G	9145	A	ATP6	Non-synonymous	A207T	0.999	0	0.563
Modern terminal	R0	G	9145	A	ATP6	Non-synonymous	A207T	0.999	0	0.563
Modern terminal	R0	G	9145	A	ATP6	Non-synonymous	A207T	0.999	0	0.563
Modern terminal	R0	G	9145	A	ATP6	Non-synonymous	A207T	0.999	0	0.563
Modern terminal	R0	G	9145	C	ATP6	Non-synonymous	A207P	1	0	0.798
Modern terminal	R0	G	9145	A	ATP6	Non-synonymous	A207T	0.999	0	0.563
Modern terminal	R0	G	9145	T	ATP6	Non-synonymous	A207S	0.97	0.05	0.429
Modern terminal	R0	G	9145	A	ATP6	Non-synonymous	A207T	0.999	0	0.563
Modern terminal	R0	G	9145	A	ATP6	Non-synonymous	A207T	0.999	0	0.563
Pre-terminal	R0	G	9145	A	ATP6	Non-synonymous	A207T	0.999	0	0.563
Pre-terminal	R0	G	9145	A	ATP6	Non-synonymous	A207T	0.999	0	0.563
Ancient terminal	U	G	9145	A	ATP6	Non-synonymous	A207T	0.999	0	0.563
Modern terminal	U	G	9145	A	ATP6	Non-synonymous	A207T	0.999	0	0.563
Modern terminal	U	G	9145	A	ATP6	Non-synonymous	A207T	0.999	0	0.563
Modern terminal	U	G	9145	A	ATP6	Non-synonymous	A207T	0.999	0	0.563
Modern terminal	JT	G	9145	A	ATP6	Non-synonymous	A207T	0.999	0	0.563
Modern terminal	JT	G	9145	A	ATP6	Non-synonymous	A207T	0.999	0	0.563
Modern terminal	JT	G	9145	A	ATP6	Non-synonymous	A207T	0.999	0	0.563
Pre-terminal	JT	G	9145	A	ATP6	Non-synonymous	A207T	0.999	0	0.563
Pre-terminal	JT	G	9145	A	ATP6	Non-synonymous	A207T	0.999	0	0.563
Pre-terminal	JT	G	9145	A	ATP6	Non-synonymous	A207T	0.999	0	0.563
Modern terminal	X	G	9145	A	ATP6	Non-synonymous	A207T	0.999	0	0.563
Modern terminal	N2	G	9145	A	ATP6	Non-synonymous	A207T	0.999	0	0.563
Modern terminal	N2	G	9145	A	ATP6	Non-synonymous	A207T	0.999	0	0.563
Modern terminal	R0	T	9149	C	ATP6	Non-synonymous	L208S	1	0	0.712
Pre-terminal	R0	T	9149	C	ATP6	Non-synonymous	L208S	1	0	0.712
Ancient terminal	JT	T	9149	C	ATP6	Non-synonymous	L208S	1	0	0.712
Modern terminal	R0	A	9151	G	ATP6	Non-synonymous	I209V	0.916	0.01	0.207
Modern terminal	R0	A	9151	G	ATP6	Non-synonymous	I209V	0.916	0.01	0.207
Pre-terminal	R0	A	9151	G	ATP6	Non-synonymous	I209V	0.916	0.01	0.207
Ancient terminal	U	A	9151	G	ATP6	Non-synonymous	I209V	0.916	0.01	0.207
Pre-terminal	X	A	9151	G	ATP6	Non-synonymous	I209V	0.916	0.01	0.207
Pre-terminal	X	A	9151	G	ATP6	Non-synonymous	I209V	0.916	0.01	0.207
Modern terminal	R0	T	9152	C	ATP6	Non-synonymous	I209T	1	0	0.502
Modern terminal	R0	T	9152	C	ATP6	Non-synonymous	I209T	1	0	0.502
Pre-terminal	R0	T	9152	C	ATP6	Non-synonymous	I209T	1	0	0.502
Modern terminal	U	T	9152	C	ATP6	Non-synonymous	I209T	1	0	0.502
Modern terminal	U	T	9152	C	ATP6	Non-synonymous	I209T	1	0	0.502
Ancient terminal	JT	T	9152	C	ATP6	Non-synonymous	I209T	1	0	0.502
Pre-terminal	JT	T	9152	C	ATP6	Non-synonymous	I209T	1	0	0.502
Ancient terminal	R0	G	9157	T	ATP6	Non-synonymous	A211S	0.97	0.12	0.29

Modern terminal	R0	G	9157	A	ATP6	Non-synonymous	A211T	0.999	0	0.396
Pre-terminal	R0	G	9157	A	ATP6	Non-synonymous	A211T	0.999	0	0.396
Ancient terminal	U	G	9157	A	ATP6	Non-synonymous	A211T	0.999	0	0.396
Modern terminal	JT	G	9157	A	ATP6	Non-synonymous	A211T	0.999	0	0.396
Modern terminal	R0	T	9160	C	ATP6	Non-synonymous	Y212H	1	0	0.54
Modern terminal	U	T	9160	C	ATP6	Non-synonymous	Y212H	1	0	0.54
Modern terminal	R0	G	9163	A	ATP6	Non-synonymous	V213I	0.818	0	0.153
Modern terminal	R0	G	9163	C	ATP6	Non-synonymous	V213L	0.818	0	0.281
Modern terminal	R0	G	9163	A	ATP6	Non-synonymous	V213I	0.818	0	0.153
Modern terminal	R0	G	9163	A	ATP6	Non-synonymous	V213I	0.818	0	0.153
Modern terminal	R0	G	9163	A	ATP6	Non-synonymous	V213I	0.818	0	0.153
Modern terminal	R0	G	9163	A	ATP6	Non-synonymous	V213I	0.818	0	0.153
Modern terminal	U	G	9163	A	ATP6	Non-synonymous	V213I	0.818	0	0.153
Pre-terminal	U	G	9163	A	ATP6	Non-synonymous	V213I	0.818	0	0.153
Modern terminal	JT	G	9163	A	ATP6	Non-synonymous	V213I	0.818	0	0.153
Modern terminal	JT	G	9163	A	ATP6	Non-synonymous	V213I	0.818	0	0.153
Modern terminal	JT	G	9163	A	ATP6	Non-synonymous	V213I	0.818	0	0.153
Modern terminal	N1	G	9163	A	ATP6	Non-synonymous	V213I	0.818	0	0.153
Modern terminal	N1	G	9163	A	ATP6	Non-synonymous	V213I	0.818	0	0.153
Pre-terminal	N2	G	9163	A	ATP6	Non-synonymous	V213I	0.818	0	0.153
Modern terminal	R0	T	9167	C	ATP6	Non-synonymous	F214S	1	0	0.746
Pre-terminal	R0	T	9167	C	ATP6	Non-synonymous	F214S	1	0	0.746
Ancient terminal	U	T	9167	C	ATP6	Non-synonymous	F214S	1	0	0.746
Modern terminal	U	T	9167	C	ATP6	Non-synonymous	F214S	1	0	0.746
Modern terminal	R0	C	9175	A	ATP6	Non-synonymous	L217M	1	0	0.217
Modern terminal	JT	C	9175	A	ATP6	Non-synonymous	L217M	1	0	0.217
Modern terminal	JT	T	9176	C	ATP6	Non-synonymous	L217P	1	0	0.766
Modern terminal	JT	T	9176	C	ATP6	Non-synonymous	L217P	1	0	0.766
Ancient terminal	R0	G	9178	A	ATP6	Non-synonymous	V218M	0.999	0.01	0.167
Modern terminal	U	G	9178	A	ATP6	Non-synonymous	V218M	0.999	0.01	0.167
Modern terminal	R0	A	9181	G	ATP6	Non-synonymous	S219G	1	0	0.163
Modern terminal	R0	A	9181	G	ATP6	Non-synonymous	S219G	1	0	0.163
Pre-terminal	R0	A	9181	G	ATP6	Non-synonymous	S219G	1	0	0.163
Pre-terminal	R0	A	9181	G	ATP6	Non-synonymous	S219G	1	0	0.163
Pre-terminal	R0	A	9181	G	ATP6	Non-synonymous	S219G	1	0	0.163
Modern terminal	U	A	9181	G	ATP6	Non-synonymous	S219G	1	0	0.163
Modern terminal	U	A	9181	G	ATP6	Non-synonymous	S219G	1	0	0.163
Modern terminal	U	A	9181	G	ATP6	Non-synonymous	S219G	1	0	0.163
Modern terminal	U	A	9181	G	ATP6	Non-synonymous	S219G	1	0	0.163
Modern terminal	JT	A	9181	G	ATP6	Non-synonymous	S219G	1	0	0.163
Modern terminal	JT	A	9181	G	ATP6	Non-synonymous	S219G	1	0	0.163
Pre-terminal	JT	A	9181	G	ATP6	Non-synonymous	S219G	1	0	0.163
Pre-terminal	JT	A	9181	G	ATP6	Non-synonymous	S219G	1	0	0.163
Modern terminal	N1	A	9181	G	ATP6	Non-synonymous	S219G	1	0	0.163
Ancient terminal	R0	G	9182	A	ATP6	Non-synonymous	S219N	0.962	0.03	0.203
Modern terminal	R0	G	9182	A	ATP6	Non-synonymous	S219N	0.962	0.03	0.203
Modern terminal	R0	G	9182	A	ATP6	Non-synonymous	S219N	0.962	0.03	0.203
Modern terminal	R0	G	9182	A	ATP6	Non-synonymous	S219N	0.962	0.03	0.203
Modern terminal	R0	G	9182	A	ATP6	Non-synonymous	S219N	0.962	0.03	0.203
Modern terminal	R0	G	9182	A	ATP6	Non-synonymous	S219N	0.962	0.03	0.203

Modern terminal	R0	G	9182	A	ATP6	Non-synonymous	S219N	0.962	0.03	0.203
Modern terminal	R0	G	9182	A	ATP6	Non-synonymous	S219N	0.962	0.03	0.203
Pre-terminal	R0	G	9182	A	ATP6	Non-synonymous	S219N	0.962	0.03	0.203
Pre-terminal	R0	G	9182	A	ATP6	Non-synonymous	S219N	0.962	0.03	0.203
Pre-terminal	R0	G	9182	A	ATP6	Non-synonymous	S219N	0.962	0.03	0.203
Modern terminal	U	G	9182	A	ATP6	Non-synonymous	S219N	0.962	0.03	0.203
Modern terminal	U	G	9182	A	ATP6	Non-synonymous	S219N	0.962	0.03	0.203
Pre-terminal	U	G	9182	A	ATP6	Non-synonymous	S219N	0.962	0.03	0.203
Modern terminal	N1	G	9182	A	ATP6	Non-synonymous	S219N	0.962	0.03	0.203
Modern terminal	N2	G	9182	A	ATP6	Non-synonymous	S219N	0.962	0.03	0.203
Ancient terminal	R0	C	9184	A	ATP6	Non-synonymous	L220I	0.994	0	0.109
Ancient terminal	R0	T	9185	C	ATP6	Non-synonymous	L220P	1	0	0.469
Pre-terminal	U	T	9185	G	ATP6	Non-synonymous	L220R	0.999	0	0.481
Pre-terminal	U	T	9185	G	ATP6	Non-synonymous	L220R	0.999	0	0.481
Modern terminal	N1	T	9185	G	ATP6	Non-synonymous	L220R	0.999	0	0.481
Modern terminal	U	T	9187	C	ATP6	Non-synonymous	Y221H	1	0	0.246
Modern terminal	R0	A	9188	G	ATP6	Non-synonymous	Y221C	1	0	0.288
Pre-terminal	R0	A	9188	T	ATP6	Non-synonymous	Y221F	0.999	0	0.17
Pre-terminal	R0	A	9188	G	ATP6	Non-synonymous	Y221C	1	0	0.288
Modern terminal	N1	A	9188	G	ATP6	Non-synonymous	Y221C	1	0	0.288
Modern terminal	R0	C	9193	A	ATP6	Non-synonymous	H223N	0.999	0.03	0.232
Modern terminal	U	C	9193	T	ATP6	Non-synonymous	H223Y	0.983	0.01	0.236
Modern terminal	R0	A	9194	G	ATP6	Non-synonymous	H223R	0.99	0.01	0.205
Modern terminal	R0	A	9194	G	ATP6	Non-synonymous	H223R	0.99	0.01	0.205
Modern terminal	R0	A	9194	G	ATP6	Non-synonymous	H223R	0.99	0.01	0.205
Modern terminal	U	A	9194	G	ATP6	Non-synonymous	H223R	0.99	0.01	0.205
Modern terminal	N1	A	9194	G	ATP6	Non-synonymous	H223R	0.99	0.01	0.205
Modern terminal	R0	G	9196	A	ATP6	Non-synonymous	D224N	0.159	0.05	0.176
Modern terminal	R0	G	9196	A	ATP6	Non-synonymous	D224N	0.159	0.05	0.176
Modern terminal	R0	G	9196	A	ATP6	Non-synonymous	D224N	0.159	0.05	0.176
Modern terminal	R0	G	9196	A	ATP6	Non-synonymous	D224N	0.159	0.05	0.176
Pre-terminal	R0	G	9196	A	ATP6	Non-synonymous	D224N	0.159	0.05	0.176
Pre-terminal	R0	G	9196	A	ATP6	Non-synonymous	D224N	0.159	0.05	0.176
Ancient terminal	U	G	9196	A	ATP6	Non-synonymous	D224N	0.159	0.05	0.176
Modern terminal	U	G	9196	A	ATP6	Non-synonymous	D224N	0.159	0.05	0.176
Modern terminal	U	G	9196	A	ATP6	Non-synonymous	D224N	0.159	0.05	0.176
Pre-terminal	U	G	9196	A	ATP6	Non-synonymous	D224N	0.159	0.05	0.176
Pre-terminal	U	G	9196	A	ATP6	Non-synonymous	D224N	0.159	0.05	0.176
Modern terminal	JT	G	9196	A	ATP6	Non-synonymous	D224N	0.159	0.05	0.176
Modern terminal	N2	G	9196	A	ATP6	Non-synonymous	D224N	0.159	0.05	0.176
Pre-terminal	U	A	9200	G	ATP6	Non-synonymous	N225S	0.995	0.05	0.121
Pre-terminal	JT	A	9200	G	ATP6	Non-synonymous	N225S	0.995	0.05	0.121
Modern terminal	N1	A	9200	G	ATP6	Non-synonymous	N225S	0.995	0.05	0.121
Ancient terminal	R0	A	9210	G	COX3	Non-synonymous	T2A	0	0.44	0.174
Pre-terminal	R0	A	9210	G	COX3	Non-synonymous	T2A	0	0.44	0.174
Pre-terminal	R0	A	9210	G	COX3	Non-synonymous	T2A	0	0.44	0.174
Pre-terminal	R0	A	9210	G	COX3	Non-synonymous	T2A	0	0.44	0.174
Modern terminal	U	A	9210	G	COX3	Non-synonymous	T2A	0	0.44	0.174
Modern terminal	U	A	9210	G	COX3	Non-synonymous	T2A	0	0.44	0.174
Modern terminal	U	A	9210	G	COX3	Non-synonymous	T2A	0	0.44	0.174

Pre-terminal	U	A	9210	G	COX3	Non-synonymous	T2A	0	0.44	0.174
Modern terminal	JT	A	9210	G	COX3	Non-synonymous	T2A	0	0.44	0.174
Modern terminal	JT	A	9210	G	COX3	Non-synonymous	T2A	0	0.44	0.174
Pre-terminal	JT	A	9210	G	COX3	Non-synonymous	T2A	0	0.44	0.174
Pre-terminal	JT	A	9210	G	COX3	Non-synonymous	T2A	0	0.44	0.174
Modern terminal	R0	C	9211	T	COX3	Non-synonymous	T2I	0	0	0.249
Pre-terminal	N2	C	9211	T	COX3	Non-synonymous	T2I	0	0	0.249
Modern terminal	R0	A	9214	G	COX3	Non-synonymous	H3R	0.787	0.02	0.147
Modern terminal	R0	A	9214	G	COX3	Non-synonymous	H3R	0.787	0.02	0.147
Modern terminal	R0	A	9214	G	COX3	Non-synonymous	H3R	0.787	0.02	0.147
Modern terminal	R0	A	9214	G	COX3	Non-synonymous	H3R	0.787	0.02	0.147
Pre-terminal	R0	A	9214	C	COX3	Non-synonymous	H3P	0.998	0	0.262
Ancient terminal	U	A	9214	G	COX3	Non-synonymous	H3R	0.787	0.02	0.147
Modern terminal	U	A	9214	G	COX3	Non-synonymous	H3R	0.787	0.02	0.147
Modern terminal	U	A	9214	G	COX3	Non-synonymous	H3R	0.787	0.02	0.147
Modern terminal	U	A	9214	G	COX3	Non-synonymous	H3R	0.787	0.02	0.147
Modern terminal	U	A	9214	G	COX3	Non-synonymous	H3R	0.787	0.02	0.147
Modern terminal	U	A	9214	G	COX3	Non-synonymous	H3R	0.787	0.02	0.147
Modern terminal	JT	A	9214	G	COX3	Non-synonymous	H3R	0.787	0.02	0.147
Modern terminal	JT	A	9214	G	COX3	Non-synonymous	H3R	0.787	0.02	0.147
Modern terminal	JT	A	9214	G	COX3	Non-synonymous	H3R	0.787	0.02	0.147
Pre-terminal	JT	A	9214	G	COX3	Non-synonymous	H3R	0.787	0.02	0.147
Modern terminal	N1	A	9214	C	COX3	Non-synonymous	H3P	0.998	0	0.262
Modern terminal	N1	A	9214	G	COX3	Non-synonymous	H3R	0.787	0.02	0.147
Pre-terminal	JT	T	9219	G	COX3	Non-synonymous	S5A	0	0.84	0.088
Modern terminal	R0	G	9225	A	COX3	Non-synonymous	A7T	0.254	0.01	0.095
Ancient terminal	R0	C	9226	T	COX3	Non-synonymous	A7V	0.421	0	0.114
Ancient terminal	X	T	9228	C	COX3	Non-synonymous	Y8H	1	0	0.273
Modern terminal	U	A	9234	G	COX3	Non-synonymous	M10V	0.996	0.01	0.242
Modern terminal	JT	A	9234	G	COX3	Non-synonymous	M10V	0.996	0.01	0.242
Pre-terminal	JT	A	9234	G	COX3	Non-synonymous	M10V	0.996	0.01	0.242
Pre-terminal	R0	A	9240	G	COX3	Non-synonymous	K12E	0.996	0.38	0.145
Ancient terminal	U	C	9258	A	COX3	Non-synonymous	L18M	1	0.01	0.185
Modern terminal	R0	A	9261	G	COX3	Non-synonymous	T19A	0.002	0.39	0.235
Pre-terminal	JT	A	9261	G	COX3	Non-synonymous	T19A	0.002	0.39	0.235
Modern terminal	R0	C	9262	T	COX3	Non-synonymous	T19M	0.976	0.14	0.159
Modern terminal	X	C	9262	T	COX3	Non-synonymous	T19M	0.976	0.14	0.159
Modern terminal	U	G	9265	A	COX3	Non-synonymous	G20E	1	0.01	0.628
Ancient terminal	R0	G	9276	A	COX3	Non-synonymous	A24T	0.003	0.01	0.337
Pre-terminal	R0	G	9276	A	COX3	Non-synonymous	A24T	0.003	0.01	0.337
Ancient terminal	U	G	9276	A	COX3	Non-synonymous	A24T	0.003	0.01	0.337
Modern terminal	U	G	9276	A	COX3	Non-synonymous	A24T	0.003	0.01	0.337
Pre-terminal	U	G	9276	A	COX3	Non-synonymous	A24T	0.003	0.01	0.337
Modern terminal	JT	G	9276	A	COX3	Non-synonymous	A24T	0.003	0.01	0.337
Pre-terminal	JT	G	9276	A	COX3	Non-synonymous	A24T	0.003	0.01	0.337
Modern terminal	R0	C	9282	A	COX3	Non-synonymous	L26M	0.998	0.21	0.371
Pre-terminal	R0	A	9285	G	COX3	Non-synonymous	M27V	0.173	0.12	0.301
Modern terminal	U	A	9285	G	COX3	Non-synonymous	M27V	0.173	0.12	0.301
Pre-terminal	U	A	9285	G	COX3	Non-synonymous	M27V	0.173	0.12	0.301
Modern terminal	R0	T	9286	C	COX3	Non-synonymous	M27T	0	0.42	0.44

Pre-terminal	R0	T	9286	C	COX3	Non-synonymous	M27T	0	0.42	0.44
Pre-terminal	U	T	9286	C	COX3	Non-synonymous	M27T	0	0.42	0.44
Pre-terminal	JT	T	9286	C	COX3	Non-synonymous	M27T	0	0.42	0.44
Pre-terminal	N1	T	9286	C	COX3	Non-synonymous	M27T	0	0.42	0.44
Modern terminal	R0	A	9288	G	COX3	Non-synonymous	T28A	0.988	0.09	0.344
Modern terminal	R0	A	9288	G	COX3	Non-synonymous	T28A	0.988	0.09	0.344
Modern terminal	U	A	9288	G	COX3	Non-synonymous	T28A	0.988	0.09	0.344
Modern terminal	U	A	9288	G	COX3	Non-synonymous	T28A	0.988	0.09	0.344
Modern terminal	U	A	9288	G	COX3	Non-synonymous	T28A	0.988	0.09	0.344
Modern terminal	U	A	9288	G	COX3	Non-synonymous	T28A	0.988	0.09	0.344
Pre-terminal	U	A	9288	G	COX3	Non-synonymous	T28A	0.988	0.09	0.344
Modern terminal	JT	A	9288	G	COX3	Non-synonymous	T28A	0.988	0.09	0.344
Modern terminal	JT	A	9288	G	COX3	Non-synonymous	T28A	0.988	0.09	0.344
Modern terminal	JT	A	9288	G	COX3	Non-synonymous	T28A	0.988	0.09	0.344
Modern terminal	U	G	9294	T	COX3	Non-synonymous	G30C	1	0.04	0.455
Modern terminal	R0	G	9300	A	COX3	Non-synonymous	A32T	0	0.24	0.076
Modern terminal	R0	G	9300	A	COX3	Non-synonymous	A32T	0	0.24	0.076
Modern terminal	R0	G	9300	A	COX3	Non-synonymous	A32T	0	0.24	0.076
Modern terminal	R0	G	9300	A	COX3	Non-synonymous	A32T	0	0.24	0.076
Modern terminal	R0	G	9300	A	COX3	Non-synonymous	A32T	0	0.24	0.076
Modern terminal	R0	G	9300	A	COX3	Non-synonymous	A32T	0	0.24	0.076
Modern terminal	R0	G	9300	A	COX3	Non-synonymous	A32T	0	0.24	0.076
Modern terminal	R0	G	9300	A	COX3	Non-synonymous	A32T	0	0.24	0.076
Modern terminal	R0	G	9300	A	COX3	Non-synonymous	A32T	0	0.24	0.076
Pre-terminal	R0	G	9300	A	COX3	Non-synonymous	A32T	0	0.24	0.076
Pre-terminal	R0	G	9300	A	COX3	Non-synonymous	A32T	0	0.24	0.076
Modern terminal	U	G	9300	A	COX3	Non-synonymous	A32T	0	0.24	0.076
Modern terminal	U	G	9300	A	COX3	Non-synonymous	A32T	0	0.24	0.076
Modern terminal	U	G	9300	A	COX3	Non-synonymous	A32T	0	0.24	0.076
Modern terminal	U	G	9300	A	COX3	Non-synonymous	A32T	0	0.24	0.076
Modern terminal	U	G	9300	A	COX3	Non-synonymous	A32T	0	0.24	0.076
Modern terminal	U	G	9300	A	COX3	Non-synonymous	A32T	0	0.24	0.076
Modern terminal	U	G	9300	A	COX3	Non-synonymous	A32T	0	0.24	0.076
Modern terminal	U	G	9300	A	COX3	Non-synonymous	A32T	0	0.24	0.076
Modern terminal	U	G	9300	A	COX3	Non-synonymous	A32T	0	0.24	0.076
Modern terminal	U	G	9300	A	COX3	Non-synonymous	A32T	0	0.24	0.076
Modern terminal	U	G	9300	A	COX3	Non-synonymous	A32T	0	0.24	0.076
Modern terminal	U	G	9300	A	COX3	Non-synonymous	A32T	0	0.24	0.076
Modern terminal	U	G	9300	A	COX3	Non-synonymous	A32T	0	0.24	0.076
Pre-terminal	U	G	9300	A	COX3	Non-synonymous	A32T	0	0.24	0.076
Pre-terminal	U	G	9300	A	COX3	Non-synonymous	A32T	0	0.24	0.076
Pre-terminal	U	G	9300	A	COX3	Non-synonymous	A32T	0	0.24	0.076
Modern terminal	JT	G	9300	A	COX3	Non-synonymous	A32T	0	0.24	0.076
Modern terminal	JT	G	9300	A	COX3	Non-synonymous	A32T	0	0.24	0.076
Modern terminal	JT	G	9300	A	COX3	Non-synonymous	A32T	0	0.24	0.076
Modern terminal	JT	G	9300	A	COX3	Non-synonymous	A32T	0	0.24	0.076
Pre-terminal	JT	G	9300	A	COX3	Non-synonymous	A32T	0	0.24	0.076
Pre-terminal	JT	G	9300	A	COX3	Non-synonymous	A32T	0	0.24	0.076
Pre-terminal	JT	G	9300	A	COX3	Non-synonymous	A32T	0	0.24	0.076
Pre-terminal	JT	G	9300	A	COX3	Non-synonymous	A32T	0	0.24	0.076

Modern terminal	N1	G	9300	A	COX3	Non-synonymous	A32T	0	0.24	0.076
Pre-terminal	R0	C	9301	T	COX3	Non-synonymous	A32V	0	1	0.075
Modern terminal	U	C	9301	T	COX3	Non-synonymous	A32V	0	1	0.075
Ancient terminal	X	G	9307	A	COX3	Nonsense				
Modern terminal	R0	T	9309	C	COX3	Non-synonymous	F35L	0.997	0.16	0.271
Modern terminal	JT	T	9309	C	COX3	Non-synonymous	F35L	0.997	0.16	0.271
Pre-terminal	JT	T	9309	C	COX3	Non-synonymous	F35L	0.997	0.16	0.271
Pre-terminal	JT	T	9309	C	COX3	Non-synonymous	F35L	0.997	0.16	0.271
Modern terminal	R0	T	9310	C	COX3	Non-synonymous	F35S	1	0	0.551
Pre-terminal	R0	T	9315	C	COX3	Non-synonymous	F37L	0.004	0.54	0.251
Modern terminal	U	T	9315	C	COX3	Non-synonymous	F37L	0.004	0.54	0.251
Modern terminal	R0	T	9316	C	COX3	Non-synonymous	F37S	0.985	0.44	0.57
Modern terminal	R0	T	9316	C	COX3	Non-synonymous	F37S	0.985	0.44	0.57
Modern terminal	R0	T	9316	C	COX3	Non-synonymous	F37S	0.985	0.44	0.57
Pre-terminal	R0	T	9316	C	COX3	Non-synonymous	F37S	0.985	0.44	0.57
Pre-terminal	JT	T	9316	C	COX3	Non-synonymous	F37S	0.985	0.44	0.57
Modern terminal	R0	A	9324	G	COX3	Non-synonymous	M40V	0.006	0.5	0.087
Pre-terminal	R0	A	9324	G	COX3	Non-synonymous	M40V	0.006	0.5	0.087
Modern terminal	U	A	9324	G	COX3	Non-synonymous	M40V	0.006	0.5	0.087
Pre-terminal	JT	A	9324	G	COX3	Non-synonymous	M40V	0.006	0.5	0.087
Modern terminal	N1	A	9324	G	COX3	Non-synonymous	M40V	0.006	0.5	0.087
Modern terminal	R0	T	9325	C	COX3	Non-synonymous	M40T	0	0.46	0.066
Pre-terminal	R0	T	9325	C	COX3	Non-synonymous	M40T	0	0.46	0.066
Pre-terminal	R0	T	9325	C	COX3	Non-synonymous	M40T	0	0.46	0.066
Modern terminal	U	T	9325	C	COX3	Non-synonymous	M40T	0	0.46	0.066
Modern terminal	U	T	9325	C	COX3	Non-synonymous	M40T	0	0.46	0.066
Modern terminal	U	T	9325	C	COX3	Non-synonymous	M40T	0	0.46	0.066
Modern terminal	U	T	9325	C	COX3	Non-synonymous	M40T	0	0.46	0.066
Pre-terminal	N1	T	9325	C	COX3	Non-synonymous	M40T	0	0.46	0.066
Pre-terminal	N1	T	9325	C	COX3	Non-synonymous	M40T	0	0.46	0.066
Modern terminal	R0	A	9327	G	COX3	Non-synonymous	T41A	0	0.51	0.099
Modern terminal	U	A	9327	G	COX3	Non-synonymous	T41A	0	0.51	0.099
Modern terminal	JT	A	9327	G	COX3	Non-synonymous	T41A	0	0.51	0.099
Modern terminal	R0	C	9328	T	COX3	Non-synonymous	T41M	0.511	0.23	0.133
Modern terminal	R0	T	9331	C	COX3	Non-synonymous	L42P	1	0	0.515
Modern terminal	JT	T	9331	C	COX3	Non-synonymous	L42P	1	0	0.515
Modern terminal	R0	A	9336	G	COX3	Non-synonymous	M44V	0	0.41	0.134
Modern terminal	R0	A	9336	G	COX3	Non-synonymous	M44V	0	0.41	0.134
Modern terminal	R0	A	9336	G	COX3	Non-synonymous	M44V	0	0.41	0.134
Pre-terminal	R0	A	9336	G	COX3	Non-synonymous	M44V	0	0.41	0.134
Modern terminal	U	A	9336	G	COX3	Non-synonymous	M44V	0	0.41	0.134
Modern terminal	U	A	9336	G	COX3	Non-synonymous	M44V	0	0.41	0.134
Modern terminal	U	A	9336	G	COX3	Non-synonymous	M44V	0	0.41	0.134
Modern terminal	U	A	9336	G	COX3	Non-synonymous	M44V	0	0.41	0.134
Modern terminal	U	A	9336	G	COX3	Non-synonymous	M44V	0	0.41	0.134
Modern terminal	JT	A	9336	G	COX3	Non-synonymous	M44V	0	0.41	0.134
Modern terminal	JT	A	9336	G	COX3	Non-synonymous	M44V	0	0.41	0.134
Modern terminal	N1	A	9336	G	COX3	Non-synonymous	M44V	0	0.41	0.134
Modern terminal	R0	T	9337	C	COX3	Non-synonymous	M44T	0	0.61	0.115
Modern terminal	R0	T	9337	C	COX3	Non-synonymous	M44T	0	0.61	0.115

Pre-terminal	R0	T	9337	C	COX3	Non-synonymous	M44T	0	0.61	0.115
Modern terminal	R0	A	9355	G	COX3	Non-synonymous	N50S	0.001	0.02	0.357
Pre-terminal	R0	A	9355	G	COX3	Non-synonymous	N50S	0.001	0.02	0.357
Modern terminal	JT	A	9355	G	COX3	Non-synonymous	N50S	0.001	0.02	0.357
Modern terminal	R0	A	9357	G	COX3	Non-synonymous	T51A	0	0.01	0.195
Modern terminal	R0	C	9358	T	COX3	Non-synonymous	T51M	0	0.26	0.127
Modern terminal	R0	A	9366	T	COX3	Non-synonymous	M54L	0	0.04	0.565
Modern terminal	R0	T	9367	C	COX3	Non-synonymous	M54T	0.174	0	0.59
Modern terminal	R0	A	9370	G	COX3	Non-synonymous	Y55C	0.99	0.03	0.589
Ancient terminal	N2	G	9380	C	COX3	Non-synonymous	W58C	1	0	0.892
Modern terminal	R0	C	9391	T	COX3	Non-synonymous	T62M	0.001	0	0.143
Modern terminal	R0	C	9391	T	COX3	Non-synonymous	T62M	0.001	0	0.143
Pre-terminal	R0	C	9391	T	COX3	Non-synonymous	T62M	0.001	0	0.143
Ancient terminal	R0	C	9403	T	COX3	Non-synonymous	T66M	0.999	0	0.167
Ancient terminal	X	A	9420	C	COX3	Non-synonymous	T72P	1	0	0.473
Modern terminal	U	C	9423	T	COX3	Non-synonymous	P73S	0.009	0.52	0.265
Modern terminal	U	C	9424	A	COX3	Non-synonymous	P73Q	0.024	0.29	0.342
Pre-terminal	U	C	9426	T	COX3	Non-synonymous	P74S	0.009	0.41	0.153
Modern terminal	N2	G	9429	A	COX3	Non-synonymous	V75I	0.637	0	0.169
Ancient terminal	R0	A	9435	G	COX3	Non-synonymous	K77E	0.996	0.19	0.423
Modern terminal	R0	G	9438	A	COX3	Non-synonymous	G78S	0.003	0.04	0.477
Modern terminal	R0	G	9438	A	COX3	Non-synonymous	G78S	0.003	0.04	0.477
Modern terminal	R0	G	9438	A	COX3	Non-synonymous	G78S	0.003	0.04	0.477
Modern terminal	R0	G	9438	A	COX3	Non-synonymous	G78S	0.003	0.04	0.477
Modern terminal	R0	G	9438	A	COX3	Non-synonymous	G78S	0.003	0.04	0.477
Modern terminal	R0	G	9438	A	COX3	Non-synonymous	G78S	0.003	0.04	0.477
Modern terminal	R0	G	9438	A	COX3	Non-synonymous	G78S	0.003	0.04	0.477
Pre-terminal	R0	G	9438	A	COX3	Non-synonymous	G78S	0.003	0.04	0.477
Pre-terminal	R0	G	9438	A	COX3	Non-synonymous	G78S	0.003	0.04	0.477
Modern terminal	U	G	9438	A	COX3	Non-synonymous	G78S	0.003	0.04	0.477
Modern terminal	U	G	9438	A	COX3	Non-synonymous	G78S	0.003	0.04	0.477
Modern terminal	U	G	9438	A	COX3	Non-synonymous	G78S	0.003	0.04	0.477
Modern terminal	U	G	9438	A	COX3	Non-synonymous	G78S	0.003	0.04	0.477
Pre-terminal	U	G	9438	A	COX3	Non-synonymous	G78S	0.003	0.04	0.477
Modern terminal	JT	G	9438	A	COX3	Non-synonymous	G78S	0.003	0.04	0.477
Modern terminal	JT	G	9438	A	COX3	Non-synonymous	G78S	0.003	0.04	0.477
Pre-terminal	JT	G	9438	A	COX3	Non-synonymous	G78S	0.003	0.04	0.477
Pre-terminal	JT	G	9438	A	COX3	Non-synonymous	G78S	0.003	0.04	0.477
Pre-terminal	JT	G	9438	A	COX3	Non-synonymous	G78S	0.003	0.04	0.477
Pre-terminal	JT	G	9438	A	COX3	Non-synonymous	G78S	0.003	0.04	0.477
Pre-terminal	JT	G	9438	A	COX3	Non-synonymous	G78S	0.003	0.04	0.477
Modern terminal	N1	G	9438	A	COX3	Non-synonymous	G78S	0.003	0.04	0.477
Pre-terminal	N1	G	9438	A	COX3	Non-synonymous	G78S	0.003	0.04	0.477
Pre-terminal	N1	G	9438	A	COX3	Non-synonymous	G78S	0.003	0.04	0.477
Pre-terminal	N1	G	9438	A	COX3	Non-synonymous	G78S	0.003	0.04	0.477
Modern terminal	X	G	9438	A	COX3	Non-synonymous	G78S	0.003	0.04	0.477
Modern terminal	N2	G	9438	A	COX3	Non-synonymous	G78S	0.003	0.04	0.477
Modern terminal	R0	C	9444	T	COX3	Non-synonymous	R80W	1	0	0.586
Modern terminal	JT	G	9445	A	COX3	Non-synonymous	R80Q	1	0.07	0.381
Modern terminal	R0	A	9448	G	COX3	Non-synonymous	Y81C	1	0	0.569

Modern terminal	R0	A	9448	G	COX3	Non-synonymous	Y81C	1	0	0.569
Modern terminal	U	A	9448	G	COX3	Non-synonymous	Y81C	1	0	0.569
Modern terminal	JT	A	9448	G	COX3	Non-synonymous	Y81C	1	0	0.569
Modern terminal	JT	A	9448	G	COX3	Non-synonymous	Y81C	1	0	0.569
Ancient terminal	R0	A	9456	G	COX3	Non-synonymous	I84V	0	0.17	0.208
Pre-terminal	R0	A	9456	G	COX3	Non-synonymous	I84V	0	0.17	0.208
Modern terminal	U	A	9456	G	COX3	Non-synonymous	I84V	0	0.17	0.208
Modern terminal	U	A	9456	G	COX3	Non-synonymous	I84V	0	0.17	0.208
Pre-terminal	U	A	9456	G	COX3	Non-synonymous	I84V	0	0.17	0.208
Modern terminal	JT	A	9456	G	COX3	Non-synonymous	I84V	0	0.17	0.208
Pre-terminal	N1	A	9456	G	COX3	Non-synonymous	I84V	0	0.17	0.208
Pre-terminal	R0	A	9468	G	COX3	Non-synonymous	T88A	0.013	0.12	0.316
Pre-terminal	R0	A	9468	G	COX3	Non-synonymous	T88A	0.013	0.12	0.316
Modern terminal	U	A	9468	G	COX3	Non-synonymous	T88A	0.013	0.12	0.316
Modern terminal	U	A	9468	G	COX3	Non-synonymous	T88A	0.013	0.12	0.316
Modern terminal	N1	A	9468	G	COX3	Non-synonymous	T88A	0.013	0.12	0.316
Modern terminal	X	A	9468	G	COX3	Non-synonymous	T88A	0.013	0.12	0.316
Pre-terminal	X	A	9468	G	COX3	Non-synonymous	T88A	0.013	0.12	0.316
Pre-terminal	U	C	9469	T	COX3	Non-synonymous	T88I	0	0.98	0.198
Pre-terminal	JT	C	9469	T	COX3	Non-synonymous	T88I	0	0.98	0.198
Pre-terminal	N1	C	9469	T	COX3	Non-synonymous	T88I	0	0.98	0.198
Ancient terminal	U	T	9471	C	COX3	Non-synonymous	S89P	0.988	0	0.89
Modern terminal	R0	G	9477	A	COX3	Non-synonymous	V91I	0	0.19	0.328
Modern terminal	R0	G	9477	A	COX3	Non-synonymous	V91I	0	0.19	0.328
Modern terminal	R0	G	9477	A	COX3	Non-synonymous	V91I	0	0.19	0.328
Modern terminal	R0	G	9477	A	COX3	Non-synonymous	V91I	0	0.19	0.328
Modern terminal	R0	G	9477	A	COX3	Non-synonymous	V91I	0	0.19	0.328
Pre-terminal	R0	G	9477	A	COX3	Non-synonymous	V91I	0	0.19	0.328
Pre-terminal	R0	G	9477	A	COX3	Non-synonymous	V91I	0	0.19	0.328
Modern terminal	U	G	9477	A	COX3	Non-synonymous	V91I	0	0.19	0.328
Modern terminal	U	G	9477	A	COX3	Non-synonymous	V91I	0	0.19	0.328
Modern terminal	U	G	9477	A	COX3	Non-synonymous	V91I	0	0.19	0.328
Modern terminal	U	G	9477	A	COX3	Non-synonymous	V91I	0	0.19	0.328
Modern terminal	JT	G	9477	A	COX3	Non-synonymous	V91I	0	0.19	0.328
Modern terminal	JT	G	9477	A	COX3	Non-synonymous	V91I	0	0.19	0.328
Pre-terminal	JT	G	9477	A	COX3	Non-synonymous	V91I	0	0.19	0.328
Pre-terminal	JT	G	9477	A	COX3	Non-synonymous	V91I	0	0.19	0.328
Modern terminal	X	G	9477	A	COX3	Non-synonymous	V91I	0	0.19	0.328
Modern terminal	R0	T	9478	C	COX3	Non-synonymous	V91A	0.014	0.12	0.63
Modern terminal	R0	T	9478	C	COX3	Non-synonymous	V91A	0.014	0.12	0.63
Modern terminal	R0	T	9478	C	COX3	Non-synonymous	V91A	0.014	0.12	0.63
Pre-terminal	R0	T	9478	C	COX3	Non-synonymous	V91A	0.014	0.12	0.63
Modern terminal	U	T	9478	C	COX3	Non-synonymous	V91A	0.014	0.12	0.63
Modern terminal	U	T	9478	C	COX3	Non-synonymous	V91A	0.014	0.12	0.63
Modern terminal	U	T	9478	C	COX3	Non-synonymous	V91A	0.014	0.12	0.63
Modern terminal	JT	T	9478	C	COX3	Non-synonymous	V91A	0.014	0.12	0.63
Pre-terminal	U	T	9480	C	COX3	Non-synonymous	F92L	0.998	1	0.585
Modern terminal	R0	G	9489	A	COX3	Non-synonymous	A95T	0.001	0.48	0.221
Modern terminal	R0	G	9489	A	COX3	Non-synonymous	A95T	0.001	0.48	0.221
Modern terminal	R0	G	9489	A	COX3	Non-synonymous	A95T	0.001	0.48	0.221

Pre-terminal	R0	G	9489	A	COX3	Non-synonymous	A95T	0.001	0.48	0.221
Pre-terminal	R0	G	9489	A	COX3	Non-synonymous	A95T	0.001	0.48	0.221
Modern terminal	U	G	9489	A	COX3	Non-synonymous	A95T	0.001	0.48	0.221
Pre-terminal	U	G	9489	A	COX3	Non-synonymous	A95T	0.001	0.48	0.221
Ancient terminal	R0	C	9490	A	COX3	Non-synonymous	A95E	0.465	0.05	0.526
Modern terminal	R0	C	9490	T	COX3	Non-synonymous	A95V	0.001	1	0.345
Pre-terminal	JT	C	9490	T	COX3	Non-synonymous	A95V	0.001	1	0.345
Pre-terminal	U	T	9495	C	COX3	Non-synonymous	F97L	0.998	0.07	0.577
Modern terminal	U	T	9498	C	COX3	Non-synonymous	F98L	0.997	0	0.514
Pre-terminal	JT	T	9498	C	COX3	Non-synonymous	F98L	0.997	0	0.514
Modern terminal	U	C	9500	G	COX3	Non-synonymous	F98L	0.997	0	0.514
Modern terminal	U	A	9519	G	COX3	Non-synonymous	S105G	0.997	0	0.291
Modern terminal	R0	G	9525	A	COX3	Non-synonymous	A107T	0.989	0.1	0.23
Modern terminal	R0	G	9525	A	COX3	Non-synonymous	A107T	0.989	0.1	0.23
Modern terminal	R0	G	9525	A	COX3	Non-synonymous	A107T	0.989	0.1	0.23
Modern terminal	R0	G	9525	A	COX3	Non-synonymous	A107T	0.989	0.1	0.23
Pre-terminal	R0	G	9525	A	COX3	Non-synonymous	A107T	0.989	0.1	0.23
Pre-terminal	JT	G	9525	A	COX3	Non-synonymous	A107T	0.989	0.1	0.23
Modern terminal	R0	A	9531	G	COX3	Non-synonymous	T109A	0.967	0.15	0.225
Modern terminal	R0	A	9531	G	COX3	Non-synonymous	T109A	0.967	0.15	0.225
Modern terminal	R0	A	9531	G	COX3	Non-synonymous	T109A	0.967	0.15	0.225
Modern terminal	R0	A	9531	G	COX3	Non-synonymous	T109A	0.967	0.15	0.225
Pre-terminal	R0	A	9531	G	COX3	Non-synonymous	T109A	0.967	0.15	0.225
Pre-terminal	R0	A	9531	G	COX3	Non-synonymous	T109A	0.967	0.15	0.225
Ancient terminal	U	A	9531	G	COX3	Non-synonymous	T109A	0.967	0.15	0.225
Pre-terminal	U	A	9531	G	COX3	Non-synonymous	T109A	0.967	0.15	0.225
Pre-terminal	U	A	9531	G	COX3	Non-synonymous	T109A	0.967	0.15	0.225
Modern terminal	JT	A	9531	G	COX3	Non-synonymous	T109A	0.967	0.15	0.225
Modern terminal	JT	A	9531	G	COX3	Non-synonymous	T109A	0.967	0.15	0.225
Modern terminal	JT	A	9531	G	COX3	Non-synonymous	T109A	0.967	0.15	0.225
Pre-terminal	JT	A	9531	G	COX3	Non-synonymous	T109A	0.967	0.15	0.225
Modern terminal	N1	A	9531	G	COX3	Non-synonymous	T109A	0.967	0.15	0.225
Modern terminal	N2	A	9531	G	COX3	Non-synonymous	T109A	0.967	0.15	0.225
Ancient terminal	U	C	9534	A	COX3	Non-synonymous	P110T	0.993	0.46	0.244
Modern terminal	U	T	9541	C	COX3	Non-synonymous	L112S	1	0	0.392
Modern terminal	U	T	9541	C	COX3	Non-synonymous	L112S	1	0	0.392
Pre-terminal	JT	T	9541	C	COX3	Non-synonymous	L112S	1	0	0.392
Modern terminal	R0	A	9550	G	COX3	Non-synonymous	H115R	0.889	0.27	0.227
Ancient terminal	JT	C	9556	A	COX3	Non-synonymous	P117H	1	0	0.571
Ancient terminal	U	C	9559	A	COX3	Non-synonymous	P118Q	0.999	0	0.439
Modern terminal	JT	C	9559	G	COX3	Non-synonymous	P118R	0.999	0	0.485
Ancient terminal	U	A	9561	C	COX3	Non-synonymous	T119P	0.999	0.09	0.545
Ancient terminal	JT	C	9569	G	COX3	Non-synonymous	I121M	0.998	0	0.306
Pre-terminal	U	C	9571	T	COX3	Non-synonymous	T122I	0	0.41	0.142
Modern terminal	U	C	9573	A	COX3	Non-synonymous	P123T	0.993	0.33	0.484
Modern terminal	JT	T	9577	C	COX3	Non-synonymous	L124P	1	0	0.824
Modern terminal	R0	A	9579	G	COX3	Non-synonymous	N125D	0.002	0.44	0.279
Ancient terminal	U	C	9582	A	COX3	Non-synonymous	P126T	0.993	0	0.38
Ancient terminal	R0	T	9586	A	COX3	Non-synonymous	L127Q	0.995	0.01	0.612
Modern terminal	JT	G	9588	A	COX3	Non-synonymous	E128K	0.946	0.05	0.442

Pre-terminal	JT	G	9588	A	COX3	Non-synonymous	E128K	0.946	0.05	0.442
Modern terminal	R0	G	9591	A	COX3	Non-synonymous	V129I	0.264	0.5	0.148
Modern terminal	R0	G	9591	A	COX3	Non-synonymous	V129I	0.264	0.5	0.148
Modern terminal	R0	G	9591	A	COX3	Non-synonymous	V129I	0.264	0.5	0.148
Modern terminal	R0	G	9591	A	COX3	Non-synonymous	V129I	0.264	0.5	0.148
Modern terminal	R0	G	9591	A	COX3	Non-synonymous	V129I	0.264	0.5	0.148
Modern terminal	R0	G	9591	A	COX3	Non-synonymous	V129I	0.264	0.5	0.148
Modern terminal	R0	G	9591	A	COX3	Non-synonymous	V129I	0.264	0.5	0.148
Modern terminal	R0	G	9591	A	COX3	Non-synonymous	V129I	0.264	0.5	0.148
Pre-terminal	R0	G	9591	A	COX3	Non-synonymous	V129I	0.264	0.5	0.148
Modern terminal	U	G	9591	A	COX3	Non-synonymous	V129I	0.264	0.5	0.148
Modern terminal	U	G	9591	A	COX3	Non-synonymous	V129I	0.264	0.5	0.148
Modern terminal	U	G	9591	A	COX3	Non-synonymous	V129I	0.264	0.5	0.148
Modern terminal	U	G	9591	A	COX3	Non-synonymous	V129I	0.264	0.5	0.148
Modern terminal	U	G	9591	A	COX3	Non-synonymous	V129I	0.264	0.5	0.148
Modern terminal	U	G	9591	A	COX3	Non-synonymous	V129I	0.264	0.5	0.148
Pre-terminal	U	G	9591	A	COX3	Non-synonymous	V129I	0.264	0.5	0.148
Modern terminal	JT	G	9591	A	COX3	Non-synonymous	V129I	0.264	0.5	0.148
Modern terminal	JT	G	9591	A	COX3	Non-synonymous	V129I	0.264	0.5	0.148
Modern terminal	JT	G	9591	A	COX3	Non-synonymous	V129I	0.264	0.5	0.148
Pre-terminal	JT	G	9591	A	COX3	Non-synonymous	V129I	0.264	0.5	0.148
Pre-terminal	JT	G	9591	A	COX3	Non-synonymous	V129I	0.264	0.5	0.148
Pre-terminal	JT	G	9591	A	COX3	Non-synonymous	V129I	0.264	0.5	0.148
Modern terminal	N1	G	9591	A	COX3	Non-synonymous	V129I	0.264	0.5	0.148
Modern terminal	N1	G	9591	A	COX3	Non-synonymous	V129I	0.264	0.5	0.148
Modern terminal	N2	G	9591	A	COX3	Non-synonymous	V129I	0.264	0.5	0.148
Modern terminal	R0	T	9592	C	COX3	Non-synonymous	V129A	0.997	0	0.423
Modern terminal	R0	T	9592	C	COX3	Non-synonymous	V129A	0.997	0	0.423
Modern terminal	R0	T	9592	C	COX3	Non-synonymous	V129A	0.997	0	0.423
Pre-terminal	R0	T	9592	C	COX3	Non-synonymous	V129A	0.997	0	0.423
Pre-terminal	R0	T	9592	C	COX3	Non-synonymous	V129A	0.997	0	0.423
Modern terminal	JT	T	9592	C	COX3	Non-synonymous	V129A	0.997	0	0.423
Modern terminal	N1	T	9592	C	COX3	Non-synonymous	V129A	0.997	0	0.423
Modern terminal	R0	A	9604	G	COX3	Non-synonymous	N133S	0.989	0	0.278
Modern terminal	R0	A	9604	G	COX3	Non-synonymous	N133S	0.989	0	0.278
Modern terminal	U	A	9604	G	COX3	Non-synonymous	N133S	0.989	0	0.278
Pre-terminal	U	A	9604	G	COX3	Non-synonymous	N133S	0.989	0	0.278
Modern terminal	N1	A	9604	G	COX3	Non-synonymous	N133S	0.989	0	0.278
Pre-terminal	N1	A	9604	G	COX3	Non-synonymous	N133S	0.989	0	0.278
Modern terminal	R0	G	9612	C	COX3	Non-synonymous	V136L	0.376	0.08	0.364
Modern terminal	R0	G	9612	A	COX3	Non-synonymous	V136M	0.99	0	0.348
Modern terminal	R0	G	9612	A	COX3	Non-synonymous	V136M	0.99	0	0.348
Pre-terminal	R0	G	9612	A	COX3	Non-synonymous	V136M	0.99	0	0.348
Pre-terminal	R0	G	9612	A	COX3	Non-synonymous	V136M	0.99	0	0.348
Modern terminal	U	G	9612	A	COX3	Non-synonymous	V136M	0.99	0	0.348
Pre-terminal	U	G	9612	A	COX3	Non-synonymous	V136M	0.99	0	0.348
Pre-terminal	U	G	9612	A	COX3	Non-synonymous	V136M	0.99	0	0.348
Modern terminal	JT	G	9612	A	COX3	Non-synonymous	V136M	0.99	0	0.348
Modern terminal	JT	G	9612	A	COX3	Non-synonymous	V136M	0.99	0	0.348
Modern terminal	JT	G	9612	A	COX3	Non-synonymous	V136M	0.99	0	0.348

Modern terminal	JT	G	9612	A	COX3	Non-synonymous	V136M	0.99	0	0.348
Pre-terminal	JT	G	9612	A	COX3	Non-synonymous	V136M	0.99	0	0.348
Pre-terminal	JT	G	9612	C	COX3	Non-synonymous	V136L	0.376	0.08	0.364
Pre-terminal	N2	G	9612	A	COX3	Non-synonymous	V136M	0.99	0	0.348
Modern terminal	R0	G	9621	A	COX3	Non-synonymous	A139T	0.982	0.17	0.33
Pre-terminal	R0	G	9621	A	COX3	Non-synonymous	A139T	0.982	0.17	0.33
Pre-terminal	R0	G	9621	A	COX3	Non-synonymous	A139T	0.982	0.17	0.33
Modern terminal	JT	G	9621	A	COX3	Non-synonymous	A139T	0.982	0.17	0.33
Pre-terminal	JT	G	9621	A	COX3	Non-synonymous	A139T	0.982	0.17	0.33
Modern terminal	N1	G	9621	A	COX3	Non-synonymous	A139T	0.982	0.17	0.33
Modern terminal	R0	G	9630	A	COX3	Non-synonymous	V142M	0.272	0.01	0.398
Modern terminal	R0	G	9630	A	COX3	Non-synonymous	V142M	0.272	0.01	0.398
Modern terminal	R0	G	9630	A	COX3	Non-synonymous	V142M	0.272	0.01	0.398
Modern terminal	R0	G	9630	A	COX3	Non-synonymous	V142M	0.272	0.01	0.398
Modern terminal	R0	G	9630	A	COX3	Non-synonymous	V142M	0.272	0.01	0.398
Modern terminal	U	G	9630	C	COX3	Non-synonymous	V142L	0	0.06	0.422
Pre-terminal	U	G	9630	A	COX3	Non-synonymous	V142M	0.272	0.01	0.398
Modern terminal	JT	G	9630	A	COX3	Non-synonymous	V142M	0.272	0.01	0.398
Modern terminal	JT	G	9630	A	COX3	Non-synonymous	V142M	0.272	0.01	0.398
Modern terminal	X	G	9630	A	COX3	Non-synonymous	V142M	0.272	0.01	0.398
Pre-terminal	R0	T	9631	C	COX3	Non-synonymous	V142A	0.667	0.5	0.451
Pre-terminal	U	T	9633	G	COX3	Non-synonymous	S143A	0	0.29	0.266
Pre-terminal	JT	C	9651	A	COX3	Non-synonymous	H149N	0.922	0.03	0.46
Modern terminal	R0	G	9655	A	COX3	Non-synonymous	S150N	0.666	0	0.263
Modern terminal	R0	G	9655	A	COX3	Non-synonymous	S150N	0.666	0	0.263
Modern terminal	R0	G	9655	A	COX3	Non-synonymous	S150N	0.666	0	0.263
Pre-terminal	JT	G	9655	A	COX3	Non-synonymous	S150N	0.666	0	0.263
Modern terminal	R0	A	9660	G	COX3	Non-synonymous	M152V	0.98	0.12	0.336
Modern terminal	JT	A	9660	G	COX3	Non-synonymous	M152V	0.98	0.12	0.336
Modern terminal	JT	A	9660	T	COX3	Non-synonymous	M152L	0.051	0.46	0.42
Pre-terminal	R0	T	9661	C	COX3	Non-synonymous	M152T	0.94	0.1	0.332
Modern terminal	JT	G	9663	A	COX3	Non-synonymous	E153K	0.014	0.16	0.334
Modern terminal	U	A	9664	T	COX3	Non-synonymous	E153V	0.011	0.03	0.414
Pre-terminal	U	A	9664	G	COX3	Non-synonymous	E153G	0.27	0.12	0.351
Ancient terminal	R0	A	9667	G	COX3	Non-synonymous	N154S	0.087	0.05	0.136
Modern terminal	R0	A	9667	G	COX3	Non-synonymous	N154S	0.087	0.05	0.136
Modern terminal	U	A	9667	G	COX3	Non-synonymous	N154S	0.087	0.05	0.136
Modern terminal	U	A	9667	G	COX3	Non-synonymous	N154S	0.087	0.05	0.136
Pre-terminal	U	A	9667	G	COX3	Non-synonymous	N154S	0.087	0.05	0.136
Pre-terminal	JT	A	9667	G	COX3	Non-synonymous	N154S	0.087	0.05	0.136
Modern terminal	R0	A	9670	G	COX3	Non-synonymous	N155S	0	0.02	0.165
Modern terminal	U	A	9670	G	COX3	Non-synonymous	N155S	0	0.02	0.165
Modern terminal	U	A	9670	G	COX3	Non-synonymous	N155S	0	0.02	0.165
Pre-terminal	U	A	9670	G	COX3	Non-synonymous	N155S	0	0.02	0.165
Modern terminal	JT	A	9670	G	COX3	Non-synonymous	N155S	0	0.02	0.165
Pre-terminal	R0	A	9676	G	COX3	Non-synonymous	N157S	0.002	0.22	0.114
Modern terminal	X	A	9676	G	COX3	Non-synonymous	N157S	0.002	0.22	0.114
Modern terminal	R0	A	9679	G	COX3	Non-synonymous	Q158R	0.013	0.47	0.151
Pre-terminal	R0	T	9682	C	COX3	Non-synonymous	M159T	0	0.3	0.247
Modern terminal	U	T	9682	C	COX3	Non-synonymous	M159T	0	0.3	0.247

Modern terminal	U	T	9682	C	COX3	Non-synonymous	M159T	0	0.3	0.247
Pre-terminal	U	T	9682	C	COX3	Non-synonymous	M159T	0	0.3	0.247
Pre-terminal	U	T	9682	C	COX3	Non-synonymous	M159T	0	0.3	0.247
Modern terminal	U	T	9685	A	COX3	Non-synonymous	I160N	0.004	0.02	0.344
Pre-terminal	JT	T	9685	C	COX3	Non-synonymous	I160T	0.001	0.18	0.2
Ancient terminal	R0	C	9687	A	COX3	Non-synonymous	Q161K	0.59	0.27	0.351
Modern terminal	R0	A	9699	G	COX3	Non-synonymous	I165V	0.005	0.28	0.169
Modern terminal	JT	A	9699	G	COX3	Non-synonymous	I165V	0.005	0.28	0.169
Ancient terminal	JT	T	9709	C	COX3	Non-synonymous	L168S	1	0.34	0.274
Pre-terminal	R0	C	9717	A	COX3	Non-synonymous	L171I	0	0.69	0.113
Pre-terminal	U	C	9717	T	COX3	Non-synonymous	L171F	0.01	0.3	0.365
Modern terminal	U	C	9727	T	COX3	Non-synonymous	T174I	0.998	0	0.453
Modern terminal	N1	C	9727	T	COX3	Non-synonymous	T174I	0.998	0	0.453
Modern terminal	X	C	9727	T	COX3	Non-synonymous	T174I	0.998	0	0.453
Modern terminal	R0	C	9729	T	COX3	Non-synonymous	L175F	0.994	0.19	0.343
Modern terminal	JT	C	9729	T	COX3	Non-synonymous	L175F	0.994	0.19	0.343
Modern terminal	R0	G	9738	A	COX3	Non-synonymous	A178T	0.052	0.03	0.32
Modern terminal	R0	G	9738	A	COX3	Non-synonymous	A178T	0.052	0.03	0.32
Pre-terminal	R0	G	9738	A	COX3	Non-synonymous	A178T	0.052	0.03	0.32
Pre-terminal	U	G	9738	A	COX3	Non-synonymous	A178T	0.052	0.03	0.32
Pre-terminal	U	G	9738	A	COX3	Non-synonymous	A178T	0.052	0.03	0.32
Modern terminal	JT	G	9738	A	COX3	Non-synonymous	A178T	0.052	0.03	0.32
Modern terminal	R0	C	9739	T	COX3	Non-synonymous	A178V	0.004	0.05	0.387
Modern terminal	U	C	9739	T	COX3	Non-synonymous	A178V	0.004	0.05	0.387
Modern terminal	JT	C	9739	T	COX3	Non-synonymous	A178V	0.004	0.05	0.387
Pre-terminal	JT	C	9739	T	COX3	Non-synonymous	A178V	0.004	0.05	0.387
Modern terminal	R0	C	9742	T	COX3	Non-synonymous	S179L	0.991	0.65	0.548
Modern terminal	R0	C	9742	T	COX3	Non-synonymous	S179L	0.991	0.65	0.548
Modern terminal	JT	C	9742	T	COX3	Non-synonymous	S179L	0.991	0.65	0.548
Ancient terminal	X	C	9742	T	COX3	Non-synonymous	S179L	0.991	0.65	0.548
Modern terminal	JT	G	9744	A	COX3	Non-synonymous	E180K	0.946	0	0.599
Modern terminal	JT	G	9744	A	COX3	Non-synonymous	E180K	0.946	0	0.599
Modern terminal	JT	G	9744	A	COX3	Non-synonymous	E180K	0.946	0	0.599
Modern terminal	R0	T	9747	C	COX3	Non-synonymous	Y181H	1	0	0.548
Modern terminal	R0	T	9750	C	COX3	Non-synonymous	F182L	0.006	0.21	0.292
Pre-terminal	R0	T	9750	C	COX3	Non-synonymous	F182L	0.006	0.21	0.292
Modern terminal	R0	G	9753	A	COX3	Non-synonymous	E183K	0.946	0.18	0.59
Modern terminal	R0	G	9753	C	COX3	Non-synonymous	E183Q	0.968	0.26	0.453
Modern terminal	U	G	9753	A	COX3	Non-synonymous	E183K	0.946	0.18	0.59
Modern terminal	U	G	9753	A	COX3	Non-synonymous	E183K	0.946	0.18	0.59
Modern terminal	R0	A	9754	G	COX3	Non-synonymous	E183G	0.999	0.09	0.579
Pre-terminal	JT	A	9754	G	COX3	Non-synonymous	E183G	0.999	0.09	0.579
Modern terminal	R0	T	9756	G	COX3	Non-synonymous	S184A	0	1	0.077
Pre-terminal	R0	T	9756	G	COX3	Non-synonymous	S184A	0	1	0.077
Pre-terminal	N2	T	9756	G	COX3	Non-synonymous	S184A	0	1	0.077
Modern terminal	U	C	9759	T	COX3	Non-synonymous	P185S	0.998	0.44	0.179
Modern terminal	R0	A	9765	T	COX3	Non-synonymous	T187S	0.049	0.12	0.241
Pre-terminal	U	G	9777	A	COX3	Non-synonymous	G191S	1	0.57	0.593
Modern terminal	R0	A	9780	G	COX3	Non-synonymous	I192V	0.724	1	0.092
Modern terminal	JT	A	9780	G	COX3	Non-synonymous	I192V	0.724	1	0.092

Modern terminal	N1	A	9780	G	COX3	Non-synonymous	I192V	0.724	1	0.092
Modern terminal	R0	A	9792	G	COX3	Non-synonymous	T196A	0.988	0.07	0.654
Modern terminal	R0	G	9801	A	COX3	Non-synonymous	V199M	0.002	0.55	0.159
Modern terminal	R0	G	9801	A	COX3	Non-synonymous	V199M	0.002	0.55	0.159
Pre-terminal	R0	G	9801	A	COX3	Non-synonymous	V199M	0.002	0.55	0.159
Pre-terminal	R0	G	9801	A	COX3	Non-synonymous	V199M	0.002	0.55	0.159
Modern terminal	U	G	9801	A	COX3	Non-synonymous	V199M	0.002	0.55	0.159
Pre-terminal	U	G	9801	A	COX3	Non-synonymous	V199M	0.002	0.55	0.159
Pre-terminal	U	G	9801	A	COX3	Non-synonymous	V199M	0.002	0.55	0.159
Modern terminal	JT	G	9801	A	COX3	Non-synonymous	V199M	0.002	0.55	0.159
Modern terminal	N1	G	9801	A	COX3	Non-synonymous	V199M	0.002	0.55	0.159
Modern terminal	U	T	9802	C	COX3	Non-synonymous	V199A	0.388	0.1	0.53
Ancient terminal	R0	G	9804	T	COX3	Non-synonymous	A200S	0.008	0.08	0.521
Modern terminal	R0	G	9804	A	COX3	Non-synonymous	A200T	0.001	0.08	0.651
Modern terminal	R0	G	9804	A	COX3	Non-synonymous	A200T	0.001	0.08	0.651
Modern terminal	R0	G	9804	A	COX3	Non-synonymous	A200T	0.001	0.08	0.651
Modern terminal	R0	G	9804	A	COX3	Non-synonymous	A200T	0.001	0.08	0.651
Modern terminal	R0	G	9804	A	COX3	Non-synonymous	A200T	0.001	0.08	0.651
Modern terminal	R0	G	9804	A	COX3	Non-synonymous	A200T	0.001	0.08	0.651
Modern terminal	R0	G	9804	A	COX3	Non-synonymous	A200T	0.001	0.08	0.651
Modern terminal	R0	G	9804	A	COX3	Non-synonymous	A200T	0.001	0.08	0.651
Modern terminal	R0	G	9804	A	COX3	Non-synonymous	A200T	0.001	0.08	0.651
Modern terminal	R0	G	9804	A	COX3	Non-synonymous	A200T	0.001	0.08	0.651
Modern terminal	R0	G	9804	A	COX3	Non-synonymous	A200T	0.001	0.08	0.651
Modern terminal	R0	G	9804	A	COX3	Non-synonymous	A200T	0.001	0.08	0.651
Modern terminal	R0	G	9804	A	COX3	Non-synonymous	A200T	0.001	0.08	0.651
Modern terminal	R0	G	9804	A	COX3	Non-synonymous	A200T	0.001	0.08	0.651
Pre-terminal	R0	G	9804	A	COX3	Non-synonymous	A200T	0.001	0.08	0.651
Pre-terminal	R0	G	9804	A	COX3	Non-synonymous	A200T	0.001	0.08	0.651
Pre-terminal	R0	G	9804	A	COX3	Non-synonymous	A200T	0.001	0.08	0.651
Pre-terminal	R0	G	9804	A	COX3	Non-synonymous	A200T	0.001	0.08	0.651
Pre-terminal	R0	G	9804	A	COX3	Non-synonymous	A200T	0.001	0.08	0.651
Pre-terminal	R0	G	9804	A	COX3	Non-synonymous	A200T	0.001	0.08	0.651
Pre-terminal	R0	G	9804	A	COX3	Non-synonymous	A200T	0.001	0.08	0.651
Ancient terminal	U	G	9804	A	COX3	Non-synonymous	A200T	0.001	0.08	0.651
Ancient terminal	U	G	9804	A	COX3	Non-synonymous	A200T	0.001	0.08	0.651
Ancient terminal	U	G	9804	A	COX3	Non-synonymous	A200T	0.001	0.08	0.651
Ancient terminal	U	G	9804	A	COX3	Non-synonymous	A200T	0.001	0.08	0.651
Modern terminal	U	G	9804	A	COX3	Non-synonymous	A200T	0.001	0.08	0.651
Modern terminal	U	G	9804	A	COX3	Non-synonymous	A200T	0.001	0.08	0.651
Modern terminal	U	G	9804	A	COX3	Non-synonymous	A200T	0.001	0.08	0.651
Modern terminal	U	G	9804	A	COX3	Non-synonymous	A200T	0.001	0.08	0.651
Modern terminal	U	G	9804	A	COX3	Non-synonymous	A200T	0.001	0.08	0.651
Modern terminal	U	G	9804	A	COX3	Non-synonymous	A200T	0.001	0.08	0.651
Modern terminal	U	G	9804	A	COX3	Non-synonymous	A200T	0.001	0.08	0.651
Modern terminal	U	G	9804	A	COX3	Non-synonymous	A200T	0.001	0.08	0.651
Modern terminal	U	G	9804	A	COX3	Non-synonymous	A200T	0.001	0.08	0.651
Modern terminal	U	G	9804	A	COX3	Non-synonymous	A200T	0.001	0.08	0.651
Modern terminal	U	G	9804	C	COX3	Non-synonymous	A200P	0.787	0	0.831
Modern terminal	U	G	9804	T	COX3	Non-synonymous	A200S	0.008	0.08	0.521

Pre-terminal	U	G	9804	A	COX3	Non-synonymous	A200T	0.001	0.08	0.651
Pre-terminal	U	G	9804	A	COX3	Non-synonymous	A200T	0.001	0.08	0.651
Modern terminal	JT	G	9804	A	COX3	Non-synonymous	A200T	0.001	0.08	0.651
Modern terminal	JT	G	9804	A	COX3	Non-synonymous	A200T	0.001	0.08	0.651
Modern terminal	JT	G	9804	A	COX3	Non-synonymous	A200T	0.001	0.08	0.651
Modern terminal	JT	G	9804	A	COX3	Non-synonymous	A200T	0.001	0.08	0.651
Modern terminal	JT	G	9804	A	COX3	Non-synonymous	A200T	0.001	0.08	0.651
Pre-terminal	JT	G	9804	A	COX3	Non-synonymous	A200T	0.001	0.08	0.651
Pre-terminal	JT	G	9804	A	COX3	Non-synonymous	A200T	0.001	0.08	0.651
Pre-terminal	JT	G	9804	A	COX3	Non-synonymous	A200T	0.001	0.08	0.651
Modern terminal	N1	G	9804	A	COX3	Non-synonymous	A200T	0.001	0.08	0.651
Pre-terminal	N1	G	9804	A	COX3	Non-synonymous	A200T	0.001	0.08	0.651
Pre-terminal	N1	G	9804	A	COX3	Non-synonymous	A200T	0.001	0.08	0.651
Modern terminal	N2	G	9804	A	COX3	Non-synonymous	A200T	0.001	0.08	0.651
Modern terminal	U	G	9810	A	COX3	Non-synonymous	G202S	1	0	0.722
Modern terminal	N2	T	9813	C	COX3	Non-synonymous	F203L	0.998	0.15	0.653
Modern terminal	R0	C	9822	T	COX3	Non-synonymous	L206F	0.004	0.29	0.603
Modern terminal	R0	G	9828	A	COX3	Non-synonymous	V208I	0.637	0.05	0.336
Ancient terminal	U	G	9828	A	COX3	Non-synonymous	V208I	0.637	0.05	0.336
Modern terminal	JT	G	9828	A	COX3	Non-synonymous	V208I	0.637	0.05	0.336
Pre-terminal	R0	T	9829	C	COX3	Non-synonymous	V208A	0.999	0	0.574
Modern terminal	JT	T	9829	C	COX3	Non-synonymous	V208A	0.999	0	0.574
Modern terminal	R0	A	9831	G	COX3	Non-synonymous	I209V	0.808	0.01	0.211
Modern terminal	R0	A	9834	G	COX3	Non-synonymous	I210V	0.808	0.14	0.225
Ancient terminal	U	A	9834	T	COX3	Non-synonymous	I210F	0.975	0	0.568
Modern terminal	N1	T	9840	A	COX3	Non-synonymous	S212T	0.714	1	0.202
Pre-terminal	X	T	9840	A	COX3	Non-synonymous	S212T	0.714	1	0.202
Modern terminal	R0	A	9843	G	COX3	Non-synonymous	T213A	0.191	0.43	0.248
Ancient terminal	U	A	9843	G	COX3	Non-synonymous	T213A	0.191	0.43	0.248
Pre-terminal	JT	A	9843	G	COX3	Non-synonymous	T213A	0.191	0.43	0.248
Modern terminal	U	T	9846	C	COX3	Non-synonymous	F214L	0.997	0.05	0.429
Pre-terminal	R0	A	9852	G	COX3	Non-synonymous	T216A	0	0.64	0.095
Pre-terminal	U	A	9852	T	COX3	Non-synonymous	T216S	0	0.1	0.07
Pre-terminal	U	A	9852	G	COX3	Non-synonymous	T216A	0	0.64	0.095
Modern terminal	JT	A	9852	G	COX3	Non-synonymous	T216A	0	0.64	0.095
Modern terminal	R0	C	9853	T	COX3	Non-synonymous	T216I	0	1	0.076
Ancient terminal	JT	C	9853	T	COX3	Non-synonymous	T216I	0	1	0.076
Modern terminal	R0	A	9855	G	COX3	Non-synonymous	I217V	0	1	0.049
Modern terminal	U	A	9855	G	COX3	Non-synonymous	I217V	0	1	0.049
Modern terminal	JT	A	9855	G	COX3	Non-synonymous	I217V	0	1	0.049
Pre-terminal	R0	T	9856	C	COX3	Non-synonymous	I217T	0.003	0.05	0.242
Pre-terminal	JT	T	9856	C	COX3	Non-synonymous	I217T	0.003	0.05	0.242
Pre-terminal	R0	T	9861	C	COX3	Non-synonymous	F219L	0	1	0.127
Pre-terminal	R0	T	9861	C	COX3	Non-synonymous	F219L	0	1	0.127
Modern terminal	U	T	9861	C	COX3	Non-synonymous	F219L	0	1	0.127
Modern terminal	U	T	9861	C	COX3	Non-synonymous	F219L	0	1	0.127
Modern terminal	JT	T	9861	C	COX3	Non-synonymous	F219L	0	1	0.127
Modern terminal	JT	T	9861	C	COX3	Non-synonymous	F219L	0	1	0.127
Modern terminal	JT	T	9861	C	COX3	Non-synonymous	F219L	0	1	0.127
Pre-terminal	JT	T	9861	C	COX3	Non-synonymous	F219L	0	1	0.127

Modern terminal	N1	T	9861	C	COX3	Non-synonymous	F219L	0	1	0.127
Pre-terminal	N1	T	9861	C	COX3	Non-synonymous	F219L	0	1	0.127
Pre-terminal	N1	T	9861	C	COX3	Non-synonymous	F219L	0	1	0.127
Pre-terminal	R0	T	9862	A	COX3	Non-synonymous	F219Y	0.004	0.23	0.242
Modern terminal	R0	T	9865	C	COX3	Non-synonymous	I220T	0.001	0.07	0.209
Pre-terminal	R0	C	9866	G	COX3	Non-synonymous	I220M	0.542	0.09	0.129
Modern terminal	JT	A	9872	T	COX3	Non-synonymous	Q222H	0.979	0.03	0.228
Modern terminal	JT	T	9880	C	COX3	Non-synonymous	F225S	0.883	0.17	0.617
Modern terminal	N1	C	9882	T	COX3	Non-synonymous	H226Y	0.821	0.03	0.285
Modern terminal	R0	T	9891	C	COX3	Non-synonymous	S229P	0	0.1	0.474
Pre-terminal	U	T	9891	C	COX3	Non-synonymous	S229P	0	0.1	0.474
Modern terminal	R0	C	9902	A	COX3	Non-synonymous	H232Q	0.995	0	0.454
Pre-terminal	U	T	9903	C	COX3	Non-synonymous	F233L	0.998	0.32	0.488
Pre-terminal	X	T	9903	C	COX3	Non-synonymous	F233L	0.998	0.32	0.488
Ancient terminal	U	G	9907	A	COX3	Non-synonymous	G234D	1	0	0.726
Modern terminal	R0	T	9909	C	COX3	Non-synonymous	F235L	0.997	0	0.482
Modern terminal	R0	T	9909	C	COX3	Non-synonymous	F235L	0.997	0	0.482
Pre-terminal	R0	T	9909	C	COX3	Non-synonymous	F235L	0.997	0	0.482
Modern terminal	JT	T	9909	C	COX3	Non-synonymous	F235L	0.997	0	0.482
Modern terminal	JT	T	9909	C	COX3	Non-synonymous	F235L	0.997	0	0.482
Modern terminal	JT	T	9909	C	COX3	Non-synonymous	F235L	0.997	0	0.482
Pre-terminal	JT	T	9909	C	COX3	Non-synonymous	F235L	0.997	0	0.482
Modern terminal	N2	T	9909	C	COX3	Non-synonymous	F235L	0.997	0	0.482
Modern terminal	R0	C	9911	A	COX3	Non-synonymous	F235L	0.997	0	0.482
Modern terminal	JT	C	9911	A	COX3	Non-synonymous	F235L	0.997	0	0.482
Modern terminal	N2	C	9911	A	COX3	Non-synonymous	F235L	0.997	0	0.482
Modern terminal	R0	G	9912	A	COX3	Non-synonymous	E236K	0.988	0	0.548
Modern terminal	R0	G	9921	A	COX3	Non-synonymous	A239T	0.003	0.1	0.624
Modern terminal	R0	G	9921	A	COX3	Non-synonymous	A239T	0.003	0.1	0.624
Modern terminal	R0	G	9921	A	COX3	Non-synonymous	A239T	0.003	0.1	0.624
Pre-terminal	R0	G	9921	A	COX3	Non-synonymous	A239T	0.003	0.1	0.624
Pre-terminal	R0	G	9921	A	COX3	Non-synonymous	A239T	0.003	0.1	0.624
Modern terminal	U	G	9921	A	COX3	Non-synonymous	A239T	0.003	0.1	0.624
Modern terminal	U	G	9921	A	COX3	Non-synonymous	A239T	0.003	0.1	0.624
Modern terminal	U	G	9921	A	COX3	Non-synonymous	A239T	0.003	0.1	0.624
Pre-terminal	U	G	9921	A	COX3	Non-synonymous	A239T	0.003	0.1	0.624
Modern terminal	JT	G	9921	A	COX3	Non-synonymous	A239T	0.003	0.1	0.624
Modern terminal	JT	G	9921	A	COX3	Non-synonymous	A239T	0.003	0.1	0.624
Modern terminal	N1	G	9921	A	COX3	Non-synonymous	A239T	0.003	0.1	0.624
Modern terminal	N1	G	9921	A	COX3	Non-synonymous	A239T	0.003	0.1	0.624
Modern terminal	N1	G	9921	A	COX3	Non-synonymous	A239T	0.003	0.1	0.624
Modern terminal	R0	C	9922	T	COX3	Non-synonymous	A239V	0.481	0.04	0.635
Modern terminal	R0	C	9922	T	COX3	Non-synonymous	A239V	0.481	0.04	0.635
Modern terminal	R0	C	9922	T	COX3	Non-synonymous	A239V	0.481	0.04	0.635
Modern terminal	U	C	9922	T	COX3	Non-synonymous	A239V	0.481	0.04	0.635
Modern terminal	U	G	9945	A	COX3	Non-synonymous	V247M	0.997	0	0.463
Modern terminal	R0	G	9948	A	COX3	Non-synonymous	V248I	0	0.12	0.372
Modern terminal	R0	G	9948	A	COX3	Non-synonymous	V248I	0	0.12	0.372
Modern terminal	R0	G	9948	A	COX3	Non-synonymous	V248I	0	0.12	0.372
Modern terminal	R0	G	9948	A	COX3	Non-synonymous	V248I	0	0.12	0.372

Modern terminal	R0	G	9948	A	COX3	Non-synonymous	V248I	0	0.12	0.372
Pre-terminal	R0	G	9948	A	COX3	Non-synonymous	V248I	0	0.12	0.372
Pre-terminal	R0	G	9948	A	COX3	Non-synonymous	V248I	0	0.12	0.372
Pre-terminal	R0	G	9948	A	COX3	Non-synonymous	V248I	0	0.12	0.372
Ancient terminal	U	G	9948	A	COX3	Non-synonymous	V248I	0	0.12	0.372
Modern terminal	U	G	9948	A	COX3	Non-synonymous	V248I	0	0.12	0.372
Modern terminal	U	G	9948	A	COX3	Non-synonymous	V248I	0	0.12	0.372
Modern terminal	U	G	9948	A	COX3	Non-synonymous	V248I	0	0.12	0.372
Modern terminal	U	G	9948	A	COX3	Non-synonymous	V248I	0	0.12	0.372
Modern terminal	U	G	9948	A	COX3	Non-synonymous	V248I	0	0.12	0.372
Pre-terminal	U	G	9948	A	COX3	Non-synonymous	V248I	0	0.12	0.372
Pre-terminal	U	G	9948	A	COX3	Non-synonymous	V248I	0	0.12	0.372
Pre-terminal	U	G	9948	A	COX3	Non-synonymous	V248I	0	0.12	0.372
Modern terminal	JT	G	9948	A	COX3	Non-synonymous	V248I	0	0.12	0.372
Modern terminal	JT	G	9948	A	COX3	Non-synonymous	V248I	0	0.12	0.372
Pre-terminal	JT	G	9948	A	COX3	Non-synonymous	V248I	0	0.12	0.372
Pre-terminal	JT	G	9948	A	COX3	Non-synonymous	V248I	0	0.12	0.372
Pre-terminal	JT	G	9948	A	COX3	Non-synonymous	V248I	0	0.12	0.372
Pre-terminal	X	G	9948	A	COX3	Non-synonymous	V248I	0	0.12	0.372
Modern terminal	N2	G	9948	A	COX3	Non-synonymous	V248I	0	0.12	0.372
Modern terminal	R0	T	9951	C	COX3	Non-synonymous	W249R	1	0.06	0.877
Modern terminal	U	T	9951	C	COX3	Non-synonymous	W249R	1	0.06	0.877
Modern terminal	U	T	9951	C	COX3	Non-synonymous	W249R	1	0.06	0.877
Modern terminal	U	T	9951	C	COX3	Non-synonymous	W249R	1	0.06	0.877
Modern terminal	U	T	9951	C	COX3	Non-synonymous	W249R	1	0.06	0.877
Modern terminal	R0	T	9957	C	COX3	Non-synonymous	F251L	0.001	0.01	0.585
Modern terminal	U	T	9957	C	COX3	Non-synonymous	F251L	0.001	0.01	0.585
Modern terminal	JT	T	9957	C	COX3	Non-synonymous	F251L	0.001	0.01	0.585
Pre-terminal	N1	T	9957	C	COX3	Non-synonymous	F251L	0.001	0.01	0.585
Ancient terminal	R0	T	9963	C	COX3	Non-synonymous	Y253H	1	0	0.606
Modern terminal	R0	T	9963	C	COX3	Non-synonymous	Y253H	1	0	0.606
Modern terminal	R0	G	9966	A	COX3	Non-synonymous	V254I	0	0.51	0.224
Modern terminal	R0	G	9966	A	COX3	Non-synonymous	V254I	0	0.51	0.224
Modern terminal	R0	G	9966	A	COX3	Non-synonymous	V254I	0	0.51	0.224
Modern terminal	R0	G	9966	A	COX3	Non-synonymous	V254I	0	0.51	0.224
Modern terminal	R0	G	9966	A	COX3	Non-synonymous	V254I	0	0.51	0.224
Modern terminal	R0	G	9966	A	COX3	Non-synonymous	V254I	0	0.51	0.224
Modern terminal	R0	G	9966	A	COX3	Non-synonymous	V254I	0	0.51	0.224
Modern terminal	R0	G	9966	A	COX3	Non-synonymous	V254I	0	0.51	0.224
Modern terminal	R0	G	9966	A	COX3	Non-synonymous	V254I	0	0.51	0.224
Modern terminal	R0	G	9966	A	COX3	Non-synonymous	V254I	0	0.51	0.224
Modern terminal	R0	G	9966	A	COX3	Non-synonymous	V254I	0	0.51	0.224
Pre-terminal	R0	G	9966	A	COX3	Non-synonymous	V254I	0	0.51	0.224
Pre-terminal	R0	G	9966	A	COX3	Non-synonymous	V254I	0	0.51	0.224
Pre-terminal	R0	G	9966	A	COX3	Non-synonymous	V254I	0	0.51	0.224
Pre-terminal	R0	G	9966	A	COX3	Non-synonymous	V254I	0	0.51	0.224
Ancient terminal	U	G	9966	A	COX3	Non-synonymous	V254I	0	0.51	0.224
Ancient terminal	U	G	9966	A	COX3	Non-synonymous	V254I	0	0.51	0.224
Modern terminal	U	G	9966	A	COX3	Non-synonymous	V254I	0	0.51	0.224
Modern terminal	U	G	9966	A	COX3	Non-synonymous	V254I	0	0.51	0.224

Modern terminal	U	G	9966	A	COX3	Non-synonymous	V254I	0	0.51	0.224
Modern terminal	U	G	9966	A	COX3	Non-synonymous	V254I	0	0.51	0.224
Modern terminal	U	G	9966	A	COX3	Non-synonymous	V254I	0	0.51	0.224
Modern terminal	U	G	9966	A	COX3	Non-synonymous	V254I	0	0.51	0.224
Modern terminal	U	G	9966	A	COX3	Non-synonymous	V254I	0	0.51	0.224
Pre-terminal	U	G	9966	A	COX3	Non-synonymous	V254I	0	0.51	0.224
Pre-terminal	U	G	9966	A	COX3	Non-synonymous	V254I	0	0.51	0.224
Modern terminal	JT	G	9966	A	COX3	Non-synonymous	V254I	0	0.51	0.224
Modern terminal	N1	G	9966	A	COX3	Non-synonymous	V254I	0	0.51	0.224
Modern terminal	N1	G	9966	A	COX3	Non-synonymous	V254I	0	0.51	0.224
Pre-terminal	N1	G	9966	A	COX3	Non-synonymous	V254I	0	0.51	0.224
Modern terminal	N2	G	9966	A	COX3	Non-synonymous	V254I	0	0.51	0.224
Ancient terminal	R0	A	9972	T	COX3	Non-synonymous	I256F	0.246	0	0.666
Modern terminal	U	A	9972	C	COX3	Non-synonymous	I256L	0.001	0	0.471
Modern terminal	U	A	9972	G	COX3	Non-synonymous	I256V	0	0.13	0.256
Modern terminal	N2	A	9972	G	COX3	Non-synonymous	I256V	0	0.13	0.256
Modern terminal	JT	T	9973	C	COX3	Non-synonymous	I256T	0.81	0	0.45
Modern terminal	U	T	9981	G	COX3	Non-synonymous	W259G	1	0	0.861
Modern terminal	JT	G	9985	A	COX3	Non-synonymous	G260E	1	0	0.634
Modern terminal	R0	T	9987	C	COX3	Non-synonymous	S261P	0.944	0.01	0.579
Modern terminal	JT	C	9988	A	COX3	Non-synonymous	S261Y	0.996	0	0.479
Ancient terminal	R0	A	10063	G	ND3	Non-synonymous	N2S	0.977	0.04	0.487
Modern terminal	R0	T	10065	C	ND3	Non-synonymous	F3L	0.993	1	0.142
Modern terminal	N1	C	10069	T	ND3	Non-synonymous	A4V	0	0.39	0.159
Ancient terminal	N1	T	10072	A	ND3	Nonsense				
Modern terminal	JT	A	10073	T	ND3	Non-synonymous	L5F	0.988	0.02	0.345
Ancient terminal	JT	A	10074	C	ND3	Non-synonymous	I6L	0.001	0.4	0.161
Ancient terminal	U	A	10079	C	ND3	Non-synonymous	L7F	0.988	0.02	0.386
Pre-terminal	U	A	10080	G	ND3	Non-synonymous	M8V	0.152	0.27	0.156
Ancient terminal	U	T	10081	C	ND3	Non-synonymous	M8T	0	0.28	0.116
Ancient terminal	U	A	10082	T	ND3	Non-synonymous	M8I	0	0.56	0.134
Ancient terminal	U	A	10082	T	ND3	Non-synonymous	M8I	0	0.56	0.134
Modern terminal	JT	A	10083	G	ND3	Non-synonymous	I9V	0	0.59	0.108
Modern terminal	N1	A	10083	G	ND3	Non-synonymous	I9V	0	0.59	0.108
Modern terminal	R0	T	10084	C	ND3	Non-synonymous	I9T	0	0.39	0.106
Modern terminal	R0	T	10084	C	ND3	Non-synonymous	I9T	0	0.39	0.106
Modern terminal	R0	T	10084	C	ND3	Non-synonymous	I9T	0	0.39	0.106
Modern terminal	R0	T	10084	C	ND3	Non-synonymous	I9T	0	0.39	0.106
Pre-terminal	R0	T	10084	C	ND3	Non-synonymous	I9T	0	0.39	0.106
Pre-terminal	R0	T	10084	C	ND3	Non-synonymous	I9T	0	0.39	0.106
Pre-terminal	R0	T	10084	C	ND3	Non-synonymous	I9T	0	0.39	0.106
Pre-terminal	R0	T	10084	C	ND3	Non-synonymous	I9T	0	0.39	0.106
Ancient terminal	U	T	10084	C	ND3	Non-synonymous	I9T	0	0.39	0.106
Ancient terminal	U	T	10084	C	ND3	Non-synonymous	I9T	0	0.39	0.106
Modern terminal	U	T	10084	C	ND3	Non-synonymous	I9T	0	0.39	0.106
Modern terminal	U	T	10084	C	ND3	Non-synonymous	I9T	0	0.39	0.106
Modern terminal	U	T	10084	C	ND3	Non-synonymous	I9T	0	0.39	0.106
Modern terminal	U	T	10084	C	ND3	Non-synonymous	I9T	0	0.39	0.106
Modern terminal	U	T	10084	C	ND3	Non-synonymous	I9T	0	0.39	0.106
Pre-terminal	U	T	10084	C	ND3	Non-synonymous	I9T	0	0.39	0.106

Pre-terminal	U	T	10084	C	ND3	Non-synonymous	I9T	0	0.39	0.106
Pre-terminal	U	T	10084	C	ND3	Non-synonymous	I9T	0	0.39	0.106
Ancient terminal	JT	T	10084	C	ND3	Non-synonymous	I9T	0	0.39	0.106
Modern terminal	JT	T	10084	C	ND3	Non-synonymous	I9T	0	0.39	0.106
Pre-terminal	JT	T	10084	C	ND3	Non-synonymous	I9T	0	0.39	0.106
Pre-terminal	JT	T	10084	C	ND3	Non-synonymous	I9T	0	0.39	0.106
Modern terminal	N1	T	10084	C	ND3	Non-synonymous	I9T	0	0.39	0.106
Modern terminal	X	T	10084	C	ND3	Non-synonymous	I9T	0	0.39	0.106
Modern terminal	X	T	10084	C	ND3	Non-synonymous	I9T	0	0.39	0.106
Pre-terminal	X	T	10084	C	ND3	Non-synonymous	I9T	0	0.39	0.106
Pre-terminal	X	T	10084	C	ND3	Non-synonymous	I9T	0	0.39	0.106
Modern terminal	N2	T	10084	C	ND3	Non-synonymous	I9T	0	0.39	0.106
Modern terminal	U	A	10086	G	ND3	Non-synonymous	N10D	0.977	0.07	0.311
Modern terminal	JT	A	10086	G	ND3	Non-synonymous	N10D	0.977	0.07	0.311
Pre-terminal	N2	A	10086	G	ND3	Non-synonymous	N10D	0.977	0.07	0.311
Ancient terminal	U	C	10088	G	ND3	Non-synonymous	N10K	0.989	0.02	0.425
Modern terminal	U	C	10090	A	ND3	Non-synonymous	T11N	0.99	0.09	0.194
Ancient terminal	R0	C	10092	G	ND3	Non-synonymous	L12V	0.871	0.87	0.169
Ancient terminal	JT	T	10093	C	ND3	Non-synonymous	L12P	1	0.09	0.481
Modern terminal	R0	C	10095	A	ND3	Non-synonymous	L13M	0.999	0	0.252
Ancient terminal	R0	G	10098	T	ND3	Non-synonymous	A14S	0.719	1	0.101
Modern terminal	N1	G	10098	A	ND3	Non-synonymous	A14T	0.982	0.11	0.144
Modern terminal	X	G	10098	T	ND3	Non-synonymous	A14S	0.719	1	0.101
Ancient terminal	R0	C	10099	A	ND3	Non-synonymous	A14D	0.997	0.01	0.504
Ancient terminal	JT	T	10108	C	ND3	Non-synonymous	L17P	1	0	0.9
Ancient terminal	U	A	10110	T	ND3	Non-synonymous	M18L	0.051	0.33	0.365
Ancient terminal	R0	T	10111	A	ND3	Non-synonymous	M18K	0.405	0.01	0.608
Pre-terminal	U	T	10111	C	ND3	Non-synonymous	M18T	0.94	0.12	0.355
Pre-terminal	R0	A	10113	G	ND3	Non-synonymous	I19V	0.007	0.47	0.112
Modern terminal	U	A	10113	G	ND3	Non-synonymous	I19V	0.007	0.47	0.112
Modern terminal	U	T	10114	C	ND3	Non-synonymous	I19T	0.008	0.4	0.116
Modern terminal	U	T	10114	C	ND3	Non-synonymous	I19T	0.008	0.4	0.116
Pre-terminal	U	T	10114	C	ND3	Non-synonymous	I19T	0.008	0.4	0.116
Ancient terminal	R0	A	10116	G	ND3	Non-synonymous	I20V	0.007	0.49	0.162
Modern terminal	R0	G	10143	A	ND3	Non-synonymous	G29S	0	0.57	0.201
Modern terminal	R0	G	10143	A	ND3	Non-synonymous	G29S	0	0.57	0.201
Modern terminal	R0	G	10143	A	ND3	Non-synonymous	G29S	0	0.57	0.201
Modern terminal	R0	G	10143	A	ND3	Non-synonymous	G29S	0	0.57	0.201
Pre-terminal	R0	G	10143	A	ND3	Non-synonymous	G29S	0	0.57	0.201
Ancient terminal	U	G	10143	A	ND3	Non-synonymous	G29S	0	0.57	0.201
Ancient terminal	U	G	10143	A	ND3	Non-synonymous	G29S	0	0.57	0.201
Modern terminal	U	G	10143	A	ND3	Non-synonymous	G29S	0	0.57	0.201
Modern terminal	U	G	10143	A	ND3	Non-synonymous	G29S	0	0.57	0.201
Modern terminal	U	G	10143	A	ND3	Non-synonymous	G29S	0	0.57	0.201
Modern terminal	U	G	10143	A	ND3	Non-synonymous	G29S	0	0.57	0.201
Pre-terminal	U	G	10143	A	ND3	Non-synonymous	G29S	0	0.57	0.201
Modern terminal	JT	G	10143	A	ND3	Non-synonymous	G29S	0	0.57	0.201
Pre-terminal	JT	G	10143	A	ND3	Non-synonymous	G29S	0	0.57	0.201
Pre-terminal	JT	G	10143	A	ND3	Non-synonymous	G29S	0	0.57	0.201
Modern terminal	X	G	10143	A	ND3	Non-synonymous	G29S	0	0.57	0.201

Modern terminal	N2	G	10143	A	ND3	Non-synonymous	G29S	0	0.57	0.201
Modern terminal	R0	C	10159	A	ND3	Non-synonymous	S34Y	0.401	0.64	0.42
Pre-terminal	U	C	10159	A	ND3	Non-synonymous	S34Y	0.401	0.64	0.42
Modern terminal	N2	C	10159	A	ND3	Non-synonymous	S34Y	0.401	0.64	0.42
Ancient terminal	R0	A	10161	T	ND3	Non-synonymous	T35S	0	1	0.079
Ancient terminal	N1	C	10164	A	ND3	Non-synonymous	P36T	1	0	0.574
Ancient terminal	R0	T	10179	G	ND3	Non-synonymous	F41V	0.998	0	0.65
Pre-terminal	R0	A	10188	G	ND3	Non-synonymous	M44V	0.001	0.05	0.477
Pre-terminal	R0	A	10188	G	ND3	Non-synonymous	M44V	0.001	0.05	0.477
Pre-terminal	R0	A	10188	G	ND3	Non-synonymous	M44V	0.001	0.05	0.477
Ancient terminal	U	A	10188	G	ND3	Non-synonymous	M44V	0.001	0.05	0.477
Modern terminal	JT	A	10188	G	ND3	Non-synonymous	M44V	0.001	0.05	0.477
Modern terminal	N1	A	10188	G	ND3	Non-synonymous	M44V	0.001	0.05	0.477
Ancient terminal	U	T	10189	C	ND3	Non-synonymous	M44T	0.006	0.05	0.45
Modern terminal	U	T	10189	C	ND3	Non-synonymous	M44T	0.006	0.05	0.45
Ancient terminal	R0	C	10192	A	ND3	Non-synonymous	S45Y	0	0.04	0.417
Modern terminal	R0	C	10192	T	ND3	Non-synonymous	S45F	0	0	0.431
Modern terminal	R0	C	10192	T	ND3	Non-synonymous	S45F	0	0	0.431
Modern terminal	R0	C	10192	A	ND3	Non-synonymous	S45Y	0	0.04	0.417
Pre-terminal	R0	C	10192	T	ND3	Non-synonymous	S45F	0	0	0.431
Pre-terminal	R0	C	10192	T	ND3	Non-synonymous	S45F	0	0	0.431
Pre-terminal	R0	C	10192	T	ND3	Non-synonymous	S45F	0	0	0.431
Modern terminal	U	C	10192	T	ND3	Non-synonymous	S45F	0	0	0.431
Modern terminal	JT	C	10192	T	ND3	Non-synonymous	S45F	0	0	0.431
Pre-terminal	JT	C	10192	T	ND3	Non-synonymous	S45F	0	0	0.431
Modern terminal	X	C	10192	A	ND3	Non-synonymous	S45Y	0	0.04	0.417
Pre-terminal	N2	C	10194	T	ND3	Non-synonymous	P46S	0.337	1	0.066
Modern terminal	R0	G	10197	A	ND3	Non-synonymous	A47T	0.996	0	0.555
Modern terminal	R0	G	10197	A	ND3	Non-synonymous	A47T	0.996	0	0.555
Modern terminal	R0	G	10203	A	ND3	Non-synonymous	V49I	0	0.18	0.083
Modern terminal	R0	G	10203	A	ND3	Non-synonymous	V49I	0	0.18	0.083
Modern terminal	R0	G	10203	A	ND3	Non-synonymous	V49I	0	0.18	0.083
Modern terminal	U	G	10203	A	ND3	Non-synonymous	V49I	0	0.18	0.083
Pre-terminal	U	G	10203	A	ND3	Non-synonymous	V49I	0	0.18	0.083
Pre-terminal	U	G	10203	A	ND3	Non-synonymous	V49I	0	0.18	0.083
Modern terminal	JT	G	10203	A	ND3	Non-synonymous	V49I	0	0.18	0.083
Pre-terminal	JT	G	10203	A	ND3	Non-synonymous	V49I	0	0.18	0.083
Pre-terminal	JT	G	10203	A	ND3	Non-synonymous	V49I	0	0.18	0.083
Pre-terminal	N1	G	10203	A	ND3	Non-synonymous	V49I	0	0.18	0.083
Modern terminal	X	G	10203	A	ND3	Non-synonymous	V49I	0	0.18	0.083
Ancient terminal	R0	T	10209	C	ND3	Non-synonymous	F51L	0.999	0.03	0.677
Ancient terminal	R0	T	10212	A	ND3	Non-synonymous	S52T	0.91	0.02	0.519
Ancient terminal	R0	T	10212	C	ND3	Non-synonymous	S52P	0.999	0	0.754
Ancient terminal	R0	T	10231	C	ND3	Non-synonymous	V58A	0.615	0	0.696
Ancient terminal	JT	C	10234	G	ND3	Non-synonymous	A59G	1	0.18	0.685
Modern terminal	U	A	10236	G	ND3	Non-synonymous	I60V	0.866	0.02	0.44
Modern terminal	U	A	10236	G	ND3	Non-synonymous	I60V	0.866	0.02	0.44
Ancient terminal	N1	A	10236	G	ND3	Non-synonymous	I60V	0.866	0.02	0.44
Ancient terminal	R0	T	10237	C	ND3	Non-synonymous	I60T	1	0	0.711
Ancient terminal	R0	T	10237	C	ND3	Non-synonymous	I60T	1	0	0.711

Modern terminal	R0	T	10237	C	ND3	Non-synonymous	I60T	1	0	0.711
Modern terminal	R0	T	10237	C	ND3	Non-synonymous	I60T	1	0	0.711
Modern terminal	R0	T	10237	C	ND3	Non-synonymous	I60T	1	0	0.711
Pre-terminal	R0	T	10237	C	ND3	Non-synonymous	I60T	1	0	0.711
Pre-terminal	R0	T	10237	C	ND3	Non-synonymous	I60T	1	0	0.711
Pre-terminal	R0	T	10237	C	ND3	Non-synonymous	I60T	1	0	0.711
Pre-terminal	R0	T	10237	C	ND3	Non-synonymous	I60T	1	0	0.711
Pre-terminal	R0	T	10237	C	ND3	Non-synonymous	I60T	1	0	0.711
Pre-terminal	R0	T	10237	C	ND3	Non-synonymous	I60T	1	0	0.711
Pre-terminal	R0	T	10237	C	ND3	Non-synonymous	I60T	1	0	0.711
Ancient terminal	U	T	10237	C	ND3	Non-synonymous	I60T	1	0	0.711
Modern terminal	U	T	10237	C	ND3	Non-synonymous	I60T	1	0	0.711
Modern terminal	U	T	10237	C	ND3	Non-synonymous	I60T	1	0	0.711
Modern terminal	U	T	10237	C	ND3	Non-synonymous	I60T	1	0	0.711
Modern terminal	U	T	10237	C	ND3	Non-synonymous	I60T	1	0	0.711
Modern terminal	U	T	10237	C	ND3	Non-synonymous	I60T	1	0	0.711
Pre-terminal	U	T	10237	C	ND3	Non-synonymous	I60T	1	0	0.711
Modern terminal	JT	T	10237	C	ND3	Non-synonymous	I60T	1	0	0.711
Modern terminal	JT	T	10237	C	ND3	Non-synonymous	I60T	1	0	0.711
Modern terminal	JT	T	10237	C	ND3	Non-synonymous	I60T	1	0	0.711
Pre-terminal	JT	T	10237	C	ND3	Non-synonymous	I60T	1	0	0.711
Pre-terminal	JT	T	10237	C	ND3	Non-synonymous	I60T	1	0	0.711
Ancient terminal	X	T	10237	C	ND3	Non-synonymous	I60T	1	0	0.711
Modern terminal	U	C	10240	T	ND3	Non-synonymous	T61I	1	0.35	0.836
Ancient terminal	JT	A	10255	G	ND3	Non-synonymous	D66G	1	0	0.862
Modern terminal	U	C	10257	A	ND3	Non-synonymous	L67M	1	0	0.5
Modern terminal	U	T	10265	G	ND3	Non-synonymous	I69M	1	0	0.634
Ancient terminal	R0	T	10276	A	ND3	Nonsense				
Ancient terminal	R0	A	10277	T	ND3	Non-synonymous	L73F	0.999	0.12	0.684
Ancient terminal	U	G	10290	A	ND3	Non-synonymous	A78T	0.977	0	0.536
Modern terminal	U	T	10294	C	ND3	Non-synonymous	L79P	1	0.09	0.595
Modern terminal	R0	C	10300	A	ND3	Non-synonymous	T81K	0.992	0.28	0.497
Modern terminal	R0	C	10303	T	ND3	Non-synonymous	T82I	0.575	0.41	0.24
Modern terminal	R0	A	10306	G	ND3	Non-synonymous	N83S	0.306	0.22	0.282
Pre-terminal	R0	A	10306	G	ND3	Non-synonymous	N83S	0.306	0.22	0.282
Ancient terminal	U	T	10315	C	ND3	Non-synonymous	L86P	1	0.2	0.563
Ancient terminal	R0	G	10320	A	ND3	Non-synonymous	V88I	0	0.49	0.075
Modern terminal	R0	G	10320	A	ND3	Non-synonymous	V88I	0	0.49	0.075
Modern terminal	R0	G	10320	A	ND3	Non-synonymous	V88I	0	0.49	0.075
Modern terminal	U	G	10320	A	ND3	Non-synonymous	V88I	0	0.49	0.075
Modern terminal	U	G	10320	A	ND3	Non-synonymous	V88I	0	0.49	0.075
Pre-terminal	U	G	10320	A	ND3	Non-synonymous	V88I	0	0.49	0.075
Pre-terminal	U	G	10320	A	ND3	Non-synonymous	V88I	0	0.49	0.075
Modern terminal	JT	G	10320	A	ND3	Non-synonymous	V88I	0	0.49	0.075
Ancient terminal	N2	G	10320	T	ND3	Non-synonymous	V88F	0	0.15	0.365
Modern terminal	R0	T	10321	C	ND3	Non-synonymous	V88A	0.007	0.04	0.208
Modern terminal	R0	T	10321	C	ND3	Non-synonymous	V88A	0.007	0.04	0.208
Modern terminal	R0	T	10321	C	ND3	Non-synonymous	V88A	0.007	0.04	0.208
Modern terminal	R0	T	10321	C	ND3	Non-synonymous	V88A	0.007	0.04	0.208
Modern terminal	JT	T	10321	C	ND3	Non-synonymous	V88A	0.007	0.04	0.208

Modern terminal	JT	T	10321	C	ND3	Non-synonymous	V88A	0.007	0.04	0.208
Modern terminal	JT	T	10321	C	ND3	Non-synonymous	V88A	0.007	0.04	0.208
Modern terminal	JT	T	10321	C	ND3	Non-synonymous	V88A	0.007	0.04	0.208
Modern terminal	JT	T	10321	C	ND3	Non-synonymous	V88A	0.007	0.04	0.208
Pre-terminal	JT	T	10321	C	ND3	Non-synonymous	V88A	0.007	0.04	0.208
Pre-terminal	JT	T	10321	C	ND3	Non-synonymous	V88A	0.007	0.04	0.208
Pre-terminal	R0	A	10323	G	ND3	Non-synonymous	M89V	0.334	0.24	0.109
Ancient terminal	U	A	10323	G	ND3	Non-synonymous	M89V	0.334	0.24	0.109
Ancient terminal	U	T	10324	C	ND3	Non-synonymous	M89T	0	0.64	0.068
Pre-terminal	U	T	10324	C	ND3	Non-synonymous	M89T	0	0.64	0.068
Pre-terminal	U	T	10326	A	ND3	Non-synonymous	S90T	0.036	0.39	0.16
Ancient terminal	JT	C	10330	A	ND3	Non-synonymous	S91Y	1	0	0.414
Pre-terminal	R0	T	10336	C	ND3	Non-synonymous	L93S	0.973	0.12	0.352
Modern terminal	U	T	10336	C	ND3	Non-synonymous	L93S	0.973	0.12	0.352
Modern terminal	U	T	10336	C	ND3	Non-synonymous	L93S	0.973	0.12	0.352
Pre-terminal	U	T	10336	C	ND3	Non-synonymous	L93S	0.973	0.12	0.352
Modern terminal	R0	A	10344	G	ND3	Non-synonymous	I96V	0.002	1	0.1
Modern terminal	R0	A	10344	G	ND3	Non-synonymous	I96V	0.002	1	0.1
Modern terminal	JT	T	10345	C	ND3	Non-synonymous	I96T	0.016	0.31	0.132
Pre-terminal	JT	T	10345	C	ND3	Non-synonymous	I96T	0.016	0.31	0.132
Pre-terminal	JT	C	10346	G	ND3	Non-synonymous	I96M	0.973	0.15	0.192
Pre-terminal	R0	T	10348	C	ND3	Non-synonymous	I97T	1	0	0.463
Ancient terminal	R0	T	10351	C	ND3	Non-synonymous	L98P	1	0	0.901
Pre-terminal	JT	G	10360	A	ND3	Non-synonymous	S101N	0.389	0	0.373
Modern terminal	R0	G	10365	A	ND3	Non-synonymous	A103T	0.002	0.28	0.406
Modern terminal	R0	G	10365	A	ND3	Non-synonymous	A103T	0.002	0.28	0.406
Modern terminal	R0	G	10365	A	ND3	Non-synonymous	A103T	0.002	0.28	0.406
Pre-terminal	R0	G	10365	A	ND3	Non-synonymous	A103T	0.002	0.28	0.406
Pre-terminal	R0	G	10365	A	ND3	Non-synonymous	A103T	0.002	0.28	0.406
Pre-terminal	R0	G	10365	A	ND3	Non-synonymous	A103T	0.002	0.28	0.406
Modern terminal	U	G	10365	A	ND3	Non-synonymous	A103T	0.002	0.28	0.406
Ancient terminal	JT	G	10365	A	ND3	Non-synonymous	A103T	0.002	0.28	0.406
Ancient terminal	U	T	10370	A	ND3	Nonsense				
Modern terminal	U	G	10371	A	ND3	Non-synonymous	E105K	1	0	0.569
Modern terminal	R0	A	10383	T	ND3	Nonsense				
Ancient terminal	JT	G	10387	T	ND3	Non-synonymous	G110V	1	0	0.77
Ancient terminal	JT	T	10390	C	ND3	Non-synonymous	L111S	1	0	0.548
Ancient terminal	JT	A	10393	C	ND3	Non-synonymous	D112A	0.844	0	0.315
Ancient terminal	R0	A	10398	G	ND3	Non-synonymous	T114A	0	0.66	0.203
Modern terminal	R0	A	10398	G	ND3	Non-synonymous	T114A	0	0.66	0.203
Modern terminal	R0	A	10398	G	ND3	Non-synonymous	T114A	0	0.66	0.203
Modern terminal	R0	A	10398	G	ND3	Non-synonymous	T114A	0	0.66	0.203
Modern terminal	R0	A	10398	G	ND3	Non-synonymous	T114A	0	0.66	0.203
Modern terminal	R0	A	10398	G	ND3	Non-synonymous	T114A	0	0.66	0.203
Modern terminal	R0	A	10398	T	ND3	Non-synonymous	T114S	0.006	0.14	0.159
Modern terminal	R0	A	10398	G	ND3	Non-synonymous	T114A	0	0.66	0.203
Modern terminal	R0	A	10398	G	ND3	Non-synonymous	T114A	0	0.66	0.203
Pre-terminal	R0	A	10398	G	ND3	Non-synonymous	T114A	0	0.66	0.203
Pre-terminal	R0	A	10398	G	ND3	Non-synonymous	T114A	0	0.66	0.203
Pre-terminal	R0	A	10398	G	ND3	Non-synonymous	T114A	0	0.66	0.203

Ancient terminal	U	A	10398	G	ND3	Non-synonymous	T114A	0	0.66	0.203
Ancient terminal	U	A	10398	G	ND3	Non-synonymous	T114A	0	0.66	0.203
Ancient terminal	U	A	10398	G	ND3	Non-synonymous	T114A	0	0.66	0.203
Modern terminal	U	A	10398	G	ND3	Non-synonymous	T114A	0	0.66	0.203
Modern terminal	U	A	10398	G	ND3	Non-synonymous	T114A	0	0.66	0.203
Modern terminal	U	A	10398	G	ND3	Non-synonymous	T114A	0	0.66	0.203
Modern terminal	U	A	10398	G	ND3	Non-synonymous	T114A	0	0.66	0.203
Modern terminal	U	A	10398	G	ND3	Non-synonymous	T114A	0	0.66	0.203
Modern terminal	U	A	10398	G	ND3	Non-synonymous	T114A	0	0.66	0.203
Modern terminal	U	A	10398	G	ND3	Non-synonymous	T114A	0	0.66	0.203
Modern terminal	U	A	10398	G	ND3	Non-synonymous	T114A	0	0.66	0.203
Modern terminal	U	A	10398	G	ND3	Non-synonymous	T114A	0	0.66	0.203
Modern terminal	U	A	10398	G	ND3	Non-synonymous	T114A	0	0.66	0.203
Modern terminal	U	A	10398	G	ND3	Non-synonymous	T114A	0	0.66	0.203
Pre-terminal	U	A	10398	G	ND3	Non-synonymous	T114A	0	0.66	0.203
Pre-terminal	U	A	10398	G	ND3	Non-synonymous	T114A	0	0.66	0.203
Pre-terminal	U	A	10398	G	ND3	Non-synonymous	T114A	0	0.66	0.203
Pre-terminal	U	A	10398	G	ND3	Non-synonymous	T114A	0	0.66	0.203
Pre-terminal	U	A	10398	G	ND3	Non-synonymous	T114A	0	0.66	0.203
Pre-terminal	U	A	10398	G	ND3	Non-synonymous	T114A	0	0.66	0.203
Pre-terminal	U	A	10398	G	ND3	Non-synonymous	T114A	0	0.66	0.203
Ancient terminal	JT	A	10398	G	ND3	Non-synonymous	T114A	0	0.66	0.203
Modern terminal	JT	A	10398	G	ND3	Non-synonymous	T114A	0	0.66	0.203
Modern terminal	JT	A	10398	G	ND3	Non-synonymous	T114A	0	0.66	0.203
Modern terminal	JT	A	10398	G	ND3	Non-synonymous	T114A	0	0.66	0.203
Modern terminal	JT	A	10398	G	ND3	Non-synonymous	T114A	0	0.66	0.203
Modern terminal	JT	A	10398	G	ND3	Non-synonymous	T114A	0	0.66	0.203
Modern terminal	JT	A	10398	G	ND3	Non-synonymous	T114A	0	0.66	0.203
Modern terminal	JT	A	10398	G	ND3	Non-synonymous	T114A	0	0.66	0.203
Modern terminal	JT	A	10398	G	ND3	Non-synonymous	T114A	0	0.66	0.203
Modern terminal	JT	A	10398	G	ND3	Non-synonymous	T114A	0	0.66	0.203
Modern terminal	JT	A	10398	G	ND3	Non-synonymous	T114A	0	0.66	0.203
Modern terminal	JT	A	10398	G	ND3	Non-synonymous	T114A	0	0.66	0.203
Modern terminal	JT	A	10398	G	ND3	Non-synonymous	T114A	0	0.66	0.203
Modern terminal	JT	A	10398	G	ND3	Non-synonymous	T114A	0	0.66	0.203
Modern terminal	JT	A	10398	G	ND3	Non-synonymous	T114A	0	0.66	0.203
Modern terminal	JT	A	10398	G	ND3	Non-synonymous	T114A	0	0.66	0.203
Modern terminal	JT	A	10398	G	ND3	Non-synonymous	T114A	0	0.66	0.203
Modern terminal	JT	A	10398	G	ND3	Non-synonymous	T114A	0	0.66	0.203
Pre-terminal	JT	A	10398	G	ND3	Non-synonymous	T114A	0	0.66	0.203
Pre-terminal	JT	A	10398	G	ND3	Non-synonymous	T114A	0	0.66	0.203
Pre-terminal	JT	A	10398	G	ND3	Non-synonymous	T114A	0	0.66	0.203
Pre-terminal	JT	A	10398	G	ND3	Non-synonymous	T114A	0	0.66	0.203
Pre-terminal	JT	A	10398	G	ND3	Non-synonymous	T114A	0	0.66	0.203
Modern terminal	N1	A	10398	G	ND3	Non-synonymous	T114A	0	0.66	0.203
Pre-terminal	N1	A	10398	G	ND3	Non-synonymous	T114A	0	0.66	0.203
Modern terminal	X	A	10398	G	ND3	Non-synonymous	T114A	0	0.66	0.203
Modern terminal	X	A	10398	G	ND3	Non-synonymous	T114A	0	0.66	0.203
Modern terminal	X	A	10398	G	ND3	Non-synonymous	T114A	0	0.66	0.203
Pre-terminal	X	A	10398	G	ND3	Non-synonymous	T114A	0	0.66	0.203

Modern terminal	N2	A	10398	G	ND3	Non-synonymous	T114A	0	0.66	0.203
Pre-terminal	N2	A	10398	G	ND3	Non-synonymous	T114A	0	0.66	0.203
Pre-terminal	N2	A	10398	G	ND3	Non-synonymous	T114A	0	0.66	0.203
Modern terminal	U	C	10399	G	ND3	Non-synonymous	T114S	0.006	0.14	0.159
Ancient terminal	JT	G	10472	T	ND4L	Non-synonymous	M1I	0.972	0	0.86
Pre-terminal	N1	C	10474	T	ND4L	Non-synonymous	P2L	0.002	0.01	0.233
Ancient terminal	R0	T	10477	C	ND4L	Non-synonymous	L3P	0.001	0.45	0.315
Modern terminal	U	A	10479	G	ND4L	Non-synonymous	I4V	0.002	0.72	0.102
Ancient terminal	JT	A	10483	T	ND4L	Non-synonymous	Y5F	0.03	0.19	0.179
Ancient terminal	R0	A	10489	G	ND4L	Non-synonymous	N7S	0.267	0.16	0.237
Ancient terminal	N2	T	10490	G	ND4L	Non-synonymous	N7K	0.931	0	0.517
Modern terminal	R0	T	10493	G	ND4L	Non-synonymous	I8M	0.992	0.13	0.22
Pre-terminal	R0	T	10493	G	ND4L	Non-synonymous	I8M	0.992	0.13	0.22
Ancient terminal	R0	A	10494	G	ND4L	Non-synonymous	M9V	0	0.25	0.243
Modern terminal	U	A	10506	C	ND4L	Non-synonymous	T13P	0.762	0.06	0.475
Pre-terminal	U	A	10506	G	ND4L	Non-synonymous	T13A	0.001	0.37	0.208
Modern terminal	N2	A	10506	G	ND4L	Non-synonymous	T13A	0.001	0.37	0.208
Modern terminal	JT	T	10510	C	ND4L	Non-synonymous	I14T	0.001	0.27	0.261
Modern terminal	R0	C	10511	A	ND4L	Non-synonymous	I14M	0.004	0.49	0.148
Modern terminal	R0	A	10524	G	ND4L	Non-synonymous	M19V	0.001	0.25	0.196
Modern terminal	JT	A	10526	T	ND4L	Non-synonymous	M19I	0.001	0.01	0.213
Pre-terminal	N1	G	10530	A	ND4L	Non-synonymous	V21M	0.03	1	0.096
Modern terminal	U	A	10548	C	ND4L	Non-synonymous	M27L	0.013	0.23	0.498
Modern terminal	JT	T	10576	C	ND4L	Non-synonymous	M36T	0.503	0.01	0.598
Modern terminal	R0	G	10599	A	ND4L	Non-synonymous	A44T	0	1	0.152
Modern terminal	U	G	10599	A	ND4L	Non-synonymous	A44T	0	1	0.152
Pre-terminal	U	G	10599	A	ND4L	Non-synonymous	A44T	0	1	0.152
Modern terminal	N2	G	10599	A	ND4L	Non-synonymous	A44T	0	1	0.152
Ancient terminal	R0	A	10602	G	ND4L	Non-synonymous	T45A	0.003	0.58	0.176
Modern terminal	N2	C	10605	T	ND4L	Non-synonymous	L46F	0.015	0.01	0.43
Pre-terminal	R0	A	10608	G	ND4L	Non-synonymous	M47V	0.014	0.57	0.185
Ancient terminal	R0	T	10609	C	ND4L	Non-synonymous	M47T	0.001	0.21	0.159
Modern terminal	R0	T	10609	C	ND4L	Non-synonymous	M47T	0.001	0.21	0.159
Modern terminal	R0	T	10609	C	ND4L	Non-synonymous	M47T	0.001	0.21	0.159
Modern terminal	R0	T	10609	C	ND4L	Non-synonymous	M47T	0.001	0.21	0.159
Pre-terminal	R0	T	10609	C	ND4L	Non-synonymous	M47T	0.001	0.21	0.159
Pre-terminal	R0	T	10609	C	ND4L	Non-synonymous	M47T	0.001	0.21	0.159
Pre-terminal	R0	T	10609	C	ND4L	Non-synonymous	M47T	0.001	0.21	0.159
Modern terminal	U	T	10609	C	ND4L	Non-synonymous	M47T	0.001	0.21	0.159
Modern terminal	JT	T	10609	C	ND4L	Non-synonymous	M47T	0.001	0.21	0.159
Pre-terminal	R0	A	10620	G	ND4L	Non-synonymous	T51A	0.001	0.58	0.226
Modern terminal	U	A	10641	T	ND4L	Non-synonymous	I58F	0.951	0	0.502
Modern terminal	U	T	10642	C	ND4L	Non-synonymous	I58T	0.846	0.24	0.322
Modern terminal	R0	G	10644	A	ND4L	Non-synonymous	V59M	0.002	1	0.145
Modern terminal	R0	G	10644	A	ND4L	Non-synonymous	V59M	0.002	1	0.145
Modern terminal	U	G	10644	A	ND4L	Non-synonymous	V59M	0.002	1	0.145
Pre-terminal	JT	G	10644	A	ND4L	Non-synonymous	V59M	0.002	1	0.145
Modern terminal	R0	G	10653	A	ND4L	Non-synonymous	A62T	0	0.07	0.161
Pre-terminal	R0	G	10653	A	ND4L	Non-synonymous	A62T	0	0.07	0.161
Pre-terminal	R0	G	10653	A	ND4L	Non-synonymous	A62T	0	0.07	0.161

Modern terminal	U	G	10653	A	ND4L	Non-synonymous	A62T	0	0.07	0.161
Modern terminal	U	G	10653	A	ND4L	Non-synonymous	A62T	0	0.07	0.161
Modern terminal	N2	G	10653	A	ND4L	Non-synonymous	A62T	0	0.07	0.161
Modern terminal	R0	C	10654	T	ND4L	Non-synonymous	A62V	0	0.14	0.265
Pre-terminal	R0	C	10654	T	ND4L	Non-synonymous	A62V	0	0.14	0.265
Modern terminal	U	C	10654	T	ND4L	Non-synonymous	A62V	0	0.14	0.265
Modern terminal	U	C	10654	T	ND4L	Non-synonymous	A62V	0	0.14	0.265
Modern terminal	U	C	10654	T	ND4L	Non-synonymous	A62V	0	0.14	0.265
Pre-terminal	U	C	10654	T	ND4L	Non-synonymous	A62V	0	0.14	0.265
Pre-terminal	U	C	10654	T	ND4L	Non-synonymous	A62V	0	0.14	0.265
Pre-terminal	U	C	10654	T	ND4L	Non-synonymous	A62V	0	0.14	0.265
Modern terminal	JT	C	10654	T	ND4L	Non-synonymous	A62V	0	0.14	0.265
Modern terminal	JT	C	10654	T	ND4L	Non-synonymous	A62V	0	0.14	0.265
Pre-terminal	JT	C	10654	T	ND4L	Non-synonymous	A62V	0	0.14	0.265
Modern terminal	N1	C	10654	T	ND4L	Non-synonymous	A62V	0	0.14	0.265
Ancient terminal	U	G	10662	A	ND4L	Non-synonymous	V65I	0.19	0.02	0.588
Modern terminal	JT	T	10663	C	ND4L	Non-synonymous	V65A	0.946	0.19	0.694
Ancient terminal	R0	C	10669	T	ND4L	Non-synonymous	A67V	0.952	0	0.789
Modern terminal	R0	G	10680	A	ND4L	Non-synonymous	A71T	0.03	0.04	0.761
Modern terminal	R0	G	10680	A	ND4L	Non-synonymous	A71T	0.03	0.04	0.761
Modern terminal	R0	G	10680	A	ND4L	Non-synonymous	A71T	0.03	0.04	0.761
Modern terminal	R0	G	10680	A	ND4L	Non-synonymous	A71T	0.03	0.04	0.761
Modern terminal	R0	G	10680	A	ND4L	Non-synonymous	A71T	0.03	0.04	0.761
Modern terminal	U	G	10680	A	ND4L	Non-synonymous	A71T	0.03	0.04	0.761
Pre-terminal	U	G	10680	A	ND4L	Non-synonymous	A71T	0.03	0.04	0.761
Ancient terminal	JT	C	10681	A	ND4L	Non-synonymous	A71E	0.972	0	0.909
Modern terminal	X	T	10687	G	ND4L	Non-synonymous	V73G	0.995	0.03	0.817
Modern terminal	R0	T	10702	C	ND4L	Non-synonymous	L78P	1	0	0.966
Pre-terminal	R0	G	10704	A	ND4L	Non-synonymous	V79I	0.001	0.1	0.486
Modern terminal	U	G	10704	A	ND4L	Non-synonymous	V79I	0.001	0.1	0.486
Ancient terminal	JT	G	10704	A	ND4L	Non-synonymous	V79I	0.001	0.1	0.486
Modern terminal	R0	T	10707	G	ND4L	Non-synonymous	S80A	0.883	0.69	0.426
Pre-terminal	R0	T	10707	G	ND4L	Non-synonymous	S80A	0.883	0.69	0.426
Ancient terminal	N1	T	10707	G	ND4L	Non-synonymous	S80A	0.883	0.69	0.426
Ancient terminal	JT	G	10731	A	ND4L	Non-synonymous	D88N	0.066	0	0.678
Ancient terminal	R0	A	10735	T	ND4L	Non-synonymous	Y89F	0.684	0.03	0.433
Ancient terminal	U	C	10736	A	ND4L	Nonsense				
Modern terminal	U	A	10744	G	ND4L	Non-synonymous	N92S	0.999	0.08	0.591
Modern terminal	R0	A	10750	G	ND4L	Non-synonymous	N94S	0.005	0.03	0.382
Modern terminal	R0	A	10750	G	ND4L	Non-synonymous	N94S	0.005	0.03	0.382
Modern terminal	R0	A	10750	G	ND4L	Non-synonymous	N94S	0.005	0.03	0.382
Pre-terminal	R0	A	10750	G	ND4L	Non-synonymous	N94S	0.005	0.03	0.382
Pre-terminal	R0	A	10750	G	ND4L	Non-synonymous	N94S	0.005	0.03	0.382
Pre-terminal	R0	A	10750	G	ND4L	Non-synonymous	N94S	0.005	0.03	0.382
Ancient terminal	U	A	10750	G	ND4L	Non-synonymous	N94S	0.005	0.03	0.382
Ancient terminal	U	A	10750	G	ND4L	Non-synonymous	N94S	0.005	0.03	0.382
Modern terminal	U	A	10750	G	ND4L	Non-synonymous	N94S	0.005	0.03	0.382
Pre-terminal	U	A	10750	G	ND4L	Non-synonymous	N94S	0.005	0.03	0.382
Pre-terminal	U	A	10750	G	ND4L	Non-synonymous	N94S	0.005	0.03	0.382
Modern terminal	JT	A	10750	G	ND4L	Non-synonymous	N94S	0.005	0.03	0.382

Pre-terminal	JT	A	10750	G	ND4L	Non-synonymous	N94S	0.005	0.03	0.382
Pre-terminal	JT	A	10750	G	ND4L	Non-synonymous	N94S	0.005	0.03	0.382
Ancient terminal	N1	A	10750	G	ND4L	Non-synonymous	N94S	0.005	0.03	0.382
Modern terminal	X	A	10750	G	ND4L	Non-synonymous	N94S	0.005	0.03	0.382
Modern terminal	N2	A	10750	G	ND4L	Non-synonymous	N94S	0.005	0.03	0.382
Ancient terminal	N2	C	10763	G	ND4L	Non-synonymous	C98W	1	0	0.877
Ancient terminal	N2	C	10763	G	ND4	Non-synonymous	L2V	0.946	0	0.456
Modern terminal	U	A	10768	T	ND4	Non-synonymous	K3N	0.999	0	0.509
Ancient terminal	R0	G	10775	A	ND4	Non-synonymous	V6I	0	1	0.05
Modern terminal	R0	G	10775	A	ND4	Non-synonymous	V6I	0	1	0.05
Pre-terminal	R0	G	10775	A	ND4	Non-synonymous	V6I	0	1	0.05
Modern terminal	U	G	10775	C	ND4	Non-synonymous	V6L	0	0.39	0.227
Pre-terminal	U	G	10775	A	ND4	Non-synonymous	V6I	0	1	0.05
Modern terminal	JT	G	10775	A	ND4	Non-synonymous	V6I	0	1	0.05
Modern terminal	JT	G	10775	A	ND4	Non-synonymous	V6I	0	1	0.05
Modern terminal	JT	G	10775	A	ND4	Non-synonymous	V6I	0	1	0.05
Pre-terminal	X	G	10775	A	ND4	Non-synonymous	V6I	0	1	0.05
Modern terminal	R0	A	10784	G	ND4	Non-synonymous	I9V	0	0.15	0.12
Pre-terminal	U	A	10784	G	ND4	Non-synonymous	I9V	0	0.15	0.12
Pre-terminal	U	A	10784	G	ND4	Non-synonymous	I9V	0	0.15	0.12
Ancient terminal	N2	C	10796	T	ND4	Non-synonymous	P13S	1	0.01	0.41
Ancient terminal	R0	C	10808	T	ND4	Non-synonymous	L17F	0.996	0.27	0.257
Pre-terminal	U	C	10808	A	ND4	Non-synonymous	L17I	0.971	0.19	0.202
Ancient terminal	X	C	10808	T	ND4	Non-synonymous	L17F	0.996	0.27	0.257
Modern terminal	R0	T	10811	C	ND4	Non-synonymous	S18P	0.978	0.08	0.734
Modern terminal	R0	A	10832	G	ND4	Non-synonymous	I25V	0.788	0.51	0.193
Modern terminal	N2	A	10841	G	ND4	Non-synonymous	T28A	0.993	0.05	0.332
Pre-terminal	N2	C	10845	T	ND4	Non-synonymous	T29I	0.996	0.52	0.325
Modern terminal	R0	A	10850	G	ND4	Non-synonymous	S31G	0.999	0.04	0.329
Modern terminal	R0	A	10856	C	ND4	Non-synonymous	I33L	0.762	1	0.11
Pre-terminal	U	G	10863	A	ND4	Non-synonymous	S35N	0.962	0	0.274
Ancient terminal	JT	T	10866	C	ND4	Non-synonymous	I36T	0.294	0.15	0.145
Modern terminal	X	T	10875	C	ND4	Non-synonymous	L39P	1	0	0.672
Modern terminal	R0	A	10887	G	ND4	Non-synonymous	N43S	0.003	0.14	0.104
Modern terminal	R0	A	10892	T	ND4	Non-synonymous	I45F	0.162	0.7	0.07
Modern terminal	U	A	10892	T	ND4	Non-synonymous	I45F	0.162	0.7	0.07
Pre-terminal	JT	T	10893	A	ND4	Non-synonymous	I45N	0.936	0.31	0.148
Modern terminal	R0	A	10899	G	ND4	Non-synonymous	N47S	0.003	0.36	0.073
Modern terminal	U	A	10899	G	ND4	Non-synonymous	N47S	0.003	0.36	0.073
Modern terminal	JT	A	10899	G	ND4	Non-synonymous	N47S	0.003	0.36	0.073
Modern terminal	JT	A	10899	G	ND4	Non-synonymous	N47S	0.003	0.36	0.073
Modern terminal	JT	A	10899	G	ND4	Non-synonymous	N47S	0.003	0.36	0.073
Modern terminal	R0	T	10907	C	ND4	Non-synonymous	F50L	0.001	0.75	0.043
Modern terminal	R0	T	10907	C	ND4	Non-synonymous	F50L	0.001	0.75	0.043
Modern terminal	R0	T	10907	C	ND4	Non-synonymous	F50L	0.001	0.75	0.043
Pre-terminal	R0	T	10907	C	ND4	Non-synonymous	F50L	0.001	0.75	0.043
Modern terminal	U	T	10907	C	ND4	Non-synonymous	F50L	0.001	0.75	0.043
Modern terminal	U	T	10907	C	ND4	Non-synonymous	F50L	0.001	0.75	0.043
Modern terminal	U	T	10907	C	ND4	Non-synonymous	F50L	0.001	0.75	0.043
Pre-terminal	U	T	10907	C	ND4	Non-synonymous	F50L	0.001	0.75	0.043

Modern terminal	JT	T	10907	C	ND4	Non-synonymous	F50L	0.001	0.75	0.043
Pre-terminal	JT	T	10907	C	ND4	Non-synonymous	F50L	0.001	0.75	0.043
Modern terminal	R0	T	10908	A	ND4	Non-synonymous	F50Y	0	1	0.078
Modern terminal	U	T	10908	C	ND4	Non-synonymous	F50S	0.019	0.42	0.112
Pre-terminal	U	T	10908	C	ND4	Non-synonymous	F50S	0.019	0.42	0.112
Pre-terminal	JT	T	10908	C	ND4	Non-synonymous	F50S	0.019	0.42	0.112
Modern terminal	R0	G	10914	A	ND4	Non-synonymous	C52Y	0	0.76	0.088
Modern terminal	JT	G	10914	A	ND4	Non-synonymous	C52Y	0	0.76	0.088
Pre-terminal	JT	G	10914	A	ND4	Non-synonymous	C52Y	0	0.76	0.088
Ancient terminal	U	T	10916	C	ND4	Non-synonymous	S53P	0.993	0.01	0.338
Modern terminal	U	C	10920	T	ND4	Non-synonymous	P54L	0	0.69	0.035
Modern terminal	U	C	10920	T	ND4	Non-synonymous	P54L	0	0.69	0.035
Pre-terminal	U	C	10920	T	ND4	Non-synonymous	P54L	0	0.69	0.035
Modern terminal	JT	C	10920	T	ND4	Non-synonymous	P54L	0	0.69	0.035
Modern terminal	JT	C	10920	T	ND4	Non-synonymous	P54L	0	0.69	0.035
Modern terminal	JT	C	10920	T	ND4	Non-synonymous	P54L	0	0.69	0.035
Modern terminal	N1	C	10920	T	ND4	Non-synonymous	P54L	0	0.69	0.035
Ancient terminal	X	C	10920	T	ND4	Non-synonymous	P54L	0	0.69	0.035
Modern terminal	R0	A	10922	G	ND4	Non-synonymous	T55A	0.001	0.51	0.091
Pre-terminal	R0	A	10922	G	ND4	Non-synonymous	T55A	0.001	0.51	0.091
Modern terminal	R0	C	10923	T	ND4	Non-synonymous	T55I	0.003	0.41	0.108
Modern terminal	U	C	10923	T	ND4	Non-synonymous	T55I	0.003	0.41	0.108
Modern terminal	R0	T	10931	C	ND4	Non-synonymous	S58P	0.987	0.13	0.272
Pre-terminal	R0	T	10931	C	ND4	Non-synonymous	S58P	0.987	0.13	0.272
Ancient terminal	U	G	10934	A	ND4	Non-synonymous	D59N	0.994	0	0.15
Modern terminal	N2	G	10934	A	ND4	Non-synonymous	D59N	0.994	0	0.15
Ancient terminal	R0	C	10950	G	ND4	Non-synonymous	P64R	1	0	0.327
Ancient terminal	R0	C	10950	T	ND4	Non-synonymous	P64L	1	0	0.251
Ancient terminal	U	C	10950	T	ND4	Non-synonymous	P64L	1	0	0.251
Ancient terminal	U	A	10960	T	ND4	Non-synonymous	M67I	0.789	1	0.074
Ancient terminal	JT	T	10970	G	ND4	Non-synonymous	W71G	1	0	0.708
Modern terminal	N1	C	10973	T	ND4	Non-synonymous	L72F	0.999	0.03	0.231
Ancient terminal	U	A	10988	T	ND4	Non-synonymous	I77F	0.99	0.02	0.235
Modern terminal	R0	G	10994	A	ND4	Non-synonymous	A79T	0.999	0	0.235
Pre-terminal	U	C	11013	T	ND4	Non-synonymous	S85F	0.379	0.1	0.103
Modern terminal	N1	C	11013	A	ND4	Non-synonymous	S85Y	0.002	0.09	0.112
Modern terminal	U	A	11015	G	ND4	Non-synonymous	S86G	0.036	0.08	0.066
Ancient terminal	JT	A	11015	T	ND4	Non-synonymous	S86C	0.562	0.01	0.077
Modern terminal	R0	G	11016	A	ND4	Non-synonymous	S86N	0	0.44	0.042
Modern terminal	R0	G	11016	A	ND4	Non-synonymous	S86N	0	0.44	0.042
Modern terminal	R0	G	11016	A	ND4	Non-synonymous	S86N	0	0.44	0.042
Pre-terminal	R0	G	11016	A	ND4	Non-synonymous	S86N	0	0.44	0.042
Pre-terminal	R0	G	11016	A	ND4	Non-synonymous	S86N	0	0.44	0.042
Pre-terminal	R0	G	11016	A	ND4	Non-synonymous	S86N	0	0.44	0.042
Pre-terminal	R0	G	11016	A	ND4	Non-synonymous	S86N	0	0.44	0.042
Pre-terminal	R0	G	11016	A	ND4	Non-synonymous	S86N	0	0.44	0.042
Pre-terminal	R0	G	11016	A	ND4	Non-synonymous	S86N	0	0.44	0.042
Pre-terminal	R0	G	11016	A	ND4	Non-synonymous	S86N	0	0.44	0.042
Pre-terminal	R0	G	11016	A	ND4	Non-synonymous	S86N	0	0.44	0.042
Ancient terminal	U	G	11016	A	ND4	Non-synonymous	S86N	0	0.44	0.042

Ancient terminal	U	G	11016	A	ND4	Non-synonymous	S86N	0	0.44	0.042
Modern terminal	U	G	11016	A	ND4	Non-synonymous	S86N	0	0.44	0.042
Modern terminal	U	G	11016	A	ND4	Non-synonymous	S86N	0	0.44	0.042
Modern terminal	U	G	11016	A	ND4	Non-synonymous	S86N	0	0.44	0.042
Modern terminal	U	G	11016	A	ND4	Non-synonymous	S86N	0	0.44	0.042
Pre-terminal	U	G	11016	A	ND4	Non-synonymous	S86N	0	0.44	0.042
Pre-terminal	U	G	11016	A	ND4	Non-synonymous	S86N	0	0.44	0.042
Pre-terminal	U	G	11016	A	ND4	Non-synonymous	S86N	0	0.44	0.042
Modern terminal	JT	G	11016	A	ND4	Non-synonymous	S86N	0	0.44	0.042
Modern terminal	JT	G	11016	A	ND4	Non-synonymous	S86N	0	0.44	0.042
Modern terminal	JT	G	11016	A	ND4	Non-synonymous	S86N	0	0.44	0.042
Modern terminal	JT	G	11016	A	ND4	Non-synonymous	S86N	0	0.44	0.042
Modern terminal	JT	G	11016	A	ND4	Non-synonymous	S86N	0	0.44	0.042
Modern terminal	JT	G	11016	A	ND4	Non-synonymous	S86N	0	0.44	0.042
Pre-terminal	JT	G	11016	A	ND4	Non-synonymous	S86N	0	0.44	0.042
Pre-terminal	JT	G	11016	A	ND4	Non-synonymous	S86N	0	0.44	0.042
Pre-terminal	N1	G	11016	A	ND4	Non-synonymous	S86N	0	0.44	0.042
Pre-terminal	N1	G	11016	A	ND4	Non-synonymous	S86N	0	0.44	0.042
Pre-terminal	X	G	11016	A	ND4	Non-synonymous	S86N	0	0.44	0.042
Modern terminal	N2	G	11016	A	ND4	Non-synonymous	S86N	0	0.44	0.042
Modern terminal	R0	T	11025	C	ND4	Non-synonymous	L89P	0.008	0.28	0.086
Modern terminal	R0	T	11025	C	ND4	Non-synonymous	L89P	0.008	0.28	0.086
Pre-terminal	R0	T	11025	C	ND4	Non-synonymous	L89P	0.008	0.28	0.086
Modern terminal	U	T	11025	C	ND4	Non-synonymous	L89P	0.008	0.28	0.086
Pre-terminal	U	T	11025	C	ND4	Non-synonymous	L89P	0.008	0.28	0.086
Pre-terminal	U	T	11025	C	ND4	Non-synonymous	L89P	0.008	0.28	0.086
Pre-terminal	U	T	11025	C	ND4	Non-synonymous	L89P	0.008	0.28	0.086
Pre-terminal	JT	T	11025	C	ND4	Non-synonymous	L89P	0.008	0.28	0.086
Pre-terminal	N1	T	11025	C	ND4	Non-synonymous	L89P	0.008	0.28	0.086
Modern terminal	N2	T	11025	C	ND4	Non-synonymous	L89P	0.008	0.28	0.086
Modern terminal	N2	T	11025	C	ND4	Non-synonymous	L89P	0.008	0.28	0.086
Ancient terminal	R0	G	11031	T	ND4	Non-synonymous	R91L	1	0.11	0.15
Modern terminal	U	A	11036	G	ND4	Non-synonymous	K93E	0.993	0.01	0.161
Modern terminal	R0	C	11039	T	ND4	Non-synonymous	L94F	0.996	0.11	0.158
Pre-terminal	R0	C	11039	T	ND4	Non-synonymous	L94F	0.996	0.11	0.158
Ancient terminal	U	C	11039	T	ND4	Non-synonymous	L94F	0.996	0.11	0.158
Pre-terminal	U	C	11039	T	ND4	Non-synonymous	L94F	0.996	0.11	0.158
Ancient terminal	R0	C	11054	A	ND4	Non-synonymous	L99M	1	0.03	0.096
Pre-terminal	R0	C	11061	T	ND4	Non-synonymous	S101F	0.726	0.19	0.11
Pre-terminal	R0	C	11061	T	ND4	Non-synonymous	S101F	0.726	0.19	0.11
Modern terminal	U	C	11061	T	ND4	Non-synonymous	S101F	0.726	0.19	0.11
Modern terminal	JT	C	11061	G	ND4	Non-synonymous	S101C	0.939	0.04	0.183
Pre-terminal	R0	A	11069	G	ND4	Non-synonymous	I104V	0.002	0.59	0.089
Modern terminal	U	A	11069	G	ND4	Non-synonymous	I104V	0.002	0.59	0.089
Ancient terminal	U	C	11071	A	ND4	Non-synonymous	I104M	0.984	0.25	0.144
Modern terminal	R0	A	11081	T	ND4	Non-synonymous	M108L	0.296	0.64	0.414
Modern terminal	R0	A	11084	G	ND4	Non-synonymous	T109A	0.993	0.59	0.461
Modern terminal	R0	A	11084	G	ND4	Non-synonymous	T109A	0.993	0.59	0.461
Modern terminal	R0	A	11084	G	ND4	Non-synonymous	T109A	0.993	0.59	0.461
Modern terminal	R0	A	11084	G	ND4	Non-synonymous	T109A	0.993	0.59	0.461

Modern terminal	R0	A	11084	G	ND4	Non-synonymous	T109A	0.993	0.59	0.461
Modern terminal	R0	A	11084	G	ND4	Non-synonymous	T109A	0.993	0.59	0.461
Modern terminal	R0	A	11084	G	ND4	Non-synonymous	T109A	0.993	0.59	0.461
Modern terminal	R0	A	11084	G	ND4	Non-synonymous	T109A	0.993	0.59	0.461
Modern terminal	R0	A	11084	G	ND4	Non-synonymous	T109A	0.993	0.59	0.461
Modern terminal	R0	A	11084	G	ND4	Non-synonymous	T109A	0.993	0.59	0.461
Pre-terminal	R0	A	11084	G	ND4	Non-synonymous	T109A	0.993	0.59	0.461
Pre-terminal	R0	A	11084	G	ND4	Non-synonymous	T109A	0.993	0.59	0.461
Pre-terminal	R0	A	11084	G	ND4	Non-synonymous	T109A	0.993	0.59	0.461
Pre-terminal	R0	A	11084	G	ND4	Non-synonymous	T109A	0.993	0.59	0.461
Ancient terminal	U	A	11084	G	ND4	Non-synonymous	T109A	0.993	0.59	0.461
Ancient terminal	U	A	11084	G	ND4	Non-synonymous	T109A	0.993	0.59	0.461
Modern terminal	U	A	11084	G	ND4	Non-synonymous	T109A	0.993	0.59	0.461
Modern terminal	U	A	11084	G	ND4	Non-synonymous	T109A	0.993	0.59	0.461
Modern terminal	U	A	11084	G	ND4	Non-synonymous	T109A	0.993	0.59	0.461
Modern terminal	U	A	11084	G	ND4	Non-synonymous	T109A	0.993	0.59	0.461
Modern terminal	U	A	11084	G	ND4	Non-synonymous	T109A	0.993	0.59	0.461
Modern terminal	U	A	11084	G	ND4	Non-synonymous	T109A	0.993	0.59	0.461
Modern terminal	U	A	11084	G	ND4	Non-synonymous	T109A	0.993	0.59	0.461
Modern terminal	U	A	11084	G	ND4	Non-synonymous	T109A	0.993	0.59	0.461
Pre-terminal	U	A	11084	G	ND4	Non-synonymous	T109A	0.993	0.59	0.461
Pre-terminal	U	A	11084	G	ND4	Non-synonymous	T109A	0.993	0.59	0.461
Pre-terminal	U	A	11084	G	ND4	Non-synonymous	T109A	0.993	0.59	0.461
Ancient terminal	JT	A	11084	G	ND4	Non-synonymous	T109A	0.993	0.59	0.461
Modern terminal	JT	A	11084	G	ND4	Non-synonymous	T109A	0.993	0.59	0.461
Modern terminal	JT	A	11084	G	ND4	Non-synonymous	T109A	0.993	0.59	0.461
Modern terminal	JT	A	11084	G	ND4	Non-synonymous	T109A	0.993	0.59	0.461
Pre-terminal	JT	A	11084	G	ND4	Non-synonymous	T109A	0.993	0.59	0.461
Pre-terminal	JT	A	11084	G	ND4	Non-synonymous	T109A	0.993	0.59	0.461
Modern terminal	N1	A	11084	G	ND4	Non-synonymous	T109A	0.993	0.59	0.461
Modern terminal	X	A	11084	G	ND4	Non-synonymous	T109A	0.993	0.59	0.461
Modern terminal	X	A	11084	G	ND4	Non-synonymous	T109A	0.993	0.59	0.461
Modern terminal	N2	A	11084	G	ND4	Non-synonymous	T109A	0.993	0.59	0.461
Modern terminal	N2	A	11084	G	ND4	Non-synonymous	T109A	0.993	0.59	0.461
Modern terminal	R0	T	11087	C	ND4	Non-synonymous	F110L	0.998	0	0.65
Modern terminal	R0	T	11087	C	ND4	Non-synonymous	F110L	0.998	0	0.65
Pre-terminal	R0	T	11087	C	ND4	Non-synonymous	F110L	0.998	0	0.65
Pre-terminal	R0	T	11087	C	ND4	Non-synonymous	F110L	0.998	0	0.65
Pre-terminal	R0	T	11087	C	ND4	Non-synonymous	F110L	0.998	0	0.65
Modern terminal	U	T	11087	C	ND4	Non-synonymous	F110L	0.998	0	0.65
Modern terminal	U	T	11087	C	ND4	Non-synonymous	F110L	0.998	0	0.65
Modern terminal	U	T	11087	C	ND4	Non-synonymous	F110L	0.998	0	0.65
Pre-terminal	U	T	11087	C	ND4	Non-synonymous	F110L	0.998	0	0.65
Ancient terminal	JT	T	11087	C	ND4	Non-synonymous	F110L	0.998	0	0.65
Ancient terminal	JT	T	11087	C	ND4	Non-synonymous	F110L	0.998	0	0.65
Modern terminal	JT	T	11087	C	ND4	Non-synonymous	F110L	0.998	0	0.65
Modern terminal	JT	T	11087	C	ND4	Non-synonymous	F110L	0.998	0	0.65
Pre-terminal	JT	T	11087	C	ND4	Non-synonymous	F110L	0.998	0	0.65
Pre-terminal	JT	T	11087	C	ND4	Non-synonymous	F110L	0.998	0	0.65
Pre-terminal	N1	T	11087	C	ND4	Non-synonymous	F110L	0.998	0	0.65

Modern terminal	JT	T	11088	C	ND4	Non-synonymous	F110S	1	0	0.837
Modern terminal	R0	A	11090	G	ND4	Non-synonymous	T111A	0.984	0.47	0.404
Ancient terminal	U	G	11093	C	ND4	Non-synonymous	A112P	0.988	0	0.734
Modern terminal	JT	T	11111	C	ND4	Non-synonymous	F118L	0.998	0.06	0.695
Modern terminal	U	T	11120	C	ND4	Non-synonymous	F121L	0.001	1	0.091
Modern terminal	JT	C	11122	A	ND4	Non-synonymous	F121L	0.001	1	0.091
Modern terminal	R0	A	11129	T	ND4	Non-synonymous	T124S	0.016	0.38	0.239
Modern terminal	JT	A	11129	T	ND4	Non-synonymous	T124S	0.016	0.38	0.239
Ancient terminal	N2	T	11139	G	ND4	Non-synonymous	I127S	1	0	0.773
Ancient terminal	JT	C	11140	A	ND4	Non-synonymous	I127M	1	0	0.504
Modern terminal	U	C	11141	A	ND4	Non-synonymous	P128T	0.997	0	0.792
Modern terminal	R0	A	11144	G	ND4	Non-synonymous	T129A	0.978	0.01	0.651
Pre-terminal	R0	A	11144	T	ND4	Non-synonymous	T129S	0.978	0.01	0.433
Ancient terminal	U	A	11144	G	ND4	Non-synonymous	T129A	0.978	0.01	0.651
Modern terminal	U	A	11144	T	ND4	Non-synonymous	T129S	0.978	0.01	0.433
Modern terminal	U	A	11144	T	ND4	Non-synonymous	T129S	0.978	0.01	0.433
Modern terminal	R0	G	11150	A	ND4	Non-synonymous	A131T	0.005	0.05	0.277
Pre-terminal	R0	G	11150	A	ND4	Non-synonymous	A131T	0.005	0.05	0.277
Pre-terminal	R0	G	11150	A	ND4	Non-synonymous	A131T	0.005	0.05	0.277
Modern terminal	U	G	11150	A	ND4	Non-synonymous	A131T	0.005	0.05	0.277
Pre-terminal	U	G	11150	A	ND4	Non-synonymous	A131T	0.005	0.05	0.277
Pre-terminal	U	G	11150	A	ND4	Non-synonymous	A131T	0.005	0.05	0.277
Pre-terminal	U	G	11150	A	ND4	Non-synonymous	A131T	0.005	0.05	0.277
Pre-terminal	U	G	11150	A	ND4	Non-synonymous	A131T	0.005	0.05	0.277
Modern terminal	JT	G	11150	A	ND4	Non-synonymous	A131T	0.005	0.05	0.277
Modern terminal	JT	G	11150	A	ND4	Non-synonymous	A131T	0.005	0.05	0.277
Modern terminal	JT	G	11150	A	ND4	Non-synonymous	A131T	0.005	0.05	0.277
Modern terminal	JT	G	11150	A	ND4	Non-synonymous	A131T	0.005	0.05	0.277
Modern terminal	JT	G	11150	A	ND4	Non-synonymous	A131T	0.005	0.05	0.277
Pre-terminal	JT	G	11150	A	ND4	Non-synonymous	A131T	0.005	0.05	0.277
Pre-terminal	JT	G	11150	A	ND4	Non-synonymous	A131T	0.005	0.05	0.277
Modern terminal	N1	G	11150	A	ND4	Non-synonymous	A131T	0.005	0.05	0.277
Ancient terminal	R0	C	11151	T	ND4	Non-synonymous	A131V	0	0.14	0.501
Modern terminal	R0	C	11151	T	ND4	Non-synonymous	A131V	0	0.14	0.501
Modern terminal	R0	C	11151	T	ND4	Non-synonymous	A131V	0	0.14	0.501
Modern terminal	R0	C	11151	T	ND4	Non-synonymous	A131V	0	0.14	0.501
Modern terminal	R0	C	11151	T	ND4	Non-synonymous	A131V	0	0.14	0.501
Pre-terminal	R0	C	11151	T	ND4	Non-synonymous	A131V	0	0.14	0.501
Pre-terminal	R0	C	11151	T	ND4	Non-synonymous	A131V	0	0.14	0.501
Pre-terminal	R0	C	11151	T	ND4	Non-synonymous	A131V	0	0.14	0.501
Ancient terminal	U	C	11151	T	ND4	Non-synonymous	A131V	0	0.14	0.501
Ancient terminal	U	C	11151	T	ND4	Non-synonymous	A131V	0	0.14	0.501
Modern terminal	U	C	11151	T	ND4	Non-synonymous	A131V	0	0.14	0.501
Modern terminal	U	C	11151	T	ND4	Non-synonymous	A131V	0	0.14	0.501
Modern terminal	U	C	11151	T	ND4	Non-synonymous	A131V	0	0.14	0.501
Pre-terminal	U	C	11151	T	ND4	Non-synonymous	A131V	0	0.14	0.501
Pre-terminal	U	C	11151	T	ND4	Non-synonymous	A131V	0	0.14	0.501
Pre-terminal	U	C	11151	T	ND4	Non-synonymous	A131V	0	0.14	0.501
Modern terminal	JT	C	11151	T	ND4	Non-synonymous	A131V	0	0.14	0.501
Modern terminal	JT	C	11151	T	ND4	Non-synonymous	A131V	0	0.14	0.501

Modern terminal	JT	C	11151	T	ND4	Non-synonymous	A131V	0	0.14	0.501
Modern terminal	JT	C	11151	T	ND4	Non-synonymous	A131V	0	0.14	0.501
Pre-terminal	JT	C	11151	T	ND4	Non-synonymous	A131V	0	0.14	0.501
Pre-terminal	JT	C	11151	T	ND4	Non-synonymous	A131V	0	0.14	0.501
Modern terminal	U	T	11157	C	ND4	Non-synonymous	I133T	0.999	0	0.628
Modern terminal	R0	A	11172	G	ND4	Non-synonymous	N138S	0.993	0.05	0.376
Modern terminal	R0	A	11172	G	ND4	Non-synonymous	N138S	0.993	0.05	0.376
Pre-terminal	R0	A	11172	G	ND4	Non-synonymous	N138S	0.993	0.05	0.376
Pre-terminal	R0	A	11172	G	ND4	Non-synonymous	N138S	0.993	0.05	0.376
Modern terminal	U	A	11172	G	ND4	Non-synonymous	N138S	0.993	0.05	0.376
Modern terminal	U	A	11172	G	ND4	Non-synonymous	N138S	0.993	0.05	0.376
Modern terminal	U	A	11172	G	ND4	Non-synonymous	N138S	0.993	0.05	0.376
Pre-terminal	U	A	11172	G	ND4	Non-synonymous	N138S	0.993	0.05	0.376
Modern terminal	N1	A	11172	G	ND4	Non-synonymous	N138S	0.993	0.05	0.376
Modern terminal	X	A	11172	G	ND4	Non-synonymous	N138S	0.993	0.05	0.376
Modern terminal	JT	C	11177	T	ND4	Non-synonymous	P140S	0.999	0.27	0.319
Pre-terminal	JT	C	11177	T	ND4	Non-synonymous	P140S	0.999	0.27	0.319
Ancient terminal	U	G	11180	A	ND4	Non-synonymous	E141K	0.978	0	0.724
Ancient terminal	R0	G	11192	T	ND4	Non-synonymous	A145S	0.006	0	0.41
Ancient terminal	U	G	11195	T	ND4	Non-synonymous	G146C	1	0	0.787
Modern terminal	R0	T	11204	C	ND4	Non-synonymous	F149L	0.001	0.11	0.596
Modern terminal	R0	T	11204	C	ND4	Non-synonymous	F149L	0.001	0.11	0.596
Modern terminal	R0	T	11204	C	ND4	Non-synonymous	F149L	0.001	0.11	0.596
Modern terminal	R0	T	11204	C	ND4	Non-synonymous	F149L	0.001	0.11	0.596
Modern terminal	R0	T	11204	C	ND4	Non-synonymous	F149L	0.001	0.11	0.596
Modern terminal	R0	T	11204	C	ND4	Non-synonymous	F149L	0.001	0.11	0.596
Modern terminal	R0	T	11204	C	ND4	Non-synonymous	F149L	0.001	0.11	0.596
Modern terminal	R0	T	11204	C	ND4	Non-synonymous	F149L	0.001	0.11	0.596
Modern terminal	R0	T	11204	C	ND4	Non-synonymous	F149L	0.001	0.11	0.596
Modern terminal	R0	T	11204	C	ND4	Non-synonymous	F149L	0.001	0.11	0.596
Modern terminal	R0	T	11204	C	ND4	Non-synonymous	F149L	0.001	0.11	0.596
Modern terminal	R0	T	11204	C	ND4	Non-synonymous	F149L	0.001	0.11	0.596
Modern terminal	R0	T	11204	C	ND4	Non-synonymous	F149L	0.001	0.11	0.596
Modern terminal	R0	T	11204	C	ND4	Non-synonymous	F149L	0.001	0.11	0.596
Pre-terminal	R0	T	11204	C	ND4	Non-synonymous	F149L	0.001	0.11	0.596
Pre-terminal	R0	T	11204	C	ND4	Non-synonymous	F149L	0.001	0.11	0.596
Pre-terminal	R0	T	11204	C	ND4	Non-synonymous	F149L	0.001	0.11	0.596
Ancient terminal	U	T	11204	C	ND4	Non-synonymous	F149L	0.001	0.11	0.596
Ancient terminal	U	T	11204	C	ND4	Non-synonymous	F149L	0.001	0.11	0.596
Modern terminal	U	T	11204	C	ND4	Non-synonymous	F149L	0.001	0.11	0.596
Modern terminal	U	T	11204	C	ND4	Non-synonymous	F149L	0.001	0.11	0.596
Modern terminal	U	T	11204	C	ND4	Non-synonymous	F149L	0.001	0.11	0.596
Modern terminal	U	T	11204	C	ND4	Non-synonymous	F149L	0.001	0.11	0.596
Modern terminal	U	T	11204	C	ND4	Non-synonymous	F149L	0.001	0.11	0.596
Modern terminal	U	T	11204	C	ND4	Non-synonymous	F149L	0.001	0.11	0.596
Modern terminal	U	T	11204	C	ND4	Non-synonymous	F149L	0.001	0.11	0.596
Modern terminal	U	T	11204	C	ND4	Non-synonymous	F149L	0.001	0.11	0.596
Modern terminal	U	T	11204	C	ND4	Non-synonymous	F149L	0.001	0.11	0.596
Modern terminal	U	T	11204	C	ND4	Non-synonymous	F149L	0.001	0.11	0.596
Pre-terminal	U	T	11204	C	ND4	Non-synonymous	F149L	0.001	0.11	0.596

Pre-terminal	U	T	11204	C	ND4	Non-synonymous	F149L	0.001	0.11	0.596
Pre-terminal	U	T	11204	C	ND4	Non-synonymous	F149L	0.001	0.11	0.596
Pre-terminal	U	T	11204	C	ND4	Non-synonymous	F149L	0.001	0.11	0.596
Modern terminal	JT	T	11204	C	ND4	Non-synonymous	F149L	0.001	0.11	0.596
Modern terminal	JT	T	11204	C	ND4	Non-synonymous	F149L	0.001	0.11	0.596
Modern terminal	JT	T	11204	C	ND4	Non-synonymous	F149L	0.001	0.11	0.596
Pre-terminal	JT	T	11204	C	ND4	Non-synonymous	F149L	0.001	0.11	0.596
Pre-terminal	JT	T	11204	C	ND4	Non-synonymous	F149L	0.001	0.11	0.596
Pre-terminal	JT	T	11204	C	ND4	Non-synonymous	F149L	0.001	0.11	0.596
Pre-terminal	JT	T	11204	C	ND4	Non-synonymous	F149L	0.001	0.11	0.596
Pre-terminal	JT	T	11204	C	ND4	Non-synonymous	F149L	0.001	0.11	0.596
Pre-terminal	JT	T	11204	C	ND4	Non-synonymous	F149L	0.001	0.11	0.596
Pre-terminal	JT	T	11204	C	ND4	Non-synonymous	F149L	0.001	0.11	0.596
Pre-terminal	JT	T	11204	C	ND4	Non-synonymous	F149L	0.001	0.11	0.596
Ancient terminal	N1	T	11204	C	ND4	Non-synonymous	F149L	0.001	0.11	0.596
Pre-terminal	N1	T	11204	C	ND4	Non-synonymous	F149L	0.001	0.11	0.596
Pre-terminal	X	T	11204	C	ND4	Non-synonymous	F149L	0.001	0.11	0.596
Pre-terminal	N2	T	11204	C	ND4	Non-synonymous	F149L	0.001	0.11	0.596
Modern terminal	X	C	11229	T	ND4	Non-synonymous	S157F	0.999	0	0.506
Modern terminal	R0	A	11252	G	ND4	Non-synonymous	I165V	0.001	0.16	0.086
Modern terminal	R0	A	11252	G	ND4	Non-synonymous	I165V	0.001	0.16	0.086
Modern terminal	U	A	11252	G	ND4	Non-synonymous	I165V	0.001	0.16	0.086
Modern terminal	JT	A	11252	G	ND4	Non-synonymous	I165V	0.001	0.16	0.086
Pre-terminal	JT	A	11252	G	ND4	Non-synonymous	I165V	0.001	0.16	0.086
Ancient terminal	R0	T	11253	C	ND4	Non-synonymous	I165T	0.023	0.11	0.179
Modern terminal	R0	T	11253	C	ND4	Non-synonymous	I165T	0.023	0.11	0.179
Modern terminal	R0	T	11253	C	ND4	Non-synonymous	I165T	0.023	0.11	0.179
Modern terminal	R0	T	11253	C	ND4	Non-synonymous	I165T	0.023	0.11	0.179
Modern terminal	R0	T	11253	C	ND4	Non-synonymous	I165T	0.023	0.11	0.179
Modern terminal	R0	T	11253	C	ND4	Non-synonymous	I165T	0.023	0.11	0.179
Modern terminal	R0	T	11253	C	ND4	Non-synonymous	I165T	0.023	0.11	0.179
Pre-terminal	R0	T	11253	C	ND4	Non-synonymous	I165T	0.023	0.11	0.179
Pre-terminal	R0	T	11253	C	ND4	Non-synonymous	I165T	0.023	0.11	0.179
Modern terminal	U	T	11253	C	ND4	Non-synonymous	I165T	0.023	0.11	0.179
Modern terminal	U	T	11253	C	ND4	Non-synonymous	I165T	0.023	0.11	0.179
Modern terminal	U	T	11253	C	ND4	Non-synonymous	I165T	0.023	0.11	0.179
Modern terminal	U	T	11253	C	ND4	Non-synonymous	I165T	0.023	0.11	0.179
Modern terminal	JT	T	11253	C	ND4	Non-synonymous	I165T	0.023	0.11	0.179
Modern terminal	JT	T	11253	C	ND4	Non-synonymous	I165T	0.023	0.11	0.179
Modern terminal	JT	T	11253	C	ND4	Non-synonymous	I165T	0.023	0.11	0.179
Pre-terminal	JT	T	11253	C	ND4	Non-synonymous	I165T	0.023	0.11	0.179
Pre-terminal	JT	T	11253	C	ND4	Non-synonymous	I165T	0.023	0.11	0.179
Modern terminal	N1	T	11253	C	ND4	Non-synonymous	I165T	0.023	0.11	0.179
Modern terminal	R0	T	11255	C	ND4	Non-synonymous	Y166H	0	0.48	0.343
Modern terminal	R0	T	11255	C	ND4	Non-synonymous	Y166H	0	0.48	0.343
Pre-terminal	R0	T	11255	C	ND4	Non-synonymous	Y166H	0	0.48	0.343
Modern terminal	U	T	11255	C	ND4	Non-synonymous	Y166H	0	0.48	0.343
Modern terminal	U	T	11255	C	ND4	Non-synonymous	Y166H	0	0.48	0.343
Pre-terminal	U	T	11255	C	ND4	Non-synonymous	Y166H	0	0.48	0.343
Pre-terminal	U	T	11255	C	ND4	Non-synonymous	Y166H	0	0.48	0.343

Modern terminal	JT	T	11255	C	ND4	Non-synonymous	Y166H	0	0.48	0.343
Pre-terminal	N1	T	11255	C	ND4	Non-synonymous	Y166H	0	0.48	0.343
Modern terminal	U	A	11258	T	ND4	Non-synonymous	T167S	0.993	0.1	0.146
Ancient terminal	JT	A	11262	T	ND4	Non-synonymous	H168L	0.019	0.33	0.37
Ancient terminal	JT	A	11264	C	ND4	Non-synonymous	N169H	0.998	0.01	0.31
Modern terminal	U	C	11268	T	ND4	Non-synonymous	T170I	0.998	0.45	0.202
Pre-terminal	U	C	11268	T	ND4	Non-synonymous	T170I	0.998	0.45	0.202
Ancient terminal	X	C	11277	T	ND4	Non-synonymous	S173L	0.994	0	0.567
Ancient terminal	N2	T	11280	C	ND4	Non-synonymous	L174P	1	0.04	0.848
Ancient terminal	R0	A	11283	C	ND4	Non-synonymous	N175T	0.973	0.1	0.449
Modern terminal	R0	T	11286	C	ND4	Non-synonymous	I176T	0.999	0.1	0.336
Ancient terminal	U	T	11286	A	ND4	Non-synonymous	I176N	1	0	0.626
Modern terminal	U	T	11289	C	ND4	Non-synonymous	L177P	0.99	0.25	0.712
Ancient terminal	U	C	11309	A	ND4	Non-synonymous	Q184K	0.002	0.4	0.249
Pre-terminal	JT	C	11309	A	ND4	Non-synonymous	Q184K	0.002	0.4	0.249
Pre-terminal	R0	T	11318	G	ND4	Non-synonymous	S187A	0	0.87	0.069
Pre-terminal	R0	T	11318	A	ND4	Non-synonymous	S187T	0	0.84	0.04
Modern terminal	R0	A	11321	G	ND4	Non-synonymous	N188D	0	0.4	0.127
Pre-terminal	U	A	11322	G	ND4	Non-synonymous	N188S	0.048	0.89	0.106
Modern terminal	R0	T	11324	A	ND4	Non-synonymous	S189T	0.679	0.65	0.095
Pre-terminal	X	T	11324	A	ND4	Non-synonymous	S189T	0.679	0.65	0.095
Modern terminal	R0	A	11337	G	ND4	Non-synonymous	N193S	0.005	0.84	0.108
Modern terminal	R0	A	11337	G	ND4	Non-synonymous	N193S	0.005	0.84	0.108
Modern terminal	R0	A	11337	G	ND4	Non-synonymous	N193S	0.005	0.84	0.108
Pre-terminal	R0	A	11337	G	ND4	Non-synonymous	N193S	0.005	0.84	0.108
Pre-terminal	R0	A	11337	G	ND4	Non-synonymous	N193S	0.005	0.84	0.108
Ancient terminal	U	A	11337	G	ND4	Non-synonymous	N193S	0.005	0.84	0.108
Modern terminal	U	A	11337	G	ND4	Non-synonymous	N193S	0.005	0.84	0.108
Modern terminal	U	A	11337	G	ND4	Non-synonymous	N193S	0.005	0.84	0.108
Pre-terminal	U	A	11337	G	ND4	Non-synonymous	N193S	0.005	0.84	0.108
Pre-terminal	JT	A	11337	G	ND4	Non-synonymous	N193S	0.005	0.84	0.108
Modern terminal	N1	A	11337	G	ND4	Non-synonymous	N193S	0.005	0.84	0.108
Ancient terminal	N1	C	11352	A	ND4	Non-synonymous	A198D	1	0.01	0.675
Modern terminal	R0	A	11360	G	ND4	Non-synonymous	M201V	0.995	0.2	0.621
Pre-terminal	JT	A	11360	G	ND4	Non-synonymous	M201V	0.995	0.2	0.621
Modern terminal	R0	T	11361	C	ND4	Non-synonymous	M201T	0.985	0.09	0.664
Pre-terminal	R0	T	11361	C	ND4	Non-synonymous	M201T	0.985	0.09	0.664
Modern terminal	U	T	11361	C	ND4	Non-synonymous	M201T	0.985	0.09	0.664
Modern terminal	U	T	11361	C	ND4	Non-synonymous	M201T	0.985	0.09	0.664
Pre-terminal	U	T	11361	C	ND4	Non-synonymous	M201T	0.985	0.09	0.664
Modern terminal	X	T	11361	C	ND4	Non-synonymous	M201T	0.985	0.09	0.664
Modern terminal	R0	C	11384	A	ND4	Non-synonymous	L209I	0.994	0.18	0.25
Ancient terminal	U	C	11384	A	ND4	Non-synonymous	L209I	0.994	0.18	0.25
Ancient terminal	JT	G	11390	T	ND4	Non-synonymous	G211W	1	0	0.624
Ancient terminal	JT	T	11400	A	ND4	Nonsense				
Ancient terminal	U	A	11418	T	ND4	Non-synonymous	H220L	0.972	0	0.793
Pre-terminal	R0	A	11432	G	ND4	Non-synonymous	I225V	0.788	0.21	0.259
Ancient terminal	N1	A	11432	T	ND4	Non-synonymous	I225F	0.972	0	0.701
Ancient terminal	JT	G	11438	A	ND4	Nonsense				
Modern terminal	R0	G	11447	A	ND4	Non-synonymous	V230M	0.007	0	0.556

Modern terminal	R0	G	11447	A	ND4	Non-synonymous	V230M	0.007	0	0.556
Modern terminal	R0	G	11447	A	ND4	Non-synonymous	V230M	0.007	0	0.556
Modern terminal	R0	G	11447	A	ND4	Non-synonymous	V230M	0.007	0	0.556
Modern terminal	R0	G	11447	A	ND4	Non-synonymous	V230M	0.007	0	0.556
Pre-terminal	R0	G	11447	A	ND4	Non-synonymous	V230M	0.007	0	0.556
Modern terminal	U	G	11447	A	ND4	Non-synonymous	V230M	0.007	0	0.556
Modern terminal	U	G	11447	A	ND4	Non-synonymous	V230M	0.007	0	0.556
Modern terminal	U	G	11447	A	ND4	Non-synonymous	V230M	0.007	0	0.556
Modern terminal	U	G	11447	A	ND4	Non-synonymous	V230M	0.007	0	0.556
Pre-terminal	U	G	11447	A	ND4	Non-synonymous	V230M	0.007	0	0.556
Modern terminal	JT	G	11447	A	ND4	Non-synonymous	V230M	0.007	0	0.556
Modern terminal	JT	G	11447	A	ND4	Non-synonymous	V230M	0.007	0	0.556
Pre-terminal	JT	G	11447	A	ND4	Non-synonymous	V230M	0.007	0	0.556
Pre-terminal	N1	G	11447	A	ND4	Non-synonymous	V230M	0.007	0	0.556
Modern terminal	N2	G	11447	A	ND4	Non-synonymous	V230M	0.007	0	0.556
Pre-terminal	N2	G	11447	A	ND4	Non-synonymous	V230M	0.007	0	0.556
Ancient terminal	U	T	11448	C	ND4	Non-synonymous	V230A	0.683	0	0.672
Ancient terminal	JT	T	11451	A	ND4	Non-synonymous	L231H	1	0	0.78
Modern terminal	R0	G	11453	A	ND4	Non-synonymous	A232T	0.999	0	0.738
Modern terminal	R0	G	11453	A	ND4	Non-synonymous	A232T	0.999	0	0.738
Modern terminal	R0	G	11453	A	ND4	Non-synonymous	A232T	0.999	0	0.738
Modern terminal	R0	G	11453	A	ND4	Non-synonymous	A232T	0.999	0	0.738
Pre-terminal	R0	G	11453	A	ND4	Non-synonymous	A232T	0.999	0	0.738
Modern terminal	U	G	11453	A	ND4	Non-synonymous	A232T	0.999	0	0.738
Modern terminal	U	G	11453	A	ND4	Non-synonymous	A232T	0.999	0	0.738
Modern terminal	JT	G	11453	A	ND4	Non-synonymous	A232T	0.999	0	0.738
Ancient terminal	JT	G	11493	A	ND4	Non-synonymous	R245D	1	0	0.751
Modern terminal	R0	C	11499	T	ND4	Non-synonymous	T247M	0	0.14	0.59
Modern terminal	R0	C	11499	T	ND4	Non-synonymous	T247M	0	0.14	0.59
Pre-terminal	R0	C	11499	T	ND4	Non-synonymous	T247M	0	0.14	0.59
Modern terminal	U	C	11499	T	ND4	Non-synonymous	T247M	0	0.14	0.59
Modern terminal	U	C	11499	T	ND4	Non-synonymous	T247M	0	0.14	0.59
Pre-terminal	U	C	11499	T	ND4	Non-synonymous	T247M	0	0.14	0.59
Modern terminal	JT	C	11499	T	ND4	Non-synonymous	T247M	0	0.14	0.59
Ancient terminal	JT	T	11505	G	ND4	Non-synonymous	I249S	0.993	0.01	0.665
Ancient terminal	N1	C	11513	A	ND4	Non-synonymous	P252T	0.998	0.21	0.507
Modern terminal	R0	T	11517	C	ND4	Non-synonymous	L253P	1	0.05	0.778
Ancient terminal	JT	C	11525	G	ND4	Non-synonymous	H256D	0.999	0.28	0.422
Modern terminal	JT	T	11540	C	ND4	Non-synonymous	F261L	0.999	0.25	0.619
Ancient terminal	X	C	11543	T	ND4	Non-synonymous	L262F	0.893	0.01	0.603
Modern terminal	U	T	11544	C	ND4	Non-synonymous	L262P	1	0	0.889
Ancient terminal	R0	T	11547	C	ND4	Non-synonymous	V263A	0.002	0.47	0.385
Ancient terminal	N1	C	11553	T	ND4	Non-synonymous	S265F	1	0.02	0.7
Ancient terminal	U	A	11567	G	ND4	Non-synonymous	I270V	0.003	0.35	0.239
Modern terminal	U	T	11571	C	ND4	Non-synonymous	M271T	0.992	0	0.526
Modern terminal	R0	A	11576	G	ND4	Non-synonymous	S273G	1	0.29	0.533
Modern terminal	U	A	11582	G	ND4	Non-synonymous	I275V	0.916	0.03	0.149
Modern terminal	U	A	11582	G	ND4	Non-synonymous	I275V	0.916	0.03	0.149
Modern terminal	U	A	11582	G	ND4	Non-synonymous	I275V	0.916	0.03	0.149
Pre-terminal	U	A	11582	G	ND4	Non-synonymous	I275V	0.916	0.03	0.149

Ancient terminal	JT	G	11586	A	ND4	Non-synonymous	C276Y	0.821	0	0.719
Ancient terminal	R0	A	11608	C	ND4	Non-synonymous	K283N	1	0	0.268
Modern terminal	R0	A	11630	G	ND4	Non-synonymous	I291V	0	1	0.052
Modern terminal	U	G	11651	A	ND4	Non-synonymous	V298M	0.848	0.02	0.179
Ancient terminal	N1	G	11651	A	ND4	Non-synonymous	V298M	0.848	0.02	0.179
Modern terminal	R0	A	11654	G	ND4	Non-synonymous	T299A	0	1	0.213
Modern terminal	R0	A	11654	G	ND4	Non-synonymous	T299A	0	1	0.213
Modern terminal	U	A	11654	G	ND4	Non-synonymous	T299A	0	1	0.213
Modern terminal	U	A	11654	G	ND4	Non-synonymous	T299A	0	1	0.213
Pre-terminal	JT	A	11654	G	ND4	Non-synonymous	T299A	0	1	0.213
Pre-terminal	N1	A	11654	G	ND4	Non-synonymous	T299A	0	1	0.213
Ancient terminal	JT	G	11657	T	ND4	Non-synonymous	A300S	0.97	0.12	0.354
Modern terminal	JT	T	11685	A	ND4	Non-synonymous	F309Y	0.976	0.5	0.307
Pre-terminal	JT	T	11685	C	ND4	Non-synonymous	F309S	1	0.01	0.774
Modern terminal	U	G	11696	A	ND4	Non-synonymous	V313I	0	0.41	0.061
Modern terminal	U	G	11696	A	ND4	Non-synonymous	V313I	0	0.41	0.061
Pre-terminal	U	G	11696	A	ND4	Non-synonymous	V313I	0	0.41	0.061
Pre-terminal	N1	G	11696	A	ND4	Non-synonymous	V313I	0	0.41	0.061
Modern terminal	X	G	11696	A	ND4	Non-synonymous	V313I	0	0.41	0.061
Modern terminal	X	G	11696	A	ND4	Non-synonymous	V313I	0	0.41	0.061
Ancient terminal	JT	A	11699	G	ND4	Non-synonymous	I314V	0	1	0.104
Modern terminal	N1	A	11699	G	ND4	Non-synonymous	I314V	0	1	0.104
Modern terminal	U	A	11708	G	ND4	Non-synonymous	I317V	0.003	0.07	0.25
Modern terminal	JT	A	11708	G	ND4	Non-synonymous	I317V	0.003	0.07	0.25
Ancient terminal	R0	C	11730	T	ND4	Non-synonymous	S324L	0.999	0	0.537
Modern terminal	U	T	11733	C	ND4	Non-synonymous	L325S	1	0.48	0.723
Modern terminal	R0	C	11748	T	ND4	Non-synonymous	A330V	0.999	0	0.357
Modern terminal	R0	C	11752	G	ND4	Non-synonymous	N331K	0.999	0.03	0.4
Modern terminal	N2	C	11752	G	ND4	Non-synonymous	N331K	0.999	0.03	0.4
Modern terminal	U	C	11765	A	ND4	Non-synonymous	R336S	1	0	0.516
Modern terminal	R0	G	11778	A	ND4	Non-synonymous	R340H	0.999	0	0.494
Modern terminal	R0	G	11778	A	ND4	Non-synonymous	R340H	0.999	0	0.494
Modern terminal	R0	G	11778	A	ND4	Non-synonymous	R340H	0.999	0	0.494
Modern terminal	R0	G	11778	A	ND4	Non-synonymous	R340H	0.999	0	0.494
Modern terminal	R0	G	11778	A	ND4	Non-synonymous	R340H	0.999	0	0.494
Modern terminal	R0	G	11778	A	ND4	Non-synonymous	R340H	0.999	0	0.494
Modern terminal	R0	G	11778	A	ND4	Non-synonymous	R340H	0.999	0	0.494
Modern terminal	R0	G	11778	A	ND4	Non-synonymous	R340H	0.999	0	0.494
Modern terminal	R0	G	11778	A	ND4	Non-synonymous	R340H	0.999	0	0.494
Modern terminal	R0	G	11778	A	ND4	Non-synonymous	R340H	0.999	0	0.494
Modern terminal	R0	G	11778	A	ND4	Non-synonymous	R340H	0.999	0	0.494
Modern terminal	R0	G	11778	A	ND4	Non-synonymous	R340H	0.999	0	0.494
Modern terminal	R0	G	11778	A	ND4	Non-synonymous	R340H	0.999	0	0.494
Modern terminal	R0	G	11778	A	ND4	Non-synonymous	R340H	0.999	0	0.494
Modern terminal	R0	G	11778	A	ND4	Non-synonymous	R340H	0.999	0	0.494
Modern terminal	R0	G	11778	A	ND4	Non-synonymous	R340H	0.999	0	0.494
Modern terminal	R0	G	11778	A	ND4	Non-synonymous	R340H	0.999	0	0.494
Modern terminal	R0	G	11778	A	ND4	Non-synonymous	R340H	0.999	0	0.494
Modern terminal	R0	G	11778	A	ND4	Non-synonymous	R340H	0.999	0	0.494
Modern terminal	R0	G	11778	A	ND4	Non-synonymous	R340H	0.999	0	0.494

Modern terminal	R0	G	11778	A	ND4	Non-synonymous	R340H	0.999	0	0.494
Modern terminal	R0	G	11778	A	ND4	Non-synonymous	R340H	0.999	0	0.494
Modern terminal	R0	G	11778	A	ND4	Non-synonymous	R340H	0.999	0	0.494
Modern terminal	R0	G	11778	A	ND4	Non-synonymous	R340H	0.999	0	0.494
Modern terminal	R0	G	11778	A	ND4	Non-synonymous	R340H	0.999	0	0.494
Pre-terminal	R0	G	11778	A	ND4	Non-synonymous	R340H	0.999	0	0.494
Modern terminal	U	G	11778	A	ND4	Non-synonymous	R340H	0.999	0	0.494
Modern terminal	U	G	11778	A	ND4	Non-synonymous	R340H	0.999	0	0.494
Modern terminal	U	G	11778	A	ND4	Non-synonymous	R340H	0.999	0	0.494
Modern terminal	U	G	11778	A	ND4	Non-synonymous	R340H	0.999	0	0.494
Modern terminal	U	G	11778	A	ND4	Non-synonymous	R340H	0.999	0	0.494
Modern terminal	U	G	11778	A	ND4	Non-synonymous	R340H	0.999	0	0.494
Modern terminal	U	G	11778	A	ND4	Non-synonymous	R340H	0.999	0	0.494
Modern terminal	U	G	11778	A	ND4	Non-synonymous	R340H	0.999	0	0.494
Modern terminal	U	G	11778	A	ND4	Non-synonymous	R340H	0.999	0	0.494
Modern terminal	U	G	11778	A	ND4	Non-synonymous	R340H	0.999	0	0.494
Modern terminal	U	G	11778	A	ND4	Non-synonymous	R340H	0.999	0	0.494
Modern terminal	U	G	11778	A	ND4	Non-synonymous	R340H	0.999	0	0.494
Modern terminal	U	G	11778	A	ND4	Non-synonymous	R340H	0.999	0	0.494
Modern terminal	U	G	11778	A	ND4	Non-synonymous	R340H	0.999	0	0.494
Modern terminal	U	G	11778	A	ND4	Non-synonymous	R340H	0.999	0	0.494
Modern terminal	U	G	11778	A	ND4	Non-synonymous	R340H	0.999	0	0.494
Modern terminal	U	G	11778	A	ND4	Non-synonymous	R340H	0.999	0	0.494
Modern terminal	U	G	11778	A	ND4	Non-synonymous	R340H	0.999	0	0.494
Modern terminal	U	G	11778	A	ND4	Non-synonymous	R340H	0.999	0	0.494
Modern terminal	U	G	11778	A	ND4	Non-synonymous	R340H	0.999	0	0.494
Modern terminal	U	G	11778	A	ND4	Non-synonymous	R340H	0.999	0	0.494
Modern terminal	U	G	11778	A	ND4	Non-synonymous	R340H	0.999	0	0.494
Modern terminal	U	G	11778	A	ND4	Non-synonymous	R340H	0.999	0	0.494
Modern terminal	U	G	11778	A	ND4	Non-synonymous	R340H	0.999	0	0.494
Pre-terminal	U	G	11778	A	ND4	Non-synonymous	R340H	0.999	0	0.494
Pre-terminal	U	G	11778	A	ND4	Non-synonymous	R340H	0.999	0	0.494
Pre-terminal	U	G	11778	A	ND4	Non-synonymous	R340H	0.999	0	0.494
Modern terminal	JT	G	11778	A	ND4	Non-synonymous	R340H	0.999	0	0.494
Modern terminal	JT	G	11778	A	ND4	Non-synonymous	R340H	0.999	0	0.494
Modern terminal	JT	G	11778	A	ND4	Non-synonymous	R340H	0.999	0	0.494
Modern terminal	JT	G	11778	A	ND4	Non-synonymous	R340H	0.999	0	0.494
Modern terminal	JT	G	11778	A	ND4	Non-synonymous	R340H	0.999	0	0.494
Modern terminal	JT	G	11778	A	ND4	Non-synonymous	R340H	0.999	0	0.494
Modern terminal	JT	G	11778	A	ND4	Non-synonymous	R340H	0.999	0	0.494
Modern terminal	JT	G	11778	A	ND4	Non-synonymous	R340H	0.999	0	0.494
Modern terminal	JT	G	11778	A	ND4	Non-synonymous	R340H	0.999	0	0.494
Modern terminal	JT	G	11778	A	ND4	Non-synonymous	R340H	0.999	0	0.494
Modern terminal	JT	G	11778	A	ND4	Non-synonymous	R340H	0.999	0	0.494
Modern terminal	JT	G	11778	A	ND4	Non-synonymous	R340H	0.999	0	0.494
Modern terminal	JT	G	11778	A	ND4	Non-synonymous	R340H	0.999	0	0.494
Modern terminal	JT	G	11778	A	ND4	Non-synonymous	R340H	0.999	0	0.494
Modern terminal	JT	G	11778	A	ND4	Non-synonymous	R340H	0.999	0	0.494
Modern terminal	JT	G	11778	A	ND4	Non-synonymous	R340H	0.999	0	0.494
Modern terminal	JT	G	11778	A	ND4	Non-synonymous	R340H	0.999	0	0.494

Modern terminal	JT	G	11778	A	ND4	Non-synonymous	R340H	0.999	0	0.494
Modern terminal	JT	G	11778	A	ND4	Non-synonymous	R340H	0.999	0	0.494
Modern terminal	JT	G	11778	A	ND4	Non-synonymous	R340H	0.999	0	0.494
Modern terminal	JT	G	11778	A	ND4	Non-synonymous	R340H	0.999	0	0.494
Modern terminal	JT	G	11778	A	ND4	Non-synonymous	R340H	0.999	0	0.494
Modern terminal	JT	G	11778	A	ND4	Non-synonymous	R340H	0.999	0	0.494
Pre-terminal	JT	G	11778	A	ND4	Non-synonymous	R340H	0.999	0	0.494
Pre-terminal	JT	G	11778	A	ND4	Non-synonymous	R340H	0.999	0	0.494
Pre-terminal	JT	G	11778	A	ND4	Non-synonymous	R340H	0.999	0	0.494
Pre-terminal	JT	G	11778	A	ND4	Non-synonymous	R340H	0.999	0	0.494
Modern terminal	N1	G	11778	A	ND4	Non-synonymous	R340H	0.999	0	0.494
Modern terminal	N1	G	11778	A	ND4	Non-synonymous	R340H	0.999	0	0.494
Modern terminal	N1	G	11778	A	ND4	Non-synonymous	R340H	0.999	0	0.494
Modern terminal	N1	G	11778	A	ND4	Non-synonymous	R340H	0.999	0	0.494
Pre-terminal	N1	G	11778	A	ND4	Non-synonymous	R340H	0.999	0	0.494
Modern terminal	X	G	11778	A	ND4	Non-synonymous	R340H	0.999	0	0.494
Pre-terminal	X	G	11778	A	ND4	Non-synonymous	R340H	0.999	0	0.494
Modern terminal	N2	G	11778	A	ND4	Non-synonymous	R340H	0.999	0	0.494
Modern terminal	N2	G	11778	A	ND4	Non-synonymous	R340H	0.999	0	0.494
Modern terminal	JT	T	11792	A	ND4	Non-synonymous	S345T	0.78	0.67	0.188
Pre-terminal	R0	C	11804	T	ND4	Nonsense				
Modern terminal	R0	A	11807	G	ND4	Non-synonymous	T350A	0	0.45	0.244
Modern terminal	R0	A	11807	G	ND4	Non-synonymous	T350A	0	0.45	0.244
Modern terminal	R0	G	11825	A	ND4	Non-synonymous	A356T	0.002	0.71	0.364
Modern terminal	R0	G	11852	A	ND4	Non-synonymous	A365T	0.003	0.99	0.054
Modern terminal	JT	G	11852	A	ND4	Non-synonymous	A365T	0.003	0.99	0.054
Pre-terminal	X	G	11852	A	ND4	Non-synonymous	A365T	0.003	0.99	0.054
Ancient terminal	X	G	11861	A	ND4	Non-synonymous	A368T	0.999	0	0.573
Modern terminal	U	T	11864	G	ND4	Non-synonymous	L369V	0.988	0	0.564
Modern terminal	N1	C	11898	T	ND4	Non-synonymous	S380F	0.003	0.51	0.065
Modern terminal	R0	G	11906	A	ND4	Non-synonymous	V383M	0.002	0.24	0.104
Modern terminal	U	C	11919	T	ND4	Non-synonymous	S387F	0.999	0.01	0.763
Modern terminal	U	A	11928	G	ND4	Non-synonymous	N390S	0.995	0.49	0.306
Modern terminal	JT	A	11928	G	ND4	Non-synonymous	N390S	0.995	0.49	0.306
Modern terminal	JT	A	11928	G	ND4	Non-synonymous	N390S	0.995	0.49	0.306
Modern terminal	X	A	11928	G	ND4	Non-synonymous	N390S	0.995	0.49	0.306
Modern terminal	R0	A	11930	G	ND4	Non-synonymous	I391V	0.002	0.48	0.216
Modern terminal	R0	A	11930	G	ND4	Non-synonymous	I391V	0.002	0.48	0.216
Modern terminal	R0	A	11930	G	ND4	Non-synonymous	I391V	0.002	0.48	0.216
Pre-terminal	N1	A	11930	G	ND4	Non-synonymous	I391V	0.002	0.48	0.216
Modern terminal	U	A	11933	G	ND4	Non-synonymous	T392A	0.99	0.01	0.531
Modern terminal	JT	A	11933	T	ND4	Non-synonymous	T392S	0.99	0.25	0.366
Modern terminal	JT	A	11933	T	ND4	Non-synonymous	T392S	0.99	0.25	0.366
Modern terminal	N2	C	11936	G	ND4	Non-synonymous	L393V	0.002	0.08	0.39
Ancient terminal	R0	G	11948	T	ND4	Non-synonymous	G397W	1	0	0.875
Ancient terminal	JT	T	11952	G	ND4	Non-synonymous	L398R	0.375	0.03	0.857
Modern terminal	R0	A	11957	G	ND4	Non-synonymous	M400V	0.025	1	0.645
Ancient terminal	R0	A	11959	T	ND4	Non-synonymous	M400I	0.002	0.63	0.45
Ancient terminal	R0	G	11963	A	ND4	Non-synonymous	V402I	0	1	0.044
Modern terminal	R0	G	11963	A	ND4	Non-synonymous	V402I	0	1	0.044

Modern terminal	R0	G	11963	A	ND4	Non-synonymous	V402I	0	1	0.044
Modern terminal	R0	G	11963	A	ND4	Non-synonymous	V402I	0	1	0.044
Pre-terminal	R0	G	11963	C	ND4	Non-synonymous	V402L	0	0.26	0.348
Ancient terminal	U	G	11963	A	ND4	Non-synonymous	V402I	0	1	0.044
Modern terminal	U	G	11963	A	ND4	Non-synonymous	V402I	0	1	0.044
Modern terminal	U	G	11963	A	ND4	Non-synonymous	V402I	0	1	0.044
Modern terminal	U	G	11963	A	ND4	Non-synonymous	V402I	0	1	0.044
Modern terminal	U	G	11963	A	ND4	Non-synonymous	V402I	0	1	0.044
Modern terminal	U	G	11963	A	ND4	Non-synonymous	V402I	0	1	0.044
Modern terminal	U	G	11963	A	ND4	Non-synonymous	V402I	0	1	0.044
Pre-terminal	U	G	11963	A	ND4	Non-synonymous	V402I	0	1	0.044
Pre-terminal	U	G	11963	A	ND4	Non-synonymous	V402I	0	1	0.044
Modern terminal	JT	G	11963	A	ND4	Non-synonymous	V402I	0	1	0.044
Pre-terminal	JT	G	11963	A	ND4	Non-synonymous	V402I	0	1	0.044
Modern terminal	N2	G	11963	A	ND4	Non-synonymous	V402I	0	1	0.044
Pre-terminal	N2	G	11963	A	ND4	Non-synonymous	V402I	0	1	0.044
Ancient terminal	U	A	11966	T	ND4	Non-synonymous	T403S	0.99	0.15	0.459
Ancient terminal	R0	G	11969	A	ND4	Non-synonymous	A404T	0.001	0.13	0.54
Modern terminal	R0	G	11969	A	ND4	Non-synonymous	A404T	0.001	0.13	0.54
Modern terminal	R0	G	11969	A	ND4	Non-synonymous	A404T	0.001	0.13	0.54
Modern terminal	R0	G	11969	A	ND4	Non-synonymous	A404T	0.001	0.13	0.54
Modern terminal	R0	G	11969	A	ND4	Non-synonymous	A404T	0.001	0.13	0.54
Pre-terminal	R0	G	11969	A	ND4	Non-synonymous	A404T	0.001	0.13	0.54
Pre-terminal	R0	G	11969	A	ND4	Non-synonymous	A404T	0.001	0.13	0.54
Pre-terminal	R0	G	11969	A	ND4	Non-synonymous	A404T	0.001	0.13	0.54
Pre-terminal	R0	G	11969	A	ND4	Non-synonymous	A404T	0.001	0.13	0.54
Pre-terminal	R0	G	11969	A	ND4	Non-synonymous	A404T	0.001	0.13	0.54
Modern terminal	U	G	11969	A	ND4	Non-synonymous	A404T	0.001	0.13	0.54
Modern terminal	U	G	11969	A	ND4	Non-synonymous	A404T	0.001	0.13	0.54
Modern terminal	U	G	11969	A	ND4	Non-synonymous	A404T	0.001	0.13	0.54
Modern terminal	U	G	11969	A	ND4	Non-synonymous	A404T	0.001	0.13	0.54
Modern terminal	U	G	11969	A	ND4	Non-synonymous	A404T	0.001	0.13	0.54
Modern terminal	JT	G	11969	A	ND4	Non-synonymous	A404T	0.001	0.13	0.54
Modern terminal	JT	G	11969	A	ND4	Non-synonymous	A404T	0.001	0.13	0.54
Pre-terminal	JT	G	11969	A	ND4	Non-synonymous	A404T	0.001	0.13	0.54
Ancient terminal	JT	C	11970	A	ND4	Non-synonymous	A404D	0.685	0	0.783
Pre-terminal	R0	T	11978	A	ND4	Non-synonymous	S407T	0.827	0.12	0.559
Modern terminal	U	C	11981	T	ND4	Non-synonymous	L408F	0.998	0.02	0.59
Modern terminal	R0	T	11984	C	ND4	Non-synonymous	Y409H	1	0.25	0.448
Pre-terminal	JT	T	11984	C	ND4	Non-synonymous	Y409H	1	0.25	0.448
Ancient terminal	U	T	12005	A	ND4	Nonsense				
Modern terminal	U	T	12005	C	ND4	Non-synonymous	W416R	0.002	1	0.061
Modern terminal	R0	T	12011	C	ND4	Non-synonymous	S418P	0	0.32	0.272
Ancient terminal	U	T	12011	A	ND4	Non-synonymous	S418T	0	0.55	0.102
Modern terminal	U	T	12011	C	ND4	Non-synonymous	S418P	0	0.32	0.272
Pre-terminal	U	T	12011	C	ND4	Non-synonymous	S418P	0	0.32	0.272
Pre-terminal	U	T	12011	C	ND4	Non-synonymous	S418P	0	0.32	0.272
Modern terminal	JT	T	12011	G	ND4	Non-synonymous	S418A	0	0.36	0.099
Modern terminal	N1	T	12011	C	ND4	Non-synonymous	S418P	0	0.32	0.272
Pre-terminal	X	T	12011	C	ND4	Non-synonymous	S418P	0	0.32	0.272

Modern terminal	R0	C	12014	T	ND4	Non-synonymous	L419F	0.733	0.73	0.255
Modern terminal	R0	C	12014	T	ND4	Non-synonymous	L419F	0.733	0.73	0.255
Pre-terminal	R0	C	12014	T	ND4	Non-synonymous	L419F	0.733	0.73	0.255
Pre-terminal	R0	C	12014	T	ND4	Non-synonymous	L419F	0.733	0.73	0.255
Pre-terminal	U	C	12014	T	ND4	Non-synonymous	L419F	0.733	0.73	0.255
Pre-terminal	R0	T	12015	C	ND4	Non-synonymous	L419P	0.025	0.26	0.616
Modern terminal	U	T	12015	C	ND4	Non-synonymous	L419P	0.025	0.26	0.616
Ancient terminal	U	A	12017	G	ND4	Non-synonymous	T420A	0.035	0.16	0.384
Modern terminal	U	A	12017	G	ND4	Non-synonymous	T420A	0.035	0.16	0.384
Pre-terminal	U	A	12017	G	ND4	Non-synonymous	T420A	0.035	0.16	0.384
Pre-terminal	JT	C	12020	T	ND4	Non-synonymous	H421Y	0.846	0.76	0.236
Modern terminal	U	A	12021	C	ND4	Non-synonymous	H421P	0.999	0.15	0.612
Pre-terminal	R0	A	12026	G	ND4	Non-synonymous	I423V	0.003	0.12	0.142
Pre-terminal	R0	A	12026	G	ND4	Non-synonymous	I423V	0.003	0.12	0.142
Pre-terminal	N1	A	12026	G	ND4	Non-synonymous	I423V	0.003	0.12	0.142
Modern terminal	U	T	12027	C	ND4	Non-synonymous	I423T	0.004	0.24	0.281
Modern terminal	JT	T	12027	C	ND4	Non-synonymous	I423T	0.004	0.24	0.281
Modern terminal	R0	A	12030	T	ND4	Non-synonymous	N424I	0.005	0.41	0.218
Pre-terminal	R0	A	12030	G	ND4	Non-synonymous	N424S	0.003	0.43	0.141
Modern terminal	U	A	12030	G	ND4	Non-synonymous	N424S	0.003	0.43	0.141
Modern terminal	U	A	12030	G	ND4	Non-synonymous	N424S	0.003	0.43	0.141
Modern terminal	JT	A	12030	G	ND4	Non-synonymous	N424S	0.003	0.43	0.141
Modern terminal	JT	A	12030	G	ND4	Non-synonymous	N424S	0.003	0.43	0.141
Modern terminal	JT	A	12030	G	ND4	Non-synonymous	N424S	0.003	0.43	0.141
Pre-terminal	JT	A	12030	G	ND4	Non-synonymous	N424S	0.003	0.43	0.141
Modern terminal	X	A	12030	G	ND4	Non-synonymous	N424S	0.003	0.43	0.141
Modern terminal	U	C	12031	A	ND4	Non-synonymous	N424K	0	0.34	0.172
Modern terminal	R0	A	12033	G	ND4	Non-synonymous	N425S	0.985	0.67	0.246
Modern terminal	R0	A	12033	G	ND4	Non-synonymous	N425S	0.985	0.67	0.246
Modern terminal	R0	A	12033	G	ND4	Non-synonymous	N425S	0.985	0.67	0.246
Modern terminal	R0	A	12033	G	ND4	Non-synonymous	N425S	0.985	0.67	0.246
Modern terminal	R0	A	12033	G	ND4	Non-synonymous	N425S	0.985	0.67	0.246
Pre-terminal	R0	A	12033	G	ND4	Non-synonymous	N425S	0.985	0.67	0.246
Pre-terminal	R0	A	12033	G	ND4	Non-synonymous	N425S	0.985	0.67	0.246
Modern terminal	U	T	12048	C	ND4	Non-synonymous	F430S	0.001	0.35	0.628
Modern terminal	R0	A	12059	C	ND4	Non-synonymous	N434H	0.999	1	0.22
Modern terminal	R0	A	12059	C	ND4	Non-synonymous	N434H	0.999	1	0.22
Modern terminal	R0	A	12059	C	ND4	Non-synonymous	N434H	0.999	1	0.22
Pre-terminal	R0	A	12059	C	ND4	Non-synonymous	N434H	0.999	1	0.22
Pre-terminal	R0	A	12059	C	ND4	Non-synonymous	N434H	0.999	1	0.22
Ancient terminal	R0	C	12063	T	ND4	Non-synonymous	T435I	0	0.24	0.212
Pre-terminal	R0	C	12063	T	ND4	Non-synonymous	T435I	0	0.24	0.212
Pre-terminal	R0	C	12063	T	ND4	Non-synonymous	T435I	0	0.24	0.212
Modern terminal	U	C	12063	T	ND4	Non-synonymous	T435I	0	0.24	0.212
Pre-terminal	U	C	12063	T	ND4	Non-synonymous	T435I	0	0.24	0.212
Modern terminal	JT	C	12063	T	ND4	Non-synonymous	T435I	0	0.24	0.212
Modern terminal	X	C	12063	T	ND4	Non-synonymous	T435I	0	0.24	0.212
Modern terminal	N2	C	12063	T	ND4	Non-synonymous	T435I	0	0.24	0.212
Modern terminal	U	C	12065	T	ND4	Non-synonymous	L436F	0.998	0.04	0.404
Modern terminal	U	T	12071	A	ND4	Non-synonymous	F438I	0.063	0.37	0.284

Modern terminal	U	A	12074	C	ND4	Non-synonymous	M439L	0.239	1	0.076
Modern terminal	JT	A	12074	C	ND4	Non-synonymous	M439L	0.239	1	0.076
Ancient terminal	R0	C	12080	G	ND4	Non-synonymous	L441V	0.975	0.07	0.19
Ancient terminal	U	T	12081	A	ND4	Non-synonymous	L441Q	1	0	0.501
Modern terminal	R0	T	12083	G	ND4	Non-synonymous	S442A	0.004	0.16	0.062
Modern terminal	U	T	12083	G	ND4	Non-synonymous	S442A	0.004	0.16	0.062
Pre-terminal	JT	T	12083	G	ND4	Non-synonymous	S442A	0.004	0.16	0.062
Pre-terminal	N1	T	12083	G	ND4	Non-synonymous	S442A	0.004	0.16	0.062
Modern terminal	R0	C	12084	T	ND4	Non-synonymous	S442F	0.001	0.33	0.149
Modern terminal	R0	C	12084	T	ND4	Non-synonymous	S442F	0.001	0.33	0.149
Modern terminal	JT	C	12084	T	ND4	Non-synonymous	S442F	0.001	0.33	0.149
Modern terminal	N1	C	12084	T	ND4	Non-synonymous	S442F	0.001	0.33	0.149
Ancient terminal	R0	A	12089	T	ND4	Non-synonymous	I444F	0.141	0.01	0.246
Modern terminal	R0	T	12090	C	ND4	Non-synonymous	I444T	0.861	0.01	0.17
Ancient terminal	X	T	12090	C	ND4	Non-synonymous	I444T	0.861	0.01	0.17
Modern terminal	R0	C	12092	A	ND4	Non-synonymous	L445I	0.987	0.92	0.062
Modern terminal	R0	C	12092	A	ND4	Non-synonymous	L445I	0.987	0.92	0.062
Modern terminal	R0	C	12092	A	ND4	Non-synonymous	L445I	0.987	0.92	0.062
Modern terminal	U	C	12092	T	ND4	Non-synonymous	L445F	0.998	0.01	0.162
Pre-terminal	U	C	12092	T	ND4	Non-synonymous	L445F	0.998	0.01	0.162
Pre-terminal	U	C	12092	T	ND4	Non-synonymous	L445F	0.998	0.01	0.162
Modern terminal	JT	C	12092	A	ND4	Non-synonymous	L445I	0.987	0.92	0.062
Modern terminal	JT	C	12092	A	ND4	Non-synonymous	L445I	0.987	0.92	0.062
Modern terminal	JT	C	12092	T	ND4	Non-synonymous	L445F	0.998	0.01	0.162
Modern terminal	R0	C	12098	G	ND4	Non-synonymous	L447V	0.975	0.01	0.317
Pre-terminal	N1	C	12104	A	ND4	Non-synonymous	L449I	0.009	0.25	0.115
Modern terminal	JT	C	12110	T	ND4	Non-synonymous	P451S	1	0	0.241
Modern terminal	R0	G	12113	A	ND4	Non-synonymous	D452N	0	0.23	0.046
Modern terminal	R0	A	12119	C	ND4	Non-synonymous	I454L	0.598	0.09	0.204
Modern terminal	R0	C	12123	T	ND4	Non-synonymous	T455I	0.349	0.23	0.131
Modern terminal	R0	C	12123	T	ND4	Non-synonymous	T455I	0.349	0.23	0.131
Pre-terminal	R0	C	12123	T	ND4	Non-synonymous	T455I	0.349	0.23	0.131
Modern terminal	U	C	12123	T	ND4	Non-synonymous	T455I	0.349	0.23	0.131
Modern terminal	U	C	12123	T	ND4	Non-synonymous	T455I	0.349	0.23	0.131
Modern terminal	JT	C	12123	T	ND4	Non-synonymous	T455I	0.349	0.23	0.131
Pre-terminal	JT	C	12123	T	ND4	Non-synonymous	T455I	0.349	0.23	0.131
Pre-terminal	JT	C	12123	T	ND4	Non-synonymous	T455I	0.349	0.23	0.131
Pre-terminal	JT	C	12123	T	ND4	Non-synonymous	T455I	0.349	0.23	0.131
Pre-terminal	JT	C	12123	T	ND4	Non-synonymous	T455I	0.349	0.23	0.131
Modern terminal	R0	G	12126	A	ND4	Non-synonymous	G456E	1	0	0.548
Modern terminal	U	T	12128	C	ND4	Non-synonymous	F457L	0.004	1	0.173
Modern terminal	JT	T	12128	C	ND4	Non-synonymous	F457L	0.004	1	0.173
Modern terminal	R0	T	12134	C	ND4	Non-synonymous	S459P	0.29	0.14	0.494
Modern terminal	R0	T	12134	A	ND4	Non-synonymous	S459T	0.004	0.38	0.201
Modern terminal	U	T	12134	C	ND4	Non-synonymous	S459P	0.29	0.14	0.494
Pre-terminal	U	T	12134	C	ND4	Non-synonymous	S459P	0.29	0.14	0.494
Modern terminal	JT	T	12134	C	ND4	Non-synonymous	S459P	0.29	0.14	0.494
Pre-terminal	JT	T	12134	C	ND4	Non-synonymous	S459P	0.29	0.14	0.494
Modern terminal	N1	T	12134	C	ND4	Non-synonymous	S459P	0.29	0.14	0.494
Modern terminal	N1	T	12134	C	ND4	Non-synonymous	S459P	0.29	0.14	0.494

Modern terminal	U	C	12135	A	ND4	Non-synonymous	S459Y	0.843	0.08	0.443
Modern terminal	U	C	12135	A	ND4	Non-synonymous	S459Y	0.843	0.08	0.443
Pre-terminal	U	A	12337	G	ND5	Non-synonymous	M1V	*	0	0.643
Ancient terminal	JT	A	12337	G	ND5	Non-synonymous	M1V	*	0	0.643
Modern terminal	R0	T	12338	C	ND5	Non-synonymous	M1T	*	0	0.707
Pre-terminal	U	T	12338	C	ND5	Non-synonymous	M1T	*	0	0.707
Modern terminal	R0	A	12340	G	ND5	Non-synonymous	T2A	*	0.89	0.183
Modern terminal	R0	A	12340	G	ND5	Non-synonymous	T2A	*	0.89	0.183
Modern terminal	JT	A	12340	G	ND5	Non-synonymous	T2A	*	0.89	0.183
Modern terminal	R0	C	12341	T	ND5	Non-synonymous	T2I	*	0.13	0.21
Modern terminal	R0	C	12341	A	ND5	Non-synonymous	T2N	*	1	0.274
Pre-terminal	R0	C	12341	T	ND5	Non-synonymous	T2I	*	0.13	0.21
Ancient terminal	R0	C	12346	T	ND5	Non-synonymous	H4Y	*	0.49	0.119
Modern terminal	R0	C	12346	T	ND5	Non-synonymous	H4Y	*	0.49	0.119
Modern terminal	R0	C	12346	T	ND5	Non-synonymous	H4Y	*	0.49	0.119
Pre-terminal	R0	C	12346	T	ND5	Non-synonymous	H4Y	*	0.49	0.119
Pre-terminal	R0	C	12346	T	ND5	Non-synonymous	H4Y	*	0.49	0.119
Pre-terminal	R0	C	12346	T	ND5	Non-synonymous	H4Y	*	0.49	0.119
Pre-terminal	R0	C	12346	T	ND5	Non-synonymous	H4Y	*	0.49	0.119
Ancient terminal	U	C	12346	T	ND5	Non-synonymous	H4Y	*	0.49	0.119
Modern terminal	U	C	12346	T	ND5	Non-synonymous	H4Y	*	0.49	0.119
Modern terminal	U	C	12346	T	ND5	Non-synonymous	H4Y	*	0.49	0.119
Modern terminal	U	C	12346	T	ND5	Non-synonymous	H4Y	*	0.49	0.119
Pre-terminal	U	C	12346	T	ND5	Non-synonymous	H4Y	*	0.49	0.119
Pre-terminal	U	C	12346	T	ND5	Non-synonymous	H4Y	*	0.49	0.119
Pre-terminal	U	C	12346	T	ND5	Non-synonymous	H4Y	*	0.49	0.119
Modern terminal	JT	C	12346	T	ND5	Non-synonymous	H4Y	*	0.49	0.119
Modern terminal	JT	C	12346	T	ND5	Non-synonymous	H4Y	*	0.49	0.119
Modern terminal	JT	C	12346	T	ND5	Non-synonymous	H4Y	*	0.49	0.119
Modern terminal	JT	C	12346	T	ND5	Non-synonymous	H4Y	*	0.49	0.119
Pre-terminal	JT	C	12346	T	ND5	Non-synonymous	H4Y	*	0.49	0.119
Modern terminal	N1	C	12346	T	ND5	Non-synonymous	H4Y	*	0.49	0.119
Modern terminal	R0	A	12349	G	ND5	Non-synonymous	T5A	*	0.07	0.152
Pre-terminal	R0	A	12349	G	ND5	Non-synonymous	T5A	*	0.07	0.152
Modern terminal	U	A	12352	G	ND5	Non-synonymous	T6A	*	0.04	0.177
Modern terminal	N1	A	12352	G	ND5	Non-synonymous	T6A	*	0.04	0.177
Modern terminal	R0	A	12358	G	ND5	Non-synonymous	T8A	*	0.38	0.163
Modern terminal	R0	A	12358	G	ND5	Non-synonymous	T8A	*	0.38	0.163
Modern terminal	R0	A	12358	G	ND5	Non-synonymous	T8A	*	0.38	0.163
Modern terminal	R0	A	12358	G	ND5	Non-synonymous	T8A	*	0.38	0.163
Modern terminal	R0	A	12358	G	ND5	Non-synonymous	T8A	*	0.38	0.163
Modern terminal	R0	A	12358	G	ND5	Non-synonymous	T8A	*	0.38	0.163
Modern terminal	R0	A	12358	G	ND5	Non-synonymous	T8A	*	0.38	0.163
Modern terminal	R0	A	12358	G	ND5	Non-synonymous	T8A	*	0.38	0.163
Modern terminal	R0	A	12358	G	ND5	Non-synonymous	T8A	*	0.38	0.163
Pre-terminal	R0	A	12358	G	ND5	Non-synonymous	T8A	*	0.38	0.163
Pre-terminal	R0	A	12358	G	ND5	Non-synonymous	T8A	*	0.38	0.163
Pre-terminal	R0	A	12358	G	ND5	Non-synonymous	T8A	*	0.38	0.163
Pre-terminal	R0	A	12358	G	ND5	Non-synonymous	T8A	*	0.38	0.163
Pre-terminal	R0	A	12358	G	ND5	Non-synonymous	T8A	*	0.38	0.163

Pre-terminal	R0	A	12358	G	ND5	Non-synonymous	T8A	*	0.38	0.163
Ancient terminal	U	A	12358	G	ND5	Non-synonymous	T8A	*	0.38	0.163
Ancient terminal	U	A	12358	G	ND5	Non-synonymous	T8A	*	0.38	0.163
Modern terminal	U	A	12358	G	ND5	Non-synonymous	T8A	*	0.38	0.163
Modern terminal	U	A	12358	G	ND5	Non-synonymous	T8A	*	0.38	0.163
Modern terminal	U	A	12358	G	ND5	Non-synonymous	T8A	*	0.38	0.163
Modern terminal	U	A	12358	G	ND5	Non-synonymous	T8A	*	0.38	0.163
Modern terminal	U	A	12358	G	ND5	Non-synonymous	T8A	*	0.38	0.163
Modern terminal	U	A	12358	G	ND5	Non-synonymous	T8A	*	0.38	0.163
Modern terminal	U	A	12358	G	ND5	Non-synonymous	T8A	*	0.38	0.163
Pre-terminal	U	A	12358	G	ND5	Non-synonymous	T8A	*	0.38	0.163
Pre-terminal	U	A	12358	G	ND5	Non-synonymous	T8A	*	0.38	0.163
Pre-terminal	U	A	12358	G	ND5	Non-synonymous	T8A	*	0.38	0.163
Pre-terminal	U	A	12358	G	ND5	Non-synonymous	T8A	*	0.38	0.163
Pre-terminal	U	A	12358	G	ND5	Non-synonymous	T8A	*	0.38	0.163
Pre-terminal	U	A	12358	G	ND5	Non-synonymous	T8A	*	0.38	0.163
Pre-terminal	U	A	12358	G	ND5	Non-synonymous	T8A	*	0.38	0.163
Pre-terminal	U	A	12358	G	ND5	Non-synonymous	T8A	*	0.38	0.163
Modern terminal	JT	A	12358	G	ND5	Non-synonymous	T8A	*	0.38	0.163
Modern terminal	JT	A	12358	G	ND5	Non-synonymous	T8A	*	0.38	0.163
Modern terminal	JT	A	12358	G	ND5	Non-synonymous	T8A	*	0.38	0.163
Modern terminal	JT	A	12358	G	ND5	Non-synonymous	T8A	*	0.38	0.163
Modern terminal	JT	A	12358	G	ND5	Non-synonymous	T8A	*	0.38	0.163
Modern terminal	JT	A	12358	G	ND5	Non-synonymous	T8A	*	0.38	0.163
Modern terminal	JT	A	12358	G	ND5	Non-synonymous	T8A	*	0.38	0.163
Modern terminal	JT	A	12358	G	ND5	Non-synonymous	T8A	*	0.38	0.163
Pre-terminal	JT	A	12358	G	ND5	Non-synonymous	T8A	*	0.38	0.163
Pre-terminal	JT	A	12358	G	ND5	Non-synonymous	T8A	*	0.38	0.163
Pre-terminal	JT	A	12358	G	ND5	Non-synonymous	T8A	*	0.38	0.163
Pre-terminal	N1	A	12358	G	ND5	Non-synonymous	T8A	*	0.38	0.163
Pre-terminal	N1	A	12358	G	ND5	Non-synonymous	T8A	*	0.38	0.163
Modern terminal	X	A	12358	G	ND5	Non-synonymous	T8A	*	0.38	0.163
Modern terminal	X	A	12358	G	ND5	Non-synonymous	T8A	*	0.38	0.163
Modern terminal	N2	A	12358	G	ND5	Non-synonymous	T8A	*	0.38	0.163
Modern terminal	N2	A	12358	G	ND5	Non-synonymous	T8A	*	0.38	0.163
Pre-terminal	N2	A	12358	G	ND5	Non-synonymous	T8A	*	0.38	0.163
Ancient terminal	R0	A	12361	G	ND5	Non-synonymous	T9A	*	0.44	0.113
Modern terminal	R0	A	12361	G	ND5	Non-synonymous	T9A	*	0.44	0.113
Modern terminal	R0	A	12361	G	ND5	Non-synonymous	T9A	*	0.44	0.113
Modern terminal	R0	A	12361	G	ND5	Non-synonymous	T9A	*	0.44	0.113
Pre-terminal	R0	A	12361	G	ND5	Non-synonymous	T9A	*	0.44	0.113
Modern terminal	U	A	12361	G	ND5	Non-synonymous	T9A	*	0.44	0.113
Modern terminal	U	A	12361	G	ND5	Non-synonymous	T9A	*	0.44	0.113
Modern terminal	U	A	12361	G	ND5	Non-synonymous	T9A	*	0.44	0.113
Modern terminal	U	A	12361	G	ND5	Non-synonymous	T9A	*	0.44	0.113
Modern terminal	U	A	12361	G	ND5	Non-synonymous	T9A	*	0.44	0.113
Modern terminal	U	A	12361	G	ND5	Non-synonymous	T9A	*	0.44	0.113
Pre-terminal	U	A	12361	G	ND5	Non-synonymous	T9A	*	0.44	0.113
Pre-terminal	U	A	12361	G	ND5	Non-synonymous	T9A	*	0.44	0.113
Modern terminal	JT	A	12361	G	ND5	Non-synonymous	T9A	*	0.44	0.113

Modern terminal	JT	A	12361	G	ND5	Non-synonymous	T9A	*	0.44	0.113
Modern terminal	JT	A	12361	G	ND5	Non-synonymous	T9A	*	0.44	0.113
Pre-terminal	JT	A	12361	G	ND5	Non-synonymous	T9A	*	0.44	0.113
Modern terminal	N1	A	12361	G	ND5	Non-synonymous	T9A	*	0.44	0.113
Pre-terminal	N1	A	12361	G	ND5	Non-synonymous	T9A	*	0.44	0.113
Modern terminal	R0	C	12362	T	ND5	Non-synonymous	T9I	*	0.57	0.121
Modern terminal	R0	C	12362	T	ND5	Non-synonymous	T9I	*	0.57	0.121
Modern terminal	R0	C	12362	T	ND5	Non-synonymous	T9I	*	0.57	0.121
Pre-terminal	R0	C	12362	T	ND5	Non-synonymous	T9I	*	0.57	0.121
Pre-terminal	R0	C	12362	T	ND5	Non-synonymous	T9I	*	0.57	0.121
Modern terminal	U	C	12362	T	ND5	Non-synonymous	T9I	*	0.57	0.121
Modern terminal	U	C	12362	T	ND5	Non-synonymous	T9I	*	0.57	0.121
Modern terminal	R0	A	12367	G	ND5	Non-synonymous	T11A	*	0.21	0.111
Modern terminal	U	A	12367	G	ND5	Non-synonymous	T11A	*	0.21	0.111
Modern terminal	R0	A	12373	G	ND5	Non-synonymous	T13A	*	0.43	0.064
Modern terminal	R0	A	12373	G	ND5	Non-synonymous	T13A	*	0.43	0.064
Modern terminal	R0	A	12373	G	ND5	Non-synonymous	T13A	*	0.43	0.064
Modern terminal	R0	A	12373	G	ND5	Non-synonymous	T13A	*	0.43	0.064
Modern terminal	R0	A	12373	G	ND5	Non-synonymous	T13A	*	0.43	0.064
Modern terminal	R0	A	12373	G	ND5	Non-synonymous	T13A	*	0.43	0.064
Modern terminal	R0	A	12373	G	ND5	Non-synonymous	T13A	*	0.43	0.064
Modern terminal	R0	A	12373	G	ND5	Non-synonymous	T13A	*	0.43	0.064
Modern terminal	R0	A	12373	G	ND5	Non-synonymous	T13A	*	0.43	0.064
Modern terminal	R0	A	12373	G	ND5	Non-synonymous	T13A	*	0.43	0.064
Pre-terminal	R0	A	12373	G	ND5	Non-synonymous	T13A	*	0.43	0.064
Pre-terminal	R0	A	12373	G	ND5	Non-synonymous	T13A	*	0.43	0.064
Pre-terminal	R0	A	12373	G	ND5	Non-synonymous	T13A	*	0.43	0.064
Pre-terminal	R0	A	12373	G	ND5	Non-synonymous	T13A	*	0.43	0.064
Modern terminal	U	A	12373	G	ND5	Non-synonymous	T13A	*	0.43	0.064
Modern terminal	U	A	12373	G	ND5	Non-synonymous	T13A	*	0.43	0.064
Modern terminal	U	A	12373	G	ND5	Non-synonymous	T13A	*	0.43	0.064
Modern terminal	U	A	12373	G	ND5	Non-synonymous	T13A	*	0.43	0.064
Modern terminal	U	A	12373	G	ND5	Non-synonymous	T13A	*	0.43	0.064
Modern terminal	U	A	12373	G	ND5	Non-synonymous	T13A	*	0.43	0.064
Modern terminal	U	A	12373	G	ND5	Non-synonymous	T13A	*	0.43	0.064
Modern terminal	U	A	12373	G	ND5	Non-synonymous	T13A	*	0.43	0.064
Pre-terminal	U	A	12373	G	ND5	Non-synonymous	T13A	*	0.43	0.064
Pre-terminal	U	A	12373	G	ND5	Non-synonymous	T13A	*	0.43	0.064
Pre-terminal	U	A	12373	G	ND5	Non-synonymous	T13A	*	0.43	0.064
Pre-terminal	U	A	12373	G	ND5	Non-synonymous	T13A	*	0.43	0.064
Pre-terminal	U	A	12373	G	ND5	Non-synonymous	T13A	*	0.43	0.064
Pre-terminal	U	A	12373	G	ND5	Non-synonymous	T13A	*	0.43	0.064
Modern terminal	JT	A	12373	G	ND5	Non-synonymous	T13A	*	0.43	0.064
Modern terminal	JT	A	12373	G	ND5	Non-synonymous	T13A	*	0.43	0.064
Modern terminal	JT	A	12373	G	ND5	Non-synonymous	T13A	*	0.43	0.064
Modern terminal	JT	A	12373	G	ND5	Non-synonymous	T13A	*	0.43	0.064
Modern terminal	N1	A	12373	G	ND5	Non-synonymous	T13A	*	0.43	0.064
Modern terminal	N2	A	12373	G	ND5	Non-synonymous	T13A	*	0.43	0.064
Pre-terminal	U	T	12376	G	ND5	Non-synonymous	S14A	*	0.16	0.086
Ancient terminal	JT	C	12386	A	ND5	Non-synonymous	P17H	*	0.51	0.142

Modern terminal	JT	C	12389	T	ND5	Non-synonymous	P18L	*	0.01	0.295
Ancient terminal	N2	C	12389	A	ND5	Non-synonymous	P18H	*	0	0.313
Modern terminal	R0	A	12397	G	ND5	Non-synonymous	T21A	*	0.8	0.073
Modern terminal	R0	A	12397	G	ND5	Non-synonymous	T21A	*	0.8	0.073
Modern terminal	R0	A	12397	G	ND5	Non-synonymous	T21A	*	0.8	0.073
Modern terminal	R0	A	12397	G	ND5	Non-synonymous	T21A	*	0.8	0.073
Modern terminal	R0	A	12397	G	ND5	Non-synonymous	T21A	*	0.8	0.073
Modern terminal	R0	A	12397	G	ND5	Non-synonymous	T21A	*	0.8	0.073
Modern terminal	R0	A	12397	G	ND5	Non-synonymous	T21A	*	0.8	0.073
Modern terminal	R0	A	12397	G	ND5	Non-synonymous	T21A	*	0.8	0.073
Modern terminal	R0	A	12397	G	ND5	Non-synonymous	T21A	*	0.8	0.073
Modern terminal	R0	A	12397	G	ND5	Non-synonymous	T21A	*	0.8	0.073
Modern terminal	R0	A	12397	G	ND5	Non-synonymous	T21A	*	0.8	0.073
Modern terminal	R0	A	12397	G	ND5	Non-synonymous	T21A	*	0.8	0.073
Modern terminal	R0	A	12397	G	ND5	Non-synonymous	T21A	*	0.8	0.073
Pre-terminal	R0	A	12397	G	ND5	Non-synonymous	T21A	*	0.8	0.073
Pre-terminal	R0	A	12397	G	ND5	Non-synonymous	T21A	*	0.8	0.073
Pre-terminal	R0	A	12397	G	ND5	Non-synonymous	T21A	*	0.8	0.073
Pre-terminal	R0	A	12397	G	ND5	Non-synonymous	T21A	*	0.8	0.073
Pre-terminal	R0	A	12397	G	ND5	Non-synonymous	T21A	*	0.8	0.073
Pre-terminal	R0	A	12397	G	ND5	Non-synonymous	T21A	*	0.8	0.073
Pre-terminal	R0	A	12397	G	ND5	Non-synonymous	T21A	*	0.8	0.073
Modern terminal	U	A	12397	G	ND5	Non-synonymous	T21A	*	0.8	0.073
Modern terminal	U	A	12397	G	ND5	Non-synonymous	T21A	*	0.8	0.073
Modern terminal	U	A	12397	G	ND5	Non-synonymous	T21A	*	0.8	0.073
Modern terminal	U	A	12397	G	ND5	Non-synonymous	T21A	*	0.8	0.073
Modern terminal	U	A	12397	G	ND5	Non-synonymous	T21A	*	0.8	0.073
Modern terminal	U	A	12397	G	ND5	Non-synonymous	T21A	*	0.8	0.073
Modern terminal	U	A	12397	G	ND5	Non-synonymous	T21A	*	0.8	0.073
Modern terminal	U	A	12397	G	ND5	Non-synonymous	T21A	*	0.8	0.073
Modern terminal	U	A	12397	G	ND5	Non-synonymous	T21A	*	0.8	0.073
Modern terminal	U	A	12397	G	ND5	Non-synonymous	T21A	*	0.8	0.073
Pre-terminal	U	A	12397	G	ND5	Non-synonymous	T21A	*	0.8	0.073
Pre-terminal	U	A	12397	G	ND5	Non-synonymous	T21A	*	0.8	0.073
Pre-terminal	U	A	12397	G	ND5	Non-synonymous	T21A	*	0.8	0.073
Modern terminal	JT	A	12397	G	ND5	Non-synonymous	T21A	*	0.8	0.073
Modern terminal	JT	A	12397	G	ND5	Non-synonymous	T21A	*	0.8	0.073
Pre-terminal	JT	A	12397	G	ND5	Non-synonymous	T21A	*	0.8	0.073
Pre-terminal	JT	A	12397	G	ND5	Non-synonymous	T21A	*	0.8	0.073
Pre-terminal	JT	A	12397	G	ND5	Non-synonymous	T21A	*	0.8	0.073
Pre-terminal	JT	A	12397	G	ND5	Non-synonymous	T21A	*	0.8	0.073
Modern terminal	N1	A	12397	G	ND5	Non-synonymous	T21A	*	0.8	0.073
Pre-terminal	N1	A	12397	G	ND5	Non-synonymous	T21A	*	0.8	0.073
Modern terminal	U	C	12398	T	ND5	Non-synonymous	T21I	*	0.38	0.138
Modern terminal	U	C	12398	T	ND5	Non-synonymous	T21I	*	0.38	0.138
Ancient terminal	U	A	12400	T	ND5	Non-synonymous	T22S	*	0.91	0.15
Modern terminal	N2	A	12400	G	ND5	Non-synonymous	T22A	*	0.12	0.145
Modern terminal	R0	C	12401	T	ND5	Non-synonymous	T22I	*	0.17	0.207
Pre-terminal	R0	C	12401	T	ND5	Non-synonymous	T22I	*	0.17	0.207
Ancient terminal	U	C	12403	T	ND5	Non-synonymous	L23F	*	0.83	0.217

Modern terminal	U	C	12403	T	ND5	Non-synonymous	L23F	*	0.83	0.217
Modern terminal	JT	C	12403	T	ND5	Non-synonymous	L23F	*	0.83	0.217
Ancient terminal	X	C	12403	T	ND5	Non-synonymous	L23F	*	0.83	0.217
Ancient terminal	R0	G	12406	A	ND5	Non-synonymous	V24I	0	0.61	0.026
Modern terminal	R0	G	12406	A	ND5	Non-synonymous	V24I	0	0.61	0.026
Modern terminal	R0	G	12406	A	ND5	Non-synonymous	V24I	0	0.61	0.026
Modern terminal	R0	G	12406	A	ND5	Non-synonymous	V24I	0	0.61	0.026
Modern terminal	R0	G	12406	A	ND5	Non-synonymous	V24I	0	0.61	0.026
Modern terminal	R0	G	12406	A	ND5	Non-synonymous	V24I	0	0.61	0.026
Modern terminal	R0	G	12406	A	ND5	Non-synonymous	V24I	0	0.61	0.026
Modern terminal	R0	G	12406	A	ND5	Non-synonymous	V24I	0	0.61	0.026
Modern terminal	R0	G	12406	A	ND5	Non-synonymous	V24I	0	0.61	0.026
Modern terminal	R0	G	12406	A	ND5	Non-synonymous	V24I	0	0.61	0.026
Modern terminal	R0	G	12406	A	ND5	Non-synonymous	V24I	0	0.61	0.026
Pre-terminal	R0	G	12406	A	ND5	Non-synonymous	V24I	0	0.61	0.026
Pre-terminal	R0	G	12406	A	ND5	Non-synonymous	V24I	0	0.61	0.026
Pre-terminal	R0	G	12406	A	ND5	Non-synonymous	V24I	0	0.61	0.026
Ancient terminal	U	G	12406	A	ND5	Non-synonymous	V24I	0	0.61	0.026
Modern terminal	U	G	12406	A	ND5	Non-synonymous	V24I	0	0.61	0.026
Modern terminal	U	G	12406	A	ND5	Non-synonymous	V24I	0	0.61	0.026
Modern terminal	U	G	12406	A	ND5	Non-synonymous	V24I	0	0.61	0.026
Modern terminal	U	G	12406	A	ND5	Non-synonymous	V24I	0	0.61	0.026
Pre-terminal	U	G	12406	A	ND5	Non-synonymous	V24I	0	0.61	0.026
Pre-terminal	U	G	12406	A	ND5	Non-synonymous	V24I	0	0.61	0.026
Pre-terminal	U	G	12406	A	ND5	Non-synonymous	V24I	0	0.61	0.026
Pre-terminal	U	G	12406	A	ND5	Non-synonymous	V24I	0	0.61	0.026
Pre-terminal	U	G	12406	A	ND5	Non-synonymous	V24I	0	0.61	0.026
Pre-terminal	U	G	12406	A	ND5	Non-synonymous	V24I	0	0.61	0.026
Pre-terminal	U	G	12406	A	ND5	Non-synonymous	V24I	0	0.61	0.026
Modern terminal	JT	G	12406	A	ND5	Non-synonymous	V24I	0	0.61	0.026
Modern terminal	JT	G	12406	A	ND5	Non-synonymous	V24I	0	0.61	0.026
Modern terminal	JT	G	12406	A	ND5	Non-synonymous	V24I	0	0.61	0.026
Modern terminal	JT	G	12406	A	ND5	Non-synonymous	V24I	0	0.61	0.026
Modern terminal	JT	G	12406	A	ND5	Non-synonymous	V24I	0	0.61	0.026
Modern terminal	JT	G	12406	A	ND5	Non-synonymous	V24I	0	0.61	0.026
Pre-terminal	JT	G	12406	A	ND5	Non-synonymous	V24I	0	0.61	0.026
Pre-terminal	JT	G	12406	A	ND5	Non-synonymous	V24I	0	0.61	0.026
Pre-terminal	JT	G	12406	A	ND5	Non-synonymous	V24I	0	0.61	0.026
Pre-terminal	JT	G	12406	A	ND5	Non-synonymous	V24I	0	0.61	0.026
Modern terminal	N1	G	12406	A	ND5	Non-synonymous	V24I	0	0.61	0.026
Pre-terminal	N1	G	12406	A	ND5	Non-synonymous	V24I	0	0.61	0.026
Modern terminal	X	G	12406	A	ND5	Non-synonymous	V24I	0	0.61	0.026
Modern terminal	X	G	12406	A	ND5	Non-synonymous	V24I	0	0.61	0.026
Pre-terminal	X	G	12406	A	ND5	Non-synonymous	V24I	0	0.61	0.026
Modern terminal	N2	G	12406	A	ND5	Non-synonymous	V24I	0	0.61	0.026
Pre-terminal	R0	A	12416	G	ND5	Non-synonymous	N27S	0.994	0.37	0.098
Modern terminal	U	A	12416	G	ND5	Non-synonymous	N27S	0.994	0.37	0.098
Modern terminal	U	A	12421	G	ND5	Non-synonymous	K29E	0.994	0.72	0.285
Modern terminal	U	A	12421	G	ND5	Non-synonymous	K29E	0.994	0.72	0.285
Pre-terminal	R0	A	12425	G	ND5	Non-synonymous	N30S	0.02	0.53	0.074

Modern terminal	U	A	12425	G	ND5	Non-synonymous	N30S	0.02	0.53	0.074
Modern terminal	U	A	12425	T	ND5	Non-synonymous	N30I	0.894	0.14	0.193
Modern terminal	U	C	12434	T	ND5	Non-synonymous	P33L	1	0.26	0.381
Modern terminal	R0	A	12437	G	ND5	Non-synonymous	H34R	0.978	0.36	0.258
Modern terminal	R0	A	12437	G	ND5	Non-synonymous	H34R	0.978	0.36	0.258
Modern terminal	U	T	12438	G	ND5	Non-synonymous	H34Q	0.999	0.32	0.242
Pre-terminal	U	T	12448	A	ND5	Non-synonymous	S38T	0	1	0.064
Modern terminal	R0	A	12451	G	ND5	Non-synonymous	I39V	0.002	0.5	0.069
Ancient terminal	U	A	12451	G	ND5	Non-synonymous	I39V	0.002	0.5	0.069
Modern terminal	R0	T	12452	A	ND5	Non-synonymous	I39N	0.998	0.19	0.252
Pre-terminal	R0	G	12454	A	ND5	Non-synonymous	V40I	0	0.79	0.063
Modern terminal	U	G	12454	A	ND5	Non-synonymous	V40I	0	0.79	0.063
Modern terminal	JT	G	12454	A	ND5	Non-synonymous	V40I	0	0.79	0.063
Modern terminal	JT	G	12454	A	ND5	Non-synonymous	V40I	0	0.79	0.063
Modern terminal	JT	G	12454	A	ND5	Non-synonymous	V40I	0	0.79	0.063
Modern terminal	N1	G	12454	A	ND5	Non-synonymous	V40I	0	0.79	0.063
Modern terminal	X	G	12454	A	ND5	Non-synonymous	V40I	0	0.79	0.063
Ancient terminal	JT	T	12460	G	ND5	Non-synonymous	S42A	0.132	0.49	0.173
Modern terminal	JT	A	12469	G	ND5	Non-synonymous	I45V	0.03	0.18	0.1
Modern terminal	N1	A	12469	G	ND5	Non-synonymous	I45V	0.03	0.18	0.1
Pre-terminal	JT	T	12481	A	ND5	Non-synonymous	F49I	0.005	1	0.168
Pre-terminal	R0	A	12490	G	ND5	Non-synonymous	T52A	0.34	0.12	0.184
Pre-terminal	R0	A	12490	G	ND5	Non-synonymous	T52A	0.34	0.12	0.184
Modern terminal	JT	A	12490	G	ND5	Non-synonymous	T52A	0.34	0.12	0.184
Modern terminal	R0	C	12491	T	ND5	Non-synonymous	T52M	0.102	0.77	0.174
Pre-terminal	U	C	12491	T	ND5	Non-synonymous	T52M	0.102	0.77	0.174
Modern terminal	R0	A	12493	G	ND5	Non-synonymous	M53V	0.566	0.3	0.202
Pre-terminal	U	A	12493	G	ND5	Non-synonymous	M53V	0.566	0.3	0.202
Modern terminal	U	A	12499	G	ND5	Non-synonymous	M55V	0.201	0.08	0.148
Modern terminal	R0	G	12503	A	ND5	Non-synonymous	C56Y	0.995	1	0.216
Modern terminal	N2	A	12509	G	ND5	Non-synonymous	D58G	0.006	1	0.286
Modern terminal	R0	A	12512	T	ND5	Non-synonymous	Q59L	0.86	0.73	0.554
Modern terminal	R0	A	12523	G	ND5	Non-synonymous	I63V	0.015	0.44	0.12
Modern terminal	U	T	12524	C	ND5	Non-synonymous	I63T	0.997	0.19	0.283
Modern terminal	R0	A	12530	G	ND5	Non-synonymous	N65S	0.075	0.16	0.101
Modern terminal	U	A	12530	G	ND5	Non-synonymous	N65S	0.075	0.16	0.101
Modern terminal	U	A	12530	G	ND5	Non-synonymous	N65S	0.075	0.16	0.101
Modern terminal	U	A	12530	G	ND5	Non-synonymous	N65S	0.075	0.16	0.101
Pre-terminal	U	A	12530	G	ND5	Non-synonymous	N65S	0.075	0.16	0.101
Modern terminal	JT	A	12530	G	ND5	Non-synonymous	N65S	0.075	0.16	0.101
Modern terminal	X	A	12530	G	ND5	Non-synonymous	N65S	0.075	0.16	0.101
Ancient terminal	U	G	12533	A	ND5	Nonsense				
Modern terminal	R0	C	12535	T	ND5	Non-synonymous	H67Y	0.975	0.18	0.331
Pre-terminal	U	C	12535	T	ND5	Non-synonymous	H67Y	0.975	0.18	0.331
Modern terminal	JT	C	12535	T	ND5	Non-synonymous	H67Y	0.975	0.18	0.331
Modern terminal	N1	C	12535	T	ND5	Non-synonymous	H67Y	0.975	0.18	0.331
Ancient terminal	N1	G	12541	A	ND5	Non-synonymous	A69T	0.001	0.12	0.054
Pre-terminal	R0	C	12542	T	ND5	Non-synonymous	A69V	0.562	0.26	0.091
Modern terminal	U	A	12544	G	ND5	Non-synonymous	T70A	0.011	0.26	0.146
Modern terminal	R0	C	12557	T	ND5	Non-synonymous	T74I	0.001	0.24	0.094

Modern terminal	R0	C	12557	T	ND5	Non-synonymous	T74I	0.001	0.24	0.094
Modern terminal	R0	C	12557	T	ND5	Non-synonymous	T74I	0.001	0.24	0.094
Pre-terminal	R0	C	12557	T	ND5	Non-synonymous	T74I	0.001	0.24	0.094
Pre-terminal	R0	C	12557	T	ND5	Non-synonymous	T74I	0.001	0.24	0.094
Pre-terminal	U	C	12557	T	ND5	Non-synonymous	T74I	0.001	0.24	0.094
Pre-terminal	U	C	12557	T	ND5	Non-synonymous	T74I	0.001	0.24	0.094
Pre-terminal	U	C	12557	T	ND5	Non-synonymous	T74I	0.001	0.24	0.094
Pre-terminal	JT	C	12557	T	ND5	Non-synonymous	T74I	0.001	0.24	0.094
Pre-terminal	JT	C	12557	T	ND5	Non-synonymous	T74I	0.001	0.24	0.094
Pre-terminal	N1	C	12557	T	ND5	Non-synonymous	T74I	0.001	0.24	0.094
Modern terminal	N2	C	12557	T	ND5	Non-synonymous	T74I	0.001	0.24	0.094
Pre-terminal	N2	C	12557	T	ND5	Non-synonymous	T74I	0.001	0.24	0.094
Modern terminal	R0	C	12562	T	ND5	Non-synonymous	L76F	1	0.01	0.148
Modern terminal	R0	C	12562	G	ND5	Non-synonymous	L76V	0.993	0.19	0.125
Modern terminal	R0	C	12562	G	ND5	Non-synonymous	L76V	0.993	0.19	0.125
Modern terminal	U	C	12562	G	ND5	Non-synonymous	L76V	0.993	0.19	0.125
Modern terminal	U	C	12562	G	ND5	Non-synonymous	L76V	0.993	0.19	0.125
Pre-terminal	U	C	12562	T	ND5	Non-synonymous	L76F	1	0.01	0.148
Ancient terminal	JT	C	12562	A	ND5	Non-synonymous	L76I	0.996	0.52	0.074
Modern terminal	JT	T	12563	A	ND5	Non-synonymous	L76H	1	0	0.317
Ancient terminal	R0	T	12565	A	ND5	Non-synonymous	S77T	0.942	0.57	0.032
Pre-terminal	JT	T	12565	A	ND5	Non-synonymous	S77T	0.942	0.57	0.032
Modern terminal	U	A	12571	G	ND5	Non-synonymous	S79G	1	0.06	0.163
Ancient terminal	R0	T	12574	A	ND5	Non-synonymous	F80I	0.999	0.05	0.307
Modern terminal	N2	T	12574	C	ND5	Non-synonymous	F80L	1	0.09	0.256
Ancient terminal	R0	A	12587	T	ND5	Non-synonymous	Y84F	0.997	0.4	0.144
Pre-terminal	JT	A	12587	T	ND5	Non-synonymous	Y84F	0.997	0.4	0.144
Modern terminal	R0	A	12595	G	ND5	Non-synonymous	M87V	0.088	0.68	0.21
Modern terminal	N1	A	12598	G	ND5	Non-synonymous	M88V	0.827	0.39	0.262
Pre-terminal	R0	T	12599	C	ND5	Non-synonymous	M88T	0.003	0.51	0.276
Pre-terminal	R0	T	12599	C	ND5	Non-synonymous	M88T	0.003	0.51	0.276
Pre-terminal	R0	T	12599	C	ND5	Non-synonymous	M88T	0.003	0.51	0.276
Ancient terminal	U	T	12599	C	ND5	Non-synonymous	M88T	0.003	0.51	0.276
Ancient terminal	U	T	12599	C	ND5	Non-synonymous	M88T	0.003	0.51	0.276
Ancient terminal	U	T	12599	C	ND5	Non-synonymous	M88T	0.003	0.51	0.276
Modern terminal	U	T	12599	C	ND5	Non-synonymous	M88T	0.003	0.51	0.276
Pre-terminal	U	T	12599	C	ND5	Non-synonymous	M88T	0.003	0.51	0.276
Modern terminal	JT	T	12599	C	ND5	Non-synonymous	M88T	0.003	0.51	0.276
Pre-terminal	JT	T	12599	C	ND5	Non-synonymous	M88T	0.003	0.51	0.276
Pre-terminal	X	T	12599	C	ND5	Non-synonymous	M88T	0.003	0.51	0.276
Ancient terminal	N1	C	12607	A	ND5	Non-synonymous	P91T	1	0.07	0.508
Ancient terminal	R0	T	12611	C	ND5	Non-synonymous	V92A	0.977	0	0.297
Pre-terminal	R0	G	12613	T	ND5	Non-synonymous	A93S	0.981	0.03	0.275
Modern terminal	U	G	12613	A	ND5	Non-synonymous	A93T	0.999	0	0.396
Modern terminal	X	G	12613	A	ND5	Non-synonymous	A93T	0.999	0	0.396
Pre-terminal	X	G	12613	A	ND5	Non-synonymous	A93T	0.999	0	0.396
Pre-terminal	X	G	12613	A	ND5	Non-synonymous	A93T	0.999	0	0.396
Modern terminal	N2	G	12613	A	ND5	Non-synonymous	A93T	0.999	0	0.396
Modern terminal	N2	G	12613	A	ND5	Non-synonymous	A93T	0.999	0	0.396
Pre-terminal	N2	G	12613	A	ND5	Non-synonymous	A93T	0.999	0	0.396

Ancient terminal	N2	G	12618	T	ND5	Non-synonymous	L94F	1	0	0.53
Modern terminal	JT	T	12619	C	ND5	Non-synonymous	F95L	0.139	0.1	0.596
Modern terminal	R0	G	12622	A	ND5	Non-synonymous	V96I	0.001	0.33	0.055
Modern terminal	R0	G	12622	A	ND5	Non-synonymous	V96I	0.001	0.33	0.055
Modern terminal	U	G	12622	A	ND5	Non-synonymous	V96I	0.001	0.33	0.055
Pre-terminal	U	G	12622	A	ND5	Non-synonymous	V96I	0.001	0.33	0.055
Modern terminal	JT	G	12622	A	ND5	Non-synonymous	V96I	0.001	0.33	0.055
Modern terminal	U	T	12623	G	ND5	Non-synonymous	V96G	1	0	0.505
Ancient terminal	X	C	12626	T	ND5	Non-synonymous	T97M	1	0	0.554
Modern terminal	N2	G	12630	T	ND5	Non-synonymous	W98C	1	0	0.908
Modern terminal	R0	A	12634	G	ND5	Non-synonymous	I100V	0.944	0	0.312
Modern terminal	R0	A	12634	G	ND5	Non-synonymous	I100V	0.944	0	0.312
Pre-terminal	R0	A	12634	G	ND5	Non-synonymous	I100V	0.944	0	0.312
Pre-terminal	R0	A	12634	G	ND5	Non-synonymous	I100V	0.944	0	0.312
Modern terminal	U	A	12634	G	ND5	Non-synonymous	I100V	0.944	0	0.312
Modern terminal	U	A	12634	G	ND5	Non-synonymous	I100V	0.944	0	0.312
Modern terminal	U	A	12634	G	ND5	Non-synonymous	I100V	0.944	0	0.312
Modern terminal	U	A	12634	G	ND5	Non-synonymous	I100V	0.944	0	0.312
Modern terminal	U	A	12634	G	ND5	Non-synonymous	I100V	0.944	0	0.312
Modern terminal	U	A	12634	G	ND5	Non-synonymous	I100V	0.944	0	0.312
Modern terminal	U	A	12634	G	ND5	Non-synonymous	I100V	0.944	0	0.312
Pre-terminal	U	A	12634	G	ND5	Non-synonymous	I100V	0.944	0	0.312
Pre-terminal	JT	A	12634	G	ND5	Non-synonymous	I100V	0.944	0	0.312
Modern terminal	R0	T	12635	C	ND5	Non-synonymous	I100T	1	0	0.538
Modern terminal	U	T	12638	A	ND5	Non-synonymous	M101K	0.93	0	0.825
Modern terminal	R0	G	12640	A	ND5	Non-synonymous	E102K	0.995	0.01	0.794
Modern terminal	R0	G	12640	A	ND5	Non-synonymous	E102K	0.995	0.01	0.794
Modern terminal	R0	A	12642	T	ND5	Non-synonymous	E102D	0.995	0.04	0.662
Modern terminal	X	T	12650	A	ND5	Non-synonymous	L105Q	1	0.19	0.878
Modern terminal	R0	T	12655	A	ND5	Non-synonymous	Y107N	1	0	0.853
Modern terminal	U	T	12655	A	ND5	Non-synonymous	Y107N	1	0	0.853
Modern terminal	U	T	12655	A	ND5	Non-synonymous	Y107N	1	0	0.853
Modern terminal	R0	T	12657	A	ND5	Nonsense				
Modern terminal	U	A	12658	T	ND5	Non-synonymous	M108L	0.515	0	0.58
Pre-terminal	R0	A	12661	G	ND5	Non-synonymous	N109D	0.001	0.48	0.364
Modern terminal	JT	A	12661	G	ND5	Non-synonymous	N109D	0.001	0.48	0.364
Modern terminal	N2	A	12661	G	ND5	Non-synonymous	N109D	0.001	0.48	0.364
Modern terminal	R0	A	12662	G	ND5	Non-synonymous	N109S	0.212	0.39	0.188
Modern terminal	R0	A	12662	G	ND5	Non-synonymous	N109S	0.212	0.39	0.188
Modern terminal	R0	A	12662	G	ND5	Non-synonymous	N109S	0.212	0.39	0.188
Pre-terminal	R0	A	12662	G	ND5	Non-synonymous	N109S	0.212	0.39	0.188
Pre-terminal	R0	A	12662	G	ND5	Non-synonymous	N109S	0.212	0.39	0.188
Pre-terminal	R0	A	12662	G	ND5	Non-synonymous	N109S	0.212	0.39	0.188
Pre-terminal	R0	A	12662	G	ND5	Non-synonymous	N109S	0.212	0.39	0.188
Modern terminal	U	A	12662	G	ND5	Non-synonymous	N109S	0.212	0.39	0.188
Modern terminal	U	A	12662	G	ND5	Non-synonymous	N109S	0.212	0.39	0.188
Modern terminal	JT	A	12662	C	ND5	Non-synonymous	N109T	0.117	0.11	0.498
Modern terminal	JT	A	12662	G	ND5	Non-synonymous	N109S	0.212	0.39	0.188
Modern terminal	JT	A	12662	G	ND5	Non-synonymous	N109S	0.212	0.39	0.188
Modern terminal	U	C	12663	A	ND5	Non-synonymous	N109K	0.515	0.25	0.513

Modern terminal	JT	C	12663	A	ND5	Non-synonymous	N109K	0.515	0.25	0.513
Modern terminal	JT	G	12667	A	ND5	Non-synonymous	D111N	0.998	0.01	0.503
Pre-terminal	R0	A	12674	G	ND5	Non-synonymous	N113S	0.999	0.36	0.24
Modern terminal	R0	A	12679	G	ND5	Non-synonymous	N115D	0.999	0.23	0.323
Modern terminal	JT	C	12708	A	ND5	Non-synonymous	F124L	0.999	0	0.725
Modern terminal	R0	A	12712	G	ND5	Non-synonymous	I126V	0.1	0.12	0.215
Modern terminal	R0	A	12712	G	ND5	Non-synonymous	I126V	0.1	0.12	0.215
Pre-terminal	R0	A	12712	G	ND5	Non-synonymous	I126V	0.1	0.12	0.215
Modern terminal	U	A	12712	G	ND5	Non-synonymous	I126V	0.1	0.12	0.215
Modern terminal	R0	A	12715	G	ND5	Non-synonymous	T127A	0.994	0.42	0.295
Ancient terminal	U	A	12715	G	ND5	Non-synonymous	T127A	0.994	0.42	0.295
Modern terminal	U	A	12715	G	ND5	Non-synonymous	T127A	0.994	0.42	0.295
Modern terminal	U	A	12715	G	ND5	Non-synonymous	T127A	0.994	0.42	0.295
Pre-terminal	JT	A	12715	G	ND5	Non-synonymous	T127A	0.994	0.42	0.295
Modern terminal	R0	C	12721	A	ND5	Non-synonymous	L129M	0.998	0.38	0.37
Pre-terminal	R0	G	12730	A	ND5	Non-synonymous	V132I	0.001	0.23	0.069
Modern terminal	JT	A	12733	G	ND5	Non-synonymous	T133A	0.994	0.01	0.584
Ancient terminal	U	A	12743	G	ND5	Non-synonymous	N136S	0.999	0.13	0.47
Modern terminal	R0	T	12757	C	ND5	Non-synonymous	F141L	1	0.1	0.619
Modern terminal	R0	T	12757	C	ND5	Non-synonymous	F141L	1	0.1	0.619
Modern terminal	R0	T	12757	C	ND5	Non-synonymous	F141L	1	0.1	0.619
Modern terminal	U	T	12757	C	ND5	Non-synonymous	F141L	1	0.1	0.619
Modern terminal	U	T	12757	C	ND5	Non-synonymous	F141L	1	0.1	0.619
Pre-terminal	JT	T	12757	C	ND5	Non-synonymous	F141L	1	0.1	0.619
Pre-terminal	R0	G	12775	A	ND5	Non-synonymous	V147M	0.999	0.04	0.574
Pre-terminal	U	G	12775	A	ND5	Non-synonymous	V147M	0.999	0.04	0.574
Modern terminal	N1	G	12775	A	ND5	Non-synonymous	V147M	0.999	0.04	0.574
Ancient terminal	U	G	12778	T	ND5	Non-synonymous	G148W	1	0	0.887
Modern terminal	JT	G	12779	A	ND5	Non-synonymous	G148E	1	0	0.836
Pre-terminal	R0	A	12781	G	ND5	Non-synonymous	I149V	0.944	0.01	0.404
Ancient terminal	R0	T	12811	C	ND5	Non-synonymous	Y159H	0.137	0.62	0.35
Modern terminal	R0	T	12811	C	ND5	Non-synonymous	Y159H	0.137	0.62	0.35
Modern terminal	R0	T	12811	C	ND5	Non-synonymous	Y159H	0.137	0.62	0.35
Pre-terminal	R0	T	12811	C	ND5	Non-synonymous	Y159H	0.137	0.62	0.35
Ancient terminal	U	T	12811	C	ND5	Non-synonymous	Y159H	0.137	0.62	0.35
Ancient terminal	U	T	12811	C	ND5	Non-synonymous	Y159H	0.137	0.62	0.35
Modern terminal	U	T	12811	C	ND5	Non-synonymous	Y159H	0.137	0.62	0.35
Pre-terminal	U	T	12811	C	ND5	Non-synonymous	Y159H	0.137	0.62	0.35
Pre-terminal	U	T	12811	C	ND5	Non-synonymous	Y159H	0.137	0.62	0.35
Ancient terminal	JT	T	12811	C	ND5	Non-synonymous	Y159H	0.137	0.62	0.35
Modern terminal	JT	T	12811	C	ND5	Non-synonymous	Y159H	0.137	0.62	0.35
Pre-terminal	N1	T	12811	C	ND5	Non-synonymous	Y159H	0.137	0.62	0.35
Modern terminal	U	A	12812	G	ND5	Non-synonymous	Y159C	0.046	0.02	0.651
Modern terminal	U	G	12814	T	ND5	Non-synonymous	A160S	0.981	0.13	0.165
Modern terminal	U	G	12820	A	ND5	Non-synonymous	A162T	0	1	0.062
Modern terminal	U	G	12820	A	ND5	Non-synonymous	A162T	0	1	0.062
Modern terminal	JT	G	12823	A	ND5	Non-synonymous	D163N	0.887	0.09	0.575
Ancient terminal	U	A	12832	G	ND5	Non-synonymous	T166A	0.997	0.01	0.685
Ancient terminal	X	G	12835	A	ND5	Non-synonymous	A167T	0.999	0	0.692
Modern terminal	R0	A	12841	G	ND5	Non-synonymous	I169V	0.005	0.01	0.348

Modern terminal	N1	A	12841	G	ND5	Non-synonymous	I169V	0.005	0.01	0.348
Modern terminal	R0	T	12842	C	ND5	Non-synonymous	I169T	0.991	0	0.672
Pre-terminal	R0	T	12842	C	ND5	Non-synonymous	I169T	0.991	0	0.672
Pre-terminal	U	T	12842	C	ND5	Non-synonymous	I169T	0.991	0	0.672
Modern terminal	R0	A	12850	G	ND5	Non-synonymous	I172V	0.944	0.99	0.298
Modern terminal	U	A	12850	G	ND5	Non-synonymous	I172V	0.944	0.99	0.298
Modern terminal	U	A	12850	G	ND5	Non-synonymous	I172V	0.944	0.99	0.298
Ancient terminal	JT	A	12850	T	ND5	Non-synonymous	I172F	0.994	0.01	0.782
Modern terminal	JT	A	12850	G	ND5	Non-synonymous	I172V	0.944	0.99	0.298
Modern terminal	JT	A	12850	G	ND5	Non-synonymous	I172V	0.944	0.99	0.298
Modern terminal	N1	A	12850	G	ND5	Non-synonymous	I172V	0.944	0.99	0.298
Ancient terminal	R0	T	12856	A	ND5	Non-synonymous	Y174N	1	0	0.903
Modern terminal	R0	A	12865	G	ND5	Non-synonymous	I177V	0.944	0.51	0.318
Modern terminal	JT	C	12867	A	ND5	Non-synonymous	I177M	1	0.04	0.599
Pre-terminal	JT	C	12867	A	ND5	Non-synonymous	I177M	1	0.04	0.599
Modern terminal	R0	A	12874	G	ND5	Non-synonymous	I180V	0.944	0.16	0.323
Modern terminal	R0	T	12880	C	ND5	Non-synonymous	F182L	1	1	0.312
Modern terminal	R0	T	12880	C	ND5	Non-synonymous	F182L	1	1	0.312
Modern terminal	U	T	12880	C	ND5	Non-synonymous	F182L	1	1	0.312
Modern terminal	U	T	12880	C	ND5	Non-synonymous	F182L	1	1	0.312
Modern terminal	JT	T	12880	C	ND5	Non-synonymous	F182L	1	1	0.312
Modern terminal	JT	T	12880	C	ND5	Non-synonymous	F182L	1	1	0.312
Pre-terminal	N1	T	12880	C	ND5	Non-synonymous	F182L	1	1	0.312
Modern terminal	JT	A	12883	G	ND5	Non-synonymous	I183V	0.005	0.16	0.179
Modern terminal	R0	G	12889	A	ND5	Non-synonymous	A185T	0.011	0.68	0.412
Ancient terminal	JT	G	12895	A	ND5	Non-synonymous	A187T	0.019	0.06	0.29
Pre-terminal	R0	A	12904	G	ND5	Non-synonymous	I190V	0.002	0.53	0.068
Modern terminal	N1	A	12904	G	ND5	Non-synonymous	I190V	0.002	0.53	0.068
Modern terminal	N1	A	12904	G	ND5	Non-synonymous	I190V	0.002	0.53	0.068
Modern terminal	R0	T	12905	G	ND5	Non-synonymous	I190S	0.944	0.12	0.281
Pre-terminal	R0	C	12906	A	ND5	Non-synonymous	I190M	0.498	0.21	0.09
Pre-terminal	R0	C	12906	A	ND5	Non-synonymous	I190M	0.498	0.21	0.09
Modern terminal	U	C	12906	A	ND5	Non-synonymous	I190M	0.498	0.21	0.09
Modern terminal	JT	C	12906	A	ND5	Non-synonymous	I190M	0.498	0.21	0.09
Modern terminal	JT	C	12906	A	ND5	Non-synonymous	I190M	0.498	0.21	0.09
Pre-terminal	JT	C	12906	A	ND5	Non-synonymous	I190M	0.498	0.21	0.09
Pre-terminal	JT	C	12906	A	ND5	Non-synonymous	I190M	0.498	0.21	0.09
Modern terminal	U	T	12908	A	ND5	Non-synonymous	L191Q	1	0.05	0.602
Modern terminal	U	A	12916	C	ND5	Non-synonymous	N194H	1	0	0.197
Modern terminal	JT	G	12923	T	ND5	Non-synonymous	W196L	1	0.01	0.627
Modern terminal	N2	G	12923	T	ND5	Non-synonymous	W196L	1	0.01	0.627
Pre-terminal	N2	G	12923	T	ND5	Non-synonymous	W196L	1	0.01	0.627
Modern terminal	R0	G	12940	A	ND5	Non-synonymous	A202T	0.001	0.08	0.101
Modern terminal	R0	G	12940	A	ND5	Non-synonymous	A202T	0.001	0.08	0.101
Pre-terminal	R0	G	12940	A	ND5	Non-synonymous	A202T	0.001	0.08	0.101
Pre-terminal	R0	G	12940	A	ND5	Non-synonymous	A202T	0.001	0.08	0.101
Ancient terminal	JT	G	12940	A	ND5	Non-synonymous	A202T	0.001	0.08	0.101
Modern terminal	JT	G	12940	A	ND5	Non-synonymous	A202T	0.001	0.08	0.101
Pre-terminal	JT	G	12940	A	ND5	Non-synonymous	A202T	0.001	0.08	0.101
Modern terminal	R0	C	12941	T	ND5	Non-synonymous	A202V	0.001	0.02	0.095

Ancient terminal	U	C	12941	T	ND5	Non-synonymous	A202V	0.001	0.02	0.095
Modern terminal	N2	C	12941	T	ND5	Non-synonymous	A202V	0.001	0.02	0.095
Modern terminal	R0	A	12950	G	ND5	Non-synonymous	N205S	0.019	0.84	0.111
Modern terminal	R0	A	12950	G	ND5	Non-synonymous	N205S	0.019	0.84	0.111
Modern terminal	R0	A	12950	G	ND5	Non-synonymous	N205S	0.019	0.84	0.111
Modern terminal	R0	A	12950	G	ND5	Non-synonymous	N205S	0.019	0.84	0.111
Pre-terminal	R0	A	12950	G	ND5	Non-synonymous	N205S	0.019	0.84	0.111
Pre-terminal	R0	A	12950	G	ND5	Non-synonymous	N205S	0.019	0.84	0.111
Modern terminal	U	A	12950	G	ND5	Non-synonymous	N205S	0.019	0.84	0.111
Modern terminal	U	A	12950	G	ND5	Non-synonymous	N205S	0.019	0.84	0.111
Modern terminal	U	A	12950	G	ND5	Non-synonymous	N205S	0.019	0.84	0.111
Pre-terminal	U	A	12950	G	ND5	Non-synonymous	N205S	0.019	0.84	0.111
Pre-terminal	U	A	12950	G	ND5	Non-synonymous	N205S	0.019	0.84	0.111
Pre-terminal	U	A	12950	G	ND5	Non-synonymous	N205S	0.019	0.84	0.111
Pre-terminal	U	A	12950	G	ND5	Non-synonymous	N205S	0.019	0.84	0.111
Modern terminal	JT	A	12950	G	ND5	Non-synonymous	N205S	0.019	0.84	0.111
Modern terminal	JT	A	12950	G	ND5	Non-synonymous	N205S	0.019	0.84	0.111
Modern terminal	JT	A	12950	G	ND5	Non-synonymous	N205S	0.019	0.84	0.111
Modern terminal	R0	G	12952	A	ND5	Non-synonymous	A206T	0	0.63	0.062
Modern terminal	R0	G	12952	A	ND5	Non-synonymous	A206T	0	0.63	0.062
Modern terminal	U	G	12952	A	ND5	Non-synonymous	A206T	0	0.63	0.062
Modern terminal	U	G	12952	A	ND5	Non-synonymous	A206T	0	0.63	0.062
Modern terminal	JT	G	12952	A	ND5	Non-synonymous	A206T	0	0.63	0.062
Modern terminal	JT	G	12952	A	ND5	Non-synonymous	A206T	0	0.63	0.062
Modern terminal	JT	G	12952	A	ND5	Non-synonymous	A206T	0	0.63	0.062
Modern terminal	U	A	12961	G	ND5	Non-synonymous	S209G	0.726	0.33	0.094
Pre-terminal	N1	A	12961	G	ND5	Non-synonymous	S209G	0.726	0.33	0.094
Modern terminal	R0	C	12964	A	ND5	Non-synonymous	L210I	0.492	0.47	0.062
Modern terminal	R0	T	12965	C	ND5	Non-synonymous	L210P	1	0.2	0.226
Pre-terminal	JT	A	12967	G	ND5	Non-synonymous	T211A	0.217	0.05	0.094
Modern terminal	R0	C	12982	T	ND5	Non-synonymous	L216F	0.016	0.04	0.214
Ancient terminal	R0	C	12998	A	ND5	Non-synonymous	A221E	0.93	0	0.317
Modern terminal	JT	C	13036	T	ND5	Non-synonymous	P234S	1	0	0.233
Modern terminal	R0	G	13042	A	ND5	Non-synonymous	A236T	0.999	0	0.242
Ancient terminal	U	G	13042	A	ND5	Non-synonymous	A236T	0.999	0	0.242
Modern terminal	R0	C	13043	T	ND5	Non-synonymous	A236V	1	0	0.237
Ancient terminal	U	A	13049	G	ND5	Non-synonymous	E238G	1	0	0.377
Modern terminal	U	G	13051	C	ND5	Non-synonymous	G239R	1	0	0.409
Ancient terminal	JT	G	13063	T	ND5	Non-synonymous	V243F	0.999	0	0.314
Ancient terminal	R0	T	13064	A	ND5	Non-synonymous	V243D	1	0	0.547
Modern terminal	JT	T	13064	C	ND5	Non-synonymous	V243A	1	0	0.23
Modern terminal	N1	T	13064	C	ND5	Non-synonymous	V243A	1	0	0.23
Pre-terminal	N1	T	13064	C	ND5	Non-synonymous	V243A	1	0	0.23
Modern terminal	N2	T	13064	C	ND5	Non-synonymous	V243A	1	0	0.23
Ancient terminal	R0	T	13066	A	ND5	Non-synonymous	S244T	0.942	0.01	0.205
Ancient terminal	U	T	13073	A	ND5	Non-synonymous	L246Q	1	0	0.597
Ancient terminal	U	T	13076	A	ND5	Non-synonymous	L247H	1	0	0.795
Ancient terminal	X	C	13078	T	ND5	Non-synonymous	H248Y	0.989	0	0.353
Ancient terminal	U	G	13093	T	ND5	Non-synonymous	V253F	0.999	0	0.443
Ancient terminal	R0	G	13099	T	ND5	Non-synonymous	A255S	0.981	0.05	0.268

Modern terminal	R0	G	13099	A	ND5	Non-synonymous	A255T	0.999	0	0.437
Ancient terminal	R0	A	13105	G	ND5	Non-synonymous	I257V	0.002	0.94	0.032
Modern terminal	R0	A	13105	G	ND5	Non-synonymous	I257V	0.002	0.94	0.032
Modern terminal	R0	A	13105	G	ND5	Non-synonymous	I257V	0.002	0.94	0.032
Modern terminal	R0	A	13105	G	ND5	Non-synonymous	I257V	0.002	0.94	0.032
Modern terminal	R0	A	13105	G	ND5	Non-synonymous	I257V	0.002	0.94	0.032
Modern terminal	R0	A	13105	G	ND5	Non-synonymous	I257V	0.002	0.94	0.032
Modern terminal	R0	A	13105	G	ND5	Non-synonymous	I257V	0.002	0.94	0.032
Modern terminal	R0	A	13105	G	ND5	Non-synonymous	I257V	0.002	0.94	0.032
Modern terminal	R0	A	13105	G	ND5	Non-synonymous	I257V	0.002	0.94	0.032
Modern terminal	R0	A	13105	G	ND5	Non-synonymous	I257V	0.002	0.94	0.032
Modern terminal	R0	A	13105	G	ND5	Non-synonymous	I257V	0.002	0.94	0.032
Modern terminal	R0	A	13105	G	ND5	Non-synonymous	I257V	0.002	0.94	0.032
Modern terminal	R0	A	13105	G	ND5	Non-synonymous	I257V	0.002	0.94	0.032
Modern terminal	R0	A	13105	G	ND5	Non-synonymous	I257V	0.002	0.94	0.032
Modern terminal	R0	A	13105	G	ND5	Non-synonymous	I257V	0.002	0.94	0.032
Modern terminal	R0	A	13105	G	ND5	Non-synonymous	I257V	0.002	0.94	0.032
Pre-terminal	R0	A	13105	G	ND5	Non-synonymous	I257V	0.002	0.94	0.032
Pre-terminal	R0	A	13105	G	ND5	Non-synonymous	I257V	0.002	0.94	0.032
Pre-terminal	R0	A	13105	G	ND5	Non-synonymous	I257V	0.002	0.94	0.032
Pre-terminal	R0	A	13105	G	ND5	Non-synonymous	I257V	0.002	0.94	0.032
Pre-terminal	R0	A	13105	G	ND5	Non-synonymous	I257V	0.002	0.94	0.032
Pre-terminal	R0	A	13105	G	ND5	Non-synonymous	I257V	0.002	0.94	0.032
Pre-terminal	R0	A	13105	G	ND5	Non-synonymous	I257V	0.002	0.94	0.032
Pre-terminal	R0	A	13105	G	ND5	Non-synonymous	I257V	0.002	0.94	0.032
Pre-terminal	R0	A	13105	G	ND5	Non-synonymous	I257V	0.002	0.94	0.032
Pre-terminal	R0	A	13105	G	ND5	Non-synonymous	I257V	0.002	0.94	0.032
Pre-terminal	R0	A	13105	G	ND5	Non-synonymous	I257V	0.002	0.94	0.032
Ancient terminal	U	A	13105	G	ND5	Non-synonymous	I257V	0.002	0.94	0.032
Ancient terminal	U	A	13105	G	ND5	Non-synonymous	I257V	0.002	0.94	0.032
Ancient terminal	U	A	13105	G	ND5	Non-synonymous	I257V	0.002	0.94	0.032
Modern terminal	U	A	13105	G	ND5	Non-synonymous	I257V	0.002	0.94	0.032
Modern terminal	U	A	13105	G	ND5	Non-synonymous	I257V	0.002	0.94	0.032
Modern terminal	U	A	13105	G	ND5	Non-synonymous	I257V	0.002	0.94	0.032
Modern terminal	U	A	13105	G	ND5	Non-synonymous	I257V	0.002	0.94	0.032
Modern terminal	U	A	13105	G	ND5	Non-synonymous	I257V	0.002	0.94	0.032
Modern terminal	U	A	13105	G	ND5	Non-synonymous	I257V	0.002	0.94	0.032
Modern terminal	U	A	13105	G	ND5	Non-synonymous	I257V	0.002	0.94	0.032
Modern terminal	U	A	13105	G	ND5	Non-synonymous	I257V	0.002	0.94	0.032
Modern terminal	U	A	13105	G	ND5	Non-synonymous	I257V	0.002	0.94	0.032
Modern terminal	U	A	13105	G	ND5	Non-synonymous	I257V	0.002	0.94	0.032
Modern terminal	U	A	13105	G	ND5	Non-synonymous	I257V	0.002	0.94	0.032
Modern terminal	U	A	13105	G	ND5	Non-synonymous	I257V	0.002	0.94	0.032
Modern terminal	U	A	13105	G	ND5	Non-synonymous	I257V	0.002	0.94	0.032
Modern terminal	U	A	13105	G	ND5	Non-synonymous	I257V	0.002	0.94	0.032
Modern terminal	U	A	13105	G	ND5	Non-synonymous	I257V	0.002	0.94	0.032
Modern terminal	U	A	13105	G	ND5	Non-synonymous	I257V	0.002	0.94	0.032
Modern terminal	U	A	13105	G	ND5	Non-synonymous	I257V	0.002	0.94	0.032
Modern terminal	U	A	13105	G	ND5	Non-synonymous	I257V	0.002	0.94	0.032
Pre-terminal	U	A	13105	G	ND5	Non-synonymous	I257V	0.002	0.94	0.032

Pre-terminal	U	A	13105	G	ND5	Non-synonymous	I257V	0.002	0.94	0.032
Pre-terminal	U	A	13105	G	ND5	Non-synonymous	I257V	0.002	0.94	0.032
Pre-terminal	U	A	13105	G	ND5	Non-synonymous	I257V	0.002	0.94	0.032
Pre-terminal	U	A	13105	G	ND5	Non-synonymous	I257V	0.002	0.94	0.032
Modern terminal	JT	A	13105	G	ND5	Non-synonymous	I257V	0.002	0.94	0.032
Modern terminal	JT	A	13105	G	ND5	Non-synonymous	I257V	0.002	0.94	0.032
Modern terminal	JT	A	13105	G	ND5	Non-synonymous	I257V	0.002	0.94	0.032
Modern terminal	JT	A	13105	G	ND5	Non-synonymous	I257V	0.002	0.94	0.032
Modern terminal	JT	A	13105	G	ND5	Non-synonymous	I257V	0.002	0.94	0.032
Pre-terminal	JT	A	13105	G	ND5	Non-synonymous	I257V	0.002	0.94	0.032
Modern terminal	N1	A	13105	G	ND5	Non-synonymous	I257V	0.002	0.94	0.032
Modern terminal	N1	A	13105	G	ND5	Non-synonymous	I257V	0.002	0.94	0.032
Modern terminal	N1	A	13105	G	ND5	Non-synonymous	I257V	0.002	0.94	0.032
Modern terminal	X	A	13105	G	ND5	Non-synonymous	I257V	0.002	0.94	0.032
Modern terminal	N2	A	13105	G	ND5	Non-synonymous	I257V	0.002	0.94	0.032
Modern terminal	N2	A	13105	G	ND5	Non-synonymous	I257V	0.002	0.94	0.032
Modern terminal	N1	C	13114	A	ND5	Non-synonymous	L260I	0.996	0.01	0.295
Pre-terminal	U	A	13117	G	ND5	Non-synonymous	I261V	0.944	0.19	0.187
Modern terminal	JT	C	13126	T	ND5	Non-synonymous	H264Y	0.001	0.47	0.537
Modern terminal	U	A	13127	G	ND5	Non-synonymous	H264R	0.001	0.02	0.513
Modern terminal	R0	G	13135	A	ND5	Non-synonymous	A267T	0.002	0.23	0.109
Modern terminal	R0	G	13135	A	ND5	Non-synonymous	A267T	0.002	0.23	0.109
Modern terminal	R0	G	13135	A	ND5	Non-synonymous	A267T	0.002	0.23	0.109
Modern terminal	R0	G	13135	A	ND5	Non-synonymous	A267T	0.002	0.23	0.109
Modern terminal	R0	G	13135	A	ND5	Non-synonymous	A267T	0.002	0.23	0.109
Modern terminal	R0	G	13135	A	ND5	Non-synonymous	A267T	0.002	0.23	0.109
Pre-terminal	R0	G	13135	A	ND5	Non-synonymous	A267T	0.002	0.23	0.109
Pre-terminal	R0	G	13135	A	ND5	Non-synonymous	A267T	0.002	0.23	0.109
Pre-terminal	R0	G	13135	A	ND5	Non-synonymous	A267T	0.002	0.23	0.109
Pre-terminal	R0	G	13135	A	ND5	Non-synonymous	A267T	0.002	0.23	0.109
Modern terminal	U	G	13135	A	ND5	Non-synonymous	A267T	0.002	0.23	0.109
Modern terminal	U	G	13135	A	ND5	Non-synonymous	A267T	0.002	0.23	0.109
Modern terminal	U	G	13135	A	ND5	Non-synonymous	A267T	0.002	0.23	0.109
Modern terminal	U	G	13135	A	ND5	Non-synonymous	A267T	0.002	0.23	0.109
Modern terminal	U	G	13135	A	ND5	Non-synonymous	A267T	0.002	0.23	0.109
Modern terminal	U	G	13135	A	ND5	Non-synonymous	A267T	0.002	0.23	0.109
Modern terminal	U	G	13135	A	ND5	Non-synonymous	A267T	0.002	0.23	0.109
Modern terminal	U	G	13135	A	ND5	Non-synonymous	A267T	0.002	0.23	0.109
Modern terminal	U	G	13135	A	ND5	Non-synonymous	A267T	0.002	0.23	0.109
Modern terminal	U	G	13135	A	ND5	Non-synonymous	A267T	0.002	0.23	0.109
Modern terminal	U	G	13135	A	ND5	Non-synonymous	A267T	0.002	0.23	0.109
Pre-terminal	U	G	13135	A	ND5	Non-synonymous	A267T	0.002	0.23	0.109
Pre-terminal	U	G	13135	A	ND5	Non-synonymous	A267T	0.002	0.23	0.109
Pre-terminal	U	G	13135	A	ND5	Non-synonymous	A267T	0.002	0.23	0.109
Modern terminal	JT	G	13135	A	ND5	Non-synonymous	A267T	0.002	0.23	0.109
Modern terminal	JT	G	13135	A	ND5	Non-synonymous	A267T	0.002	0.23	0.109
Modern terminal	JT	G	13135	A	ND5	Non-synonymous	A267T	0.002	0.23	0.109
Modern terminal	JT	G	13135	A	ND5	Non-synonymous	A267T	0.002	0.23	0.109
Modern terminal	JT	G	13135	A	ND5	Non-synonymous	A267T	0.002	0.23	0.109
Modern terminal	JT	G	13135	A	ND5	Non-synonymous	A267T	0.002	0.23	0.109

Pre-terminal	JT	G	13135	A	ND5	Non-synonymous	A267T	0.002	0.23	0.109
Pre-terminal	JT	G	13135	A	ND5	Non-synonymous	A267T	0.002	0.23	0.109
Pre-terminal	JT	G	13135	A	ND5	Non-synonymous	A267T	0.002	0.23	0.109
Pre-terminal	X	G	13135	A	ND5	Non-synonymous	A267T	0.002	0.23	0.109
Modern terminal	N2	G	13135	A	ND5	Non-synonymous	A267T	0.002	0.23	0.109
Modern terminal	JT	C	13136	T	ND5	Non-synonymous	A267V	0.443	0.06	0.161
Ancient terminal	R0	G	13145	A	ND5	Non-synonymous	S270N	0	1	0.042
Modern terminal	R0	G	13145	A	ND5	Non-synonymous	S270N	0	1	0.042
Modern terminal	R0	G	13145	A	ND5	Non-synonymous	S270N	0	1	0.042
Modern terminal	R0	G	13145	A	ND5	Non-synonymous	S270N	0	1	0.042
Modern terminal	R0	G	13145	A	ND5	Non-synonymous	S270N	0	1	0.042
Modern terminal	R0	G	13145	A	ND5	Non-synonymous	S270N	0	1	0.042
Modern terminal	R0	G	13145	A	ND5	Non-synonymous	S270N	0	1	0.042
Modern terminal	R0	G	13145	A	ND5	Non-synonymous	S270N	0	1	0.042
Modern terminal	R0	G	13145	A	ND5	Non-synonymous	S270N	0	1	0.042
Modern terminal	R0	G	13145	A	ND5	Non-synonymous	S270N	0	1	0.042
Modern terminal	R0	G	13145	A	ND5	Non-synonymous	S270N	0	1	0.042
Modern terminal	R0	G	13145	A	ND5	Non-synonymous	S270N	0	1	0.042
Modern terminal	R0	G	13145	A	ND5	Non-synonymous	S270N	0	1	0.042
Modern terminal	R0	G	13145	A	ND5	Non-synonymous	S270N	0	1	0.042
Pre-terminal	R0	G	13145	A	ND5	Non-synonymous	S270N	0	1	0.042
Pre-terminal	R0	G	13145	A	ND5	Non-synonymous	S270N	0	1	0.042
Pre-terminal	R0	G	13145	A	ND5	Non-synonymous	S270N	0	1	0.042
Ancient terminal	U	G	13145	A	ND5	Non-synonymous	S270N	0	1	0.042
Modern terminal	U	G	13145	A	ND5	Non-synonymous	S270N	0	1	0.042
Modern terminal	U	G	13145	A	ND5	Non-synonymous	S270N	0	1	0.042
Modern terminal	U	G	13145	A	ND5	Non-synonymous	S270N	0	1	0.042
Modern terminal	U	G	13145	A	ND5	Non-synonymous	S270N	0	1	0.042
Modern terminal	U	G	13145	A	ND5	Non-synonymous	S270N	0	1	0.042
Modern terminal	U	G	13145	A	ND5	Non-synonymous	S270N	0	1	0.042
Modern terminal	U	G	13145	A	ND5	Non-synonymous	S270N	0	1	0.042
Modern terminal	U	G	13145	A	ND5	Non-synonymous	S270N	0	1	0.042
Modern terminal	U	G	13145	A	ND5	Non-synonymous	S270N	0	1	0.042
Pre-terminal	U	G	13145	A	ND5	Non-synonymous	S270N	0	1	0.042
Pre-terminal	U	G	13145	A	ND5	Non-synonymous	S270N	0	1	0.042
Pre-terminal	U	G	13145	A	ND5	Non-synonymous	S270N	0	1	0.042
Pre-terminal	U	G	13145	A	ND5	Non-synonymous	S270N	0	1	0.042
Pre-terminal	U	G	13145	A	ND5	Non-synonymous	S270N	0	1	0.042
Pre-terminal	U	G	13145	A	ND5	Non-synonymous	S270N	0	1	0.042
Pre-terminal	U	G	13145	A	ND5	Non-synonymous	S270N	0	1	0.042
Modern terminal	JT	G	13145	A	ND5	Non-synonymous	S270N	0	1	0.042
Modern terminal	JT	G	13145	A	ND5	Non-synonymous	S270N	0	1	0.042
Modern terminal	JT	G	13145	A	ND5	Non-synonymous	S270N	0	1	0.042
Pre-terminal	JT	G	13145	A	ND5	Non-synonymous	S270N	0	1	0.042
Pre-terminal	JT	G	13145	A	ND5	Non-synonymous	S270N	0	1	0.042
Pre-terminal	JT	G	13145	A	ND5	Non-synonymous	S270N	0	1	0.042
Pre-terminal	JT	G	13145	A	ND5	Non-synonymous	S270N	0	1	0.042
Pre-terminal	JT	G	13145	A	ND5	Non-synonymous	S270N	0	1	0.042
Modern terminal	N2	G	13145	A	ND5	Non-synonymous	S270N	0	1	0.042
Modern terminal	N2	G	13145	A	ND5	Non-synonymous	S270N	0	1	0.042

Modern terminal	N2	G	13145	A	ND5	Non-synonymous	S270N	0	1	0.042
Pre-terminal	R0	C	13147	T	ND5	Non-synonymous	P271S	0.022	0.47	0.195
Modern terminal	U	C	13147	T	ND5	Non-synonymous	P271S	0.022	0.47	0.195
Modern terminal	R0	C	13148	A	ND5	Non-synonymous	P271Q	0.005	0.61	0.214
Pre-terminal	R0	A	13153	G	ND5	Non-synonymous	I273V	0.003	0.38	0.098
Pre-terminal	U	A	13153	G	ND5	Non-synonymous	I273V	0.003	0.38	0.098
Pre-terminal	U	A	13153	G	ND5	Non-synonymous	I273V	0.003	0.38	0.098
Pre-terminal	JT	A	13153	G	ND5	Non-synonymous	I273V	0.003	0.38	0.098
Pre-terminal	R0	T	13154	C	ND5	Non-synonymous	I273T	0.967	0.05	0.285
Modern terminal	U	T	13154	C	ND5	Non-synonymous	I273T	0.967	0.05	0.285
Modern terminal	R0	A	13183	G	ND5	Non-synonymous	I283V	0.008	0.04	0.171
Modern terminal	JT	A	13183	G	ND5	Non-synonymous	I283V	0.008	0.04	0.171
Modern terminal	R0	T	13184	C	ND5	Non-synonymous	I283T	0.995	0.17	0.364
Modern terminal	R0	T	13184	C	ND5	Non-synonymous	I283T	0.995	0.17	0.364
Modern terminal	U	C	13185	A	ND5	Non-synonymous	I283M	0.998	0.4	0.336
Modern terminal	U	C	13185	A	ND5	Non-synonymous	I283M	0.998	0.4	0.336
Modern terminal	U	T	13195	C	ND5	Non-synonymous	F287L	1	0.01	0.78
Modern terminal	X	G	13198	A	ND5	Non-synonymous	A288T	0.019	1	0.298
Modern terminal	R0	G	13204	A	ND5	Non-synonymous	V290I	0	1	0.064
Modern terminal	R0	G	13204	A	ND5	Non-synonymous	V290I	0	1	0.064
Pre-terminal	U	G	13204	A	ND5	Non-synonymous	V290I	0	1	0.064
Modern terminal	JT	G	13204	A	ND5	Non-synonymous	V290I	0	1	0.064
Modern terminal	JT	G	13204	A	ND5	Non-synonymous	V290I	0	1	0.064
Modern terminal	JT	G	13204	A	ND5	Non-synonymous	V290I	0	1	0.064
Modern terminal	JT	G	13204	A	ND5	Non-synonymous	V290I	0	1	0.064
Modern terminal	JT	A	13221	C	ND5	Non-synonymous	Q295H	0.998	0	0.546
Modern terminal	U	G	13225	A	ND5	Non-synonymous	D297N	0.998	0	0.553
Ancient terminal	X	G	13225	A	ND5	Non-synonymous	D297N	0.998	0	0.553
Modern terminal	R0	T	13246	C	ND5	Non-synonymous	F304L	1	0.1	0.688
Pre-terminal	U	T	13246	C	ND5	Non-synonymous	F304L	1	0.1	0.688
Modern terminal	N1	C	13248	A	ND5	Non-synonymous	F304L	1	0.1	0.688
Modern terminal	R0	T	13249	C	ND5	Non-synonymous	S305P	0.997	0	0.854
Ancient terminal	U	T	13255	C	ND5	Non-synonymous	S307P	0.997	0	0.82
Ancient terminal	R0	G	13267	A	ND5	Nonsense				
Pre-terminal	R0	A	13276	G	ND5	Non-synonymous	M314V	0.08	0.19	0.529
Ancient terminal	U	C	13283	A	ND5	Non-synonymous	T316K	0.998	0	0.729
Modern terminal	R0	A	13285	G	ND5	Non-synonymous	I317V	0.913	0.13	0.272
Ancient terminal	U	C	13297	G	ND5	Non-synonymous	Q321E	0.991	0.01	0.482
Modern terminal	R0	T	13322	C	ND5	Non-synonymous	I329T	1	0	0.396
Modern terminal	R0	A	13327	G	ND5	Non-synonymous	T331A	0.995	0.01	0.469
Modern terminal	R0	A	13327	T	ND5	Non-synonymous	T331S	0.995	0.02	0.229
Modern terminal	R0	A	13327	G	ND5	Non-synonymous	T331A	0.995	0.01	0.469
Modern terminal	R0	A	13327	G	ND5	Non-synonymous	T331A	0.995	0.01	0.469
Modern terminal	R0	A	13327	G	ND5	Non-synonymous	T331A	0.995	0.01	0.469
Pre-terminal	R0	A	13327	G	ND5	Non-synonymous	T331A	0.995	0.01	0.469
Pre-terminal	R0	A	13327	G	ND5	Non-synonymous	T331A	0.995	0.01	0.469
Pre-terminal	R0	A	13327	G	ND5	Non-synonymous	T331A	0.995	0.01	0.469
Pre-terminal	R0	A	13327	G	ND5	Non-synonymous	T331A	0.995	0.01	0.469
Modern terminal	U	A	13327	G	ND5	Non-synonymous	T331A	0.995	0.01	0.469
Pre-terminal	U	A	13327	G	ND5	Non-synonymous	T331A	0.995	0.01	0.469

Modern terminal	JT	A	13327	G	ND5	Non-synonymous	T331A	0.995	0.01	0.469
Modern terminal	N1	A	13327	G	ND5	Non-synonymous	T331A	0.995	0.01	0.469
Pre-terminal	X	A	13327	G	ND5	Non-synonymous	T331A	0.995	0.01	0.469
Ancient terminal	JT	T	13340	C	ND5	Non-synonymous	F335S	1	0	0.746
Modern terminal	JT	T	13340	C	ND5	Non-synonymous	F335S	1	0	0.746
Ancient terminal	U	A	13342	T	ND5	Nonsense				
Pre-terminal	JT	C	13346	A	ND5	Non-synonymous	A337D	1	0	0.692
Modern terminal	JT	T	13349	C	ND5	Non-synonymous	M338T	0.845	0	0.465
Modern terminal	N2	C	13351	A	ND5	Non-synonymous	L339M	1	0	0.381
Ancient terminal	R0	G	13359	C	ND5	Non-synonymous	M341I	0.866	0.05	0.404
Modern terminal	U	G	13359	C	ND5	Non-synonymous	M341I	0.866	0.05	0.404
Modern terminal	U	T	13369	C	ND5	Non-synonymous	S345P	0.995	0.05	0.845
Pre-terminal	U	A	13375	G	ND5	Non-synonymous	I347V	*	0.01	0.298
Modern terminal	R0	T	13376	C	ND5	Non-synonymous	I347T	0.913	0	0.6
Modern terminal	U	A	13382	G	ND5	Non-synonymous	N349S	0.998	1	0.335
Modern terminal	R0	A	13387	T	ND5	Non-synonymous	N351Y	0.998	0	0.665
Ancient terminal	R0	G	13393	C	ND5	Non-synonymous	E353Q	0.997	0	0.556
Modern terminal	JT	C	13396	A	ND5	Non-synonymous	Q354K	0.946	0.01	0.582
Modern terminal	R0	G	13399	A	ND5	Non-synonymous	D355N	0.998	0.06	0.564
Ancient terminal	JT	T	13412	C	ND5	Non-synonymous	M359T	0.995	0.01	0.673
Ancient terminal	X	G	13418	A	ND5	Non-synonymous	G361E	1	0	0.825
Ancient terminal	U	T	13421	A	ND5	Non-synonymous	L362Q	1	0	0.775
Pre-terminal	JT	C	13430	T	ND5	Non-synonymous	T365I	0.002	0.62	0.421
Modern terminal	N1	C	13430	T	ND5	Non-synonymous	T365I	0.002	0.62	0.421
Ancient terminal	R0	T	13433	C	ND5	Non-synonymous	M366T	0.751	0.12	0.567
Ancient terminal	R0	G	13466	C	ND5	Non-synonymous	S377T	0.07	0.01	0.162
Pre-terminal	R0	G	13466	C	ND5	Non-synonymous	S377T	0.07	0.01	0.162
Modern terminal	U	G	13466	A	ND5	Non-synonymous	S377N	0	0.16	0.089
Modern terminal	R0	C	13468	A	ND5	Non-synonymous	L378M	1	0.07	0.273
Pre-terminal	R0	C	13468	A	ND5	Non-synonymous	L378M	1	0.07	0.273
Modern terminal	R0	T	13469	A	ND5	Non-synonymous	L378Q	1	0	0.741
Modern terminal	JT	T	13469	A	ND5	Non-synonymous	L378Q	1	0	0.741
Modern terminal	R0	G	13471	A	ND5	Non-synonymous	A379T	0.994	0.02	0.364
Modern terminal	R0	G	13471	A	ND5	Non-synonymous	A379T	0.994	0.02	0.364
Pre-terminal	R0	G	13471	A	ND5	Non-synonymous	A379T	0.994	0.02	0.364
Modern terminal	R0	G	13477	A	ND5	Non-synonymous	A381T	0.001	1	0.051
Pre-terminal	R0	G	13477	A	ND5	Non-synonymous	A381T	0.001	1	0.051
Modern terminal	U	G	13477	A	ND5	Non-synonymous	A381T	0.001	1	0.051
Modern terminal	U	G	13477	A	ND5	Non-synonymous	A381T	0.001	1	0.051
Modern terminal	U	C	13478	T	ND5	Non-synonymous	A381V	0.097	0.02	0.171
Modern terminal	R0	T	13490	A	ND5	Non-synonymous	F385Y	0.929	0.08	0.439
Ancient terminal	U	C	13508	T	ND5	Non-synonymous	S391F	0.999	0	0.728
Ancient terminal	R0	C	13516	T	ND5	Non-synonymous	H394Y	0.002	0.24	0.256
Modern terminal	R0	C	13524	A	ND5	Non-synonymous	I396M	1	0.01	0.422
Modern terminal	R0	G	13525	A	ND5	Non-synonymous	E397K	0.986	0	0.699
Modern terminal	R0	A	13528	G	ND5	Non-synonymous	T398A	0.986	1	0.216
Modern terminal	R0	A	13528	G	ND5	Non-synonymous	T398A	0.986	1	0.216
Modern terminal	U	A	13528	G	ND5	Non-synonymous	T398A	0.986	1	0.216
Modern terminal	U	A	13528	G	ND5	Non-synonymous	T398A	0.986	1	0.216
Modern terminal	JT	A	13528	G	ND5	Non-synonymous	T398A	0.986	1	0.216

Modern terminal	JT	A	13528	G	ND5	Non-synonymous	T398A	0.986	1	0.216
Pre-terminal	JT	A	13528	G	ND5	Non-synonymous	T398A	0.986	1	0.216
Ancient terminal	U	G	13531	A	ND5	Non-synonymous	A399T	0.995	0.06	0.16
Modern terminal	R0	A	13535	G	ND5	Non-synonymous	N400S	0.022	0.18	0.396
Modern terminal	R0	A	13535	G	ND5	Non-synonymous	N400S	0.022	0.18	0.396
Pre-terminal	R0	A	13535	G	ND5	Non-synonymous	N400S	0.022	0.18	0.396
Pre-terminal	R0	A	13535	G	ND5	Non-synonymous	N400S	0.022	0.18	0.396
Modern terminal	JT	A	13535	G	ND5	Non-synonymous	N400S	0.022	0.18	0.396
Ancient terminal	U	T	13543	C	ND5	Non-synonymous	Y403H	0.067	0.36	0.602
Modern terminal	U	T	13543	C	ND5	Non-synonymous	Y403H	0.067	0.36	0.602
Pre-terminal	U	T	13543	C	ND5	Non-synonymous	Y403H	0.067	0.36	0.602
Modern terminal	U	C	13547	T	ND5	Non-synonymous	T404M	0.021	0.1	0.402
Pre-terminal	U	C	13547	T	ND5	Non-synonymous	T404M	0.021	0.1	0.402
Modern terminal	JT	C	13547	T	ND5	Non-synonymous	T404M	0.021	0.1	0.402
Pre-terminal	R0	G	13552	A	ND5	Non-synonymous	A406T	0.006	0.3	0.513
Ancient terminal	U	T	13562	G	ND5	Non-synonymous	L409R	0.998	0	0.827
Modern terminal	U	T	13564	C	ND5	Non-synonymous	S410P	0.99	0.06	0.703
Modern terminal	U	C	13565	T	ND5	Non-synonymous	S410F	0.999	0.26	0.513
Pre-terminal	U	C	13565	T	ND5	Non-synonymous	S410F	0.999	0.26	0.513
Modern terminal	JT	C	13565	T	ND5	Non-synonymous	S410F	0.999	0.26	0.513
Modern terminal	R0	A	13576	G	ND5	Non-synonymous	I414V	0.887	0.26	0.148
Modern terminal	JT	A	13576	G	ND5	Non-synonymous	I414V	0.887	0.26	0.148
Ancient terminal	JT	G	13579	A	ND5	Non-synonymous	A415T	0.998	0	0.469
Ancient terminal	U	A	13594	G	ND5	Non-synonymous	S420G	0.042	0.03	0.337
Modern terminal	JT	A	13594	G	ND5	Non-synonymous	S420G	0.042	0.03	0.337
Modern terminal	R0	G	13604	C	ND5	Non-synonymous	S423T	0.942	0	0.37
Modern terminal	JT	A	13606	G	ND5	Non-synonymous	T424A	0.997	0.16	0.411
Modern terminal	JT	A	13606	G	ND5	Non-synonymous	T424A	0.997	0.16	0.411
Ancient terminal	X	C	13607	T	ND5	Non-synonymous	T424I	1	0.46	0.498
Ancient terminal	X	C	13609	T	ND5	Non-synonymous	R425W	1	0	0.604
Pre-terminal	JT	A	13612	T	ND5	Non-synonymous	M426L	0	0.48	0.175
Modern terminal	R0	A	13615	G	ND5	Non-synonymous	I427V	0.008	0.2	0.22
Modern terminal	U	A	13615	G	ND5	Non-synonymous	I427V	0.008	0.2	0.22
Ancient terminal	JT	A	13615	G	ND5	Non-synonymous	I427V	0.008	0.2	0.22
Modern terminal	JT	A	13615	G	ND5	Non-synonymous	I427V	0.008	0.2	0.22
Modern terminal	R0	C	13618	A	ND5	Non-synonymous	L428I	0.994	0.07	0.112
Modern terminal	R0	G	13633	A	ND5	Non-synonymous	G433S	0.028	0.17	0.409
Modern terminal	U	A	13637	G	ND5	Non-synonymous	Q434R	0.001	0.38	0.23
Modern terminal	U	A	13637	G	ND5	Non-synonymous	Q434R	0.001	0.38	0.23
Pre-terminal	U	A	13637	G	ND5	Non-synonymous	Q434R	0.001	0.38	0.23
Modern terminal	JT	T	13646	A	ND5	Non-synonymous	F437Y	0.589	0.33	0.349
Modern terminal	R0	A	13651	G	ND5	Non-synonymous	T439A	0.992	0.21	0.195
Modern terminal	R0	A	13651	G	ND5	Non-synonymous	T439A	0.992	0.21	0.195
Modern terminal	R0	A	13651	C	ND5	Non-synonymous	T439P	1	0.98	0.566
Modern terminal	R0	A	13651	G	ND5	Non-synonymous	T439A	0.992	0.21	0.195
Modern terminal	R0	A	13651	G	ND5	Non-synonymous	T439A	0.992	0.21	0.195
Pre-terminal	R0	A	13651	G	ND5	Non-synonymous	T439A	0.992	0.21	0.195
Pre-terminal	R0	A	13651	G	ND5	Non-synonymous	T439A	0.992	0.21	0.195
Pre-terminal	R0	A	13651	G	ND5	Non-synonymous	T439A	0.992	0.21	0.195
Pre-terminal	R0	A	13651	C	ND5	Non-synonymous	T439P	1	0.98	0.566

Pre-terminal	R0	A	13651	G	ND5	Non-synonymous	T439A	0.992	0.21	0.195
Pre-terminal	R0	A	13651	G	ND5	Non-synonymous	T439A	0.992	0.21	0.195
Modern terminal	U	A	13651	G	ND5	Non-synonymous	T439A	0.992	0.21	0.195
Modern terminal	U	A	13651	G	ND5	Non-synonymous	T439A	0.992	0.21	0.195
Modern terminal	U	A	13651	G	ND5	Non-synonymous	T439A	0.992	0.21	0.195
Pre-terminal	U	A	13651	G	ND5	Non-synonymous	T439A	0.992	0.21	0.195
Pre-terminal	U	A	13651	G	ND5	Non-synonymous	T439A	0.992	0.21	0.195
Pre-terminal	U	A	13651	G	ND5	Non-synonymous	T439A	0.992	0.21	0.195
Pre-terminal	U	A	13651	G	ND5	Non-synonymous	T439A	0.992	0.21	0.195
Ancient terminal	JT	A	13651	C	ND5	Non-synonymous	T439P	1	0.98	0.566
Modern terminal	JT	A	13651	C	ND5	Non-synonymous	T439P	1	0.98	0.566
Modern terminal	JT	A	13651	G	ND5	Non-synonymous	T439A	0.992	0.21	0.195
Modern terminal	JT	A	13651	G	ND5	Non-synonymous	T439A	0.992	0.21	0.195
Modern terminal	JT	A	13651	G	ND5	Non-synonymous	T439A	0.992	0.21	0.195
Modern terminal	JT	A	13651	G	ND5	Non-synonymous	T439A	0.992	0.21	0.195
Modern terminal	JT	A	13651	G	ND5	Non-synonymous	T439A	0.992	0.21	0.195
Pre-terminal	JT	A	13651	G	ND5	Non-synonymous	T439A	0.992	0.21	0.195
Modern terminal	N1	A	13651	G	ND5	Non-synonymous	T439A	0.992	0.21	0.195
Ancient terminal	X	A	13651	G	ND5	Non-synonymous	T439A	0.992	0.21	0.195
Pre-terminal	U	C	13654	T	ND5	Non-synonymous	L440F	0.003	0.1	0.244
Ancient terminal	R0	C	13658	T	ND5	Non-synonymous	T441I	0.004	0.39	0.15
Modern terminal	R0	C	13658	T	ND5	Non-synonymous	T441I	0.004	0.39	0.15
Modern terminal	R0	C	13658	T	ND5	Non-synonymous	T441I	0.004	0.39	0.15
Modern terminal	U	A	13661	G	ND5	Non-synonymous	N442S	0.045	0.43	0.153
Ancient terminal	U	A	13675	G	ND5	Non-synonymous	N447D	0.005	0.09	0.329
Ancient terminal	U	A	13675	T	ND5	Non-synonymous	N447Y	0.01	0.14	0.49
Pre-terminal	JT	A	13675	G	ND5	Non-synonymous	N447D	0.005	0.09	0.329
Pre-terminal	JT	A	13675	G	ND5	Non-synonymous	N447D	0.005	0.09	0.329
Modern terminal	R0	A	13676	G	ND5	Non-synonymous	N447S	0.605	0.06	0.242
Ancient terminal	U	A	13676	T	ND5	Non-synonymous	N447I	0.807	0.03	0.43
Modern terminal	U	A	13676	G	ND5	Non-synonymous	N447S	0.605	0.06	0.242
Ancient terminal	N2	C	13677	G	ND5	Non-synonymous	N447K	0.762	0.07	0.442
Modern terminal	R0	C	13679	T	ND5	Non-synonymous	P448L	0.872	0.02	0.356
Modern terminal	R0	C	13679	T	ND5	Non-synonymous	P448L	0.872	0.02	0.356
Modern terminal	R0	A	13681	G	ND5	Non-synonymous	T449A	0.003	0.52	0.106
Modern terminal	R0	A	13681	G	ND5	Non-synonymous	T449A	0.003	0.52	0.106
Modern terminal	R0	A	13681	G	ND5	Non-synonymous	T449A	0.003	0.52	0.106
Modern terminal	R0	A	13681	G	ND5	Non-synonymous	T449A	0.003	0.52	0.106
Modern terminal	R0	A	13681	G	ND5	Non-synonymous	T449A	0.003	0.52	0.106
Modern terminal	R0	A	13681	G	ND5	Non-synonymous	T449A	0.003	0.52	0.106
Pre-terminal	R0	A	13681	G	ND5	Non-synonymous	T449A	0.003	0.52	0.106
Pre-terminal	R0	A	13681	G	ND5	Non-synonymous	T449A	0.003	0.52	0.106
Modern terminal	U	A	13681	G	ND5	Non-synonymous	T449A	0.003	0.52	0.106
Modern terminal	U	A	13681	G	ND5	Non-synonymous	T449A	0.003	0.52	0.106
Modern terminal	U	A	13681	G	ND5	Non-synonymous	T449A	0.003	0.52	0.106
Pre-terminal	U	A	13681	G	ND5	Non-synonymous	T449A	0.003	0.52	0.106
Pre-terminal	U	A	13681	G	ND5	Non-synonymous	T449A	0.003	0.52	0.106
Modern terminal	JT	A	13681	G	ND5	Non-synonymous	T449A	0.003	0.52	0.106
Modern terminal	JT	A	13681	G	ND5	Non-synonymous	T449A	0.003	0.52	0.106
Modern terminal	JT	A	13681	G	ND5	Non-synonymous	T449A	0.003	0.52	0.106

Modern terminal	JT	A	13681	G	ND5	Non-synonymous	T449A	0.003	0.52	0.106
Modern terminal	JT	A	13681	G	ND5	Non-synonymous	T449A	0.003	0.52	0.106
Modern terminal	JT	A	13681	G	ND5	Non-synonymous	T449A	0.003	0.52	0.106
Modern terminal	JT	A	13681	G	ND5	Non-synonymous	T449A	0.003	0.52	0.106
Pre-terminal	JT	A	13681	G	ND5	Non-synonymous	T449A	0.003	0.52	0.106
Pre-terminal	JT	A	13681	G	ND5	Non-synonymous	T449A	0.003	0.52	0.106
Modern terminal	N1	A	13681	G	ND5	Non-synonymous	T449A	0.003	0.52	0.106
Modern terminal	N1	A	13681	G	ND5	Non-synonymous	T449A	0.003	0.52	0.106
Modern terminal	N1	A	13681	G	ND5	Non-synonymous	T449A	0.003	0.52	0.106
Modern terminal	N1	A	13681	G	ND5	Non-synonymous	T449A	0.003	0.52	0.106
Modern terminal	N1	A	13681	T	ND5	Non-synonymous	T449S	0.012	0.41	0.22
Pre-terminal	N1	A	13681	G	ND5	Non-synonymous	T449A	0.003	0.52	0.106
Modern terminal	X	A	13681	G	ND5	Non-synonymous	T449A	0.003	0.52	0.106
Modern terminal	X	A	13681	G	ND5	Non-synonymous	T449A	0.003	0.52	0.106
Modern terminal	X	A	13681	G	ND5	Non-synonymous	T449A	0.003	0.52	0.106
Pre-terminal	X	A	13681	G	ND5	Non-synonymous	T449A	0.003	0.52	0.106
Modern terminal	R0	C	13682	T	ND5	Non-synonymous	T449I	0.68	0.39	0.285
Ancient terminal	JT	T	13685	C	ND5	Non-synonymous	L450P	1	0	0.912
Ancient terminal	U	C	13693	A	ND5	Non-synonymous	P453T	0.999	0.08	0.589
Ancient terminal	JT	C	13693	A	ND5	Non-synonymous	P453T	0.999	0.08	0.589
Modern terminal	JT	A	13696	G	ND5	Non-synonymous	I454V	0.913	0.01	0.219
Modern terminal	JT	C	13702	G	ND5	Non-synonymous	R456G	1	0	0.776
Ancient terminal	R0	G	13708	A	ND5	Non-synonymous	A458T	0	0.45	0.067
Ancient terminal	R0	G	13708	A	ND5	Non-synonymous	A458T	0	0.45	0.067
Modern terminal	R0	G	13708	A	ND5	Non-synonymous	A458T	0	0.45	0.067
Modern terminal	R0	G	13708	A	ND5	Non-synonymous	A458T	0	0.45	0.067
Modern terminal	R0	G	13708	A	ND5	Non-synonymous	A458T	0	0.45	0.067
Modern terminal	R0	G	13708	A	ND5	Non-synonymous	A458T	0	0.45	0.067
Modern terminal	R0	G	13708	A	ND5	Non-synonymous	A458T	0	0.45	0.067
Modern terminal	R0	G	13708	A	ND5	Non-synonymous	A458T	0	0.45	0.067
Modern terminal	R0	G	13708	A	ND5	Non-synonymous	A458T	0	0.45	0.067
Modern terminal	R0	G	13708	A	ND5	Non-synonymous	A458T	0	0.45	0.067
Modern terminal	R0	G	13708	A	ND5	Non-synonymous	A458T	0	0.45	0.067
Modern terminal	R0	G	13708	A	ND5	Non-synonymous	A458T	0	0.45	0.067
Modern terminal	R0	G	13708	A	ND5	Non-synonymous	A458T	0	0.45	0.067
Modern terminal	R0	G	13708	A	ND5	Non-synonymous	A458T	0	0.45	0.067
Modern terminal	R0	G	13708	A	ND5	Non-synonymous	A458T	0	0.45	0.067
Modern terminal	R0	G	13708	A	ND5	Non-synonymous	A458T	0	0.45	0.067
Modern terminal	R0	G	13708	A	ND5	Non-synonymous	A458T	0	0.45	0.067
Modern terminal	R0	G	13708	A	ND5	Non-synonymous	A458T	0	0.45	0.067
Modern terminal	R0	G	13708	A	ND5	Non-synonymous	A458T	0	0.45	0.067
Modern terminal	R0	G	13708	A	ND5	Non-synonymous	A458T	0	0.45	0.067
Modern terminal	R0	G	13708	A	ND5	Non-synonymous	A458T	0	0.45	0.067
Modern terminal	R0	G	13708	A	ND5	Non-synonymous	A458T	0	0.45	0.067
Modern terminal	R0	G	13708	A	ND5	Non-synonymous	A458T	0	0.45	0.067
Modern terminal	R0	G	13708	A	ND5	Non-synonymous	A458T	0	0.45	0.067
Modern terminal	R0	G	13708	A	ND5	Non-synonymous	A458T	0	0.45	0.067
Modern terminal	R0	G	13708	A	ND5	Non-synonymous	A458T	0	0.45	0.067
Modern terminal	R0	G	13708	A	ND5	Non-synonymous	A458T	0	0.45	0.067
Modern terminal	R0	G	13708	A	ND5	Non-synonymous	A458T	0	0.45	0.067

Modern terminal	R0	G	13708	A	ND5	Non-synonymous	A458T	0	0.45	0.067
Modern terminal	R0	G	13708	A	ND5	Non-synonymous	A458T	0	0.45	0.067
Modern terminal	R0	G	13708	A	ND5	Non-synonymous	A458T	0	0.45	0.067
Pre-terminal	R0	G	13708	A	ND5	Non-synonymous	A458T	0	0.45	0.067
Pre-terminal	R0	G	13708	A	ND5	Non-synonymous	A458T	0	0.45	0.067
Pre-terminal	R0	G	13708	A	ND5	Non-synonymous	A458T	0	0.45	0.067
Pre-terminal	R0	G	13708	A	ND5	Non-synonymous	A458T	0	0.45	0.067
Pre-terminal	R0	G	13708	A	ND5	Non-synonymous	A458T	0	0.45	0.067
Pre-terminal	R0	G	13708	A	ND5	Non-synonymous	A458T	0	0.45	0.067
Pre-terminal	R0	G	13708	A	ND5	Non-synonymous	A458T	0	0.45	0.067
Pre-terminal	R0	G	13708	A	ND5	Non-synonymous	A458T	0	0.45	0.067
Pre-terminal	R0	G	13708	A	ND5	Non-synonymous	A458T	0	0.45	0.067
Pre-terminal	R0	G	13708	A	ND5	Non-synonymous	A458T	0	0.45	0.067
Pre-terminal	R0	G	13708	A	ND5	Non-synonymous	A458T	0	0.45	0.067
Pre-terminal	R0	G	13708	A	ND5	Non-synonymous	A458T	0	0.45	0.067
Pre-terminal	R0	G	13708	A	ND5	Non-synonymous	A458T	0	0.45	0.067
Pre-terminal	R0	G	13708	A	ND5	Non-synonymous	A458T	0	0.45	0.067
Ancient terminal	U	G	13708	A	ND5	Non-synonymous	A458T	0	0.45	0.067
Ancient terminal	U	G	13708	A	ND5	Non-synonymous	A458T	0	0.45	0.067
Ancient terminal	U	G	13708	A	ND5	Non-synonymous	A458T	0	0.45	0.067
Ancient terminal	U	G	13708	A	ND5	Non-synonymous	A458T	0	0.45	0.067
Modern terminal	U	G	13708	A	ND5	Non-synonymous	A458T	0	0.45	0.067
Modern terminal	U	G	13708	A	ND5	Non-synonymous	A458T	0	0.45	0.067
Modern terminal	U	G	13708	A	ND5	Non-synonymous	A458T	0	0.45	0.067
Modern terminal	U	G	13708	A	ND5	Non-synonymous	A458T	0	0.45	0.067
Modern terminal	U	G	13708	A	ND5	Non-synonymous	A458T	0	0.45	0.067
Modern terminal	U	G	13708	A	ND5	Non-synonymous	A458T	0	0.45	0.067
Modern terminal	U	G	13708	A	ND5	Non-synonymous	A458T	0	0.45	0.067
Modern terminal	U	G	13708	A	ND5	Non-synonymous	A458T	0	0.45	0.067
Modern terminal	U	G	13708	A	ND5	Non-synonymous	A458T	0	0.45	0.067
Modern terminal	U	G	13708	A	ND5	Non-synonymous	A458T	0	0.45	0.067
Modern terminal	U	G	13708	A	ND5	Non-synonymous	A458T	0	0.45	0.067
Modern terminal	U	G	13708	A	ND5	Non-synonymous	A458T	0	0.45	0.067
Modern terminal	U	G	13708	A	ND5	Non-synonymous	A458T	0	0.45	0.067
Modern terminal	U	G	13708	A	ND5	Non-synonymous	A458T	0	0.45	0.067
Modern terminal	U	G	13708	A	ND5	Non-synonymous	A458T	0	0.45	0.067
Modern terminal	U	G	13708	A	ND5	Non-synonymous	A458T	0	0.45	0.067
Modern terminal	U	G	13708	A	ND5	Non-synonymous	A458T	0	0.45	0.067
Modern terminal	U	G	13708	A	ND5	Non-synonymous	A458T	0	0.45	0.067
Modern terminal	U	G	13708	A	ND5	Non-synonymous	A458T	0	0.45	0.067
Modern terminal	U	G	13708	A	ND5	Non-synonymous	A458T	0	0.45	0.067
Modern terminal	U	G	13708	A	ND5	Non-synonymous	A458T	0	0.45	0.067
Modern terminal	U	G	13708	A	ND5	Non-synonymous	A458T	0	0.45	0.067
Modern terminal	U	G	13708	A	ND5	Non-synonymous	A458T	0	0.45	0.067
Modern terminal	U	G	13708	A	ND5	Non-synonymous	A458T	0	0.45	0.067
Modern terminal	U	G	13708	A	ND5	Non-synonymous	A458T	0	0.45	0.067
Modern terminal	U	G	13708	A	ND5	Non-synonymous	A458T	0	0.45	0.067
Modern terminal	U	G	13708	A	ND5	Non-synonymous	A458T	0	0.45	0.067
Modern terminal	U	G	13708	A	ND5	Non-synonymous	A458T	0	0.45	0.067
Pre-terminal	U	G	13708	A	ND5	Non-synonymous	A458T	0	0.45	0.067

Pre-terminal	U	G	13708	A	ND5	Non-synonymous	A458T	0	0.45	0.067
Pre-terminal	U	G	13708	A	ND5	Non-synonymous	A458T	0	0.45	0.067
Pre-terminal	U	G	13708	A	ND5	Non-synonymous	A458T	0	0.45	0.067
Pre-terminal	U	G	13708	A	ND5	Non-synonymous	A458T	0	0.45	0.067
Pre-terminal	U	G	13708	A	ND5	Non-synonymous	A458T	0	0.45	0.067
Pre-terminal	U	G	13708	A	ND5	Non-synonymous	A458T	0	0.45	0.067
Pre-terminal	U	G	13708	A	ND5	Non-synonymous	A458T	0	0.45	0.067
Pre-terminal	U	G	13708	A	ND5	Non-synonymous	A458T	0	0.45	0.067
Pre-terminal	U	G	13708	A	ND5	Non-synonymous	A458T	0	0.45	0.067
Pre-terminal	U	G	13708	A	ND5	Non-synonymous	A458T	0	0.45	0.067
Pre-terminal	U	G	13708	A	ND5	Non-synonymous	A458T	0	0.45	0.067
Pre-terminal	U	G	13708	A	ND5	Non-synonymous	A458T	0	0.45	0.067
Pre-terminal	U	G	13708	A	ND5	Non-synonymous	A458T	0	0.45	0.067
Pre-terminal	U	G	13708	A	ND5	Non-synonymous	A458T	0	0.45	0.067
Modern terminal	JT	G	13708	A	ND5	Non-synonymous	A458T	0	0.45	0.067
Modern terminal	JT	G	13708	A	ND5	Non-synonymous	A458T	0	0.45	0.067
Modern terminal	JT	G	13708	A	ND5	Non-synonymous	A458T	0	0.45	0.067
Modern terminal	JT	G	13708	A	ND5	Non-synonymous	A458T	0	0.45	0.067
Modern terminal	JT	G	13708	A	ND5	Non-synonymous	A458T	0	0.45	0.067
Modern terminal	JT	G	13708	A	ND5	Non-synonymous	A458T	0	0.45	0.067
Modern terminal	JT	G	13708	A	ND5	Non-synonymous	A458T	0	0.45	0.067
Modern terminal	JT	G	13708	A	ND5	Non-synonymous	A458T	0	0.45	0.067
Modern terminal	JT	G	13708	A	ND5	Non-synonymous	A458T	0	0.45	0.067
Pre-terminal	JT	G	13708	A	ND5	Non-synonymous	A458T	0	0.45	0.067
Pre-terminal	JT	G	13708	A	ND5	Non-synonymous	A458T	0	0.45	0.067
Pre-terminal	JT	G	13708	A	ND5	Non-synonymous	A458T	0	0.45	0.067
Pre-terminal	JT	G	13708	A	ND5	Non-synonymous	A458T	0	0.45	0.067
Modern terminal	N1	G	13708	A	ND5	Non-synonymous	A458T	0	0.45	0.067
Pre-terminal	N1	G	13708	A	ND5	Non-synonymous	A458T	0	0.45	0.067
Pre-terminal	N1	G	13708	A	ND5	Non-synonymous	A458T	0	0.45	0.067
Pre-terminal	N1	G	13708	A	ND5	Non-synonymous	A458T	0	0.45	0.067
Modern terminal	X	G	13708	A	ND5	Non-synonymous	A458T	0	0.45	0.067
Modern terminal	N2	G	13708	A	ND5	Non-synonymous	A458T	0	0.45	0.067
Pre-terminal	N2	G	13708	A	ND5	Non-synonymous	A458T	0	0.45	0.067
Modern terminal	R0	C	13709	T	ND5	Non-synonymous	A458V	0.004	0.81	0.294
Modern terminal	U	C	13709	T	ND5	Non-synonymous	A458V	0.004	0.81	0.294
Modern terminal	U	C	13709	T	ND5	Non-synonymous	A458V	0.004	0.81	0.294
Ancient terminal	R0	G	13711	A	ND5	Non-synonymous	A459T	0.001	0.18	0.21
Modern terminal	R0	G	13711	A	ND5	Non-synonymous	A459T	0.001	0.18	0.21
Pre-terminal	R0	G	13711	A	ND5	Non-synonymous	A459T	0.001	0.18	0.21
Pre-terminal	R0	G	13711	A	ND5	Non-synonymous	A459T	0.001	0.18	0.21
Modern terminal	U	G	13711	A	ND5	Non-synonymous	A459T	0.001	0.18	0.21
Pre-terminal	U	G	13711	A	ND5	Non-synonymous	A459T	0.001	0.18	0.21
Ancient terminal	JT	G	13711	A	ND5	Non-synonymous	A459T	0.001	0.18	0.21
Modern terminal	JT	G	13711	A	ND5	Non-synonymous	A459T	0.001	0.18	0.21
Modern terminal	JT	G	13711	A	ND5	Non-synonymous	A459T	0.001	0.18	0.21
Modern terminal	JT	G	13711	A	ND5	Non-synonymous	A459T	0.001	0.18	0.21
Modern terminal	U	G	13718	C	ND5	Non-synonymous	S461T	0.908	0.14	0.475
Pre-terminal	R0	C	13735	A	ND5	Non-synonymous	L467I	0.192	1	0.287
Modern terminal	JT	C	13735	T	ND5	Non-synonymous	L467F	0.003	0.35	0.508

Modern terminal	R0	A	13745	G	ND5	Non-synonymous	N470S	0.045	0.57	0.214
Pre-terminal	U	A	13745	G	ND5	Non-synonymous	N470S	0.045	0.57	0.214
Modern terminal	X	A	13745	G	ND5	Non-synonymous	N470S	0.045	0.57	0.214
Modern terminal	R0	A	13748	G	ND5	Non-synonymous	N471S	0.045	0.51	0.159
Modern terminal	R0	A	13748	G	ND5	Non-synonymous	N471S	0.045	0.51	0.159
Modern terminal	R0	A	13748	G	ND5	Non-synonymous	N471S	0.045	0.51	0.159
Modern terminal	R0	A	13748	G	ND5	Non-synonymous	N471S	0.045	0.51	0.159
Pre-terminal	R0	A	13748	G	ND5	Non-synonymous	N471S	0.045	0.51	0.159
Modern terminal	U	A	13748	G	ND5	Non-synonymous	N471S	0.045	0.51	0.159
Modern terminal	N1	A	13748	G	ND5	Non-synonymous	N471S	0.045	0.51	0.159
Pre-terminal	N1	A	13748	G	ND5	Non-synonymous	N471S	0.045	0.51	0.159
Pre-terminal	N2	A	13748	G	ND5	Non-synonymous	N471S	0.045	0.51	0.159
Ancient terminal	JT	T	13751	G	ND5	Non-synonymous	I472S	1	0	0.504
Pre-terminal	R0	T	13753	C	ND5	Non-synonymous	S473P	0	0.34	0.087
Pre-terminal	R0	T	13753	C	ND5	Non-synonymous	S473P	0	0.34	0.087
Modern terminal	U	T	13753	C	ND5	Non-synonymous	S473P	0	0.34	0.087
Modern terminal	U	T	13753	C	ND5	Non-synonymous	S473P	0	0.34	0.087
Modern terminal	JT	T	13753	C	ND5	Non-synonymous	S473P	0	0.34	0.087
Pre-terminal	JT	T	13753	C	ND5	Non-synonymous	S473P	0	0.34	0.087
Modern terminal	X	T	13753	C	ND5	Non-synonymous	S473P	0	0.34	0.087
Modern terminal	N2	T	13753	C	ND5	Non-synonymous	S473P	0	0.34	0.087
Modern terminal	U	C	13754	T	ND5	Non-synonymous	S473F	0.298	0.55	0.198
Modern terminal	R0	G	13759	A	ND5	Non-synonymous	A475T	0	0.77	0.044
Modern terminal	R0	G	13759	A	ND5	Non-synonymous	A475T	0	0.77	0.044
Modern terminal	R0	G	13759	A	ND5	Non-synonymous	A475T	0	0.77	0.044
Modern terminal	R0	G	13759	A	ND5	Non-synonymous	A475T	0	0.77	0.044
Modern terminal	R0	G	13759	A	ND5	Non-synonymous	A475T	0	0.77	0.044
Modern terminal	R0	G	13759	A	ND5	Non-synonymous	A475T	0	0.77	0.044
Modern terminal	R0	G	13759	A	ND5	Non-synonymous	A475T	0	0.77	0.044
Modern terminal	R0	G	13759	A	ND5	Non-synonymous	A475T	0	0.77	0.044
Modern terminal	R0	G	13759	A	ND5	Non-synonymous	A475T	0	0.77	0.044
Modern terminal	R0	G	13759	A	ND5	Non-synonymous	A475T	0	0.77	0.044
Modern terminal	R0	G	13759	A	ND5	Non-synonymous	A475T	0	0.77	0.044
Modern terminal	R0	G	13759	A	ND5	Non-synonymous	A475T	0	0.77	0.044
Modern terminal	R0	G	13759	A	ND5	Non-synonymous	A475T	0	0.77	0.044
Modern terminal	R0	G	13759	A	ND5	Non-synonymous	A475T	0	0.77	0.044
Modern terminal	R0	G	13759	A	ND5	Non-synonymous	A475T	0	0.77	0.044
Modern terminal	R0	G	13759	A	ND5	Non-synonymous	A475T	0	0.77	0.044
Modern terminal	R0	G	13759	A	ND5	Non-synonymous	A475T	0	0.77	0.044
Modern terminal	R0	G	13759	A	ND5	Non-synonymous	A475T	0	0.77	0.044
Modern terminal	R0	G	13759	A	ND5	Non-synonymous	A475T	0	0.77	0.044
Modern terminal	R0	G	13759	A	ND5	Non-synonymous	A475T	0	0.77	0.044
Pre-terminal	R0	G	13759	A	ND5	Non-synonymous	A475T	0	0.77	0.044
Pre-terminal	R0	G	13759	A	ND5	Non-synonymous	A475T	0	0.77	0.044
Pre-terminal	R0	G	13759	A	ND5	Non-synonymous	A475T	0	0.77	0.044
Pre-terminal	R0	G	13759	A	ND5	Non-synonymous	A475T	0	0.77	0.044
Pre-terminal	R0	G	13759	A	ND5	Non-synonymous	A475T	0	0.77	0.044
Pre-terminal	R0	G	13759	A	ND5	Non-synonymous	A475T	0	0.77	0.044
Ancient terminal	U	G	13759	A	ND5	Non-synonymous	A475T	0	0.77	0.044
Ancient terminal	U	G	13759	A	ND5	Non-synonymous	A475T	0	0.77	0.044

Modern terminal	U	G	13759	A	ND5	Non-synonymous	A475T	0	0.77	0.044
Modern terminal	U	G	13759	A	ND5	Non-synonymous	A475T	0	0.77	0.044
Modern terminal	U	G	13759	A	ND5	Non-synonymous	A475T	0	0.77	0.044
Modern terminal	U	G	13759	A	ND5	Non-synonymous	A475T	0	0.77	0.044
Modern terminal	U	G	13759	A	ND5	Non-synonymous	A475T	0	0.77	0.044
Modern terminal	U	G	13759	A	ND5	Non-synonymous	A475T	0	0.77	0.044
Modern terminal	U	G	13759	A	ND5	Non-synonymous	A475T	0	0.77	0.044
Modern terminal	U	G	13759	A	ND5	Non-synonymous	A475T	0	0.77	0.044
Modern terminal	U	G	13759	A	ND5	Non-synonymous	A475T	0	0.77	0.044
Pre-terminal	U	G	13759	A	ND5	Non-synonymous	A475T	0	0.77	0.044
Pre-terminal	U	G	13759	A	ND5	Non-synonymous	A475T	0	0.77	0.044
Pre-terminal	U	G	13759	A	ND5	Non-synonymous	A475T	0	0.77	0.044
Pre-terminal	U	G	13759	A	ND5	Non-synonymous	A475T	0	0.77	0.044
Pre-terminal	U	G	13759	A	ND5	Non-synonymous	A475T	0	0.77	0.044
Modern terminal	JT	G	13759	A	ND5	Non-synonymous	A475T	0	0.77	0.044
Modern terminal	JT	G	13759	A	ND5	Non-synonymous	A475T	0	0.77	0.044
Modern terminal	JT	G	13759	A	ND5	Non-synonymous	A475T	0	0.77	0.044
Modern terminal	JT	G	13759	A	ND5	Non-synonymous	A475T	0	0.77	0.044
Pre-terminal	JT	G	13759	A	ND5	Non-synonymous	A475T	0	0.77	0.044
Pre-terminal	JT	G	13759	A	ND5	Non-synonymous	A475T	0	0.77	0.044
Pre-terminal	JT	G	13759	A	ND5	Non-synonymous	A475T	0	0.77	0.044
Pre-terminal	JT	G	13759	A	ND5	Non-synonymous	A475T	0	0.77	0.044
Pre-terminal	JT	G	13759	A	ND5	Non-synonymous	A475T	0	0.77	0.044
Ancient terminal	N1	G	13759	A	ND5	Non-synonymous	A475T	0	0.77	0.044
Ancient terminal	N1	G	13759	A	ND5	Non-synonymous	A475T	0	0.77	0.044
Modern terminal	N2	G	13759	A	ND5	Non-synonymous	A475T	0	0.77	0.044
Modern terminal	R0	C	13760	T	ND5	Non-synonymous	A475V	0	0.57	0.13
Modern terminal	U	C	13760	T	ND5	Non-synonymous	A475V	0	0.57	0.13
Modern terminal	JT	C	13760	T	ND5	Non-synonymous	A475V	0	0.57	0.13
Pre-terminal	R0	T	13762	G	ND5	Non-synonymous	S476A	0.063	0.41	0.13
Pre-terminal	U	C	13763	T	ND5	Non-synonymous	S476F	0.011	0.1	0.247
Pre-terminal	R0	C	13765	G	ND5	Non-synonymous	P477A	0.009	0.47	0.125
Modern terminal	JT	C	13765	T	ND5	Non-synonymous	P477S	0.137	0.22	0.133
Modern terminal	JT	C	13766	A	ND5	Non-synonymous	P477H	0.801	0.04	0.14
Modern terminal	R0	T	13768	C	ND5	Non-synonymous	F478L	0.01	0.24	0.091
Modern terminal	R0	T	13768	C	ND5	Non-synonymous	F478L	0.01	0.24	0.091
Modern terminal	R0	T	13768	C	ND5	Non-synonymous	F478L	0.01	0.24	0.091
Modern terminal	R0	T	13768	C	ND5	Non-synonymous	F478L	0.01	0.24	0.091
Modern terminal	R0	T	13768	C	ND5	Non-synonymous	F478L	0.01	0.24	0.091
Modern terminal	R0	T	13768	C	ND5	Non-synonymous	F478L	0.01	0.24	0.091
Modern terminal	R0	T	13768	C	ND5	Non-synonymous	F478L	0.01	0.24	0.091
Pre-terminal	R0	T	13768	C	ND5	Non-synonymous	F478L	0.01	0.24	0.091
Pre-terminal	R0	T	13768	C	ND5	Non-synonymous	F478L	0.01	0.24	0.091
Pre-terminal	R0	T	13768	C	ND5	Non-synonymous	F478L	0.01	0.24	0.091
Modern terminal	U	T	13768	C	ND5	Non-synonymous	F478L	0.01	0.24	0.091
Pre-terminal	U	T	13768	C	ND5	Non-synonymous	F478L	0.01	0.24	0.091
Pre-terminal	U	T	13768	C	ND5	Non-synonymous	F478L	0.01	0.24	0.091
Pre-terminal	U	T	13768	C	ND5	Non-synonymous	F478L	0.01	0.24	0.091
Ancient terminal	JT	T	13768	A	ND5	Non-synonymous	F478I	0.004	0.22	0.148
Modern terminal	JT	T	13768	A	ND5	Non-synonymous	F478I	0.01	0.22	0.148

Modern terminal	JT	T	13768	C	ND5	Non-synonymous	F478L	0.01	0.24	0.091
Modern terminal	JT	T	13768	C	ND5	Non-synonymous	F478L	0.01	0.24	0.091
Modern terminal	JT	T	13768	C	ND5	Non-synonymous	F478L	0.01	0.24	0.091
Modern terminal	JT	T	13768	C	ND5	Non-synonymous	F478L	0.01	0.24	0.091
Pre-terminal	JT	T	13768	C	ND5	Non-synonymous	F478L	0.01	0.24	0.091
Modern terminal	N1	T	13768	C	ND5	Non-synonymous	F478L	0.01	0.24	0.091
Pre-terminal	N1	T	13768	C	ND5	Non-synonymous	F478L	0.01	0.24	0.091
Modern terminal	R0	C	13770	A	ND5	Non-synonymous	F478L	0.01	0.24	0.091
Pre-terminal	U	C	13770	A	ND5	Non-synonymous	F478L	0.01	0.24	0.091
Pre-terminal	JT	A	13773	T	ND5	Non-synonymous	Q479H	0.991	0.17	0.186
Modern terminal	R0	A	13780	G	ND5	Non-synonymous	I482V	0.001	0.01	0.053
Modern terminal	R0	A	13780	G	ND5	Non-synonymous	I482V	0.001	0.01	0.053
Modern terminal	N1	A	13780	C	ND5	Non-synonymous	I482L	0.002	0.19	0.141
Modern terminal	N1	A	13780	G	ND5	Non-synonymous	I482V	0.001	0.01	0.053
Modern terminal	R0	T	13781	C	ND5	Non-synonymous	I482T	0.024	0.04	0.115
Pre-terminal	R0	T	13781	C	ND5	Non-synonymous	I482T	0.024	0.04	0.115
Pre-terminal	R0	T	13781	C	ND5	Non-synonymous	I482T	0.024	0.04	0.115
Modern terminal	U	T	13781	C	ND5	Non-synonymous	I482T	0.024	0.04	0.115
Modern terminal	U	T	13781	C	ND5	Non-synonymous	I482T	0.024	0.04	0.115
Pre-terminal	U	T	13781	C	ND5	Non-synonymous	I482T	0.024	0.04	0.115
Pre-terminal	U	T	13781	C	ND5	Non-synonymous	I482T	0.024	0.04	0.115
Pre-terminal	U	C	13782	A	ND5	Non-synonymous	I482M	0.089	1	0.047
Ancient terminal	U	C	13783	T	ND5	Non-synonymous	P483S	0.999	0.09	0.198
Ancient terminal	U	C	13784	T	ND5	Non-synonymous	P483L	1	0	0.246
Ancient terminal	R0	C	13786	A	ND5	Non-synonymous	L484I	0.885	0.4	0.068
Ancient terminal	U	C	13786	A	ND5	Non-synonymous	L484I	0.885	0.4	0.068
Pre-terminal	U	T	13789	C	ND5	Non-synonymous	Y485H	1	0.52	0.234
Modern terminal	X	T	13789	C	ND5	Non-synonymous	Y485H	1	0.52	0.234
Modern terminal	R0	A	13790	G	ND5	Non-synonymous	Y485C	1	0.11	0.255
Modern terminal	R0	A	13790	G	ND5	Non-synonymous	Y485C	1	0.11	0.255
Pre-terminal	U	A	13790	G	ND5	Non-synonymous	Y485C	1	0.11	0.255
Modern terminal	N1	A	13790	G	ND5	Non-synonymous	Y485C	1	0.11	0.255
Pre-terminal	N2	A	13790	G	ND5	Non-synonymous	Y485C	1	0.11	0.255
Modern terminal	R0	A	13801	G	ND5	Non-synonymous	T489A	0.004	1	0.072
Modern terminal	R0	A	13801	G	ND5	Non-synonymous	T489A	0.004	1	0.072
Modern terminal	R0	A	13801	G	ND5	Non-synonymous	T489A	0.004	1	0.072
Pre-terminal	R0	A	13801	G	ND5	Non-synonymous	T489A	0.004	1	0.072
Modern terminal	JT	C	13802	T	ND5	Non-synonymous	T489M	0.983	0.36	0.081
Modern terminal	R0	G	13804	A	ND5	Non-synonymous	A490T	0.995	0	0.25
Modern terminal	X	G	13804	A	ND5	Non-synonymous	A490T	0.995	0	0.25
Modern terminal	X	C	13807	T	ND5	Non-synonymous	L491F	0.998	0.01	0.415
Modern terminal	X	C	13807	T	ND5	Non-synonymous	L491F	0.998	0.01	0.415
Pre-terminal	X	C	13807	T	ND5	Non-synonymous	L491F	0.998	0.01	0.415
Pre-terminal	R0	G	13810	A	ND5	Non-synonymous	A492T	0	0.56	0.119
Modern terminal	U	G	13810	A	ND5	Non-synonymous	A492T	0	0.56	0.119
Modern terminal	N1	G	13810	A	ND5	Non-synonymous	A492T	0	0.56	0.119
Modern terminal	R0	G	13813	A	ND5	Non-synonymous	V493I	0	0.13	0.066
Pre-terminal	R0	G	13813	A	ND5	Non-synonymous	V493I	0	0.13	0.066
Pre-terminal	JT	G	13813	A	ND5	Non-synonymous	V493I	0	0.13	0.066
Pre-terminal	N1	G	13813	A	ND5	Non-synonymous	V493I	0	0.13	0.066

Modern terminal	R0	A	13816	G	ND5	Non-synonymous	T494A	0.995	0.01	0.345
Ancient terminal	U	A	13816	G	ND5	Non-synonymous	T494A	0.995	0.01	0.345
Modern terminal	U	A	13816	G	ND5	Non-synonymous	T494A	0.995	0.01	0.345
Modern terminal	R0	T	13819	C	ND5	Non-synonymous	F495L	0.004	0.58	0.122
Modern terminal	U	T	13819	C	ND5	Non-synonymous	F495L	0.004	0.58	0.122
Pre-terminal	R0	T	13820	C	ND5	Non-synonymous	F495S	0.043	0	0.263
Pre-terminal	U	T	13820	C	ND5	Non-synonymous	F495S	0.043	0	0.263
Modern terminal	R0	C	13828	T	ND5	Non-synonymous	L498F	0.018	0.71	0.049
Modern terminal	R0	C	13828	T	ND5	Non-synonymous	L498F	0.018	0.71	0.049
Modern terminal	N2	C	13831	A	ND5	Non-synonymous	L499M	1	0.17	0.299
Modern terminal	U	A	13834	G	ND5	Non-synonymous	T500A	0.005	0.03	0.158
Modern terminal	U	A	13834	G	ND5	Non-synonymous	T500A	0.005	0.03	0.158
Modern terminal	U	A	13834	G	ND5	Non-synonymous	T500A	0.005	0.03	0.158
Pre-terminal	U	A	13834	G	ND5	Non-synonymous	T500A	0.005	0.03	0.158
Ancient terminal	N2	A	13834	G	ND5	Non-synonymous	T500A	0.005	0.03	0.158
Modern terminal	R0	C	13835	T	ND5	Non-synonymous	T500M	0.964	0.14	0.073
Pre-terminal	R0	C	13835	T	ND5	Non-synonymous	T500M	0.964	0.14	0.073
Modern terminal	U	A	13849	T	ND5	Non-synonymous	N505Y	0.008	0.76	0.328
Modern terminal	X	T	13852	C	ND5	Non-synonymous	Y506H	0.999	0.54	0.234
Pre-terminal	R0	A	13858	G	ND5	Non-synonymous	T508A	0.001	0.13	0.137
Modern terminal	JT	A	13858	G	ND5	Non-synonymous	T508A	0.001	0.13	0.137
Pre-terminal	JT	A	13858	G	ND5	Non-synonymous	T508A	0.001	0.13	0.137
Modern terminal	U	A	13861	T	ND5	Non-synonymous	N509Y	0.994	1	0.42
Modern terminal	R0	A	13862	G	ND5	Non-synonymous	N509S	0.011	0.5	0.17
Modern terminal	R0	T	13879	C	ND5	Non-synonymous	S515P	0.001	0.3	0.199
Modern terminal	R0	T	13879	C	ND5	Non-synonymous	S515P	0.001	0.3	0.199
Modern terminal	R0	T	13879	C	ND5	Non-synonymous	S515P	0.001	0.3	0.199
Modern terminal	R0	T	13879	C	ND5	Non-synonymous	S515P	0.001	0.3	0.199
Modern terminal	R0	T	13879	C	ND5	Non-synonymous	S515P	0.001	0.3	0.199
Modern terminal	R0	T	13879	C	ND5	Non-synonymous	S515P	0.001	0.3	0.199
Modern terminal	R0	T	13879	C	ND5	Non-synonymous	S515P	0.001	0.3	0.199
Modern terminal	R0	T	13879	C	ND5	Non-synonymous	S515P	0.001	0.3	0.199
Pre-terminal	R0	T	13879	A	ND5	Non-synonymous	S515T	0	0.57	0.061
Pre-terminal	R0	T	13879	C	ND5	Non-synonymous	S515P	0.001	0.3	0.199
Pre-terminal	R0	T	13879	A	ND5	Non-synonymous	S515T	0	0.57	0.061
Modern terminal	U	T	13879	C	ND5	Non-synonymous	S515P	0.001	0.3	0.199
Pre-terminal	U	T	13879	C	ND5	Non-synonymous	S515P	0.001	0.3	0.199
Ancient terminal	JT	T	13879	C	ND5	Non-synonymous	S515P	0.001	0.3	0.199
Modern terminal	N1	T	13879	C	ND5	Non-synonymous	S515P	0.001	0.3	0.199
Pre-terminal	X	T	13879	C	ND5	Non-synonymous	S515P	0.001	0.3	0.199
Modern terminal	U	C	13880	A	ND5	Non-synonymous	S515Y	0.43	1	0.137
Modern terminal	U	C	13885	G	ND5	Non-synonymous	L517V	0.083	0.5	0.101
Modern terminal	R0	T	13886	C	ND5	Non-synonymous	L517P	0.026	0.21	0.211
Modern terminal	R0	T	13886	C	ND5	Non-synonymous	L517P	0.026	0.21	0.211
Modern terminal	R0	T	13886	C	ND5	Non-synonymous	L517P	0.026	0.21	0.211
Modern terminal	R0	T	13886	C	ND5	Non-synonymous	L517P	0.026	0.21	0.211
Ancient terminal	U	T	13886	C	ND5	Non-synonymous	L517P	0.026	0.21	0.211
Modern terminal	U	T	13886	C	ND5	Non-synonymous	L517P	0.026	0.21	0.211
Modern terminal	U	T	13886	C	ND5	Non-synonymous	L517P	0.026	0.21	0.211
Modern terminal	U	T	13886	C	ND5	Non-synonymous	L517P	0.026	0.21	0.211

Pre-terminal	U	T	13886	C	ND5	Non-synonymous	L517P	0.026	0.21	0.211
Modern terminal	JT	T	13886	C	ND5	Non-synonymous	L517P	0.026	0.21	0.211
Modern terminal	JT	T	13886	C	ND5	Non-synonymous	L517P	0.026	0.21	0.211
Modern terminal	R0	G	13889	A	ND5	Non-synonymous	C518Y	0	1	0.149
Modern terminal	R0	G	13889	A	ND5	Non-synonymous	C518Y	0	1	0.149
Modern terminal	R0	G	13889	A	ND5	Non-synonymous	C518Y	0	1	0.149
Pre-terminal	R0	G	13889	A	ND5	Non-synonymous	C518Y	0	1	0.149
Pre-terminal	R0	G	13889	A	ND5	Non-synonymous	C518Y	0	1	0.149
Pre-terminal	R0	G	13889	A	ND5	Non-synonymous	C518Y	0	1	0.149
Ancient terminal	U	G	13889	A	ND5	Non-synonymous	C518Y	0	1	0.149
Ancient terminal	U	G	13889	A	ND5	Non-synonymous	C518Y	0	1	0.149
Ancient terminal	U	G	13889	T	ND5	Non-synonymous	C518F	0.002	0.71	0.146
Modern terminal	U	G	13889	A	ND5	Non-synonymous	C518Y	0	1	0.149
Modern terminal	U	G	13889	C	ND5	Non-synonymous	C518S	0.07	0.53	0.149
Modern terminal	N1	G	13889	A	ND5	Non-synonymous	C518Y	0	1	0.149
Modern terminal	X	G	13889	A	ND5	Non-synonymous	C518Y	0	1	0.149
Ancient terminal	N2	C	13890	A	ND5	Non-synonymous	C518W	0.009	0.18	0.188
Pre-terminal	R0	A	13907	G	ND5	Non-synonymous	N524S	0.815	0.59	0.304
Pre-terminal	R0	A	13907	G	ND5	Non-synonymous	N524S	0.815	0.59	0.304
Pre-terminal	R0	A	13907	G	ND5	Non-synonymous	N524S	0.815	0.59	0.304
Modern terminal	JT	A	13907	G	ND5	Non-synonymous	N524S	0.815	0.59	0.304
Pre-terminal	R0	T	13919	A	ND5	Non-synonymous	F528Y	0.986	0.85	0.175
Ancient terminal	U	C	13924	T	ND5	Non-synonymous	P530S	1	0.02	0.436
Modern terminal	R0	A	13927	T	ND5	Non-synonymous	S531C	0.996	0.18	0.213
Ancient terminal	R0	G	13928	A	ND5	Non-synonymous	S531N	0	0.67	0.108
Modern terminal	R0	G	13928	C	ND5	Non-synonymous	S531T	0.117	1	0.057
Modern terminal	R0	G	13928	A	ND5	Non-synonymous	S531N	0	0.67	0.108
Modern terminal	R0	G	13928	C	ND5	Non-synonymous	S531T	0.117	1	0.057
Modern terminal	R0	G	13928	C	ND5	Non-synonymous	S531T	0.117	1	0.057
Modern terminal	R0	G	13928	C	ND5	Non-synonymous	S531T	0.117	1	0.057
Modern terminal	R0	G	13928	A	ND5	Non-synonymous	S531N	0	0.67	0.108
Modern terminal	R0	G	13928	C	ND5	Non-synonymous	S531T	0.117	1	0.057
Modern terminal	R0	G	13928	C	ND5	Non-synonymous	S531T	0.117	1	0.057
Modern terminal	R0	G	13928	A	ND5	Non-synonymous	S531N	0	0.67	0.108
Modern terminal	R0	G	13928	C	ND5	Non-synonymous	S531T	0.117	1	0.057
Modern terminal	R0	G	13928	C	ND5	Non-synonymous	S531T	0.117	1	0.057
Modern terminal	R0	G	13928	C	ND5	Non-synonymous	S531T	0.117	1	0.057
Pre-terminal	R0	G	13928	C	ND5	Non-synonymous	S531T	0.117	1	0.057
Pre-terminal	R0	G	13928	C	ND5	Non-synonymous	S531T	0.117	1	0.057
Pre-terminal	R0	G	13928	C	ND5	Non-synonymous	S531T	0.117	1	0.057
Ancient terminal	U	G	13928	C	ND5	Non-synonymous	S531T	0.117	1	0.057
Ancient terminal	U	G	13928	C	ND5	Non-synonymous	S531T	0.117	1	0.057
Modern terminal	U	G	13928	A	ND5	Non-synonymous	S531N	0	0.67	0.108
Modern terminal	U	G	13928	A	ND5	Non-synonymous	S531N	0	0.67	0.108
Modern terminal	U	G	13928	A	ND5	Non-synonymous	S531N	0	0.67	0.108
Modern terminal	U	G	13928	A	ND5	Non-synonymous	S531N	0	0.67	0.108
Modern terminal	U	G	13928	A	ND5	Non-synonymous	S531N	0	0.67	0.108
Modern terminal	U	G	13928	C	ND5	Non-synonymous	S531T	0.117	1	0.057
Modern terminal	U	G	13928	C	ND5	Non-synonymous	S531T	0.117	1	0.057
Modern terminal	U	G	13928	C	ND5	Non-synonymous	S531T	0.117	1	0.057

Modern terminal	U	G	13928	C	ND5	Non-synonymous	S531T	0.117	1	0.057
Modern terminal	U	G	13928	C	ND5	Non-synonymous	S531T	0.117	1	0.057
Modern terminal	U	G	13928	C	ND5	Non-synonymous	S531T	0.117	1	0.057
Pre-terminal	U	G	13928	C	ND5	Non-synonymous	S531T	0.117	1	0.057
Pre-terminal	U	G	13928	A	ND5	Non-synonymous	S531N	0	0.67	0.108
Pre-terminal	U	G	13928	A	ND5	Non-synonymous	S531N	0	0.67	0.108
Pre-terminal	U	G	13928	A	ND5	Non-synonymous	S531N	0	0.67	0.108
Modern terminal	JT	G	13928	A	ND5	Non-synonymous	S531N	0	0.67	0.108
Modern terminal	JT	G	13928	A	ND5	Non-synonymous	S531N	0	0.67	0.108
Modern terminal	JT	G	13928	T	ND5	Non-synonymous	S531I	0.987	0.47	0.217
Modern terminal	JT	G	13928	T	ND5	Non-synonymous	S531I	0.987	0.47	0.217
Modern terminal	JT	G	13928	T	ND5	Non-synonymous	S531I	0.987	0.47	0.217
Pre-terminal	JT	G	13928	C	ND5	Non-synonymous	S531T	0.117	1	0.057
Pre-terminal	JT	G	13928	A	ND5	Non-synonymous	S531N	0	0.67	0.108
Pre-terminal	JT	G	13928	C	ND5	Non-synonymous	S531T	0.117	1	0.057
Pre-terminal	JT	G	13928	A	ND5	Non-synonymous	S531N	0	0.67	0.108
Modern terminal	N1	G	13928	A	ND5	Non-synonymous	S531N	0	0.67	0.108
Modern terminal	N1	G	13928	A	ND5	Non-synonymous	S531N	0	0.67	0.108
Modern terminal	N1	G	13928	C	ND5	Non-synonymous	S531T	0.117	1	0.057
Modern terminal	N1	G	13928	T	ND5	Non-synonymous	S531I	0.987	0.47	0.217
Modern terminal	N2	G	13928	C	ND5	Non-synonymous	S531T	0.117	1	0.057
Modern terminal	N2	G	13928	A	ND5	Non-synonymous	S531N	0	0.67	0.108
Modern terminal	R0	A	13933	G	ND5	Non-synonymous	T533A	0.152	0.3	0.115
Modern terminal	R0	A	13933	G	ND5	Non-synonymous	T533A	0.152	0.3	0.115
Modern terminal	R0	A	13933	G	ND5	Non-synonymous	T533A	0.152	0.3	0.115
Pre-terminal	R0	A	13933	G	ND5	Non-synonymous	T533A	0.152	0.3	0.115
Pre-terminal	R0	A	13933	G	ND5	Non-synonymous	T533A	0.152	0.3	0.115
Pre-terminal	R0	A	13933	G	ND5	Non-synonymous	T533A	0.152	0.3	0.115
Pre-terminal	R0	A	13933	G	ND5	Non-synonymous	T533A	0.152	0.3	0.115
Modern terminal	U	A	13933	G	ND5	Non-synonymous	T533A	0.152	0.3	0.115
Pre-terminal	U	A	13933	G	ND5	Non-synonymous	T533A	0.152	0.3	0.115
Pre-terminal	U	A	13933	G	ND5	Non-synonymous	T533A	0.152	0.3	0.115
Pre-terminal	U	A	13933	G	ND5	Non-synonymous	T533A	0.152	0.3	0.115
Modern terminal	R0	C	13934	T	ND5	Non-synonymous	T533M	0.026	0.8	0.113
Modern terminal	R0	C	13934	T	ND5	Non-synonymous	T533M	0.026	0.8	0.113
Modern terminal	R0	C	13934	T	ND5	Non-synonymous	T533M	0.026	0.8	0.113
Pre-terminal	R0	C	13934	T	ND5	Non-synonymous	T533M	0.026	0.8	0.113
Pre-terminal	R0	C	13934	T	ND5	Non-synonymous	T533M	0.026	0.8	0.113
Pre-terminal	R0	C	13934	T	ND5	Non-synonymous	T533M	0.026	0.8	0.113
Pre-terminal	R0	C	13934	T	ND5	Non-synonymous	T533M	0.026	0.8	0.113
Modern terminal	U	C	13934	T	ND5	Non-synonymous	T533M	0.026	0.8	0.113
Modern terminal	U	C	13934	T	ND5	Non-synonymous	T533M	0.026	0.8	0.113
Modern terminal	U	C	13934	T	ND5	Non-synonymous	T533M	0.026	0.8	0.113
Modern terminal	U	C	13934	T	ND5	Non-synonymous	T533M	0.026	0.8	0.113
Pre-terminal	U	C	13934	T	ND5	Non-synonymous	T533M	0.026	0.8	0.113
Pre-terminal	U	C	13934	T	ND5	Non-synonymous	T533M	0.026	0.8	0.113
Pre-terminal	U	C	13934	T	ND5	Non-synonymous	T533M	0.026	0.8	0.113
Pre-terminal	U	C	13934	T	ND5	Non-synonymous	T533M	0.026	0.8	0.113
Pre-terminal	U	C	13934	T	ND5	Non-synonymous	T533M	0.026	0.8	0.113
Modern terminal	JT	C	13934	T	ND5	Non-synonymous	T533M	0.026	0.8	0.113

Modern terminal	JT	C	13934	T	ND5	Non-synonymous	T533M	0.026	0.8	0.113
Modern terminal	JT	C	13934	T	ND5	Non-synonymous	T533M	0.026	0.8	0.113
Modern terminal	JT	C	13934	T	ND5	Non-synonymous	T533M	0.026	0.8	0.113
Modern terminal	JT	C	13934	T	ND5	Non-synonymous	T533M	0.026	0.8	0.113
Pre-terminal	JT	C	13934	T	ND5	Non-synonymous	T533M	0.026	0.8	0.113
Pre-terminal	JT	C	13934	T	ND5	Non-synonymous	T533M	0.026	0.8	0.113
Modern terminal	N1	C	13934	T	ND5	Non-synonymous	T533M	0.026	0.8	0.113
Modern terminal	N1	C	13934	T	ND5	Non-synonymous	T533M	0.026	0.8	0.113
Modern terminal	N2	C	13934	T	ND5	Non-synonymous	T533M	0.026	0.8	0.113
Pre-terminal	N2	C	13934	T	ND5	Non-synonymous	T533M	0.026	0.8	0.113
Modern terminal	R0	A	13942	G	ND5	Non-synonymous	T536A	0.357	0.12	0.221
Modern terminal	R0	A	13942	G	ND5	Non-synonymous	T536A	0.357	0.12	0.221
Pre-terminal	R0	A	13942	G	ND5	Non-synonymous	T536A	0.357	0.12	0.221
Pre-terminal	R0	A	13942	G	ND5	Non-synonymous	T536A	0.357	0.12	0.221
Modern terminal	U	A	13942	G	ND5	Non-synonymous	T536A	0.357	0.12	0.221
Modern terminal	U	A	13942	G	ND5	Non-synonymous	T536A	0.357	0.12	0.221
Modern terminal	JT	A	13942	G	ND5	Non-synonymous	T536A	0.357	0.12	0.221
Modern terminal	JT	A	13942	G	ND5	Non-synonymous	T536A	0.357	0.12	0.221
Modern terminal	JT	A	13942	G	ND5	Non-synonymous	T536A	0.357	0.12	0.221
Modern terminal	R0	C	13943	T	ND5	Non-synonymous	T536M	0.194	0.19	0.088
Pre-terminal	R0	C	13943	T	ND5	Non-synonymous	T536M	0.194	0.19	0.088
Modern terminal	U	C	13943	T	ND5	Non-synonymous	T536M	0.194	0.19	0.088
Modern terminal	U	C	13943	T	ND5	Non-synonymous	T536M	0.194	0.19	0.088
Pre-terminal	U	C	13943	T	ND5	Non-synonymous	T536M	0.194	0.19	0.088
Pre-terminal	JT	C	13943	T	ND5	Non-synonymous	T536M	0.194	0.19	0.088
Modern terminal	N1	C	13943	T	ND5	Non-synonymous	T536M	0.194	0.19	0.088
Modern terminal	JT	A	13945	G	ND5	Non-synonymous	I537V	0.014	0.55	0.088
Ancient terminal	N2	A	13945	C	ND5	Non-synonymous	I537L	0.112	0.96	0.289
Pre-terminal	R0	C	13948	T	ND5	Non-synonymous	P538S	1	0.26	0.316
Modern terminal	R0	A	13952	G	ND5	Non-synonymous	Y539C	1	0.17	0.346
Modern terminal	R0	G	13958	C	ND5	Non-synonymous	G541A	0.498	0.73	0.164
Modern terminal	U	G	13958	C	ND5	Non-synonymous	G541A	0.498	0.73	0.164
Pre-terminal	U	G	13958	C	ND5	Non-synonymous	G541A	0.498	0.73	0.164
Pre-terminal	U	G	13958	C	ND5	Non-synonymous	G541A	0.498	0.73	0.164
Ancient terminal	JT	G	13958	C	ND5	Non-synonymous	G541A	0.498	0.73	0.164
Modern terminal	JT	G	13958	C	ND5	Non-synonymous	G541A	0.498	0.73	0.164
Modern terminal	R0	A	13966	G	ND5	Non-synonymous	T544A	0.001	0.39	0.249
Modern terminal	R0	A	13966	G	ND5	Non-synonymous	T544A	0.001	0.39	0.249
Modern terminal	R0	A	13966	G	ND5	Non-synonymous	T544A	0.001	0.39	0.249
Modern terminal	R0	A	13966	G	ND5	Non-synonymous	T544A	0.001	0.39	0.249
Modern terminal	R0	A	13966	G	ND5	Non-synonymous	T544A	0.001	0.39	0.249
Ancient terminal	U	A	13966	G	ND5	Non-synonymous	T544A	0.001	0.39	0.249
Modern terminal	U	A	13966	G	ND5	Non-synonymous	T544A	0.001	0.39	0.249
Modern terminal	U	A	13966	G	ND5	Non-synonymous	T544A	0.001	0.39	0.249
Modern terminal	U	A	13966	G	ND5	Non-synonymous	T544A	0.001	0.39	0.249
Pre-terminal	U	A	13966	G	ND5	Non-synonymous	T544A	0.001	0.39	0.249
Pre-terminal	U	A	13966	G	ND5	Non-synonymous	T544A	0.001	0.39	0.249
Modern terminal	JT	A	13966	G	ND5	Non-synonymous	T544A	0.001	0.39	0.249
Modern terminal	JT	A	13966	G	ND5	Non-synonymous	T544A	0.001	0.39	0.249
Modern terminal	R0	C	13967	T	ND5	Non-synonymous	T544M	0.015	1	0.086

Pre-terminal	R0	C	13967	T	ND5	Non-synonymous	T544M	0.015	1	0.086
Pre-terminal	R0	C	13967	T	ND5	Non-synonymous	T544M	0.015	1	0.086
Pre-terminal	R0	C	13967	T	ND5	Non-synonymous	T544M	0.015	1	0.086
Modern terminal	U	C	13967	T	ND5	Non-synonymous	T544M	0.015	1	0.086
Modern terminal	U	C	13967	T	ND5	Non-synonymous	T544M	0.015	1	0.086
Pre-terminal	U	C	13967	T	ND5	Non-synonymous	T544M	0.015	1	0.086
Modern terminal	JT	C	13967	T	ND5	Non-synonymous	T544M	0.015	1	0.086
Modern terminal	N1	C	13967	T	ND5	Non-synonymous	T544M	0.015	1	0.086
Modern terminal	JT	A	13969	T	ND5	Non-synonymous	S545C	1	0.04	0.275
Pre-terminal	N1	A	13974	T	ND5	Non-synonymous	Q546H	0.999	0.03	0.256
Ancient terminal	JT	C	13981	T	ND5	Non-synonymous	P549S	0.066	0.45	0.105
Modern terminal	U	C	13982	T	ND5	Non-synonymous	P549L	0.627	0.01	0.156
Modern terminal	U	T	13988	G	ND5	Non-synonymous	L551R	0.999	0.63	0.692
Modern terminal	U	T	13988	G	ND5	Non-synonymous	L551R	0.999	0.63	0.692
Pre-terminal	U	T	13988	G	ND5	Non-synonymous	L551R	0.999	0.63	0.692
Modern terminal	U	C	13990	T	ND5	Non-synonymous	L552F	0.999	0.03	0.29
Modern terminal	U	T	14000	C	ND5	Non-synonymous	L555P	1	0.21	0.268
Modern terminal	JT	T	14000	A	ND5	Non-synonymous	L555Q	1	0.38	0.177
Pre-terminal	JT	T	14000	A	ND5	Non-synonymous	L555Q	1	0.38	0.177
Modern terminal	R0	A	14002	G	ND5	Non-synonymous	T556A	0.001	0.66	0.059
Modern terminal	R0	A	14002	G	ND5	Non-synonymous	T556A	0.001	0.66	0.059
Modern terminal	R0	A	14002	G	ND5	Non-synonymous	T556A	0.001	0.66	0.059
Modern terminal	R0	A	14002	G	ND5	Non-synonymous	T556A	0.001	0.66	0.059
Modern terminal	R0	A	14002	G	ND5	Non-synonymous	T556A	0.001	0.66	0.059
Pre-terminal	R0	A	14002	G	ND5	Non-synonymous	T556A	0.001	0.66	0.059
Pre-terminal	R0	A	14002	G	ND5	Non-synonymous	T556A	0.001	0.66	0.059
Modern terminal	U	A	14002	G	ND5	Non-synonymous	T556A	0.001	0.66	0.059
Modern terminal	U	A	14002	G	ND5	Non-synonymous	T556A	0.001	0.66	0.059
Modern terminal	U	A	14002	G	ND5	Non-synonymous	T556A	0.001	0.66	0.059
Pre-terminal	U	A	14002	G	ND5	Non-synonymous	T556A	0.001	0.66	0.059
Pre-terminal	U	A	14002	G	ND5	Non-synonymous	T556A	0.001	0.66	0.059
Ancient terminal	JT	A	14002	G	ND5	Non-synonymous	T556A	0.001	0.66	0.059
Modern terminal	JT	A	14002	G	ND5	Non-synonymous	T556A	0.001	0.66	0.059
Modern terminal	JT	A	14002	G	ND5	Non-synonymous	T556A	0.001	0.66	0.059
Modern terminal	U	C	14003	T	ND5	Non-synonymous	T556I	0.007	0.5	0.082
Pre-terminal	U	C	14003	T	ND5	Non-synonymous	T556I	0.007	0.5	0.082
Modern terminal	JT	A	14015	T	ND5	Non-synonymous	K560M	1	0.04	0.207
Modern terminal	R0	A	14029	G	ND5	Non-synonymous	T565A	0.006	0.39	0.128
Modern terminal	JT	A	14029	G	ND5	Non-synonymous	T565A	0.006	0.39	0.128
Pre-terminal	JT	A	14029	G	ND5	Non-synonymous	T565A	0.006	0.39	0.128
Modern terminal	R0	C	14041	T	ND5	Non-synonymous	H569Y	0.002	1	0.148
Pre-terminal	R0	A	14042	T	ND5	Non-synonymous	H569L	0.312	0.65	0.16
Modern terminal	U	A	14047	G	ND5	Non-synonymous	I571V	0.002	1	0.061
Modern terminal	JT	A	14047	G	ND5	Non-synonymous	I571V	0.002	1	0.061
Modern terminal	R0	T	14048	C	ND5	Non-synonymous	I571T	0.052	0.46	0.08
Pre-terminal	R0	T	14050	A	ND5	Non-synonymous	S572T	0.855	0.64	0.05
Ancient terminal	U	C	14051	T	ND5	Non-synonymous	S572F	0.999	0.12	0.159
Modern terminal	R0	A	14053	G	ND5	Non-synonymous	T573A	0	1	0.041
Modern terminal	R0	A	14053	G	ND5	Non-synonymous	T573A	0	1	0.041
Modern terminal	R0	A	14053	G	ND5	Non-synonymous	T573A	0	1	0.041

Modern terminal	R0	A	14053	G	ND5	Non-synonymous	T573A	0	1	0.041
Modern terminal	R0	A	14053	G	ND5	Non-synonymous	T573A	0	1	0.041
Modern terminal	R0	A	14053	G	ND5	Non-synonymous	T573A	0	1	0.041
Modern terminal	R0	A	14053	G	ND5	Non-synonymous	T573A	0	1	0.041
Modern terminal	R0	A	14053	G	ND5	Non-synonymous	T573A	0	1	0.041
Pre-terminal	R0	A	14053	G	ND5	Non-synonymous	T573A	0	1	0.041
Pre-terminal	R0	A	14053	G	ND5	Non-synonymous	T573A	0	1	0.041
Ancient terminal	U	A	14053	G	ND5	Non-synonymous	T573A	0	1	0.041
Ancient terminal	U	A	14053	G	ND5	Non-synonymous	T573A	0	1	0.041
Ancient terminal	U	A	14053	G	ND5	Non-synonymous	T573A	0	1	0.041
Modern terminal	U	A	14053	G	ND5	Non-synonymous	T573A	0	1	0.041
Modern terminal	U	A	14053	G	ND5	Non-synonymous	T573A	0	1	0.041
Modern terminal	U	A	14053	G	ND5	Non-synonymous	T573A	0	1	0.041
Modern terminal	U	A	14053	G	ND5	Non-synonymous	T573A	0	1	0.041
Pre-terminal	U	A	14053	G	ND5	Non-synonymous	T573A	0	1	0.041
Pre-terminal	U	A	14053	G	ND5	Non-synonymous	T573A	0	1	0.041
Pre-terminal	U	A	14053	G	ND5	Non-synonymous	T573A	0	1	0.041
Pre-terminal	U	A	14053	G	ND5	Non-synonymous	T573A	0	1	0.041
Modern terminal	JT	A	14053	G	ND5	Non-synonymous	T573A	0	1	0.041
Modern terminal	JT	A	14053	G	ND5	Non-synonymous	T573A	0	1	0.041
Modern terminal	JT	A	14053	G	ND5	Non-synonymous	T573A	0	1	0.041
Modern terminal	JT	A	14053	G	ND5	Non-synonymous	T573A	0	1	0.041
Modern terminal	JT	A	14053	G	ND5	Non-synonymous	T573A	0	1	0.041
Pre-terminal	JT	A	14053	G	ND5	Non-synonymous	T573A	0	1	0.041
Pre-terminal	JT	A	14053	G	ND5	Non-synonymous	T573A	0	1	0.041
Modern terminal	X	A	14053	G	ND5	Non-synonymous	T573A	0	1	0.041
Pre-terminal	R0	T	14060	C	ND5	Non-synonymous	I575T	0.088	0.8	0.108
Modern terminal	R0	C	14061	A	ND5	Non-synonymous	I575M	0.995	0.26	0.094
Pre-terminal	R0	T	14063	C	ND5	Non-synonymous	I576T	0.008	0.63	0.062
Pre-terminal	U	T	14063	C	ND5	Non-synonymous	I576T	0.008	0.63	0.062
Pre-terminal	U	T	14063	C	ND5	Non-synonymous	I576T	0.008	0.63	0.062
Modern terminal	JT	A	14065	G	ND5	Non-synonymous	T577A	0.083	0.42	0.133
Pre-terminal	R0	C	14066	T	ND5	Non-synonymous	T577I	0	0.58	0.116
Ancient terminal	R0	A	14071	G	ND5	Non-synonymous	T579A	0.497	0.86	0.185
Pre-terminal	R0	A	14071	G	ND5	Non-synonymous	T579A	0.497	0.86	0.185
Modern terminal	U	A	14071	G	ND5	Non-synonymous	T579A	0.497	0.86	0.185
Modern terminal	U	A	14071	G	ND5	Non-synonymous	T579A	0.497	0.86	0.185
Modern terminal	U	A	14071	G	ND5	Non-synonymous	T579A	0.497	0.86	0.185
Modern terminal	U	A	14071	G	ND5	Non-synonymous	T579A	0.497	0.86	0.185
Pre-terminal	U	A	14071	G	ND5	Non-synonymous	T579A	0.497	0.86	0.185
Modern terminal	R0	T	14093	C	ND5	Non-synonymous	L586P	1	0.12	0.697
Pre-terminal	U	T	14095	A	ND5	Non-synonymous	Y587N	1	0	0.599
Ancient terminal	R0	T	14110	C	ND5	Non-synonymous	F592L	0.655	1	0.042
Modern terminal	R0	T	14110	C	ND5	Non-synonymous	F592L	0.655	1	0.042
Modern terminal	R0	T	14110	C	ND5	Non-synonymous	F592L	0.655	1	0.042
Modern terminal	R0	T	14110	C	ND5	Non-synonymous	F592L	0.655	1	0.042
Pre-terminal	R0	T	14110	C	ND5	Non-synonymous	F592L	0.655	1	0.042
Pre-terminal	R0	T	14110	C	ND5	Non-synonymous	F592L	0.655	1	0.042
Pre-terminal	R0	T	14110	C	ND5	Non-synonymous	F592L	0.655	1	0.042
Pre-terminal	R0	T	14110	C	ND5	Non-synonymous	F592L	0.655	1	0.042

Pre-terminal	R0	T	14110	C	ND5	Non-synonymous	F592L	0.655	1	0.042
Modern terminal	U	T	14110	C	ND5	Non-synonymous	F592L	0.655	1	0.042
Modern terminal	U	T	14110	C	ND5	Non-synonymous	F592L	0.655	1	0.042
Modern terminal	U	T	14110	C	ND5	Non-synonymous	F592L	0.655	1	0.042
Modern terminal	U	T	14110	C	ND5	Non-synonymous	F592L	0.655	1	0.042
Modern terminal	U	T	14110	C	ND5	Non-synonymous	F592L	0.655	1	0.042
Pre-terminal	U	T	14110	C	ND5	Non-synonymous	F592L	0.655	1	0.042
Pre-terminal	U	T	14110	C	ND5	Non-synonymous	F592L	0.655	1	0.042
Pre-terminal	U	T	14110	C	ND5	Non-synonymous	F592L	0.655	1	0.042
Pre-terminal	U	T	14110	C	ND5	Non-synonymous	F592L	0.655	1	0.042
Pre-terminal	U	T	14110	C	ND5	Non-synonymous	F592L	0.655	1	0.042
Pre-terminal	U	T	14110	C	ND5	Non-synonymous	F592L	0.655	1	0.042
Pre-terminal	U	T	14110	C	ND5	Non-synonymous	F592L	0.655	1	0.042
Ancient terminal	JT	T	14110	C	ND5	Non-synonymous	F592L	0.655	1	0.042
Modern terminal	JT	T	14110	C	ND5	Non-synonymous	F592L	0.655	1	0.042
Modern terminal	JT	T	14110	C	ND5	Non-synonymous	F592L	0.655	1	0.042
Modern terminal	JT	T	14110	C	ND5	Non-synonymous	F592L	0.655	1	0.042
Pre-terminal	JT	T	14110	C	ND5	Non-synonymous	F592L	0.655	1	0.042
Ancient terminal	N1	T	14110	C	ND5	Non-synonymous	F592L	0.655	1	0.042
Ancient terminal	N2	T	14110	C	ND5	Non-synonymous	F592L	0.655	1	0.042
Pre-terminal	U	C	14112	A	ND5	Non-synonymous	F592L	0.655	1	0.042
Modern terminal	R0	C	14116	T	ND5	Non-synonymous	P594S	0.425	0.56	0.199
Modern terminal	U	A	14122	G	ND5	Non-synonymous	I596V	0.15	1	0.044
Modern terminal	U	A	14122	G	ND5	Non-synonymous	I596V	0.15	1	0.044
Ancient terminal	X	A	14122	G	ND5	Non-synonymous	I596V	0.15	1	0.044
Modern terminal	R0	A	14128	G	ND5	Non-synonymous	T598A	0.15	0.85	0.066
Modern terminal	R0	A	14128	G	ND5	Non-synonymous	T598A	0.15	0.85	0.066
Pre-terminal	R0	A	14128	G	ND5	Non-synonymous	T598A	0.15	0.85	0.066
Modern terminal	U	A	14128	G	ND5	Non-synonymous	T598A	0.15	0.85	0.066
Modern terminal	JT	A	14128	G	ND5	Non-synonymous	T598A	0.15	0.85	0.066
Modern terminal	JT	A	14128	G	ND5	Non-synonymous	T598A	0.15	0.85	0.066
Modern terminal	JT	A	14128	G	ND5	Non-synonymous	T598A	0.15	0.85	0.066
Pre-terminal	JT	A	14128	G	ND5	Non-synonymous	T598A	0.15	0.85	0.066
Pre-terminal	JT	A	14128	G	ND5	Non-synonymous	T598A	0.15	0.85	0.066
Pre-terminal	JT	A	14128	G	ND5	Non-synonymous	T598A	0.15	0.85	0.066
Modern terminal	R0	C	14129	T	ND5	Non-synonymous	T598I	0.151	0.3	0.062
Modern terminal	R0	C	14129	T	ND5	Non-synonymous	T598I	0.151	0.3	0.062
Modern terminal	R0	C	14129	T	ND5	Non-synonymous	T598I	0.151	0.3	0.062
Pre-terminal	R0	C	14129	T	ND5	Non-synonymous	T598I	0.151	0.3	0.062
Pre-terminal	U	C	14129	T	ND5	Non-synonymous	T598I	0.151	0.3	0.062
Pre-terminal	U	C	14129	T	ND5	Non-synonymous	T598I	0.151	0.3	0.062
Modern terminal	X	C	14129	T	ND5	Non-synonymous	T598I	0.151	0.3	0.062
Modern terminal	X	C	14129	T	ND5	Non-synonymous	T598I	0.151	0.3	0.062
Modern terminal	N2	C	14129	T	ND5	Non-synonymous	T598I	0.151	0.3	0.062
Modern terminal	R0	A	14140	G	ND5	Non-synonymous	I602V	0.15	0.13	0.047
Modern terminal	R0	C	14142	A	ND5	Non-synonymous	I602M	0.698	0.24	0.048
Pre-terminal	U	C	14142	A	ND5	Non-synonymous	I602M	0.698	0.24	0.048
Pre-terminal	U	C	14142	G	ND5	Non-synonymous	I602M	0.698	0.24	0.048
Ancient terminal	R0	C	14144	T	ND5	Non-synonymous	T603M	*	0.01	0.104
Modern terminal	R0	T	14153	C	ND6	Non-synonymous	N174S	0.999	0.28	0.808

Pre-terminal	R0	T	14153	C	ND6	Non-synonymous	N174S	0.999	0.28	0.808
Modern terminal	U	T	14153	C	ND6	Non-synonymous	N174S	0.999	0.28	0.808
Modern terminal	U	T	14153	C	ND6	Non-synonymous	N174S	0.999	0.28	0.808
Modern terminal	U	T	14153	C	ND6	Non-synonymous	N174S	0.999	0.28	0.808
Modern terminal	N2	T	14153	C	ND6	Non-synonymous	N174S	0.999	0.28	0.808
Ancient terminal	U	C	14157	A	ND6	Non-synonymous	G173W	1	0	0.933
Modern terminal	U	G	14160	A	ND6	Non-synonymous	R172W	1	0	0.894
Modern terminal	R0	G	14162	A	ND6	Non-synonymous	A171V	0.001	0.19	0.59
Modern terminal	R0	G	14162	A	ND6	Non-synonymous	A171V	0.001	0.19	0.59
Modern terminal	R0	G	14162	A	ND6	Non-synonymous	A171V	0.001	0.19	0.59
Modern terminal	R0	G	14162	A	ND6	Non-synonymous	A171V	0.001	0.19	0.59
Modern terminal	R0	G	14162	A	ND6	Non-synonymous	A171V	0.001	0.19	0.59
Pre-terminal	R0	G	14162	A	ND6	Non-synonymous	A171V	0.001	0.19	0.59
Pre-terminal	R0	G	14162	A	ND6	Non-synonymous	A171V	0.001	0.19	0.59
Pre-terminal	R0	G	14162	A	ND6	Non-synonymous	A171V	0.001	0.19	0.59
Modern terminal	U	G	14162	A	ND6	Non-synonymous	A171V	0.001	0.19	0.59
Modern terminal	JT	G	14162	A	ND6	Non-synonymous	A171V	0.001	0.19	0.59
Modern terminal	JT	G	14162	A	ND6	Non-synonymous	A171V	0.001	0.19	0.59
Modern terminal	JT	G	14162	A	ND6	Non-synonymous	A171V	0.001	0.19	0.59
Modern terminal	JT	G	14162	A	ND6	Non-synonymous	A171V	0.001	0.19	0.59
Modern terminal	R0	C	14163	T	ND6	Non-synonymous	A171T	0	1	0.177
Modern terminal	R0	C	14163	T	ND6	Non-synonymous	A171T	0	1	0.177
Modern terminal	U	T	14166	C	ND6	Non-synonymous	I170V	0.578	1	0.399
Modern terminal	U	T	14166	C	ND6	Non-synonymous	I170V	0.578	1	0.399
Modern terminal	JT	A	14170	T	ND6	Non-synonymous	I168M	1	0.82	0.625
Ancient terminal	R0	T	14178	C	ND6	Non-synonymous	I166V	0.007	1	0.387
Modern terminal	R0	T	14178	C	ND6	Non-synonymous	I166V	0.007	1	0.387
Modern terminal	R0	T	14178	C	ND6	Non-synonymous	I166V	0.007	1	0.387
Modern terminal	R0	T	14178	C	ND6	Non-synonymous	I166V	0.007	1	0.387
Modern terminal	R0	T	14178	C	ND6	Non-synonymous	I166V	0.007	1	0.387
Modern terminal	R0	T	14178	C	ND6	Non-synonymous	I166V	0.007	1	0.387
Pre-terminal	R0	T	14178	C	ND6	Non-synonymous	I166V	0.007	1	0.387
Modern terminal	U	T	14178	C	ND6	Non-synonymous	I166V	0.007	1	0.387
Modern terminal	U	T	14178	C	ND6	Non-synonymous	I166V	0.007	1	0.387
Modern terminal	U	T	14178	C	ND6	Non-synonymous	I166V	0.007	1	0.387
Modern terminal	U	T	14178	C	ND6	Non-synonymous	I166V	0.007	1	0.387
Modern terminal	U	T	14178	C	ND6	Non-synonymous	I166V	0.007	1	0.387
Modern terminal	U	T	14178	C	ND6	Non-synonymous	I166V	0.007	1	0.387
Modern terminal	U	T	14178	C	ND6	Non-synonymous	I166V	0.007	1	0.387
Modern terminal	U	T	14178	C	ND6	Non-synonymous	I166V	0.007	1	0.387
Pre-terminal	U	T	14178	C	ND6	Non-synonymous	I166V	0.007	1	0.387
Modern terminal	JT	T	14178	C	ND6	Non-synonymous	I166V	0.007	1	0.387
Pre-terminal	JT	T	14178	C	ND6	Non-synonymous	I166V	0.007	1	0.387
Pre-terminal	JT	T	14178	C	ND6	Non-synonymous	I166V	0.007	1	0.387
Modern terminal	N1	T	14178	C	ND6	Non-synonymous	I166V	0.007	1	0.387
Modern terminal	N1	T	14178	C	ND6	Non-synonymous	I166V	0.007	1	0.387
Pre-terminal	N1	T	14178	C	ND6	Non-synonymous	I166V	0.007	1	0.387
Modern terminal	X	T	14178	C	ND6	Non-synonymous	I166V	0.007	1	0.387
Pre-terminal	X	T	14178	C	ND6	Non-synonymous	I166V	0.007	1	0.387
Ancient terminal	R0	T	14180	C	ND6	Non-synonymous	Y165C	1	0.02	0.87

Modern terminal	R0	T	14180	C	ND6	Non-synonymous	Y165C	1	0.02	0.87
Modern terminal	R0	T	14180	C	ND6	Non-synonymous	Y165C	1	0.02	0.87
Modern terminal	R0	T	14180	C	ND6	Non-synonymous	Y165C	1	0.02	0.87
Modern terminal	R0	T	14180	C	ND6	Non-synonymous	Y165C	1	0.02	0.87
Modern terminal	R0	T	14180	C	ND6	Non-synonymous	Y165C	1	0.02	0.87
Modern terminal	R0	T	14180	C	ND6	Non-synonymous	Y165C	1	0.02	0.87
Modern terminal	R0	T	14180	C	ND6	Non-synonymous	Y165C	1	0.02	0.87
Modern terminal	R0	T	14180	C	ND6	Non-synonymous	Y165C	1	0.02	0.87
Modern terminal	R0	T	14180	C	ND6	Non-synonymous	Y165C	1	0.02	0.87
Modern terminal	R0	T	14180	C	ND6	Non-synonymous	Y165C	1	0.02	0.87
Modern terminal	R0	T	14180	C	ND6	Non-synonymous	Y165C	1	0.02	0.87
Modern terminal	R0	T	14180	C	ND6	Non-synonymous	Y165C	1	0.02	0.87
Modern terminal	R0	T	14180	C	ND6	Non-synonymous	Y165C	1	0.02	0.87
Modern terminal	R0	T	14180	C	ND6	Non-synonymous	Y165C	1	0.02	0.87
Modern terminal	R0	T	14180	C	ND6	Non-synonymous	Y165C	1	0.02	0.87
Modern terminal	R0	T	14180	C	ND6	Non-synonymous	Y165C	1	0.02	0.87
Modern terminal	R0	T	14180	C	ND6	Non-synonymous	Y165C	1	0.02	0.87
Modern terminal	R0	T	14180	C	ND6	Non-synonymous	Y165C	1	0.02	0.87
Modern terminal	R0	T	14180	C	ND6	Non-synonymous	Y165C	1	0.02	0.87
Modern terminal	R0	T	14180	C	ND6	Non-synonymous	Y165C	1	0.02	0.87
Modern terminal	R0	T	14180	C	ND6	Non-synonymous	Y165C	1	0.02	0.87
Modern terminal	R0	T	14180	C	ND6	Non-synonymous	Y165C	1	0.02	0.87
Modern terminal	R0	T	14180	C	ND6	Non-synonymous	Y165C	1	0.02	0.87
Modern terminal	R0	T	14180	C	ND6	Non-synonymous	Y165C	1	0.02	0.87
Pre-terminal	R0	T	14180	C	ND6	Non-synonymous	Y165C	1	0.02	0.87
Pre-terminal	R0	T	14180	C	ND6	Non-synonymous	Y165C	1	0.02	0.87
Pre-terminal	R0	T	14180	C	ND6	Non-synonymous	Y165C	1	0.02	0.87
Pre-terminal	R0	T	14180	C	ND6	Non-synonymous	Y165C	1	0.02	0.87
Pre-terminal	R0	T	14180	C	ND6	Non-synonymous	Y165C	1	0.02	0.87
Pre-terminal	R0	T	14180	G	ND6	Non-synonymous	Y165C	1	0.02	0.87
Pre-terminal	R0	T	14180	C	ND6	Non-synonymous	Y165C	1	0.02	0.87
Pre-terminal	R0	T	14180	C	ND6	Non-synonymous	Y165C	1	0.02	0.87
Pre-terminal	R0	T	14180	C	ND6	Non-synonymous	Y165C	1	0.02	0.87
Pre-terminal	R0	T	14180	C	ND6	Non-synonymous	Y165C	1	0.02	0.87
Pre-terminal	R0	T	14180	C	ND6	Non-synonymous	Y165C	1	0.02	0.87
Pre-terminal	R0	T	14180	C	ND6	Non-synonymous	Y165C	1	0.02	0.87
Pre-terminal	R0	T	14180	C	ND6	Non-synonymous	Y165C	1	0.02	0.87
Ancient terminal	U	T	14180	C	ND6	Non-synonymous	Y165C	1	0.02	0.87
Ancient terminal	U	T	14180	C	ND6	Non-synonymous	Y165C	1	0.02	0.87
Modern terminal	U	T	14180	C	ND6	Non-synonymous	Y165C	1	0.02	0.87
Modern terminal	U	T	14180	C	ND6	Non-synonymous	Y165C	1	0.02	0.87
Modern terminal	U	T	14180	C	ND6	Non-synonymous	Y165C	1	0.02	0.87
Modern terminal	U	T	14180	C	ND6	Non-synonymous	Y165C	1	0.02	0.87
Modern terminal	U	T	14180	C	ND6	Non-synonymous	Y165C	1	0.02	0.87
Modern terminal	U	T	14180	C	ND6	Non-synonymous	Y165C	1	0.02	0.87
Modern terminal	U	T	14180	C	ND6	Non-synonymous	Y165C	1	0.02	0.87
Modern terminal	U	T	14180	C	ND6	Non-synonymous	Y165C	1	0.02	0.87
Modern terminal	U	T	14180	C	ND6	Non-synonymous	Y165C	1	0.02	0.87
Modern terminal	U	T	14180	C	ND6	Non-synonymous	Y165C	1	0.02	0.87
Modern terminal	U	T	14180	C	ND6	Non-synonymous	Y165C	1	0.02	0.87

Modern terminal	U	T	14180	C	ND6	Non-synonymous	Y165C	1	0.02	0.87
Modern terminal	U	T	14180	C	ND6	Non-synonymous	Y165C	1	0.02	0.87
Modern terminal	U	T	14180	C	ND6	Non-synonymous	Y165C	1	0.02	0.87
Modern terminal	U	T	14180	C	ND6	Non-synonymous	Y165C	1	0.02	0.87
Modern terminal	U	T	14180	C	ND6	Non-synonymous	Y165C	1	0.02	0.87
Modern terminal	U	T	14180	C	ND6	Non-synonymous	Y165C	1	0.02	0.87
Modern terminal	U	T	14180	G	ND6	Non-synonymous	Y165S	0.999	0.02	0.865
Modern terminal	U	T	14180	G	ND6	Non-synonymous	Y165S	0.999	0.02	0.865
Pre-terminal	U	T	14180	C	ND6	Non-synonymous	Y165C	1	0.02	0.87
Pre-terminal	U	T	14180	C	ND6	Non-synonymous	Y165C	1	0.02	0.87
Pre-terminal	U	T	14180	C	ND6	Non-synonymous	Y165C	1	0.02	0.87
Pre-terminal	U	T	14180	C	ND6	Non-synonymous	Y165C	1	0.02	0.87
Ancient terminal	JT	T	14180	C	ND6	Non-synonymous	Y165C	1	0.02	0.87
Modern terminal	JT	T	14180	C	ND6	Non-synonymous	Y165C	1	0.02	0.87
Modern terminal	JT	T	14180	C	ND6	Non-synonymous	Y165C	1	0.02	0.87
Modern terminal	JT	T	14180	C	ND6	Non-synonymous	Y165C	1	0.02	0.87
Modern terminal	JT	T	14180	C	ND6	Non-synonymous	Y165C	1	0.02	0.87
Modern terminal	JT	T	14180	C	ND6	Non-synonymous	Y165C	1	0.02	0.87
Modern terminal	JT	T	14180	C	ND6	Non-synonymous	Y165C	1	0.02	0.87
Modern terminal	JT	T	14180	C	ND6	Non-synonymous	Y165C	1	0.02	0.87
Modern terminal	JT	T	14180	C	ND6	Non-synonymous	Y165C	1	0.02	0.87
Modern terminal	JT	T	14180	C	ND6	Non-synonymous	Y165C	1	0.02	0.87
Modern terminal	JT	T	14180	C	ND6	Non-synonymous	Y165C	1	0.02	0.87
Modern terminal	JT	T	14180	C	ND6	Non-synonymous	Y165C	1	0.02	0.87
Modern terminal	JT	T	14180	C	ND6	Non-synonymous	Y165C	1	0.02	0.87
Modern terminal	JT	T	14180	C	ND6	Non-synonymous	Y165C	1	0.02	0.87
Modern terminal	JT	T	14180	C	ND6	Non-synonymous	Y165C	1	0.02	0.87
Modern terminal	JT	T	14180	C	ND6	Non-synonymous	Y165C	1	0.02	0.87
Modern terminal	JT	T	14180	C	ND6	Non-synonymous	Y165C	1	0.02	0.87
Pre-terminal	JT	T	14180	C	ND6	Non-synonymous	Y165C	1	0.02	0.87
Pre-terminal	JT	T	14180	C	ND6	Non-synonymous	Y165C	1	0.02	0.87
Pre-terminal	JT	T	14180	C	ND6	Non-synonymous	Y165C	1	0.02	0.87
Pre-terminal	JT	T	14180	C	ND6	Non-synonymous	Y165C	1	0.02	0.87
Pre-terminal	JT	T	14180	C	ND6	Non-synonymous	Y165C	1	0.02	0.87
Modern terminal	N1	T	14180	C	ND6	Non-synonymous	Y165C	1	0.02	0.87
Modern terminal	N1	T	14180	C	ND6	Non-synonymous	Y165C	1	0.02	0.87
Modern terminal	N1	T	14180	C	ND6	Non-synonymous	Y165C	1	0.02	0.87
Pre-terminal	N1	T	14180	C	ND6	Non-synonymous	Y165C	1	0.02	0.87
Modern terminal	X	T	14180	C	ND6	Non-synonymous	Y165C	1	0.02	0.87
Ancient terminal	N2	T	14180	C	ND6	Non-synonymous	Y165C	1	0.02	0.87
Pre-terminal	R0	A	14181	G	ND6	Non-synonymous	Y165H	0.211	0.02	0.738
Modern terminal	U	A	14181	G	ND6	Non-synonymous	Y165H	0.211	0.02	0.738
Ancient terminal	R0	C	14186	T	ND6	Non-synonymous	G163D	1	0.02	0.87
Modern terminal	R0	C	14187	T	ND6	Non-synonymous	G163S	1	0.59	0.65
Modern terminal	R0	C	14187	T	ND6	Non-synonymous	G163S	1	0.59	0.65
Modern terminal	R0	C	14187	T	ND6	Non-synonymous	G163S	1	0.59	0.65
Modern terminal	JT	C	14187	A	ND6	Non-synonymous	G163C	1	0.07	0.876
Modern terminal	R0	A	14189	G	ND6	Non-synonymous	V162A	0.987	0.22	0.598
Modern terminal	R0	A	14189	G	ND6	Non-synonymous	V162A	0.987	0.22	0.598
Modern terminal	R0	A	14189	G	ND6	Non-synonymous	V162A	0.987	0.22	0.598

Pre-terminal	R0	A	14189	G	ND6	Non-synonymous	V162A	0.987	0.22	0.598
Pre-terminal	R0	A	14189	G	ND6	Non-synonymous	V162A	0.987	0.22	0.598
Modern terminal	U	A	14189	G	ND6	Non-synonymous	V162A	0.987	0.22	0.598
Pre-terminal	U	A	14189	G	ND6	Non-synonymous	V162A	0.987	0.22	0.598
Modern terminal	JT	A	14189	G	ND6	Non-synonymous	V162A	0.987	0.22	0.598
Modern terminal	JT	A	14189	G	ND6	Non-synonymous	V162A	0.987	0.22	0.598
Ancient terminal	N1	C	14190	A	ND6	Non-synonymous	V162F	0.976	0.01	0.781
Modern terminal	U	A	14191	T	ND6	Non-synonymous	F161L	0.148	0.43	0.78
Modern terminal	U	A	14191	T	ND6	Non-synonymous	F161L	0.148	0.43	0.78
Modern terminal	R0	A	14193	G	ND6	Non-synonymous	F161L	0.148	0.43	0.78
Pre-terminal	R0	A	14193	G	ND6	Non-synonymous	F161L	0.148	0.43	0.78
Pre-terminal	X	A	14193	G	ND6	Non-synonymous	F161L	0.148	0.43	0.78
Modern terminal	R0	G	14198	A	ND6	Non-synonymous	T159M	0.999	0.11	0.517
Modern terminal	R0	G	14198	A	ND6	Non-synonymous	T159M	0.999	0.11	0.517
Modern terminal	R0	G	14198	A	ND6	Non-synonymous	T159M	0.999	0.11	0.517
Modern terminal	R0	G	14198	A	ND6	Non-synonymous	T159M	0.999	0.11	0.517
Modern terminal	R0	G	14198	A	ND6	Non-synonymous	T159M	0.999	0.11	0.517
Modern terminal	R0	G	14198	A	ND6	Non-synonymous	T159M	0.999	0.11	0.517
Modern terminal	R0	G	14198	A	ND6	Non-synonymous	T159M	0.999	0.11	0.517
Modern terminal	R0	G	14198	A	ND6	Non-synonymous	T159M	0.999	0.11	0.517
Modern terminal	R0	G	14198	A	ND6	Non-synonymous	T159M	0.999	0.11	0.517
Pre-terminal	R0	G	14198	A	ND6	Non-synonymous	T159M	0.999	0.11	0.517
Pre-terminal	R0	G	14198	A	ND6	Non-synonymous	T159M	0.999	0.11	0.517
Modern terminal	U	G	14198	A	ND6	Non-synonymous	T159M	0.999	0.11	0.517
Modern terminal	U	G	14198	A	ND6	Non-synonymous	T159M	0.999	0.11	0.517
Modern terminal	U	G	14198	A	ND6	Non-synonymous	T159M	0.999	0.11	0.517
Modern terminal	U	G	14198	A	ND6	Non-synonymous	T159M	0.999	0.11	0.517
Modern terminal	U	G	14198	A	ND6	Non-synonymous	T159M	0.999	0.11	0.517
Modern terminal	U	G	14198	A	ND6	Non-synonymous	T159M	0.999	0.11	0.517
Pre-terminal	U	G	14198	A	ND6	Non-synonymous	T159M	0.999	0.11	0.517
Ancient terminal	JT	G	14198	A	ND6	Non-synonymous	T159M	0.999	0.11	0.517
Modern terminal	JT	G	14198	A	ND6	Non-synonymous	T159M	0.999	0.11	0.517
Modern terminal	JT	G	14198	A	ND6	Non-synonymous	T159M	0.999	0.11	0.517
Modern terminal	JT	G	14198	A	ND6	Non-synonymous	T159M	0.999	0.11	0.517
Modern terminal	JT	G	14198	A	ND6	Non-synonymous	T159M	0.999	0.11	0.517
Modern terminal	R0	T	14199	C	ND6	Non-synonymous	T159A	0.015	0.11	0.468
Modern terminal	R0	T	14199	C	ND6	Non-synonymous	T159A	0.015	0.11	0.468
Modern terminal	R0	T	14199	C	ND6	Non-synonymous	T159A	0.015	0.11	0.468
Pre-terminal	U	T	14199	C	ND6	Non-synonymous	T159A	0.015	0.11	0.468
Modern terminal	JT	T	14199	G	ND6	Non-synonymous	T159P	0.999	0.11	0.826
Ancient terminal	R0	G	14207	A	ND6	Non-synonymous	T156I	0.375	0.05	0.603
Modern terminal	R0	G	14207	A	ND6	Non-synonymous	T156I	0.375	0.05	0.603
Modern terminal	R0	G	14207	A	ND6	Non-synonymous	T156I	0.375	0.05	0.603
Modern terminal	R0	G	14207	A	ND6	Non-synonymous	T156I	0.375	0.05	0.603
Modern terminal	R0	G	14207	A	ND6	Non-synonymous	T156I	0.375	0.05	0.603
Modern terminal	R0	G	14207	A	ND6	Non-synonymous	T156I	0.375	0.05	0.603
Modern terminal	R0	G	14207	A	ND6	Non-synonymous	T156I	0.375	0.05	0.603
Modern terminal	R0	G	14207	A	ND6	Non-synonymous	T156I	0.375	0.05	0.603
Pre-terminal	R0	G	14207	A	ND6	Non-synonymous	T156I	0.375	0.05	0.603
Modern terminal	U	G	14207	A	ND6	Non-synonymous	T156I	0.375	0.05	0.603

Modern terminal	U	G	14207	A	ND6	Non-synonymous	T156I	0.375	0.05	0.603
Modern terminal	U	G	14207	A	ND6	Non-synonymous	T156I	0.375	0.05	0.603
Modern terminal	JT	G	14207	A	ND6	Non-synonymous	T156I	0.375	0.05	0.603
Modern terminal	JT	G	14207	A	ND6	Non-synonymous	T156I	0.375	0.05	0.603
Modern terminal	JT	G	14207	A	ND6	Non-synonymous	T156I	0.375	0.05	0.603
Modern terminal	JT	G	14207	A	ND6	Non-synonymous	T156I	0.375	0.05	0.603
Pre-terminal	JT	G	14207	A	ND6	Non-synonymous	T156I	0.375	0.05	0.603
Modern terminal	N1	G	14207	A	ND6	Non-synonymous	T156I	0.375	0.05	0.603
Modern terminal	X	G	14207	A	ND6	Non-synonymous	T156I	0.375	0.05	0.603
Modern terminal	R0	T	14208	C	ND6	Non-synonymous	T156A	0.002	0.57	0.203
Modern terminal	R0	T	14208	C	ND6	Non-synonymous	T156A	0.002	0.57	0.203
Modern terminal	R0	T	14208	C	ND6	Non-synonymous	T156A	0.002	0.57	0.203
Modern terminal	R0	T	14208	C	ND6	Non-synonymous	T156A	0.002	0.57	0.203
Pre-terminal	R0	T	14208	C	ND6	Non-synonymous	T156A	0.002	0.57	0.203
Modern terminal	U	T	14208	C	ND6	Non-synonymous	T156A	0.002	0.57	0.203
Modern terminal	U	T	14208	C	ND6	Non-synonymous	T156A	0.002	0.57	0.203
Modern terminal	U	T	14208	C	ND6	Non-synonymous	T156A	0.002	0.57	0.203
Pre-terminal	U	T	14208	C	ND6	Non-synonymous	T156A	0.002	0.57	0.203
Modern terminal	N2	T	14208	C	ND6	Non-synonymous	T156A	0.002	0.57	0.203
Modern terminal	U	A	14210	G	ND6	Non-synonymous	V155A	1	0.01	0.735
Modern terminal	R0	C	14211	T	ND6	Non-synonymous	V155I	0.988	0.3	0.48
Modern terminal	R0	C	14211	T	ND6	Non-synonymous	V155I	0.988	0.3	0.48
Modern terminal	U	C	14211	T	ND6	Non-synonymous	V155I	0.988	0.3	0.48
Modern terminal	R0	A	14220	T	ND6	Non-synonymous	L152N	1	0.01	0.923
Ancient terminal	U	C	14225	T	ND6	Non-synonymous	R150H	0.982	0.54	0.392
Modern terminal	R0	G	14226	A	ND6	Non-synonymous	R150C	0.37	0.18	0.46
Modern terminal	R0	G	14226	A	ND6	Non-synonymous	R150C	0.37	0.18	0.46
Modern terminal	R0	G	14226	A	ND6	Non-synonymous	R150C	0.37	0.18	0.46
Modern terminal	R0	G	14226	A	ND6	Non-synonymous	R150C	0.37	0.18	0.46
Modern terminal	R0	G	14226	A	ND6	Non-synonymous	R150C	0.37	0.18	0.46
Modern terminal	R0	G	14226	A	ND6	Non-synonymous	R150C	0.37	0.18	0.46
Modern terminal	R0	G	14226	A	ND6	Non-synonymous	R150C	0.37	0.18	0.46
Pre-terminal	R0	G	14226	A	ND6	Non-synonymous	R150C	0.37	0.18	0.46
Ancient terminal	U	G	14226	A	ND6	Non-synonymous	R150C	0.37	0.18	0.46
Modern terminal	U	G	14226	A	ND6	Non-synonymous	R150C	0.37	0.18	0.46
Modern terminal	U	G	14226	A	ND6	Non-synonymous	R150C	0.37	0.18	0.46
Modern terminal	JT	G	14226	A	ND6	Non-synonymous	R150C	0.37	0.18	0.46
Modern terminal	N1	G	14226	A	ND6	Non-synonymous	R150C	0.37	0.18	0.46
Pre-terminal	N1	G	14226	A	ND6	Non-synonymous	R150C	0.37	0.18	0.46
Ancient terminal	N2	G	14226	A	ND6	Non-synonymous	R150C	0.37	0.18	0.46
Modern terminal	N2	A	14233	C	ND6	Non-synonymous	D147E	0.285	0.03	0.529
Modern terminal	R0	G	14249	A	ND6	Non-synonymous	A142V	0.915	1	0.231
Modern terminal	R0	G	14249	A	ND6	Non-synonymous	A142V	0.915	1	0.231
Pre-terminal	R0	G	14249	A	ND6	Non-synonymous	A142V	0.915	1	0.231
Ancient terminal	U	G	14249	A	ND6	Non-synonymous	A142V	0.915	1	0.231
Modern terminal	U	G	14249	A	ND6	Non-synonymous	A142V	0.915	1	0.231
Modern terminal	U	G	14249	A	ND6	Non-synonymous	A142V	0.915	1	0.231
Modern terminal	U	G	14249	A	ND6	Non-synonymous	A142V	0.915	1	0.231
Modern terminal	U	G	14249	A	ND6	Non-synonymous	A142V	0.915	1	0.231
Modern terminal	U	G	14249	A	ND6	Non-synonymous	A142V	0.915	1	0.231

Modern terminal	U	A	14255	G	ND6	Non-synonymous	I140T	0.414	0.4	0.555
Modern terminal	R0	T	14256	C	ND6	Non-synonymous	I140V	0.509	1	0.215
Modern terminal	R0	T	14256	C	ND6	Non-synonymous	I140V	0.509	1	0.215
Pre-terminal	R0	T	14256	C	ND6	Non-synonymous	I140V	0.509	1	0.215
Ancient terminal	U	T	14256	C	ND6	Non-synonymous	I140V	0.509	1	0.215
Modern terminal	U	T	14256	C	ND6	Non-synonymous	I140V	0.509	1	0.215
Modern terminal	JT	T	14256	C	ND6	Non-synonymous	I140V	0.509	1	0.215
Modern terminal	R0	G	14258	A	ND6	Non-synonymous	P139L	0.647	0.63	0.404
Modern terminal	R0	G	14258	A	ND6	Non-synonymous	P139L	0.647	0.63	0.404
Pre-terminal	R0	G	14258	A	ND6	Non-synonymous	P139L	0.647	0.63	0.404
Pre-terminal	R0	G	14258	A	ND6	Non-synonymous	P139L	0.647	0.63	0.404
Pre-terminal	R0	G	14258	A	ND6	Non-synonymous	P139L	0.647	0.63	0.404
Pre-terminal	R0	G	14258	A	ND6	Non-synonymous	P139L	0.647	0.63	0.404
Modern terminal	U	G	14258	A	ND6	Non-synonymous	P139L	0.647	0.63	0.404
Modern terminal	U	G	14258	A	ND6	Non-synonymous	P139L	0.647	0.63	0.404
Pre-terminal	U	G	14258	A	ND6	Non-synonymous	P139L	0.647	0.63	0.404
Pre-terminal	U	G	14258	A	ND6	Non-synonymous	P139L	0.647	0.63	0.404
Pre-terminal	U	G	14258	A	ND6	Non-synonymous	P139L	0.647	0.63	0.404
Modern terminal	JT	G	14258	A	ND6	Non-synonymous	P139L	0.647	0.63	0.404
Modern terminal	X	G	14258	A	ND6	Non-synonymous	P139L	0.647	0.63	0.404
Pre-terminal	N2	G	14258	A	ND6	Non-synonymous	P139L	0.647	0.63	0.404
Modern terminal	R0	G	14259	A	ND6	Non-synonymous	P139S	0.007	0.41	0.289
Modern terminal	R0	G	14259	A	ND6	Non-synonymous	P139S	0.007	0.41	0.289
Modern terminal	R0	G	14259	A	ND6	Non-synonymous	P139S	0.007	0.41	0.289
Modern terminal	R0	G	14259	A	ND6	Non-synonymous	P139S	0.007	0.41	0.289
Modern terminal	R0	G	14259	A	ND6	Non-synonymous	P139S	0.007	0.41	0.289
Modern terminal	R0	G	14259	A	ND6	Non-synonymous	P139S	0.007	0.41	0.289
Modern terminal	R0	G	14259	A	ND6	Non-synonymous	P139S	0.007	0.41	0.289
Pre-terminal	R0	G	14259	A	ND6	Non-synonymous	P139S	0.007	0.41	0.289
Pre-terminal	R0	G	14259	A	ND6	Non-synonymous	P139S	0.007	0.41	0.289
Pre-terminal	R0	G	14259	A	ND6	Non-synonymous	P139S	0.007	0.41	0.289
Modern terminal	U	G	14259	A	ND6	Non-synonymous	P139S	0.007	0.41	0.289
Modern terminal	U	G	14259	A	ND6	Non-synonymous	P139S	0.007	0.41	0.289
Modern terminal	U	G	14259	A	ND6	Non-synonymous	P139S	0.007	0.41	0.289
Modern terminal	U	G	14259	A	ND6	Non-synonymous	P139S	0.007	0.41	0.289
Pre-terminal	U	G	14259	A	ND6	Non-synonymous	P139S	0.007	0.41	0.289
Pre-terminal	U	G	14259	A	ND6	Non-synonymous	P139S	0.007	0.41	0.289
Modern terminal	JT	G	14259	A	ND6	Non-synonymous	P139S	0.007	0.41	0.289
Modern terminal	JT	G	14259	A	ND6	Non-synonymous	P139S	0.007	0.41	0.289
Modern terminal	JT	G	14259	A	ND6	Non-synonymous	P139S	0.007	0.41	0.289
Pre-terminal	JT	G	14259	A	ND6	Non-synonymous	P139S	0.007	0.41	0.289
Ancient terminal	N1	G	14259	A	ND6	Non-synonymous	P139S	0.007	0.41	0.289
Modern terminal	N1	G	14259	A	ND6	Non-synonymous	P139S	0.007	0.41	0.289
Modern terminal	U	C	14267	T	ND6	Non-synonymous	R136Q	1	0.44	0.511
Pre-terminal	R0	T	14271	C	ND6	Non-synonymous	I135V	0.002	0.92	0.187
Modern terminal	U	T	14271	C	ND6	Non-synonymous	I135V	0.002	0.92	0.187
Pre-terminal	JT	T	14271	C	ND6	Non-synonymous	I135V	0.002	0.92	0.187
Modern terminal	U	C	14272	G	ND6	Non-synonymous	L134F	0.998	0.6	0.397
Modern terminal	U	C	14272	G	ND6	Non-synonymous	L134F	0.998	0.6	0.397
Modern terminal	JT	C	14272	G	ND6	Non-synonymous	L134F	0.998	0.6	0.397

Pre-terminal	R0	C	14276	T	ND6	Non-synonymous	G133E	0.964	0.28	0.85
Modern terminal	R0	G	14279	A	ND6	Non-synonymous	S132L	0.005	0.7	0.58
Modern terminal	R0	G	14279	A	ND6	Non-synonymous	S132L	0.005	0.7	0.58
Modern terminal	U	G	14279	C	ND6	Non-synonymous	S132W	0.997	0.19	0.757
Modern terminal	JT	G	14279	A	ND6	Non-synonymous	S132L	0.005	0.7	0.58
Ancient terminal	R0	A	14280	G	ND6	Non-synonymous	S132P	0	0.28	0.667
Modern terminal	R0	A	14280	G	ND6	Non-synonymous	S132P	0	0.28	0.667
Pre-terminal	R0	A	14280	G	ND6	Non-synonymous	S132P	0	0.28	0.667
Modern terminal	U	A	14280	G	ND6	Non-synonymous	S132P	0	0.28	0.667
Modern terminal	U	A	14280	G	ND6	Non-synonymous	S132P	0	0.28	0.667
Modern terminal	JT	A	14280	G	ND6	Non-synonymous	S132P	0	0.28	0.667
Modern terminal	U	C	14282	T	ND6	Non-synonymous	G131E	0.964	0.55	0.612
Ancient terminal	R0	C	14284	A	ND6	Non-synonymous	E130D	0.401	0.62	0.362
Modern terminal	R0	T	14298	C	ND6	Non-synonymous	I126V	0.01	1	0.296
Ancient terminal	N2	C	14303	A	ND6	Non-synonymous	W124L	1	0.04	0.895
Pre-terminal	JT	C	14306	T	ND6	Non-synonymous	S123N	0	0.28	0.393
Modern terminal	U	A	14312	G	ND6	Non-synonymous	V121A	0.035	0.13	0.451
Modern terminal	JT	T	14316	C	ND6	Non-synonymous	S120G	0.597	1	0.196
Modern terminal	JT	T	14316	C	ND6	Non-synonymous	S120G	0.597	1	0.196
Pre-terminal	JT	T	14316	C	ND6	Non-synonymous	S120G	0.597	1	0.196
Modern terminal	R0	T	14318	C	ND6	Non-synonymous	N119S	0.034	0.49	0.366
Modern terminal	R0	T	14318	C	ND6	Non-synonymous	N119S	0.034	0.49	0.366
Modern terminal	N1	T	14318	C	ND6	Non-synonymous	N119S	0.034	0.49	0.366
Modern terminal	N2	T	14318	C	ND6	Non-synonymous	N119S	0.034	0.49	0.366
Ancient terminal	R0	T	14319	C	ND6	Non-synonymous	N119D	0.003	0.85	0.374
Ancient terminal	R0	T	14319	C	ND6	Non-synonymous	N119D	0.003	0.85	0.374
Modern terminal	R0	T	14319	C	ND6	Non-synonymous	N119D	0.003	0.85	0.374
Modern terminal	R0	T	14319	C	ND6	Non-synonymous	N119D	0.003	0.85	0.374
Modern terminal	R0	T	14319	C	ND6	Non-synonymous	N119D	0.003	0.85	0.374
Modern terminal	R0	T	14319	C	ND6	Non-synonymous	N119D	0.003	0.85	0.374
Pre-terminal	R0	T	14319	C	ND6	Non-synonymous	N119D	0.003	0.85	0.374
Pre-terminal	R0	T	14319	C	ND6	Non-synonymous	N119D	0.003	0.85	0.374
Pre-terminal	R0	T	14319	C	ND6	Non-synonymous	N119D	0.003	0.85	0.374
Modern terminal	U	T	14319	C	ND6	Non-synonymous	N119D	0.003	0.85	0.374
Modern terminal	U	T	14319	C	ND6	Non-synonymous	N119D	0.003	0.85	0.374
Pre-terminal	U	T	14319	C	ND6	Non-synonymous	N119D	0.003	0.85	0.374
Pre-terminal	U	T	14319	C	ND6	Non-synonymous	N119D	0.003	0.85	0.374
Modern terminal	JT	T	14319	C	ND6	Non-synonymous	N119D	0.003	0.85	0.374
Modern terminal	JT	T	14319	C	ND6	Non-synonymous	N119D	0.003	0.85	0.374
Modern terminal	JT	T	14319	C	ND6	Non-synonymous	N119D	0.003	0.85	0.374
Modern terminal	JT	T	14319	C	ND6	Non-synonymous	N119D	0.003	0.85	0.374
Pre-terminal	JT	T	14319	C	ND6	Non-synonymous	N119D	0.003	0.85	0.374
Modern terminal	X	T	14319	C	ND6	Non-synonymous	N119D	0.003	0.85	0.374
Pre-terminal	X	T	14319	C	ND6	Non-synonymous	N119D	0.003	0.85	0.374
Modern terminal	N2	T	14319	C	ND6	Non-synonymous	N119D	0.003	0.85	0.374
Modern terminal	R0	T	14325	C	ND6	Non-synonymous	N117D	0.869	0.61	0.273
Modern terminal	R0	T	14325	C	ND6	Non-synonymous	N117D	0.869	0.61	0.273
Modern terminal	R0	T	14325	C	ND6	Non-synonymous	N117D	0.869	0.61	0.273
Modern terminal	R0	T	14325	C	ND6	Non-synonymous	N117D	0.869	0.61	0.273
Pre-terminal	R0	T	14325	C	ND6	Non-synonymous	N117D	0.869	0.61	0.273

Pre-terminal	R0	T	14325	C	ND6	Non-synonymous	N117D	0.869	0.61	0.273
Pre-terminal	R0	T	14325	C	ND6	Non-synonymous	N117D	0.869	0.61	0.273
Pre-terminal	R0	T	14325	C	ND6	Non-synonymous	N117D	0.869	0.61	0.273
Modern terminal	U	T	14325	C	ND6	Non-synonymous	N117D	0.869	0.61	0.273
Pre-terminal	U	T	14325	C	ND6	Non-synonymous	N117D	0.869	0.61	0.273
Pre-terminal	U	T	14325	C	ND6	Non-synonymous	N117D	0.869	0.61	0.273
Ancient terminal	JT	T	14325	C	ND6	Non-synonymous	N117D	0.869	0.61	0.273
Pre-terminal	JT	T	14325	C	ND6	Non-synonymous	N117D	0.869	0.61	0.273
Ancient terminal	X	T	14325	C	ND6	Non-synonymous	N117D	0.869	0.61	0.273
Modern terminal	X	T	14325	C	ND6	Non-synonymous	N117D	0.869	0.61	0.273
Modern terminal	R0	C	14334	T	ND6	Non-synonymous	V114I	0.001	0.64	0.219
Modern terminal	U	C	14334	T	ND6	Non-synonymous	V114I	0.001	0.64	0.219
Modern terminal	JT	C	14334	T	ND6	Non-synonymous	V114I	0.001	0.64	0.219
Modern terminal	JT	C	14334	T	ND6	Non-synonymous	V114I	0.001	0.64	0.219
Pre-terminal	JT	C	14334	T	ND6	Non-synonymous	V114I	0.001	0.64	0.219
Ancient terminal	U	A	14336	G	ND6	Non-synonymous	V113A	0.969	0.95	0.25
Pre-terminal	R0	A	14339	T	ND6	Non-synonymous	V112E	0.866	0.25	0.791
Ancient terminal	U	C	14352	A	ND6	Nonsense				
Modern terminal	JT	A	14357	G	ND6	Non-synonymous	V106A	0.005	0.54	0.38
Ancient terminal	U	C	14358	A	ND6	Non-synonymous	V106L	0	0.85	0.277
Pre-terminal	U	C	14358	T	ND6	Non-synonymous	V106M	0.003	0.33	0.258
Modern terminal	JT	C	14368	G	ND6	Non-synonymous	L102F	0.927	0.13	0.521
Modern terminal	U	C	14372	A	ND6	Non-synonymous	G101V	0.001	0.42	0.654
Modern terminal	R0	A	14375	G	ND6	Non-synonymous	V100A	0.012	0.09	0.431
Modern terminal	R0	G	14384	A	ND6	Non-synonymous	A97V	0	0.28	0.31
Modern terminal	R0	G	14384	A	ND6	Non-synonymous	A97V	0	0.28	0.31
Modern terminal	R0	G	14384	A	ND6	Non-synonymous	A97V	0	0.28	0.31
Modern terminal	R0	G	14384	A	ND6	Non-synonymous	A97V	0	0.28	0.31
Modern terminal	R0	G	14384	A	ND6	Non-synonymous	A97V	0	0.28	0.31
Pre-terminal	R0	G	14384	A	ND6	Non-synonymous	A97V	0	0.28	0.31
Pre-terminal	R0	G	14384	A	ND6	Non-synonymous	A97V	0	0.28	0.31
Pre-terminal	R0	G	14384	A	ND6	Non-synonymous	A97V	0	0.28	0.31
Modern terminal	U	G	14384	A	ND6	Non-synonymous	A97V	0	0.28	0.31
Modern terminal	U	G	14384	A	ND6	Non-synonymous	A97V	0	0.28	0.31
Modern terminal	U	G	14384	A	ND6	Non-synonymous	A97V	0	0.28	0.31
Modern terminal	U	G	14384	A	ND6	Non-synonymous	A97V	0	0.28	0.31
Modern terminal	U	G	14384	A	ND6	Non-synonymous	A97V	0	0.28	0.31
Modern terminal	U	G	14384	A	ND6	Non-synonymous	A97V	0	0.28	0.31
Modern terminal	U	G	14384	A	ND6	Non-synonymous	A97V	0	0.28	0.31
Modern terminal	U	G	14384	A	ND6	Non-synonymous	A97V	0	0.28	0.31
Pre-terminal	U	G	14384	A	ND6	Non-synonymous	A97V	0	0.28	0.31
Modern terminal	JT	G	14384	A	ND6	Non-synonymous	A97V	0	0.28	0.31
Modern terminal	JT	G	14384	A	ND6	Non-synonymous	A97V	0	0.28	0.31
Ancient terminal	X	G	14384	A	ND6	Non-synonymous	A97V	0	0.28	0.31
Modern terminal	R0	T	14386	G	ND6	Non-synonymous	L96F	0.024	0.07	0.554
Ancient terminal	JT	C	14391	T	ND6	Nonsense				
Ancient terminal	X	A	14393	G	ND6	Non-synonymous	V94A	0.024	0.2	0.447
Modern terminal	R0	C	14394	T	ND6	Non-synonymous	V94M	0.005	0.17	0.297
Modern terminal	JT	C	14394	T	ND6	Non-synonymous	V94M	0.005	0.17	0.297
Pre-terminal	R0	A	14405	G	ND6	Non-synonymous	V90A	0.002	0.4	0.183

Pre-terminal	JT	A	14405	G	ND6	Non-synonymous	V90A	0.002	0.4	0.183
Pre-terminal	N1	A	14405	G	ND6	Non-synonymous	V90A	0.002	0.4	0.183
Modern terminal	R0	C	14412	T	ND6	Non-synonymous	V88I	0.007	0.23	0.191
Modern terminal	U	A	14417	G	ND6	Non-synonymous	V86A	0.009	0.62	0.247
Modern terminal	U	A	14417	G	ND6	Non-synonymous	V86A	0.009	0.62	0.247
Ancient terminal	U	C	14418	T	ND6	Non-synonymous	V86I	0	0.43	0.112
Pre-terminal	R0	C	14420	T	ND6	Non-synonymous	G85E	0.993	0.01	0.636
Modern terminal	U	C	14420	T	ND6	Non-synonymous	G85E	0.993	0.01	0.636
Pre-terminal	U	C	14420	T	ND6	Non-synonymous	G85E	0.993	0.01	0.636
Modern terminal	R0	C	14433	T	ND6	Non-synonymous	A81T	0.002	0.84	0.132
Pre-terminal	R0	C	14433	T	ND6	Non-synonymous	A81T	0.002	0.84	0.132
Modern terminal	JT	C	14433	T	ND6	Non-synonymous	A81T	0.002	0.84	0.132
Pre-terminal	JT	C	14433	T	ND6	Non-synonymous	A81T	0.002	0.84	0.132
Modern terminal	N2	C	14433	T	ND6	Non-synonymous	A81T	0.002	0.84	0.132
Ancient terminal	R0	T	14435	A	ND6	Non-synonymous	E80V	0.983	0	0.813
Ancient terminal	U	C	14448	A	ND6	Nonsense				
Pre-terminal	JT	G	14459	A	ND6	Non-synonymous	A72V	1	0	0.872
Modern terminal	R0	G	14462	A	ND6	Non-synonymous	T71M	0.175	0.01	0.809
Modern terminal	R0	A	14477	G	ND6	Non-synonymous	V66A	1	0	0.856
Pre-terminal	R0	A	14477	G	ND6	Non-synonymous	V66A	1	0	0.856
Ancient terminal	JT	C	14478	A	ND6	Non-synonymous	V66F	1	0	0.89
Ancient terminal	X	C	14478	T	ND6	Non-synonymous	V66I	0.98	0	0.778
Modern terminal	JT	C	14482	A	ND6	Non-synonymous	M64I	0.975	0.02	0.778
Modern terminal	N1	C	14482	A	ND6	Non-synonymous	M64I	0.975	0.02	0.778
Ancient terminal	R0	T	14484	C	ND6	Non-synonymous	M64V	1	0.01	0.787
Modern terminal	R0	T	14484	C	ND6	Non-synonymous	M64V	1	0.01	0.787
Modern terminal	R0	T	14484	C	ND6	Non-synonymous	M64V	1	0.01	0.787
Modern terminal	R0	T	14484	C	ND6	Non-synonymous	M64V	1	0.01	0.787
Modern terminal	R0	T	14484	C	ND6	Non-synonymous	M64V	1	0.01	0.787
Modern terminal	R0	T	14484	C	ND6	Non-synonymous	M64V	1	0.01	0.787
Modern terminal	R0	T	14484	C	ND6	Non-synonymous	M64V	1	0.01	0.787
Modern terminal	R0	T	14484	C	ND6	Non-synonymous	M64V	1	0.01	0.787
Modern terminal	R0	T	14484	C	ND6	Non-synonymous	M64V	1	0.01	0.787
Modern terminal	R0	T	14484	C	ND6	Non-synonymous	M64V	1	0.01	0.787
Modern terminal	R0	T	14484	C	ND6	Non-synonymous	M64V	1	0.01	0.787
Modern terminal	R0	T	14484	C	ND6	Non-synonymous	M64V	1	0.01	0.787
Pre-terminal	R0	T	14484	C	ND6	Non-synonymous	M64V	1	0.01	0.787
Modern terminal	U	T	14484	C	ND6	Non-synonymous	M64V	1	0.01	0.787
Modern terminal	U	T	14484	C	ND6	Non-synonymous	M64V	1	0.01	0.787
Modern terminal	U	T	14484	C	ND6	Non-synonymous	M64V	1	0.01	0.787
Modern terminal	U	T	14484	C	ND6	Non-synonymous	M64V	1	0.01	0.787
Modern terminal	U	T	14484	C	ND6	Non-synonymous	M64V	1	0.01	0.787
Modern terminal	U	T	14484	C	ND6	Non-synonymous	M64V	1	0.01	0.787
Modern terminal	U	T	14484	C	ND6	Non-synonymous	M64V	1	0.01	0.787
Modern terminal	U	T	14484	C	ND6	Non-synonymous	M64V	1	0.01	0.787
Modern terminal	U	T	14484	C	ND6	Non-synonymous	M64V	1	0.01	0.787
Pre-terminal	U	T	14484	C	ND6	Non-synonymous	M64V	1	0.01	0.787
Pre-terminal	U	T	14484	C	ND6	Non-synonymous	M64V	1	0.01	0.787
Pre-terminal	U	T	14484	C	ND6	Non-synonymous	M64V	1	0.01	0.787
Modern terminal	JT	T	14484	C	ND6	Non-synonymous	M64V	1	0.01	0.787

Modern terminal	JT	T	14484	C	ND6	Non-synonymous	M64V	1	0.01	0.787
Modern terminal	JT	T	14484	C	ND6	Non-synonymous	M64V	1	0.01	0.787
Modern terminal	JT	T	14484	C	ND6	Non-synonymous	M64V	1	0.01	0.787
Modern terminal	JT	T	14484	C	ND6	Non-synonymous	M64V	1	0.01	0.787
Modern terminal	JT	T	14484	C	ND6	Non-synonymous	M64V	1	0.01	0.787
Modern terminal	JT	T	14484	C	ND6	Non-synonymous	M64V	1	0.01	0.787
Modern terminal	N1	T	14484	C	ND6	Non-synonymous	M64V	1	0.01	0.787
Modern terminal	N1	T	14484	C	ND6	Non-synonymous	M64V	1	0.01	0.787
Modern terminal	N1	T	14484	C	ND6	Non-synonymous	M64V	1	0.01	0.787
Modern terminal	N2	T	14484	C	ND6	Non-synonymous	M64V	1	0.01	0.787
Modern terminal	R0	A	14495	G	ND6	Non-synonymous	L60S	1	0	0.864
Ancient terminal	U	T	14498	A	ND6	Non-synonymous	Y59F	1	0	0.621
Pre-terminal	R0	A	14501	G	ND6	Non-synonymous	I58T	0.979	0	0.774
Ancient terminal	U	A	14501	G	ND6	Non-synonymous	I58T	0.979	0	0.774
Modern terminal	U	A	14501	G	ND6	Non-synonymous	I58T	0.979	0	0.774
Pre-terminal	JT	A	14501	G	ND6	Non-synonymous	I58T	0.979	0	0.774
Ancient terminal	R0	T	14502	C	ND6	Non-synonymous	I58V	0.004	0.39	0.364
Modern terminal	R0	T	14502	C	ND6	Non-synonymous	I58V	0.004	0.39	0.364
Modern terminal	R0	T	14502	C	ND6	Non-synonymous	I58V	0.004	0.39	0.364
Modern terminal	R0	T	14502	C	ND6	Non-synonymous	I58V	0.004	0.39	0.364
Modern terminal	R0	T	14502	C	ND6	Non-synonymous	I58V	0.004	0.39	0.364
Modern terminal	R0	T	14502	C	ND6	Non-synonymous	I58V	0.004	0.39	0.364
Modern terminal	R0	T	14502	C	ND6	Non-synonymous	I58V	0.004	0.39	0.364
Pre-terminal	R0	T	14502	C	ND6	Non-synonymous	I58V	0.004	0.39	0.364
Pre-terminal	R0	T	14502	C	ND6	Non-synonymous	I58V	0.004	0.39	0.364
Pre-terminal	R0	T	14502	C	ND6	Non-synonymous	I58V	0.004	0.39	0.364
Pre-terminal	R0	T	14502	C	ND6	Non-synonymous	I58V	0.004	0.39	0.364
Pre-terminal	R0	T	14502	C	ND6	Non-synonymous	I58V	0.004	0.39	0.364
Pre-terminal	R0	T	14502	C	ND6	Non-synonymous	I58V	0.004	0.39	0.364
Modern terminal	U	T	14502	C	ND6	Non-synonymous	I58V	0.004	0.39	0.364
Modern terminal	U	T	14502	C	ND6	Non-synonymous	I58V	0.004	0.39	0.364
Modern terminal	U	T	14502	C	ND6	Non-synonymous	I58V	0.004	0.39	0.364
Modern terminal	U	T	14502	C	ND6	Non-synonymous	I58V	0.004	0.39	0.364
Pre-terminal	U	T	14502	C	ND6	Non-synonymous	I58V	0.004	0.39	0.364
Modern terminal	JT	T	14502	C	ND6	Non-synonymous	I58V	0.004	0.39	0.364
Modern terminal	JT	T	14502	C	ND6	Non-synonymous	I58V	0.004	0.39	0.364
Modern terminal	JT	T	14502	C	ND6	Non-synonymous	I58V	0.004	0.39	0.364
Modern terminal	JT	T	14502	C	ND6	Non-synonymous	I58V	0.004	0.39	0.364
Modern terminal	JT	T	14502	C	ND6	Non-synonymous	I58V	0.004	0.39	0.364
Modern terminal	JT	T	14502	C	ND6	Non-synonymous	I58V	0.004	0.39	0.364
Pre-terminal	JT	T	14502	C	ND6	Non-synonymous	I58V	0.004	0.39	0.364
Pre-terminal	JT	T	14502	C	ND6	Non-synonymous	I58V	0.004	0.39	0.364
Ancient terminal	N1	T	14502	C	ND6	Non-synonymous	I58V	0.004	0.39	0.364
Pre-terminal	N1	T	14502	C	ND6	Non-synonymous	I58V	0.004	0.39	0.364
Ancient terminal	R0	T	14503	A	ND6	Non-synonymous	L57F	0.988	0	0.806
Ancient terminal	R0	A	14504	T	ND6	Nonsense				
Ancient terminal	JT	A	14506	T	ND6	Non-synonymous	F56L	1	0	0.816
Ancient terminal	JT	A	14507	T	ND6	Non-synonymous	F56Y	0.999	0	0.749
Ancient terminal	JT	A	14508	G	ND6	Non-synonymous	F56L	1	0	0.816
Pre-terminal	R0	C	14511	T	ND6	Non-synonymous	V55I	0.114	0.02	0.669

Ancient terminal	R0	A	14513	C	ND6	Nonsense				
Modern terminal	U	A	14513	G	ND6	Non-synonymous	M54T	0.841	0.01	0.812
Ancient terminal	JT	A	14513	G	ND6	Non-synonymous	M54T	0.841	0.01	0.812
Ancient terminal	R0	T	14514	C	ND6	Non-synonymous	M54V	0.071	0.47	0.68
Modern terminal	R0	T	14514	C	ND6	Non-synonymous	M54V	0.071	0.47	0.68
Modern terminal	R0	T	14514	C	ND6	Non-synonymous	M54V	0.071	0.47	0.68
Modern terminal	R0	T	14514	C	ND6	Non-synonymous	M54V	0.071	0.47	0.68
Pre-terminal	R0	T	14514	C	ND6	Non-synonymous	M54V	0.071	0.47	0.68
Pre-terminal	R0	T	14514	C	ND6	Non-synonymous	M54V	0.071	0.47	0.68
Ancient terminal	U	T	14514	C	ND6	Non-synonymous	M54V	0.071	0.47	0.68
Modern terminal	U	T	14514	C	ND6	Non-synonymous	M54V	0.071	0.47	0.68
Modern terminal	JT	T	14514	C	ND6	Non-synonymous	M54V	0.071	0.47	0.68
Ancient terminal	JT	A	14516	C	ND6	Non-synonymous	L53W	1	0	0.851
Ancient terminal	R0	C	14519	T	ND6	Non-synonymous	G52D	1	0	0.865
Ancient terminal	N1	A	14522	G	ND6	Non-synonymous	M51T	0.66	0	0.649
Pre-terminal	R0	T	14550	C	ND6	Non-synonymous	I42V	0.004	0.26	0.38
Modern terminal	R0	A	14552	G	ND6	Non-synonymous	V41A	0.009	0.29	0.17
Pre-terminal	R0	A	14552	G	ND6	Non-synonymous	V41A	0.009	0.29	0.17
Pre-terminal	U	A	14552	G	ND6	Non-synonymous	V41A	0.009	0.29	0.17
Modern terminal	JT	A	14552	G	ND6	Non-synonymous	V41A	0.009	0.29	0.17
Modern terminal	JT	A	14552	G	ND6	Non-synonymous	V41A	0.009	0.29	0.17
Modern terminal	JT	A	14552	G	ND6	Non-synonymous	V41A	0.009	0.29	0.17
Pre-terminal	JT	A	14552	G	ND6	Non-synonymous	V41A	0.009	0.29	0.17
Modern terminal	N1	A	14552	G	ND6	Non-synonymous	V41A	0.009	0.29	0.17
Modern terminal	N1	A	14552	G	ND6	Non-synonymous	V41A	0.009	0.29	0.17
Modern terminal	N1	A	14552	G	ND6	Non-synonymous	V41A	0.009	0.29	0.17
Modern terminal	R0	C	14553	T	ND6	Non-synonymous	V41I	0	0.04	0.181
Pre-terminal	R0	C	14553	T	ND6	Non-synonymous	V41I	0	0.04	0.181
Pre-terminal	R0	C	14553	T	ND6	Non-synonymous	V41I	0	0.04	0.181
Modern terminal	R0	A	14561	G	ND6	Non-synonymous	V38A	0.999	0.07	0.577
Ancient terminal	JT	A	14561	G	ND6	Non-synonymous	V38I	0.006	0.15	0.294
Modern terminal	R0	C	14562	T	ND6	Non-synonymous	V38I	0.006	0.15	0.294
Pre-terminal	U	C	14562	T	ND6	Non-synonymous	V38I	0.006	0.15	0.294
Modern terminal	JT	C	14562	T	ND6	Non-synonymous	V38I	0.006	0.15	0.294
Modern terminal	JT	C	14562	T	ND6	Non-synonymous	V38I	0.006	0.15	0.294
Modern terminal	JT	C	14562	T	ND6	Non-synonymous	V38I	0.006	0.15	0.294
Modern terminal	R0	A	14564	G	ND6	Non-synonymous	V37A	0.056	0.35	0.312
Modern terminal	R0	A	14564	G	ND6	Non-synonymous	V37A	0.056	0.35	0.312
Pre-terminal	R0	A	14564	G	ND6	Non-synonymous	V37A	0.056	0.35	0.312
Modern terminal	U	A	14564	G	ND6	Non-synonymous	V37A	0.056	0.35	0.312
Modern terminal	R0	C	14568	T	ND6	Non-synonymous	G36S	1	0.01	0.848
Modern terminal	U	C	14568	T	ND6	Non-synonymous	G36S	1	0.01	0.848
Modern terminal	U	C	14568	T	ND6	Non-synonymous	G36S	1	0.01	0.848
Modern terminal	JT	C	14568	T	ND6	Non-synonymous	G36S	1	0.01	0.848
Pre-terminal	JT	C	14568	T	ND6	Non-synonymous	G36S	1	0.01	0.848
Ancient terminal	R0	A	14573	G	ND6	Non-synonymous	V34A	0.949	0.04	0.681
Modern terminal	R0	A	14573	G	ND6	Non-synonymous	V34A	0.949	0.04	0.681
Modern terminal	R0	C	14574	T	ND6	Non-synonymous	V34I	0	0.13	0.318
Modern terminal	R0	C	14574	T	ND6	Non-synonymous	V34I	0	0.13	0.318
Modern terminal	R0	C	14574	T	ND6	Non-synonymous	V34I	0	0.13	0.318

Pre-terminal	U	C	14574	T	ND6	Non-synonymous	V34I	0	0.13	0.318
Pre-terminal	N1	C	14574	T	ND6	Non-synonymous	V34I	0	0.13	0.318
Modern terminal	R0	T	14577	C	ND6	Non-synonymous	I33V	0	0.66	0.286
Modern terminal	R0	T	14577	C	ND6	Non-synonymous	I33V	0	0.66	0.286
Modern terminal	R0	T	14577	C	ND6	Non-synonymous	I33V	0	0.66	0.286
Modern terminal	R0	T	14577	C	ND6	Non-synonymous	I33V	0	0.66	0.286
Modern terminal	R0	T	14577	C	ND6	Non-synonymous	I33V	0	0.66	0.286
Pre-terminal	R0	T	14577	C	ND6	Non-synonymous	I33V	0	0.66	0.286
Modern terminal	U	T	14577	C	ND6	Non-synonymous	I33V	0	0.66	0.286
Modern terminal	U	T	14577	C	ND6	Non-synonymous	I33V	0	0.66	0.286
Pre-terminal	U	T	14577	G	ND6	Non-synonymous	I33L	0	0.02	0.659
Pre-terminal	U	T	14577	C	ND6	Non-synonymous	I33V	0	0.66	0.286
Pre-terminal	U	T	14577	C	ND6	Non-synonymous	I33V	0	0.66	0.286
Ancient terminal	JT	T	14577	C	ND6	Non-synonymous	I33V	0	0.66	0.286
Modern terminal	JT	T	14577	C	ND6	Non-synonymous	I33V	0	0.66	0.286
Modern terminal	R0	A	14582	G	ND6	Non-synonymous	V31A	0.022	0.96	0.181
Modern terminal	R0	A	14582	G	ND6	Non-synonymous	V31A	0.022	0.96	0.181
Pre-terminal	R0	A	14582	G	ND6	Non-synonymous	V31A	0.022	0.96	0.181
Pre-terminal	R0	A	14582	G	ND6	Non-synonymous	V31A	0.022	0.96	0.181
Modern terminal	U	A	14582	G	ND6	Non-synonymous	V31A	0.022	0.96	0.181
Modern terminal	U	A	14582	G	ND6	Non-synonymous	V31A	0.022	0.96	0.181
Modern terminal	U	A	14582	G	ND6	Non-synonymous	V31A	0.022	0.96	0.181
Modern terminal	JT	A	14582	G	ND6	Non-synonymous	V31A	0.022	0.96	0.181
Modern terminal	JT	A	14582	G	ND6	Non-synonymous	V31A	0.022	0.96	0.181
Pre-terminal	JT	A	14582	G	ND6	Non-synonymous	V31A	0.022	0.96	0.181
Ancient terminal	X	C	14588	T	ND6	Non-synonymous	G29D	1	0	0.822
Ancient terminal	JT	T	14594	C	ND6	Non-synonymous	Y27C	1	0	0.681
Modern terminal	JT	T	14598	C	ND6	Non-synonymous	I26V	0.988	0.33	0.238
Modern terminal	R0	G	14612	T	ND6	Non-synonymous	S21Y	1	0	0.757
Modern terminal	R0	A	14627	G	ND6	Non-synonymous	F16S	1	0	0.735
Modern terminal	R0	A	14633	G	ND6	Non-synonymous	M14T	0.11	0.09	0.291
Modern terminal	R0	T	14634	C	ND6	Non-synonymous	M14V	0.168	1	0.169
Modern terminal	R0	T	14634	C	ND6	Non-synonymous	M14V	0.168	1	0.169
Modern terminal	R0	T	14634	C	ND6	Non-synonymous	M14V	0.168	1	0.169
Pre-terminal	U	T	14634	C	ND6	Non-synonymous	M14V	0.168	1	0.169
Modern terminal	N1	T	14634	C	ND6	Non-synonymous	M14V	0.168	1	0.169
Modern terminal	N2	T	14634	C	ND6	Non-synonymous	M14V	0.168	1	0.169
Modern terminal	U	G	14655	T	ND6	Non-synonymous	L7M	0.997	0.01	0.19
Modern terminal	R0	G	14663	A	ND6	Non-synonymous	A4V	0	0.52	0.183
Ancient terminal	JT	C	14664	T	ND6	Non-synonymous	A4T	0	0.24	0.209
Modern terminal	JT	C	14664	T	ND6	Non-synonymous	A4T	0	0.24	0.209
Modern terminal	U	C	14668	G	ND6	Non-synonymous	M2I	0.007	0.42	0.307
Modern terminal	U	A	14669	G	ND6	Non-synonymous	M2T	0.001	1	0.223
Modern terminal	U	A	14669	G	ND6	Non-synonymous	M2T	0.001	1	0.223
Modern terminal	JT	A	14669	G	ND6	Non-synonymous	M2T	0.001	1	0.223
Modern terminal	X	A	14669	G	ND6	Non-synonymous	M2T	0.001	1	0.223
Modern terminal	R0	A	14750	G	CYTB	Non-synonymous	T2A	0.001	0	0.224
Modern terminal	R0	A	14750	T	CYTB	Non-synonymous	T2S	0.129	0	0.124
Modern terminal	R0	A	14750	G	CYTB	Non-synonymous	T2A	0.001	0	0.224
Modern terminal	R0	A	14750	G	CYTB	Non-synonymous	T2A	0.001	0	0.224

Pre-terminal	R0	A	14750	G	CYTB	Non-synonymous	T2A	0.001	0	0.224
Pre-terminal	R0	A	14750	G	CYTB	Non-synonymous	T2A	0.001	0	0.224
Pre-terminal	R0	A	14750	G	CYTB	Non-synonymous	T2A	0.001	0	0.224
Modern terminal	U	A	14750	G	CYTB	Non-synonymous	T2A	0.001	0	0.224
Modern terminal	U	A	14750	G	CYTB	Non-synonymous	T2A	0.001	0	0.224
Pre-terminal	U	A	14750	G	CYTB	Non-synonymous	T2A	0.001	0	0.224
Modern terminal	JT	A	14750	G	CYTB	Non-synonymous	T2A	0.001	0	0.224
Modern terminal	R0	C	14751	T	CYTB	Non-synonymous	T2I	0.419	0.02	0.191
Modern terminal	R0	C	14751	T	CYTB	Non-synonymous	T2I	0.419	0.02	0.191
Modern terminal	R0	C	14751	T	CYTB	Non-synonymous	T2I	0.419	0.02	0.191
Ancient terminal	U	C	14751	T	CYTB	Non-synonymous	T2I	0.419	0.02	0.191
Ancient terminal	U	C	14751	T	CYTB	Non-synonymous	T2I	0.419	0.02	0.191
Modern terminal	U	C	14751	T	CYTB	Non-synonymous	T2I	0.419	0.02	0.191
Modern terminal	U	C	14751	T	CYTB	Non-synonymous	T2I	0.419	0.02	0.191
Ancient terminal	JT	C	14751	T	CYTB	Non-synonymous	T2I	0.419	0.02	0.191
Modern terminal	JT	C	14751	T	CYTB	Non-synonymous	T2I	0.419	0.02	0.191
Modern terminal	JT	C	14751	T	CYTB	Non-synonymous	T2I	0.419	0.02	0.191
Pre-terminal	JT	C	14751	T	CYTB	Non-synonymous	T2I	0.419	0.02	0.191
Modern terminal	X	C	14751	T	CYTB	Non-synonymous	T2I	0.419	0.02	0.191
Ancient terminal	R0	C	14753	T	CYTB	Non-synonymous	P3S	0.004	0.07	0.196
Pre-terminal	U	C	14753	T	CYTB	Non-synonymous	P3S	0.004	0.07	0.196
Modern terminal	R0	A	14756	G	CYTB	Non-synonymous	M4V	0.067	0	0.115
Modern terminal	U	A	14756	G	CYTB	Non-synonymous	M4V	0.067	0	0.115
Modern terminal	R0	T	14757	C	CYTB	Non-synonymous	M4T	0	0.11	0.108
Modern terminal	R0	T	14757	C	CYTB	Non-synonymous	M4T	0	0.11	0.108
Pre-terminal	R0	T	14757	C	CYTB	Non-synonymous	M4T	0	0.11	0.108
Modern terminal	U	T	14757	C	CYTB	Non-synonymous	M4T	0	0.11	0.108
Modern terminal	U	T	14757	C	CYTB	Non-synonymous	M4T	0	0.11	0.108
Modern terminal	U	T	14757	C	CYTB	Non-synonymous	M4T	0	0.11	0.108
Pre-terminal	U	T	14757	C	CYTB	Non-synonymous	M4T	0	0.11	0.108
Pre-terminal	JT	T	14757	C	CYTB	Non-synonymous	M4T	0	0.11	0.108
Pre-terminal	N1	T	14757	C	CYTB	Non-synonymous	M4T	0	0.11	0.108
Ancient terminal	R0	C	14766	T	CYTB	Non-synonymous	T7I	0	0.01	0.064
Ancient terminal	R0	C	14766	T	CYTB	Non-synonymous	T7I	0	0.01	0.064
Modern terminal	R0	C	14766	T	CYTB	Non-synonymous	T7I	0	0.01	0.064
Modern terminal	R0	C	14766	T	CYTB	Non-synonymous	T7I	0	0.01	0.064
Modern terminal	R0	C	14766	T	CYTB	Non-synonymous	T7I	0	0.01	0.064
Modern terminal	R0	C	14766	T	CYTB	Non-synonymous	T7I	0	0.01	0.064
Modern terminal	R0	C	14766	T	CYTB	Non-synonymous	T7I	0	0.01	0.064
Pre-terminal	R0	C	14766	T	CYTB	Non-synonymous	T7I	0	0.01	0.064
Pre-terminal	R0	C	14766	T	CYTB	Non-synonymous	T7I	0	0.01	0.064
Modern terminal	JT	C	14766	T	CYTB	Non-synonymous	T7I	0	0.01	0.064
Modern terminal	R0	A	14769	G	CYTB	Non-synonymous	N8S	0.99	0	0.102
Modern terminal	R0	A	14769	G	CYTB	Non-synonymous	N8S	0.99	0	0.102
Ancient terminal	U	A	14769	G	CYTB	Non-synonymous	N8S	0.99	0	0.102
Modern terminal	U	A	14769	G	CYTB	Non-synonymous	N8S	0.99	0	0.102
Modern terminal	U	A	14769	G	CYTB	Non-synonymous	N8S	0.99	0	0.102
Modern terminal	U	A	14769	G	CYTB	Non-synonymous	N8S	0.99	0	0.102
Modern terminal	U	A	14769	G	CYTB	Non-synonymous	N8S	0.99	0	0.102
Modern terminal	U	A	14769	G	CYTB	Non-synonymous	N8S	0.99	0	0.102

Modern terminal	U	A	14769	G	CYTB	Non-synonymous	N8S	0.99	0	0.102
Modern terminal	U	A	14769	G	CYTB	Non-synonymous	N8S	0.99	0	0.102
Modern terminal	JT	A	14769	C	CYTB	Non-synonymous	N8T	0.964	0	0.129
Modern terminal	JT	A	14769	C	CYTB	Non-synonymous	N8T	0.964	0	0.129
Modern terminal	JT	A	14769	G	CYTB	Non-synonymous	N8S	0.99	0	0.102
Pre-terminal	JT	A	14769	G	CYTB	Non-synonymous	N8S	0.99	0	0.102
Pre-terminal	JT	A	14769	G	CYTB	Non-synonymous	N8S	0.99	0	0.102
Pre-terminal	JT	A	14769	G	CYTB	Non-synonymous	N8S	0.99	0	0.102
Modern terminal	N1	A	14769	G	CYTB	Non-synonymous	N8S	0.99	0	0.102
Modern terminal	N1	A	14769	G	CYTB	Non-synonymous	N8S	0.99	0	0.102
Ancient terminal	R0	C	14770	G	CYTB	Non-synonymous	N8K	0.995	0	0.151
Modern terminal	U	C	14770	A	CYTB	Non-synonymous	N8K	0.995	0	0.151
Modern terminal	R0	C	14771	A	CYTB	Non-synonymous	P9T	0.996	0	0.133
Modern terminal	R0	C	14771	A	CYTB	Non-synonymous	P9T	0.996	0	0.133
Ancient terminal	U	C	14771	T	CYTB	Non-synonymous	P9S	0.999	0	0.142
Modern terminal	R0	T	14775	C	CYTB	Non-synonymous	L10P	1	0	0.432
Ancient terminal	R0	T	14787	C	CYTB	Non-synonymous	I14T	0.949	0	0.112
Ancient terminal	R0	T	14787	G	CYTB	Non-synonymous	I14S	0.984	0	0.22
Pre-terminal	JT	A	14789	T	CYTB	Non-synonymous	N15Y	0.977	0	0.375
Modern terminal	R0	A	14790	G	CYTB	Non-synonymous	N15S	0.003	0	0.138
Modern terminal	R0	A	14790	G	CYTB	Non-synonymous	N15S	0.003	0	0.138
Pre-terminal	R0	A	14790	G	CYTB	Non-synonymous	N15S	0.003	0	0.138
Modern terminal	U	A	14790	G	CYTB	Non-synonymous	N15S	0.003	0	0.138
Modern terminal	JT	A	14790	G	CYTB	Non-synonymous	N15S	0.003	0	0.138
Modern terminal	U	C	14792	G	CYTB	Non-synonymous	H16D	0.812	0.12	0.294
Modern terminal	JT	C	14792	T	CYTB	Non-synonymous	H16Y	0.212	0.01	0.261
Pre-terminal	R0	A	14793	G	CYTB	Non-synonymous	H16R	0	0.01	0.224
Ancient terminal	U	A	14793	G	CYTB	Non-synonymous	H16R	0	0.01	0.224
Modern terminal	U	A	14793	G	CYTB	Non-synonymous	H16R	0	0.01	0.224
Modern terminal	U	A	14793	G	CYTB	Non-synonymous	H16R	0	0.01	0.224
Modern terminal	U	A	14793	G	CYTB	Non-synonymous	H16R	0	0.01	0.224
Pre-terminal	U	A	14793	G	CYTB	Non-synonymous	H16R	0	0.01	0.224
Ancient terminal	JT	A	14793	G	CYTB	Non-synonymous	H16R	0	0.01	0.224
Modern terminal	JT	A	14793	G	CYTB	Non-synonymous	H16R	0	0.01	0.224
Modern terminal	JT	A	14793	G	CYTB	Non-synonymous	H16R	0	0.01	0.224
Pre-terminal	JT	A	14793	G	CYTB	Non-synonymous	H16R	0	0.01	0.224
Ancient terminal	N1	A	14793	G	CYTB	Non-synonymous	H16R	0	0.01	0.224
Modern terminal	JT	C	14794	A	CYTB	Non-synonymous	H16Q	0.585	0.03	0.204
Modern terminal	N1	C	14794	A	CYTB	Non-synonymous	H16Q	0.585	0.03	0.204
Modern terminal	R0	T	14798	C	CYTB	Non-synonymous	F18L	0	0.2	0.127
Modern terminal	R0	T	14798	C	CYTB	Non-synonymous	F18L	0	0.2	0.127
Modern terminal	R0	T	14798	C	CYTB	Non-synonymous	F18L	0	0.2	0.127
Modern terminal	R0	T	14798	C	CYTB	Non-synonymous	F18L	0	0.2	0.127
Modern terminal	R0	T	14798	C	CYTB	Non-synonymous	F18L	0	0.2	0.127
Modern terminal	R0	T	14798	C	CYTB	Non-synonymous	F18L	0	0.2	0.127
Modern terminal	R0	T	14798	C	CYTB	Non-synonymous	F18L	0	0.2	0.127
Modern terminal	R0	T	14798	C	CYTB	Non-synonymous	F18L	0	0.2	0.127
Modern terminal	R0	T	14798	C	CYTB	Non-synonymous	F18L	0	0.2	0.127
Pre-terminal	R0	T	14798	C	CYTB	Non-synonymous	F18L	0	0.2	0.127
Pre-terminal	R0	T	14798	C	CYTB	Non-synonymous	F18L	0	0.2	0.127

Pre-terminal	R0	T	14798	C	CYTB	Non-synonymous	F18L	0	0.2	0.127
Modern terminal	U	T	14798	C	CYTB	Non-synonymous	F18L	0	0.2	0.127
Modern terminal	U	T	14798	C	CYTB	Non-synonymous	F18L	0	0.2	0.127
Modern terminal	U	T	14798	C	CYTB	Non-synonymous	F18L	0	0.2	0.127
Pre-terminal	U	T	14798	C	CYTB	Non-synonymous	F18L	0	0.2	0.127
Modern terminal	JT	T	14798	C	CYTB	Non-synonymous	F18L	0	0.2	0.127
Modern terminal	JT	T	14798	C	CYTB	Non-synonymous	F18L	0	0.2	0.127
Modern terminal	JT	T	14798	C	CYTB	Non-synonymous	F18L	0	0.2	0.127
Modern terminal	JT	T	14798	C	CYTB	Non-synonymous	F18L	0	0.2	0.127
Pre-terminal	JT	T	14798	C	CYTB	Non-synonymous	F18L	0	0.2	0.127
Modern terminal	N1	T	14798	C	CYTB	Non-synonymous	F18L	0	0.2	0.127
Pre-terminal	N1	T	14798	C	CYTB	Non-synonymous	F18L	0	0.2	0.127
Pre-terminal	X	T	14798	C	CYTB	Non-synonymous	F18L	0	0.2	0.127
Modern terminal	N2	T	14798	C	CYTB	Non-synonymous	F18L	0	0.2	0.127
Pre-terminal	R0	C	14800	A	CYTB	Non-synonymous	F18L	0	0.2	0.127
Modern terminal	U	C	14800	A	CYTB	Non-synonymous	F18L	0	0.2	0.127
Modern terminal	N2	G	14804	A	CYTB	Non-synonymous	D20N	0.992	0	0.131
Modern terminal	U	C	14814	A	CYTB	Non-synonymous	T23N	0.58	0	0.189
Ancient terminal	N2	C	14816	A	CYTB	Non-synonymous	P24T	0.999	0	0.326
Pre-terminal	U	T	14819	C	CYTB	Non-synonymous	S25P	0.001	0.02	0.295
Pre-terminal	U	T	14819	C	CYTB	Non-synonymous	S25P	0.001	0.02	0.295
Modern terminal	R0	A	14825	G	CYTB	Non-synonymous	I27V	0.889	0.01	0.084
Modern terminal	R0	T	14826	C	CYTB	Non-synonymous	I27T	1	0	0.173
Ancient terminal	U	T	14826	C	CYTB	Non-synonymous	I27T	1	0	0.173
Modern terminal	U	T	14826	C	CYTB	Non-synonymous	I27T	1	0	0.173
Ancient terminal	R0	C	14829	A	CYTB	Non-synonymous	S28Y	0.999	0	0.328
Modern terminal	R0	G	14831	A	CYTB	Non-synonymous	A29T	0	0.1	0.088
Modern terminal	R0	G	14831	A	CYTB	Non-synonymous	A29T	0	0.1	0.088
Modern terminal	R0	G	14831	A	CYTB	Non-synonymous	A29T	0	0.1	0.088
Modern terminal	R0	G	14831	A	CYTB	Non-synonymous	A29T	0	0.1	0.088
Modern terminal	R0	G	14831	A	CYTB	Non-synonymous	A29T	0	0.1	0.088
Modern terminal	R0	G	14831	A	CYTB	Non-synonymous	A29T	0	0.1	0.088
Modern terminal	R0	G	14831	A	CYTB	Non-synonymous	A29T	0	0.1	0.088
Modern terminal	R0	G	14831	A	CYTB	Non-synonymous	A29T	0	0.1	0.088
Modern terminal	R0	G	14831	A	CYTB	Non-synonymous	A29T	0	0.1	0.088
Modern terminal	R0	G	14831	A	CYTB	Non-synonymous	A29T	0	0.1	0.088
Modern terminal	R0	G	14831	A	CYTB	Non-synonymous	A29T	0	0.1	0.088
Pre-terminal	R0	G	14831	A	CYTB	Non-synonymous	A29T	0	0.1	0.088
Pre-terminal	R0	G	14831	A	CYTB	Non-synonymous	A29T	0	0.1	0.088
Pre-terminal	R0	G	14831	A	CYTB	Non-synonymous	A29T	0	0.1	0.088
Pre-terminal	R0	G	14831	A	CYTB	Non-synonymous	A29T	0	0.1	0.088
Pre-terminal	R0	G	14831	A	CYTB	Non-synonymous	A29T	0	0.1	0.088
Modern terminal	U	G	14831	A	CYTB	Non-synonymous	A29T	0	0.1	0.088
Modern terminal	U	G	14831	A	CYTB	Non-synonymous	A29T	0	0.1	0.088
Modern terminal	U	G	14831	A	CYTB	Non-synonymous	A29T	0	0.1	0.088
Modern terminal	U	G	14831	A	CYTB	Non-synonymous	A29T	0	0.1	0.088
Modern terminal	U	G	14831	A	CYTB	Non-synonymous	A29T	0	0.1	0.088
Modern terminal	U	G	14831	A	CYTB	Non-synonymous	A29T	0	0.1	0.088
Pre-terminal	U	G	14831	A	CYTB	Non-synonymous	A29T	0	0.1	0.088
Pre-terminal	U	G	14831	A	CYTB	Non-synonymous	A29T	0	0.1	0.088

Pre-terminal	U	G	14831	A	CYTB	Non-synonymous	A29T	0	0.1	0.088
Modern terminal	JT	G	14831	A	CYTB	Non-synonymous	A29T	0	0.1	0.088
Modern terminal	JT	G	14831	A	CYTB	Non-synonymous	A29T	0	0.1	0.088
Pre-terminal	JT	G	14831	A	CYTB	Non-synonymous	A29T	0	0.1	0.088
Pre-terminal	N1	G	14831	A	CYTB	Non-synonymous	A29T	0	0.1	0.088
Ancient terminal	R0	G	14835	T	CYTB	Non-synonymous	W30L	1	0	0.691
Ancient terminal	R0	A	14841	G	CYTB	Non-synonymous	N32S	0.998	0	0.141
Ancient terminal	U	A	14841	G	CYTB	Non-synonymous	N32S	0.998	0	0.141
Ancient terminal	U	C	14852	T	CYTB	Non-synonymous	L36F	0.999	0	0.299
Modern terminal	U	T	14856	C	CYTB	Non-synonymous	L37P	1	0	0.802
Modern terminal	R0	G	14858	A	CYTB	Non-synonymous	G38S	1	0	0.345
Modern terminal	R0	G	14858	A	CYTB	Non-synonymous	G38S	1	0	0.345
Modern terminal	JT	G	14858	A	CYTB	Non-synonymous	G38S	1	0	0.345
Modern terminal	R0	G	14861	A	CYTB	Non-synonymous	A39T	0.002	0.09	0.288
Modern terminal	R0	G	14861	A	CYTB	Non-synonymous	A39T	0.002	0.09	0.288
Modern terminal	R0	G	14861	A	CYTB	Non-synonymous	A39T	0.002	0.09	0.288
Modern terminal	R0	G	14861	A	CYTB	Non-synonymous	A39T	0.002	0.09	0.288
Pre-terminal	R0	G	14861	A	CYTB	Non-synonymous	A39T	0.002	0.09	0.288
Pre-terminal	R0	G	14861	A	CYTB	Non-synonymous	A39T	0.002	0.09	0.288
Pre-terminal	R0	G	14861	A	CYTB	Non-synonymous	A39T	0.002	0.09	0.288
Ancient terminal	U	G	14861	A	CYTB	Non-synonymous	A39T	0.002	0.09	0.288
Modern terminal	U	G	14861	A	CYTB	Non-synonymous	A39T	0.002	0.09	0.288
Modern terminal	U	G	14861	A	CYTB	Non-synonymous	A39T	0.002	0.09	0.288
Pre-terminal	U	G	14861	A	CYTB	Non-synonymous	A39T	0.002	0.09	0.288
Modern terminal	JT	G	14861	A	CYTB	Non-synonymous	A39T	0.002	0.09	0.288
Modern terminal	JT	G	14861	A	CYTB	Non-synonymous	A39T	0.002	0.09	0.288
Pre-terminal	JT	G	14861	A	CYTB	Non-synonymous	A39T	0.002	0.09	0.288
Modern terminal	X	G	14861	A	CYTB	Non-synonymous	A39T	0.002	0.09	0.288
Pre-terminal	X	G	14861	A	CYTB	Non-synonymous	A39T	0.002	0.09	0.288
Modern terminal	N2	G	14861	A	CYTB	Non-synonymous	A39T	0.002	0.09	0.288
Modern terminal	U	C	14867	A	CYTB	Non-synonymous	L41M	1	0	0.15
Modern terminal	U	T	14868	A	CYTB	Non-synonymous	L41Q	1	0	0.496
Modern terminal	R0	A	14870	G	CYTB	Non-synonymous	I42V	0.001	0.16	0.078
Pre-terminal	R0	A	14870	G	CYTB	Non-synonymous	I42V	0.001	0.16	0.078
Modern terminal	U	A	14870	G	CYTB	Non-synonymous	I42V	0.001	0.16	0.078
Pre-terminal	U	A	14870	G	CYTB	Non-synonymous	I42V	0.001	0.16	0.078
Modern terminal	R0	T	14871	C	CYTB	Non-synonymous	I42T	0.027	0.03	0.159
Modern terminal	R0	T	14871	C	CYTB	Non-synonymous	I42T	0.027	0.03	0.159
Pre-terminal	R0	T	14871	C	CYTB	Non-synonymous	I42T	0.027	0.03	0.159
Modern terminal	U	T	14871	C	CYTB	Non-synonymous	I42T	0.027	0.03	0.159
Modern terminal	JT	A	14879	T	CYTB	Non-synonymous	I45F	0.705	0	0.216
Modern terminal	U	A	14882	C	CYTB	Non-synonymous	T46P	0.888	0	0.195
Pre-terminal	U	C	14883	T	CYTB	Non-synonymous	T46I	0	0.18	0.113
Ancient terminal	JT	C	14883	T	CYTB	Non-synonymous	T46I	0	0.18	0.113
Modern terminal	R0	T	14894	C	CYTB	Non-synonymous	F50L	0.999	0.01	0.12
Ancient terminal	U	T	14894	C	CYTB	Non-synonymous	F50L	0.999	0.01	0.12
Modern terminal	U	T	14894	C	CYTB	Non-synonymous	F50L	0.999	0.01	0.12
Modern terminal	JT	T	14894	C	CYTB	Non-synonymous	F50L	0.999	0.01	0.12
Modern terminal	JT	T	14894	C	CYTB	Non-synonymous	F50L	0.999	0.01	0.12
Modern terminal	R0	G	14900	A	CYTB	Non-synonymous	A52T	0.998	0	0.167

Pre-terminal	R0	G	14900	A	CYTB	Non-synonymous	A52T	0.998	0	0.167
Modern terminal	R0	C	14906	G	CYTB	Non-synonymous	H54D	0.998	0	0.297
Modern terminal	R0	T	14912	G	CYTB	Non-synonymous	S56A	0	0.01	0.145
Modern terminal	R0	T	14912	G	CYTB	Non-synonymous	S56A	0	0.01	0.145
Modern terminal	U	G	14921	A	CYTB	Non-synonymous	A59T	0.994	1	0.129
Pre-terminal	U	G	14921	A	CYTB	Non-synonymous	A59T	0.994	1	0.129
Modern terminal	R0	C	14922	T	CYTB	Non-synonymous	A59V	0.997	0	0.121
Modern terminal	X	T	14924	C	CYTB	Non-synonymous	S60P	0.451	0.05	0.329
Modern terminal	R0	A	14927	G	CYTB	Non-synonymous	T61A	0.002	0	0.189
Modern terminal	R0	A	14927	G	CYTB	Non-synonymous	T61A	0.002	0	0.189
Modern terminal	R0	A	14927	G	CYTB	Non-synonymous	T61A	0.002	0	0.189
Modern terminal	R0	A	14927	G	CYTB	Non-synonymous	T61A	0.002	0	0.189
Pre-terminal	R0	A	14927	G	CYTB	Non-synonymous	T61A	0.002	0	0.189
Modern terminal	U	A	14927	G	CYTB	Non-synonymous	T61A	0.002	0	0.189
Modern terminal	U	A	14927	G	CYTB	Non-synonymous	T61A	0.002	0	0.189
Modern terminal	U	A	14927	G	CYTB	Non-synonymous	T61A	0.002	0	0.189
Pre-terminal	U	A	14927	G	CYTB	Non-synonymous	T61A	0.002	0	0.189
Modern terminal	JT	A	14927	G	CYTB	Non-synonymous	T61A	0.002	0	0.189
Modern terminal	JT	A	14927	G	CYTB	Non-synonymous	T61A	0.002	0	0.189
Pre-terminal	JT	A	14927	G	CYTB	Non-synonymous	T61A	0.002	0	0.189
Pre-terminal	N1	A	14927	G	CYTB	Non-synonymous	T61A	0.002	0	0.189
Modern terminal	R0	G	14945	A	CYTB	Non-synonymous	A67T	0.994	1	0.311
Modern terminal	R0	G	14945	A	CYTB	Non-synonymous	A67T	0.994	1	0.311
Modern terminal	U	G	14945	A	CYTB	Non-synonymous	A67T	0.994	1	0.311
Modern terminal	U	G	14945	A	CYTB	Non-synonymous	A67T	0.994	1	0.311
Modern terminal	U	G	14945	A	CYTB	Non-synonymous	A67T	0.994	1	0.311
Modern terminal	U	C	14950	G	CYTB	Non-synonymous	H68Q	0.996	0	0.206
Pre-terminal	JT	T	14952	C	CYTB	Non-synonymous	I69T	1	0	0.285
Modern terminal	R0	A	14954	G	CYTB	Non-synonymous	T70A	0.982	0	0.26
Modern terminal	R0	A	14954	G	CYTB	Non-synonymous	T70A	0.982	0	0.26
Modern terminal	JT	A	14954	G	CYTB	Non-synonymous	T70A	0.982	0	0.26
Modern terminal	JT	A	14954	G	CYTB	Non-synonymous	T70A	0.982	0	0.26
Ancient terminal	N1	A	14954	G	CYTB	Non-synonymous	T70A	0.982	0	0.26
Modern terminal	JT	G	14960	A	CYTB	Non-synonymous	D72N	0.997	0	0.305
Modern terminal	R0	G	14963	A	CYTB	Non-synonymous	V73M	0.997	0	0.246
Pre-terminal	U	A	14966	G	CYTB	Non-synonymous	N74D	0.997	0	0.273
Modern terminal	R0	A	14970	G	CYTB	Non-synonymous	Y75C	1	0	0.478
Modern terminal	R0	A	14978	G	CYTB	Non-synonymous	I78V	0	0.13	0.112
Modern terminal	R0	A	14978	G	CYTB	Non-synonymous	I78V	0	0.13	0.112
Modern terminal	R0	A	14978	G	CYTB	Non-synonymous	I78V	0	0.13	0.112
Pre-terminal	R0	A	14978	G	CYTB	Non-synonymous	I78V	0	0.13	0.112
Ancient terminal	R0	T	14979	C	CYTB	Non-synonymous	I78T	0.001	0.02	0.163
Modern terminal	R0	T	14979	G	CYTB	Non-synonymous	I78S	0.38	0	0.363
Modern terminal	R0	T	14979	C	CYTB	Non-synonymous	I78T	0.001	0.02	0.163
Pre-terminal	R0	T	14979	C	CYTB	Non-synonymous	I78T	0.001	0.02	0.163
Modern terminal	U	T	14979	C	CYTB	Non-synonymous	I78T	0.001	0.02	0.163
Pre-terminal	N1	T	14979	C	CYTB	Non-synonymous	I78T	0.001	0.02	0.163
Modern terminal	R0	C	14980	A	CYTB	Non-synonymous	I78M	0.005	0.14	0.187
Modern terminal	U	C	14980	A	CYTB	Non-synonymous	I78M	0.005	0.14	0.187
Modern terminal	JT	C	14980	A	CYTB	Non-synonymous	I78M	0.005	0.14	0.187

Modern terminal	R0	A	14981	G	CYTB	Non-synonymous	I79V	0.002	0	0.147
Modern terminal	R0	A	14981	G	CYTB	Non-synonymous	I79V	0.002	0	0.147
Pre-terminal	N2	A	14981	C	CYTB	Non-synonymous	I79V	0.002	0	0.147
Pre-terminal	R0	T	14982	C	CYTB	Non-synonymous	I79T	0.982	0	0.329
Pre-terminal	JT	C	14983	A	CYTB	Non-synonymous	I79M	0.987	0	0.25
Modern terminal	R0	C	14990	T	CYTB	Non-synonymous	L82F	0.998	0	0.561
Ancient terminal	U	A	14994	C	CYTB	Non-synonymous	H83P	1	0	0.736
Modern terminal	R0	G	14996	A	CYTB	Non-synonymous	A84T	0.005	0.01	0.479
Modern terminal	U	G	14996	A	CYTB	Non-synonymous	A84T	0.005	0.01	0.479
Pre-terminal	U	G	14996	A	CYTB	Non-synonymous	A84T	0.005	0.01	0.479
Modern terminal	JT	G	14996	A	CYTB	Non-synonymous	A84T	0.005	0.01	0.479
Modern terminal	U	A	14999	G	CYTB	Non-synonymous	N85D	0.998	0	0.385
Ancient terminal	R0	G	15002	A	CYTB	Non-synonymous	G86S	0.098	0	0.507
Modern terminal	N1	G	15002	A	CYTB	Non-synonymous	G86S	0.098	0	0.507
Modern terminal	U	G	15003	C	CYTB	Non-synonymous	G86A	0.627	0	0.565
Ancient terminal	JT	T	15008	A	CYTB	Non-synonymous	S88T	0.943	0	0.318
Pre-terminal	R0	T	15014	C	CYTB	Non-synonymous	F90L	0.048	0.01	0.506
Modern terminal	U	T	15014	C	CYTB	Non-synonymous	F90L	0.048	0.01	0.506
Modern terminal	U	T	15014	C	CYTB	Non-synonymous	F90L	0.048	0.01	0.506
Pre-terminal	JT	T	15014	C	CYTB	Non-synonymous	F90L	0.048	0.01	0.506
Modern terminal	U	C	15016	A	CYTB	Non-synonymous	F90L	0.048	0.01	0.506
Modern terminal	R0	G	15024	A	CYTB	Non-synonymous	C93Y	1	0	0.63
Modern terminal	U	G	15024	A	CYTB	Non-synonymous	C93Y	1	0	0.63
Ancient terminal	JT	T	15030	A	CYTB	Non-synonymous	F95Y	0.819	0.47	0.419
Modern terminal	U	A	15038	G	CYTB	Non-synonymous	I98V	0.856	1	0.07
Modern terminal	X	T	15039	C	CYTB	Non-synonymous	I98T	1	0.01	0.287
Modern terminal	JT	C	15040	A	CYTB	Non-synonymous	I98M	1	0	0.232
Ancient terminal	R0	G	15041	T	CYTB	Non-synonymous	G99W	1	0	0.599
Modern terminal	N1	G	15045	A	CYTB	Non-synonymous	R100Q	1	0	0.224
Modern terminal	R0	G	15047	A	CYTB	Non-synonymous	G101S	0.052	0.01	0.341
Modern terminal	R0	G	15047	A	CYTB	Non-synonymous	G101S	0.052	0.01	0.341
Pre-terminal	R0	G	15047	A	CYTB	Non-synonymous	G101S	0.052	0.01	0.341
Pre-terminal	R0	G	15047	A	CYTB	Non-synonymous	G101S	0.052	0.01	0.341
Pre-terminal	R0	G	15047	A	CYTB	Non-synonymous	G101S	0.052	0.01	0.341
Pre-terminal	R0	G	15047	A	CYTB	Non-synonymous	G101S	0.052	0.01	0.341
Modern terminal	U	G	15047	A	CYTB	Non-synonymous	G101S	0.052	0.01	0.341
Modern terminal	U	G	15047	A	CYTB	Non-synonymous	G101S	0.052	0.01	0.341
Modern terminal	U	G	15047	A	CYTB	Non-synonymous	G101S	0.052	0.01	0.341
Pre-terminal	U	G	15047	A	CYTB	Non-synonymous	G101S	0.052	0.01	0.341
Modern terminal	JT	G	15047	A	CYTB	Non-synonymous	G101S	0.052	0.01	0.341
Modern terminal	JT	G	15047	A	CYTB	Non-synonymous	G101S	0.052	0.01	0.341
Modern terminal	R0	G	15048	A	CYTB	Non-synonymous	G101D	0.992	0	0.536
Modern terminal	R0	T	15051	C	CYTB	Non-synonymous	L102P	1	0	0.771
Pre-terminal	U	C	15068	T	CYTB	Non-synonymous	L108F	0.839	0.04	0.459
Modern terminal	R0	T	15074	C	CYTB	Non-synonymous	S110P	0	0.13	0.43
Modern terminal	U	T	15074	C	CYTB	Non-synonymous	S110P	0	0.13	0.43
Pre-terminal	N1	T	15074	C	CYTB	Non-synonymous	S110P	0	0.13	0.43
Modern terminal	R0	G	15077	A	CYTB	Non-synonymous	E111K	0.997	0.03	0.331
Modern terminal	R0	G	15077	A	CYTB	Non-synonymous	E111K	0.997	0.03	0.331
Modern terminal	R0	G	15077	A	CYTB	Non-synonymous	E111K	0.997	0.03	0.331

Pre-terminal	R0	G	15077	A	CYTB	Non-synonymous	E111K	0.997	0.03	0.331
Pre-terminal	R0	G	15077	A	CYTB	Non-synonymous	E111K	0.997	0.03	0.331
Modern terminal	U	G	15077	A	CYTB	Non-synonymous	E111K	0.997	0.03	0.331
Modern terminal	U	G	15077	A	CYTB	Non-synonymous	E111K	0.997	0.03	0.331
Modern terminal	U	G	15077	T	CYTB	Nonsense				
Pre-terminal	U	G	15077	A	CYTB	Non-synonymous	E111K	0.997	0.03	0.331
Pre-terminal	U	G	15077	A	CYTB	Non-synonymous	E111K	0.997	0.03	0.331
Pre-terminal	U	G	15077	A	CYTB	Non-synonymous	E111K	0.997	0.03	0.331
Modern terminal	JT	G	15077	A	CYTB	Non-synonymous	E111K	0.997	0.03	0.331
Modern terminal	JT	G	15077	A	CYTB	Non-synonymous	E111K	0.997	0.03	0.331
Pre-terminal	JT	G	15077	A	CYTB	Non-synonymous	E111K	0.997	0.03	0.331
Modern terminal	X	G	15077	A	CYTB	Non-synonymous	E111K	0.997	0.03	0.331
Modern terminal	X	G	15077	A	CYTB	Non-synonymous	E111K	0.997	0.03	0.331
Modern terminal	X	G	15077	A	CYTB	Non-synonymous	E111K	0.997	0.03	0.331
Pre-terminal	X	G	15077	A	CYTB	Non-synonymous	E111K	0.997	0.03	0.331
Pre-terminal	JT	A	15078	G	CYTB	Non-synonymous	E111G	1	0	0.39
Pre-terminal	R0	A	15080	G	CYTB	Non-synonymous	T112A	0.997	0	0.254
Pre-terminal	R0	A	15087	T	CYTB	Non-synonymous	N114I	1	0	0.34
Modern terminal	U	A	15087	T	CYTB	Non-synonymous	N114I	1	0	0.34
Pre-terminal	R0	T	15090	C	CYTB	Non-synonymous	I115T	0.991	0	0.247
Pre-terminal	JT	T	15090	C	CYTB	Non-synonymous	I115T	0.991	0	0.247
Ancient terminal	U	G	15092	T	CYTB	Non-synonymous	G116C	1	0	0.633
Modern terminal	JT	A	15095	G	CYTB	Non-synonymous	I117V	0.001	0.67	0.105
Modern terminal	R0	A	15098	G	CYTB	Non-synonymous	I118V	0.002	0.09	0.111
Pre-terminal	JT	A	15098	G	CYTB	Non-synonymous	I118V	0.002	0.09	0.111
Modern terminal	R0	T	15099	C	CYTB	Non-synonymous	I118T	0.976	0	0.272
Modern terminal	R0	C	15107	A	CYTB	Non-synonymous	L121I	0.138	0.01	0.393
Modern terminal	R0	G	15110	A	CYTB	Non-synonymous	A122T	0	0.54	0.176
Modern terminal	R0	G	15110	A	CYTB	Non-synonymous	A122T	0	0.54	0.176
Modern terminal	R0	G	15110	A	CYTB	Non-synonymous	A122T	0	0.54	0.176
Modern terminal	R0	G	15110	A	CYTB	Non-synonymous	A122T	0	0.54	0.176
Modern terminal	R0	G	15110	A	CYTB	Non-synonymous	A122T	0	0.54	0.176
Modern terminal	R0	G	15110	A	CYTB	Non-synonymous	A122T	0	0.54	0.176
Pre-terminal	R0	G	15110	A	CYTB	Non-synonymous	A122T	0	0.54	0.176
Pre-terminal	R0	G	15110	A	CYTB	Non-synonymous	A122T	0	0.54	0.176
Pre-terminal	R0	G	15110	A	CYTB	Non-synonymous	A122T	0	0.54	0.176
Pre-terminal	R0	G	15110	A	CYTB	Non-synonymous	A122T	0	0.54	0.176
Modern terminal	U	G	15110	A	CYTB	Non-synonymous	A122T	0	0.54	0.176
Pre-terminal	U	G	15110	A	CYTB	Non-synonymous	A122T	0	0.54	0.176
Modern terminal	JT	G	15110	A	CYTB	Non-synonymous	A122T	0	0.54	0.176
Modern terminal	JT	G	15110	A	CYTB	Non-synonymous	A122T	0	0.54	0.176
Pre-terminal	JT	G	15110	A	CYTB	Non-synonymous	A122T	0	0.54	0.176
Pre-terminal	JT	G	15110	A	CYTB	Non-synonymous	A122T	0	0.54	0.176
Pre-terminal	JT	G	15110	A	CYTB	Non-synonymous	A122T	0	0.54	0.176
Pre-terminal	JT	G	15110	A	CYTB	Non-synonymous	A122T	0	0.54	0.176
Modern terminal	N1	G	15110	A	CYTB	Non-synonymous	A122T	0	0.54	0.176
Modern terminal	N2	G	15110	A	CYTB	Non-synonymous	A122T	0	0.54	0.176
Modern terminal	R0	C	15111	T	CYTB	Non-synonymous	A122V	0.047	0.06	0.391
Modern terminal	R0	A	15113	G	CYTB	Non-synonymous	T123A	0.981	0	0.54
Modern terminal	U	A	15113	G	CYTB	Non-synonymous	T123A	0.981	0	0.54

Pre-terminal	U	A	15113	G	CYTB	Non-synonymous	T123A	0.981	0	0.54
Pre-terminal	JT	A	15113	G	CYTB	Non-synonymous	T123A	0.981	0	0.54
Ancient terminal	R0	T	15117	C	CYTB	Non-synonymous	M124T	0.997	0	0.458
Modern terminal	JT	T	15117	C	CYTB	Non-synonymous	M124T	0.997	0	0.458
Ancient terminal	U	G	15119	A	CYTB	Non-synonymous	A125T	0.002	0	0.492
Modern terminal	U	G	15119	A	CYTB	Non-synonymous	A125T	0.002	0	0.492
Modern terminal	U	G	15119	A	CYTB	Non-synonymous	A125T	0.002	0	0.492
Pre-terminal	U	G	15119	A	CYTB	Non-synonymous	A125T	0.002	0	0.492
Modern terminal	JT	G	15119	A	CYTB	Non-synonymous	A125T	0.002	0	0.492
Modern terminal	JT	G	15119	A	CYTB	Non-synonymous	A125T	0.002	0	0.492
Modern terminal	JT	G	15119	A	CYTB	Non-synonymous	A125T	0.002	0	0.492
Modern terminal	JT	G	15119	A	CYTB	Non-synonymous	A125T	0.002	0	0.492
Modern terminal	JT	G	15119	A	CYTB	Non-synonymous	A125T	0.002	0	0.492
Modern terminal	JT	G	15119	A	CYTB	Non-synonymous	A125T	0.002	0	0.492
Pre-terminal	JT	G	15119	A	CYTB	Non-synonymous	A125T	0.002	0	0.492
Modern terminal	N1	G	15119	A	CYTB	Non-synonymous	A125T	0.002	0	0.492
Modern terminal	N1	G	15119	A	CYTB	Non-synonymous	A125T	0.002	0	0.492
Pre-terminal	X	G	15119	A	CYTB	Non-synonymous	A125T	0.002	0	0.492
Modern terminal	N2	G	15119	A	CYTB	Non-synonymous	A125T	0.002	0	0.492
Ancient terminal	R0	A	15122	G	CYTB	Non-synonymous	T126A	0.001	0	0.449
Pre-terminal	U	A	15122	G	CYTB	Non-synonymous	T126A	0.001	0	0.449
Modern terminal	U	G	15125	A	CYTB	Non-synonymous	A127T	0.999	0	0.461
Modern terminal	R0	A	15131	C	CYTB	Non-synonymous	M129L	0.52	0	0.432
Modern terminal	U	A	15131	C	CYTB	Non-synonymous	M129L	0.52	0	0.432
Modern terminal	R0	T	15132	C	CYTB	Non-synonymous	M129T	0.997	0	0.415
Modern terminal	R0	T	15132	C	CYTB	Non-synonymous	M129T	0.997	0	0.415
Modern terminal	U	T	15132	C	CYTB	Non-synonymous	M129T	0.997	0	0.415
Modern terminal	R0	G	15140	A	CYTB	Non-synonymous	V132I	0.878	0.01	0.215
Modern terminal	R0	G	15153	A	CYTB	Non-synonymous	G136D	1	0	0.519
Modern terminal	JT	G	15153	A	CYTB	Non-synonymous	G136D	1	0	0.519
Pre-terminal	N1	G	15153	A	CYTB	Non-synonymous	G136D	1	0	0.519
Pre-terminal	JT	T	15159	C	CYTB	Non-synonymous	M138T	0.997	0	0.384
Modern terminal	R0	T	15164	C	CYTB	Non-synonymous	F140L	0.048	0.01	0.401
Pre-terminal	R0	T	15164	C	CYTB	Non-synonymous	F140L	0.048	0.01	0.401
Ancient terminal	R0	G	15170	C	CYTB	Non-synonymous	G142R	1	0	0.462
Modern terminal	R0	G	15173	A	CYTB	Non-synonymous	A143T	0.999	0	0.344
Pre-terminal	U	A	15176	G	CYTB	Non-synonymous	T144A	0.997	0	0.271
Ancient terminal	R0	T	15183	A	CYTB	Non-synonymous	I146N	1	0	0.512
Ancient terminal	R0	A	15189	C	CYTB	Non-synonymous	N148T	0.994	0	0.275
Modern terminal	R0	T	15204	C	CYTB	Non-synonymous	I153T	0.903	0.01	0.238
Modern terminal	R0	T	15204	C	CYTB	Non-synonymous	I153T	0.903	0.01	0.238
Modern terminal	R0	T	15204	C	CYTB	Non-synonymous	I153T	0.903	0.01	0.238
Modern terminal	R0	T	15204	C	CYTB	Non-synonymous	I153T	0.903	0.01	0.238
Modern terminal	R0	T	15204	C	CYTB	Non-synonymous	I153T	0.903	0.01	0.238
Pre-terminal	R0	T	15204	C	CYTB	Non-synonymous	I153T	0.903	0.01	0.238
Modern terminal	U	T	15204	C	CYTB	Non-synonymous	I153T	0.903	0.01	0.238
Modern terminal	U	T	15204	C	CYTB	Non-synonymous	I153T	0.903	0.01	0.238
Ancient terminal	JT	T	15204	C	CYTB	Non-synonymous	I153T	0.903	0.01	0.238
Modern terminal	N1	T	15204	C	CYTB	Non-synonymous	I153T	0.903	0.01	0.238
Ancient terminal	U	C	15205	A	CYTB	Non-synonymous	I153M	0.929	0	0.216

Ancient terminal	N2	C	15206	A	CYTB	Non-synonymous	P154T	0.998	0	0.288
Ancient terminal	R0	C	15207	A	CYTB	Non-synonymous	P154Q	0.999	0	0.351
Modern terminal	R0	T	15209	C	CYTB	Non-synonymous	Y155H	1	0	0.402
Modern terminal	R0	T	15209	C	CYTB	Non-synonymous	Y155H	1	0	0.402
Pre-terminal	R0	T	15209	C	CYTB	Non-synonymous	Y155H	1	0	0.402
Modern terminal	JT	T	15209	C	CYTB	Non-synonymous	Y155H	1	0	0.402
Modern terminal	JT	T	15209	C	CYTB	Non-synonymous	Y155H	1	0	0.402
Ancient terminal	N1	T	15209	A	CYTB	Non-synonymous	Y155N	1	0	0.622
Modern terminal	U	A	15212	G	CYTB	Non-synonymous	I156V	0.78	0.07	0.078
Pre-terminal	U	A	15212	G	CYTB	Non-synonymous	I156V	0.78	0.07	0.078
Modern terminal	JT	A	15212	G	CYTB	Non-synonymous	I156V	0.78	0.07	0.078
Pre-terminal	JT	A	15212	G	CYTB	Non-synonymous	I156V	0.78	0.07	0.078
Modern terminal	R0	T	15213	C	CYTB	Non-synonymous	I156T	0.999	0.01	0.212
Modern terminal	R0	T	15213	C	CYTB	Non-synonymous	I156T	0.999	0.01	0.212
Modern terminal	R0	T	15213	C	CYTB	Non-synonymous	I156T	0.999	0.01	0.212
Modern terminal	R0	T	15213	C	CYTB	Non-synonymous	I156T	0.999	0.01	0.212
Pre-terminal	JT	T	15213	C	CYTB	Non-synonymous	I156T	0.999	0.01	0.212
Pre-terminal	JT	T	15213	C	CYTB	Non-synonymous	I156T	0.999	0.01	0.212
Ancient terminal	N1	T	15213	C	CYTB	Non-synonymous	I156T	0.999	0.01	0.212
Modern terminal	R0	A	15218	G	CYTB	Non-synonymous	T158A	0.986	0.02	0.169
Pre-terminal	R0	A	15218	C	CYTB	Non-synonymous	T158P	1	0.03	0.285
Modern terminal	U	A	15218	G	CYTB	Non-synonymous	T158A	0.986	0.02	0.169
Modern terminal	U	A	15218	G	CYTB	Non-synonymous	T158A	0.986	0.02	0.169
Modern terminal	U	A	15218	G	CYTB	Non-synonymous	T158A	0.986	0.02	0.169
Modern terminal	U	A	15218	G	CYTB	Non-synonymous	T158A	0.986	0.02	0.169
Modern terminal	U	A	15218	G	CYTB	Non-synonymous	T158A	0.986	0.02	0.169
Modern terminal	U	A	15218	G	CYTB	Non-synonymous	T158A	0.986	0.02	0.169
Modern terminal	U	A	15218	G	CYTB	Non-synonymous	T158A	0.986	0.02	0.169
Modern terminal	U	A	15218	G	CYTB	Non-synonymous	T158A	0.986	0.02	0.169
Pre-terminal	U	A	15218	G	CYTB	Non-synonymous	T158A	0.986	0.02	0.169
Pre-terminal	U	A	15218	G	CYTB	Non-synonymous	T158A	0.986	0.02	0.169
Modern terminal	JT	A	15218	G	CYTB	Non-synonymous	T158A	0.986	0.02	0.169
Modern terminal	JT	A	15218	G	CYTB	Non-synonymous	T158A	0.986	0.02	0.169
Modern terminal	JT	A	15218	G	CYTB	Non-synonymous	T158A	0.986	0.02	0.169
Modern terminal	N1	A	15218	G	CYTB	Non-synonymous	T158A	0.986	0.02	0.169
Ancient terminal	N2	A	15218	G	CYTB	Non-synonymous	T158A	0.986	0.02	0.169
Modern terminal	R0	C	15219	T	CYTB	Non-synonymous	T158M	1	0	0.117
Pre-terminal	R0	C	15219	T	CYTB	Non-synonymous	T158M	1	0	0.117
Modern terminal	R0	G	15221	A	CYTB	Non-synonymous	D159N	0.001	0.48	0.202
Modern terminal	R0	G	15221	A	CYTB	Non-synonymous	D159N	0.001	0.48	0.202
Modern terminal	R0	G	15221	A	CYTB	Non-synonymous	D159N	0.001	0.48	0.202
Modern terminal	R0	G	15221	A	CYTB	Non-synonymous	D159N	0.001	0.48	0.202
Modern terminal	R0	G	15221	A	CYTB	Non-synonymous	D159N	0.001	0.48	0.202
Modern terminal	U	G	15221	A	CYTB	Non-synonymous	D159N	0.001	0.48	0.202
Modern terminal	U	G	15221	A	CYTB	Non-synonymous	D159N	0.001	0.48	0.202
Modern terminal	U	G	15221	A	CYTB	Non-synonymous	D159N	0.001	0.48	0.202
Modern terminal	U	G	15221	A	CYTB	Non-synonymous	D159N	0.001	0.48	0.202
Modern terminal	JT	G	15221	A	CYTB	Non-synonymous	D159N	0.001	0.48	0.202
Modern terminal	N1	G	15221	C	CYTB	Non-synonymous	D159H	0.682	0	0.28
Modern terminal	R0	A	15222	G	CYTB	Non-synonymous	D159G	0.493	0.01	0.433

Modern terminal	U	A	15222	G	CYTB	Non-synonymous	D159G	0.493	0.01	0.433
Modern terminal	U	A	15222	G	CYTB	Non-synonymous	D159G	0.493	0.01	0.433
Pre-terminal	JT	A	15222	G	CYTB	Non-synonymous	D159G	0.493	0.01	0.433
Pre-terminal	X	A	15222	G	CYTB	Non-synonymous	D159G	0.493	0.01	0.433
Modern terminal	U	G	15227	A	CYTB	Non-synonymous	V161I	0.712	0	0.093
Modern terminal	JT	G	15227	A	CYTB	Non-synonymous	V161I	0.712	0	0.093
Modern terminal	JT	G	15227	A	CYTB	Non-synonymous	V161I	0.712	0	0.093
Modern terminal	R0	A	15236	G	CYTB	Non-synonymous	I164V	0	0.07	0.049
Modern terminal	R0	A	15236	G	CYTB	Non-synonymous	I164V	0	0.07	0.049
Modern terminal	R0	A	15236	G	CYTB	Non-synonymous	I164V	0	0.07	0.049
Pre-terminal	R0	A	15236	G	CYTB	Non-synonymous	I164V	0	0.07	0.049
Pre-terminal	R0	A	15236	G	CYTB	Non-synonymous	I164V	0	0.07	0.049
Pre-terminal	R0	A	15236	G	CYTB	Non-synonymous	I164V	0	0.07	0.049
Modern terminal	U	A	15236	G	CYTB	Non-synonymous	I164V	0	0.07	0.049
Modern terminal	U	A	15236	G	CYTB	Non-synonymous	I164V	0	0.07	0.049
Modern terminal	U	A	15236	G	CYTB	Non-synonymous	I164V	0	0.07	0.049
Modern terminal	U	A	15236	G	CYTB	Non-synonymous	I164V	0	0.07	0.049
Modern terminal	U	A	15236	G	CYTB	Non-synonymous	I164V	0	0.07	0.049
Modern terminal	JT	A	15236	G	CYTB	Non-synonymous	I164V	0	0.07	0.049
Pre-terminal	JT	A	15236	G	CYTB	Non-synonymous	I164V	0	0.07	0.049
Pre-terminal	U	T	15237	C	CYTB	Non-synonymous	I164T	0.723	0	0.265
Modern terminal	N1	T	15237	C	CYTB	Non-synonymous	I164T	0.723	0	0.265
Modern terminal	R0	C	15238	A	CYTB	Non-synonymous	I164M	0.946	0	0.186
Modern terminal	R0	C	15238	A	CYTB	Non-synonymous	I164M	0.946	0	0.186
Modern terminal	R0	G	15245	A	CYTB	Non-synonymous	G167S	1	0	0.287
Modern terminal	R0	G	15245	A	CYTB	Non-synonymous	G167S	1	0	0.287
Modern terminal	JT	G	15245	A	CYTB	Non-synonymous	G167S	1	0	0.287
Modern terminal	N1	G	15245	A	CYTB	Non-synonymous	G167S	1	0	0.287
Modern terminal	U	T	15248	C	CYTB	Non-synonymous	Y168H	1	0	0.361
Modern terminal	U	A	15249	G	CYTB	Non-synonymous	Y168C	1	0	0.392
Modern terminal	JT	G	15254	A	CYTB	Non-synonymous	V170M	0.999	0	0.179
Pre-terminal	R0	T	15255	C	CYTB	Non-synonymous	V170A	1	0	0.189
Modern terminal	R0	G	15257	A	CYTB	Non-synonymous	D171N	0	0.02	0.281
Modern terminal	R0	G	15257	A	CYTB	Non-synonymous	D171N	0	0.02	0.281
Modern terminal	R0	G	15257	A	CYTB	Non-synonymous	D171N	0	0.02	0.281
Modern terminal	R0	G	15257	A	CYTB	Non-synonymous	D171N	0	0.02	0.281
Modern terminal	R0	G	15257	A	CYTB	Non-synonymous	D171N	0	0.02	0.281
Modern terminal	U	G	15257	A	CYTB	Non-synonymous	D171N	0	0.02	0.281
Pre-terminal	U	G	15257	A	CYTB	Non-synonymous	D171N	0	0.02	0.281
Pre-terminal	U	G	15257	A	CYTB	Non-synonymous	D171N	0	0.02	0.281
Pre-terminal	U	G	15257	A	CYTB	Non-synonymous	D171N	0	0.02	0.281
Ancient terminal	JT	G	15257	A	CYTB	Non-synonymous	D171N	0	0.02	0.281
Pre-terminal	JT	G	15257	A	CYTB	Non-synonymous	D171N	0	0.02	0.281
Pre-terminal	JT	G	15257	A	CYTB	Non-synonymous	D171N	0	0.02	0.281
Modern terminal	R0	A	15258	G	CYTB	Non-synonymous	D171G	0.005	0	0.49
Modern terminal	U	A	15258	G	CYTB	Non-synonymous	D171G	0.005	0	0.49
Pre-terminal	U	A	15258	G	CYTB	Non-synonymous	D171G	0.005	0	0.49
Modern terminal	JT	A	15258	G	CYTB	Non-synonymous	D171G	0.005	0	0.49
Ancient terminal	JT	C	15259	A	CYTB	Non-synonymous	D171E	0.016	0	0.225
Modern terminal	R0	G	15261	A	CYTB	Non-synonymous	S172N	0	0.03	0.118

Modern terminal	R0	G	15261	A	CYTB	Non-synonymous	S172N	0	0.03	0.118
Pre-terminal	R0	G	15261	A	CYTB	Non-synonymous	S172N	0	0.03	0.118
Pre-terminal	U	G	15261	A	CYTB	Non-synonymous	S172N	0	0.03	0.118
Modern terminal	X	C	15263	T	CYTB	Non-synonymous	P173S	0.291	0	0.221
Modern terminal	R0	A	15266	G	CYTB	Non-synonymous	T174A	0.997	0	0.197
Modern terminal	U	A	15266	G	CYTB	Non-synonymous	T174A	0.997	0	0.197
Modern terminal	U	A	15266	G	CYTB	Non-synonymous	T174A	0.997	0	0.197
Ancient terminal	R0	C	15269	T	CYTB	Non-synonymous	L175F	1	0	0.268
Modern terminal	R0	A	15272	G	CYTB	Non-synonymous	T176A	0.997	0	0.191
Pre-terminal	R0	A	15272	G	CYTB	Non-synonymous	T176A	0.997	0	0.191
Modern terminal	R0	A	15284	G	CYTB	Non-synonymous	T180A	0.981	1	0.294
Ancient terminal	R0	T	15287	C	CYTB	Non-synonymous	F181L	0.001	0.03	0.23
Modern terminal	R0	T	15287	C	CYTB	Non-synonymous	F181L	0.001	0.03	0.23
Modern terminal	R0	T	15287	C	CYTB	Non-synonymous	F181L	0.001	0.03	0.23
Modern terminal	R0	T	15287	C	CYTB	Non-synonymous	F181L	0.001	0.03	0.23
Modern terminal	R0	T	15287	C	CYTB	Non-synonymous	F181L	0.001	0.03	0.23
Modern terminal	R0	T	15287	C	CYTB	Non-synonymous	F181L	0.001	0.03	0.23
Modern terminal	R0	T	15287	C	CYTB	Non-synonymous	F181L	0.001	0.03	0.23
Modern terminal	R0	T	15287	C	CYTB	Non-synonymous	F181L	0.001	0.03	0.23
Modern terminal	R0	T	15287	C	CYTB	Non-synonymous	F181L	0.001	0.03	0.23
Pre-terminal	R0	T	15287	C	CYTB	Non-synonymous	F181L	0.001	0.03	0.23
Pre-terminal	R0	T	15287	C	CYTB	Non-synonymous	F181L	0.001	0.03	0.23
Modern terminal	U	T	15287	C	CYTB	Non-synonymous	F181L	0.001	0.03	0.23
Modern terminal	U	T	15287	C	CYTB	Non-synonymous	F181L	0.001	0.03	0.23
Modern terminal	JT	T	15287	C	CYTB	Non-synonymous	F181L	0.001	0.03	0.23
Modern terminal	JT	T	15287	C	CYTB	Non-synonymous	F181L	0.001	0.03	0.23
Pre-terminal	JT	T	15287	C	CYTB	Non-synonymous	F181L	0.001	0.03	0.23
Pre-terminal	N1	T	15287	C	CYTB	Non-synonymous	F181L	0.001	0.03	0.23
Pre-terminal	N1	T	15287	C	CYTB	Non-synonymous	F181L	0.001	0.03	0.23
Modern terminal	N2	T	15287	C	CYTB	Non-synonymous	F181L	0.001	0.03	0.23
Modern terminal	U	A	15296	G	CYTB	Non-synonymous	I184V	0.051	0.06	0.101
Modern terminal	R0	T	15297	C	CYTB	Non-synonymous	I184T	0.979	0.01	0.224
Pre-terminal	R0	T	15297	C	CYTB	Non-synonymous	I184T	0.979	0.01	0.224
Modern terminal	R0	C	15298	A	CYTB	Non-synonymous	I184M	0.196	0.03	0.21
Pre-terminal	R0	T	15300	C	CYTB	Non-synonymous	L185S	1	0	0.394
Modern terminal	U	T	15300	C	CYTB	Non-synonymous	L185S	1	0	0.394
Pre-terminal	X	T	15300	C	CYTB	Non-synonymous	L185S	1	0	0.394
Modern terminal	U	A	15308	G	CYTB	Non-synonymous	I188V	0.005	0.09	0.061
Modern terminal	N2	A	15308	G	CYTB	Non-synonymous	I188V	0.005	0.09	0.061
Modern terminal	R0	A	15311	G	CYTB	Non-synonymous	I189V	0.121	0.22	0.069
Modern terminal	N1	T	15312	C	CYTB	Non-synonymous	I189T	0.133	0	0.129
Modern terminal	R0	G	15314	A	CYTB	Non-synonymous	A190T	0	0.44	0.036
Modern terminal	R0	G	15314	A	CYTB	Non-synonymous	A190T	0	0.44	0.036
Modern terminal	R0	G	15314	A	CYTB	Non-synonymous	A190T	0	0.44	0.036
Modern terminal	R0	G	15314	A	CYTB	Non-synonymous	A190T	0	0.44	0.036
Modern terminal	R0	G	15314	A	CYTB	Non-synonymous	A190T	0	0.44	0.036
Modern terminal	R0	G	15314	A	CYTB	Non-synonymous	A190T	0	0.44	0.036
Modern terminal	R0	G	15314	A	CYTB	Non-synonymous	A190T	0	0.44	0.036
Modern terminal	R0	G	15314	A	CYTB	Non-synonymous	A190T	0	0.44	0.036
Modern terminal	R0	G	15314	A	CYTB	Non-synonymous	A190T	0	0.44	0.036

Modern terminal	R0	G	15314	A	CYTB	Non-synonymous	A190T	0	0.44	0.036
Modern terminal	R0	G	15314	A	CYTB	Non-synonymous	A190T	0	0.44	0.036
Modern terminal	R0	G	15314	A	CYTB	Non-synonymous	A190T	0	0.44	0.036
Modern terminal	R0	G	15314	A	CYTB	Non-synonymous	A190T	0	0.44	0.036
Modern terminal	R0	G	15314	A	CYTB	Non-synonymous	A190T	0	0.44	0.036
Pre-terminal	R0	G	15314	A	CYTB	Non-synonymous	A190T	0	0.44	0.036
Pre-terminal	R0	G	15314	A	CYTB	Non-synonymous	A190T	0	0.44	0.036
Pre-terminal	R0	G	15314	A	CYTB	Non-synonymous	A190T	0	0.44	0.036
Pre-terminal	R0	G	15314	A	CYTB	Non-synonymous	A190T	0	0.44	0.036
Pre-terminal	R0	G	15314	A	CYTB	Non-synonymous	A190T	0	0.44	0.036
Pre-terminal	R0	G	15314	A	CYTB	Non-synonymous	A190T	0	0.44	0.036
Modern terminal	U	G	15314	A	CYTB	Non-synonymous	A190T	0	0.44	0.036
Modern terminal	U	G	15314	A	CYTB	Non-synonymous	A190T	0	0.44	0.036
Modern terminal	U	G	15314	A	CYTB	Non-synonymous	A190T	0	0.44	0.036
Modern terminal	U	G	15314	A	CYTB	Non-synonymous	A190T	0	0.44	0.036
Modern terminal	U	G	15314	A	CYTB	Non-synonymous	A190T	0	0.44	0.036
Modern terminal	U	G	15314	A	CYTB	Non-synonymous	A190T	0	0.44	0.036
Modern terminal	U	G	15314	A	CYTB	Non-synonymous	A190T	0	0.44	0.036
Modern terminal	U	G	15314	A	CYTB	Non-synonymous	A190T	0	0.44	0.036
Modern terminal	U	G	15314	A	CYTB	Non-synonymous	A190T	0	0.44	0.036
Modern terminal	U	G	15314	A	CYTB	Non-synonymous	A190T	0	0.44	0.036
Pre-terminal	U	G	15314	A	CYTB	Non-synonymous	A190T	0	0.44	0.036
Pre-terminal	U	G	15314	A	CYTB	Non-synonymous	A190T	0	0.44	0.036
Modern terminal	JT	G	15314	A	CYTB	Non-synonymous	A190T	0	0.44	0.036
Modern terminal	JT	G	15314	A	CYTB	Non-synonymous	A190T	0	0.44	0.036
Modern terminal	JT	G	15314	A	CYTB	Non-synonymous	A190T	0	0.44	0.036
Modern terminal	JT	G	15314	A	CYTB	Non-synonymous	A190T	0	0.44	0.036
Pre-terminal	JT	G	15314	A	CYTB	Non-synonymous	A190T	0	0.44	0.036
Pre-terminal	JT	G	15314	A	CYTB	Non-synonymous	A190T	0	0.44	0.036
Pre-terminal	JT	G	15314	A	CYTB	Non-synonymous	A190T	0	0.44	0.036
Pre-terminal	JT	G	15314	A	CYTB	Non-synonymous	A190T	0	0.44	0.036
Modern terminal	N1	G	15314	A	CYTB	Non-synonymous	A190T	0	0.44	0.036
Modern terminal	N1	G	15314	A	CYTB	Non-synonymous	A190T	0	0.44	0.036
Pre-terminal	X	G	15314	A	CYTB	Non-synonymous	A190T	0	0.44	0.036
Pre-terminal	X	G	15314	A	CYTB	Non-synonymous	A190T	0	0.44	0.036
Modern terminal	N2	G	15314	A	CYTB	Non-synonymous	A190T	0	0.44	0.036
Modern terminal	N2	G	15314	A	CYTB	Non-synonymous	A190T	0	0.44	0.036
Modern terminal	R0	C	15315	T	CYTB	Non-synonymous	A190V	0.047	0.03	0.091
Modern terminal	R0	C	15315	T	CYTB	Non-synonymous	A190V	0.047	0.03	0.091
Modern terminal	R0	C	15315	T	CYTB	Non-synonymous	A190V	0.047	0.03	0.091
Modern terminal	R0	C	15315	T	CYTB	Non-synonymous	A190V	0.047	0.03	0.091
Modern terminal	R0	C	15315	T	CYTB	Non-synonymous	A190V	0.047	0.03	0.091
Modern terminal	R0	C	15315	T	CYTB	Non-synonymous	A190V	0.047	0.03	0.091
Pre-terminal	R0	C	15315	T	CYTB	Non-synonymous	A190V	0.047	0.03	0.091
Pre-terminal	R0	C	15315	T	CYTB	Non-synonymous	A190V	0.047	0.03	0.091
Pre-terminal	R0	C	15315	T	CYTB	Non-synonymous	A190V	0.047	0.03	0.091
Pre-terminal	R0	C	15315	T	CYTB	Non-synonymous	A190V	0.047	0.03	0.091
Modern terminal	U	C	15315	T	CYTB	Non-synonymous	A190V	0.047	0.03	0.091
Pre-terminal	N1	C	15315	T	CYTB	Non-synonymous	A190V	0.047	0.03	0.091
Modern terminal	N2	C	15315	T	CYTB	Non-synonymous	A190V	0.047	0.03	0.091

Modern terminal	R0	G	15317	A	CYTB	Non-synonymous	A191T	0.004	0.02	0.106
Modern terminal	R0	G	15317	A	CYTB	Non-synonymous	A191T	0.004	0.02	0.106
Modern terminal	R0	G	15317	A	CYTB	Non-synonymous	A191T	0.004	0.02	0.106
Modern terminal	R0	G	15317	A	CYTB	Non-synonymous	A191T	0.004	0.02	0.106
Modern terminal	R0	G	15317	A	CYTB	Non-synonymous	A191T	0.004	0.02	0.106
Pre-terminal	R0	G	15317	A	CYTB	Non-synonymous	A191T	0.004	0.02	0.106
Pre-terminal	R0	G	15317	A	CYTB	Non-synonymous	A191T	0.004	0.02	0.106
Ancient terminal	U	G	15317	A	CYTB	Non-synonymous	A191T	0.004	0.02	0.106
Modern terminal	U	G	15317	A	CYTB	Non-synonymous	A191T	0.004	0.02	0.106
Modern terminal	U	G	15317	A	CYTB	Non-synonymous	A191T	0.004	0.02	0.106
Modern terminal	U	G	15317	A	CYTB	Non-synonymous	A191T	0.004	0.02	0.106
Modern terminal	U	G	15317	A	CYTB	Non-synonymous	A191T	0.004	0.02	0.106
Modern terminal	U	G	15317	A	CYTB	Non-synonymous	A191T	0.004	0.02	0.106
Modern terminal	U	G	15317	A	CYTB	Non-synonymous	A191T	0.004	0.02	0.106
Modern terminal	U	G	15317	A	CYTB	Non-synonymous	A191T	0.004	0.02	0.106
Modern terminal	U	G	15317	A	CYTB	Non-synonymous	A191T	0.004	0.02	0.106
Modern terminal	JT	G	15317	A	CYTB	Non-synonymous	A191T	0.004	0.02	0.106
Modern terminal	JT	G	15317	A	CYTB	Non-synonymous	A191T	0.004	0.02	0.106
Modern terminal	JT	G	15317	A	CYTB	Non-synonymous	A191T	0.004	0.02	0.106
Modern terminal	N1	G	15317	A	CYTB	Non-synonymous	A191T	0.004	0.02	0.106
Modern terminal	N1	G	15317	A	CYTB	Non-synonymous	A191T	0.004	0.02	0.106
Modern terminal	X	G	15317	A	CYTB	Non-synonymous	A191T	0.004	0.02	0.106
Modern terminal	U	C	15318	A	CYTB	Non-synonymous	A191D	0.882	0	0.232
Pre-terminal	R0	C	15320	A	CYTB	Non-synonymous	L192M	0.998	0.07	0.105
Modern terminal	R0	G	15323	A	CYTB	Non-synonymous	A193T	0.001	0.11	0.04
Modern terminal	R0	G	15323	A	CYTB	Non-synonymous	A193T	0.001	0.11	0.04
Modern terminal	R0	G	15323	A	CYTB	Non-synonymous	A193T	0.001	0.11	0.04
Modern terminal	R0	G	15323	A	CYTB	Non-synonymous	A193T	0.001	0.11	0.04
Modern terminal	R0	G	15323	A	CYTB	Non-synonymous	A193T	0.001	0.11	0.04
Pre-terminal	R0	G	15323	A	CYTB	Non-synonymous	A193T	0.001	0.11	0.04
Pre-terminal	R0	G	15323	A	CYTB	Non-synonymous	A193T	0.001	0.11	0.04
Pre-terminal	R0	G	15323	A	CYTB	Non-synonymous	A193T	0.001	0.11	0.04
Modern terminal	U	G	15323	A	CYTB	Non-synonymous	A193T	0.001	0.11	0.04
Modern terminal	U	G	15323	A	CYTB	Non-synonymous	A193T	0.001	0.11	0.04
Modern terminal	U	G	15323	A	CYTB	Non-synonymous	A193T	0.001	0.11	0.04
Pre-terminal	U	G	15323	A	CYTB	Non-synonymous	A193T	0.001	0.11	0.04
Pre-terminal	U	G	15323	A	CYTB	Non-synonymous	A193T	0.001	0.11	0.04
Modern terminal	JT	G	15323	A	CYTB	Non-synonymous	A193T	0.001	0.11	0.04
Modern terminal	JT	G	15323	A	CYTB	Non-synonymous	A193T	0.001	0.11	0.04
Pre-terminal	JT	G	15323	A	CYTB	Non-synonymous	A193T	0.001	0.11	0.04
Pre-terminal	JT	G	15323	A	CYTB	Non-synonymous	A193T	0.001	0.11	0.04
Pre-terminal	N1	G	15323	A	CYTB	Non-synonymous	A193T	0.001	0.11	0.04
Modern terminal	R0	C	15324	T	CYTB	Non-synonymous	A193V	0.001	0.65	0.053
Modern terminal	R0	C	15324	T	CYTB	Non-synonymous	A193V	0.001	0.65	0.053
Modern terminal	R0	C	15324	T	CYTB	Non-synonymous	A193V	0.001	0.65	0.053
Pre-terminal	R0	C	15324	T	CYTB	Non-synonymous	A193V	0.001	0.65	0.053
Modern terminal	U	C	15324	T	CYTB	Non-synonymous	A193V	0.001	0.65	0.053
Ancient terminal	R0	A	15326	G	CYTB	Non-synonymous	T194A	0	0.36	0.097
Ancient terminal	R0	A	15326	G	CYTB	Non-synonymous	T194A	0	0.36	0.097
Ancient terminal	R0	A	15326	G	CYTB	Non-synonymous	T194A	0	0.36	0.097

Modern terminal	R0	A	15326	G	CYTB	Non-synonymous	T194A	0	0.36	0.097
Modern terminal	JT	A	15326	G	CYTB	Non-synonymous	T194A	0	0.36	0.097
Ancient terminal	R0	C	15327	T	CYTB	Non-synonymous	T194M	0.773	1	0.074
Modern terminal	R0	C	15327	T	CYTB	Non-synonymous	T194M	0.773	1	0.074
Modern terminal	U	C	15327	G	CYTB	Nonsense				
Modern terminal	U	C	15327	T	CYTB	Non-synonymous	T194M	0.773	1	0.074
Modern terminal	U	C	15327	T	CYTB	Non-synonymous	T194M	0.773	1	0.074
Pre-terminal	U	C	15327	T	CYTB	Non-synonymous	T194M	0.773	1	0.074
Modern terminal	JT	C	15327	T	CYTB	Non-synonymous	T194M	0.773	1	0.074
Modern terminal	U	T	15330	C	CYTB	Non-synonymous	L195P	1	0	0.558
Modern terminal	JT	T	15341	C	CYTB	Non-synonymous	F199L	1	0	0.284
Pre-terminal	JT	T	15341	C	CYTB	Non-synonymous	F199L	1	0	0.284
Ancient terminal	U	A	15351	T	CYTB	Non-synonymous	E202V	0.99	0	0.208
Modern terminal	R0	A	15353	G	CYTB	Non-synonymous	T203A	0.01	0	0.107
Pre-terminal	U	A	15353	G	CYTB	Non-synonymous	T203A	0.01	0	0.107
Ancient terminal	JT	G	15356	A	CYTB	Nonsense				
Modern terminal	U	T	15372	C	CYTB	Non-synonymous	L209P	1	0.01	0.491
Pre-terminal	R0	A	15377	G	CYTB	Non-synonymous	I211V	0.945	0.03	0.059
Pre-terminal	R0	A	15377	G	CYTB	Non-synonymous	I211V	0.945	0.03	0.059
Modern terminal	JT	T	15378	C	CYTB	Non-synonymous	I211T	1	0.03	0.141
Modern terminal	R0	A	15380	C	CYTB	Non-synonymous	T212P	0.001	1	0.113
Modern terminal	R0	A	15380	G	CYTB	Non-synonymous	T212A	0.002	0.22	0.088
Pre-terminal	R0	A	15380	G	CYTB	Non-synonymous	T212A	0.002	0.22	0.088
Pre-terminal	U	A	15380	G	CYTB	Non-synonymous	T212A	0.002	0.22	0.088
Pre-terminal	U	A	15380	G	CYTB	Non-synonymous	T212A	0.002	0.22	0.088
Modern terminal	JT	A	15380	C	CYTB	Non-synonymous	T212P	0.001	1	0.113
Modern terminal	JT	A	15380	G	CYTB	Non-synonymous	T212A	0.002	0.22	0.088
Modern terminal	R0	C	15381	T	CYTB	Non-synonymous	T212I	0.056	0.45	0.094
Pre-terminal	R0	C	15381	T	CYTB	Non-synonymous	T212I	0.056	0.45	0.094
Pre-terminal	U	C	15381	T	CYTB	Non-synonymous	T212I	0.056	0.45	0.094
Pre-terminal	U	C	15381	T	CYTB	Non-synonymous	T212I	0.056	0.45	0.094
Modern terminal	JT	C	15381	T	CYTB	Non-synonymous	T212I	0.056	0.45	0.094
Modern terminal	JT	C	15381	T	CYTB	Non-synonymous	T212I	0.056	0.45	0.094
Pre-terminal	JT	C	15381	T	CYTB	Non-synonymous	T212I	0.056	0.45	0.094
Modern terminal	R0	T	15383	C	CYTB	Non-synonymous	S213P	0.997	0.01	0.399
Modern terminal	R0	T	15383	C	CYTB	Non-synonymous	S213P	0.997	0.01	0.399
Modern terminal	R0	T	15383	C	CYTB	Non-synonymous	S213P	0.997	0.01	0.399
Ancient terminal	R0	C	15386	G	CYTB	Non-synonymous	H214D	0.557	1	0.284
Modern terminal	R0	C	15386	A	CYTB	Non-synonymous	H214N	0.742	0.17	0.216
Modern terminal	R0	C	15386	T	CYTB	Non-synonymous	H214Y	0.152	0	0.189
Modern terminal	JT	C	15390	G	CYTB	Non-synonymous	S215C	0.857	0.07	0.164
Pre-terminal	JT	C	15390	A	CYTB	Non-synonymous	S215Y	0.957	0	0.181
Modern terminal	R0	A	15401	G	CYTB	Non-synonymous	T219A	0.001	0.01	0.167
Modern terminal	R0	A	15401	C	CYTB	Non-synonymous	T219P	0.99	1	0.194
Modern terminal	U	A	15401	G	CYTB	Non-synonymous	T219A	0.001	0.01	0.167
Modern terminal	R0	C	15402	G	CYTB	Non-synonymous	T219S	0.174	0	0.075
Pre-terminal	R0	C	15402	G	CYTB	Non-synonymous	T219S	0.174	0	0.075
Ancient terminal	U	C	15402	T	CYTB	Non-synonymous	T219I	0.506	0	0.174
Modern terminal	JT	C	15402	T	CYTB	Non-synonymous	T219I	0.506	0	0.174
Modern terminal	U	T	15404	C	CYTB	Non-synonymous	F220L	1	0.01	0.265

Pre-terminal	U	T	15404	C	CYTB	Non-synonymous	F220L	1	0.01	0.265
Modern terminal	R0	T	15413	C	CYTB	Non-synonymous	Y223H	1	0	0.265
Ancient terminal	JT	A	15414	T	CYTB	Non-synonymous	Y223F	0.999	0	0.188
Modern terminal	JT	A	15414	T	CYTB	Non-synonymous	Y223F	0.999	0	0.188
Modern terminal	JT	A	15414	T	CYTB	Non-synonymous	Y223F	0.999	0	0.188
Pre-terminal	R0	A	15422	T	CYTB	Non-synonymous	I226F	0.216	0.02	0.322
Modern terminal	N1	A	15422	G	CYTB	Non-synonymous	I226V	0.001	0.04	0.098
Pre-terminal	JT	T	15423	C	CYTB	Non-synonymous	I226T	0.005	0.08	0.196
Pre-terminal	R0	C	15424	A	CYTB	Non-synonymous	I226M	0.946	0.03	0.179
Modern terminal	R0	G	15431	A	CYTB	Non-synonymous	A229T	0.02	0.03	0.059
Modern terminal	R0	G	15431	A	CYTB	Non-synonymous	A229T	0.02	0.03	0.059
Pre-terminal	R0	G	15431	A	CYTB	Non-synonymous	A229T	0.02	0.03	0.059
Pre-terminal	R0	G	15431	A	CYTB	Non-synonymous	A229T	0.02	0.03	0.059
Modern terminal	U	G	15431	A	CYTB	Non-synonymous	A229T	0.02	0.03	0.059
Modern terminal	U	G	15431	A	CYTB	Non-synonymous	A229T	0.02	0.03	0.059
Modern terminal	U	G	15431	A	CYTB	Non-synonymous	A229T	0.02	0.03	0.059
Modern terminal	U	G	15431	A	CYTB	Non-synonymous	A229T	0.02	0.03	0.059
Pre-terminal	U	G	15431	A	CYTB	Non-synonymous	A229T	0.02	0.03	0.059
Pre-terminal	U	G	15431	A	CYTB	Non-synonymous	A229T	0.02	0.03	0.059
Pre-terminal	JT	G	15431	A	CYTB	Non-synonymous	A229T	0.02	0.03	0.059
Pre-terminal	N1	G	15431	A	CYTB	Non-synonymous	A229T	0.02	0.03	0.059
Modern terminal	R0	C	15434	T	CYTB	Non-synonymous	L230F	1	0.01	0.171
Pre-terminal	JT	C	15434	T	CYTB	Non-synonymous	L230F	1	0.01	0.171
Ancient terminal	U	T	15435	A	CYTB	Non-synonymous	L230H	1	0	0.444
Modern terminal	R0	T	15440	A	CYTB	Non-synonymous	L232M	0.652	0.11	0.137
Modern terminal	U	C	15446	T	CYTB	Non-synonymous	L234F	0.999	0.32	0.185
Modern terminal	U	C	15446	T	CYTB	Non-synonymous	L234F	0.999	0.32	0.185
Modern terminal	JT	C	15446	T	CYTB	Non-synonymous	L234F	0.999	0.32	0.185
Modern terminal	R0	T	15449	C	CYTB	Non-synonymous	F235L	0.001	1	0.185
Pre-terminal	R0	T	15449	C	CYTB	Non-synonymous	F235L	0.001	1	0.185
Pre-terminal	R0	T	15449	C	CYTB	Non-synonymous	F235L	0.001	1	0.185
Pre-terminal	U	T	15449	C	CYTB	Non-synonymous	F235L	0.001	1	0.185
Pre-terminal	U	T	15449	C	CYTB	Non-synonymous	F235L	0.001	1	0.185
Modern terminal	JT	T	15449	C	CYTB	Non-synonymous	F235L	0.001	1	0.185
Modern terminal	JT	T	15449	C	CYTB	Non-synonymous	F235L	0.001	1	0.185
Modern terminal	X	T	15449	C	CYTB	Non-synonymous	F235L	0.001	1	0.185
Modern terminal	JT	T	15450	C	CYTB	Non-synonymous	F235S	0.979	0	0.459
Modern terminal	R0	C	15452	A	CYTB	Non-synonymous	L236I	0.289	1	0.086
Modern terminal	U	C	15452	A	CYTB	Non-synonymous	L236I	0.289	1	0.086
Modern terminal	JT	C	15452	A	CYTB	Non-synonymous	L236I	0.289	1	0.086
Modern terminal	N1	C	15452	A	CYTB	Non-synonymous	L236I	0.289	1	0.086
Pre-terminal	JT	T	15453	C	CYTB	Non-synonymous	L236P	1	0	0.542
Modern terminal	R0	T	15456	G	CYTB	Non-synonymous	L237R	0.938	0	0.41
Ancient terminal	R0	T	15458	C	CYTB	Non-synonymous	S238P	0.083	0.08	0.213
Modern terminal	R0	T	15458	C	CYTB	Non-synonymous	S238P	0.083	0.08	0.213
Modern terminal	U	T	15458	C	CYTB	Non-synonymous	S238P	0.083	0.08	0.213
Pre-terminal	U	T	15458	C	CYTB	Non-synonymous	S238P	0.083	0.08	0.213
Pre-terminal	U	T	15458	C	CYTB	Non-synonymous	S238P	0.083	0.08	0.213
Modern terminal	JT	T	15458	C	CYTB	Non-synonymous	S238P	0.083	0.08	0.213
Modern terminal	JT	T	15458	C	CYTB	Non-synonymous	S238P	0.083	0.08	0.213

Modern terminal	N1	T	15458	C	CYTB	Non-synonymous	S238P	0.083	0.08	0.213
Modern terminal	R0	C	15459	T	CYTB	Non-synonymous	S238F	0.143	0.11	0.124
Pre-terminal	R0	C	15459	T	CYTB	Non-synonymous	S238F	0.143	0.11	0.124
Ancient terminal	JT	C	15459	T	CYTB	Non-synonymous	S238F	0.143	0.11	0.124
Modern terminal	JT	C	15459	T	CYTB	Non-synonymous	S238F	0.143	0.11	0.124
Modern terminal	N1	C	15459	T	CYTB	Non-synonymous	S238F	0.143	0.11	0.124
Modern terminal	JT	T	15462	C	CYTB	Non-synonymous	L239S	1	0	0.264
Modern terminal	X	T	15462	C	CYTB	Non-synonymous	L239S	1	0	0.264
Modern terminal	R0	A	15464	T	CYTB	Non-synonymous	M240L	0.271	0.57	0.164
Ancient terminal	R0	T	15465	C	CYTB	Non-synonymous	M240T	0.991	0.31	0.136
Modern terminal	R0	T	15465	C	CYTB	Non-synonymous	M240T	0.991	0.31	0.136
Pre-terminal	R0	T	15465	C	CYTB	Non-synonymous	M240T	0.991	0.31	0.136
Pre-terminal	R0	T	15465	C	CYTB	Non-synonymous	M240T	0.991	0.31	0.136
Pre-terminal	R0	T	15465	C	CYTB	Non-synonymous	M240T	0.991	0.31	0.136
Ancient terminal	R0	A	15467	G	CYTB	Non-synonymous	T241A	0.015	0.22	0.087
Modern terminal	R0	A	15467	G	CYTB	Non-synonymous	T241A	0.015	0.22	0.087
Modern terminal	R0	A	15467	G	CYTB	Non-synonymous	T241A	0.015	0.22	0.087
Ancient terminal	JT	A	15467	G	CYTB	Non-synonymous	T241A	0.015	0.22	0.087
Modern terminal	JT	A	15467	G	CYTB	Non-synonymous	T241A	0.015	0.22	0.087
Pre-terminal	JT	A	15467	G	CYTB	Non-synonymous	T241A	0.015	0.22	0.087
Ancient terminal	R0	C	15468	T	CYTB	Non-synonymous	T241M	0.893	0.1	0.079
Pre-terminal	R0	C	15468	T	CYTB	Non-synonymous	T241M	0.893	0.1	0.079
Modern terminal	U	C	15468	T	CYTB	Non-synonymous	T241M	0.893	0.1	0.079
Pre-terminal	JT	C	15468	T	CYTB	Non-synonymous	T241M	0.893	0.1	0.079
Pre-terminal	U	A	15473	G	CYTB	Non-synonymous	T243A	0.033	0.02	0.1
Ancient terminal	R0	T	15479	C	CYTB	Non-synonymous	F245L	0.012	0.02	0.225
Modern terminal	R0	T	15479	C	CYTB	Non-synonymous	F245L	0.012	0.02	0.225
Modern terminal	R0	T	15479	C	CYTB	Non-synonymous	F245L	0.012	0.02	0.225
Modern terminal	R0	T	15479	C	CYTB	Non-synonymous	F245L	0.012	0.02	0.225
Modern terminal	R0	T	15479	C	CYTB	Non-synonymous	F245L	0.012	0.02	0.225
Modern terminal	R0	T	15479	C	CYTB	Non-synonymous	F245L	0.012	0.02	0.225
Modern terminal	U	T	15479	C	CYTB	Non-synonymous	F245L	0.012	0.02	0.225
Modern terminal	U	T	15479	C	CYTB	Non-synonymous	F245L	0.012	0.02	0.225
Modern terminal	U	T	15479	C	CYTB	Non-synonymous	F245L	0.012	0.02	0.225
Pre-terminal	U	T	15479	C	CYTB	Non-synonymous	F245L	0.012	0.02	0.225
Pre-terminal	U	T	15479	C	CYTB	Non-synonymous	F245L	0.012	0.02	0.225
Modern terminal	JT	T	15479	C	CYTB	Non-synonymous	F245L	0.012	0.02	0.225
Pre-terminal	JT	T	15479	C	CYTB	Non-synonymous	F245L	0.012	0.02	0.225
Modern terminal	R0	T	15482	G	CYTB	Non-synonymous	S246A	0.001	0.05	0.086
Pre-terminal	R0	T	15482	C	CYTB	Non-synonymous	S246P	0.762	0	0.26
Modern terminal	U	T	15482	C	CYTB	Non-synonymous	S246P	0.762	0	0.26
Modern terminal	N1	T	15482	G	CYTB	Non-synonymous	S246A	0.001	0.05	0.086
Modern terminal	JT	C	15486	A	CYTB	Non-synonymous	P247Q	1	0	0.198
Modern terminal	R0	C	15491	T	CYTB	Non-synonymous	L249F	0.999	0.01	0.169
Ancient terminal	R0	C	15494	A	CYTB	Non-synonymous	L250M	1	0	0.133
Modern terminal	R0	G	15497	A	CYTB	Non-synonymous	G251S	0.004	0.03	0.217
Modern terminal	R0	G	15497	A	CYTB	Non-synonymous	G251S	0.004	0.03	0.217
Modern terminal	R0	G	15497	A	CYTB	Non-synonymous	G251S	0.004	0.03	0.217
Modern terminal	R0	G	15497	A	CYTB	Non-synonymous	G251S	0.004	0.03	0.217
Modern terminal	R0	G	15497	A	CYTB	Non-synonymous	G251S	0.004	0.03	0.217

Modern terminal	R0	G	15497	A	CYTB	Non-synonymous	G251S	0.004	0.03	0.217
Pre-terminal	R0	G	15497	A	CYTB	Non-synonymous	G251S	0.004	0.03	0.217
Pre-terminal	R0	G	15497	A	CYTB	Non-synonymous	G251S	0.004	0.03	0.217
Pre-terminal	R0	G	15497	A	CYTB	Non-synonymous	G251S	0.004	0.03	0.217
Pre-terminal	R0	G	15497	A	CYTB	Non-synonymous	G251S	0.004	0.03	0.217
Pre-terminal	R0	G	15497	A	CYTB	Non-synonymous	G251S	0.004	0.03	0.217
Pre-terminal	R0	G	15497	A	CYTB	Non-synonymous	G251S	0.004	0.03	0.217
Ancient terminal	U	G	15497	A	CYTB	Non-synonymous	G251S	0.004	0.03	0.217
Modern terminal	U	G	15497	A	CYTB	Non-synonymous	G251S	0.004	0.03	0.217
Modern terminal	U	G	15497	A	CYTB	Non-synonymous	G251S	0.004	0.03	0.217
Modern terminal	U	G	15497	A	CYTB	Non-synonymous	G251S	0.004	0.03	0.217
Modern terminal	U	G	15497	A	CYTB	Non-synonymous	G251S	0.004	0.03	0.217
Modern terminal	U	G	15497	A	CYTB	Non-synonymous	G251S	0.004	0.03	0.217
Modern terminal	U	G	15497	A	CYTB	Non-synonymous	G251S	0.004	0.03	0.217
Pre-terminal	U	G	15497	A	CYTB	Non-synonymous	G251S	0.004	0.03	0.217
Pre-terminal	U	G	15497	A	CYTB	Non-synonymous	G251S	0.004	0.03	0.217
Modern terminal	JT	G	15497	A	CYTB	Non-synonymous	G251S	0.004	0.03	0.217
Modern terminal	JT	G	15497	A	CYTB	Non-synonymous	G251S	0.004	0.03	0.217
Modern terminal	N1	G	15497	A	CYTB	Non-synonymous	G251S	0.004	0.03	0.217
Modern terminal	X	G	15497	A	CYTB	Non-synonymous	G251S	0.004	0.03	0.217
Modern terminal	R0	G	15498	A	CYTB	Non-synonymous	G251D	0.913	0	0.356
Modern terminal	R0	G	15498	A	CYTB	Non-synonymous	G251D	0.913	0	0.356
Modern terminal	R0	G	15498	A	CYTB	Non-synonymous	G251D	0.913	0	0.356
Pre-terminal	R0	G	15498	A	CYTB	Non-synonymous	G251D	0.913	0	0.356
Pre-terminal	U	G	15498	A	CYTB	Non-synonymous	G251D	0.913	0	0.356
Modern terminal	JT	G	15498	A	CYTB	Non-synonymous	G251D	0.913	0	0.356
Modern terminal	N1	G	15498	A	CYTB	Non-synonymous	G251D	0.913	0	0.356
Modern terminal	R0	G	15500	A	CYTB	Non-synonymous	D252N	0.994	0	0.11
Modern terminal	JT	G	15500	A	CYTB	Non-synonymous	D252N	0.994	0	0.11
Modern terminal	N1	G	15500	A	CYTB	Non-synonymous	D252N	0.994	0	0.11
Pre-terminal	N2	G	15500	A	CYTB	Non-synonymous	D252N	0.994	0	0.11
Ancient terminal	U	C	15503	T	CYTB	Non-synonymous	P253S	0.999	0	0.191
Modern terminal	U	G	15506	A	CYTB	Non-synonymous	D254N	0.045	0	0.208
Ancient terminal	JT	G	15506	A	CYTB	Non-synonymous	D254N	0.045	0	0.208
Pre-terminal	JT	A	15509	G	CYTB	Non-synonymous	N255D	0.003	0	0.221
Pre-terminal	R0	T	15512	C	CYTB	Non-synonymous	Y256H	1	0	0.249
Ancient terminal	U	T	15512	C	CYTB	Non-synonymous	Y256H	1	0	0.249
Pre-terminal	R0	A	15515	G	CYTB	Non-synonymous	T257A	0.029	0	0.2
Ancient terminal	R0	T	15519	C	CYTB	Non-synonymous	L258P	0.966	1	0.473
Modern terminal	R0	T	15519	C	CYTB	Non-synonymous	L258P	0.966	1	0.473
Modern terminal	R0	T	15519	A	CYTB	Non-synonymous	L258Q	0.003	0.04	0.216
Modern terminal	R0	T	15519	G	CYTB	Non-synonymous	L258R	0.044	0	0.385
Modern terminal	R0	T	15519	C	CYTB	Non-synonymous	L258P	0.966	1	0.473
Modern terminal	R0	T	15519	C	CYTB	Non-synonymous	L258P	0.966	1	0.473
Modern terminal	R0	T	15519	C	CYTB	Non-synonymous	L258P	0.966	1	0.473
Pre-terminal	R0	T	15519	A	CYTB	Non-synonymous	L258Q	0.003	0.04	0.216
Pre-terminal	R0	T	15519	C	CYTB	Non-synonymous	L258P	0.966	1	0.473
Pre-terminal	R0	T	15519	C	CYTB	Non-synonymous	L258P	0.966	1	0.473
Pre-terminal	R0	T	15519	C	CYTB	Non-synonymous	L258P	0.966	1	0.473
Pre-terminal	R0	T	15519	C	CYTB	Non-synonymous	L258P	0.966	1	0.473

Pre-terminal	R0	T	15519	C	CYTB	Non-synonymous	L258P	0.966	1	0.473
Pre-terminal	R0	T	15519	C	CYTB	Non-synonymous	L258P	0.966	1	0.473
Pre-terminal	R0	T	15519	C	CYTB	Non-synonymous	L258P	0.966	1	0.473
Pre-terminal	R0	T	15519	C	CYTB	Non-synonymous	L258P	0.966	1	0.473
Modern terminal	U	T	15519	C	CYTB	Non-synonymous	L258P	0.966	1	0.473
Pre-terminal	U	T	15519	C	CYTB	Non-synonymous	L258P	0.966	1	0.473
Pre-terminal	U	T	15519	C	CYTB	Non-synonymous	L258P	0.966	1	0.473
Modern terminal	JT	T	15519	C	CYTB	Non-synonymous	L258P	0.966	1	0.473
Pre-terminal	JT	T	15519	C	CYTB	Non-synonymous	L258P	0.966	1	0.473
Pre-terminal	JT	T	15519	C	CYTB	Non-synonymous	L258P	0.966	1	0.473
Pre-terminal	JT	T	15519	C	CYTB	Non-synonymous	L258P	0.966	1	0.473
Pre-terminal	JT	T	15519	C	CYTB	Non-synonymous	L258P	0.966	1	0.473
Modern terminal	N1	T	15519	C	CYTB	Non-synonymous	L258P	0.966	1	0.473
Modern terminal	R0	G	15521	A	CYTB	Non-synonymous	A259T	0.997	0	0.159
Pre-terminal	U	G	15521	A	CYTB	Non-synonymous	A259T	0.997	0	0.159
Pre-terminal	R0	A	15524	G	CYTB	Non-synonymous	N260D	0.995	0	0.177
Modern terminal	R0	A	15525	G	CYTB	Non-synonymous	N260S	0.996	0	0.112
Pre-terminal	U	A	15525	G	CYTB	Non-synonymous	N260S	0.996	0	0.112
Ancient terminal	N2	A	15525	G	CYTB	Non-synonymous	N260S	0.996	0	0.112
Modern terminal	R0	C	15527	T	CYTB	Non-synonymous	P261S	0.999	0	0.172
Pre-terminal	U	A	15533	G	CYTB	Non-synonymous	N263D	0.048	0	0.145
Modern terminal	JT	A	15534	G	CYTB	Non-synonymous	N263S	0	0.23	0.099
Modern terminal	JT	A	15534	G	CYTB	Non-synonymous	N263S	0	0.23	0.099
Ancient terminal	X	C	15542	T	CYTB	Non-synonymous	P266S	0.999	0.01	0.235
Modern terminal	JT	C	15543	T	CYTB	Non-synonymous	P266L	1	0	0.264
Modern terminal	R0	A	15546	G	CYTB	Non-synonymous	H267R	0.97	0	0.171
Ancient terminal	U	G	15557	C	CYTB	Non-synonymous	E271Q	0.992	0	0.189
Ancient terminal	R0	G	15575	A	CYTB	Non-synonymous	A277T	0.999	0	0.192
Modern terminal	U	G	15575	A	CYTB	Non-synonymous	A277T	0.999	0	0.192
Ancient terminal	U	A	15581	G	CYTB	Non-synonymous	T279A	0	1	0.083
Modern terminal	R0	G	15596	A	CYTB	Non-synonymous	V284I	0	1	0.069
Modern terminal	R0	G	15596	A	CYTB	Non-synonymous	V284I	0	1	0.069
Modern terminal	R0	G	15596	A	CYTB	Non-synonymous	V284I	0	1	0.069
Pre-terminal	R0	G	15596	A	CYTB	Non-synonymous	V284I	0	1	0.069
Pre-terminal	R0	G	15596	A	CYTB	Non-synonymous	V284I	0	1	0.069
Pre-terminal	U	G	15596	A	CYTB	Non-synonymous	V284I	0	1	0.069
Modern terminal	JT	G	15596	A	CYTB	Non-synonymous	V284I	0	1	0.069
Pre-terminal	JT	G	15596	A	CYTB	Non-synonymous	V284I	0	1	0.069
Pre-terminal	JT	G	15596	A	CYTB	Non-synonymous	V284I	0	1	0.069
Pre-terminal	R0	T	15597	C	CYTB	Non-synonymous	V284A	0.954	0	0.216
Modern terminal	R0	A	15603	G	CYTB	Non-synonymous	N286S	0.996	0.01	0.094
Modern terminal	R0	A	15603	G	CYTB	Non-synonymous	N286S	0.996	0.01	0.094
Modern terminal	R0	G	15617	A	CYTB	Non-synonymous	V291I	0.878	0	0.061
Modern terminal	R0	G	15617	A	CYTB	Non-synonymous	V291I	0.878	0	0.061
Modern terminal	R0	G	15617	A	CYTB	Non-synonymous	V291I	0.878	0	0.061
Modern terminal	R0	G	15617	A	CYTB	Non-synonymous	V291I	0.878	0	0.061
Modern terminal	R0	G	15617	A	CYTB	Non-synonymous	V291I	0.878	0	0.061
Pre-terminal	R0	G	15617	A	CYTB	Non-synonymous	V291I	0.878	0	0.061
Pre-terminal	R0	G	15617	A	CYTB	Non-synonymous	V291I	0.878	0	0.061
Modern terminal	U	G	15617	A	CYTB	Non-synonymous	V291I	0.878	0	0.061

Modern terminal	U	G	15617	A	CYTB	Non-synonymous	V291I	0.878	0	0.061
Modern terminal	U	G	15617	A	CYTB	Non-synonymous	V291I	0.878	0	0.061
Modern terminal	JT	G	15617	A	CYTB	Non-synonymous	V291I	0.878	0	0.061
Pre-terminal	JT	G	15617	A	CYTB	Non-synonymous	V291I	0.878	0	0.061
Modern terminal	N1	G	15617	A	CYTB	Non-synonymous	V291I	0.878	0	0.061
Modern terminal	R0	T	15618	C	CYTB	Non-synonymous	V291A	1	0	0.119
Modern terminal	R0	T	15618	C	CYTB	Non-synonymous	V291A	1	0	0.119
Pre-terminal	R0	T	15618	C	CYTB	Non-synonymous	V291A	1	0	0.119
Modern terminal	U	T	15618	C	CYTB	Non-synonymous	V291A	1	0	0.119
Modern terminal	U	T	15618	C	CYTB	Non-synonymous	V291A	1	0	0.119
Pre-terminal	R0	C	15620	T	CYTB	Non-synonymous	L292F	0.892	0	0.182
Modern terminal	U	C	15620	T	CYTB	Non-synonymous	L292F	0.892	0	0.182
Modern terminal	N1	C	15624	T	CYTB	Non-synonymous	A293V	0.999	0	0.166
Modern terminal	N2	T	15630	C	CYTB	Non-synonymous	L295S	0.999	0	0.389
Modern terminal	U	T	15635	C	CYTB	Non-synonymous	S297P	0.997	0	0.456
Pre-terminal	R0	A	15638	T	CYTB	Non-synonymous	I298F	0.994	0	0.225
Modern terminal	R0	T	15639	C	CYTB	Non-synonymous	I298T	1	0	0.121
Modern terminal	JT	C	15641	T	CYTB	Non-synonymous	L299F	1	0	0.23
Modern terminal	U	T	15642	C	CYTB	Non-synonymous	L299P	1	0	0.701
Modern terminal	R0	A	15644	G	CYTB	Non-synonymous	I300V	0.945	0.37	0.047
Modern terminal	X	T	15645	C	CYTB	Non-synonymous	I300T	1	0	0.085
Modern terminal	R0	G	15650	A	CYTB	Non-synonymous	A302T	0.001	0.1	0.059
Modern terminal	R0	G	15650	A	CYTB	Non-synonymous	A302T	0.001	0.1	0.059
Modern terminal	R0	G	15650	A	CYTB	Non-synonymous	A302T	0.001	0.1	0.059
Modern terminal	U	G	15650	A	CYTB	Non-synonymous	A302T	0.001	0.1	0.059
Modern terminal	U	G	15650	A	CYTB	Non-synonymous	A302T	0.001	0.1	0.059
Pre-terminal	U	G	15650	A	CYTB	Non-synonymous	A302T	0.001	0.1	0.059
Ancient terminal	JT	G	15650	T	CYTB	Non-synonymous	A302S	0.009	0	0.059
Modern terminal	JT	G	15650	A	CYTB	Non-synonymous	A302T	0.001	0.1	0.059
Modern terminal	JT	G	15650	A	CYTB	Non-synonymous	A302T	0.001	0.1	0.059
Pre-terminal	X	G	15650	A	CYTB	Non-synonymous	A302T	0.001	0.1	0.059
Modern terminal	R0	C	15651	T	CYTB	Non-synonymous	A302V	0.001	0.11	0.059
Modern terminal	U	C	15651	T	CYTB	Non-synonymous	A302V	0.001	0.11	0.059
Pre-terminal	R0	A	15653	G	CYTB	Non-synonymous	M303V	0.007	0.35	0.081
Pre-terminal	R0	A	15653	G	CYTB	Non-synonymous	M303V	0.007	0.35	0.081
Pre-terminal	U	A	15653	T	CYTB	Non-synonymous	M303L	0.002	0.99	0.087
Modern terminal	JT	A	15653	G	CYTB	Non-synonymous	M303V	0.007	0.35	0.081
Modern terminal	N2	A	15653	G	CYTB	Non-synonymous	M303V	0.007	0.35	0.081
Modern terminal	JT	T	15654	C	CYTB	Non-synonymous	M303T	0	0.1	0.097
Pre-terminal	X	T	15654	C	CYTB	Non-synonymous	M303T	0	0.1	0.097
Modern terminal	R0	A	15656	G	CYTB	Non-synonymous	I304V	0.045	0.57	0.041
Modern terminal	U	T	15657	C	CYTB	Non-synonymous	I304T	0.05	0.01	0.056
Modern terminal	U	T	15657	C	CYTB	Non-synonymous	I304T	0.05	0.01	0.056
Pre-terminal	U	T	15657	C	CYTB	Non-synonymous	I304T	0.05	0.01	0.056
Pre-terminal	R0	A	15662	G	CYTB	Non-synonymous	I306V	0	0.05	0.048
Pre-terminal	U	A	15662	G	CYTB	Non-synonymous	I306V	0	0.05	0.048
Modern terminal	JT	A	15662	G	CYTB	Non-synonymous	I306V	0	0.05	0.048
Pre-terminal	JT	A	15662	G	CYTB	Non-synonymous	I306V	0	0.05	0.048
Pre-terminal	JT	A	15662	G	CYTB	Non-synonymous	I306V	0	0.05	0.048
Pre-terminal	JT	A	15662	G	CYTB	Non-synonymous	I306V	0	0.05	0.048

Modern terminal	R0	T	15663	C	CYTB	Non-synonymous	I306T	0.002	0.06	0.069
Pre-terminal	U	T	15663	C	CYTB	Non-synonymous	I306T	0.002	0.06	0.069
Modern terminal	JT	T	15663	C	CYTB	Non-synonymous	I306T	0.002	0.06	0.069
Pre-terminal	JT	T	15663	C	CYTB	Non-synonymous	I306T	0.002	0.06	0.069
Modern terminal	R0	A	15671	G	CYTB	Non-synonymous	M309V	0.007	0.05	0.088
Modern terminal	U	A	15671	C	CYTB	Non-synonymous	M309L	0.001	0.18	0.076
Modern terminal	U	A	15671	G	CYTB	Non-synonymous	M309V	0.007	0.05	0.088
Modern terminal	U	A	15671	G	CYTB	Non-synonymous	M309V	0.007	0.05	0.088
Modern terminal	U	A	15671	T	CYTB	Non-synonymous	M309L	0.001	0.18	0.076
Modern terminal	R0	T	15672	C	CYTB	Non-synonymous	M309T	0	1	0.054
Modern terminal	R0	T	15672	C	CYTB	Non-synonymous	M309T	0	1	0.054
Pre-terminal	R0	T	15672	C	CYTB	Non-synonymous	M309T	0	1	0.054
Pre-terminal	R0	T	15672	C	CYTB	Non-synonymous	M309T	0	1	0.054
Ancient terminal	U	T	15672	C	CYTB	Non-synonymous	M309T	0	1	0.054
Pre-terminal	JT	T	15672	C	CYTB	Non-synonymous	M309T	0	1	0.054
Pre-terminal	JT	T	15672	C	CYTB	Non-synonymous	M309T	0	1	0.054
Pre-terminal	X	T	15672	C	CYTB	Non-synonymous	M309T	0	1	0.054
Ancient terminal	U	T	15674	C	CYTB	Non-synonymous	S310P	0.236	0	0.217
Modern terminal	U	T	15674	C	CYTB	Non-synonymous	S310P	0.236	0	0.217
Modern terminal	R0	A	15684	G	CYTB	Non-synonymous	Q313R	0.937	1	0.132
Pre-terminal	JT	A	15684	G	CYTB	Non-synonymous	Q313R	0.937	1	0.132
Ancient terminal	R0	T	15690	A	CYTB	Non-synonymous	M315K	0.932	0	0.321
Modern terminal	U	T	15690	C	CYTB	Non-synonymous	M315T	0.997	0	0.132
Modern terminal	R0	A	15692	G	CYTB	Non-synonymous	M316V	0.756	0.01	0.07
Modern terminal	R0	A	15692	G	CYTB	Non-synonymous	M316V	0.756	0.01	0.07
Ancient terminal	R0	T	15693	A	CYTB	Non-synonymous	M316K	0.079	0	0.315
Pre-terminal	R0	T	15693	C	CYTB	Non-synonymous	M316T	0.002	0.09	0.163
Pre-terminal	R0	T	15693	C	CYTB	Non-synonymous	M316T	0.002	0.09	0.163
Modern terminal	U	T	15693	C	CYTB	Non-synonymous	M316T	0.002	0.09	0.163
Pre-terminal	JT	T	15693	C	CYTB	Non-synonymous	M316T	0.002	0.09	0.163
Pre-terminal	N1	T	15705	C	CYTB	Non-synonymous	L320P	1	0	0.306
Modern terminal	U	G	15708	C	CYTB	Non-synonymous	S321T	0.943	0.08	0.056
Pre-terminal	R0	T	15713	G	CYTB	Non-synonymous	S323A	0.001	0.02	0.051
Pre-terminal	U	T	15713	A	CYTB	Non-synonymous	S323T	0.001	0.08	0.051
Ancient terminal	JT	C	15716	T	CYTB	Non-synonymous	L324F	0.308	0.01	0.086
Ancient terminal	R0	T	15717	A	CYTB	Non-synonymous	L324H	0.998	0	0.237
Modern terminal	R0	A	15724	T	CYTB	Non-synonymous	W326C	1	0	0.562
Modern terminal	JT	A	15724	C	CYTB	Non-synonymous	W326C	1	0	0.562
Modern terminal	R0	C	15725	T	CYTB	Non-synonymous	L327F	0.001	0.04	0.086
Ancient terminal	N1	C	15725	T	CYTB	Non-synonymous	L327F	0.001	0.04	0.086
Modern terminal	N1	C	15725	T	CYTB	Non-synonymous	L327F	0.001	0.04	0.086
Ancient terminal	R0	T	15726	A	CYTB	Non-synonymous	L327H	0.999	0	0.295
Ancient terminal	R0	G	15731	A	CYTB	Non-synonymous	A329T	0	0.06	0.066
Modern terminal	R0	G	15731	A	CYTB	Non-synonymous	A329T	0	0.06	0.066
Pre-terminal	R0	G	15731	A	CYTB	Non-synonymous	A329T	0	0.06	0.066
Modern terminal	U	G	15731	A	CYTB	Non-synonymous	A329T	0	0.06	0.066
Modern terminal	U	G	15731	A	CYTB	Non-synonymous	A329T	0	0.06	0.066
Modern terminal	JT	G	15731	A	CYTB	Non-synonymous	A329T	0	0.06	0.066
Modern terminal	JT	G	15731	A	CYTB	Non-synonymous	A329T	0	0.06	0.066
Modern terminal	JT	G	15731	A	CYTB	Non-synonymous	A329T	0	0.06	0.066

Modern terminal	JT	G	15731	A	CYTB	Non-synonymous	A329T	0	0.06	0.066
Pre-terminal	JT	G	15731	A	CYTB	Non-synonymous	A329T	0	0.06	0.066
Pre-terminal	JT	G	15731	A	CYTB	Non-synonymous	A329T	0	0.06	0.066
Modern terminal	N1	G	15731	A	CYTB	Non-synonymous	A329T	0	0.06	0.066
Modern terminal	N1	G	15731	A	CYTB	Non-synonymous	A329T	0	0.06	0.066
Modern terminal	N1	G	15731	A	CYTB	Non-synonymous	A329T	0	0.06	0.066
Modern terminal	N1	G	15731	A	CYTB	Non-synonymous	A329T	0	0.06	0.066
Pre-terminal	N1	G	15731	A	CYTB	Non-synonymous	A329T	0	0.06	0.066
Modern terminal	X	G	15731	A	CYTB	Non-synonymous	A329T	0	0.06	0.066
Pre-terminal	U	C	15732	T	CYTB	Non-synonymous	A329V	0	1	0.05
Modern terminal	R0	G	15734	A	CYTB	Non-synonymous	A330T	0.001	0.03	0.094
Modern terminal	R0	G	15734	A	CYTB	Non-synonymous	A330T	0.001	0.03	0.094
Modern terminal	R0	G	15734	A	CYTB	Non-synonymous	A330T	0.001	0.03	0.094
Modern terminal	R0	G	15734	A	CYTB	Non-synonymous	A330T	0.001	0.03	0.094
Modern terminal	R0	G	15734	A	CYTB	Non-synonymous	A330T	0.001	0.03	0.094
Modern terminal	R0	G	15734	A	CYTB	Non-synonymous	A330T	0.001	0.03	0.094
Modern terminal	R0	G	15734	A	CYTB	Non-synonymous	A330T	0.001	0.03	0.094
Modern terminal	R0	G	15734	A	CYTB	Non-synonymous	A330T	0.001	0.03	0.094
Pre-terminal	R0	G	15734	A	CYTB	Non-synonymous	A330T	0.001	0.03	0.094
Pre-terminal	R0	G	15734	A	CYTB	Non-synonymous	A330T	0.001	0.03	0.094
Pre-terminal	R0	G	15734	A	CYTB	Non-synonymous	A330T	0.001	0.03	0.094
Modern terminal	U	G	15734	A	CYTB	Non-synonymous	A330T	0.001	0.03	0.094
Modern terminal	U	G	15734	A	CYTB	Non-synonymous	A330T	0.001	0.03	0.094
Modern terminal	U	G	15734	A	CYTB	Non-synonymous	A330T	0.001	0.03	0.094
Modern terminal	U	G	15734	A	CYTB	Non-synonymous	A330T	0.001	0.03	0.094
Modern terminal	U	G	15734	A	CYTB	Non-synonymous	A330T	0.001	0.03	0.094
Modern terminal	U	G	15734	A	CYTB	Non-synonymous	A330T	0.001	0.03	0.094
Pre-terminal	U	G	15734	A	CYTB	Non-synonymous	A330T	0.001	0.03	0.094
Pre-terminal	U	G	15734	A	CYTB	Non-synonymous	A330T	0.001	0.03	0.094
Pre-terminal	U	G	15734	A	CYTB	Non-synonymous	A330T	0.001	0.03	0.094
Ancient terminal	JT	G	15734	A	CYTB	Non-synonymous	A330T	0.001	0.03	0.094
Modern terminal	JT	G	15734	A	CYTB	Non-synonymous	A330T	0.001	0.03	0.094
Pre-terminal	JT	G	15734	A	CYTB	Non-synonymous	A330T	0.001	0.03	0.094
Pre-terminal	N1	G	15734	A	CYTB	Non-synonymous	A330T	0.001	0.03	0.094
Modern terminal	X	G	15734	A	CYTB	Non-synonymous	A330T	0.001	0.03	0.094
Pre-terminal	X	G	15734	A	CYTB	Non-synonymous	A330T	0.001	0.03	0.094
Modern terminal	R0	C	15735	T	CYTB	Non-synonymous	A330V	0.001	0	0.108
Modern terminal	X	C	15735	T	CYTB	Non-synonymous	A330V	0.001	0	0.108
Ancient terminal	U	G	15737	A	CYTB	Non-synonymous	D331N	0	0.13	0.045
Modern terminal	U	G	15737	A	CYTB	Non-synonymous	D331N	0	0.13	0.045
Modern terminal	JT	G	15737	A	CYTB	Non-synonymous	D331N	0	0.13	0.045
Ancient terminal	X	G	15737	A	CYTB	Non-synonymous	D331N	0	0.13	0.045
Modern terminal	JT	C	15740	T	CYTB	Non-synonymous	L332F	1	0.01	0.176
Pre-terminal	JT	C	15740	T	CYTB	Non-synonymous	L332F	1	0.01	0.176
Modern terminal	R0	A	15746	G	CYTB	Non-synonymous	I334V	0	0.08	0.041
Pre-terminal	R0	A	15746	G	CYTB	Non-synonymous	I334V	0	0.08	0.041
Modern terminal	U	A	15746	C	CYTB	Non-synonymous	I334L	0.001	0	0.116
Modern terminal	U	A	15746	G	CYTB	Non-synonymous	I334V	0	0.08	0.041
Ancient terminal	JT	A	15746	G	CYTB	Non-synonymous	I334V	0	0.08	0.041
Pre-terminal	N1	A	15746	G	CYTB	Non-synonymous	I334V	0	0.08	0.041

Ancient terminal	R0	T	15747	C	CYTB	Non-synonymous	I334T	0.001	1	0.059
Modern terminal	R0	T	15747	C	CYTB	Non-synonymous	I334T	0.001	1	0.059
Modern terminal	R0	T	15747	C	CYTB	Non-synonymous	I334T	0.001	1	0.059
Pre-terminal	R0	T	15747	C	CYTB	Non-synonymous	I334T	0.001	1	0.059
Pre-terminal	R0	T	15747	C	CYTB	Non-synonymous	I334T	0.001	1	0.059
Pre-terminal	R0	T	15747	C	CYTB	Non-synonymous	I334T	0.001	1	0.059
Modern terminal	U	T	15747	C	CYTB	Non-synonymous	I334T	0.001	1	0.059
Modern terminal	U	T	15747	C	CYTB	Non-synonymous	I334T	0.001	1	0.059
Modern terminal	JT	T	15747	C	CYTB	Non-synonymous	I334T	0.001	1	0.059
Ancient terminal	R0	A	15758	G	CYTB	Non-synonymous	I338V	0.003	0.01	0.075
Modern terminal	R0	A	15758	G	CYTB	Non-synonymous	I338V	0.003	0.01	0.075
Modern terminal	R0	A	15758	G	CYTB	Non-synonymous	I338V	0.003	0.01	0.075
Modern terminal	R0	A	15758	G	CYTB	Non-synonymous	I338V	0.003	0.01	0.075
Modern terminal	R0	A	15758	G	CYTB	Non-synonymous	I338V	0.003	0.01	0.075
Modern terminal	R0	A	15758	G	CYTB	Non-synonymous	I338V	0.003	0.01	0.075
Modern terminal	R0	A	15758	G	CYTB	Non-synonymous	I338V	0.003	0.01	0.075
Modern terminal	R0	A	15758	G	CYTB	Non-synonymous	I338V	0.003	0.01	0.075
Modern terminal	R0	A	15758	G	CYTB	Non-synonymous	I338V	0.003	0.01	0.075
Modern terminal	R0	A	15758	G	CYTB	Non-synonymous	I338V	0.003	0.01	0.075
Modern terminal	R0	A	15758	G	CYTB	Non-synonymous	I338V	0.003	0.01	0.075
Modern terminal	R0	A	15758	G	CYTB	Non-synonymous	I338V	0.003	0.01	0.075
Modern terminal	R0	A	15758	G	CYTB	Non-synonymous	I338V	0.003	0.01	0.075
Modern terminal	R0	A	15758	G	CYTB	Non-synonymous	I338V	0.003	0.01	0.075
Modern terminal	R0	A	15758	G	CYTB	Non-synonymous	I338V	0.003	0.01	0.075
Modern terminal	R0	A	15758	G	CYTB	Non-synonymous	I338V	0.003	0.01	0.075
Modern terminal	R0	A	15758	G	CYTB	Non-synonymous	I338V	0.003	0.01	0.075
Pre-terminal	R0	A	15758	G	CYTB	Non-synonymous	I338V	0.003	0.01	0.075
Pre-terminal	R0	A	15758	G	CYTB	Non-synonymous	I338V	0.003	0.01	0.075
Pre-terminal	R0	A	15758	G	CYTB	Non-synonymous	I338V	0.003	0.01	0.075
Pre-terminal	R0	A	15758	G	CYTB	Non-synonymous	I338V	0.003	0.01	0.075
Ancient terminal	U	A	15758	G	CYTB	Non-synonymous	I338V	0.003	0.01	0.075
Ancient terminal	U	A	15758	G	CYTB	Non-synonymous	I338V	0.003	0.01	0.075
Ancient terminal	U	A	15758	G	CYTB	Non-synonymous	I338V	0.003	0.01	0.075
Ancient terminal	U	A	15758	G	CYTB	Non-synonymous	I338V	0.003	0.01	0.075
Modern terminal	U	A	15758	G	CYTB	Non-synonymous	I338V	0.003	0.01	0.075
Modern terminal	U	A	15758	G	CYTB	Non-synonymous	I338V	0.003	0.01	0.075
Modern terminal	U	A	15758	G	CYTB	Non-synonymous	I338V	0.003	0.01	0.075
Modern terminal	U	A	15758	G	CYTB	Non-synonymous	I338V	0.003	0.01	0.075
Modern terminal	U	A	15758	G	CYTB	Non-synonymous	I338V	0.003	0.01	0.075
Modern terminal	U	A	15758	G	CYTB	Non-synonymous	I338V	0.003	0.01	0.075
Modern terminal	U	A	15758	G	CYTB	Non-synonymous	I338V	0.003	0.01	0.075
Modern terminal	U	A	15758	G	CYTB	Non-synonymous	I338V	0.003	0.01	0.075
Modern terminal	U	A	15758	G	CYTB	Non-synonymous	I338V	0.003	0.01	0.075
Modern terminal	U	A	15758	G	CYTB	Non-synonymous	I338V	0.003	0.01	0.075
Modern terminal	U	A	15758	G	CYTB	Non-synonymous	I338V	0.003	0.01	0.075
Modern terminal	U	A	15758	G	CYTB	Non-synonymous	I338V	0.003	0.01	0.075
Pre-terminal	U	A	15758	G	CYTB	Non-synonymous	I338V	0.003	0.01	0.075
Pre-terminal	U	A	15758	G	CYTB	Non-synonymous	I338V	0.003	0.01	0.075
Pre-terminal	U	A	15758	G	CYTB	Non-synonymous	I338V	0.003	0.01	0.075
Ancient terminal	JT	A	15758	G	CYTB	Non-synonymous	I338V	0.003	0.01	0.075

Modern terminal	JT	A	15758	G	CYTB	Non-synonymous	I338V	0.003	0.01	0.075
Modern terminal	JT	A	15758	G	CYTB	Non-synonymous	I338V	0.003	0.01	0.075
Modern terminal	JT	A	15758	G	CYTB	Non-synonymous	I338V	0.003	0.01	0.075
Modern terminal	JT	A	15758	G	CYTB	Non-synonymous	I338V	0.003	0.01	0.075
Modern terminal	JT	A	15758	G	CYTB	Non-synonymous	I338V	0.003	0.01	0.075
Modern terminal	JT	A	15758	G	CYTB	Non-synonymous	I338V	0.003	0.01	0.075
Modern terminal	JT	A	15758	G	CYTB	Non-synonymous	I338V	0.003	0.01	0.075
Pre-terminal	JT	A	15758	G	CYTB	Non-synonymous	I338V	0.003	0.01	0.075
Pre-terminal	JT	A	15758	G	CYTB	Non-synonymous	I338V	0.003	0.01	0.075
Modern terminal	N1	A	15758	G	CYTB	Non-synonymous	I338V	0.003	0.01	0.075
Modern terminal	N1	A	15758	G	CYTB	Non-synonymous	I338V	0.003	0.01	0.075
Pre-terminal	N1	A	15758	G	CYTB	Non-synonymous	I338V	0.003	0.01	0.075
Modern terminal	X	A	15758	G	CYTB	Non-synonymous	I338V	0.003	0.01	0.075
Modern terminal	X	A	15758	G	CYTB	Non-synonymous	I338V	0.003	0.01	0.075
Pre-terminal	N2	A	15758	G	CYTB	Non-synonymous	I338V	0.003	0.01	0.075
Modern terminal	JT	T	15759	C	CYTB	Non-synonymous	I338T	0.087	0	0.159
Modern terminal	JT	T	15759	C	CYTB	Non-synonymous	I338T	0.087	0	0.159
Ancient terminal	JT	G	15764	C	CYTB	Non-synonymous	G340R	1	0	0.428
Modern terminal	R0	G	15765	T	CYTB	Non-synonymous	G340V	1	0	0.492
Modern terminal	R0	G	15765	A	CYTB	Non-synonymous	G340E	1	0	0.516
Modern terminal	U	C	15770	T	CYTB	Non-synonymous	P342S	0.999	0.01	0.247
Modern terminal	R0	G	15773	A	CYTB	Non-synonymous	V343M	0.996	0	0.191
Modern terminal	R0	G	15773	A	CYTB	Non-synonymous	V343M	0.996	0	0.191
Modern terminal	R0	G	15773	A	CYTB	Non-synonymous	V343M	0.996	0	0.191
Modern terminal	R0	G	15773	A	CYTB	Non-synonymous	V343M	0.996	0	0.191
Modern terminal	R0	G	15773	A	CYTB	Non-synonymous	V343M	0.996	0	0.191
Pre-terminal	R0	G	15773	A	CYTB	Non-synonymous	V343M	0.996	0	0.191
Pre-terminal	R0	G	15773	A	CYTB	Non-synonymous	V343M	0.996	0	0.191
Pre-terminal	R0	G	15773	A	CYTB	Non-synonymous	V343M	0.996	0	0.191
Pre-terminal	R0	G	15773	A	CYTB	Non-synonymous	V343M	0.996	0	0.191
Pre-terminal	R0	G	15773	A	CYTB	Non-synonymous	V343M	0.996	0	0.191
Modern terminal	U	G	15773	A	CYTB	Non-synonymous	V343M	0.996	0	0.191
Modern terminal	U	G	15773	A	CYTB	Non-synonymous	V343M	0.996	0	0.191
Modern terminal	U	G	15773	A	CYTB	Non-synonymous	V343M	0.996	0	0.191
Modern terminal	U	G	15773	A	CYTB	Non-synonymous	V343M	0.996	0	0.191
Modern terminal	U	G	15773	A	CYTB	Non-synonymous	V343M	0.996	0	0.191
Modern terminal	U	G	15773	A	CYTB	Non-synonymous	V343M	0.996	0	0.191
Modern terminal	U	G	15773	A	CYTB	Non-synonymous	V343M	0.996	0	0.191
Modern terminal	U	G	15773	A	CYTB	Non-synonymous	V343M	0.996	0	0.191
Pre-terminal	U	G	15773	A	CYTB	Non-synonymous	V343M	0.996	0	0.191
Pre-terminal	U	G	15773	A	CYTB	Non-synonymous	V343M	0.996	0	0.191
Pre-terminal	U	G	15773	A	CYTB	Non-synonymous	V343M	0.996	0	0.191
Pre-terminal	U	G	15773	A	CYTB	Non-synonymous	V343M	0.996	0	0.191
Pre-terminal	U	G	15773	A	CYTB	Non-synonymous	V343M	0.996	0	0.191
Modern terminal	JT	G	15773	A	CYTB	Non-synonymous	V343M	0.996	0	0.191
Modern terminal	JT	G	15773	A	CYTB	Non-synonymous	V343M	0.996	0	0.191
Modern terminal	JT	G	15773	A	CYTB	Non-synonymous	V343M	0.996	0	0.191
Modern terminal	JT	G	15773	A	CYTB	Non-synonymous	V343M	0.996	0	0.191
Modern terminal	JT	G	15773	A	CYTB	Non-synonymous	V343M	0.996	0	0.191
Modern terminal	JT	G	15773	A	CYTB	Non-synonymous	V343M	0.996	0	0.191

Modern terminal	JT	G	15773	A	CYTB	Non-synonymous	V343M	0.996	0	0.191
Pre-terminal	JT	G	15773	A	CYTB	Non-synonymous	V343M	0.996	0	0.191
Pre-terminal	JT	G	15773	A	CYTB	Non-synonymous	V343M	0.996	0	0.191
Pre-terminal	JT	G	15773	A	CYTB	Non-synonymous	V343M	0.996	0	0.191
Modern terminal	N1	G	15773	A	CYTB	Non-synonymous	V343M	0.996	0	0.191
Modern terminal	N1	G	15773	A	CYTB	Non-synonymous	V343M	0.996	0	0.191
Pre-terminal	N1	G	15773	A	CYTB	Non-synonymous	V343M	0.996	0	0.191
Modern terminal	R0	T	15774	C	CYTB	Non-synonymous	V343A	0.999	0	0.218
Modern terminal	R0	T	15774	C	CYTB	Non-synonymous	V343A	0.999	0	0.218
Modern terminal	R0	T	15774	C	CYTB	Non-synonymous	V343A	0.999	0	0.218
Modern terminal	R0	T	15774	C	CYTB	Non-synonymous	V343A	0.999	0	0.218
Modern terminal	U	T	15774	C	CYTB	Non-synonymous	V343A	0.999	0	0.218
Modern terminal	U	T	15774	C	CYTB	Non-synonymous	V343A	0.999	0	0.218
Modern terminal	JT	T	15774	C	CYTB	Non-synonymous	V343A	0.999	0	0.218
Modern terminal	R0	A	15776	G	CYTB	Non-synonymous	S344G	0.74	0	0.222
Modern terminal	U	A	15776	G	CYTB	Non-synonymous	S344G	0.74	0	0.222
Pre-terminal	U	A	15776	G	CYTB	Non-synonymous	S344G	0.74	0	0.222
Pre-terminal	U	A	15776	G	CYTB	Non-synonymous	S344G	0.74	0	0.222
Ancient terminal	R0	G	15777	A	CYTB	Non-synonymous	S344N	0	0	0.264
Modern terminal	R0	G	15777	A	CYTB	Non-synonymous	S344N	0	0	0.264
Modern terminal	R0	G	15777	C	CYTB	Non-synonymous	S344T	0.025	0	0.158
Pre-terminal	R0	G	15777	A	CYTB	Non-synonymous	S344N	0	0	0.264
Ancient terminal	U	G	15777	A	CYTB	Non-synonymous	S344N	0	0	0.264
Modern terminal	U	G	15777	A	CYTB	Non-synonymous	S344N	0	0	0.264
Modern terminal	U	G	15777	A	CYTB	Non-synonymous	S344N	0	0	0.264
Modern terminal	U	G	15777	A	CYTB	Non-synonymous	S344N	0	0	0.264
Pre-terminal	U	G	15777	A	CYTB	Non-synonymous	S344N	0	0	0.264
Modern terminal	JT	G	15777	A	CYTB	Non-synonymous	S344N	0	0	0.264
Modern terminal	JT	G	15777	A	CYTB	Non-synonymous	S344N	0	0	0.264
Modern terminal	JT	G	15777	A	CYTB	Non-synonymous	S344N	0	0	0.264
Modern terminal	JT	G	15777	C	CYTB	Non-synonymous	S344T	0.025	0	0.158
Pre-terminal	JT	G	15777	A	CYTB	Non-synonymous	S344N	0	0	0.264
Modern terminal	N1	G	15777	A	CYTB	Non-synonymous	S344N	0	0	0.264
Modern terminal	X	G	15777	A	CYTB	Non-synonymous	S344N	0	0	0.264
Modern terminal	N2	G	15777	A	CYTB	Non-synonymous	S344N	0	0	0.264
Modern terminal	N2	G	15777	A	CYTB	Non-synonymous	S344N	0	0	0.264
Modern terminal	R0	T	15779	C	CYTB	Non-synonymous	Y345H	0.137	1	0.355
Pre-terminal	R0	T	15779	C	CYTB	Non-synonymous	Y345H	0.137	1	0.355
Pre-terminal	R0	T	15779	C	CYTB	Non-synonymous	Y345H	0.137	1	0.355
Modern terminal	U	T	15779	C	CYTB	Non-synonymous	Y345H	0.137	1	0.355
Modern terminal	R0	A	15788	G	CYTB	Non-synonymous	T348A	0.006	0	0.057
Pre-terminal	R0	C	15789	T	CYTB	Non-synonymous	T348I	0	1	0.046
Modern terminal	U	C	15789	T	CYTB	Non-synonymous	T348I	0	1	0.046
Pre-terminal	U	C	15789	T	CYTB	Non-synonymous	T348I	0	1	0.046
Pre-terminal	N1	C	15789	G	CYTB	Non-synonymous	T348S	0.028	0	0.045
Ancient terminal	U	A	15791	G	CYTB	Non-synonymous	I349V	0.001	0.06	0.053
Modern terminal	U	A	15791	G	CYTB	Non-synonymous	I349V	0.001	0.06	0.053
Pre-terminal	U	A	15791	G	CYTB	Non-synonymous	I349V	0.001	0.06	0.053
Modern terminal	R0	T	15792	C	CYTB	Non-synonymous	I349T	0.001	0.35	0.06
Pre-terminal	R0	T	15792	C	CYTB	Non-synonymous	I349T	0.001	0.35	0.06

Pre-terminal	R0	T	15792	C	CYTB	Non-synonymous	I349T	0.001	0.35	0.06
Modern terminal	U	T	15792	C	CYTB	Non-synonymous	I349T	0.001	0.35	0.06
Pre-terminal	U	T	15792	C	CYTB	Non-synonymous	I349T	0.001	0.35	0.06
Ancient terminal	JT	G	15797	C	CYTB	Non-synonymous	G351R	0.979	0	0.403
Modern terminal	R0	G	15803	A	CYTB	Non-synonymous	V353M	0.022	0.09	0.156
Modern terminal	R0	G	15803	A	CYTB	Non-synonymous	V353M	0.022	0.09	0.156
Pre-terminal	R0	G	15803	A	CYTB	Non-synonymous	V353M	0.022	0.09	0.156
Modern terminal	U	G	15803	A	CYTB	Non-synonymous	V353M	0.022	0.09	0.156
Modern terminal	U	G	15803	A	CYTB	Non-synonymous	V353M	0.022	0.09	0.156
Pre-terminal	U	G	15803	A	CYTB	Non-synonymous	V353M	0.022	0.09	0.156
Modern terminal	JT	G	15803	A	CYTB	Non-synonymous	V353M	0.022	0.09	0.156
Modern terminal	JT	G	15803	A	CYTB	Non-synonymous	V353M	0.022	0.09	0.156
Modern terminal	N1	G	15803	A	CYTB	Non-synonymous	V353M	0.022	0.09	0.156
Modern terminal	X	G	15803	A	CYTB	Non-synonymous	V353M	0.022	0.09	0.156
Modern terminal	N2	G	15803	A	CYTB	Non-synonymous	V353M	0.022	0.09	0.156
Modern terminal	R0	T	15804	C	CYTB	Non-synonymous	V353A	0.873	0.02	0.199
Modern terminal	R0	T	15804	C	CYTB	Non-synonymous	V353A	0.873	0.02	0.199
Modern terminal	R0	T	15804	C	CYTB	Non-synonymous	V353A	0.873	0.02	0.199
Modern terminal	R0	T	15804	C	CYTB	Non-synonymous	V353A	0.873	0.02	0.199
Pre-terminal	R0	T	15804	C	CYTB	Non-synonymous	V353A	0.873	0.02	0.199
Pre-terminal	R0	T	15804	C	CYTB	Non-synonymous	V353A	0.873	0.02	0.199
Pre-terminal	U	T	15804	C	CYTB	Non-synonymous	V353A	0.873	0.02	0.199
Pre-terminal	U	T	15804	C	CYTB	Non-synonymous	V353A	0.873	0.02	0.199
Modern terminal	JT	T	15804	C	CYTB	Non-synonymous	V353A	0.873	0.02	0.199
Pre-terminal	X	T	15804	C	CYTB	Non-synonymous	V353A	0.873	0.02	0.199
Modern terminal	R0	G	15812	A	CYTB	Non-synonymous	V356M	0.002	0.04	0.2
Modern terminal	R0	G	15812	A	CYTB	Non-synonymous	V356M	0.002	0.04	0.2
Pre-terminal	R0	G	15812	A	CYTB	Non-synonymous	V356M	0.002	0.04	0.2
Modern terminal	U	G	15812	A	CYTB	Non-synonymous	V356M	0.002	0.04	0.2
Modern terminal	U	G	15812	A	CYTB	Non-synonymous	V356M	0.002	0.04	0.2
Modern terminal	U	G	15812	A	CYTB	Non-synonymous	V356M	0.002	0.04	0.2
Ancient terminal	JT	G	15812	T	CYTB	Non-synonymous	V356L	0	0.05	0.265
Pre-terminal	JT	G	15812	A	CYTB	Non-synonymous	V356M	0.002	0.04	0.2
Pre-terminal	R0	T	15813	C	CYTB	Non-synonymous	V356A	0.009	0.01	0.282
Modern terminal	U	T	15813	C	CYTB	Non-synonymous	V356A	0.009	0.01	0.282
Pre-terminal	U	T	15813	C	CYTB	Non-synonymous	V356A	0.009	0.01	0.282
Ancient terminal	JT	T	15813	A	CYTB	Non-synonymous	V356E	0.735	0	0.596
Pre-terminal	JT	T	15813	C	CYTB	Non-synonymous	V356A	0.009	0.01	0.282
Ancient terminal	JT	T	15822	C	CYTB	Non-synonymous	F359S	1	0	0.581
Pre-terminal	U	C	15825	T	CYTB	Non-synonymous	T360M	0.438	0.24	0.149
Modern terminal	R0	A	15836	G	CYTB	Non-synonymous	I364V	0	0.84	0.052
Modern terminal	JT	A	15836	G	CYTB	Non-synonymous	I364V	0	0.84	0.052
Pre-terminal	JT	A	15836	G	CYTB	Non-synonymous	I364V	0	0.84	0.052
Modern terminal	X	A	15836	G	CYTB	Non-synonymous	I364V	0	0.84	0.052
Ancient terminal	U	C	15838	A	CYTB	Non-synonymous	I364M	0.005	0.01	0.141
Ancient terminal	R0	C	15845	T	CYTB	Non-synonymous	P367S	0.999	0	0.339
Modern terminal	R0	A	15848	G	CYTB	Non-synonymous	T368A	0	0.27	0.058
Modern terminal	R0	A	15848	G	CYTB	Non-synonymous	T368A	0	0.27	0.058
Modern terminal	R0	A	15848	G	CYTB	Non-synonymous	T368A	0	0.27	0.058
Modern terminal	R0	A	15848	G	CYTB	Non-synonymous	T368A	0	0.27	0.058

Pre-terminal	R0	A	15848	G	CYTB	Non-synonymous	T368A	0	0.27	0.058
Ancient terminal	U	A	15848	G	CYTB	Non-synonymous	T368A	0	0.27	0.058
Ancient terminal	U	A	15848	G	CYTB	Non-synonymous	T368A	0	0.27	0.058
Modern terminal	U	A	15848	G	CYTB	Non-synonymous	T368A	0	0.27	0.058
Modern terminal	U	A	15848	G	CYTB	Non-synonymous	T368A	0	0.27	0.058
Pre-terminal	U	A	15848	G	CYTB	Non-synonymous	T368A	0	0.27	0.058
Modern terminal	JT	A	15848	G	CYTB	Non-synonymous	T368A	0	0.27	0.058
Modern terminal	JT	A	15848	G	CYTB	Non-synonymous	T368A	0	0.27	0.058
Modern terminal	JT	A	15848	G	CYTB	Non-synonymous	T368A	0	0.27	0.058
Pre-terminal	JT	A	15848	G	CYTB	Non-synonymous	T368A	0	0.27	0.058
Ancient terminal	R0	C	15849	T	CYTB	Non-synonymous	T368I	0	1	0.074
Modern terminal	R0	C	15849	T	CYTB	Non-synonymous	T368I	0	1	0.074
Modern terminal	U	C	15849	T	CYTB	Non-synonymous	T368I	0	1	0.074
Modern terminal	U	C	15849	T	CYTB	Non-synonymous	T368I	0	1	0.074
Modern terminal	JT	C	15849	T	CYTB	Non-synonymous	T368I	0	1	0.074
Modern terminal	JT	C	15849	T	CYTB	Non-synonymous	T368I	0	1	0.074
Modern terminal	JT	C	15849	T	CYTB	Non-synonymous	T368I	0	1	0.074
Modern terminal	JT	C	15849	T	CYTB	Non-synonymous	T368I	0	1	0.074
Modern terminal	JT	C	15849	T	CYTB	Non-synonymous	T368I	0	1	0.074
Modern terminal	JT	C	15849	T	CYTB	Non-synonymous	T368I	0	1	0.074
Ancient terminal	U	A	15851	G	CYTB	Non-synonymous	I369V	0	0.61	0.073
Modern terminal	U	A	15851	G	CYTB	Non-synonymous	I369V	0	0.61	0.073
Modern terminal	JT	A	15851	G	CYTB	Non-synonymous	I369V	0	0.61	0.073
Modern terminal	JT	A	15851	G	CYTB	Non-synonymous	I369V	0	0.61	0.073
Modern terminal	JT	A	15851	G	CYTB	Non-synonymous	I369V	0	0.61	0.073
Modern terminal	R0	T	15852	C	CYTB	Non-synonymous	I369T	0	0.79	0.119
Pre-terminal	R0	T	15852	C	CYTB	Non-synonymous	I369T	0	0.79	0.119
Ancient terminal	U	T	15852	A	CYTB	Non-synonymous	I369N	0.176	0.01	0.314
Modern terminal	U	T	15852	C	CYTB	Non-synonymous	I369T	0	0.79	0.119
Modern terminal	JT	T	15852	C	CYTB	Non-synonymous	I369T	0	0.79	0.119
Modern terminal	JT	T	15852	C	CYTB	Non-synonymous	I369T	0	0.79	0.119
Modern terminal	JT	T	15852	C	CYTB	Non-synonymous	I369T	0	0.79	0.119
Pre-terminal	JT	T	15852	C	CYTB	Non-synonymous	I369T	0	0.79	0.119
Modern terminal	R0	A	15860	G	CYTB	Non-synonymous	I372V	0.001	0.12	0.074
Modern terminal	R0	A	15860	G	CYTB	Non-synonymous	I372V	0.001	0.12	0.074
Modern terminal	JT	A	15860	G	CYTB	Non-synonymous	I372V	0.001	0.12	0.074
Pre-terminal	R0	A	15866	G	CYTB	Non-synonymous	N374D	0.99	0.03	0.137
Modern terminal	U	A	15869	G	CYTB	Non-synonymous	K375E	0.121	0.01	0.252
Ancient terminal	R0	A	15872	G	CYTB	Non-synonymous	M376V	0.994	0.01	0.141
Modern terminal	U	A	15872	G	CYTB	Non-synonymous	M376V	0.994	0.01	0.141
Modern terminal	U	A	15872	T	CYTB	Non-synonymous	M376L	0.147	1	0.171
Pre-terminal	R0	T	15881	C	CYTB	Non-synonymous	W379R	1	0	0.558
Modern terminal	R0	G	15884	A	CYTB	Non-synonymous	A380T	0	1	0.136
Modern terminal	R0	G	15884	A	CYTB	Non-synonymous	A380T	0	1	0.136
Modern terminal	R0	G	15884	A	CYTB	Non-synonymous	A380T	0	1	0.136
Modern terminal	R0	G	15884	A	CYTB	Non-synonymous	A380T	0	1	0.136
Modern terminal	R0	G	15884	A	CYTB	Non-synonymous	A380T	0	1	0.136
Modern terminal	R0	G	15884	A	CYTB	Non-synonymous	A380T	0	1	0.136
Modern terminal	R0	G	15884	A	CYTB	Non-synonymous	A380T	0	1	0.136
Modern terminal	R0	G	15884	A	CYTB	Non-synonymous	A380T	0	1	0.136

Modern terminal	R0	G	15884	A	CYTB	Non-synonymous	A380T	0	1	0.136
Modern terminal	R0	G	15884	A	CYTB	Non-synonymous	A380T	0	1	0.136
Modern terminal	R0	G	15884	A	CYTB	Non-synonymous	A380T	0	1	0.136
Modern terminal	R0	G	15884	A	CYTB	Non-synonymous	A380T	0	1	0.136
Modern terminal	R0	G	15884	C	CYTB	Non-synonymous	A380P	0.167	0.06	0.292
Modern terminal	R0	G	15884	A	CYTB	Non-synonymous	A380T	0	1	0.136
Modern terminal	R0	G	15884	A	CYTB	Non-synonymous	A380T	0	1	0.136
Modern terminal	R0	G	15884	A	CYTB	Non-synonymous	A380T	0	1	0.136
Modern terminal	R0	G	15884	A	CYTB	Non-synonymous	A380T	0	1	0.136
Pre-terminal	R0	G	15884	A	CYTB	Non-synonymous	A380T	0	1	0.136
Pre-terminal	R0	G	15884	A	CYTB	Non-synonymous	A380T	0	1	0.136
Pre-terminal	R0	G	15884	A	CYTB	Non-synonymous	A380T	0	1	0.136
Pre-terminal	R0	G	15884	A	CYTB	Non-synonymous	A380T	0	1	0.136
Pre-terminal	R0	G	15884	A	CYTB	Non-synonymous	A380T	0	1	0.136
Modern terminal	U	G	15884	A	CYTB	Non-synonymous	A380T	0	1	0.136
Modern terminal	U	G	15884	A	CYTB	Non-synonymous	A380T	0	1	0.136
Modern terminal	U	G	15884	A	CYTB	Non-synonymous	A380T	0	1	0.136
Modern terminal	U	G	15884	A	CYTB	Non-synonymous	A380T	0	1	0.136
Modern terminal	U	G	15884	A	CYTB	Non-synonymous	A380T	0	1	0.136
Modern terminal	U	G	15884	A	CYTB	Non-synonymous	A380T	0	1	0.136
Modern terminal	U	G	15884	A	CYTB	Non-synonymous	A380T	0	1	0.136
Modern terminal	U	G	15884	A	CYTB	Non-synonymous	A380T	0	1	0.136
Modern terminal	U	G	15884	A	CYTB	Non-synonymous	A380T	0	1	0.136
Modern terminal	U	G	15884	A	CYTB	Non-synonymous	A380T	0	1	0.136
Modern terminal	U	G	15884	A	CYTB	Non-synonymous	A380T	0	1	0.136
Modern terminal	U	G	15884	A	CYTB	Non-synonymous	A380T	0	1	0.136
Modern terminal	U	G	15884	C	CYTB	Non-synonymous	A380P	0.167	0.06	0.292
Modern terminal	U	G	15884	C	CYTB	Non-synonymous	A380P	0.167	0.06	0.292
Modern terminal	U	G	15884	C	CYTB	Non-synonymous	A380P	0.167	0.06	0.292
Pre-terminal	U	G	15884	A	CYTB	Non-synonymous	A380T	0	1	0.136
Pre-terminal	U	G	15884	A	CYTB	Non-synonymous	A380T	0	1	0.136
Pre-terminal	U	G	15884	A	CYTB	Non-synonymous	A380T	0	1	0.136
Pre-terminal	U	G	15884	A	CYTB	Non-synonymous	A380T	0	1	0.136
Modern terminal	JT	G	15884	A	CYTB	Non-synonymous	A380T	0	1	0.136
Modern terminal	JT	G	15884	A	CYTB	Non-synonymous	A380T	0	1	0.136
Modern terminal	JT	G	15884	A	CYTB	Non-synonymous	A380T	0	1	0.136
Modern terminal	JT	G	15884	A	CYTB	Non-synonymous	A380T	0	1	0.136
Modern terminal	JT	G	15884	A	CYTB	Non-synonymous	A380T	0	1	0.136
Modern terminal	JT	G	15884	A	CYTB	Non-synonymous	A380T	0	1	0.136
Modern terminal	JT	G	15884	A	CYTB	Non-synonymous	A380T	0	1	0.136
Pre-terminal	JT	G	15884	A	CYTB	Non-synonymous	A380T	0	1	0.136
Pre-terminal	JT	G	15884	A	CYTB	Non-synonymous	A380T	0	1	0.136
Pre-terminal	JT	G	15884	A	CYTB	Non-synonymous	A380T	0	1	0.136
Pre-terminal	JT	G	15884	A	CYTB	Non-synonymous	A380T	0	1	0.136
Pre-terminal	JT	G	15884	A	CYTB	Non-synonymous	A380T	0	1	0.136
Pre-terminal	JT	G	15884	A	CYTB	Non-synonymous	A380T	0	1	0.136
Pre-terminal	JT	G	15884	A	CYTB	Non-synonymous	A380T	0	1	0.136
Modern terminal	N1	G	15884	A	CYTB	Non-synonymous	A380T	0	1	0.136
Modern terminal	N1	G	15884	A	CYTB	Non-synonymous	A380T	0	1	0.136
Modern terminal	N1	G	15884	A	CYTB	Non-synonymous	A380T	0	1	0.136

Modern terminal	N1	G	15884	A	CYTB	Non-synonymous	A380T	0	1	0.136
Modern terminal	N1	G	15884	A	CYTB	Non-synonymous	A380T	0	1	0.136
Modern terminal	X	G	15884	C	CYTB	Non-synonymous	A380P	0.167	0.06	0.292
Modern terminal	R0	C	15885	T	CYTB	Non-synonymous	A380V	0.02	0.06	0.147
Modern terminal	R0	C	15885	T	CYTB	Non-synonymous	A380V	0.02	0.06	0.147
Modern terminal	R0	C	15885	T	CYTB	Non-synonymous	A380V	0.02	0.06	0.147
Ancient terminal	U	C	15885	T	CYTB	Non-synonymous	A380V	0.02	0.06	0.147

* A small number of variants did not retrieve significant values using PolyPhen-2
